# Visible-light-mediated site-selective C(sp^2^)–H alkylation of tropones facilitates semi-synthesis of cephafortunoids A and B†

**DOI:** 10.1039/d5sc01006c

**Published:** 2025-04-10

**Authors:** Qi-Xiang Zeng, Cheng-Yu Zheng, Zhan-Peng Ge, Jin-Xin Zhao, Jian-Min Yue

**Affiliations:** a State Key Laboratory of Drug Research, Shanghai Institute of Materia Medica, Chinese Academy of Sciences 555 Zuchongzhi Road Shanghai 201203 China jxzhao@simm.ac.cn jmyue@simm.ac.cn; b University of Chinese Academy of Sciences No. 19A Yuquan Road Beijing 100049 China

## Abstract

The synthesis of functionalized tropones constitutes an underexplored chemical space, primarily due to the intrinsic structural properties of the aromatic nucleus. This predicament has impeded extensive investigation into their potential applications in organic and medicinal chemistry. Here, we report a mild and straightforward visible-light-mediated protocol for the α-site-selective C(sp^2^)–H alkylation of tropones, employing unactivated secondary amines as alkylating agents. This method yields up to 89% in 48 examples, and is significantly amenable to late-stage functionalization. The utility is showcased by the effective chemical transformation of fortunolide A into cephafortunoids A and B, representing the first synthetic entry to this unique class of C_20_*Cephalotaxus* troponoids. Significantly, this achievement reinforces the chemical feasibility of the newly hypothesized biosynthesis involving direct methylation *via* radical *S*-adenosylmethionine (SAM)-dependent methyltransferases.

## Abstract 2

An efficient visible-light-driven method for achieving α-site-selective C–H alkylation of tropones was developed. It was applied to transform fortunolide A into cephafortunoids A and B, marking the first synthesis of C_20_*Cephalotaxus* troponoids.

## Graphical abstract

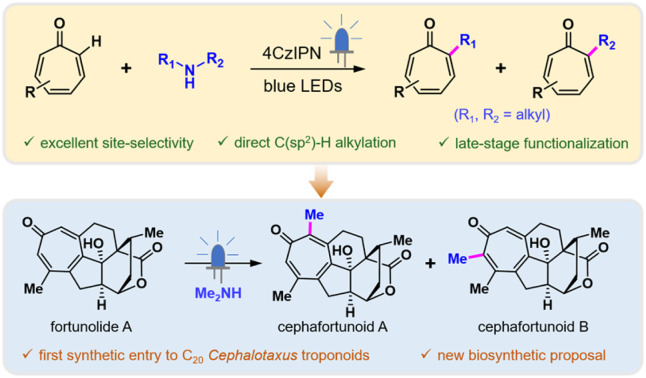

## 1 Introduction

Late-stage C–H functionalization provides a means to introduce important chemical groups and/or achieve modifications without disrupting the overall molecular integrity,^1–4^ and has thus emerged as a transformative tool widely utilized in natural product synthesis^5^ and drug discovery.^6^ Direct alkylation, particularly methylation,^7,8^ is experiencing a surge of interest in the realm of medicinal chemistry,^9–14^ due to its significant impact on drug metabolism and pharmacokinetic characteristics. The development of innovative alkylation methodologies continues to be a highly sought-after endeavor in the scientific community.

**Scheme 1:**
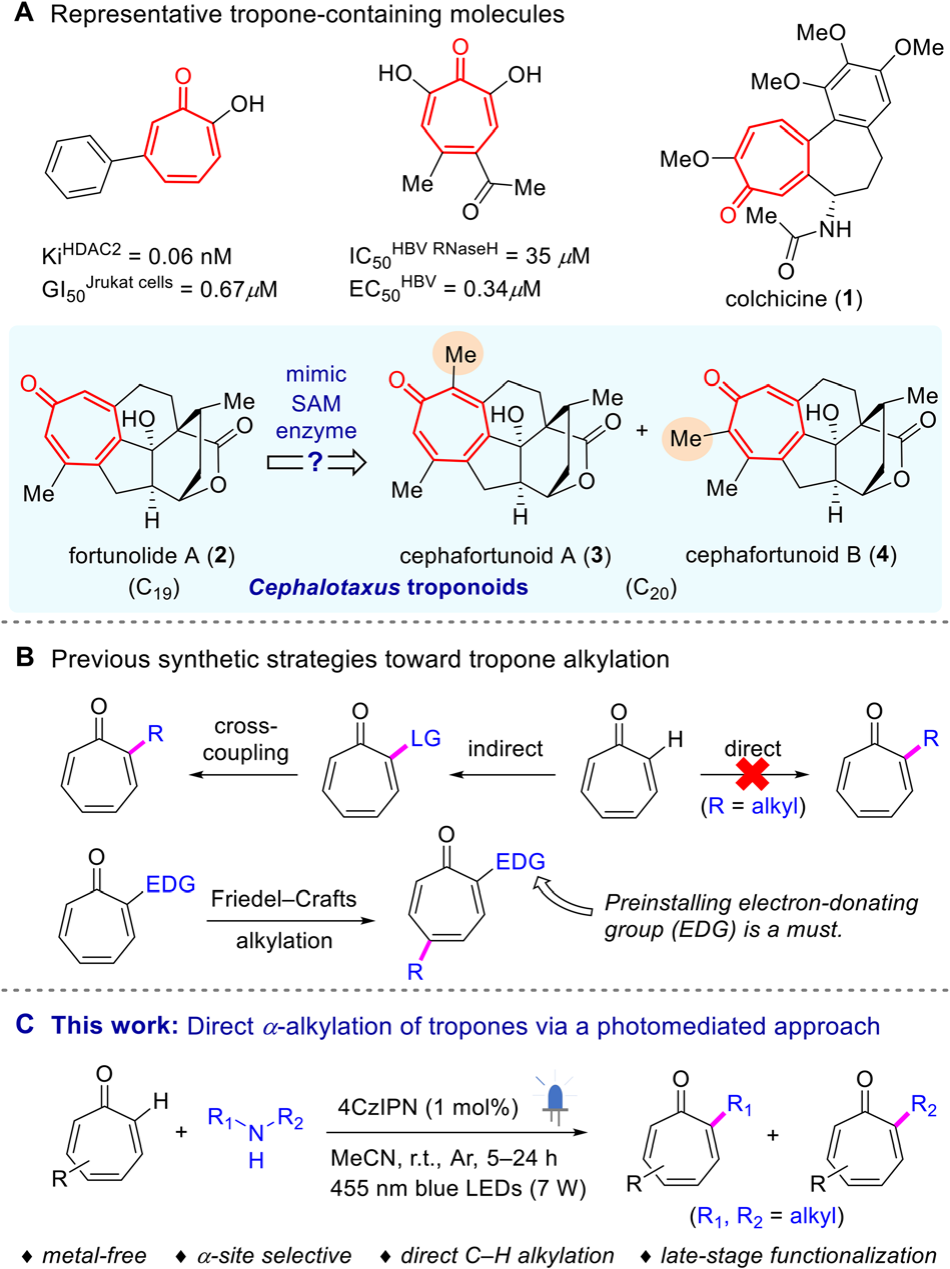
Background information and innovation of this work.

Tropone, a distinct non-benzenoid seven-membered aromatic group, can be encountered in a number of medicinally important molecules (Scheme 1A) that exhibit diverse biological properties, such as inhibition of histone deacetylase (HDAC),^15^ hepatitis B virus Ribonuclease H (HBV RNaseH),^16^ hepatitis C virus NS3 helicase,^17^*etc.* It can also be found embedded in a variety of natural products (Scheme 1A).^18–21^ Among them, colchicine (1), an alkaloid derived from the plant *Colchicum autumnale*, is the most extensively studied one and has been clinically used to treat gout and familial Mediterranean fever.^22^*Cephalotaxus* troponoids (C_19_), *e.g.* fortunolide A (2), a class of cephalotane norditerpenoids known for their broad-spectrum cytotoxicity against various tumor cell lines, constitute another important family of tropone-containing natural products.^23,24^ Noticeably, these diterpenoids have stimulated intense synthetic studies in recent years.^24–27^ In 2020, Hua and co-workers reported two unique C_20_*Cephalotaxus* troponoids, cephafortunoids A (3) and B (4), featuring an additional methyl at the α-position of the tropone core found in 2.^28^ To date, the chemical syntheses of cephafortunoids A and B have not been reported yet. Inspired by the methylating agent found in nature, *S*-adenosylmethionine (SAM),^29,30^ we postulated whether the α-methyl groups in these tropone-containing molecules could be introduced through direct methylation, starting from fortunolide A.

However, the current chemical repertoire for tropone functionalization is rather restricted, potentially owing to the inherent tendency of the nonbenzenoid dearomative cycloaddition reactions.^31,32^ Only a few alkylation methods for tropones have been documented hitherto (Scheme 1B),^33–37^ primarily relying on indirect strategies that involve the prior introduction of leaving groups. In addition, Friedel–Crafts like alkylation of tropones typically necessitates the preinstallation of an electron-donating group (EDG).^38^ In this scenario, the direct α-alkylation of tropones remains an unrealized goal.

In recent years, photoredox reactions have rapidly developed into a powerful tool for achieving late-stage C–H functionalization.^39–42^ However, upon exposure to light irradiation, tropones are prone to undergoing dimerization *via* cycloadditions. Consequently, regarding photocatalyzed alkylation of tropones, a significant challenge arises in suppressing the tendency for dimerization.^43–48^ Herein, in connection with our ongoing research studies on photoinduced reactions,^43,49^ we reported the first α-site-selective C(sp^2^)–H alkylation of tropones through a visible-light-mediated method (Scheme 1C), using unactivated secondary amines as mild alkylating agents.

## 2 Results and discussion

### 2.1. Reaction development

Amines are known to be transformed into α-aminoalkyl radicals under visible-light irritation *via* single electron oxidation.^50^ Given that acidic conditions favor higher-order cycloadditions in tropones,^44,47^ employing basic amines can mitigate their inherent dimerization tendency, thereby rendering amines ideal alkylating agents for tropones. A preliminary evaluation of amine substrates revealed that secondary amines provided optimal efficiency in the radical alkylation pathway (Scheme S1, ESI†). In contrast, primary amines predominantly underwent competitive amination, while tertiary amines exhibited slightly compromised yields likely due to steric constraints. We thus commenced our studies with tropone (5a) and dimethylamine (6a) as model substrates, as summarized in Tables S1–S3, ESI.† After extensive investigation of various factors, including photocatalysts, solvents, light sources, and the reaction atmosphere, the optimal conditions for this reaction were identified. Specifically, a combination of 5a (1 equiv.), 6a (2 equiv.), and the photocatalyst 1,2,3,5-tetrakis(carbazol-9-yl)-4,6-dicyanobenzene (4CzIPN, 1 mol%) in MeCN, under irradiation of 7 W 455 nm LEDs and an argon atmosphere for 10 h at room temperature efficiently yielded the desired α-methylated product 7a in 79% yield (Table 1, entry 1). Attempts using alternative photocatalysts, including 4DPAIPN, [Ru(bpz)_3_](PF_6_)_2_, thioxanthone, 4CzPN, and eosin Y, resulted in either low or negligible yields (entries 2–6). A solvent screening revealed that acetonitrile outperformed acetone, THF, and benzonitrile (entries 7–9), while the utilization of DMSO (entry 10) as the solvent led to the decomposition of the starting materials. The light source screening indicated that the reactions exhibited high sensitivity to different wavelengths of light, ultimately identifying 455 nm LEDs as the optimal choice (entries 11–15). Further control experiments demonstrated the critical roles of the photocatalyst and light. There was no desired product formed in the absence of either photocatalysts or light, even when heated to 60 °C (entries 16–18).

**Table 1:** Initial investigations and optimization of reaction conditions

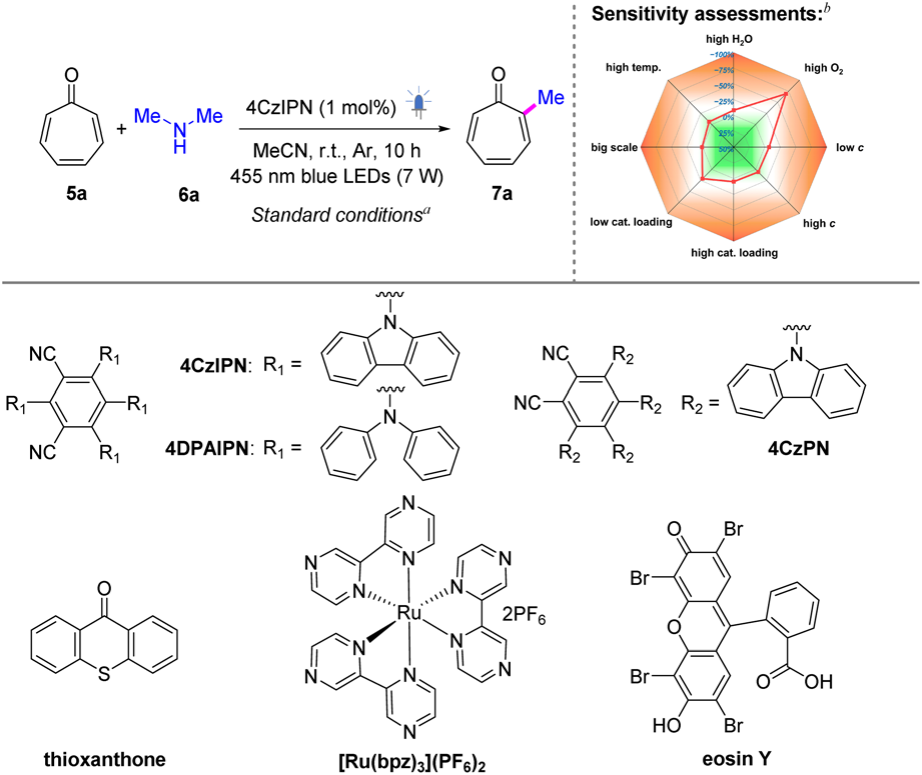		
Entry	Deviation from the standard conditions	Yield^c^ (%)
1	None	79
2	4DPAIPN	N.D.
3	[Ru(bpz)_3_](PF_6_)_2_	11
4	Thioxanthone	Dimers^d^
5	4CzPN	54
6	Eosin Y	N.D.
7	Acetone	13
8	THF	74
9	PhCN	69
10	DMSO	Decomposed
11	365 nm LEDs	Decomposed
12	395 nm LEDs	43
13	425 nm LEDs	63
14	500 nm LEDs	N.D.
15	White light	26
16	No catalyst	N.D.
17	Dark	N.D.
18	Dark, 60 °C	N.D.

a Standard conditions: 5a (0.283 mmol, 1.0 equiv.), 6a (0.566 mmol, 2.0 equiv.), and 4CzIPN (2.2 mg, 1 mol%) in 2.2 mL MeCN at room temperature under an argon atmosphere and irradiation of 7 W 455 nm LEDs for 10 h.

b Detailed information about the sensitive assessment is listed in Table S4, ESI.

c Isolated yields are reported.

d Only [6 + 4], [6 + 2], and [4 + 2] cycloadducts were obtained. See the ESI for details. N.D. = not detected, LED = light-emitting diode, MeCN = acetonitrile, THF = tetrahydrofuran, PhCN = benzonitrile, and DMSO = dimethyl sulfoxide.

To evaluate the robustness and reproducibility of the newly developed method, a condition-based sensitivity analysis was performed (Tables 1 and S4, ESI†).^51^ Notably, the reaction exhibited tolerance to variations in water content, temperature (temp.), concentration (*c*) levels, and catalyst loading. Additionally, the reaction could be successfully scaled up without compromising the isolated yield. However, the presence of oxygen was detrimental to the transformation, emphasizing the necessity of performing the reaction under an argon atmosphere.

### 2.2. Evaluation of substrate scope

**Scheme 2:**
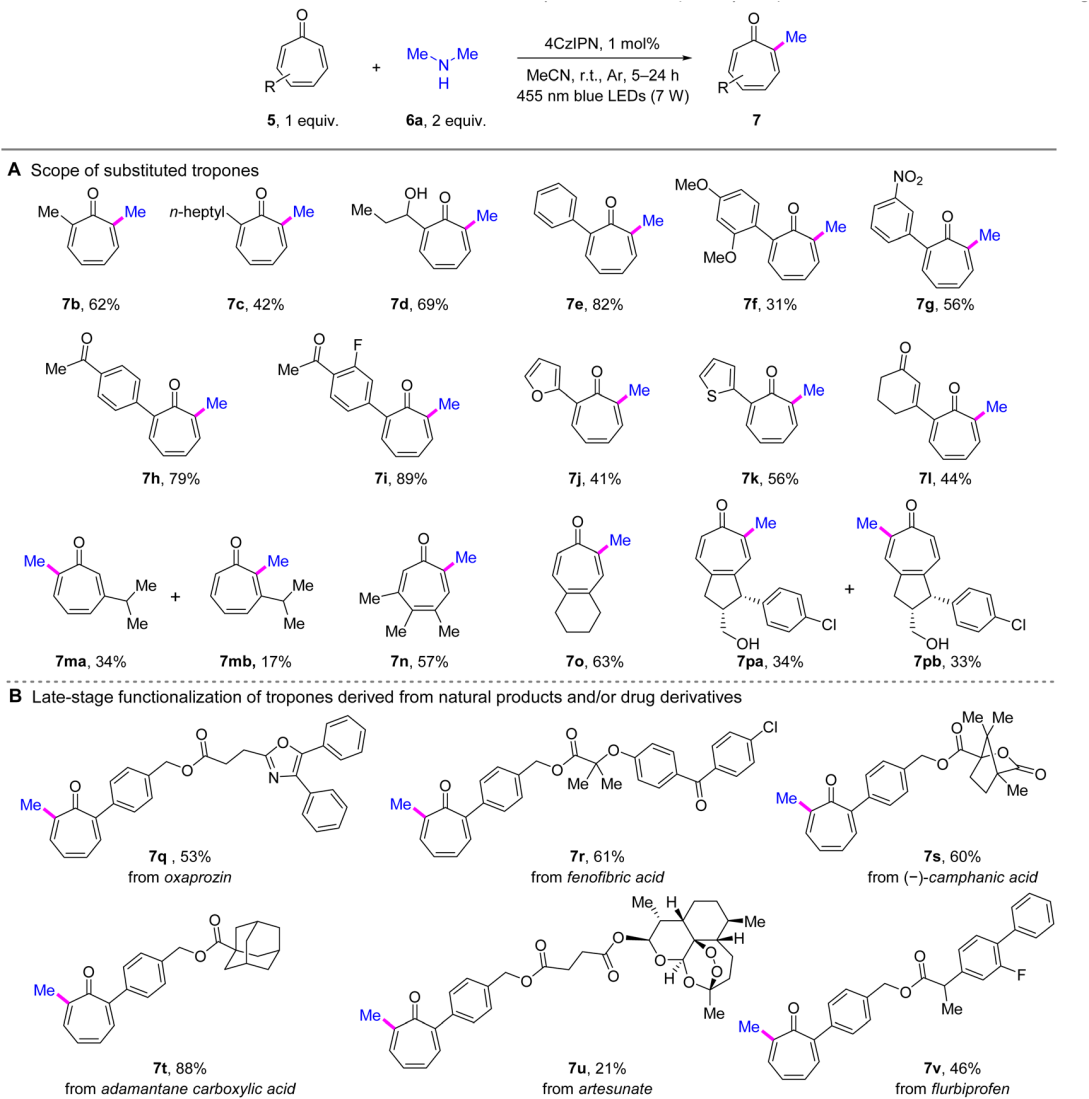
Scope of tropones for α-methylation of tropones. Reaction conditions: entry 1 in Table 1, irradiation time = 5–24 h. See the ESI† for details. Isolated yields are reported.

With the optimized reaction conditions in hand, we next embarked on the evaluation of substrate scope. First, the scope of tropones was explored by performing methylation on different substituted tropones using dimethylamine (6a) (Scheme 2A). As a result, an array of α-alkylated tropones exhibited satisfactory performance (7b–7d). Compared with 7a and 7b, the α-heptyl-substituted product 7c exhibited a decreased yield of 42%, indicating the potential impact of steric hindrance. Interestingly, the active hydroxyl group was well tolerated, as evidenced by the favorable yield (69%) achieved with the hydroxylated propyl substrate (7d). The α-arylated tropones were also compatible with the developed conditions (7e–7k). A clear electronic effect was observed among substituted phenyl derivatives. EDGs exemplified by the 2,4-dimethoxy substituent in 7f, exerted a detrimental effect on the reaction outcome. Conversely, electron-withdrawing groups (EWGs) markedly enhanced the reaction efficiency, with the dual EWG-substituted phenyl tropone 7i demonstrating optimal performance (89% yield). However, the nitro-containing product 7g showed anomalous behavior, with yield reduction likely attributable to the photosensitive nature of its nitroarene moiety. Significantly, heteroaromatic rings, including furan (7j) and thiophene (7k), were left unperturbed, yielding satisfactory results, thereby further enhancing the versatility of the reaction. Furthermore, the developed methodology successfully accommodated tropones functionalized with an enone moiety, affording product 7l in moderate yield. We next examined the reactivity of tropones lacking α-substituents (7m–7p). Methylation of 3-isopropyltropone yielded two regioisomers (7ma and 7mb), with 7ma predominating as the primary product, demonstrating significant steric control by the β-substituent. Notably, methylation of the 3,4,5-trisubstituted tropone (7n) occurred selectively at a position away from the sterically congested region. In contrast, the 4,5-disubstituted derivatives (7o and 7pa/b) exhibited diminished steric control over regioselectivity. Specifically, the symmetric substrate afforded the α-methylated product (7o)exclusively , whereas the unsymmetric analogue yielded near-equimolar amounts of both regioisomers (7pa and 7pb).

This method was also demonstrated in the late-stage C–H methylation of natural product and/or drug derivatives. As illustrated in Scheme 2B, the methylation of tropones derived from oxaprozin (7q), fenofibric acid (7r), (–)-camphanic acid (7s), adamantane carboxylic acid (7t), artesunate (7u), and flurbiprofen (7v) was all successfully achieved under the standard conditions. Notably, the adamantane-containing substrate (7t) stood out with an exceptional yield of 88%. These results demonstrated the outstanding functional group tolerance of the developed method.

**Scheme 3:**
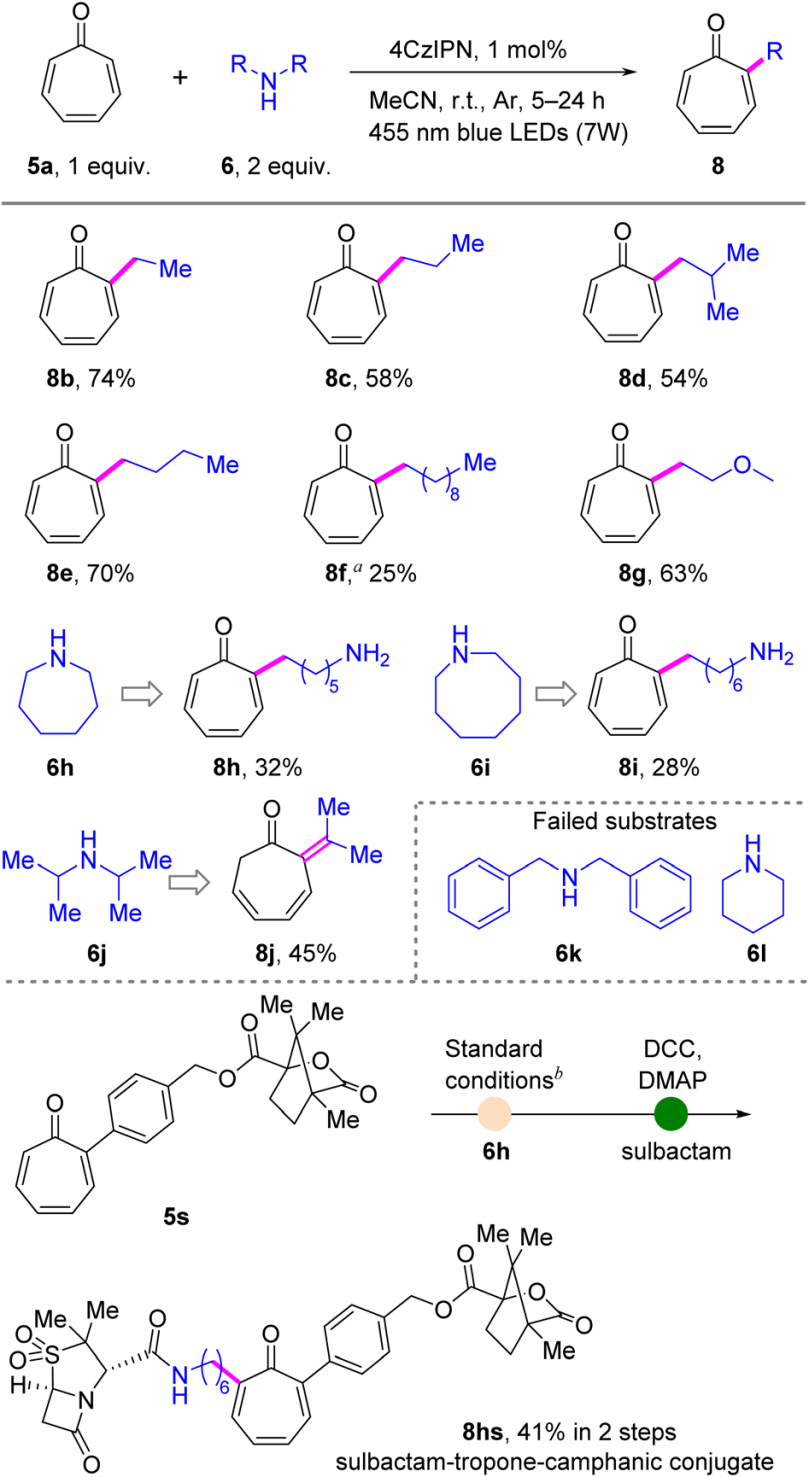
Scope of symmetric secondary amines for α-alkylation of tropones. Reaction conditions: entry 1 in Table 1, irradiation time = 5–24 h. See the ESI† for details. Isolated yields are reported.^*a*^ 0.283 mmol tropone (5a) and 0.283 mmol didecylamine (6f) were used due to the poor solubility of 6f in MeCN.^*b*^ 0.1 mmol 5s and 0.1 mmol azocane (6h) were used. DCC = *N*,*N*′-dicyclohexylcarbodiimide and DMAP = 4-dimethylaminopyridine.

Encouraged by the successful α-site-selective C–H methylation of tropones, we further ventured into direct C–H alkylation of tropones. Initially, symmetric secondary amines were used as alkylating reagents (Scheme 3), resulting in the formation of a series of α-alkylated tropones (8b–8f) in moderate to good yields (up to 74%). Moreover, the methoxy group (8g) could effectively participate in the alkylation process. However, dibenzylamine (6k) failed to give any desired alkylated products. Interestingly, some cyclic amines, such as azepane (6h) and azocane (6i), reacted smoothly with 5a, leading to the formation of the corresponding α-aminoalkyl tropones, though piperidine (6l) proved ineffective in this process. Tropone-containing natural product derivative 5s could also effectively react with azepane (6h) to generate a primary amine, which served as a versatile linker, facilitating the connection of various active sites through tropone that acts as a bridging molecule, thereby yielding a drug conjugate (8hs) in an acceptable yield. Interestingly, sterically hindered amine, diisopropylamine (6j), was also effective in engaging in the reaction process, yielding an unusual dearomatized product, 2-(propan-2-ylidene)cyclohepta-3,5-dien-1-one (8j), which is characterized by an exocyclic double bond.

**Scheme 4:**
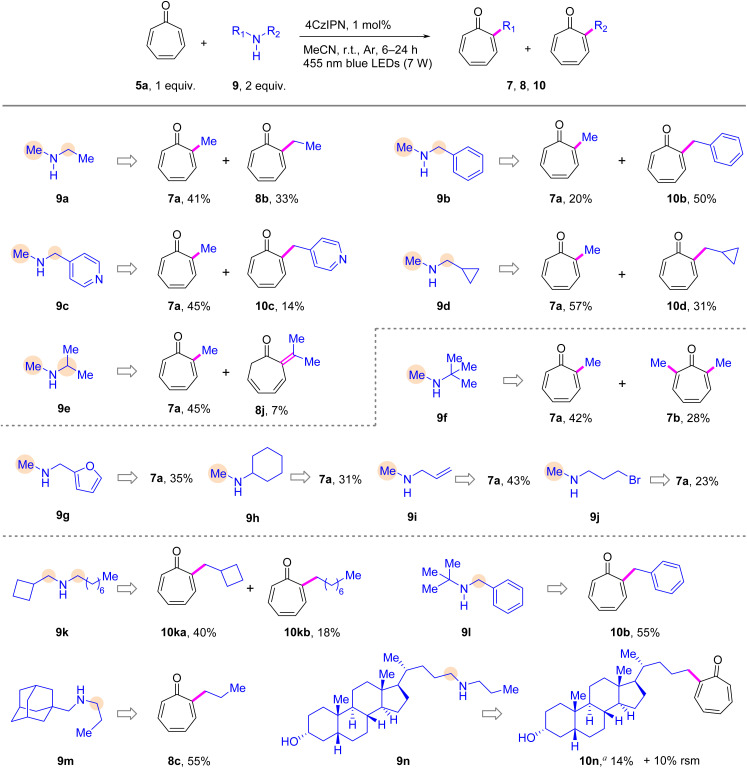
Scope of asymmetric secondary amines for α-alkylation of tropones. Reaction conditions: entry 1 in Table 1, irradiation time = 6–24 h. See the ESI† for details. Isolated yields are reported.^*a*^ 0.849 mmol 5a and 0.283 mmol 9n were used in MeCN/THF/DCM (5.7 mL/0.57 mL/1.2 mL). rsm = recovered starting material.

Furthermore, a range of asymmetric secondary amines were tested (Scheme 4). Initially, we utilized a diverse selection of methylamines as alkylating reagents (9a–9j). Considering the inherent presence of two reactive sites in these amines, it was anticipated that two distinct alkylation products would be formed, as exemplified by cases involving ethyl (9a), benzyl (9b), pyridine-4-ylmethyl (9c), cyclopropylmethyl (9d) and isopropyl (9e) methylamines. Notably, it was observed that the yield of α-methyl tropone 7a generally exceeded that of the resulting α-alkyl tropone, which can be attributed to the advantage conferred by the statistically more abundant methylene radicals resulting from the removal of any one of the three hydrogens in the methyl group in this reaction. However, benzylmethylamine 9b deviated from this trend, producing 7a and α-benzyl tropone 10b in a ratio of 1 : 2.5. This result demonstrates the superior reactivity of the benzyl group compared to methyl, likely attributable to the benzyl radical's enhanced nucleophilicity and extended lifetime due to resonance stabilization. This observation appears paradoxical given that the symmetrical dibenzylamine 6k does not undergo reaction with tropone probably owing to the lower basicity of 6k in comparison with 9b.^52^ The reduced basicity of 6k arises from the substitution of two benzyl groups, which delocalize the lone-pair electrons of N. Nevertheless, this transformation offers an alternative pathway for tropone benzylation. Furthermore, despite the presence of bi-reactive sites for isopropylmethylamine 9e, only a minor amount of the dearomatized product 8j was obtained. In particular, when *tert*-butylmethylamine (9f) was reacted with 5a, in addition to the methylation product 7a, dimethyl tropone 7b was also obtained. Regarding furfuryl (9g), cyclohexyl (9h), allyl (9i), and 3-bromopropyl (9j) methylamines, α-methyl tropone 7a was obtained as the exclusive product. This selectivity arose because of (1) the decreased nucleophilicity of furfuryl (9g) and allyl radicals (9i); (2) the steric bulk of cyclohexyl in 9h prevented alternative addition pathways; and (3) the labile bromide in 9j initiated competing radical processes, which introduced complexity into the reaction mixture. Additionally, we conducted tests on the reactivity of a few challenging asymmetric secondary amines (9k–9m) containing bulky functional groups. Gratifyingly, 9k proved to be an effective alkylation partner, producing α-cyclobutylmethyl tropone 10ka and α-*n*-octyl tropone 10kb in yields of 40% and 18%, respectively. Benzyl-*tert*-butylamine 9l also demonstrated efficient reactivity with 5a, yielding the sole benzylated product 10b in an acceptable yield. In the case of 9m, the presence of a bulky adamantane moiety resulted exclusively in the formation of α-*n*-propyl tropone 8c. Furthermore, by adjusting the solvent composition to enhance solubility, steroid-deriving amine 9n was successfully converted into the steroid-tropone coupled product 10n, albeit with a relatively low yield.

### 2.3. Investigations of the mechanism

**Scheme 5:**
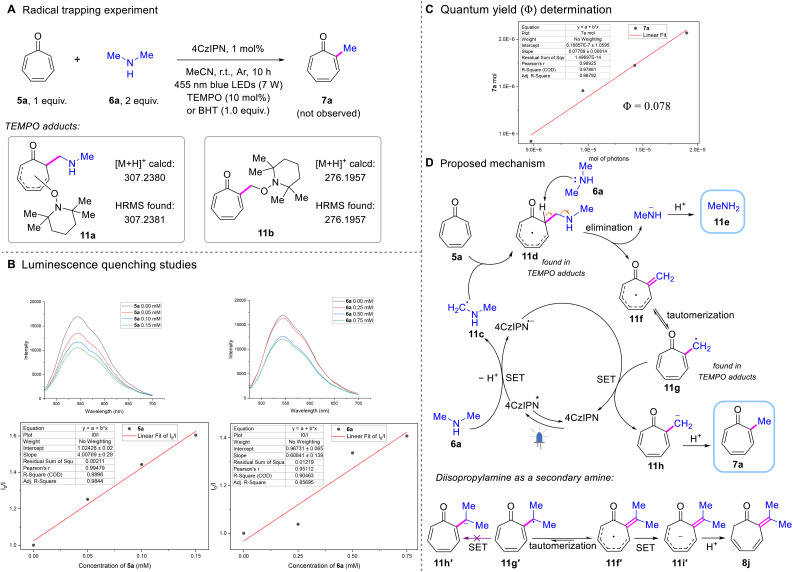
Mechanistic studies and the proposed reaction mechanism. TEMPO = (2,2,6,6-tetramethylpiperidin-1-yl)oxyl, BHT = butylated hydroxytoluene, and SET = single-electron transfer.

To gain insight into the reaction mechanism of the developed chemistry, we conducted several control experiments. To begin with, the reaction between tropone 5a and dimethylamine 6a was performed under standard conditions with the addition of radical scavengers (see the ESI† for details). Using BHT as a radical trapping reagent, total suppression of the reaction was observed, confirming the involvement of a radical reaction process. To capture the intermediates, radical scavenger TEMPO was added, resulting in the formation of two TEMPO trapping adducts, 11a and 11b (Scheme 5A), as detected by high resolution mass spectrometry (HRMS), indicating that an amine-tropone-adduct radical (11d) and a methylene radical (11g) were possibly generated during the reaction process. Subsequent Stern–Volmer quenching studies (Scheme 5B) demonstrated that both tropone 5a and dimethylamine 6a effectively quenched the excited state of the photocatalyst 4CzIPN*, albeit through distinct mechanisms. The observed quenching by tropone 5a, consistent with its extended conjugated system, was mechanistically assigned to an energy transfer (EnT) process from photoexcited 4CzIPN*. This EnT pathway was further substantiated by the facile cycloaddition of tropone 5a observed under amine-free conditions, a transformation that necessitates energy input from the photocatalyst. Notably, the introduction of a secondary amine effectively suppressed this competing cycloaddition pathway, redirecting the reaction toward the desired alkylation. Additionally, the quenching observed with dimethylamine 6a suggests a single-electron transfer (SET) mechanism between the excited state of 4CzIPN* and dimethylamine 6a. To investigate the influence of light, we then implemented a light on/off experiment for the model reaction (see the ESI† for details). The graphical analysis revealed that continuous irradiation is essential for this reaction, as no conversion occurred during the dark periods. The yield obtained under interval irradiation was significantly inferior to that achieved with continuous irradiation, suggesting that the active intermediate necessitated uninterrupted light exposure. Whenever the irradiation was paused, the active intermediate would be quenched spontaneously, preventing its regeneration during the subsequent light-on period. This underscores the necessity of continuous irradiation for sustaining the active intermediate and achieving maximum reaction yield. Even though, this observation could not conclusively rule out the potential involvement of a radical chain process.^53,54^ Quantum-yield measurement was further performed to provide mechanistic clarification. The measured quantum yield (*Φ*) of 0.078 provides evidence against a chain process, as this value is significantly below the threshold (*Φ* ≥ 1) required for radical chain reactions (Scheme 5C, see the ESI† for details). This quantitative assessment further solidifies the non-chain nature of the photoredox transformation.

Based on the experimental results presented above and insights from previous studies about amines as alkyl radical equivalents,^55–58^ along with the naturally occurring SAM methylating mechanism,^29^ a plausible mechanism was proposed (Scheme 5D). First, the photosensitizer 4CzIPN can be excited to its excited state 4CzIPN* under blue-light irradiation (*λ* = 455 nm). The latter acquires one electron (1e^−^) from dimethylamine 6a*via* a SET process, resulting in the formation of radical anion 4CzIPN˙^−^ and α-aminoalkyl radical 11c*via* loss of a proton. Subsequently, radical 11c engages in a nucleophilic attack on tropone 5a, forming the amine-tropone-adduct radical 11d. The α-site-specificity might be attributed to the stability of the resulting radical 11d, which is enhanced by the extensive delocalized π-system. Moreover, the acidity of the α-C(sp^3^)–H in the ketone would facilitate the following base-initiated elimination, catalyzed by dimethylamine 6a. This process leads to the formation of the exocyclic double bond-containing radical intermediate 11f, accompanied by the release of methylamine 11e as a reaction product. As shown in Scheme 3, the production of primary amines 8h and 8i offers compelling evidence for this process. Intermediate 11f undergoes tautomerization to form the methylene radical 11g, which subsequently acquires 1e^−^ from 4CzIPN˙^−^*via* the SET process, thereby restoring the photosensitizer to its ground state. In parallel, the tropone-methyl anion 11h is generated and subsequently undergoes protonation to produce the title product α-methyl tropone 7a. Significantly, the presence of the exocyclic intermediate was further substantiated by analysis of the plausible mechanism leading to the formation of 8j that bears an exocyclic double bond. It is assumed that the inert tertiary carbon radical 11g′ is unable to acquire 1e^−^ from 4CzIPN˙^−^ through the SET process, thereby rendering the tautomerization from 11f′ to 11g′ unfavorable. Consequently, the tropone radical intermediate 11f′ directly receives 1e^−^ from 4CzIPN˙^−^ to form the tropone anion 11i′, which then undergoes protonation to yield 8j. The preference of protonation at the α site of tropone anion 11i′ might arise from the stabilizing effect of the carbonyl group on the α-carbon anion. It is worth noting that, based on the reaction mechanism discussed above, more than one equivalent of 6a should theoretically suffice, given that the secondary amine functions dually as both an alkylation reagent and a base.

### 2.4. Application to semi-synthesis of cephafortunoids A/B and their proposed biogenesis

**Scheme 6:**
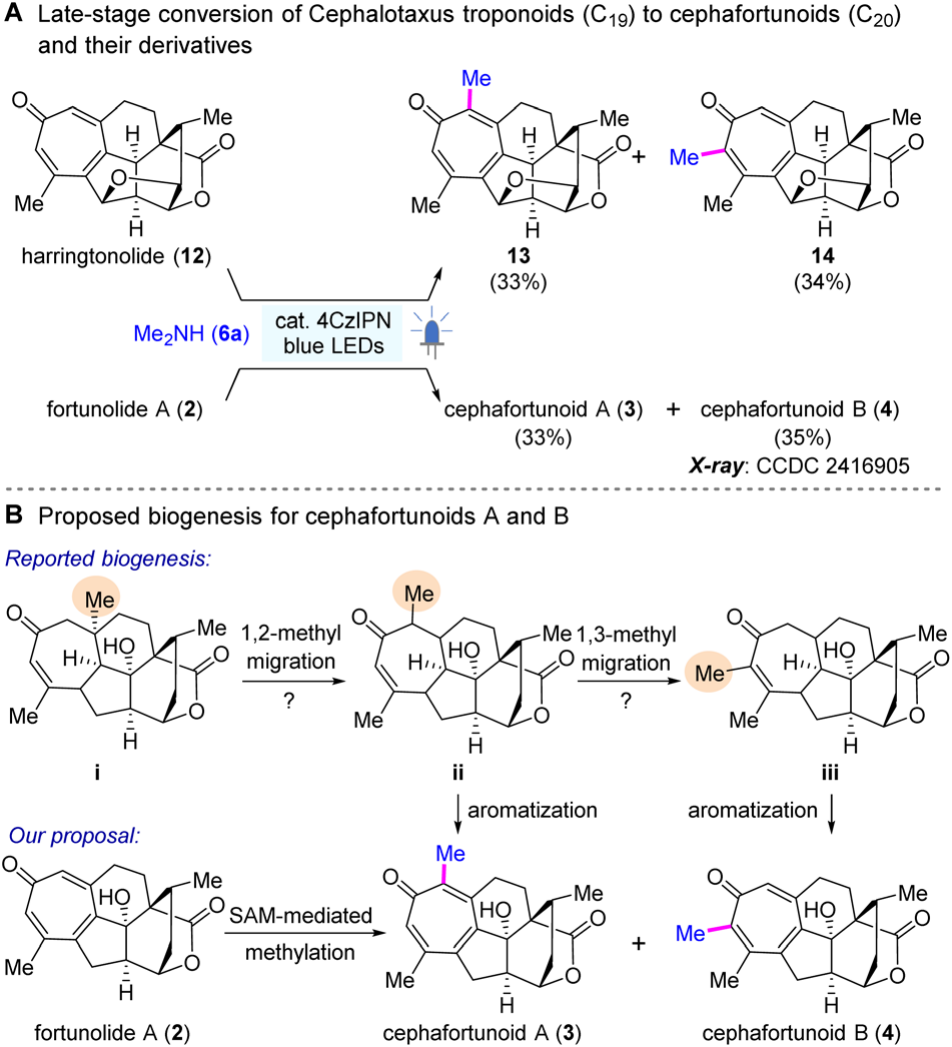
Application in late-stage conversion of *Cephalotaxus* troponoids (C_19_) to cephafortunoids A and B and reconsideration of their biosynthesis.

Next, to validate our initial postulation, we conducted direct methylation of *Cephalotaxus* troponoids (C_19_) (Scheme 6A), utilizing samples obtained through our extensive and sustained efforts in isolating cephalotane diterpenoids from *Cephalotaxus* plants.^59–66^ To our delight, harringtonolide (12) was successfully transformed into its α-methylated derivatives, 13 and 14, in appreciable yields, with a ratio of *ca.* 1 : 1. More importantly, we achieved a pioneering conversion of fortunolide A (2) to cephafortunoids A (3) and B (4) with the yields of 33% and 35%, respectively. All the spectrum data of compounds 3 and 4 were paralleled to those of the isolated ones (Tables S6–S9, ESI†).^28^ Although the NMR data of compound 4 matched roughly, likely owing to the low testing concentration,^67,68^ the structure of this compound was unambiguously confirmed by X-ray crystallography (CCDC 2416905, Table S5, ESI†).

The achievement of the successful late-stage transformation stimulated us to reexamine the biosynthetic hypothesis for cephafortunoids A (3) and B (4). In light of the lack of motivating factors for the initially proposed 1,2- (i → ii) and 1,3-(ii → iii) methyl migrations,^28^ it appears more plausible that compounds 3 and 4 arise through direct SAM-mediated methylation of the co-isolated fortunolide A (2) (Scheme 6B). Nevertheless, further endeavors in chemical biology are required to identify the relevant SAM enzyme and elucidate the precise mechanism.

## 3 Conclusions

In summary, we have developed a visible-light-mediated method for the direct C(sp^2^)–H α-alkylation of tropones under mild and easy-to-operate conditions. This method demonstrates remarkable site selectivity and robust functional group compatibility, achieving yields up to 89% in 48 examples. Leveraging this novel chemistry, we successfully accomplished the pioneering chemical conversion of fortunolide A (2) to cephafortunoids A (3) and B (4), thereby gaining access to this unique class of C_20_*Cephalotaxus* troponoids for the first time. This achievement leads to a reconsideration of the biosynthetic pathway for cephafortunoids A and B, which was hypothesized to involve direct radical SAM-mediated methylation. This finding not only fills a significant gap in late-stage modifications of tropones, but also establishes a cornerstone for future research into tropone-based pharmaceutical compounds and natural products.

## Data availability

General information, detailed experimental procedures, and characterization data for all new compounds can be found in the ESI.†

## Author contributions

Q.-X. Z.: data curation, investigation, methodology study, and writing – original draft. C.-Y. Z.: advise on mechanism study, visualization. Z.-P. G.: provide the isolated natural products, funding acquisition. J.-X. Z: conceptualization, funding acquisition, supervision, writing – review and editing, project administration. J.-M. Y: conceptualization, funding acquisition, supervision, writing – review and editing, project administration.

## Conflicts of interest

There are no conflicts to declare.

## Supplementary Material

SC-016-D5SC01006C-s001

## References

[cit1] Kim K. E., Kim A. N., McCormick C. J., Stoltz B. M. (2021). J. Am. Chem. Soc..

[cit2] Das S. K., Das S., Ghosh S., Roy S., Pareek M., Roy B., Sunoj R. B., Chattopadhyay B. (2022). Chem. Sci..

[cit3] Khatua H., Das S., Patra S., Das S. K., Roy S., Chattopadhyay B. (2022). J. Am. Chem. Soc..

[cit4] Bisht R., Haldar C., Hassan M. M. M., Hoque M. E., Chaturvedi J., Chattopadhyay B. (2022). Chem. Soc. Rev..

[cit5] Abrams D. J., Provencher P. A., Sorensen E. J. (2018). Chem. Soc. Rev..

[cit6] Guillemard L., Kaplaneris N., Ackermann L., Johansson M. J. (2021). Nat. Rev. Chem..

[cit7] Aynetdinova D., Callens M. C., Hicks H. B., Poh C. Y. X., Shennan B. D. A., Boyd A. M., Lim Z. H., Leitch J. A., Dixon D. J. (2021). Chem. Soc. Rev..

[cit8] Schönherr H., Cernak T. (2013). Angew. Chem., Int. Ed..

[cit9] Parida S. K., Hota S. K., Kumar R., Murarka S. (2021). Chem.–Asian J..

[cit10] Jin J., MacMillan D. W. C. (2015). Nature.

[cit11] Chen X., Ye F., Luo X., Liu X., Zhao J., Wang S., Zhou Q., Chen G., Wang P. (2019). J. Am. Chem. Soc..

[cit12] Le C., Liang Y., Evans R. W., Li X., MacMillan D. W. C. (2017). Nature.

[cit13] Kim D., Lee G. S., Kim D., Hong S. H. (2020). Nat. Commun..

[cit14] Hu X., Zhao Y., He T., Niu C., Liu F., Jia W., Mu Y., Li X., Rong Z.-Q. (2024). Chem. Sci..

[cit15] Ononye S. N., VanHeyst M. D., Oblak E. Z., Zhou W., Ammar M., Anderson A. C., Wright D. L. (2013). ACS Med. Chem. Lett..

[cit16] Long K. R., Lomonosova E., Li Q., Ponzar N. L., Villa J. A., Touchette E., Rapp S., Liley R. M., Murelli R. P., Grigoryan A., Buller R. M., Wilson L., Bial J., Sagartz J. E., Tavis J. E. (2018). Antiviral Res..

[cit17] Najda-Bernatowicz A., Krawczyk M., Stankiewicz-Drogoń A., Bretner M., Boguszewska-Chachulska A. M. (2010). Bioorg. Med. Chem..

[cit18] Bentley R. (2008). Nat. Prod. Rep..

[cit19] Liu N., Song W., Schienebeck C. M., Zhang M., Tang W. (2014). Tetrahedron.

[cit20] Guo H., Roman D., Beemelmanns C. (2019). Nat. Prod. Rep..

[cit21] Wang D.-D., Zhang R., Tang L.-Y., Wang L.-N.-Q., Ao M.-R., Jia J.-M., Wang A.-H. (2024). Bioorg. Chem..

[cit22] Christos A., Zoi K., Charalampos K., Agathi-Rosa V., Achilleas Z., Fotios K., Vasilios K., Georgios G. (2018). Curr. Pharm. Des..

[cit23] Abdelkafi H., Nay B. (2012). Nat. Prod. Rep..

[cit24] Zhao J.-X., Ge Z.-P., Yue J.-M. (2024). Nat. Prod. Rep..

[cit25] Wiesler S., Sennari G., Popescu M. V., Gardner K. E., Aida K., Paton R. S., Sarpong R. (2024). Nat. Commun..

[cit26] Sun Z., Shu X., Ma F., Li A., Li Y., Jin S., Wang Y., Hu X. (2024). Angew. Chem., Int. Ed..

[cit27] Shao H., Ma Z.-H., Cheng Y.-Y., Guo X.-F., Sun Y.-K., Liu W.-J., Zhao Y.-M. (2024). Angew. Chem., Int. Ed..

[cit28] Li Y., Wang Y., Shao Z., Zhao C., Jing Q., Li D., Lin B., Jing Y., Li Z., Hua H. (2020). Bioorg. Chem..

[cit29] Bauerle M. R., Schwalm E. L., Booker S. J. (2015). J. Biol. Chem..

[cit30] Nicolet Y. (2020). Nat. Catal..

[cit31] Murelli R. P., Berkowitz A. J., Zuschlag D. W. (2023). Tetrahedron.

[cit32] Jessen N. I., McLeod D., Jørgensen K. A. (2022). Chem.

[cit33] Brunetti A., Garbini M., Gino Kub N., Monari M., Pedrazzani R., Zanardi C., Bertuzzi G., Bandini M. (2024). Adv. Synth. Catal..

[cit34] Haas D., Sustac-Roman D., Schwarz S., Knochel P. (2016). Org. Lett..

[cit35] Nicolaou K. C., Yu R., Lu Z., Alvarez F. G. (2022). J. Am. Chem. Soc..

[cit36] Cavazza M., Morganti G., Pietra F. (1984). J. Chem. Soc., Perkin Trans. 1.

[cit37] Brunetti A., Kiriakidi S., Garbini M., Monda G., Zanardi C., López C. S., Bertuzzi G., Bandini M. (2025). ACS Catal..

[cit38] Gallorini G., Kiriakidi S., Bellini S., López C. S., Bertuzzi G., Bandini M. (2024). Org. Lett..

[cit39] Yoon T. P., Ischay M. A., Du J. (2010). Nat. Chem..

[cit40] Mao E., MacMillan D. W. C. (2023). J. Am. Chem. Soc..

[cit41] Niu L., Liu J., Liang X.-A., Wang S., Lei A. (2019). Nat. Commun..

[cit42] Dewanji A., van Dalsen L., Rossi-Ashton J. A., Gasson E., Crisenza G. E. M., Procter D. J. (2023). Nat. Chem..

[cit43] Ge Z.-P., Fan Y.-Y., Deng W.-D., Zheng C.-Y., Li T., Yue J.-M. (2021). Angew. Chem., Int. Ed..

[cit44] Reingold I. D., Kwong K. S., Menard M. M. (1989). J. Org. Chem..

[cit45] Mori A., Wu S.-P., Kato N., Takeshita H. (1998). J. Chem. Soc., Perkin Trans. 1.

[cit46] Reingold I. D., Kowalski J. A., Cummings G. C., Gleiter R., Lange H., Lovell S., Kahr B., Aflatooni K., Burrow P. D., Gallup G. A. (2006). J. Phys. Org. Chem..

[cit47] Mukai T., Tezuka T., Akasaki Y. (1966). J. Am. Chem. Soc..

[cit48] Kende A. S. (1966). J. Am. Chem. Soc..

[cit49] Zheng C.-Y., Yue J.-M. (2023). Nat. Commun..

[cit50] Nakajima K., Miyake Y., Nishibayashi Y. (2016). Acc. Chem. Res..

[cit51] Schäfer F., Lückemeier L., Glorius F. (2024). Chem. Sci..

[cit52] Crampton M. R., Robotham I. A. (1997). J. Chem. Res., Synop..

[cit53] Cismesia M. A., Yoon T. P. (2015). Chem. Sci..

[cit54] Buzzetti L., Crisenza G. E. M., Melchiorre P. (2019). Angew. Chem., Int. Ed..

[cit55] Zou Y.-Q. and Xiao W.-J., in Visible Light Photocatal. Org. Chem., ed. C. Stephenson, T. Yoon and D. W. C. MacMillan, Wiley-VCH Verlag GmbH & Co. KGaA, 2018, ch. 4, pp. 93–127

[cit56] Zheng L., Jiang Q., Bao H., Zhou B., Luo S.-P., Jin H., Wu H., Liu Y. (2020). Org. Lett..

[cit57] Bao H., Zhou B., Luo S.-P., Xu Z., Jin H., Liu Y. (2020). ACS Catal..

[cit58] Ni Q., Zhou Y., Chen L., Liu Y. (2025). Org. Chem. Front..

[cit59] Ge Z.-P., Xu J.-B., Zhao P., Xiang M., Zhou Y., Lin Z.-M., Zuo J.-P., Zhao J.-X., Yue J.-M. (2024). Phytochemistry.

[cit60] Ge Z.-P., Zhou B., Zimbres F. M., Haney R. S., Liu Q.-F., Wu Y., Cassera M. B., Zhao J.-X., Yue J.-M. (2022). Org. Biomol. Chem..

[cit61] Ge Z.-P., Zhou B., Zimbres F. M., Cassera M. B., Zhao J.-X., Yue J.-M. (2022). Chin. J. Chem..

[cit62] Ge Z.-P., Liu H.-C., Wang G.-C., Liu Q.-F., Xu C.-H., Ding J., Fan Y.-Y., Yue J.-M. (2019). J. Nat. Prod..

[cit63] Zhao J.-X., Fan Y.-Y., Xu J.-B., Gan L.-S., Xu C.-H., Ding J., Yue J.-M. (2017). J. Nat. Prod..

[cit64] Fan Y.-Y., Xu J.-B., Liu H.-C., Gan L.-S., Ding J., Yue J.-M. (2017). J. Nat. Prod..

[cit65] Xu J.-B., Fan Y.-Y., Gan L.-S., Zhou Y.-B., Li J., Yue J.-M. (2016). Chem. – Eur. J..

[cit66] Ni G., Zhang H., Fan Y.-Y., Liu H.-C., Ding J., Yue J.-M. (2016). Org. Lett..

[cit67] Katsuyama I., Khalil A. A., Dunbar C., Zjawiony J. K. (2003). Spectrosc. Lett..

[cit68] Mitra A., Seaton P. J., Ali Assarpour R., Williamson T. (1998). Tetrahedron.

