# Supplementary material for: Visible-light-mediated site-selective C(sp2)–H alkylation of tropones facilitates semi-synthesis of cephafortunoids A and B
Source: Chem Sci. 2025 Apr 10;16(20):8836–44. doi: 10.1039/d5sc01006c (PMC12004080; doi:10.1039/d5sc01006c)
Supplement: SC-016-D5SC01006C-s001 [file SC-016-D5SC01006C-s001.pdf]

Supplementary Information (SI) for Chemical Science.
This journal is © The Royal Society of Chemistry 2025

Supplementary Information for

Visible-light-mediated site-selective $C(sp^2)$–H alkylation of tropones facilitates semi-synthesis of cephafortunoids A and B

Qi-Xiang Zeng,[ab] Cheng-Yu Zheng,[a] Zhan-Peng Ge,[a] Jin-Xin Zhao *[ab] and Jian-Min Yue *[ab]

[a] State Key Laboratory of Drug Research, Shanghai Institute of Materia Medica, Chinese Academy of Sciences, 555 Zuchongzhi Road, Shanghai 201203, China.

[b] University of Chinese Academy of Sciences, No. 19A Yuquan Road, Beijing 100049, China.

*Corresponding authors.

Emails: jxzhao@simm.ac.cn (J.-X. Zhao); jmyue@simm.ac.cn (J.-M. Yue)

Table of Contents

General Experimental 9
Optimization of the Reaction Conditions 10
Table S1 Optimization for light sources 10
Table S2 Optimization for photocatalyst 11
Table S3 Optimization for solvent 12
Scheme S1 Optimization for amine 12
Table S4 Sensitivity assessment of the C(sp^2)–H alkylation of tropones 13
Incompatible Substrates 14
Preparation of Starting Materials 15
General procedure A, B and C 15
Synthesis of **5c** 16
Synthesis of **5d** 17
Synthesis of **5e** 17
Synthesis of **5f** 18
Synthesis of **5g** 18
Synthesis of **5h** 19
Synthesis of **5i** 19
Synthesis of **5j** 20
Synthesis of **5k** 21
Synthesis of **5l** 21
Synthesis of **5n** 22
Synthesis of **5o** 23
Synthesis of **5p** 23
Synthesis of **S8** 24
Synthesis of **5q** 24
Synthesis of **5r** 25
Synthesis of **5s** 25
Synthesis of **5t** 26
Synthesis of **5u** 27
Synthesis of **5v** 28
Synthesis of **9k** 28
Synthesis of **9m** 29
Synthesis of **9n** 29
General Procedure for Tropone Alkylation 30
General procedure D for tropone alkylation 30
Synthesis and Characteristics of Alkylated Tropones 32
Compound **7a** 32
Compound **7b** 32
Compound **7c** 33
Compound **7d** 33
Compound **7e** 34
Compound **7f** 34

Compound **7g**...35
Compound **7h**...35
Compound **7i**...36
Compound **7j**...37
Compound **7k**...37
Compound **7l**...38
Compounds **7ma**/**7mb**...38
Compound **7n**...39
Compound **7o**...40
Compounds **7pa**/**7pb**...40
Compound **7q**...42
Compound **7r**...42
Compound **7s**...43
Compound **7t**...44
Compound **7u**...44
Compound **7v**...45
Compound **8b**...46
Compound **8c**...46
Compound **8d**...47
Compound **8e**...47
Compound **8f**...48
Compound **8g**...49
Compound **8h**...49
Compound **8i**...50
Compound **8j**...50
Compound **8hs**...51
Compound **9a** to **7a** and **8b**...52
Compound **9b** to **7a** and **10b**...52
Compound **9c** to **7a** and **10c**...53
Compound **9d** to **7a** and **10d**...53
Compound **9e** to **7a** and **8j**...54
Compound **9f** to **7a** and **7b**...54
Compound **9g** to **7a**...54
Compound **9h** to **7a**...55
Compound **9i** to **7a**...55
Compound **9j** to **7a**...55
Compound **9k** to **10ka** and **10kb**...56
Compound **9l** to **10b**...57
Compound **9m** to **8c**...57
Compound **9n** to **10n**...57
Alkylation of *Cephalotaxus* Troponoids...58
Compounds **13** and **14**...58
Compounds **3** and **4**...59
Mechanistic Studies...61

Radical capture experiment.....61
Light on and off experiment.....63
Luminescence quenching studies.....64
Quantum yield measurement.....65
NMR/MS Spectra.....69
Compound **5c** ^{1}H NMR (400 MHz, $CDCl_3$).....69
Compound **5c** ^{13}C NMR (100 MHz, $CDCl_3$).....70
Compound **5c** HRMS (ESI-TOF).....71
Compound **5f** ^{1}H NMR (600 MHz, $CDCl_3$).....72
Compound **5f** ^{13}C NMR (150 MHz, $CDCl_3$).....73
Compound **5f** HRMS (ESI-TOF).....74
Compound **5g** ^{1}H NMR (400 MHz, $CDCl_3$).....75
Compound **5g** ^{13}C NMR (100 MHz, $CDCl_3$).....76
Compound **5g** HRMS (ESI-TOF).....77
Compound **5h** ^{1}H NMR (400 MHz, $CDCl_3$).....78
Compound **5h** ^{13}C NMR (100 MHz, $CDCl_3$).....79
Compound **5h** HRMS (ESI-TOF).....80
Compound **5i** ^{1}H NMR (600 MHz, $CDCl_3$).....81
Compound **5i** ^{13}C NMR (150 MHz, $CDCl_3$).....82
Compound **5i** HRMS (ESI-TOF).....83
Compound **5j** ^{1}H NMR (600 MHz, $CDCl_3$).....84
Compound **5j** ^{13}C NMR (150 MHz, $CDCl_3$).....85
Compound **5j** HRMS (ESI-TOF).....86
Compound **5l** ^{1}H NMR (400 MHz, $CDCl_3$).....87
Compound **5l** ^{13}C NMR (100 MHz, $CDCl_3$).....88
Compound **5l** HRMS (ESI-TOF).....89
Compound **5n** ^{1}H NMR (600 MHz, $CDCl_3$).....90
Compound **5n** ^{13}C NMR (150 MHz, $CDCl_3$).....91
Compound **5n** HRMS (ESI-TOF).....92
Compound **5o** ^{1}H NMR (600 MHz, $CDCl_3$).....93
Compound **5o** ^{13}C NMR (150 MHz, $CDCl_3$).....94
Compound **5o** HRMS (ESI-TOF).....95
Compound **S8** ^{1}H NMR (600 MHz, $CDCl_3$).....96
Compound **S8** ^{13}C NMR (150 MHz, $CDCl_3$).....97
Compound **S8** HRMS (ESI-TOF).....98
Compound **5q** ^{1}H NMR (400 MHz, $CDCl_3$).....99
Compound **5q** ^{13}C NMR (150 MHz, $CDCl_3$).....100
Compound **5q** HRMS (ESI-TOF):.....101
Compound **5r** ^{1}H NMR (600 MHz, $CDCl_3$).....102
Compound **5r** ^{13}C NMR (150 MHz, $CDCl_3$).....103
Compound **5r** HRMS (ESI-TOF).....104
Compound **5s** ^{1}H NMR (600 MHz, $CDCl_3$).....105
Compound **5s** ^{13}C NMR (150 MHz, $CDCl_3$).....106
Compound **5s** HRMS (ESI-TOF).....107

Compound **5t** ^{1}H NMR (600 MHz, $CDCl_3$)....108
Compound **5t** ^{13}C NMR (150 MHz, $CDCl_3$)....109
Compound **5t** HRMS (ESI-TOF)....110
Compound **5u** ^{1}H NMR (600 MHz, $CDCl_3$)....111
Compound **5u** ^{13}C NMR (150 MHz, $CDCl_3$)....112
Compound **5u** HRMS (ESI-TOF)....113
Compound **5v** ^{1}H NMR (600 MHz, $CDCl_3$)....114
Compound **5v** ^{13}C NMR (150 MHz, $CDCl_3$)....115
Compound **5v** HRMS (ESI-TOF)....116
Compound **9k** ^{1}H NMR (600 MHz, $CDCl_3$)....117
Compound **9k** ^{13}C NMR (150 MHz, $CDCl_3$)....118
Compound **9k** HRMS (ESI-TOF)....119
Compound **9m** ^{1}H NMR (600 MHz, $CDCl_3$)....120
Compound **9m** ^{13}C NMR (150 MHz, $CDCl_3$)....121
Compound **9m** HRMS (ESI-TOF)....122
Compound **9n** ^{1}H NMR (400 MHz, $CDCl_3$)....123
Compound **9n** ^{13}C NMR (100 MHz, $CDCl_3$)....124
Compound **9n** HRMS (ESI-TOF)....125
Compound **7a** ^{1}H NMR (400 MHz, $CDCl_3$)....126
Compound **7a** ^{13}C NMR (125 MHz, $CDCl_3$)....127
Compound **7a** HRMS (ESI-TOF):....128
Compound **7c** ^{1}H NMR (600 MHz, $CDCl_3$)....129
Compound **7c** ^{13}C NMR (125 MHz, $CDCl_3$)....130
Compound **7c** HRMS (ESI-TOF)....131
Compound **7d** ^{1}H NMR (600 MHz, $CDCl_3$)....132
Compound **7d** ^{13}C NMR (125 MHz, $CDCl_3$)....133
Compound **7d** HRMS (ESI-TOF)....134
Compound **7e** ^{1}H NMR (600 MHz, $CDCl_3$)....135
Compound **7e** ^{13}C NMR (150 MHz, $CDCl_3$)....136
Compound **7e** HRMS (ESI-TOF)....137
Compound **7f** ^{1}H NMR (600 MHz, $CDCl_3$)....138
Compound **7f** ^{13}C NMR (150 MHz, $CDCl_3$)....139
Compound **7f** HRMS (ESI-TOF)....140
Compound **7g** ^{1}H NMR (400 MHz, $CDCl_3$)....141
Compound **7g** ^{13}C NMR (125 MHz, $CDCl_3$)....142
Compound **7g** HRMS (ESI-TOF)....143
Compound **7h** ^{1}H NMR (500 MHz, $CDCl_3$)....144
Compound **7h** ^{13}C NMR (125 MHz, $CDCl_3$)....145
Compound **7h** HRMS (ESI-TOF)....146
Compound **7i** ^{1}H NMR (600 MHz, $CDCl_3$)....147
Compound **7i** ^{13}C NMR (125 MHz, $CDCl_3$)....148
Compound **7i** ^{19}F NMR (471 MHz, $CDCl_3$)....149
Compound **7i** HRMS (ESI-TOF)....150
Compound **7j** ^{1}H NMR (600 MHz, $CDCl_3$)....151

Compound **7j** ^{13}C NMR (125 MHz, $CDCl_3$)....152
Compound **7j** HRMS (ESI-TOF)....153
Compound **7k** ^{1}H NMR (600 MHz, $CDCl_3$)....154
Compound **7k** ^{13}C NMR (150 MHz, $CDCl_3$)....155
Compound **7k** HRMS (ESI-TOF)....156
Compound **7l** ^{1}H NMR (600 MHz, $CDCl_3$)....157
Compound **7l** ^{13}C NMR (125 MHz, $CDCl_3$)....158
Compound **7l** HRMS (ESI-TOF)....159
Compound **7ma** and **7mb** ^{1}H NMR (600 MHz, $CDCl_3$)....160
Compound **7ma** and **7mb** ^{13}C NMR (150 MHz, $CDCl_3$)....161
Compound **7ma** and **7mb** HRMS (ESI-TOF)....162
Compound **7n** ^{1}H NMR (600 MHz, $CDCl_3$)....163
Compound **7n** ^{13}C NMR (125 MHz, $CDCl_3$)....164
Compound **7n** HRMS (ESI-TOF)....165
Compound **7o** ^{1}H NMR (600 MHz, $CDCl_3$)....166
Compound **7o** ^{13}C NMR (125 MHz, $CDCl_3$)....167
Compound **7o** HRMS (ESI-TOF)....168
Compound **7pa** ^{1}H NMR (600 MHz, $CDCl_3$)....169
Compound **7pa** ^{13}C NMR (125 MHz, $CDCl_3$)....170
Compound **7pa** HRMS (ESI-TOF)....171
Compound **7pb** ^{1}H NMR (600 MHz, $CDCl_3$)....172
Compound **7pb** ^{13}C NMR (125 MHz, $CDCl_3$)....173
Compound **7pb** HRMS (ESI-TOF)....174
Compound **7q** ^{1}H NMR (600 MHz, $CDCl_3$)....175
Compound **7q** ^{13}C NMR (150 MHz, $CDCl_3$)....176
Compound **7q** HRMS (ESI-TOF)....177
Compound **7r** ^{1}H NMR (600 MHz, $CDCl_3$)....178
Compound **7r** ^{13}C NMR (150 MHz, $CDCl_3$)....179
Compound **7r** HRMS (ESI-TOF)....180
Compound **7s** ^{1}H NMR (600 MHz, $CDCl_3$)....181
Compound **7s** ^{13}C NMR (150 MHz, $CDCl_3$)....182
Compound **7s** HRMS (ESI-TOF)....183
Compound **7t** ^{1}H NMR (600 MHz, $CDCl_3$)....184
Compound **7t** ^{13}C NMR (150 MHz, $CDCl_3$)....185
Compound **7t** HRMS (ESI-TOF)....186
Compound **7u** ^{1}H NMR (600 MHz, $CDCl_3$)....187
Compound **7u** ^{13}C NMR (150 MHz, $CDCl_3$)....188
Compound **7u** HRMS (ESI-TOF)....189
Compound **7v** ^{1}H NMR (600 MHz, $CDCl_3$)....190
Compound **7v** ^{13}C NMR (125 MHz, $CDCl_3$)....191
Compound **7v** ^{19}F NMR (471 MHz, $CDCl_3$)....192
Compound **7v** HRMS (ESI-TOF)....193
Compound **8b** ^{1}H NMR (500 MHz, CD_3OD)....194
Compound **8b** ^{13}C NMR (125 MHz, CD_3OD)....195

Compound **8b** HRMS (ESI-TOF):....196
Compound **8c** ¹H NMR (400 MHz, $CDCl_3$)....197
Compound **8c** ¹³C NMR (100 MHz, $CDCl_3$)....198
Compound **8c** HRMS (ESI-TOF):....199
Compound **8d** ¹H NMR (600 MHz, $CDCl_3$)....200
Compound **8d** ¹³C NMR (150 MHz, $CDCl_3$)....201
Compound **8d** HRMS (ESI-TOF):....202
Compound **8e** ¹H NMR (600 MHz, $CDCl_3$)....203
Compound **8e** ¹³C NMR (150 MHz, $CDCl_3$)....204
Compound **8e** HRMS (ESI-TOF):....205
Compound **8f** ¹H NMR (400 MHz, $CDCl_3$)....206
Compound **8f** ¹³C NMR (100 MHz, $CDCl_3$)....207
Compound **8f** HRMS (ESI-TOF):....208
Compound **8g** ¹H NMR (600 MHz, $CDCl_3$)....209
Compound **8g** ¹³C NMR (150 MHz, $CDCl_3$)....210
Compound **8g** HRMS (ESI-TOF):....211
Compound **8h** ¹H NMR (600 MHz, $CDCl_3$)....212
Compound **8h** ¹³C NMR (150 MHz, $CDCl_3$)....213
Compound **8h** HRMS (ESI-TOF):....214
Compound **8i** ¹H NMR (400 MHz, $CDCl_3$)....215
Compound **8i** ¹³C NMR (100 MHz, $CDCl_3$)....216
Compound **8i** HRMS (ESI-TOF):....217
Compound **8j** ¹H NMR (600 MHz, $CDCl_3$)....218
Compound **8j** ¹³C NMR (150 MHz, $CDCl_3$)....219
Compound **8j** HSQC spectrum (800/200 MHz, $CDCl_3$)....220
Compound **8j** HMBC spectrum (800/200 MHz, $CDCl_3$)....221
Compound **8j** ROESY spectrum (800 MHz, $CDCl_3$)....222
Compound **8j** HRMS (ESI-TOF):....223
Compound **8hs** ¹H NMR (600 MHz, $CDCl_3$)....224
Compound **8hs** ¹³C NMR (125 MHz, $CDCl_3$)....225
Compound **8hs** HRMS (ESI-TOF):....226
Compound **10b** ¹H NMR (600 MHz, $CDCl_3$)....227
Compound **10b** ¹³C NMR (125 MHz, $CDCl_3$)....228
Compound **10b** HRMS (ESI-TOF):....229
Compound **10c** ¹H NMR (600 MHz, $CDCl_3$)....230
Compound **10c** ¹³C NMR (150 MHz, $CDCl_3$)....231
Compound **10c** HRMS (ESI-TOF):....232
Compound **10d** ¹H NMR (600 MHz, $CDCl_3$)....233
Compound **10d** ¹³C NMR (150 MHz, $CDCl_3$)....234
Compound **10d** HRMS (ESI-TOF):....235
Compound **10ka** ¹H NMR (600 MHz, $CDCl_3$)....236
Compound **10ka** ¹³C NMR (150 MHz, $CDCl_3$)....237
Compound **10ka** HRMS (ESI-TOF):....238
Compound **10kb** ¹H NMR (600 MHz, $CDCl_3$)....239

Compound **10kb** ^{13}C NMR (150 MHz, $CDCl_3$) 240
Compound **10kb** HRMS (ESI-TOF): 241
Compound **10n** ^{1}H NMR (400 MHz, $CDCl_3$) 242
Compound **10n** ^{13}C NMR (150 MHz, $CDCl_3$) 243
Compound **10n** HRMS (ESI-TOF): 244
Compound **13** ^{1}H NMR (600 MHz, $CDCl_3$) 245
Compound **13** ^{13}C NMR (125 MHz, $CDCl_3$) 246
Compound **13** HRMS (ESI-TOF) 247
Compound **14** ^{1}H NMR (600 MHz, $CDCl_3$) 248
Compound **14** ^{13}C NMR (125 MHz, $CDCl_3$) 249
Compound **14** HRMS (ESI-TOF): 250
Compound **3** ^{1}H NMR (800 MHz, $CDCl_3$) 251
Compound **3** ^{13}C NMR (125 MHz, $CDCl_3$) 252
Compound **3** HRMS (ESI-TOF): 253
Compound **4** ^{1}H NMR (500 MHz, $CDCl_3$) 254
Compound **4** ^{13}C NMR (125 MHz, $CDCl_3$) 255
Compound **4** HRMS (ESI-TOF) 256
X-Ray Crystallography Data 257
Table S5 X-ray crystallography data for compound **4** (cephafortunoid B) 257
NMR Data Comparison of Synthetic and Natural Products 258
Table S6 ^{1}H-NMR data comparison for compound **3** (cephafortunoid A) 258
Table S7 ^{13}C-NMR data comparison for compound **3** (cephafortunoid A) 259
Table S8 ^{1}H-NMR data comparison for compound **4** (cephafortunoid B) 260
Table S9 ^{13}C-NMR data comparison for compound **4** (cephafortunoid B) 261
NMR Calculations for Compound 8j and Its Isomer 8j′ 262
Reaction Setup 264
References 265

General Experimental

Unless otherwise stated, all reactions were carried out under anhydrous conditions. Super-dried solvents and reagents were purchased at the highest commercial quality and used without further purification, unless otherwise stated. Reactions were monitored by thin layer chromatography (TLC) or LC/MS. TLC was performed using precoated silica gel 60 F254 (Merck), using short-wave UV light as the visualizing agent, and cerium molybdate (CAM or Hanessian's stain), phosphomolybdic acid (PMA), or $KMnO_4$ and heat as developing agents. Flash silica gel chromatography was performed using E. Merck silica gel (60, particle size 0.043–0.063 mm). NMR data were obtained on Bruker AVANCE III 400, AVANCE III 500, Ascend 600 and/or Ascend 800 NMR spectrometers referenced to deuterated solvent peaks (δ_H 7.26 in $CDCl_3$, 1.94 in CD_3CN or 3.31 in CD_3OD; δ_C 77.16 in $CDCl_3$, 118.3 in acetonitrile-d_3 or 49.00 in CD_3OD).

Photochemical reaction was carried out under visible light irradiation by LEDs at 25 ºC. Photosyn-10 manufactured by Shanghai Quanhuan Technology Co., Ltd was used in this system.

The X-ray diffraction analysis was performed on a Bruker SMART CCD detector employing graphite monochromated Cu-Kα radiation. Melting points were recorded on an SGM X-4 apparatus. ESIMS and HRESIMS were implemented on a Bruker Daltonics Esquire 3000 plus and Waters-Micromass Q-TOF Ultima Global mass spectrometer, respectively.

Optical rotations (Na lamp, 589 nm) were obtained with an Autopol VI Automatic Polarimeter at room temperature; concentrations were reported in g/100mL.

Fluorescence quenching studies were performed using a Techcomp FL970 Fluorescence Spectrometer.

Optimization of the Reaction Conditions

Table S1 Optimization for light sources

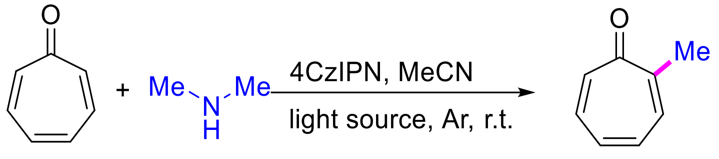

Entry	Additives (1 mol%)	Amine (2 equiv.)	Wavelength (nm) for light source[a]	Solvent (0.13 mol/L)	Irradiation time (h)	Yield
1	4CzIPN	Me_2NH	365	MeCN	10	decomposed
2	4CzIPN	Me_2NH	395	MeCN	10	43%
3	4CzIPN	Me_2NH	420	MeCN	10	63%
4	4CzIPN	Me_2NH	455	MeCN	10	79%
5	4CzIPN	Me_2NH	500	MeCN	10	N.R.
6	4CzIPN	Me_2NH	white light[b]	MeCN	10	26%

All the reactions were carried out at the same conditions with the variation of the wavelength: **5a** (0.283 mmol), **6a** (0.566 mmol), 4CzIPN (1 mol%), MeCN (2.2 mL), 10 h. [a]The power of all the light sources was 7W except otherwise noted; [b]Philips 23W essential white bulb was used.

Table S2 Optimization for photocatalyst

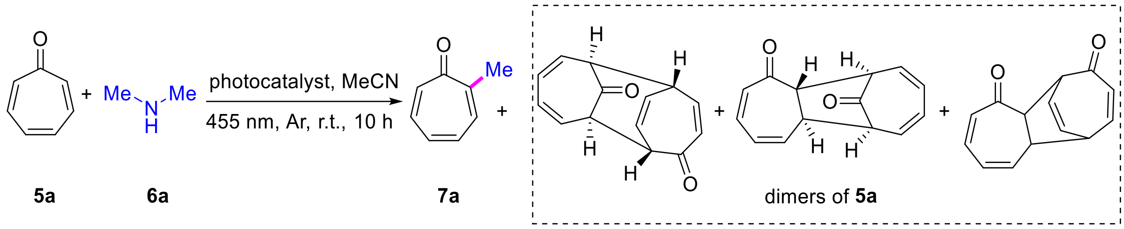

Entry	Photocatalyst	Amount of photocatalyst (mol%)	Yield
1	4CzIPN	1	79%
2	4CzPN	1	54%
3	4DPAIPN	1	N.D.
4	Eosin Y	1	N.D.
5	$[Ru(bpz)_3][PF_6]_2$	1	11%
6	thioxanthone	1	N.D. only dimers
7	9H-xanthen-9-one	1	N.D. only dimers
8	$[Ru(phen)_3]Cl_2$	1	N.D. only dimers
9	$(Ir[dF(CF_3)ppy]_2(dtbpy))PF_6$	1	15%
10	4CzIPN	2	75%

Note: All the reactions were carried out at the same conditions with the variation of the photocatalyst: **5a** (0.283 mmol), **6a** (0.566 mmol), photocatalyst, MeCN (2.2 mL), 455 nm (7 W, LEDs), 10 h.

The structures of the photocatalysts are listed below.

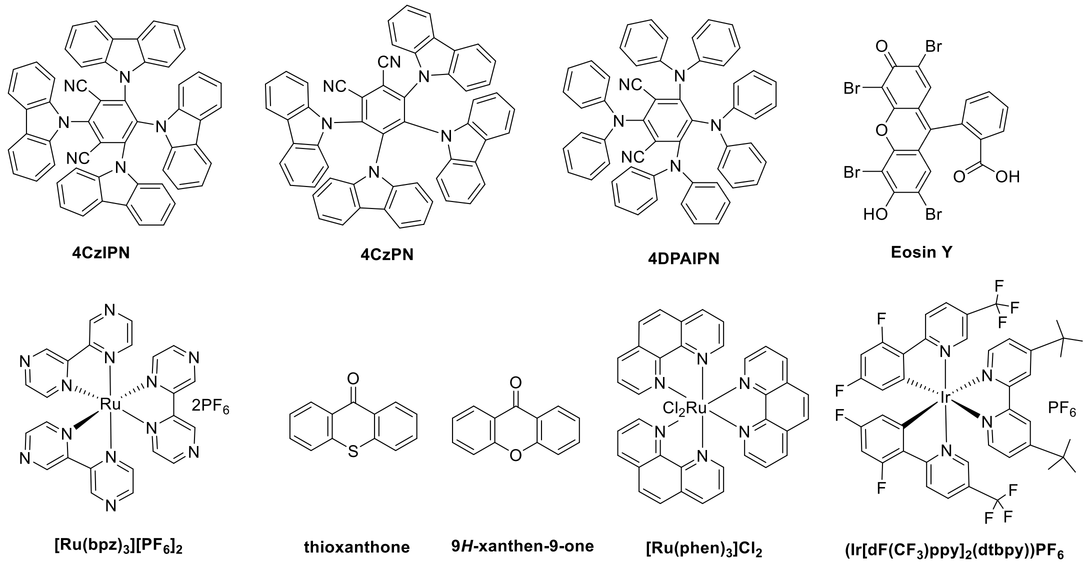

Table S3 Optimization for solvent

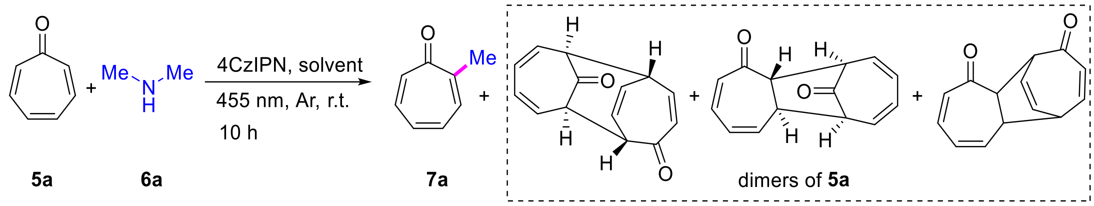

Entry	Solvent (0.13 mol/L)	Yield
1	MeCN	79%
2	PhCN	69%
3	Acetone	13%
4	MeCN/H_2O=7:1	70%
5	MTBE	N.D. only dimers
6	THF	74%
7	MeOH	Trace
8	DMSO	Decomposed
9	Toluene	Dimers
10	Pyridine	Decomposed

Note: All the reactions were carried out at the same conditions with the variation of solvent: **5a** (0.283 mmol), **6a** (0.566 mmol), 4CzIPN (1 mol%), solvent (2.2 mL), 455 nm (7 W, LEDs), 10 h.

Scheme S1 Optimization for amine

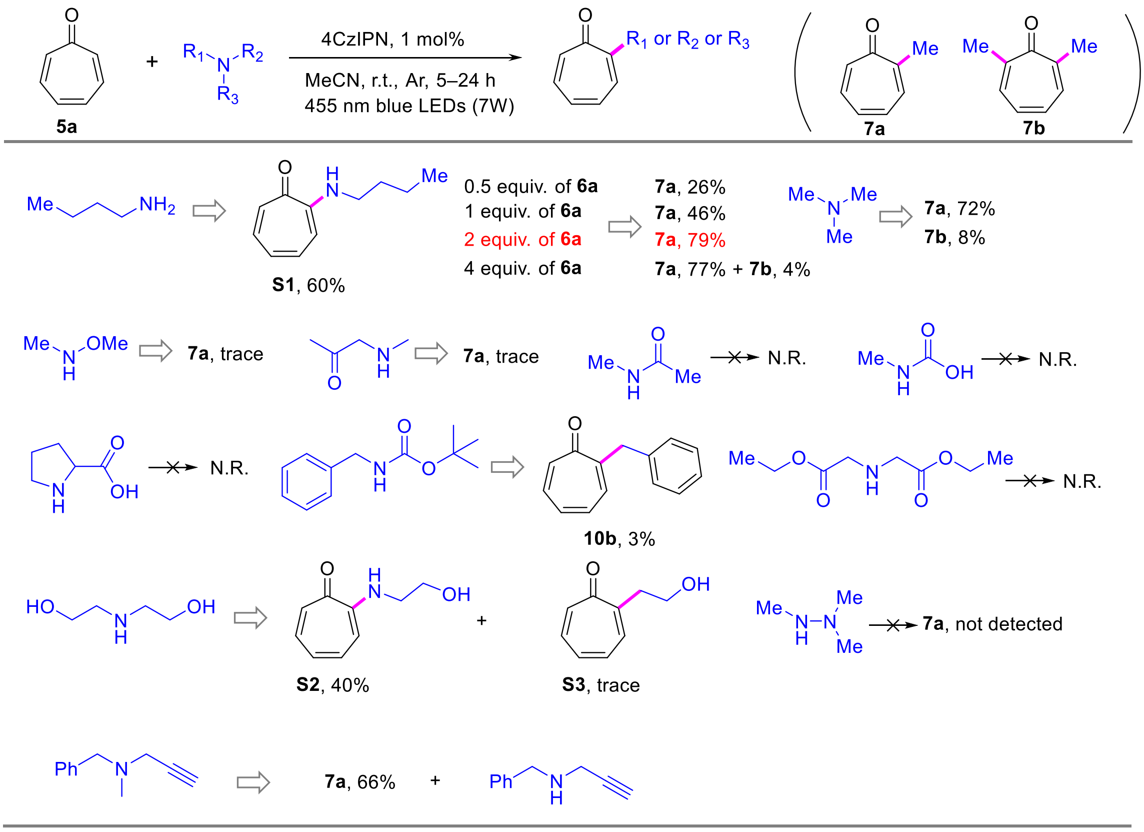

Note: All the reactions were carried out at the standard conditions with the variation of amines. **5a** (0.283

mmol), amine (2 equiv. unless otherwise noted), 4CzIPN (1 mol%), MeCN (2.2 mL), 455 nm (7 W, LEDs), 10 h. Compounds **S1** and **S2** were known compounds, and the NMR data were identical to those reported[1,2].

The optimization results showed that unactivated secondary amines were the best choice for tropone alkylation.

Table S4 Sensitivity assessment of the C(sp^2)–H alkylation of tropones

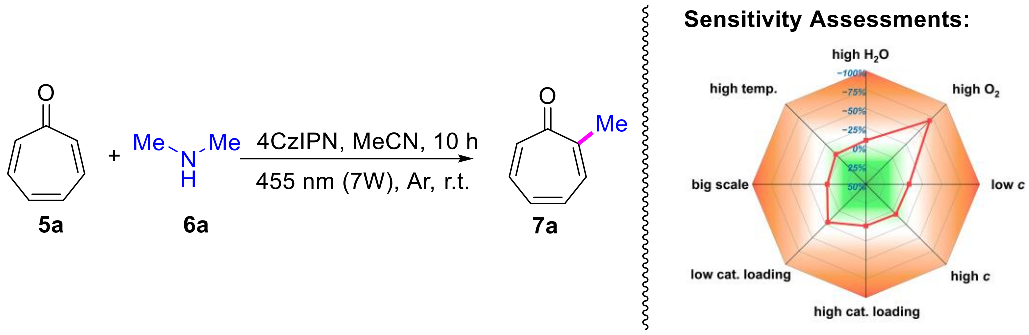

Entry	Modification	Deviation from standard conditions	Yield	Deviation from benchmark
1	none	none	79%	0%
2	high H_2O	H_2O (0.27 mL)	70%	−9%
3	high O_2	performed in O_2	10%	−69%
4	low concentration	0.065 M	72%	−7%
5	high concentration	0.30 M	73%	−6%
6	high cat. loading	4 mol% cat.	73%	−5%
7	low cat. loading	0.1 mol% cat.	58%	−21%
8	big scale	2.83 mmol **5a**	78%	−1%
9	high temp.	40 °C	72%	−6%

Incompatible Substrates

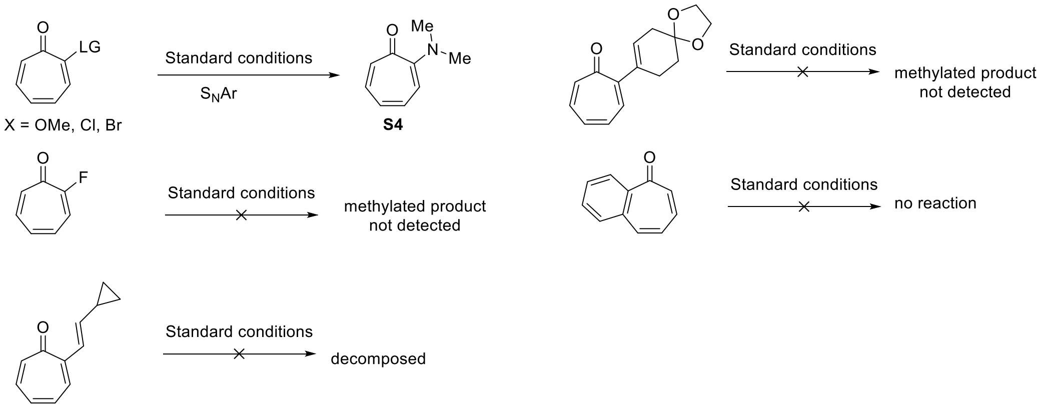

Scheme S2 Incompatible substrates of tropones.

Note: For tropones bearing leaving groups (LG), reaction patterns were similar to S_NAr which have already been reported.[3] Compound **S4** was known compound, and the NMR data were identical to those reported.[4,5]

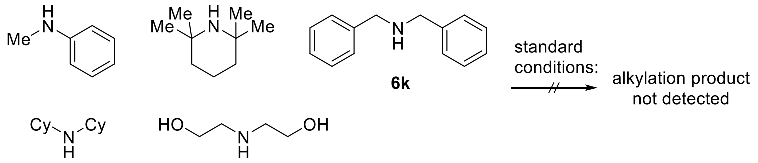

Scheme S3 Incompatible substrates of secondary amines.

Preparation of Starting Materials

Compounds **5a**, **5m**, **6b**–**6l**, **9a**–**9j**, **9l** were purchased at the highest commercial quality and used without further purification. Compounds **2** and **12** were isolated from *Cephalotaxus fortunei* as we previously reported.[6]

General procedure A, B and C

General procedure A: preparation of 5c, 5e–5f, and 5j–5k, Suzuki-Miyaura reaction

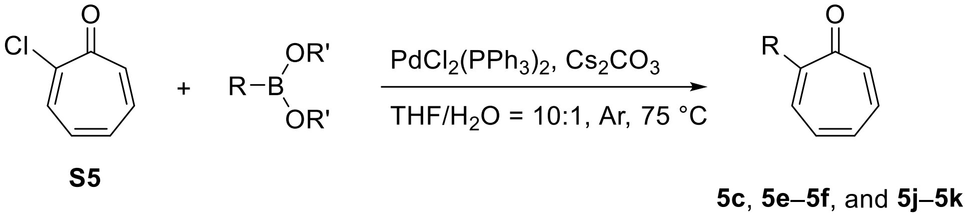

2-Chloro-2,4,6-cycloheptatrien-1-one (1.0 equiv.), boronic acid/ester (2.0 equiv.), and cesium carbonate (4.0 equiv.) were added to 10:1 THF/H_2O (0.2 M). The mixture was thoroughly degassed by bubbling argon through solution (10 min). Bis(triphenylphosphine)palladium(II) dichloride (0.1 equiv.) was added, and the mixture was again degassed with argon (5 min). The homogenous solution was heated at 75 °C for 16 h before being cooled to room temperature. Water was added, and the mixture was extracted with EtOAc. The combined organic layer was washed with brine, dried over Na_2SO_4, filtered and concentrated in vacuo. The residue was purified by silica gel column chromatography to provide the desired product.

General procedure B: preparation of 5g–5i, and 5l, Suzuki-Miyaura reaction

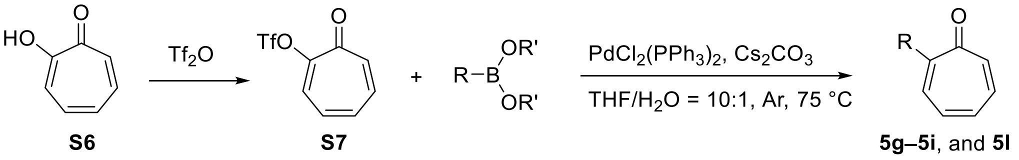

For R bearing electron withdraw groups (EWG): Suzuki-Miyaura coupling (the same reaction condition as general procedure A) went on smoothly when triflate was preinstalled to the *α* position, whereas **S7** was prepared according to the literature report.[4]

General procedure C: preparation of 5q–5v, EDCI coupling

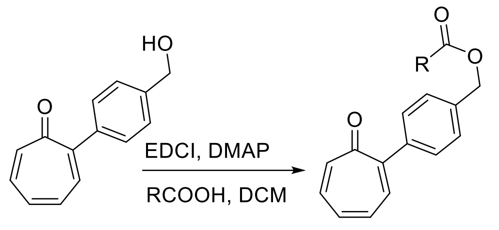

S8 5q to 5v

To a solution of the alcohol **S8** (1 equiv.) in DCM (0.02 M) was added the carboxylic acid (2.8 equiv.), DMAP (3 equiv.), and EDCl·HCl (3 equiv.). The solution was stirred at room temperature until the reaction was complete, as indicated by TLC analysis. Subsequently, saturated ammonium chloride solution was added, and the aqueous layer was extracted with DCM three times. The combined organic layers were dried with sodium sulfate, and evaporated in vacuo. The residue was purified by silica gel column chromatography (PE/EtOAc = 3:1) to give the products **5q** to **5v**.

Synthesis of 5c

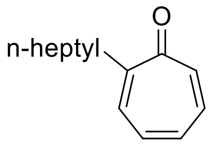

5c

Following the general procedure A, compound **5c** (149.8 mg, 74% yield) was obtained using heptylboronic acid as starting materials. The elution condition for flash chromatography: PE/EtOAc = 4:1.

Physical State: yellow oil.

TLC: R_f = 0.75 (PE/EtOAc = 1:1).

^{1}H NMR (400 MHz, CDCl$_3$): δ 7.25 – 7.21 (m, 1H), 7.12 – 7.01 (m, 2H), 6.99 – 6.84 (m, 2H), 2.68 – 2.59 (m, 2H), 1.61 – 1.49 (m, 2H), 1.38 – 1.24 (m, 6H), 0.90 – 0.84 (m, 3H).

^{13}C NMR (100 MHz, CDCl$_3$): δ 187.3, 156.5, 140.5, 135.4, 134.8, 134.0, 132.5, 35.7, 31.9, 29.8, 29.3, 29.0, 22.8, 14.2.

HRESIMS: m/z 227.1404 $[M + Na]^+$ (calcd for $C_{14}H_{20}NaO^+$, 227.1406); m/z 222.1850 $[M + NH_4]^+$ (calcd for $C_{14}H_{24}NO^+$, 222.1852).

Synthesis of 5d

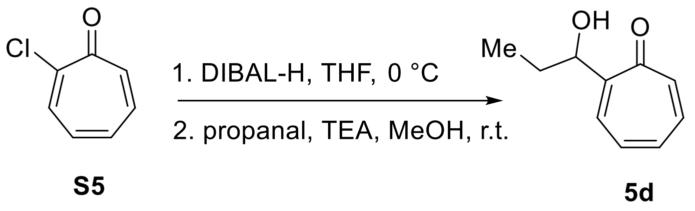

DIBAL-H (2.4 mmol, 2.4 mL of 1.0 mol/L hexane solution) was added to a stirred solution of **S5** (281 mg, 2 mmol) in THF (3 mL) at 0 °C, and the mixture was warmed up to room temperature, and stirred for another 30 min under a nitrogen atmosphere. After addition of propanal (4.0 mmol) to the reaction mixture, the mixture was stirred for 1 h; then triethylamine (342.0 mg, 2.4 mmol) and methanol (20 mL) were added to the mixture. The reaction mixture was then filtered through Celite and the filtrate was concentrated. The residue was purified by silica gel column chromatography (PE:EtOAc = 1:1) to give the product **5d** (190 mg, 1.16 mmol, 68% yield) as yellowish solid. Spectral data matched reported values therein.[7]

Synthesis of 5e

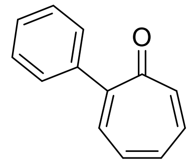

5e

Following the general procedure A, compound **5e** (86% yield, 121.2 mg) was obtained using phenylboronic acid as starting materials. Spectral data matched reported values therein.[8] The elution condition for flash chromatography: PE/EtOAc = 4:1 to 2:1.

TLC: R_f = 0.66 (PE/EtOAc = 2:1).

Physical State: white solid.

Synthesis of 5f

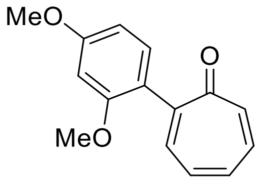

5f

Following the general procedure A, compound **5f** (77% yield, 101.3 mg) was obtained using (2,4-dimethoxyphenyl)boronic acid as starting materials. The elution condition for flash chromatography: PE/EtOAc = 3:1 to 1:1.

Physical State: yellow solid.

TLC: R_f = 0.75 (PE/EtOAc = 3:2).

^{1}H NMR (600 MHz, $CDCl_3$): δ 7.27 (dd, *J* = 8.7, 1.0 Hz, 1H), 7.15 (d, *J* = 8.3 Hz, 1H), 7.12 – 7.04 (m, 2H), 6.99 – 6.92 (m, 1H), 6.92 – 6.86 (m, 1H), 6.55 – 6.48 (m, 2H), 3.81 (s, 3H), 3.74 (s, 3H).

^{13}C NMR (150 MHz, $CDCl_3$): δ 186.9, 161.2, 157.3, 150.9, 140.7, 136.3, 134.8, 133.5, 132.9, 131.1, 122.9, 104.8, 99.0, 55.8, 55.4.

HRESIMS: m/z 243.1016 $[M + H]^+$ (calcd for $C_{15}H_5O_3^+$, 243.1016).

Synthesis of 5g

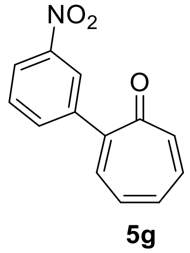

5g

Following the general procedure B, compound **5g** (57% yield, 89.7 mg) was obtained using (3-nitrophenyl)boronic acid as starting materials. The elution condition for flash chromatography: PE/EtOAc = 3:1 to 1:1.

Physical State: yellowish solid.

TLC: R_f = 0.82 (PE/EtOAc = 1:2).

^{1}H NMR (400 MHz, $CDCl_3$): δ 8.32 (t, J = 2.0 Hz, 1H), 8.21 (ddd, J = 8.2, 2.3, 1.1 Hz, 1H), 7.83 (dt, J = 7.7, 1.3 Hz, 1H), 7.56 (t, J = 8.0 Hz, 1H), 7.42 – 7.35 (m, 1H), 7.24 –

7.13 (m, 2H), 7.13 – 6.99 (m, 2H).

^{13}C NMR (100 MHz, CDCl$_3$): δ 185.8, 150.0, 148.2, 143.0, 141.5, 137.3, 135.9, 135.5, 134.7, 133.6, 129.1, 124.3, 123.3.

HRESIMS: m/z 228.0655 [M + H]$^+$ (calcd for $C_{13}H_{10}NO_3^+$, 228.0655).

Synthesis of 5h

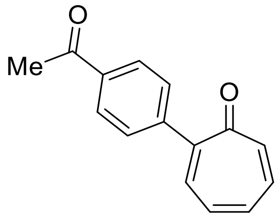

5h

Following the general procedure B, compound **5h** (57% yield, 66.3 mg) was obtained using (4-acetylphenyl)boronic acid as starting materials. The elution condition for flash chromatography: PE/EtOAc = 3:1 to 1:1.

Physical State: white solid.

TLC: R_f = 0.71 (PE/EtOAc = 1:2).

^{1}H NMR (400 MHz, CDCl$_3$): δ 7.95 – 7.87 (m, 2H), 7.54 – 7.46 (m, 2H), 7.29 (dt, *J* = 8.3, 1.3 Hz, 1H), 7.10 (dt, *J* = 4.2, 1.3 Hz, 2H), 7.03 – 6.91 (m, 2H), 2.54 (d, *J* = 1.3 Hz, 3H).

^{13}C NMR (100 MHz, CDCl$_3$): δ 197.6, 186.0, 151.2, 144.7, 142.4, 136.8, 136.5, 135.5, 134.0, 133.6, 129.3, 128.0, 26.6.

HRESIMS: m/z 225.0911 [M + H]$^+$ (calcd for $C_{15}H_{13}O_2^+$, 225.0910).

Synthesis of 5i

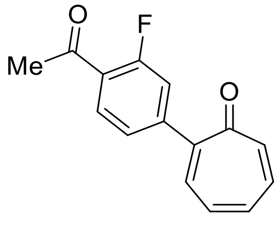

5i

Following the general procedure B, compound **5i** (51% yield, 67.9 mg) was obtained

using (4-acetyl-3-fluorophenyl)boronic acid as starting materials. The elution condition for flash chromatography: PE/EtOAc = 3:1 to 1:1.

Physical State: white solid.

TLC: R_f = 0.54 (PE/EtOAc = 1:1).

^{1}H NMR (600 MHz, $CDCl_3$): δ 7.85 (t, J = 7.9 Hz, 1H), 7.35 – 7.22 (m, 3H), 7.14 (dd, J = 4.2, 1.4 Hz, 2H), 7.06 – 6.97 (m, 2H), 2.61 (d, J = 4.7 Hz, 3H).

^{13}C NMR (150 MHz, $CDCl_3$): δ 195.5, 185.7, 162.6, 160.9, 149.9, 146.6, 146.6, 142.8, 137.0, 135.6, 134.6, 133.5, 130.3, 130.3, 125.2, 125.1, 125.1, 125.1, 117.8, 117.6, 31.4, 31.4.

HRESIMS: m/z 243.0816 $[M + H]^+$ (calcd for $C_{15}H_{12}FO_2^+$, 243.0816).

Synthesis of 5j

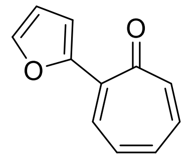

5j

Following the general procedure A, compound **5j** (76% yield, 76.2 mg) was obtained using furan-2-ylboronic acid as starting materials. The elution condition for flash chromatography: PE/EtOAc = 3:1 to 1:1.

Physical State: yellowish solid.

TLC: R_f = 0.4 (PE/EtOAc = 1:1).

^{1}H NMR (600 MHz, $CDCl_3$): δ 8.02 (d, *J* = 9.5 Hz, 1H), 7.75 (d, *J* = 3.5 Hz, 1H), 7.51 (d, *J* = 1.7 Hz, 1H), 7.19 – 7.07 (m, 3H), 6.97 – 6.90 (m, 1H), 6.52 (dd, *J* = 3.7, 1.7 Hz, 1H).

^{13}C NMR (150 MHz, $CDCl_3$): δ 183.9, 150.5, 143.9, 141.3, 139.3, 134.8, 133.8, 132.9, 131.0, 116.7, 112.9.

HRESIMS: m/z 173.0595 $[M + H]^+$ (calcd for $C_{11}H_9O_2^+$, 173.0597).

Synthesis of 5k

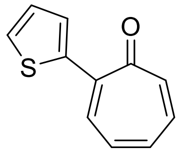

5k

Following the general procedure A, compound **5k** (49% yield, 66.6 mg) was obtained using thiophen-2-ylboronic acid as starting materials. Spectral data matched reported values therein.[7] The elution condition for flash chromatography: PE/EtOAc = 3:1 to 1:1.

Physical State: yellowish solid.

TLC: R_f = 0.38 (PE/EtOAc = 1:1).

Synthesis of 5l

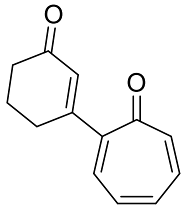

5l

Following the general procedure B, compound **5l** (56% yield, 64.3 mg) was obtained using 3-oxo-1-cyclohexen-1-boronic acid pinacol ester as starting materials. The elution condition for flash chromatography: PE/EtOAc = 2:1.

Physical State: yellow oil.

TLC: R_f = 0.68 (PE/EtOAc = 1:1).

^{1}H NMR (400 MHz, $CDCl_3$): δ 7.24 – 6.98 (m, 5H), 6.07 (s, 1H), 2.58 (t, *J* = 6.0 Hz, 2H), 2.46 (t, *J* = 6.7 Hz, 2H), 2.09 (p, *J* = 6.3 Hz, 2H).

^{13}C NMR (100 MHz, $CDCl_3$): δ 199.8, 185.7, 164.3, 153.3, 142.1, 135.8, 134.8, 134.4, 133.8, 128.3, 37.7, 28.4, 23.2.

HRESIMS: m/z 199.0765 $[M - H]^-$ (calcd for $C_{13}H_{11}O_2^-$, 199.0765).

Synthesis of 5n

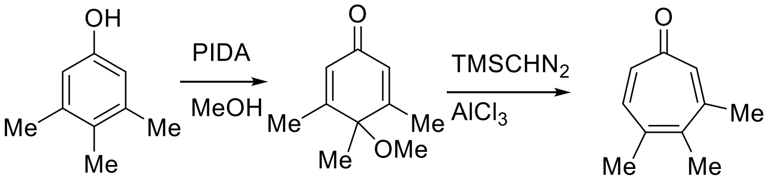

S9 **S10** **5n**

To a solution of **S9** (600mg, 4.4 mmol, 1.0 equiv.) in MeCN/MeOH (1:1 v/v, 44 mL) was added phenyliodine(III) diacetate (PIDA; 1.70 g, 5.3 mmol, 1.2 equiv.) at 0 °C. After stirring at room temperature for 5 h, the reaction mixture was quenched with sat. aq. $NaHCO_3$ (20 mL), diluted with H_2O (30 mL) and extracted with DCM (30 × 5 mL). The combined organic phase was dried over Na_2SO_4 and concentrated in vacuo. The resulting residue was purified by silica gel flash column chromatography (hexanes/EtOAc = 2:1), yielding **S10** (0.39 g, 2.6 mmol, 60%) as a colorless solid.

A flame-dried vial with a magnetic stir bar was charged with $AlCl_3$ (1.05 g, 7.8 mmol, 3.0 equiv.). The vial was evacuated and backfilled with N_2 three times and cooled to – 60 °C. Freshly distilled DCM (4.3 mL) was added, and the suspension was stirred at – 60 °C for 5 min. A solution of **S10** (0.39 g, 2.6 mmol, 1.0 equiv.) in freshly distilled DCM (21 mL) was added and stirred at – 60 °C for 10 min to give a grayish suspension. TMS-diazomethane (0.2 M, prepared from a 2.0 M solution in hexanes diluted with freshly distilled DCM, 64 mL, 13 mmol, 5.0 equiv.) was added over 2 min resulting in a yellowish solution. The mixture was stirred at –60 °C for 3 h and quenched with sat. aq. $NaHCO_3$ (50 mL). The suspension was diluted with H_2O (20 mL) and extracted with DCM (80 × 3 mL). The combined organic layers were dried over anhydrous Na_2SO_4, and the solvent was removed under reduced pressure. The residue was purified by flash chromatography (hexanes/EtOAc = 1:1), yielding **5n** (200.2 mg, 1.35 mmol, 52%) as a colorless oil.

Physical State: colorless oil.

TLC: R_f = 0.25 (PE/EtOAc = 1:2).

^{1}H NMR (600 MHz, CDCl$_3$): δ 6.93 (d, J = 12.3 Hz, 1H), 6.86 (d, J = 3.1 Hz, 1H), 6.66 (dd, J = 12.3, 3.1 Hz, 1H), 2.18 (s, 6H), 2.06 (s, 3H).

^{13}C NMR (150 MHz, CDCl$_3$): δ 186.2, 148.5, 143.3, 141.2, 140.5, 139.6, 137.4, 27.7, 25.9, 20.8.

HRESIMS: m/z 149.0960 [M + H]$^+$ (calcd for $C_{10}H_{13}O^+$, 149.0961).

Synthesis of 5o

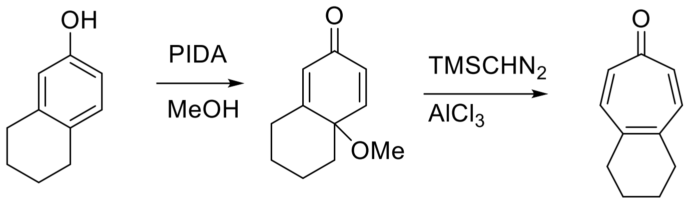

S11 **S12** **5o**

Following the synthesis procedure of **S9** to **5n**， compound **5o** (164.8 mg, 1.03 mmol, 44% yield) was obtained after flash chromatography (PE/EtOAc = 1:1).

Physical State: colorless oil.

TLC: R_f = 0.38 (PE/EtOAc = 1:2).

^{1}H NMR (600 MHz, CDCl$_3$): δ 6.91 (d, *J* = 12.3 Hz, 2H), 6.84 (d, *J* = 12.0 Hz, 2H), 2.65 (h, *J* = 3.0 Hz, 4H), 1.75 (p, *J* = 3.3 Hz, 4H).

^{13}C NMR (150 MHz, CDCl$_3$): δ 187.4, 142.8, 142.0, 138.0, 33.6, 22.1.

HRESIMS: m/z 161.0963 [M + H]$^+$ (calcd for $C_{11}H_{13}O^+$, 161.0961).

Synthesis of 5p

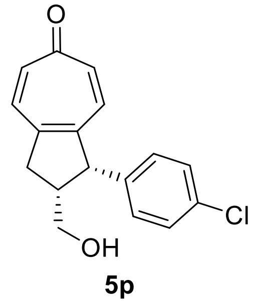

5p

Compound **5p** (57% yield, 60.1 mg) was prepared according to the literature report, and spectral data matched reported values therein.[9] The elution condition for flash chromatography: PE/EtOAc = 1:1.

TLC: R_f = 0.46 (PE/EtOAc = 1:2).

Physical State: yellowish oil.

Specific Rotation: $[\alpha]^{18}_{D}$ = −21 (c 0.11, MeOH); lit. report: $[\alpha]^{20}_{D}$ = −46 (c 0.10, EtOH).[9]

Synthesis of S8

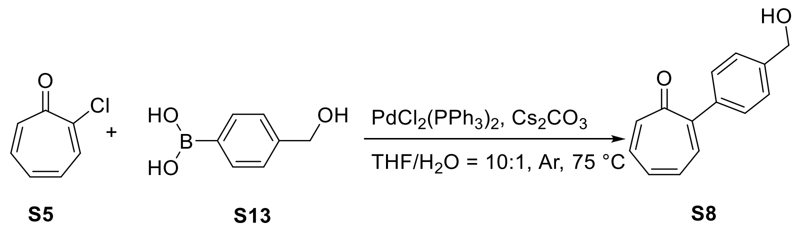

Following the general procedure A, compound **S8** (87% yield, 2.23 g) was obtained using 4-(hydroxymethyl)phenyl boronic acid (**S13**) as starting materials. The elution condition for flash chromatography: PE/EtOAc = 2:1 to 1:1.

Physical State: yellow solid.

TLC: R_f = 0.32 (PE/EtOAc = 1:1).

^{1}H NMR (600 MHz, CDCl$_3$): δ 7.43 (d, J = 7.9 Hz, 2H), 7.34 (d, J = 8.3 Hz, 3H), 7.17 – 7.06 (m, 2H), 7.02 (t, J = 9.8 Hz, 1H), 6.98 – 6.92 (m, 1H), 4.63 (s, 2H), 2.95 (s, 1H).

^{13}C NMR (150 MHz, CDCl$_3$): δ 186.7, 152.4, 142.2, 141.6, 139.0, 136.7, 135.6, 133.9, 133.4, 129.3, 126.6, 64.7.

HRESIMS: m/z 213.0910 $[M + H]^+$ (calcd for $C_{14}H_{13}O_2^+$, 213.0910).

Synthesis of 5q

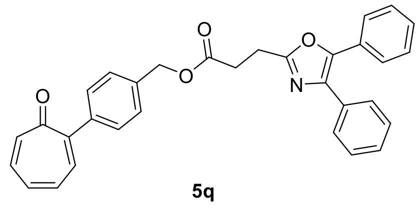

5q

Following the general procedure C, compound **5q** (99% yield, 251.1 mg) was obtained by using oxaprozin as starting material. The elution condition for flash chromatography: PE/EtOAc = 2:1 to 1:1.

Physical State: yellow solid.

TLC: R_f = 0.52 (PE/EtOAc = 1:1).

^{1}H NMR (400 MHz, CDCl$_3$): δ 7.68 – 7.59 (m, 2H), 7.59 – 7.49 (m, 2H), 7.49 – 7.22

(m, 11H), 7.20 – 7.05 (m, 2H), 7.04 – 6.89 (m, 2H), 5.19 (s, 2H), 3.21 (t, J = 7.4 Hz, 2H), 2.98 (t, J = 7.4 Hz, 2H).

^{13}C NMR (150 MHz, CDCl$_3$): δ 186.5, 171.9, 161.8, 152.2, 145.6, 142.5, 140.0, 136.6, 136.1, 135.3, 135.3, 133.8, 133.5, 132.6, 129.5, 129.1, 128.8, 128.7, 128.6, 128.2, 128.1, 128.0, 126.6, 66.4, 31.3, 23.7.

HRESIMS: m/z 488.1855 [M + H]$^+$ (calcd for $C_{32}H_{26}NO_4^+$, 488.1856).

Synthesis of 5r

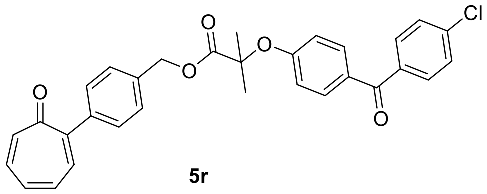

5r

Following the general procedure C, compound **5r** (98% yield, 241.3 mg) was obtained by using fenofibric acid as starting material. The elution condition for flash chromatography: PE/EtOAc = 2:1 to 1:1.

Physical State: yellowish solid.

TLC: R_f = 0.55 (PE/EtOAc = 1:1).

^{1}H NMR (600 MHz, CDCl$_3$): δ 7.67 – 7.61 (m, 4H), 7.42 – 7.35 (m, 4H), 7.30 – 7.23 (m, 3H), 7.11 – 7.05 (m, 2H), 7.01 – 6.96 (m, 1H), 6.96 – 6.90 (m, 1H), 6.79 – 6.74 (m, 2H), 5.20 (s, 2H), 1.67 (s, 6H).

^{13}C NMR (150 MHz, CDCl$_3$): δ 194.1, 186.2, 173.4, 159.5, 151.7, 142.4, 140.2, 138.3, 136.5, 136.3, 135.3, 135.2, 133.7, 133.5, 132.0, 131.2, 130.3, 129.3, 128.5, 128.2, 117.3, 79.4, 67.0, 25.5.

HRESIMS: m/z 513.1462 [M + H]$^+$ (calcd for $C_{31}H_{26}ClO_5^+$, 513.1463).

Synthesis of 5s

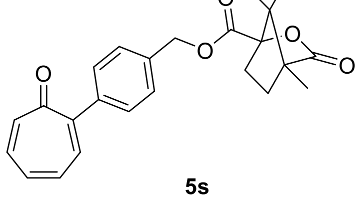

5s

Following the general procedure C, compound **5s** (95% yield, 235.1 mg) was obtained by using (−)-camphanic acid as starting material. The elution condition for flash chromatography: PE/EtOAc = 2:1 to 1:1.

Physical State: white solid.

TLC: R_f = 0.57 (PE/EtOAc = 1:1).

^{1}H NMR (600 MHz, $CDCl_3$): δ 7.49 – 7.45 (m, 2H), 7.41 – 7.36 (m, 2H), 7.33 (dd, *J* = 8.7, 1.0 Hz, 1H), 7.17 – 7.08 (m, 2H), 7.02 (ddt, *J* = 10.9, 8.8, 1.1 Hz, 1H), 6.99 – 6.93 (m, 1H), 5.26 (s, 2H), 2.42 (ddd, *J* = 13.5, 10.8, 4.2 Hz, 1H), 2.06 – 1.99 (m, 1H), 1.90 (ddd, *J* = 13.2, 10.8, 4.6 Hz, 1H), 1.66 (ddd, *J* = 13.4, 9.4, 4.2 Hz, 1H), 1.08 (s, 3H), 1.02 (s, 3H), 0.91 (s, 3H).

^{13}C NMR (150 MHz, $CDCl_3$): δ 186.4, 178.1, 167.4, 151.9, 142.4, 140.3, 136.6, 135.4, 135.3, 133.7, 133.6, 129.5, 128.0, 91.1, 66.9, 54.8, 54.3, 30.7, 29.0, 16.9, 16.8, 9.7.

HRESIMS: m/z 393.1699 $[M + H]^+$ (calcd for $C_{24}H_{25}O_5^+$, 393.1697).

Specific Rotation: $[\alpha]^{18}_D$ = −3.5 (*c* 0.11, MeOH).

Synthesis of 5t

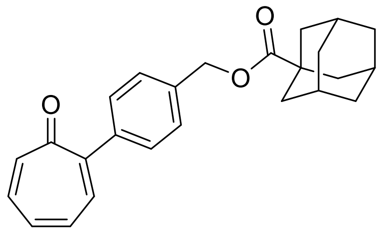

5t

Following the general procedure C, compound **5t** (95% yield, 244.2 mg) was obtained by using adamantane carboxylic acid as starting material. The elution condition for flash chromatography: PE/EtOAc = 3:1 to 1:1.

Physical State: white foam.

TLC: R_f = 0.56 (PE/EtOAc = 1:1).

^{1}H NMR (600 MHz, $CDCl_3$): δ 7.47 (d, *J* = 7.8 Hz, 2H), 7.34 (dd, *J* = 10.6, 8.4 Hz, 3H), 7.15 (d, *J* = 12.1 Hz, 1H), 7.10 (dd, *J* = 12.2, 7.5 Hz, 1H), 7.01 (t, *J* = 9.8 Hz, 1H), 6.94 (dd, *J* = 10.9, 7.6 Hz, 1H), 5.10 (s, 2H), 2.01 (dd, *J* = 7.6, 4.4 Hz, 3H), 1.92 (d, *J*

= 3.0 Hz, 6H), 1.75 – 1.66 (m, 6H).

^{13}C NMR (150 MHz, CDCl$_3$): δ 186.4, 177.4, 152.1, 142.3, 139.6, 136.8, 136.4, 135.2, 133.7, 133.4, 129.3, 127.4, 65.4, 40.8, 38.9, 36.5, 28.0.

HRESIMS: m/z 375.1953 [M + H]$^+$ (calcd for $C_{25}H_{27}O_3^+$, 375.1955).

Synthesis of 5u

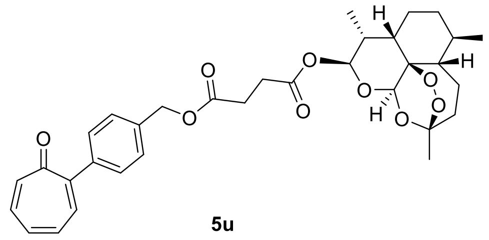

5u

Following the general procedure C, compound **5o** (198.7 mg) was obtained by using artesunate as starting material. The elution condition for flash chromatography: PE/EtOAc = 2:1 to 2:3.

Physical State: white powder.

TLC: R_f = 0.57 (PE/EtOAc = 1:1).

^{1}H NMR (600 MHz, CDCl$_3$): δ 7.51 – 7.46 (m, 2H), 7.40 – 7.36 (m, 2H), 7.35 (dd, *J* = 8.8, 1.0 Hz, 1H), 7.18 (dt, *J* = 12.1, 1.1 Hz, 1H), 7.15 (s, 1H), 7.03 (ddt, *J* = 10.9, 8.8, 1.1 Hz, 1H), 6.97 (ddt, *J* = 10.9, 7.5, 1.2 Hz, 1H), 5.79 (d, *J* = 9.8 Hz, 1H), 5.43 (s, 1H), 5.15 (s, 2H), 2.80 – 2.72 (m, 3H), 2.72 – 2.63 (m, 1H), 2.60 – 2.51 (m, 1H), 2.37 (ddd, *J* = 14.6, 13.4, 4.0 Hz, 1H), 2.04 – 2.00 (m, 1H), 1.89 (ddt, *J* = 13.6, 6.8, 3.6 Hz, 1H), 1.77 (dq, *J* = 13.5, 3.7 Hz, 1H), 1.71 (dq, *J* = 13.5, 3.4 Hz, 1H), 1.63 (t, *J* = 4.7 Hz, 1H), 1.54 – 1.45 (m, 1H), 1.42 (s, 3H), 1.40 – 1.28 (m, 3H), 1.05 – 0.99 (m, 1H), 0.96 (d, *J* = 6.2 Hz, 3H), 0.84 (d, *J* = 7.1 Hz, 3H).

^{13}C NMR (150 MHz, CDCl$_3$): δ 186.5, 172.0, 171.2, 152.2, 142.5, 140.0, 136.6, 135.3, 133.8, 133.5, 129.5, 128.1, 104.6, 92.3, 91.6, 80.2, 66.3, 51.7, 45.4, 37.4, 36.4, 34.2, 31.9, 29.3, 29.1, 26.1, 24.7, 22.1, 20.3, 12.1.

HRESIMS: m/z 601.2407 [M + Na]$^+$ (calcd for $C_{33}H_{38}NaO_9^+$, 601.2408); m/z 596.2851 [M + NH$_4$]$^+$ (calcd for $C_{33}H_{42}NO_9^+$, 596.2854).

Specific Rotation: $[\alpha]^{18}_D$ = +2.5 (*c* 0.04, MeOH).

Synthesis of 5v

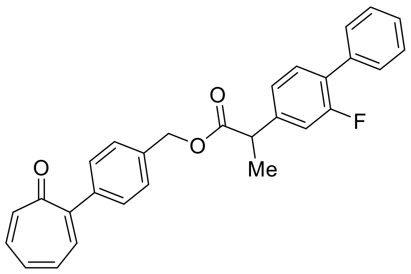

5v

Following the general procedure C, compound **5v** (201.3 mg) was obtained by using flurbiprofen as starting material. The elution condition for flash chromatography: PE/EtOAc = 2:1 to 1:1.

Physical State: yellow powder.

TLC: R_f = 0.31 (PE/EtOAc = 2:1).

^{1}H NMR (600 MHz, CDCl$_3$): δ 7.58 – 7.53 (m, 2H), 7.48 – 7.29 (m, 9H), 7.20 – 7.08 (m, 4H), 7.01 – 6.91 (m, 2H), 5.28 – 5.09 (m, 2H), 3.84 (q, *J* = 7.2 Hz, 1H), 1.58 (d, *J* = 7.2 Hz, 3H).

^{13}C NMR (150 MHz, CDCl$_3$): δ 186.4, 173.7, 160.5, 158.9, 152.0, 142.4, 141.7, 141.7, 139.9, 136.5, 136.0, 135.5, 135.3, 133.7, 133.4, 130.8, 130.8, 129.3, 129.0, 129.0, 128.5, 127.7, 127.7, 123.7, 123.7, 115.4, 115.2, 66.4, 45.1, 18.3.

HRESIMS: m/z 439.1706 $[M + H]^+$ (calcd for $C_{29}H_{24}FO_3^+$, 439.1704).

Synthesis of 9k

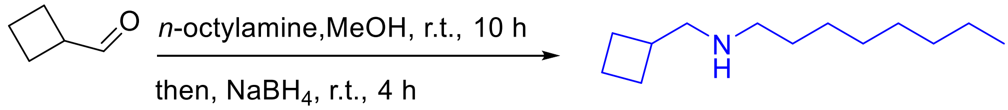

S14 **9k**

To a solution of commercially available compound **S14** (5 mmol, 1.0 equiv.) in MeOH (50 mL) was added the *n*-octylamine (20 mmol, 4.0 equiv.) at r.t. After stirring overnight, the reaction mixture was cooled to 0°C. $NaBH_4$ (285.0 mg, 7.5 mmol, 1.5 equiv.) was slowly added in portions. Then, the reaction mixture was stirred at r.t. for another 4 hours. After the reaction was completed, the reaction mixture was quenched with water. MeOH was removed under reduced pressure and extract with EtOAc. The combined organic layer was dried over anhydrous Na_2SO_4 and filtered. The solvent was removed

under reduced pressure. After passing through a flash chromatography (PE/EtOAc = 5:1 to 1:2, with the addition of 0.01% TEA), **9k** (670.5 mg) was obtained in 68% yield.

Physical State: yellow oil.

TLC: R_f = 0.32 (PE/EtOAc = 1:1).

^{1}H NMR (600 MHz, $CDCl_3$): δ 2.59 (d, *J* = 7.3 Hz, 2H), 2.57 – 2.51 (m, 2H), 2.44 (hept, *J* = 7.9 Hz, 1H), 2.08 – 1.99 (m, 2H), 1.93 – 1.77 (m, 2H), 1.67 – 1.58 (m, 2H), 1.44 (t, *J* = 7.3 Hz, 2H), 1.32 – 1.20 (m, 11H), 0.85 (t, *J* = 7.0 Hz, 3H).

^{13}C NMR (150 MHz, $CDCl_3$): δ 56.4, 50.3, 35.9, 32.0, 30.3, 29.7, 29.4, 27.5, 26.6, 22.8, 18.7, 14.2.

HRESIMS: m/z 198.2215 $[M + H]^+$ (calcd for $C_{13}H_{28}N^+$, 198.2216).

Synthesis of 9m

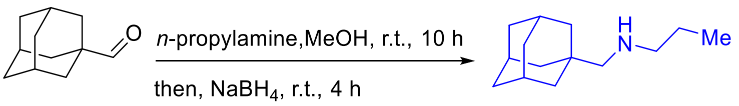

S15 **9m**

9m was prepared following the synthesis procedure of **9k** (455.2 mg) with the yield of 72%. The elution condition for flash chromatography: PE/EtOAc = 2:1 to 1:3, with the addition of 0.01% TEA.

Physical State: white powder.

TLC: R_f = 0.33 (PE/EtOAc = 1:1).

^{1}H NMR (600 MHz, $CDCl_3$): δ 2.48 (t, *J* = 7.2 Hz, 2H), 2.17 (d, *J* = 4.8 Hz, 2H), 1.89 (d, *J* = 5.7 Hz, 3H), 1.65 (s, 3H), 1.58 (d, *J* = 12.4 Hz, 3H), 1.48 – 1.39 (m, 9H), 0.83 (t, *J* = 7.4 Hz, 3H).

^{13}C NMR (150 MHz, $CDCl_3$): δ 62.9, 52.9, 41.0, 37.3, 33.3, 28.5, 22.9, 11.7.

HRESIMS: m/z 208.2063 $[M + H]^+$ (calcd for $C_{13}H_{28}N^+$, 208.2060).

Synthesis of 9n

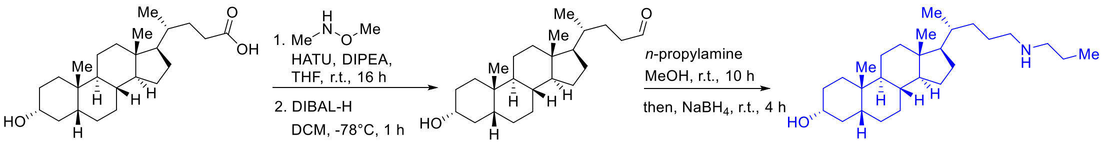

S16 **S17** **9n**

S17 was prepared following the literature protocol and spectral data matched reported values therein.[10] **9n** (455.3 mg) was prepared following the synthesis procedure of **9m** with the yield of 64%. The elution condition for flash chromatography: PE/EtOAc = 2:1 to 1:3, with the addition of 0.01% TEA.

Characteristics of 9n:

Physical State: white powder.

TLC: R_f = 0.23 (PE/EtOAc = 1:1).

^{1}H NMR (400 MHz, $CDCl_3$): δ 3.58 (tt, *J* = 10.6, 4.7 Hz, 1H), 2.54 (p, *J* = 8.0, 7.6 Hz, 4H), 2.10 – 2.02 (m, 1H), 1.97 – 1.91 (m, 1H), 1.88 – 1.58 (m, 5H), 1.50 (dt, *J* = 14.8, 7.3 Hz, 5H), 1.43 – 1.28 (m, 9H), 1.27 – 1.16 (m, 4H), 1.14 – 0.94 (m, 7H), 0.92 – 0.86 (m, 9H), 0.61 (s, 3H).

^{13}C NMR (100 MHz, $CDCl_3$): δ 71.7, 56.6, 56.3, 51.9, 50.6, 42.8, 42.2, 40.5, 40.3, 36.6, 35.9, 35.8, 35.5, 34.7, 33.6, 30.7, 28.4, 27.3, 26.6, 26.5, 24.3, 23.5, 23.2, 20.9, 18.8, 12.1, 11.9.

HRESIMS: m/z 404.3884 $[M + H]^+$ (calcd for $C_{27}H_{50}NO^+$, 404.3887).

Specific Rotation: $[\alpha]^{18}_D$ = +33 (*c* 0.015, MeOH).

General Procedure for Tropone Alkylation

General procedure D for tropone alkylation

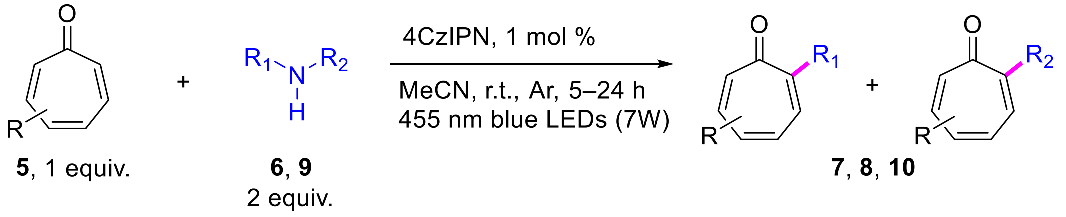

To the tropone derivatives **5** (0.283 mmol) in a 5 mL transparent test tube was added photocatalyst 4CzIPN (2.2 mg, 0.00283 mmol, 1 mol%) and magnetic stir bars. The tube was sealed with septum, degassed and backfilled with Ar. MeCN (2.2 mL) and amine (0.566 mmol) were added to the mixture through the syringe. The test tube was placed in the photoreactor and irradiated under 455 nm (7W) LEDs. Once the reaction reached to the end (either the starting materials totally consumed or no further products

converted), the solvent was removed and the crude was directly pass through flash chromatography to yield the title product**.**

Synthesis and Characteristics of Alkylated Tropones

Compound 7a

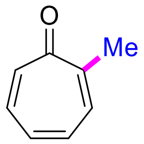

7a, 79%

Following general procedure D for 10 h with tropone and dimethylamine as starting material, compound **7a** was obtained in 79% yield (27.5 mg) as a known compound, with spectral data matched to reported values therein.[11] The elution condition for flash chromatography: PE/EtOAc = 4:1 to 1:1.

Physical State: yellow oil.

TLC: R_f = 0.77 (PE/EtOAc = 1:1).

^{1}H NMR (400 MHz, $CDCl_3$): δ 7.38 – 7.27 (m, 1H), 7.17 – 7.03 (m, 2H), 6.99 – 6.86 (m, 2H), 2.28 (s, 3H).

^{13}C NMR (125 MHz, $CDCl_3$): δ 187.6, 152.7, 140.1, 135.7, 135.3, 134.0, 132.6, 23.0.

HRESIMS: m/z 121.0647 $[M + H]^+$ (calcd for C_8H_9O, 121.0648).

Compound 7b

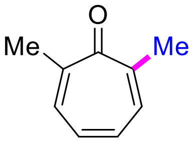

7b, 62%

Following the general procedure D for 5 h with **5b** (also **7a)** as starting material, compound **7b** was obtained in 62% yield (23.5 mg) identified as a known compound and spectral data matched reported values therein.[12] The elution condition for flash chromatography: PE/EtOAc = 6:1 to 2:1.

Physical State: yellow oil.

TLC: R_f = 0.43 (PE/EtOAc = 4:1).

Compound 7c

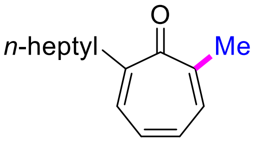

7c, 42%

Following the general procedure D for 8 h with **5c** as starting material, compound **7c** was obtained in 42% yield (25.9 mg). The elution condition for flash chromatography: PE/EtOAc = 4:1 to 1:1.

Physical State: yellow oil.

TLC: R_f = 0.89 (PE/EtOAc = 1:1).

^{1}H NMR (600 MHz, $CDCl_3$): δ 7.31 (dt, *J* = 8.2, 1.3 Hz, 1H), 7.27 – 7.24 (m, 1H), 6.91 – 6.82 (m, 2H), 2.71 – 2.66 (m, 2H), 2.31 (s, 3H), 1.41 – 1.20 (m, 10H), 0.87 (t, *J* = 7.0 Hz, 3H).

^{13}C NMR (125 MHz, $CDCl_3$): δ 186.5, 153.4, 150.0, 134.6, 134.4, 132.0, 131.8, 36.7, 32.0, 29.9, 29.4, 29.3, 23.8, 22.8, 14.2.

HRESIMS: m/z 219.1742 $[M + H]^+$ (calcd for $C_{15}H_{23}O^+$, 219.1743).

Compound 7d

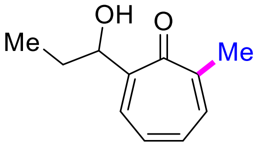

7d, 69%

Following the general procedure D for 5 h with **5d** as starting material, compound **7d** was obtained in 69% yield (34.8 mg). The elution condition for flash chromatography: PE/EtOAc = 2:1 to 1:2.

Physical State: yellow oil.

TLC: R_f = 0.79 (PE/EtOAc = 1:1).

^{1}H NMR (600 MHz, $CDCl_3$): δ 7.40 (dt, *J* = 8.4, 1.3 Hz, 1H), 7.35 (dd, *J* = 8.5, 1.4 Hz, 1H), 7.05 – 6.95 (m, 2H), 4.59 (d, *J* = 6.5 Hz, 1H), 3.89 (d, *J* = 7.6 Hz, 1H), 2.32 (s, 3H), 1.86 – 1.78 (m, 2H), 0.97 (t, *J* = 7.4 Hz, 3H).

^{13}C NMR (125 MHz, $CDCl_3$): δ 187.3, 151.7, 151.5, 135.5, 134.3, 133.5, 132.4, 78.0,

29.5, 23.4, 10.9.

HRESIMS: m/z 179.1068 $[M + H]^+$ (calcd for $C_{11}H_{15}O_2$, 179.1067).

Compound 7e

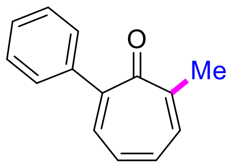

7e, 82%

Following the general procedure D for 5 h with **5e** as starting material, compound **7e** was obtained in 82% yield (45.5 mg). The elution condition for flash chromatography: PE/EtOAc = 6:1 to 2:1.

Physical State: yellowish powder.

TLC: R_f = 0.72 (PE/EA = 3:1).

^{1}H NMR (600 MHz, CDCl$_3$): δ 7.47 – 7.44 (m, 2H), 7.41 – 7.37 (m, 2H), 7.35 (ddt, *J* = 8.3, 2.4, 1.3 Hz, 3H), 7.01 – 6.89 (m, 2H), 2.36 (s, 3H).

^{13}C NMR (150 MHz, CDCl$_3$): δ 186.3, 152.5, 149.9, 141.2, 136.1, 134.5, 132.6, 131.7, 129.4, 128.1, 128.1, 23.8.

HRESIMS: m/z 197.0961 $[M + H]^+$ (calcd for $C_{14}H_{13}O^+$, 197.0961).

Compound 7f

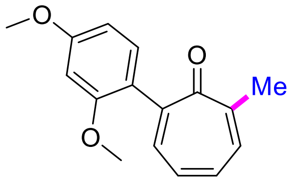

7f, 31%

Following the general procedure D for 6 h with **5f** as starting material, compound **7f** was obtained in 31% yield (22.5 mg). The elution condition for flash chromatography: PE/EtOAc = 3:1 to 1:1.

Physical State: yellowish powder.

TLC: R_f = 0.57 (PE/EtOAc = 3:2).

^{1}H NMR (600 MHz, CDCl$_3$): δ 7.29 – 7.23 (m, 1H), 7.19 (d, *J* = 8.3 Hz, 1H), 6.93 –

6.82 (m, 2H), 6.54 (dd, *J* = 8.2, 2.4 Hz, 1H), 6.50 (d, *J* = 2.4 Hz, 1H), 3.83 (s, 3H), 3.75 (s, 3H), 2.35 (s, 3H).

^{13}C NMR (150 MHz, CDCl$_3$): δ 187.3, 161.1, 157.3, 150.1, 147.4, 135.3, 133.5, 132.1, 131.3, 131.2, 124.1, 104.8, 99.1, 55.9, 55.6, 23.4.

HRESIMS: m/z 257.1171 $[M + H]^+$ (calcd for $C_{16}H_{17}O_3^+$, 257.1172).

Compound 7g

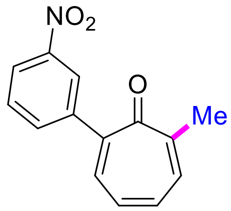

7g, 56%

Following the general procedure D for 8 h with **5g** as starting material, compound **7g** was obtained in 56% yield (38.2 mg). The elution condition for flash chromatography: PE/EtOAc = 3:1 to 1:2.

Physical State: yellowish oil.

TLC: R_f = 0.57 (PE/EtOAc = 1:2).

^{1}H NMR (400 MHz, CDCl$_3$): δ 8.32 (t, *J* = 2.0 Hz, 1H), 8.22 (ddd, *J* = 8.3, 2.3, 1.1 Hz, 1H), 7.81 (ddd, *J* = 7.7, 1.8, 1.1 Hz, 1H), 7.56 (t, *J* = 8.0 Hz, 1H), 7.46 – 7.30 (m, 2H), 7.02 (dd, *J* = 6.1, 3.7 Hz, 2H), 2.37 (d, *J* = 1.1 Hz, 3H).

^{13}C NMR (125 MHz, CDCl$_3$): δ 185.4, 153.4, 148.2, 147.5, 142.7, 136.8, 135.8, 135.1, 134.0, 131.7, 128.9, 124.6, 123.0, 23.8.

HRESIMS: m/z 242.0813 $[M + H]^+$ (calcd for $C_{14}H_{12}NO_3^+$, 242.0812).

Compound 7h

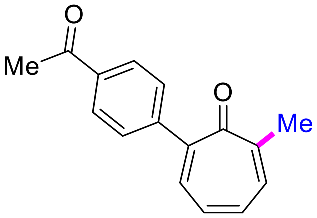

7h, 79%

Following the general procedure D for 8 h with **5h** as starting material, compound **7h** was obtained in 79% yield (53.2 mg). The elution condition for flash chromatography: PE/EtOAc = 2:1 to 1:2.

Physical State: yellowish powder.

TLC: R_f = 0.50 (PE/EtOAc = 1:2).

^{1}H NMR (500 MHz, $CDCl_3$): δ 8.01 – 7.95 (m, 2H), 7.57 – 7.51 (m, 2H), 7.42 – 7.31 (m, 2H), 7.04 – 6.93 (m, 2H), 2.62 (s, 3H), 2.36 (d, *J* = 1.1 Hz, 3H).

^{13}C NMR (125 MHz, $CDCl_3$): δ 197.9, 185.8, 153.0, 148.9, 146.1, 136.5, 136.4, 134.7, 133.4, 131.7, 129.7, 128.2, 26.8, 23.7.

HRESIMS: m/z 239.1067 $[M + H]^+$ (calcd for $C_{16}H_{15}O_2^+$, 239.1067).

Compound 7i

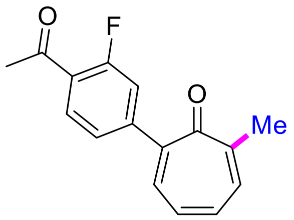

7i, 89%

Following the general procedure D for 6 h with **5i** as starting material, compound **7i** was obtained in 89% yield (64.5 mg). The elution condition for flash chromatography: PE/EtOAc = 2:1 to 1:1.

Physical State: yellowish powder.

TLC: R_f = 0.80 (PE/EtOAc = 2:3).

^{1}H NMR (600 MHz, $CDCl_3$): δ 7.91 (t, J = 7.9 Hz, 1H), 7.43 – 7.28 (m, 4H), 7.06 – 6.98 (m, 2H), 2.68 (d, J = 4.8 Hz, 3H), 2.39 (d, J = 1.1 Hz, 3H).

^{13}C NMR (125 MHz, $CDCl_3$): δ 195.8, 195.8, 185.5, 162.9, 160.9, 153.2, 148.0, 148.0, 147.5, 136.5, 134.9, 133.9, 131.6, 130.4, 130.3, 125.4, 125.4, 125.0, 124.9, 118.0, 117.8, 31.6, 31.6, 23.7.

^{19}F NMR (471 MHz, $CDCl_3$): δ −109.8.

HRESIMS: m/z 257.0974 $[M + H]^+$ (calcd for $C_{16}H_{14}FO_2^+$, 257.0972).

Compound 7j

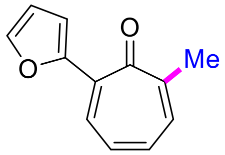

7j, 41%

Following the general procedure D for 10 h with **5j** as starting material, compound **7j** was obtained in 41% yield (21.6 mg). The elution condition for flash chromatography: PE/EtOAc = 3:1 to 1:1.

Physical State: yellow powder.

TLC: R_f = 0.59 (PE/EtOAc = 1:1).

^{1}H NMR (600 MHz, $CDCl_3$): δ 8.08 (d, J = 9.3 Hz, 1H), 7.72 (d, J = 3.5 Hz, 1H), 7.53 (dd, J = 1.8, 0.8 Hz, 1H), 7.37 (dq, J = 8.7, 1.2 Hz, 1H), 7.10 (t, J = 10.0 Hz, 1H), 6.94 (dd, J = 10.4, 8.9 Hz, 1H), 6.55 (dd, J = 3.5, 1.8 Hz, 1H), 2.41 (s, 3H).

^{13}C NMR (125 MHz, $CDCl_3$): δ 183.6, 151.2, 151.0, 143.6, 136.9, 134.4, 132.3, 131.8, 131.1, 116.2, 112.9, 24.2.

HRESIMS: m/z 187.0752 $[M + H]^+$ (calcd for $C_{12}H_{11}O_2^+$, 187.0754).

Compound 7k

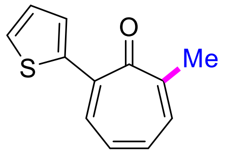

7k, 56%

Following the general procedure D for 10 h with **5k** as starting material, compound **7k** was obtained in 56% yield (32.0 mg). The elution condition for flash chromatography: PE/EtOAc = 3:1 to 1:1.

Physical State: yellow powder.

TLC: R_f = 0.55 (PE/EtOAc = 1:1).

^{1}H NMR (600 MHz, $CDCl_3$): δ 7.92 (d, J = 9.2 Hz, 1H), 7.62 (dt, J = 3.9, 1.0 Hz, 1H), 7.56 – 7.53 (m, 1H), 7.41 (dt, J = 8.5, 1.0 Hz, 1H), 7.13 (ddd, J = 5.0, 3.9, 0.9 Hz, 1H), 7.08 (t, J = 9.9 Hz, 1H), 7.01 – 6.96 (m, 1H), 2.44 (s, 3H).

^{13}C NMR (150 MHz, CDCl$_3$): δ 184.0, 150.7, 141.0, 140.3, 134.9, 132.5, 132.4, 131.5, 131.3, 127.9, 126.3, 24.1.

HRESIMS: m/z 203.0527 $[M + H]^+$ (calcd for $C_{12}H_{11}OS^+$, 203.0525).

Compound 7l

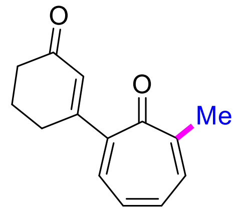

7l, 44%

Following the general procedure D for 10 h with **5l** as starting material, compound **7l** was obtained in 44% yield (26.6 mg). The elution condition for flash chromatography: PE/EtOAc = 3:1 to 1:1.

Physical State: yellow oil.

TLC: R_f = 0.80 (PE/EtOAc = 1:1).

^{1}H NMR (600 MHz, CDCl$_3$): δ 7.37 (dt, *J* = 8.2, 1.3 Hz, 1H), 7.23 (dd, *J* = 8.1, 1.6 Hz, 1H), 7.02 – 6.92 (m, 2H), 6.06 (d, *J* = 1.5 Hz, 1H), 2.58 (td, *J* = 6.0, 1.5 Hz, 2H), 2.49 (dd, *J* = 7.3, 6.0 Hz, 2H), 2.33 (d, *J* = 1.1 Hz, 3H), 2.12 (p, *J* = 6.3 Hz, 2H).

^{13}C NMR (125 MHz, CDCl$_3$): δ 200.0, 185.3, 165.8, 152.4, 150.7, 135.0, 134.2, 133.9, 131.8, 128.0, 37.9, 28.9, 23.4, 23.3.

HRESIMS: m/z 215.1067 $[M + H]^+$ (calcd for $C_{14}H_{15}O_2^+$, 215.1067).

Compounds 7ma/7mb

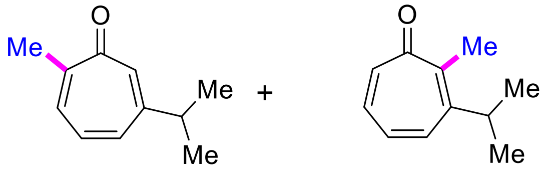

7ma, 34% **7mb**, 17%

Following to the general procedure D for 5 h with **5m** as starting material, compound **7ma** and **7mb** was obtained as an inseparable mixture, with **7ma** as a known compound.[13] The product ratio of **7ma**/**7mb** was 2:1 determined by NMR. The total

yield of **7ma** + **7mb** was 51% (23.4 mg). The elution condition for flash chromatography: PE/EtOAc = 4:1 to 1:1.

Characteristics of 7ma + **7mb**:

Physical State: pale yellow oil.

TLC: R_f = 0.43 (PE/EtOAc = 2:1).

^{1}H NMR (600 MHz, $CDCl_3$): δ 7.24 – 7.19 (m, 2H), 7.03 – 6.92 (m, 5H), 6.89 – 6.81 (m, 5H), 3.34 (dq, *J* = 13.7, 6.8 Hz, 1H), 2.75 (hept, *J* = 6.9 Hz, 2H), 2.31 (s, 3H), 2.25 (d, *J* = 1.1 Hz, 6H), 1.26 – 1.18 (m, 18H).

^{13}C NMR (150 MHz, $CDCl_3$): δ 188.9, 187.3, 156.2, 152.9, 151.9, 138.8, 136.8, 134.6, 134.3, 133.6, 132.7, 131.4, 38.2, 33.0, 23.0, 22.7, 22.1, 17.1.

HRESIMS: m/z 163.1116 $[M + H]^+$ (calcd for $C_{11}H_{15}O^+$, 163.1117).

Compound 7n

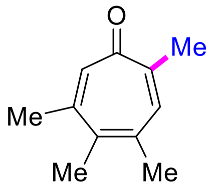

7n, 57%

Following the general procedure D for 8 h with **5n** as starting material, compound **7n** was obtained in 57% yield (26.1 mg). The elution condition for flash chromatography: PE/EtOAc = 2:1 to 1:2.

Physical State: yellowish oil.

TLC: R_f = 0.47 (PE/EtOAc = 1:1).

^{1}H NMR (600 MHz, $CDCl_3$): δ 7.25 (s, 1H), 7.03 (s, 1H), 2.31 (s, 3H), 2.31 (s, 3H), 2.23 (s, 3H), 2.17 (s, 3H).

^{13}C NMR (125 MHz, $CDCl_3$): δ 185.7, 147.7, 147.1, 140.8, 140.4, 139.6, 137.7, 27.6, 26.6, 22.3, 20.5.

HRESIMS: m/z 163.1115 $[M + H]^+$ (calcd for $C_{11}H_{15}O^+$, 163.1117).

Compound 7o

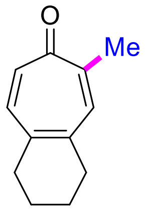

7o, 63%

Following the general procedure D for 8 h with **5o** as starting material, compound **7o** was obtained in 63% yield (31.0 mg). The elution condition for flash chromatography: PE/EtOAc = 2:1 to 1:2.

Physical State: yellowish oil.

TLC: R_f = 0.55 (PE/EtOAc = 1:2).

^{1}H NMR (600 MHz, $CDCl_3$): δ 7.14 (s, 1H), 6.94 – 6.83 (m, 2H), 2.66 (q, J = 5.2 Hz, 4H), 2.23 (s, 3H), 1.74 (p, J = 3.2 Hz, 4H).

^{13}C NMR (125 MHz, $CDCl_3$): δ 186.5, 147.3, 141.6, 141.1, 141.0, 140.4, 136.1, 34.1, 33.0, 23.0, 22.4, 22.2.

HRESIMS: m/z 175.1116 $[M + H]^+$ (calcd for $C_{12}H_{15}O^+$, 175.1117).

Compounds 7pa/7pb

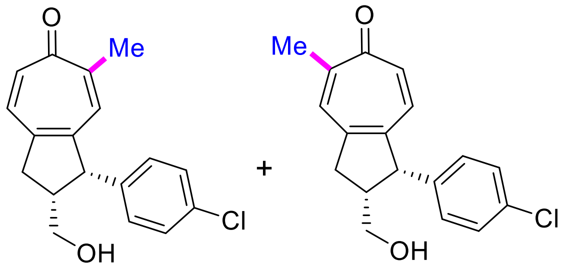

7pa, 34% **7pb**, 33%

Following the general procedure D for 7 h with **5p** as starting material, compound **7pa** was obtained in 34% yield (29.2 mg), while compound **7pb** was obtained in 33% yield (27.8 mg). During the preparation of the starting material using the method reported in the literature, it was noted that some impurities were the result of the formation of epimers during the diastereoselective synthesis.[9] The elution condition for flash chromatography: PE/EtOAc = 2:1 to 1:2.

Characteristics of 7pa:

Physical State: pale yellow powder.

TLC: R_f = 0.73 (PE/EtOAc = 2:3).

^{1}H NMR (600 MHz, $CDCl_3$): δ 7.38 (s, 1H), 7.29 – 7.26 (m, 2H), 6.97 (d, *J* = 8.0 Hz, 2H), 6.90 (d, *J* = 12.1 Hz, 1H), 6.82 (d, *J* = 12.1 Hz, 1H), 4.45 (d, *J* = 8.6 Hz, 1H), 3.35 (t, *J* = 8.4 Hz, 1H), 3.29 (t, *J* = 8.8 Hz, 1H), 3.04 (dd, *J* = 16.0, 7.4 Hz, 1H), 2.99 – 2.85 (m, 2H), 2.29 (s, 3H).

^{13}C NMR (125 MHz, $CDCl_3$): δ 186.8, 151.0, 146.6, 146.6, 138.5, 137.7, 134.9, 134.4, 133.4, 130.5, 129.1, 63.4, 56.6, 44.0, 40.4, 23.1.

HRESIMS: m/z 301.0990 $[M + H]^+$ (calcd for $C_{18}H_{18}ClO_2^+$, 301.0990).

Specific Rotation: $[\alpha]^{18}_D$ = −45 (*c* 0.06, MeOH).

Characteristics of 7pb:

Physical State: pale yellow powder.

TLC: R_f = 0.71 (PE/EtOAc = 2:3).

^{1}H NMR (600 MHz, $CDCl_3$): δ 7.29 (d, *J* = 8.1 Hz, 2H), 7.17 (d, *J* = 12.1 Hz, 1H), 7.06 – 6.96 (m, 4H), 4.42 (d, *J* = 8.7 Hz, 1H), 3.39 – 3.33 (m, 1H), 3.29 (ddd, *J* = 10.9, 7.5, 4.2 Hz, 1H), 3.03 (dd, *J* = 16.3, 8.0 Hz, 1H), 2.98 – 2.92 (m, 1H), 2.86 (dd, *J* = 16.3, 9.2 Hz, 1H), 2.15 (s, 3H).

^{13}C NMR (150 MHz, $CDCl_3$): δ 186.9, 150.7, 148.2, 145.1, 138.8, 137.6, 134.8, 134.5, 133.4, 130.6, 129.1, 63.4, 57.5, 44.1, 39.4, 22.9.

HRESIMS: m/z 301.0993 $[M + H]^+$ (calcd for $C_{18}H_{18}ClO_2^+$, 301.0990).

Specific Rotation: $[\alpha]^{18}_D$ = −40 (*c* 0.025, MeOH).

Compound 7q

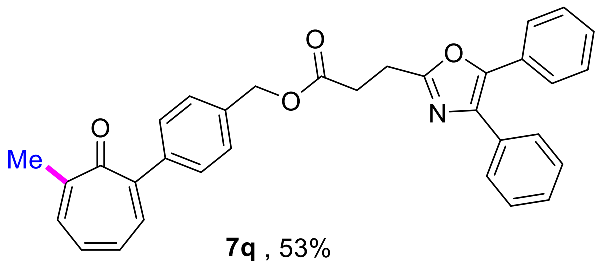

7q , 53%
from *oxaprozin*

Following the general procedure D for 7 h with **5q** as starting material, compound **7q** was obtained in 53% yield (75.2 mg). The elution condition for flash chromatography: PE/EtOAc = 3:1 to 1:1.

Physical State: pale yellow powder.

TLC: R_f = 0.71 (PE/EtOAc = 1:1).

^{1}H NMR (600 MHz, $CDCl_3$): δ 7.66 – 7.59 (m, 2H), 7.59 – 7.53 (m, 2H), 7.44 – 7.24 (m, 12H), 7.01 – 6.87 (m, 2H), 5.20 (s, 2H), 3.22 (t, *J* = 7.4 Hz, 2H), 2.98 (t, *J* = 7.4 Hz, 2H), 2.35 (d, *J* = 1.1 Hz, 3H).

^{13}C NMR (150 MHz, $CDCl_3$): δ 186.1, 172.0, 161.8, 152.5, 149.3, 145.6, 141.2, 136.1, 135.6, 135.3, 134.5, 132.8, 132.6, 131.7, 129.7, 129.1, 128.8, 128.7, 128.6, 128.2, 128.1, 127.9, 126.6, 66.5, 31.3, 23.7, 23.7.

HRESIMS: m/z 502.2013 $[M + H]^+$ (calcd for $C_{33}H_{28}NO_4^+$, 502.2013).

Compound 7r

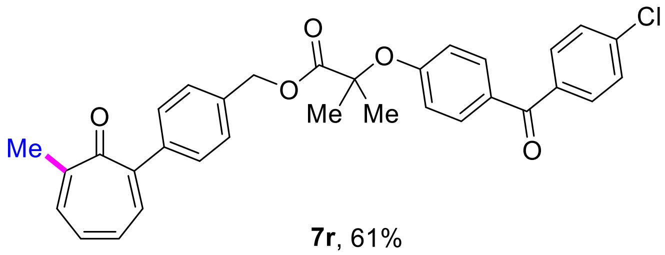

7r, 61%
from *fenofibric acid*

Following the general procedure D for 7 h with **5r** as starting material, compound **7r** was obtained in 61% yield (90.8 mg). The elution condition for flash chromatography: PE/EtOAc = 3:1 to 1:1.

Physical State: yellow solid.

TLC: R_f = 0.73 (PE/EtOAc = 1:1).

^{1}H NMR (600 MHz, CDCl$_3$): δ 7.71 – 7.61 (m, 4H), 7.39 (dd, *J* = 9.5, 8.3 Hz, 4H), 7.35 – 7.33 (m, 1H), 7.30 (dd, *J* = 7.7, 2.0 Hz, 1H), 7.27 (d, *J* = 7.6 Hz, 2H), 6.98 – 6.89 (m, 2H), 6.81 – 6.76 (m, 2H), 5.22 (s, 2H), 2.32 (s, 3H), 1.69 (s, 6H).

^{13}C NMR (150 MHz, CDCl$_3$): δ 194.3, 186.0, 173.6, 159.7, 152.6, 149.2, 141.5, 138.5, 136.5, 136.1, 134.8, 134.6, 132.9, 132.1, 131.7, 131.3, 130.5, 129.6, 128.6, 128.2, 117.4, 79.6, 67.2, 25.6, 23.7.

HRESIMS: m/z 527.1619 $[M + H]^+$ (calcd for $C_{32}H_{28}ClO_5^+$, 527.1620).

Compound 7s

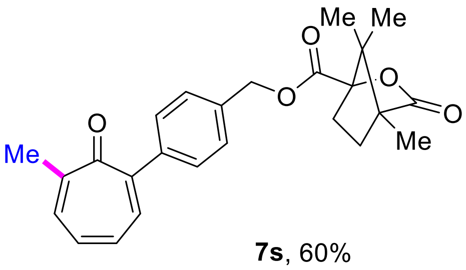

7s, 60%
from (−)-*camphanic acid*

Following the general procedure D for 10 h with **5s** as starting material, compound **7s** was obtained in 60% yield (69.0 mg). The elution condition for flash chromatography: PE/EtOAc = 3:1 to 1:1.

Physical State: white solid.

TLC: R_f = 0.83 (PE/EtOAc = 1:1).

^{1}H NMR (600 MHz, CDCl$_3$): δ 7.46 (dd, *J* = 8.1, 1.5 Hz, 2H), 7.39 (d, *J* = 8.4 Hz, 2H), 7.35 (ddd, *J* = 9.7, 8.1, 1.5 Hz, 2H), 7.01 – 6.91 (m, 2H), 5.28 (s, 2H), 2.48 – 2.40 (m, 1H), 2.35 (s, 3H), 2.05 (ddd, *J* = 13.7, 9.5, 4.7 Hz, 1H), 1.95 – 1.87 (m, 1H), 1.69 (ddd, *J* = 13.4, 9.4, 4.2 Hz, 1H), 1.10 (s, 3H), 1.04 (s, 3H), 0.94 (s, 3H).

^{13}C NMR (150 MHz, CDCl$_3$): δ 186.0, 178.2, 167.5, 152.6, 149.3, 141.5, 136.2, 134.9, 134.6, 132.9, 131.7, 129.7, 128.0, 91.2, 67.1, 54.9, 54.4, 30.8, 29.1, 23.7, 16.9, 16.9, 9.8.

HRESIMS: m/z 407.1852 $[M + H]^+$ (calcd for $C_{25}H_{27}O_5^+$, 407.1853).

Specific Rotation: $[\alpha]^{18}_D = -16$ (*c* 0.03, MeOH).

Compound 7t

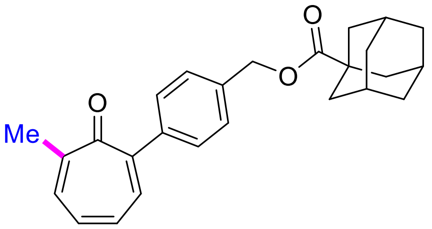

7t, 88%
from *adamantane carboxylic acid*

Following the general procedure D for 6 h with **5t** as starting material, compound **7t** was obtained in 88% yield (96.7 mg). The elution condition for flash chromatography: PE/EtOAc = 3:1 to 1:1.

Physical State: white solid.

TLC: R_f = 0.74 (PE/EtOAc = 1:1).

^{1}H NMR (600 MHz, $CDCl_3$): δ 7.48 – 7.43 (m, 2H), 7.38 – 7.33 (m, 4H), 7.00 – 6.90 (m, 2H), 5.12 (s, 2H), 2.36 (s, 3H), 2.02 (p, *J* = 3.0 Hz, 3H), 1.95 – 1.89 (m, 3H), 1.77 – 1.67 (m, 6H).

^{13}C NMR (150 MHz, $CDCl_3$): δ 186.2, 177.6, 152.5, 149.5, 140.9, 136.4, 136.0, 134.5, 132.7, 131.7, 129.6, 127.5, 65.7, 41.0, 39.0, 36.7, 28.1, 23.8.

HRESIMS: m/z 389.2122 $[M + H]^+$ (calcd for $C_{26}H_{29}O_3^+$, 389.2111).

Compound 7u

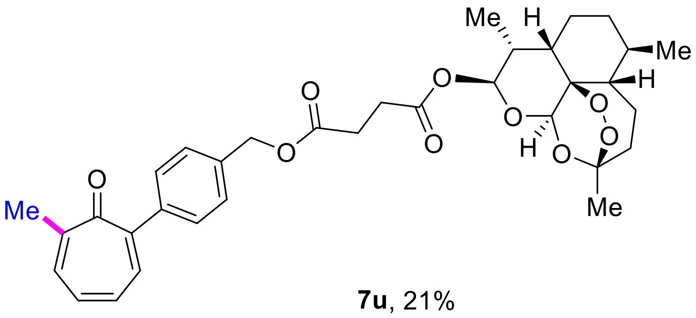

7u, 21%
from *artesunate*

Following the general procedure D for 6 h with **5u** as starting material, compound **7u** was obtained in 21% yield (35.2 mg). The elution condition for flash chromatography: PE/EtOAc = 3:1 to 1:1.

Physical State: white powder.

TLC: R_f = 0.74 (PE/EtOAc = 1:1).

^{1}H NMR (600 MHz, $CDCl_3$): δ 7.48 – 7.41 (m, 2H), 7.37 (d, *J* = 8.2 Hz, 2H), 7.36 – 7.32 (m, 2H), 7.00 – 6.90 (m, 2H), 5.80 (d, *J* = 9.8 Hz, 1H), 5.43 (s, 1H), 5.19 – 5.12 (m, 2H), 2.80 – 2.71 (m, 3H), 2.71 – 2.63 (m, 1H), 2.61 – 2.52 (m, 1H), 2.42 – 2.36 (m, 1H), 2.35 (s, 3H), 2.04 – 2.00 (m, 1H), 1.89 (ddt, *J* = 13.7, 6.8, 3.6 Hz, 1H), 1.77 (dq, *J* = 13.6, 3.7 Hz, 1H), 1.71 (dq, *J* = 13.5, 3.4 Hz, 1H), 1.63 – 1.60 (m, 1H), 1.53 – 1.46 (m, 1H), 1.43 (s, 3H), 1.40 – 1.28 (m, 3H), 1.05 – 0.97 (m, 1H), 0.97 (s, 3H), 0.84 (d, *J* = 7.1 Hz, 3H).

^{13}C NMR (150 MHz, $CDCl_3$): δ 186.1, 172.1, 171.2, 152.5, 149.4, 141.2, 136.1, 135.6, 134.5, 132.8, 131.7, 129.7, 128.0, 104.6, 92.4, 91.7, 80.3, 66.4, 51.7, 45.4, 37.4, 36.4, 34.2, 32.0, 29.4, 29.1, 26.1, 24.7, 23.7, 22.1, 20.4, 12.2.

HRESIMS: m/z 615.2570 $[M + Na]^+$ (calcd for $C_{34}H_{40}NaO_9^+$, 615.2565); m/z 610.3010 $[M + NH_4]^+$ (calcd for $C_{34}H_{44}O_9^+$, 610.3011).

Specific Rotation: $[\alpha]^{18}_D$ = +15 (*c* 0.02, MeOH).

Compound 7v

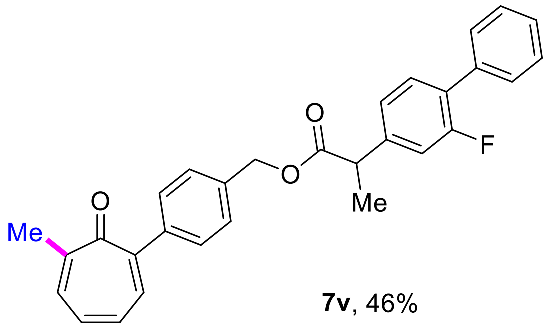

7v, 46%
from *flurbiprofen*

Following the general procedure D for 6 h with **5v** (0.1 mmol) as starting material, compound **7v** was obtained in 46% yield (20.8 mg). The elution condition for flash chromatography: PE/EtOAc = 3:1 to 1:1.

Physical State: yellowish solid.

TLC: R_f = 0.41 (PE/EtOAc = 2:1).

^{1}H NMR (600 MHz, $CDCl_3$): δ 7.56 – 7.52 (m, 2H), 7.46 – 7.28 (m, 10H), 7.18 – 7.10 (m, 2H), 6.98 – 6.90 (m, 2H), 5.23 – 5.10 (m, 2H), 3.81 (q, *J* = 7.1 Hz, 1H), 2.35 (s, 3H), 1.56 (d, *J* = 7.1 Hz, 3H).

^{13}C NMR (125 MHz, CDCl$_3$): δ 186.1, 173.9, 160.8, 158.8, 152.6, 149.4, 141.8, 141.8, 141.2, 136.1, 135.7, 135.6, 134.5, 132.8, 131.7, 131.0, 130.9, 129.6, 129.1, 129.1, 128.6, 127.8, 127.8, 123.8, 123.8, 115.5, 115.3, 66.6, 45.2, 23.8, 18.5.

^{19}F NMR (471 MHz, CDCl$_3$): δ −117.65.

HRESIMS: m/z 453.1860 [M + H]$^+$ (calcd for $C_{30}H_{26}FO_3^+$, 453.1860).

Compound 8b

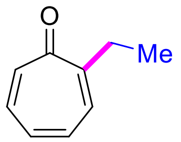

8b, 74%

Following the general procedure D for 10 h with tropone and diethylamine (**6b**) as starting material, compound **8b** was obtained in 74% yield (28.1 mg) as a known compound. The elution condition for flash chromatography: PE/EtOAc = 4:1 to 1:1.

Physical State: yellowish oil.

TLC: R_f = 0.47 (PE/EtOAc = 2:1).

^{1}H NMR (500 MHz, CD$_3$OD): δ 7.52 – 7.45 (m, 1H), 7.32 (ddd, J = 12.0, 8.0, 1.5 Hz, 1H), 7.17 (ddt, J = 9.6, 8.7, 1.1 Hz, 1H), 7.14 – 7.04 (m, 2H), 2.73 – 2.59 (m, 2H), 1.18 (t, J = 7.4 Hz, 3H).

^{13}C NMR (125 MHz, CD$_3$OD): δ 188.9, 158.4, 140.7, 138.2, 137.1, 136.1, 134.6, 29.6, 13.7.

HRESIMS: m/z 135.0803 [M + H]$^+$ (calcd for $C_9H_{11}O^+$, 135.0804).

Compound 8c

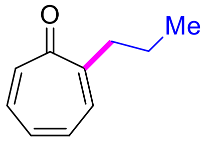

8c, 58%

Following the general procedure D for 8 h with tropone and dipropylamine (**6c**) as starting material, compound **8c** was obtained in 56% yield (23.5 mg) as a known compound.[14] The elution condition for flash chromatography: PE/EtOAc = 4:1 to 1:1.

Physical State: yellow oil.

TLC: R_f = 0.68(PE/EtOAc = 1:1).

^{1}H NMR (400 MHz, CDCl$_3$): δ 7.24 – 7.20 (m, 1H), 7.11 – 7.00 (m, 2H), 6.97 – 6.83 (m, 2H), 2.60 (dd, *J* = 8.6, 6.6 Hz, 2H), 1.65 – 1.50 (m, 2H), 0.94 (t, *J* = 7.4 Hz, 3H).

^{13}C NMR (100 MHz, CDCl$_3$): δ 187.2, 156.0, 140.4, 135.4, 134.9, 133.9, 132.6, 37.6, 22.1, 14.2.

HRESIMS: m/z 149.0958 [M + H]$^+$ (calcd for $C_{10}H_{13}O^+$, 149.0961).

Compound 8d

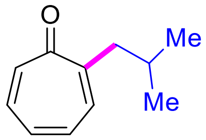

8d, 54%

Following the general procedure D for 5 h with tropone and diisobutylamine (**6d**) as starting material, compound **8d** was obtained in 54% yield (24.7 mg) as a known compound. The elution condition for flash chromatography: PE/EtOAc = 4:1 to 1:1.

Physical State: yellowish powder.

TLC: R_f = 0.62 (PE/EtOAc = 3:2).

^{1}H NMR (600 MHz, CDCl$_3$): δ 7.19 (d, *J* = 8.5 Hz, 1H), 7.10 – 7.01 (m, 2H), 6.96 – 6.86 (m, 2H), 2.52 (d, *J* = 7.1 Hz, 2H), 2.02 – 1.92 (m, 1H), 0.91 (dd, *J* = 6.6, 1.0 Hz, 6H).

^{13}C NMR (150 MHz, CDCl$_3$): δ 187.4, 155.1, 140.5, 135.8, 135.3, 133.8, 132.6, 45.0, 27.6, 22.8.

HRESIMS: m/z 163.1116 [M + H]+ (calcd for $C_{11}H_{15}O^+$, 163.1117).

Compound 8e

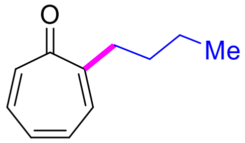

8e, 70%

Following the general procedure D for 10 h with tropone and dibutylamine (**6e**) as

starting material, compound **8e** was obtained in 70% yield (32.1 mg) as a known compound. The elution condition for flash chromatography: PE/EtOAc = 4:1 to 1:1.

Physical State: yellowish oil.

TLC: R_f = 0.62 (PE/EtOAc = 3:2).

^{1}H NMR (600 MHz, CDCl$_3$): δ 7.23 (dd, *J* = 8.7, 1.1 Hz, 1H), 7.11 – 7.02 (m, 2H), 6.97 – 6.86 (m, 2H), 2.66 – 2.62 (m, 2H), 1.59 – 1.49 (m, 2H), 1.38 (h, *J* = 7.4 Hz, 2H), 0.92 (t, *J* = 7.3 Hz, 3H).

^{13}C NMR (150 MHz, CDCl$_3$): δ 187.2, 156.4, 140.5, 135.4, 134.8, 134.0, 132.5, 35.4, 31.2, 22.9, 14.1.

HRESIMS: m/z 163.1117 [M + H]$^+$ (calcd for $C_{11}H_{15}O$, 163.1117).

Compound 8f

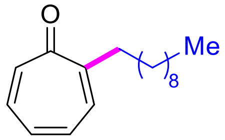

8f, 25%

Following the general procedure D with some modifications for 24 h with tropone (0.283 mmol, 30.0 mg) and N,N-didecylamine (**6f**, 0.283 mmol, 84.2 mg) as starting material, compound **8f** was obtained in 25% yield (17.4 mg). The elution condition for flash chromatography: PE/EtOAc = 4:1 to 1:1.

Physical State: yellowish solid.

TLC: R_f = 0.72 (PE/EtOAc = 1:1).

^{1}H NMR (400 MHz, CDCl$_3$): δ 7.23 (d, *J* = 8.4 Hz, 1H), 7.13 – 7.01 (m, 2H), 6.99 – 6.84 (m, 2H), 2.68 – 2.60 (m, 2H), 1.61 – 1.47 (m, 2H), 1.39 – 1.27 (m, 14H), 0.87 (t, *J* = 6.8 Hz, 3H).

^{13}C NMR (100 MHz, CDCl$_3$): δ 187.3, 156.4, 140.5, 135.4, 134.8, 134.0, 132.5, 35.7, 32.0, 29.8, 29.7, 29.7, 29.6, 29.5, 29.0, 22.8, 14.2.

HRESIMS: m/z 247.2054 [M + H]$^+$ (calcd for $C_{17}H_{27}O^+$, 247.2056).

Compound 8g

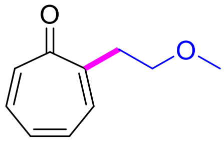

8g, 63%

Following the general procedure D for 6 h with tropone and bis(2-methoxyethyl)amine (**6g**) as starting material, compound **8g** was obtained in 63% yield (29.3 mg). The elution condition for flash chromatography: PE/EtOAc = 4:1 to 1:1.

Physical State: yellowish oil.

TLC: R_f = 0.62 (PE/EtOAc = 1:1).

^{1}H NMR (600 MHz, $CDCl_3$): δ 7.33 (dq, *J* = 8.5, 0.9 Hz, 1H), 7.14 – 7.04 (m, 2H), 6.99 – 6.90 (m, 2H), 3.62 (t, *J* = 6.3 Hz, 2H), 3.33 (s, 3H), 2.91 (td, *J* = 6.3, 0.8 Hz, 2H).

^{13}C NMR (150 MHz, $CDCl_3$): δ 187.2, 152.4, 140.8, 136.5, 135.7, 134.0, 133.1, 70.9, 58.7, 36.2.

HRESIMS: m/z 165.0910 $[M + H]^+$ (calcd for $C_{10}H_{13}O_2$, 165.0910).

Compound 8h

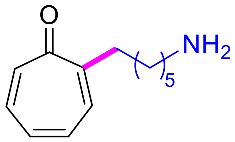

8h, 32%

Following the general procedure D for 8 h with tropone and azepane (**6h**) as starting material, compound **8h** was obtained in 32% yield (18.6 mg). The elution condition for flash chromatography: PE/EtOAc = 1:1 to 1:4 with the addition of 0.01% TEA.

Physical State: yellow oil.

TLC: R_f = 0.12 (DCM/MeOH = 10:1).

^{1}H NMR (600 MHz, $CDCl_3$): δ 7.23 (d, *J* = 8.7 Hz, 1H), 7.12 – 7.02 (m, 2H), 6.99 – 6.86 (m, 2H), 2.71 – 2.59 (m, 4H), 2.03 (s, 2H), 1.60 – 1.52 (m, 2H), 1.45 (p, *J* = 7.1 Hz, 2H), 1.36 (tq, *J* = 9.0, 5.5, 4.1 Hz, 4H).

^{13}C NMR (150 MHz, $CDCl_3$): δ 187.3, 156.3, 140.5, 135.5, 134.9, 134.0, 132.6, 42.2, 35.7, 33.5, 29.5, 28.9, 26.8.

HRESIMS: m/z 206.1537 [M + H]$^+$ (calcd for $C_{13}H_{20}NO^+$, 206.1539).

Compound 8i

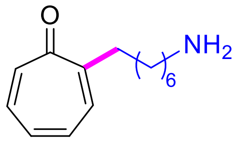

8i, 28%

Following the general procedure D for 12 h with tropone and azocane (**6i**) as starting material, compound **8i** was obtained in 28% yield (17.4 mg). The elution condition for flash chromatography: (PE/EtOAc = 1:1 to 1:4 with the addition of 0.01% TEA).

Physical State: yellowish oil.

TLC: R_f = 0.10 (DCM/MeOH = 10:1).

^{1}H NMR (400 MHz, CDCl$_3$): δ 7.23 (dd, *J* = 8.5, 1.2 Hz, 1H), 7.12 – 7.01 (m, 2H), 6.99 – 6.85 (m, 2H), 2.76 – 2.55 (m, 4H), 2.01 (s, 4H), 1.64 – 1.05 (m, 8H).

^{13}C NMR (100 MHz, CDCl$_3$): δ 187.3, 156.3, 140.5, 135.4, 134.9, 134.0, 132.6, 42.1, 35.7, 33.4, 29.7, 29.4, 28.9, 26.9.

HRESIMS: m/z 220.1696 [M + H]$^+$ (calcd for $C_{14}H_{22}NO^+$, 220.1696).

Compound 8j

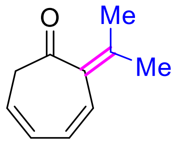

8j, 45%

Following the general procedure D for 10 h with tropone and *N*-(Propan-2-yl)propan-2-amineas (**6j**) as starting material, compound **8j** was obtained in 45% yield (18.8 mg). The elution condition for flash chromatography: (PE/EtOAc = 4:1 to 1:1).

Physical State: yellowish oil.

TLC: R_f = 0.88 (PE/EA = 3:2).

^{1}H NMR (600 MHz, CDCl$_3$): δ 6.47 (d, *J* = 11.7 Hz, 1H), 6.18 (dd, *J* = 9.8, 5.4 Hz, 1H), 6.09 (dd, *J* = 11.7, 5.4 Hz, 1H), 5.82 (dt, *J* = 9.9, 6.9 Hz, 1H), 3.18 (d, *J* = 6.9 Hz, 2H), 2.17 (s, 3H), 2.04 (s, 3H).

^{13}C NMR (150 MHz, CDCl$_3$): δ 194.5, 152.0, 134.8, 128.3, 125.6, 125.4, 123.6, 45.5, 24.5, 24.2.

HRESIMS: m/z 149.0960 [M + H]$^+$ (calcd for $C_{10}H_{13}O^+$, 149.0961).

Compound 8hs

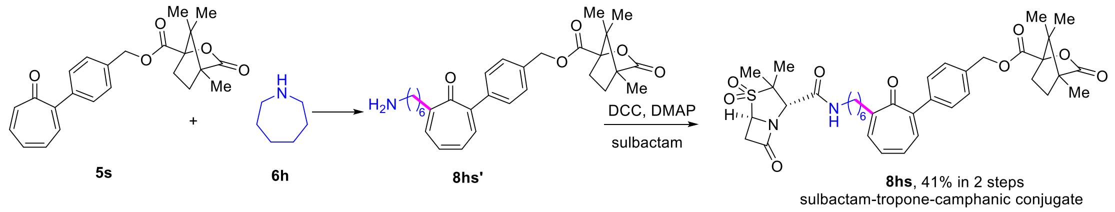

Following the general procedure D with several modifications for 10 h with tropone derivatives **5s** (0.1 mmol) and azocane **6h** (0.1 mmol) as starting materials for 7 h, compound **8hs′** was obtained. Then, compound **8hs′** was treated with sulbactam (1.0 equiv.), DCM (0.06M), dicyclohexyl carbodiimide (1.0 equiv.) and DMAP (cat. amount), and the mixture was stirred for 5h. After passing through a flash chromatography (PE/EtOAc = 2:1 to 2:3), **8hs** was obtained (28.9 mg, 41% in 2 steps).

Physical State: yellowish solid.

TLC: R_f = 0.32 (PE/EtOAc = 1:1).

^{1}H NMR (600 MHz, CDCl$_3$): δ 7.45 (d, J = 7.9 Hz, 2H), 7.40 (d, J = 7.9 Hz, 2H), 7.33 (dd, J = 7.1, 2.6 Hz, 1H), 7.30 – 7.26 (m, 1H), 7.01 – 6.93 (m, 2H), 6.65 (d, J = 6.3 Hz, 1H), 5.29 (s, 2H), 4.52 (dd, J = 4.7, 2.1 Hz, 1H), 4.16 (s, 1H), 3.46 – 3.34 (m, 2H), 3.30 (dq, J = 13.4, 6.7 Hz, 1H), 3.19 (dq, J = 13.0, 6.5 Hz, 1H), 2.70 (t, J = 7.7 Hz, 2H), 2.45 (ddd, J = 14.2, 10.7, 4.1 Hz, 1H), 2.38 – 2.17 (m, 1H), 2.08 – 2.01 (m, 1H), 1.93 (ddd, J = 15.4, 10.7, 4.6 Hz, 1H), 1.74 – 1.68 (m, 1H), 1.66 (s, 3H), 1.60 – 1.57 (m, 2H), 1.50 (p, J = 6.9 Hz, 2H), 1.40 (s, 3H), 1.37 (dd, J = 7.4, 4.0 Hz, 3H), 1.11 (s, 3H), 1.05 (s, 3H), 0.94 (s, 3H).

^{13}C NMR (125 MHz, CDCl$_3$): δ 186.3, 178.3, 172.4, 167.5, 166.3, 156.0, 149.6, 141.6, 136.1, 135.1, 134.5, 133.1, 131.9, 129.7, 128.1, 91.2, 67.0, 64.2, 63.7, 60.5, 55.0, 54.5, 39.5, 38.2, 36.2, 30.8, 29.5, 29.1, 29.1, 28.8, 26.1, 19.6, 18.5, 17.0, 16.9, 9.8.

HRESIMS: m/z 707.2999 [M + H]$^+$ (calcd for $C_{38}H_{47}N_2O_9S^+$, 707.2997).

Specific Rotation: $[\alpha]^{18}_D$ = +133 (c 0.025, MeOH).

Compound 9a to 7a and 8b

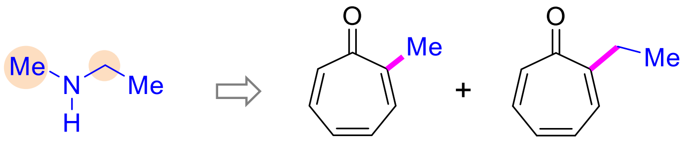

9a **7a**, 41% **8b**, 33%

Following the general procedure D for 6 h with tropone and **9a** as starting materials, compound **7a** was obtained in 41% yield (13.9 mg), while compound **8b** was obtained in 33% yield (12.5 mg). The elution condition for flash chromatography: PE/EtOAc = 5:1 to 2:1.

Compound 9b to 7a and 10b

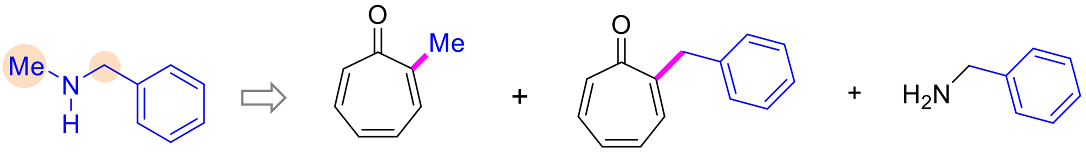

9b **7a**, 20% **10b**, 50% **10b'**

Following the general procedure D for 12 h with tropone and **9b** as starting materials, compound **10b** was obtained in 50% yield (27.7 mg), while compound **7a** was obtained in 20% yield (6.8 mg). Moreover, the benzylamine **10b′** was also detected by GC-MS, and the NMR spectrum was identical to that reported.[15] The elution condition for flash chromatography: PE/EtOAc = 5:1 to 2:1 to 1:6, with the addition of 0.01% TEA.

Characteristics of compound 10b:

Physical State: yellow oil.

TLC: R_f = 0.65 (PE/EtOAc = 1:1).

^{1}H NMR (600 MHz, $CDCl_3$): δ 7.31 (t, J = 7.6 Hz, 2H), 7.23 (dd, J = 12.7, 7.2 Hz, 3H), 7.13 – 7.06 (m, 3H), 6.92 – 6.89 (m, 2H), 3.98 (s, 2H).

^{13}C NMR (125 MHz, $CDCl_3$): δ 186.9, 155.0, 140.8, 139.3, 135.7, 135.7, 133.9, 132.9, 129.7, 128.7, 126.6, 40.7.

HRESIMS: m/z 197.0963 $[M + H]^+$ (calcd for $C_{14}H_{13}O^+$, 197.0963).

Compound 9c to 7a and 10c

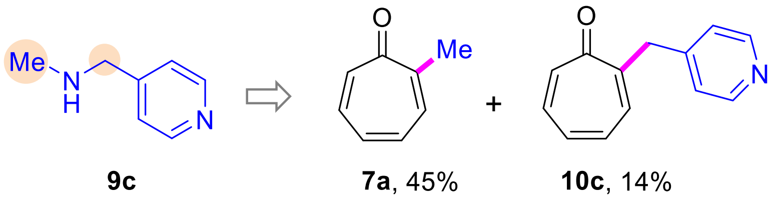

Following the general procedure D for 13 h with tropone and **9c** as starting materials, compound **10c** was obtained in 14% yield (8.0 mg), while compound **7a** was obtained in 45% yield (15.3 mg). The elution condition for flash chromatography: PE/EtOAc = 5:1 to 1:2.

Characteristics of compound 10c:

Physical State: brown oil.

TLC: R_f = 0.51 (PE/EtOAc = 1:1).

^{1}H NMR (600 MHz, $CDCl_3$): δ 8.51 (d, J = 5.0 Hz, 2H), 7.21 – 7.07 (m, 5H), 7.00 – 6.92 (m, 2H), 3.95 (s, 2H).

^{13}C NMR (150 MHz, $CDCl_3$): δ 186.5, 152.7, 150.0, 148.5, 141.1, 136.2, 135.9, 133.7, 133.6, 124.7, 40.5.

HRESIMS: m/z 198.0912 $[M + H]^+$ (calcd for $C_{13}H_{12}NO^+$, 198.0913).

Compound 9d to 7a and 10d

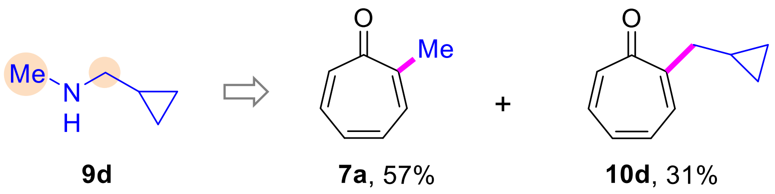

Following the general procedure D for 8 h with tropone and **9d** as starting materials, compound **10d** was obtained in 31% yield (14.0 mg), while compound **7a** was obtained in 57% yield (19.3 mg). The elution condition for flash chromatography: PE/EtOAc = 5:1 to 3:2.

Characteristics of compound 10d:

Physical State: pale yellow powder.

TLC: R_f = 0.62 (PE/EtOAc = 3:2).

^{1}H NMR (600 MHz, Chloroform-*d*): δ 7.45 (d, J = 8.8 Hz, 1H), 7.12 (ddd, J = 12.1,

7.6, 1.2 Hz, 1H), 7.07 (d, J = 11.9 Hz, 1H), 7.01 (dd, J = 10.9, 8.7 Hz, 1H), 6.92 (dd, J = 10.9, 7.7 Hz, 1H), 2.56 (d, J = 6.9 Hz, 2H), 1.00 (dtt, J = 14.8, 7.3, 3.7 Hz, 1H), 0.61 – 0.51 (m, 2H), 0.22 – 0.16 (m, 2H).

^{13}C NMR (150 MHz, CDCl$_3$): δ 187.5, 155.7, 140.5, 135.5, 134.3, 134.1, 132.6, 39.4, 9.7, 5.0.

HRESIMS: m/z 161.0961 $[M + H]^+$ (calcd for $C_{11}H_{13}O^+$, 161.0961).

Compound 9e to 7a and 8j

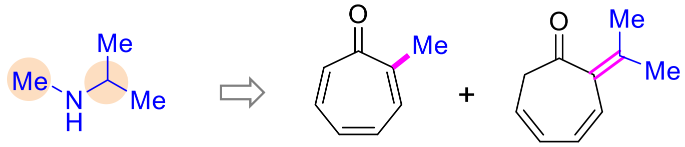

9e **7a**, 45% **8j**, 7%

Following the general procedure D for 8 h with tropone and **9e** as starting materials, compound **7a** was obtained in 45% yield (15.3 mg). while compound **8j** was obtained in 7% yield (2.9 mg). The elution condition for flash chromatography: PE/EtOAc = 5:1 to 3:2.

Compound 9f to 7a and 7b

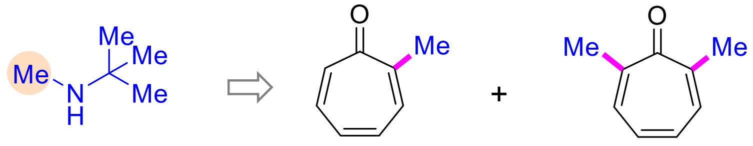

9f **7a**, 42% **7b**, 28%

Following the general procedure D for 6 h with tropone and **9f** as starting materials, compound **7a** was obtained in 42% yield (14.3 mg), while compound **7b** was obtained in 28% yield (10.6 mg). The elution condition for flash chromatography: PE/EtOAc = 5:1 to 3:2.

Compound 9g to 7a

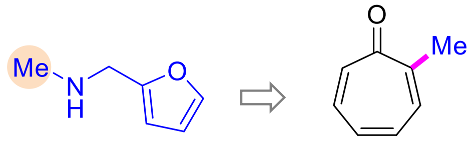

9g **7a**, 35%

Following the general procedure D for 8 h with tropone and **9g** as starting materials, compound **7a** was obtained in 35% yield (11.9 mg). The elution condition for flash chromatography: PE/EtOAc = 5:1 to 3:2.

Compound 9h to 7a

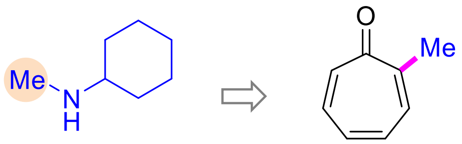

9h **7a**, 31%

Following the general procedure D for 6 h with tropone and **9h** as starting materials, compound **7a** was obtained in 31% yield (10.5 mg). The elution condition for flash chromatography: PE/EtOAc = 5:1 to 3:2.

Compound 9i to 7a

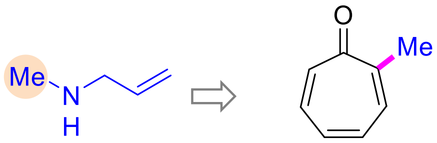

9i **7a**, 43%

Following the general procedure D for 9 h with tropone and **9i** as starting materials, compound **7a** was obtained in 43% yield (14.6 mg). The elution condition for flash chromatography: PE/EtOAc = 5:1 to 3:2.

Compound 9j to 7a

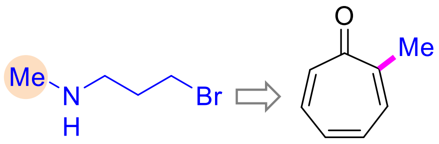

9j **7a**, 23%

Following the general procedure D for 9 h with tropone and **9j** as starting materials, compound **7a** was obtained in 23% yield (6.9 mg). Note: the reaction mixture was complex. The elution condition for flash chromatography: PE/EtOAc = 5:1 to 3:2.

Compound 9k to 10ka and 10kb

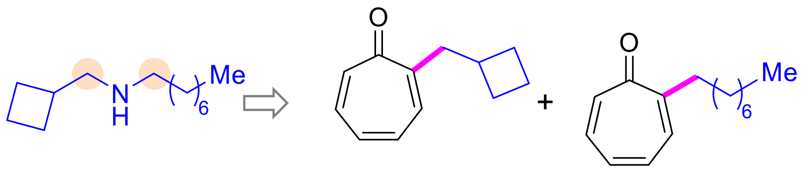

9k **10ka**, 40% **10kb**, 18%

Following the general procedure D for 24 h with tropone and **9k** as starting materials, compound **10ka** was obtained in 40% yield (19.7 mg), while compound **10kb** was obtained in 18% yield (11.1 mg). The elution condition for flash chromatography: PE/EtOAc = 5:1 to 1:1.

Characteristics of 10ka:

Physical State: yellowish powder.

TLC: R_f = 0.62 (PE/EtOAc = 3:2).

^{1}H NMR (600 MHz, $CDCl_3$): δ 7.16 (dd, *J* = 8.7, 1.1 Hz, 1H), 7.11 – 7.02 (m, 2H), 6.97 – 6.86 (m, 2H), 2.74 (d, *J* = 7.4 Hz, 2H), 2.63 (hept, *J* = 7.8 Hz, 1H), 2.09 – 2.01 (m, 2H), 1.89 – 1.80 (m, 2H), 1.74 – 1.66 (m, 2H).

^{13}C NMR (150 MHz, $CDCl_3$): δ 187.4, 154.5, 140.4, 135.4, 134.6, 133.9, 132.5, 42.2, 34.8, 28.5, 18.5.

HRESIMS: m/z 175.1119 $[M + H]^+$ (calcd for $C_{12}H_{15}O^+$, 175.1117).

Characteristics of 10kb:

Physical State: yellowish powder.

TLC: R_f = 0.69 (PE/EtOAc = 3:2).

^{1}H NMR (600 MHz, $CDCl_3$): δ 7.25 – 7.22 (m, 1H), 7.11 – 7.02 (m, 2H), 6.98 – 6.86 (m, 2H), 2.67 – 2.58 (m, 2H), 1.56 – 1.51 (m, 2H), 1.39 – 1.17 (m, 10H), 0.87 (t, *J* = 7.0 Hz, 3H).

^{13}C NMR (150 MHz, $CDCl_3$): δ 187.3, 156.5, 140.5, 135.3, 134.8, 134.0, 132.5, 35.8, 32.0, 29.8, 29.6, 29.4, 29.0, 22.8, 14.2.

HRESIMS: m/z 219.1743 $[M + H]^+$ (calcd for $C_{15}H_{23}O^+$, 219.1743).

Compound 9l to 10b

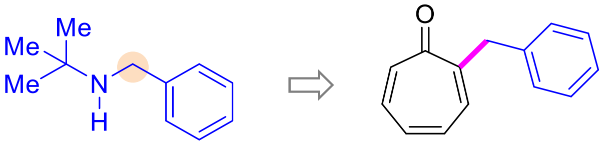

9l **10b**, 55%

Following the general procedure D for 10 h with tropone and **9l** as starting materials, compound **10b** was obtained in 55% yield (30.5 mg). The elution condition for flash chromatography: PE/EtOAc = 5:1 to 3:2.

Compound 9m to 8c

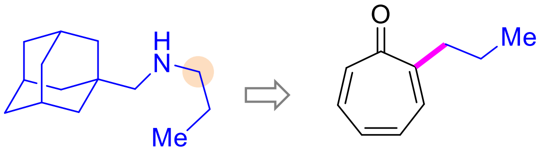

9m **8c**, 55%

Following the general procedure D for 10 h with tropone and **9m** as starting materials, compound **8c** was obtained in 55% yield (23.1 mg). The elution condition for flash chromatography: PE/EtOAc = 5:1 to 3:2.

Compound 9n to 10n

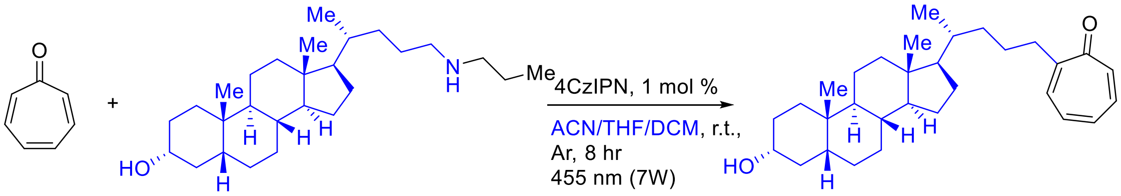

5a **9n** **10n**, 24%(b.r.s.m.)

Following the general procedure D for 8 h with **5a** (78.0mg, 0.744 mmol, 3.0 equiv.) amine **9n** (100.0 mg, 0.247 mmol, 1.0 equiv.) as starting materials, compound **10n** (0.035 mmol, 15.7 mg) was obtained with the yield of 14%, 24% based on recycling **9n** (40.7 mg, 0.101 mmol). Extended the reaction time would lead to dimerization of tropone **5a**. The elution condition for flash chromatography: PE/EtOAc = 5:1 to 1:2.

Physical State: white solid.

TLC: R_f = 0.42 (PE/EtOAc = 1:1).

^{1}H NMR (400 MHz, CDCl$_3$): δ 7.26 – 7.21 (m, 1H), 7.13 – 7.02 (m, 2H), 6.99 – 6.87 (m, 2H), 3.61 (td, *J* = 10.9, 5.4 Hz, 1H), 2.65 (ddd, *J* = 14.3, 9.8, 5.3 Hz, 1H), 2.55 (ddd, *J* = 14.0, 9.3, 5.8 Hz, 1H), 1.99 – 1.92 (m, 1H), 1.87 – 1.70 (m, 4H), 1.58 – 1.31 (m, 14H), 1.25 (d, *J* = 2.4 Hz, 3H), 1.16 – 0.94 (m, 9H), 0.91 (s, 3H), 0.63 (s, 3H).

^{13}C NMR (150 MHz, CDCl$_3$): δ 187.3, 156.5, 140.5, 135.4, 134.8, 134.0, 132.5, 72.1, 56.7, 56.4, 42.9, 42.3, 40.6, 40.4, 36.6, 36.1, 36.1, 36.0, 35.8, 35.5, 34.7, 30.7, 28.4, 27.4, 26.6, 25.6, 24.4, 23.5, 21.0, 18.8, 12.2.

HRESIMS: m/z 451.3572 [M + H]$^+$ (calcd for $C_{31}H_{47}O_2^+$, 451.3571).

Specific Rotation: $[\alpha]^{18}_D$ = +52.8 (*c* 0.03, MeOH).

Alkylation of *Cephalotaxus* Troponoids

Compounds 13 and 14

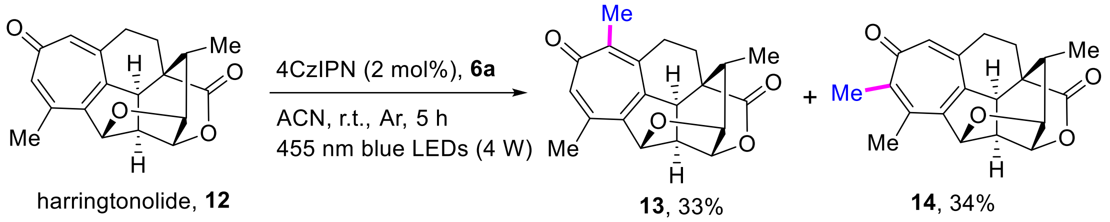

To the harringtonolide **12** (0.026mmol, 1.0 equiv.) in a 5 mL transparent test tube was added photocatalyst 4CzIPN (0.4 mg, 0.00052 mol, 2 mol%) and magnetic stir bars. The tube was sealed with septum, degassed and backfilled with Ar. Add MeCN (0.2 mL) and dimethylamine **6a** (2M in THF, 0.052 mmol, 2.0 equiv.) to the mixture through the syringe. The test tube was placed in the photoreactor and irradiated under 455 nm (4W) LEDs. The mixture was stirred under irradiation for 5 h, before the solvent was removed. After passing through a flash chromatography (PE/EtOAc = 5:1 to 1:1), the title product **13** (2.8 mg, 33%) and **14** (2.9 mg, 34%) was obtained

Characteristics of 13:

Physical State: white solid.

TLC: R_f = 0.44 (PE/EA = 1:1).

^{1}H NMR (600 MHz, CDCl$_3$): δ 6.89 (s, 1H), 5.32 (d, *J* = 5.0 Hz, 1H), 5.20 (t, *J* = 5.3 Hz, 1H), 3.99 (d, *J* = 5.6 Hz, 1H), 3.39 – 3.31 (m, 2H), 3.09 (ddd, *J* = 16.0, 6.1, 1.7 Hz,

1H), 2.85 (ddd, J = 14.7, 7.9, 1.6 Hz, 1H), 2.73 – 2.60 (m, 1H), 2.34 (d, J = 1.1 Hz, 3H), 2.28 (d, J = 1.3 Hz, 3H), 1.84 (q, J = 7.6 Hz, 1H), 1.35 – 1.27 (m, 1H), 0.90 (d, J = 7.6 Hz, 3H).

^{13}C NMR (125 MHz, CDCl$_3$): δ 187.8, 174.1, 147.7, 145.5, 143.3, 142.2, 141.7, 138.3, 86.0, 80.3, 79.8, 50.7, 46.1, 42.0, 40.4, 28.9, 23.0, 22.6, 17.0, 14.9.

HRESIMS: m/z 325.1434 [M + H]$^+$ (calcd for $C_{20}H_{21}O_4^+$, 325.1434).

Specific Rotation: $[\alpha]^{18}_{D}$ = +3 (c 0.08, MeOH).

Characteristics of 14:

Physical State: white solid.

TLC: R_f = 0.47 (PE/EA = 1:1).

^{1}H NMR (600 MHz, CDCl$_3$): δ 6.83 (d, J = 1.9 Hz, 1H), 5.44 (d, J = 4.9 Hz, 1H), 5.20 (t, J = 5.3 Hz, 1H), 3.98 (d, J = 5.6 Hz, 1H), 3.36 (t, J = 4.0 Hz, 2H), 2.91 – 2.75 (m, 2H), 2.63 (dd, J = 14.6, 6.3 Hz, 1H), 2.38 (s, 3H), 2.29 (s, 3H), 1.77 (q, J = 7.5 Hz, 1H), 1.33 – 1.28 (m, 1H), 0.89 (d, J = 7.6 Hz, 3H).

^{13}C NMR (125 MHz, CDCl$_3$): δ 187.8, 173.9, 148.9, 145.6, 143.1, 143.1, 140.6, 135.9, 87.3, 80.3, 79.7, 49.8, 46.2, 41.9, 40.1, 31.6, 22.6, 20.8, 18.6, 15.0.

HRESIMS: m/z 325.1433 [M + H]$^+$ (calcd for $C_{20}H_{21}O_4^+$, 325.1434).

Specific Rotation: $[\alpha]^{18}_{D}$ = +12 (c 0.03, MeOH).

Compounds 3 and 4

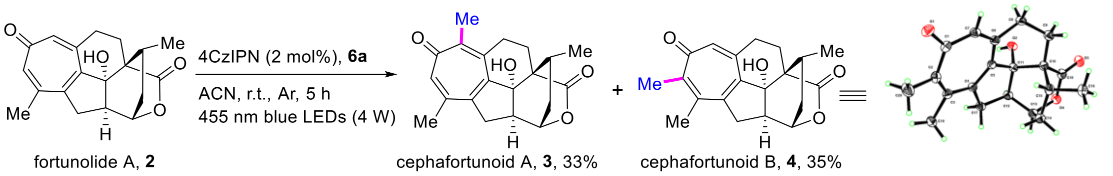

Following the synthesis method of compounds **13** and **14** for 5 h with **2** (0.094 mmol) as starting material, compound **3** was obtained in 33% yield (2.8 mg), while compound **4** was obtained in 35% yield (3.0 mg). All the spectrum data of **3** and **4** were paralleled to those isolated natural product cephafortunoids A and B.[16]

Characteristics of 3:

Physical State: white solid.

TLC: R_f = 0.33 (PE/EA = 1:1).

^{1}H NMR (800 MHz, CDCl$_3$): δ 6.78 (d, *J* = 1.3 Hz, 1H), 4.75 (td, *J* = 4.5, 1.3 Hz, 1H), 3.26 (dd, *J* = 18.5, 9.6 Hz, 1H), 2.97 (dd, *J* = 18.7, 8.5 Hz, 1H), 2.91 (s, 1H), 2.78 (dddd, *J* = 9.8, 5.0, 3.4, 1.7 Hz, 1H), 2.72 – 2.66 (m, 1H), 2.53 (dd, *J* = 18.6, 3.5 Hz, 1H), 2.46 (dt, *J* = 18.3, 8.9 Hz, 1H), 2.19 (d, *J* = 1.1 Hz, 3H), 2.15 (s, 3H), 2.07 – 2.03 (m, 1H), 2.03 – 1.99 (m, 1H), 1.79 (dqd, *J* = 14.1, 7.0, 5.1 Hz, 1H), 1.52 (dtd, *J* = 14.8, 4.7, 1.8 Hz, 1H), 0.99 (d, *J* = 7.0 Hz, 3H).

^{13}C NMR (125 MHz, CDCl$_3$): δ 187.9, 174.0, 147.0, 143.1, 142.4, 142.1, 140.6, 137.7, 87.5, 77.3, 49.5, 45.7, 35.2, 29.2, 28.7, 26.3, 23.8, 19.3, 18.4, 17.2.

HRESIMS: m/z 327.1591 $[M + H]^+$ (calcd for $C_{20}H_{23}O_4$, 327.1591).

Specific Rotation: $[\alpha]^{18}_D$ = +9 (*c* 0.085, MeOH); lit. report: $[\alpha]^{20}_D$ = +73 (*c* 0.36, MeOH).[16]

Characteristics of 4:

Physical State: colorless crystal.

Melting Point: 264–267 °C.

TLC: R_f = 0.35 (PE/EA = 1:1).

^{1}H NMR (500 MHz, CDCl$_3$): δ 6.77 (t, *J* = 1.3 Hz, 1H), 4.75 (td, *J* = 4.6, 1.2 Hz, 1H), 3.36 (dd, *J* = 18.5, 9.5 Hz, 1H), 3.07 (dddd, *J* = 17.5, 9.3, 3.8, 1.6 Hz, 1H), 2.83 – 2.74 (m, 2H), 2.70 – 2.60 (m, 2H), 2.60 – 2.52 (m, 1H), 2.24 (s, 6H), 1.97 – 1.92 (m, 1H), 1.92 – 1.88 (m, 1H), 1.66 – 1.61 (m, 1H), 1.52 – 1.46 (m, 1H), 0.95 (d, *J* = 7.0 Hz, 3H).

^{13}C NMR (125 MHz, CDCl$_3$): δ 187.3, 173.9, 148.6, 145.3, 143.3, 140.6, 140.5, 136.4, 87.3, 77.3, 50.0, 45.7, 37.2, 29.7, 29.0, 27.4, 21.2, 19.4, 18.8, 18.1.

HRESIMS: m/z 327.1592 $[M + H]^+$ (calcd for $C_{20}H_{23}O_4^+$, 327.1591).

Specific Rotation: $[\alpha]^{18}_D$ = +18 (*c* 0.02, MeOH); lit. report: $[\alpha]^{20}_D$ = +27 (*c* 0.38, MeOH).[16]

Mechanistic Studies

Radical capture experiment

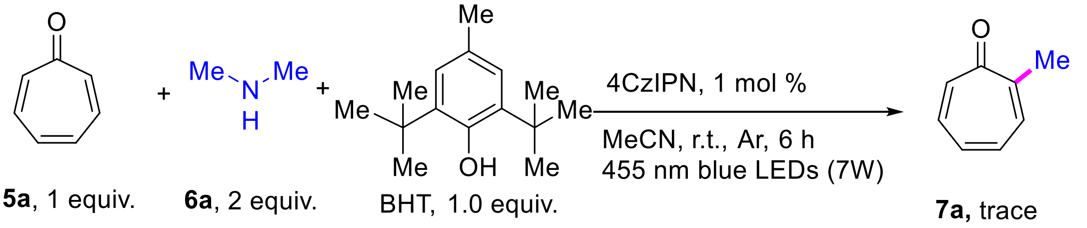

To the tropone **5a** (30.0mg, 0.283 mmol, 1.0 equiv.) in a 5 mL transparent test tube was added photocatalyst 4CzIPN (2.2 mg, 0.00283 mmol, 1 mol%), BHT (62.3 mg, 0.283 mmol, 1.0 equiv.) and magnetic stir bars. The tube was sealed with septum, degassed and backfilled with Ar. Add MeCN (2.2 mL) and dimethylamine (2M in THF, 0.566 mmol, 2.0 equiv.) to the mixture through the syringe. The test tube was placed in the photoreactor and irradiated under 455 nm (7W) LEDs for 6 h. The reaction was hampered. Only trace **7a** was found, and none of the BHT adducts was found.

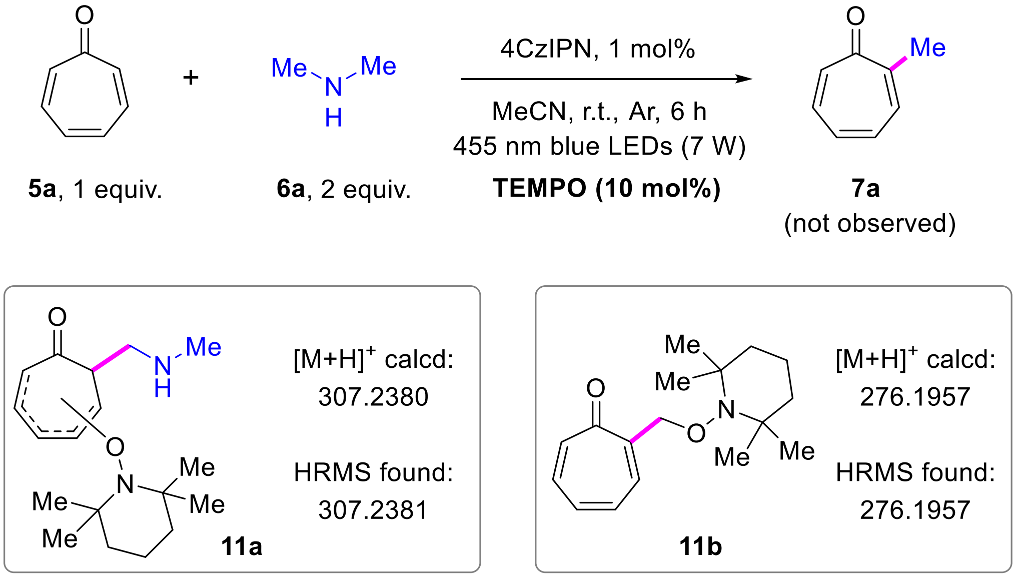

To the tropone **5a** (30.0mg, 0.283 mmol, 1.0 equiv.) in a 5 mL transparent test tube was added photocatalyst 4CzIPN (2.2 mg, 0.00283 mmol, 1 mol%), TEMPO (4.4 mg, 0.0283 mmol, 10% mol) and magnetic stir bars. The tube was sealed with septum, degassed and backfilled with Ar. Add MeCN (2.2 mL) and dimethylamine (2M in THF, 0.566 mmol, 2.0 equiv.) to the mixture through the syringe. The test tube was placed in

the photoreactor and irradiated under 455 nm (7W) LEDs for 6 h. The reaction was completely suppressed by TEMPO, resulting in the absence of **7a**. Subsequent HRMS analysis (Figure S1) revealed the presence of **11a** and **11b**, which were adducts formed between TEMPO/tropone/amine.

(a)

**Data Filename**	ESIH202404684.d	**Sample Name**	D4-ZQT22E-2
**Sample ID**		**Position**	P1-A4
**Instrument Name**	Agilent 6520 Q-TOF	**Acq Method**	20160322_MS_ESIH_POS_1min.m
**Acquired Time**	9/25/2024 2:29:52 PM	**DA Method**	ESI-HR-20231114.m
**Comment**	ESIH by fangsu		

User Spectra

Fragmentor Voltage	Collision Energy	Ionization Mode
170	0	ESI

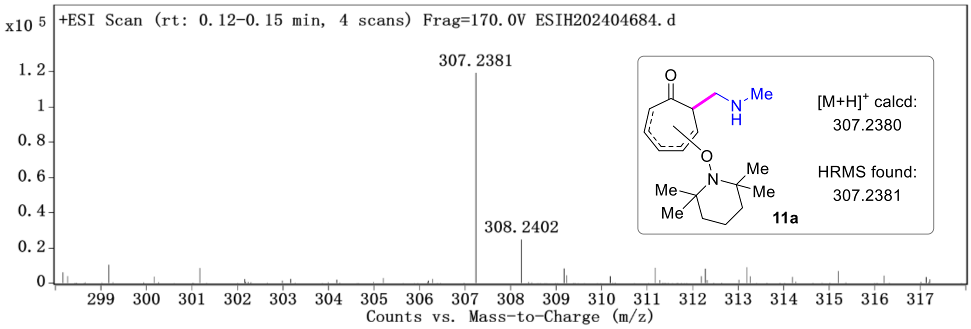

Formula Calculator Results

m/z	Calc m/z	Diff (mDa)	Diff (ppm)	Ion Formula	Ion
307.2381	307.238	-0.07	-0.23	C18 H31 N2 O2	(M+H)+

--- End Of Report ---

(b)

**Data Filename**	ESIH202404684.d	**Sample Name**	D4-ZQT22E-2
**Sample ID**		**Position**	P1-A4
**Instrument Name**	Agilent 6520 Q-TOF	**Acq Method**	20160322_MS_ESIH_POS_1min.m
**Acquired Time**	9/25/2024 2:29:52 PM	**DA Method**	ESI-HR-20231114.m
**Comment**	ESIH by fangsu		

User Spectra

Fragmentor Voltage	Collision Energy	Ionization Mode
170	0	ESI

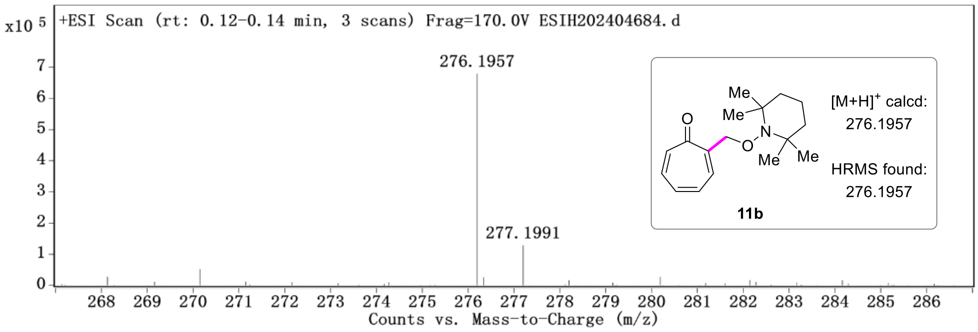

Formula Calculator Results

m/z	Calc m/z	Diff (mDa)	Diff (ppm)	Ion Formula	Ion
276.1957	276.1958	0.15	0.53	C17 H26 N O2	(M+H)+

--- End Of Report ---

Figure S1 The HRMS analysis. (a) The HRMS spectrum of **11a**; (b) The HRMS spectrum of **11b**.

Light on and off experiment

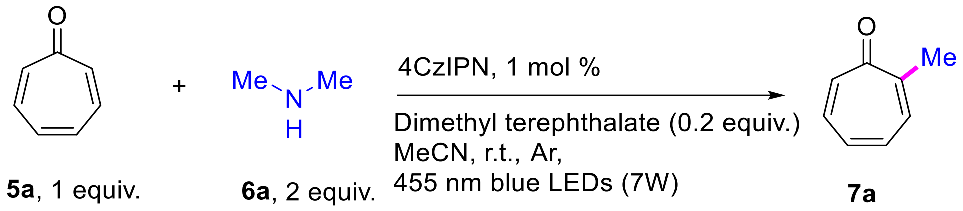

To the tropone **5a** (30.0mg, 0.283 mmol, 1.0 equiv.) in a 5 mL transparent test tube was added photocatalyst 4CzIPN (2.2 mg, 0.00283 mmol, 1 mol%), internal standard dimethyl terephthalate (11.0 mg, 0.0566 mmol, 0.2 equiv) and magnetic stir bars. The tube was sealed with septum, degassed and backfilled with Ar. Add MeCN (2.2 mL) and dimethylamine (2M in THF, 0.566 mmol, 2.0 equiv.) to the mixture through the syringe. The test tube was placed in the photoreactor and irradiated under 455 nm (7W) LEDs for 2 hours, followed by a 2-hour dark period. After each of these intervals, withdraw 0.08 mL of the reaction mixture using a syringe for NMR analysis. Repeat this light-on/off cycle for three times, resulting in six intervals of analysis over a total of 12 hours (Figure S2). For each interval, determine the yield by using NMR data with internal standard to assess the reaction's progress and the impact of light exposure on product formation.

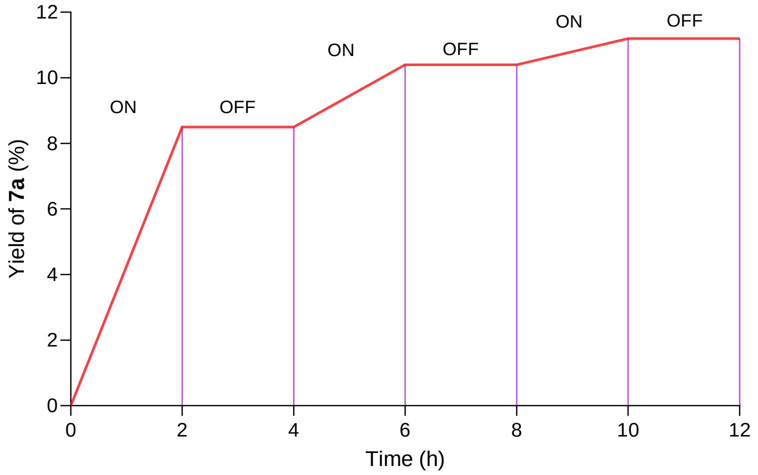

Figure S2 Light on and off experiment plot of yield versus each period of time.

Luminescence quenching studies

Luminescence quenching studies were performed using a Techcomp FL970 Spectrophotometer. In each experiment, the photocatalyst and varying concentrations of quenchers (tropone **5a** and dimethylamine **6a**) were dissolved in CH_3CN in screw-top 1.0 cm quartz cuvettes and degassed by sparging with argon for 20 minutes.

For the emission quenching of 4CzIPN, the photocatalyst concentration was 1.25 μM, the solution was irradiated at 455 nm and the fluorescence was observed at around 544nm. Plots were constructed according to the Stern–Volmer equation $I_0/I = 1 + k_q\tau_0[Q]$.

As shown in Figure S3, Stern-Volmer quenching studies demonstrated that both tropone **5a** and dimethylamine **6a** effectively quenched the excited state of photocatalyst 4CzIPN*, albeit through distinct mechanisms. For tropone **5a**, the quenching aligned with its inherent extended conjugated structures and was attributed to energy transfer (EnT) with excited-state 4CzIPN*. This EnT pathway is further corroborated by the facile cycloaddition of tropone **5a** observed in the absence of amine—a reaction requiring energy input from the photocatalyst. However, introducing a secondary amine suppresses this cycloaddition, shifting the reaction toward the desired alkylation. Additionally, the quenching observed with dimethylamine **6a** suggests a single-electron transfer (SET) mechanism between the excited state of $4CzIPN^*$ and dimethylamine **6a**.

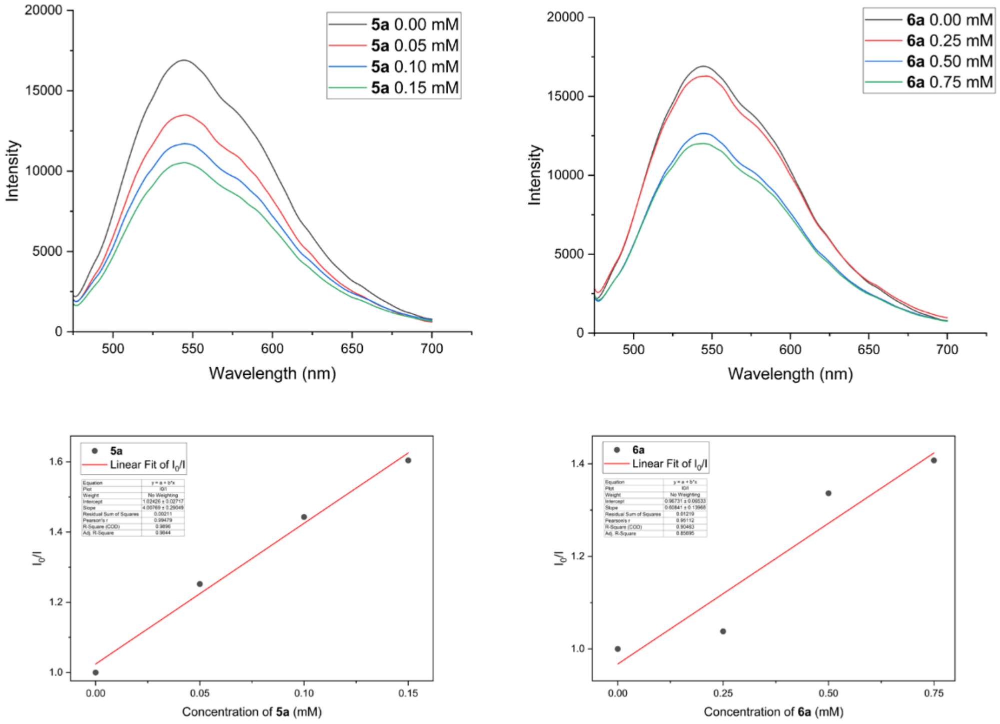

Figure S3 Luminescence quenching of 4CzIPN by **5a** or **6a** and Stern-Volmer plots.

Quantum yield measurement

Part 1: Photon Flux Detection

A ferrioxalate actinometer solution was prepared by following the Hammond variation of the Hatchard and Parker procedure outlined in *Handbook of Photochemistry*.[17] The ferrioxalate actinometer solution measures the decomposition of ferric ions to ferrous ions, which are complexed by 1,10-phenanthroline and monitored by UV/Vis absorbance at 510 nm. The moles of iron-phenanthroline complex formed are related to moles of photons absorbed. The solutions were prepared and stored in a dark laboratory according to the procedure described previously[18].

Procedure: 1 mL of the potassium ferrioxalate solution, whose quantum yield is being measured, were added to a standard 25 mL Schlenk tube bought from Synthware. The tube was placed in parallel photoreactor irradiated without stirring by blue light (455 nm) who worked at 2000 mW. This procedure was repeated twice, quenching the reactions after different time intervals: 0, 5, 10, 15 and 20 seconds. The UV-Vis

spectrum was shown in Figure S4. Based on the data, we got the correlation (Figure S5) between the moles of products and time.

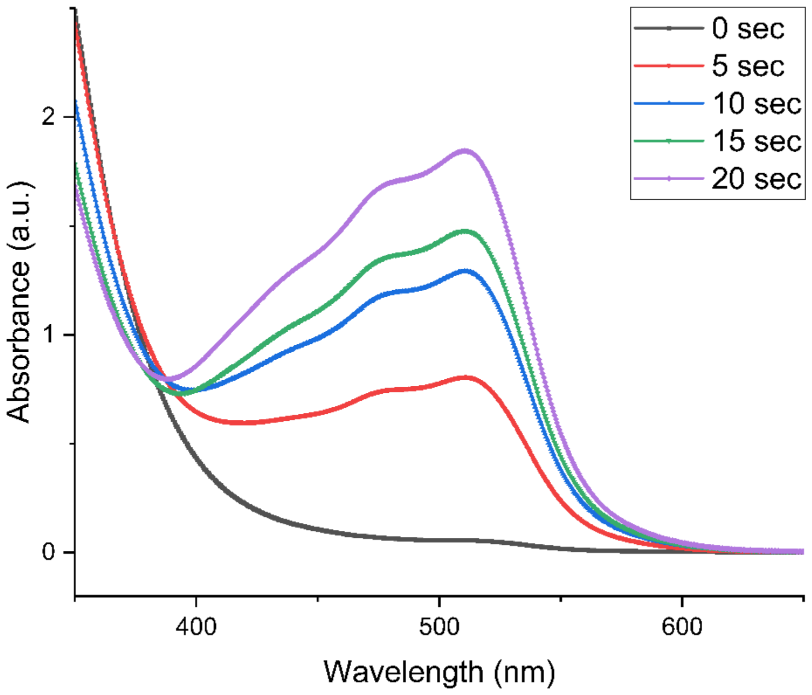

Figure S4 The UV-Vis Spectrum.

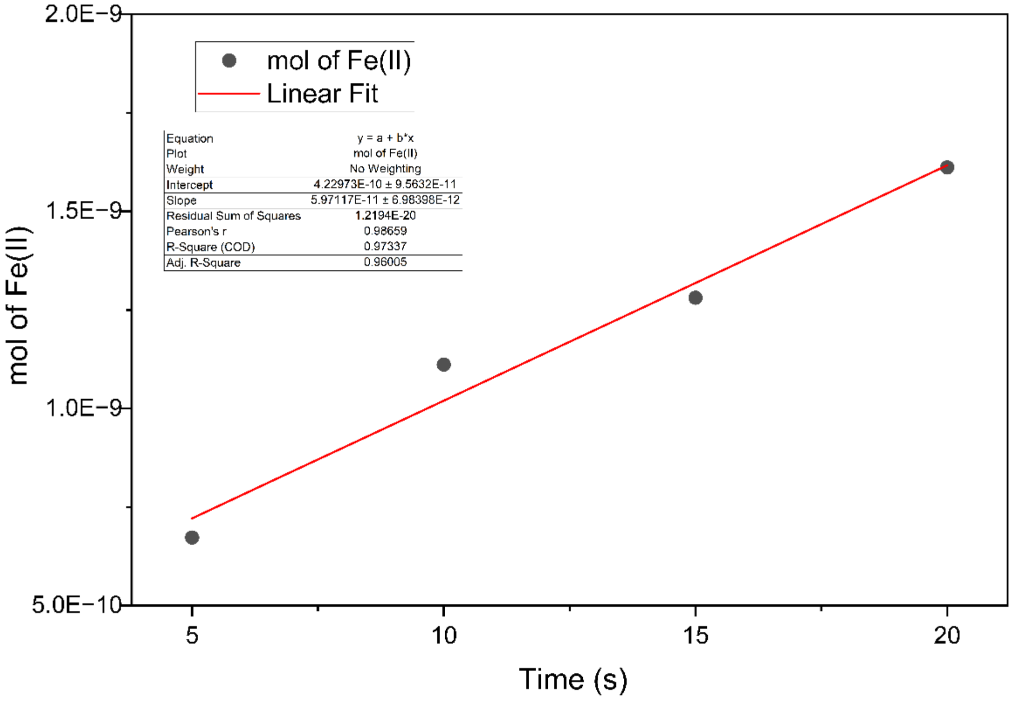

Figure S5 The correlation of mol of Fe(II) with time.

The actinometer measurements were done as follows: 1) After irradiation, the actinometer solution was removed and placed in a 10 mL volumetric flask containing 0.5 mL of 1,10-phenanthroline solution and 2 mL of buffer solution. This flask was filled to the mark with water. 2) The UV-Vis spectra of the complexed actinometer samples were recorded for each time interval. The absorbance of the complexed actinometer solution was monitored at 510 nm. The moles of Fe^{2+} formed for each sample are determined according to the Beer's Law (eq. S1):

$$moles\ (Fe^{2+}) = \frac{V_1 \times V_2 \times \Delta A(510\text{ nm})}{10^3 \times V_2 \times l \times \varepsilon(510\text{ nm})} \qquad \text{(eq. S1)}$$

where V_1 is the irradiated volume (1 mL), V_2 is the aliquot of the irradiated solution taken for the determination of the ferrous ions (1 mL), V_3 is the final volume after complexation with phenanthroline (10 mL), l is the optical path-length of the irradiation cell (1 cm), ΔA (510 nm) the optical difference in absorbance between the irradiated solution and the one stored in the dark, ε (510 nm) is that of the complex $Fe(phen)_3^{2+}$ (11100 $L.mol^{-1}.cm^{-1}$).

The moles of Fe^{2+} formed (x) are plotted as a function of time (t). The slope of this line dx/dt was correlated to the moles of incident photons by unit of time, ($q^0_{n,p}$) was determined by the use of the following Equation S2:

$$\Phi = \frac{dx/dt}{q^0_{n,p}[1-10^{-A(\lambda)}]} \qquad \text{(eq. S2)}$$

where dx/dt is the rate of change of a measurable quantity (spectral or any other property), the quantum yield (Φ) for Fe^{2+} at 455 nm is 0.9, and A (λ) is the absorbance of the actinometer at the wavelength used to carry out the experiments (455 nm). The absorbance at 455 nm A(455) was measure using a Shimadzu UV-2700 UV-Vis spectrophotometer in 1 mm path quartz cuvettes in the presence of the bandpass filter of 455 nm employed to run the measurements, obtaining an absorbance of 0.096. $q^0_{n,p}$,which is the photon flux, was determined to be 3.3×10^{-10} einstein s^{-1}.

Part 2: Determination of the reaction quantum yield

The quantum yield is defined as:

$$\Phi = \frac{\text{Moles of Product Formed}}{\text{Photons Absorbed by Sample}} \qquad \text{(eq. S3)}$$

If the transmittance of photons at blue LEDs (455 nm 2000 mW) is sufficiently small, it can be assumed that all of the photons which pass through the cell are absorbed. The above equation may be then written as:

$$\Phi = \frac{\text{Moles of Product Formed}}{\text{Photon Flux}\times\text{Time}} = \frac{\text{Moles of Product Formed}}{\text{Photons}} \qquad \text{(eq. S4)}$$

The mol of products was determined by GC-MS with dimethyl terephthalate as internal standard. Mol of photons were calculated by Photon Flux×Time. The correlation of mol of **7a** and mol of photons was shown in Figure S6, where the slope gives the quantum yield Φ = 0.078 of this developed reaction. This outcome significantly diminishes the likelihood of radical chain reactions.

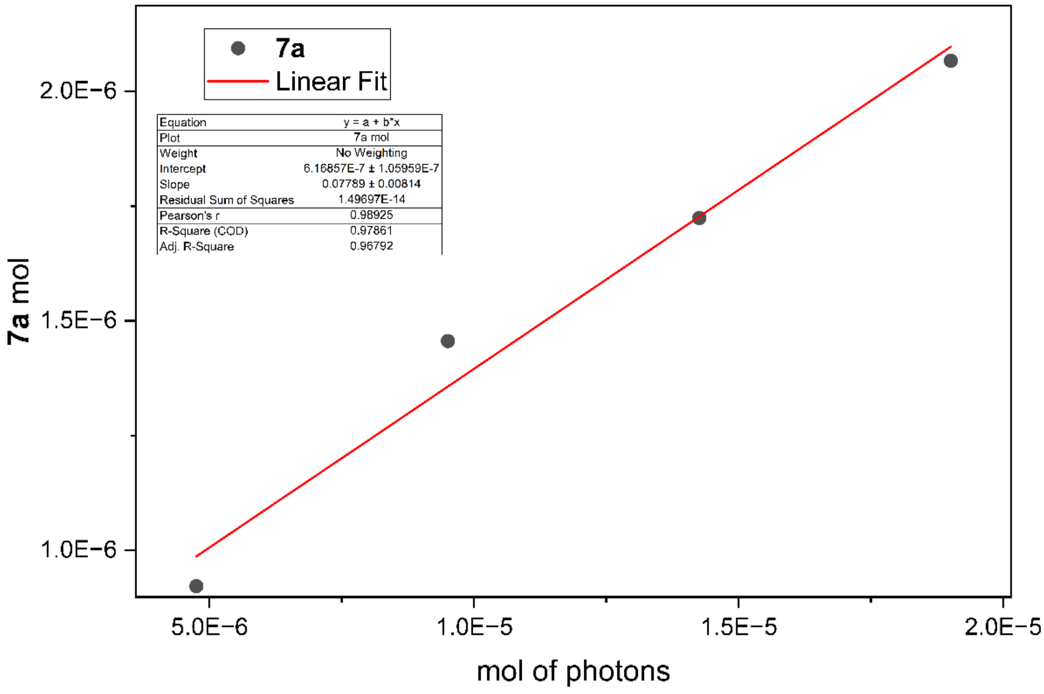

Figure S6 The quantum yield (Φ) of the developed reaction.

NMR/MS Spectra

Compound 5c ^{1}H NMR (400 MHz, $CDCl_3$)

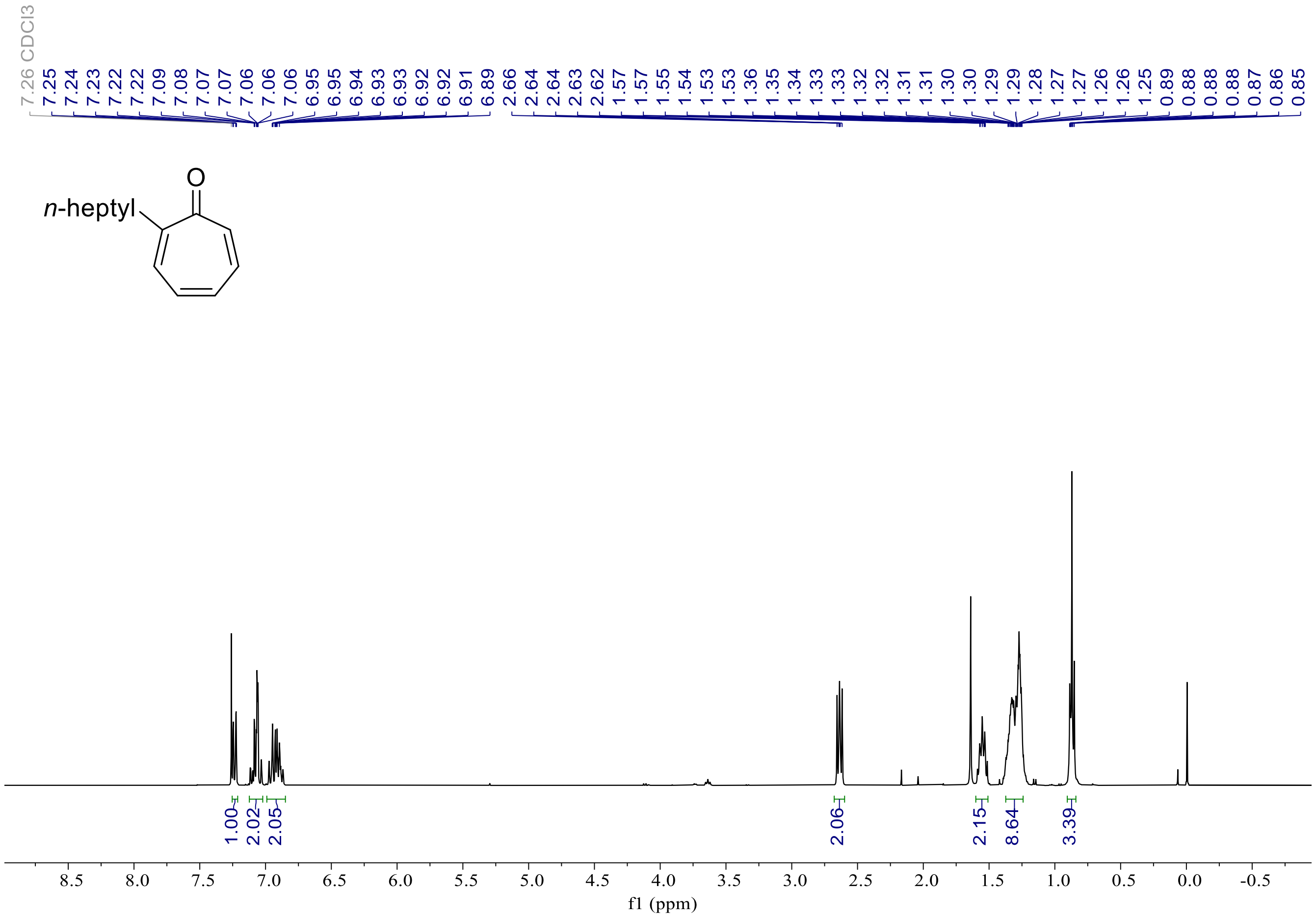

Compound 5c ^{13}C NMR (100 MHz, $CDCl_3$)

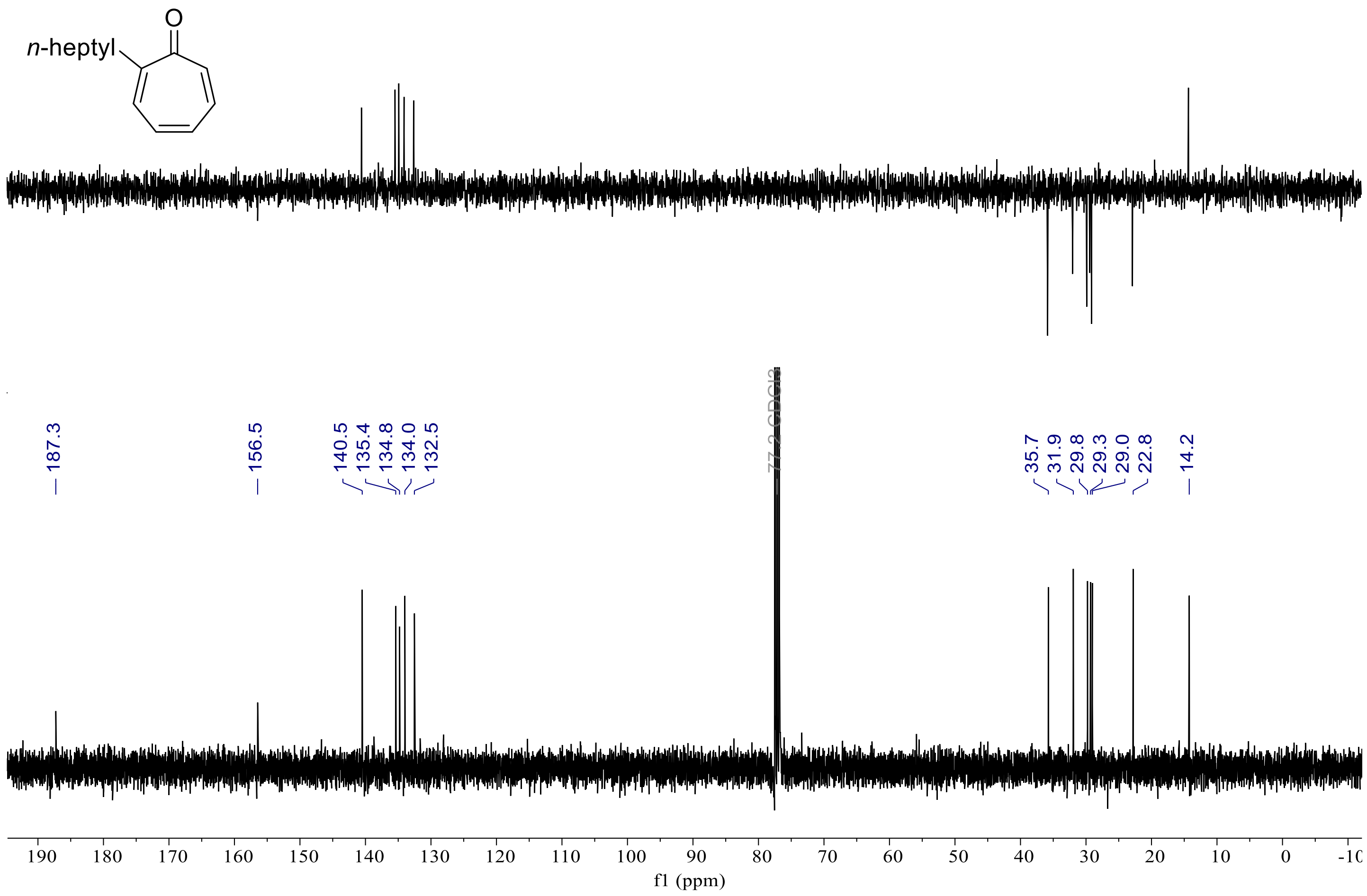

Compound 5c HRMS (ESI-TOF)

**Data Filename**	ESIH202502114-1.d	**Sample Name**	D4-ZQT18-26
**Sample ID**		**Position**	P1-A1
**Instrument Name**	Agilent 6520 Q-TOF	**Acq Method**	20160322_MS_ESIH_POS_1min.m
**Acquired Time**	4/2/2025 2:40:30 PM	**DA Method**	ESI-HR-20231114.m
**Comment**	ESIH by fangsu		

User Spectra

Fragmentor Voltage	Collision Energy	Ionization Mode
170	0	ESI

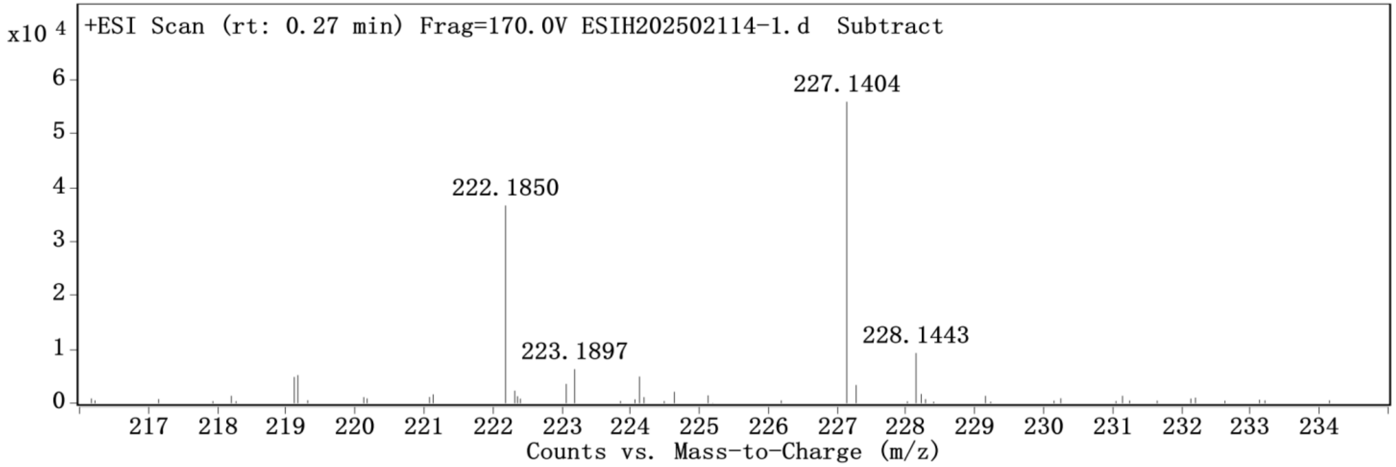

Formula Calculator Results

m/z	Calc m/z	Diff (mDa)	Diff (ppm)	Ion Formula	Ion
227.1404	227.1406	0.21	0.94	C14 H20 Na O	(M+Na)+
222.185	222.1852	0.2	0.91	C14 H24 N O	(M+NH4)+

--- End Of Report ---

Compound 5f ¹H NMR (600 MHz, $CDCl_3$)

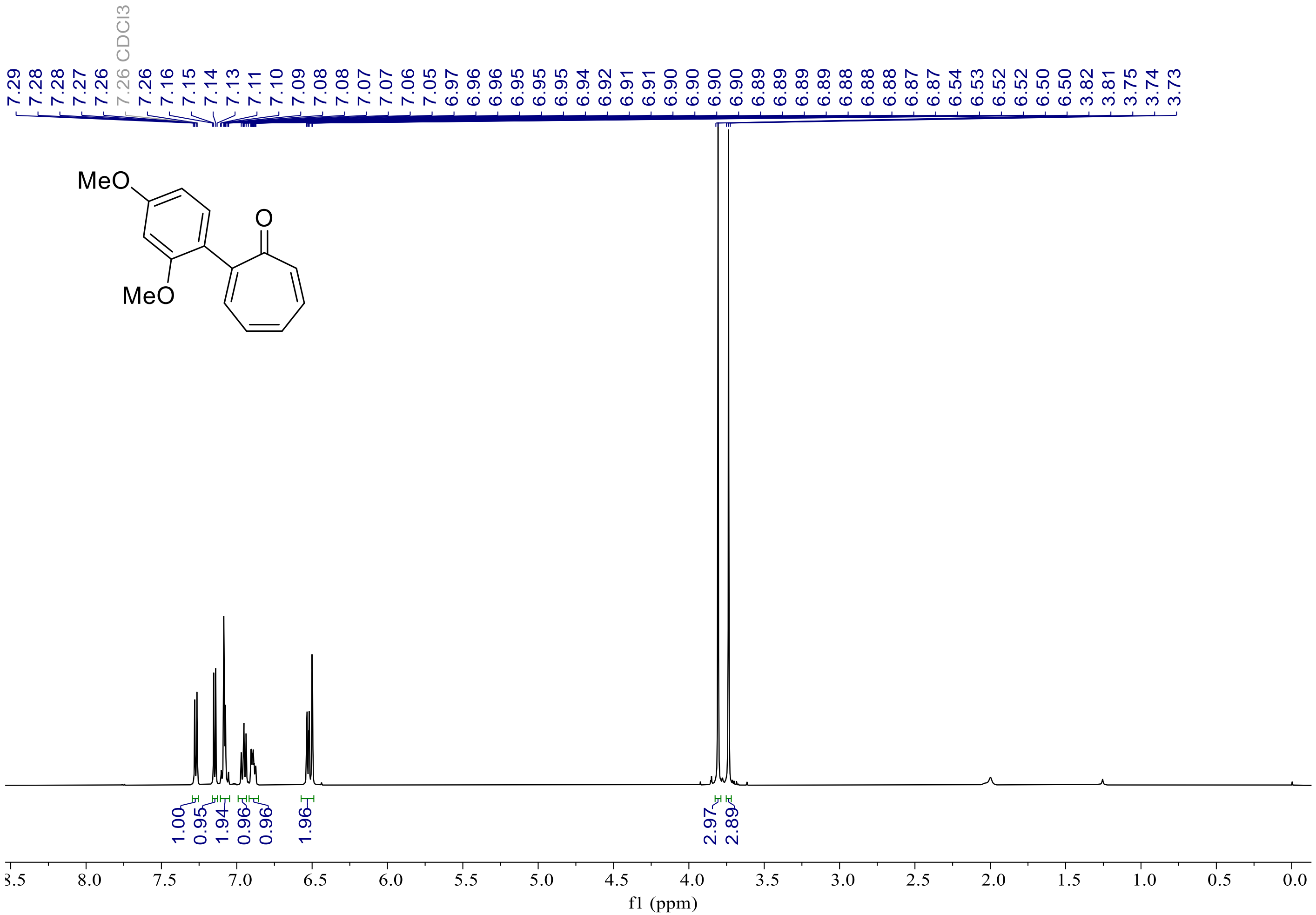

Compound 5f ^{13}C NMR (150 MHz, $CDCl_3$)

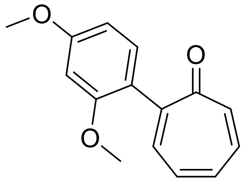

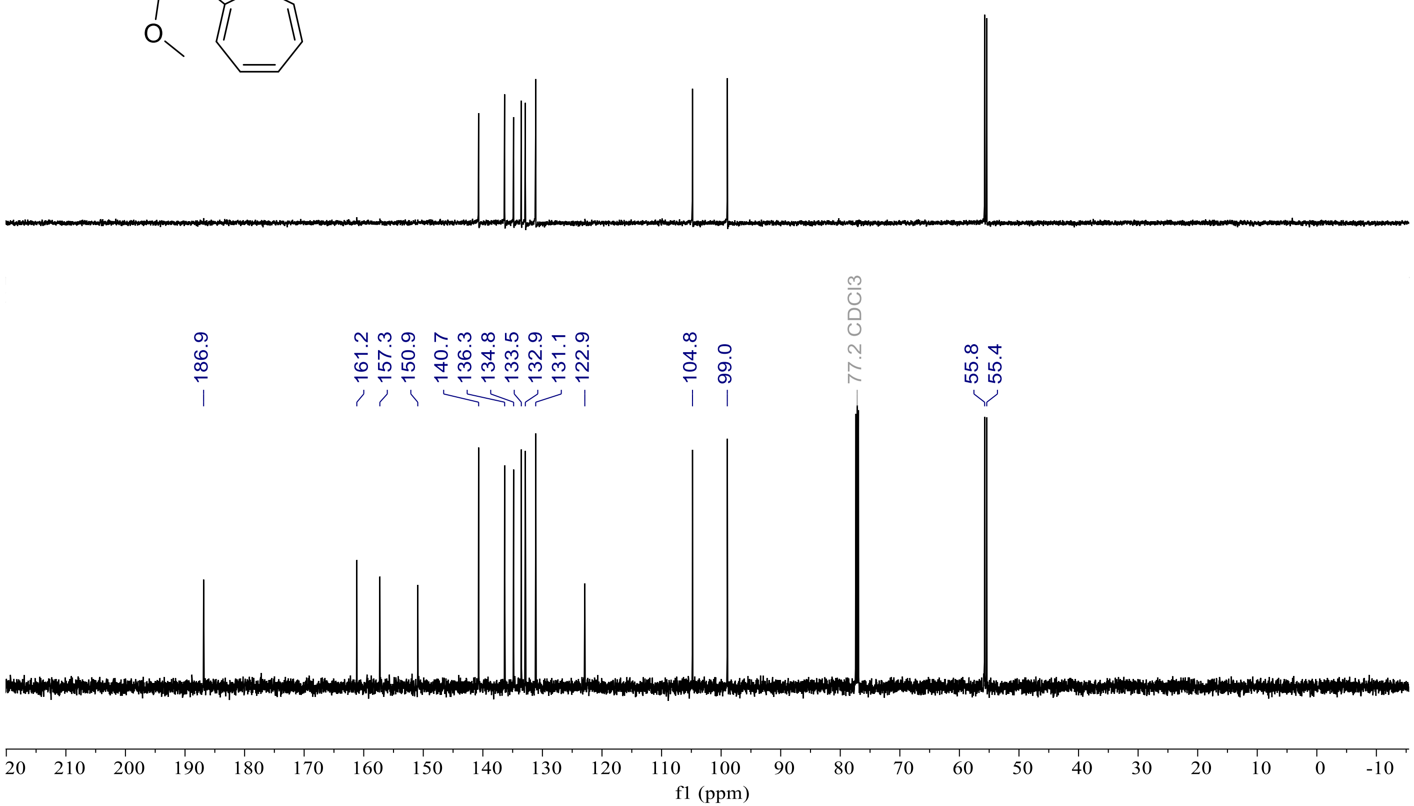

Compound 5f HRMS (ESI-TOF)

**Data Filename**	ESIH202405592.d	**Sample Name**	D4-D4-ZQT18-30
**Sample ID**		**Position**	P1-A2
**Instrument Name**	Agilent 6520 Q-TOF	**Acq Method**	20160322_MS_ESIH_POS_1min.m
**Acquired Time**	11/28/2024 2:09:58 PM	**DA Method**	ESI-HR-20231114.m
**Comment**	ESIH by fangsu		

User Spectra

Fragmentor Voltage	Collision Energy	Ionization Mode
170	0	ESI

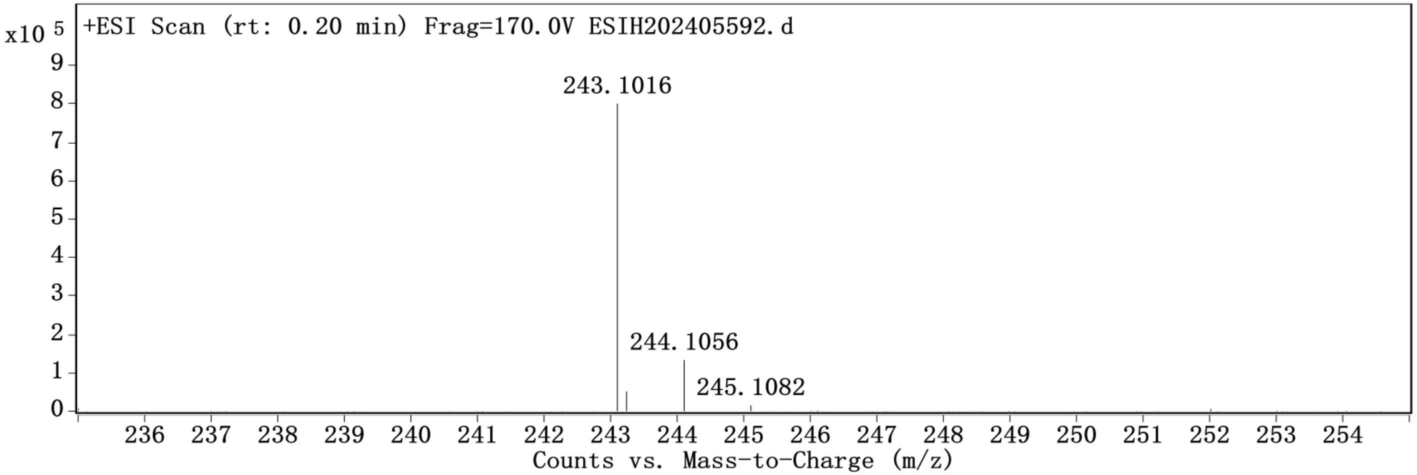

Formula Calculator Results

m/z	Calc m/z	Diff (mDa)	Diff (ppm)	Ion Formula	Ion
243.1016	243.1016	-0.08	-0.33	C15 H15 O3	(M+H)+

--- End Of Report ---

Compound 5g ^{1}H NMR (400 MHz, $CDCl_3$)

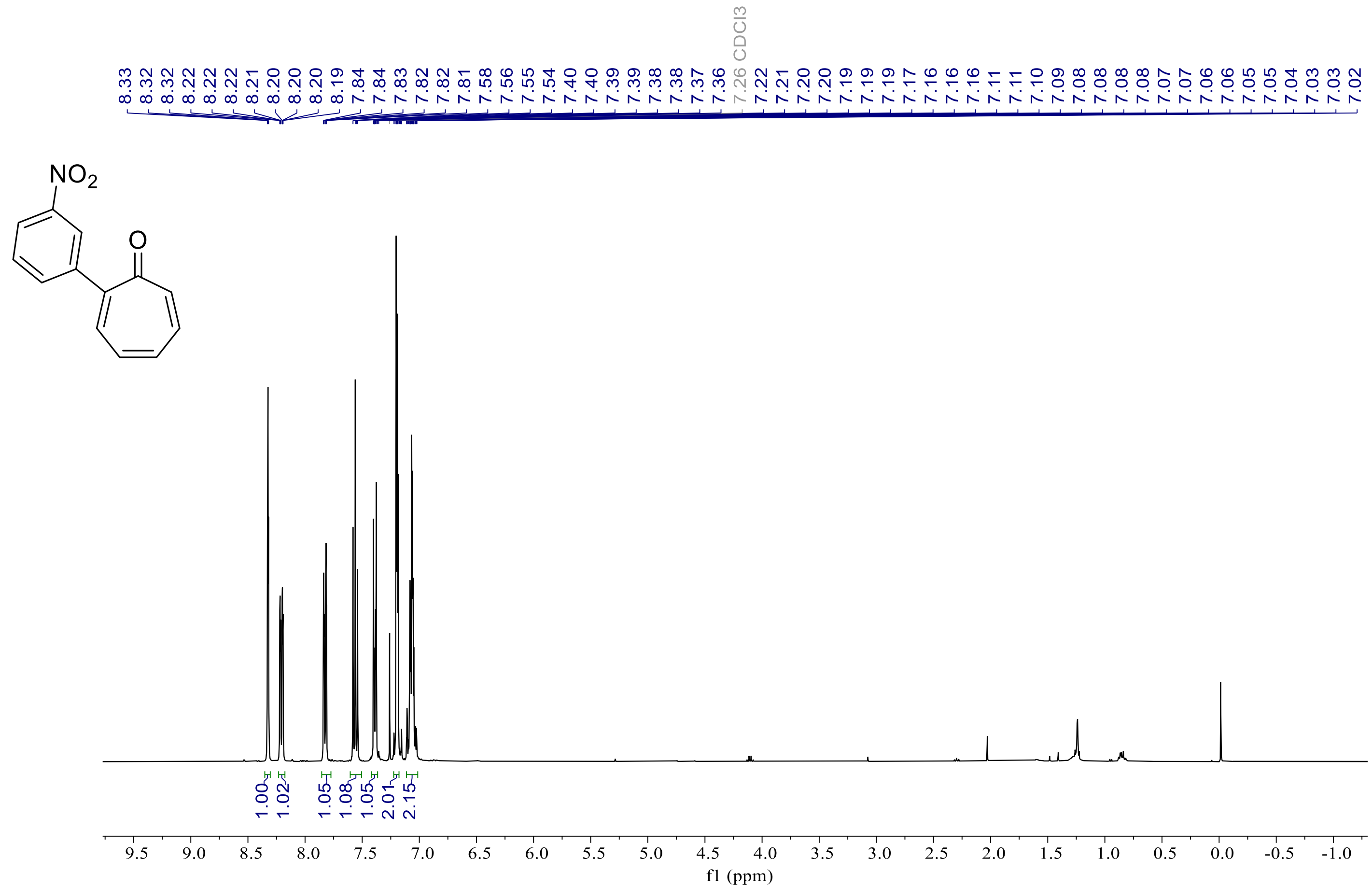

Compound 5g ^{13}C NMR (100 MHz, $CDCl_3$)

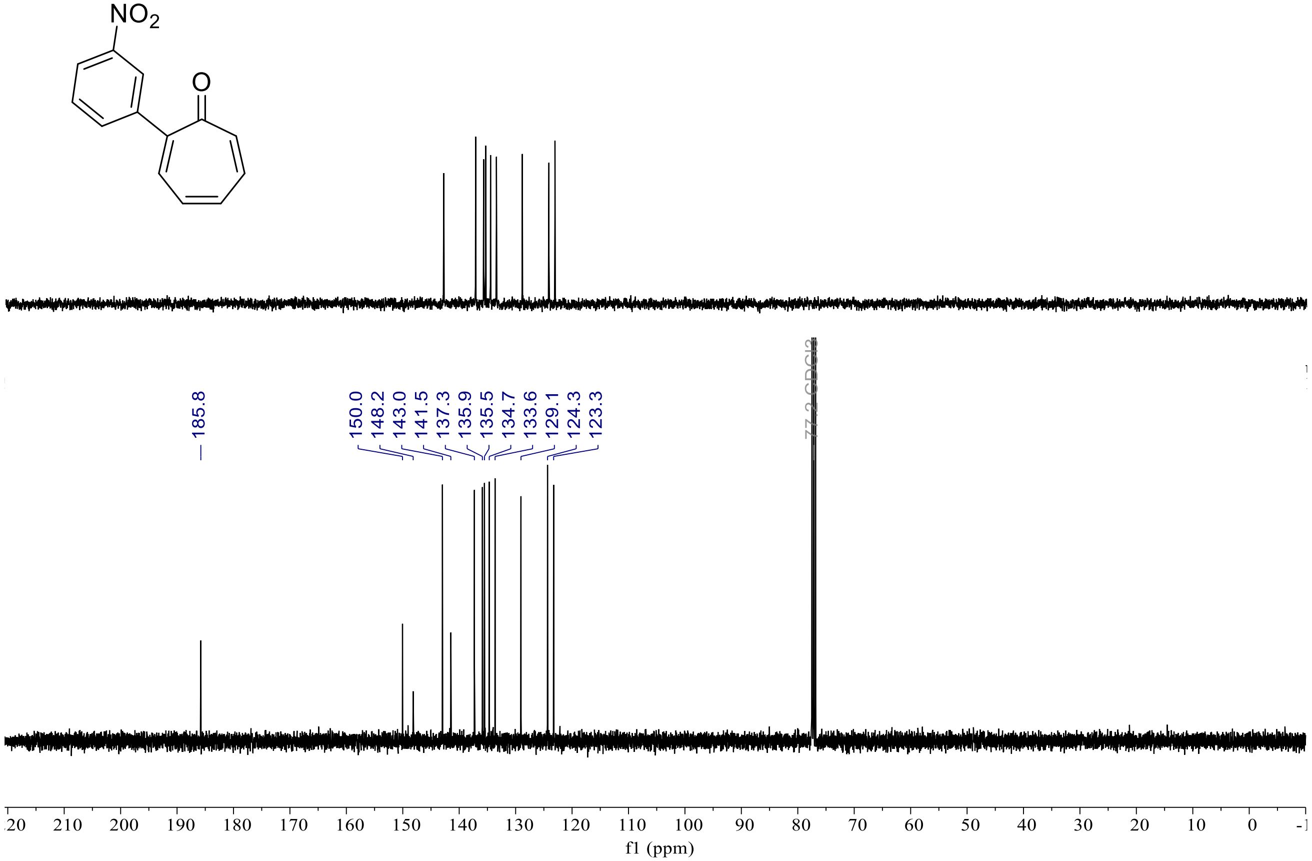

Compound 5g HRMS (ESI-TOF)

**Data Filename**	ESIH202501851.d	**Sample Name**	D4-ZQriv6
**Sample ID**		**Position**	P1-B5
**Instrument Name**	Agilent 6520 Q-TOF	**Acq Method**	20160322_MS_ESIH_POS_1min.m
**Acquired Time**	3/18/2025 2:42:24 PM	**DA Method**	ESI-HR-20231114.m
**Comment**	ESIH by fangsu		

User Spectra

Fragmentor Voltage	Collision Energy	Ionization Mode
170	0	ESI

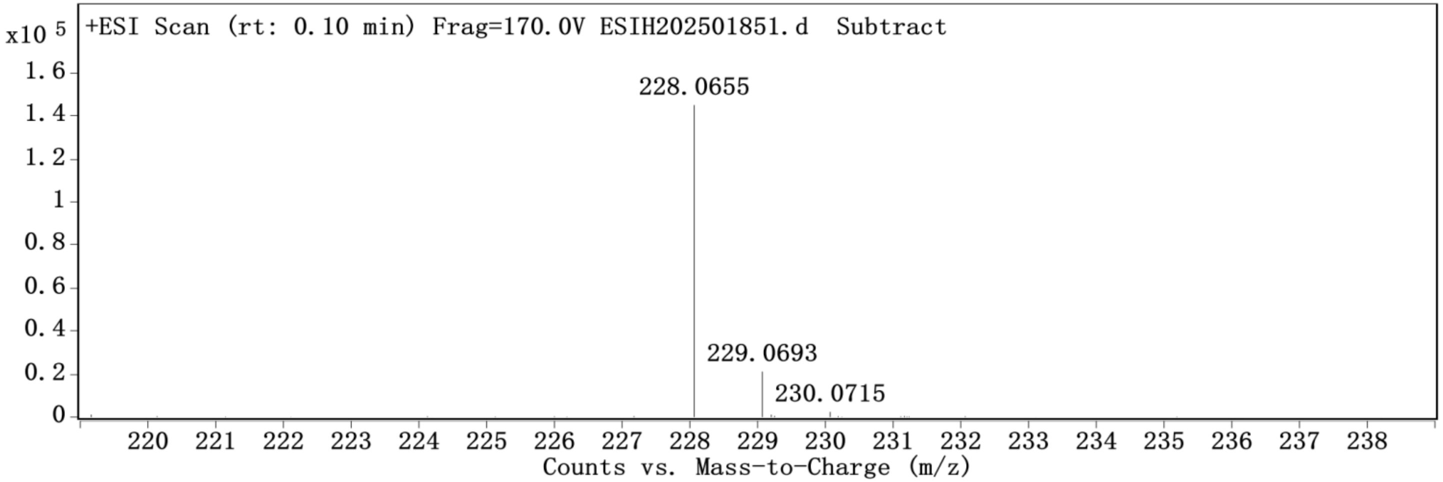

Formula Calculator Results

m/z	Calc m/z	Diff (mDa)	Diff (ppm)	Ion Formula	Ion
228.0655	228.0655	-0.02	-0.09	C13 H10 N O3	(M+H)+

--- End Of Report ---

Compound 5h ¹H NMR (400 MHz, CDCl₃)

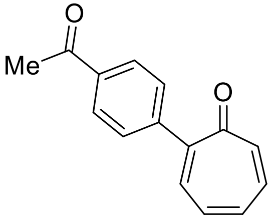

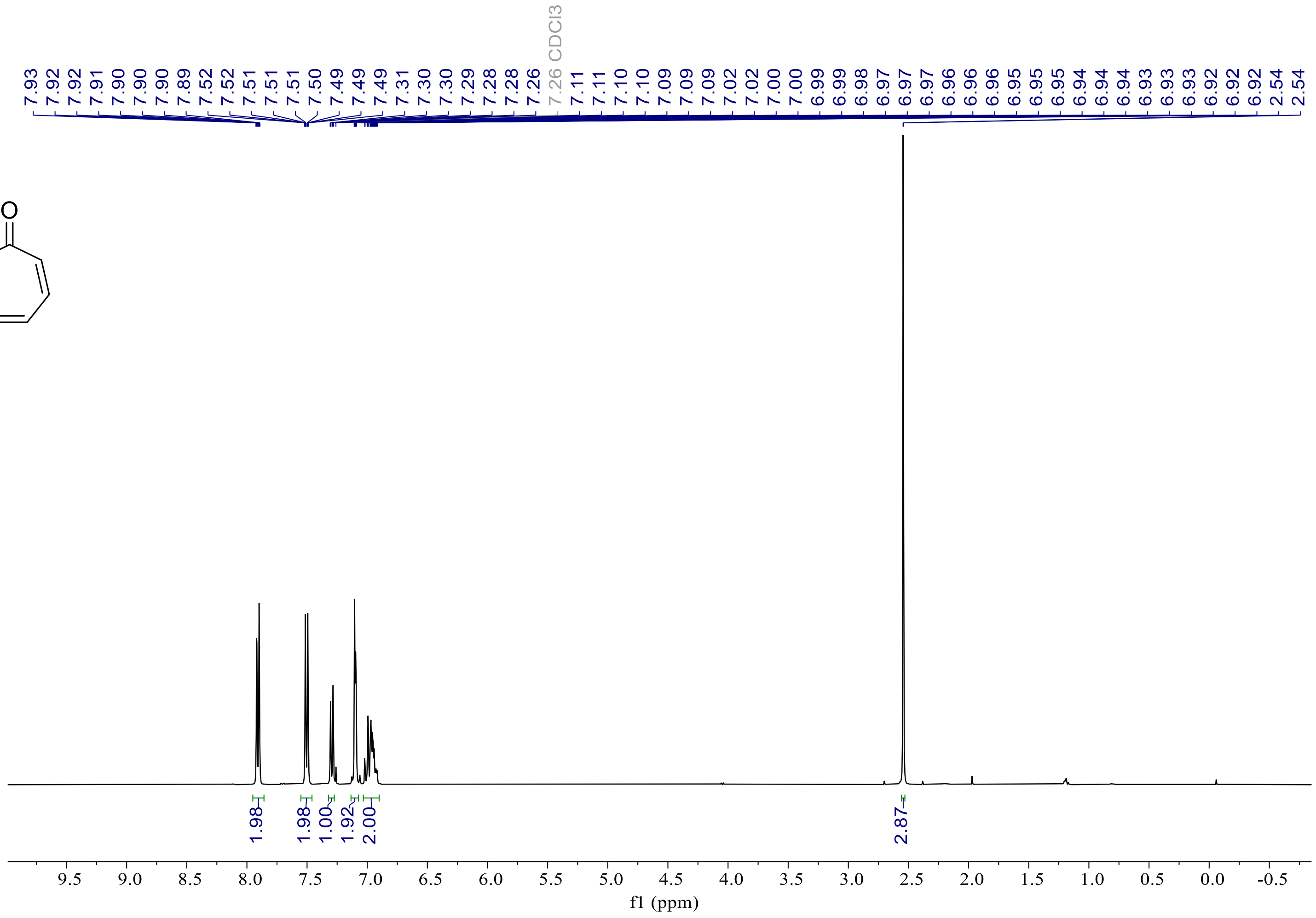

Compound 5h ^{13}C NMR (100 MHz, $CDCl_3$)

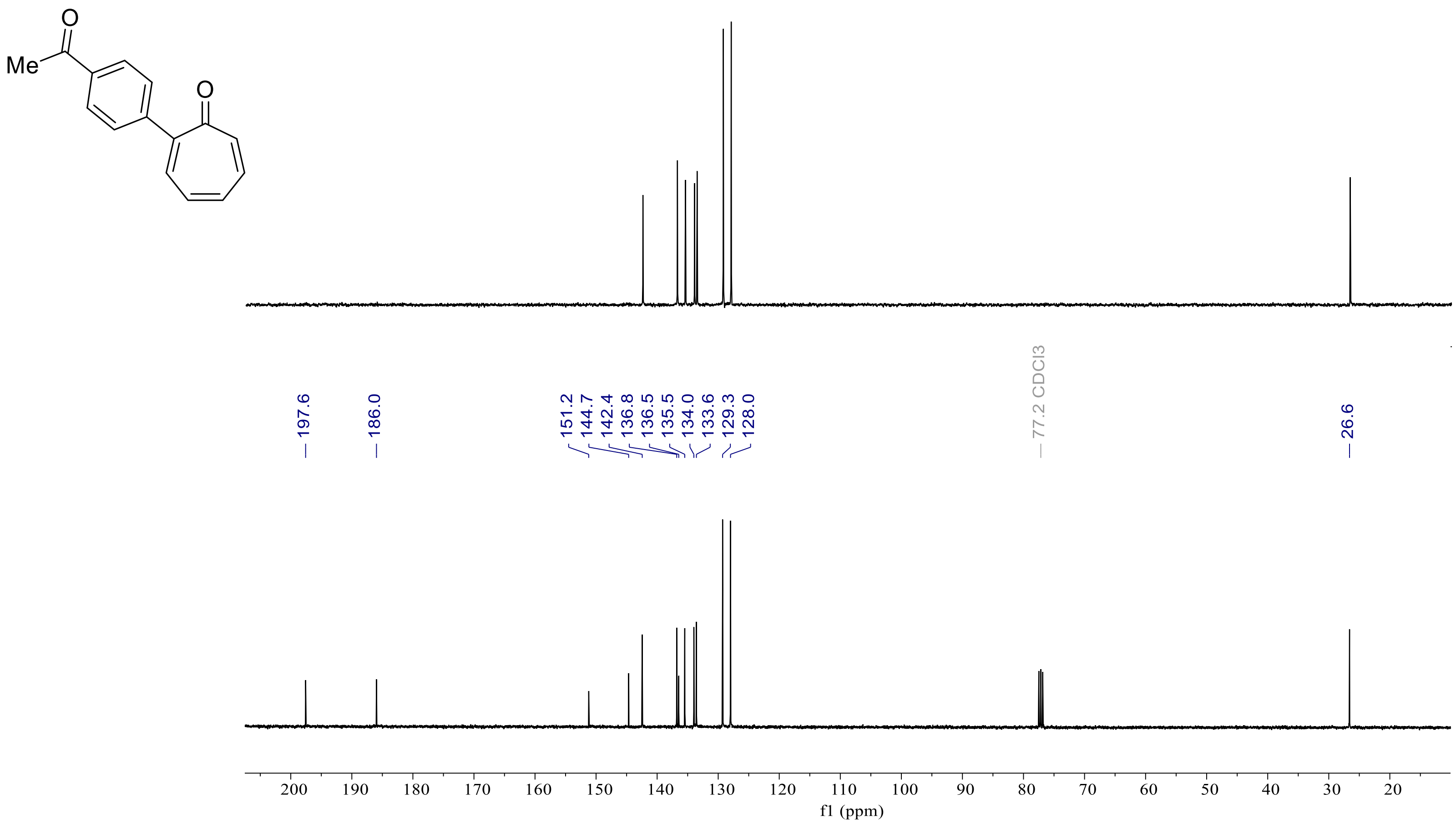

Compound 5h HRMS (ESI-TOF)

**Data Filename**	ESIH202501853.d	**Sample Name**	D4-ZQriv8
**Sample ID**		**Position**	P1-B7
**Instrument Name**	Agilent 6520 Q-TOF	**Acq Method**	20160322_MS_ESIH_POS_1min.m
**Acquired Time**	3/18/2025 2:44:57 PM	**DA Method**	ESI-HR-20231114.m
**Comment**	ESIH by fangsu		

User Spectra

Fragmentor Voltage	Collision Energy	Ionization Mode
170	0	ESI

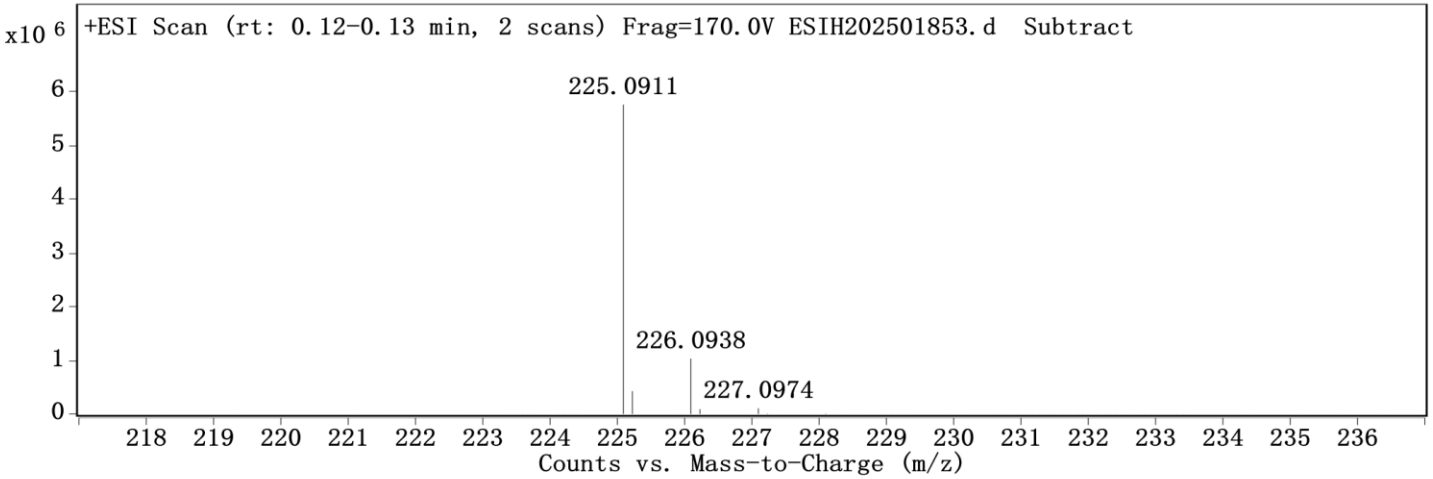

Formula Calculator Results

m/z	Calc m/z	Diff (mDa)	Diff (ppm)	Ion Formula	Ion
225.0911	225.091	-0.06	-0.27	C15 H13 O2	(M+H)+

--- End Of Report ---

Compound 5i ¹H NMR (600 MHz, CDCl₃)

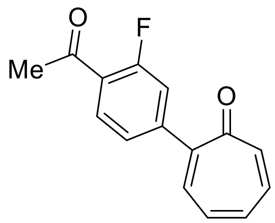

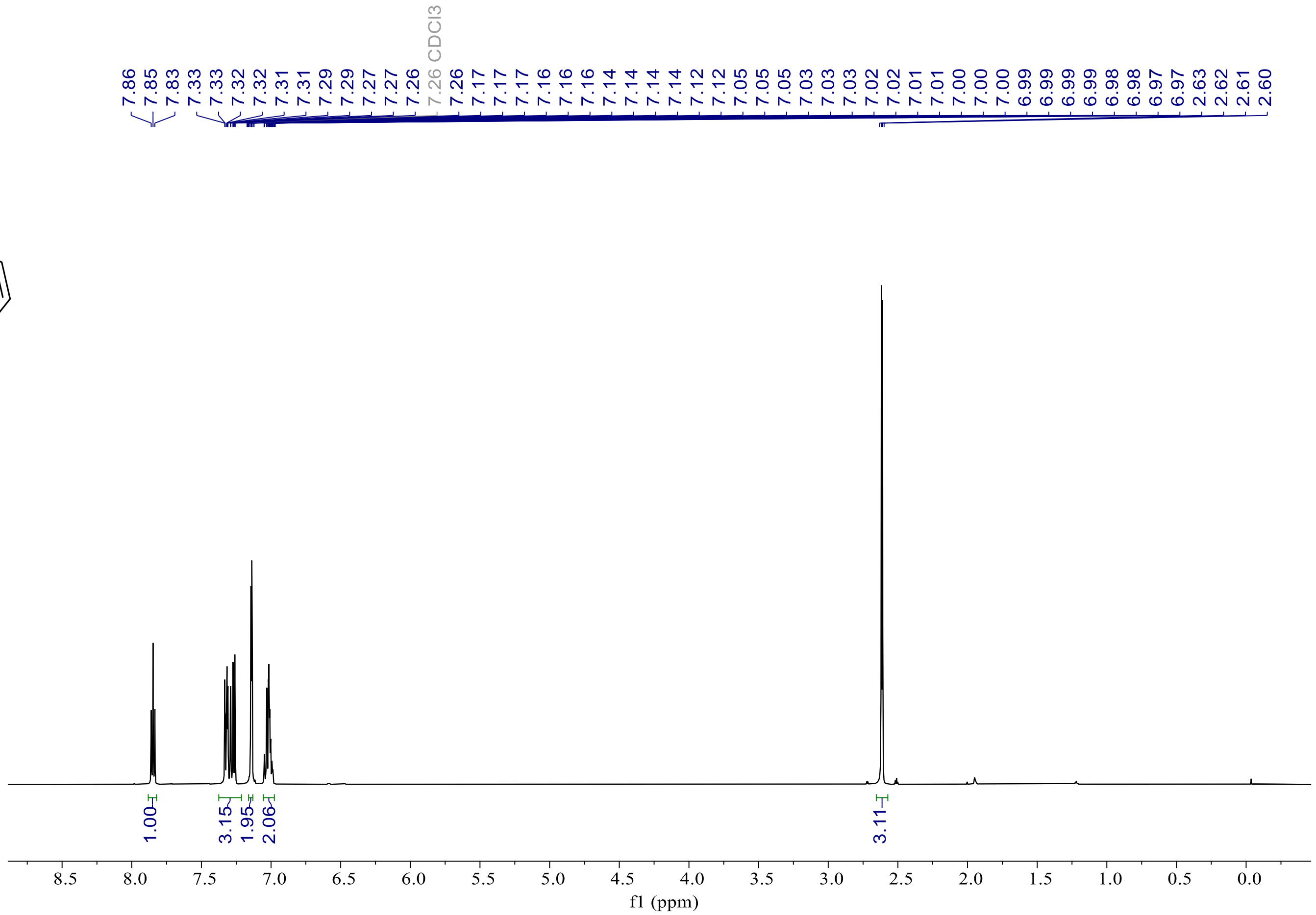

Compound 5i ^{13}C NMR (150 MHz, $CDCl_3$)

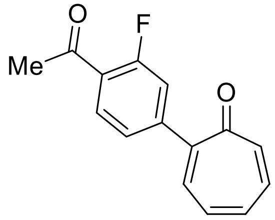

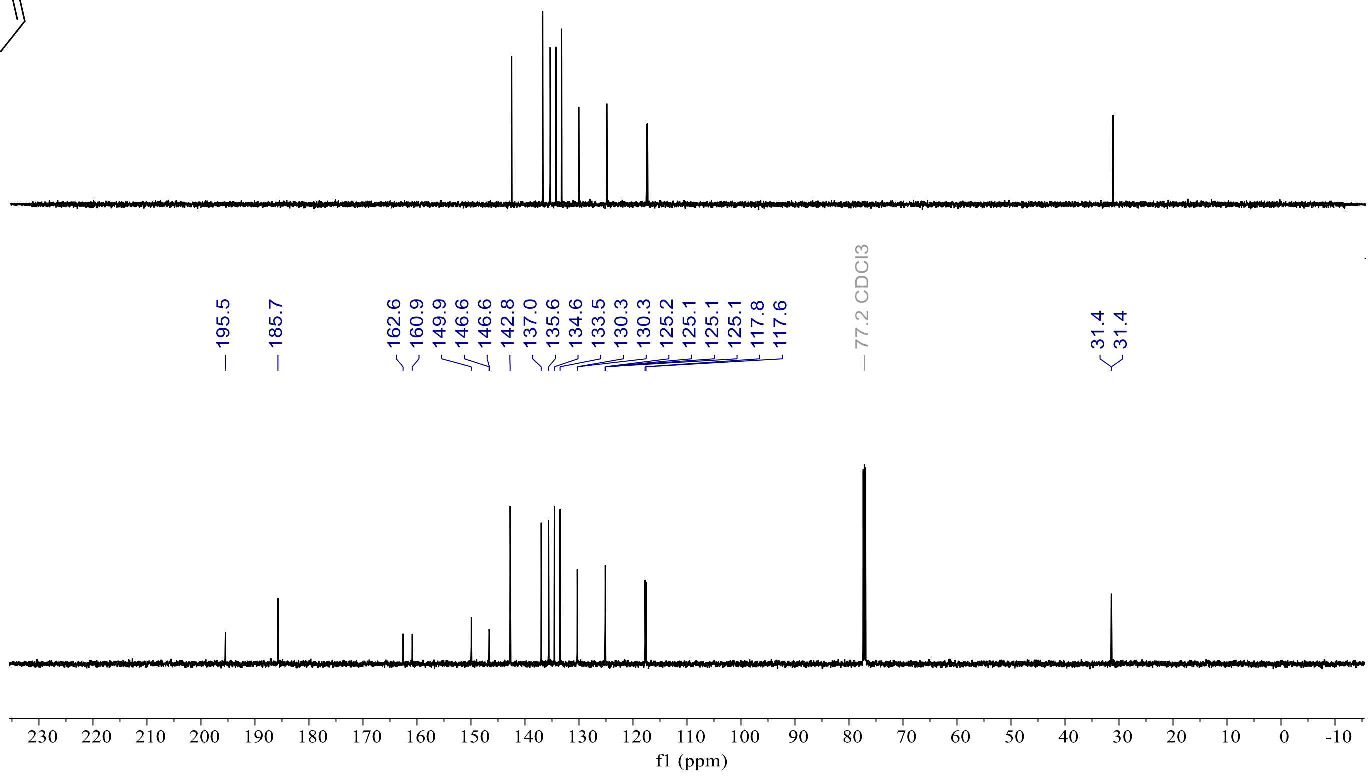

Compound 5i HRMS (ESI-TOF)

**Data Filename**	ESIH202502013.d	**Sample Name**	D4-ZQriv18-2
**Sample ID**		**Position**	P1-A4
**Instrument Name**	Agilent 6520 Q-TOF	**Acq Method**	20160322_MS_ESIH_POS_1min.m
**Acquired Time**	3/25/2025 2:56:40 PM	**DA Method**	ESI-HR-20231114.m
**Comment**	ESIH by fangsu		

User Spectra

**Fragmentor Voltage**	**Collision Energy**	**Ionization Mode**
170	0	ESI

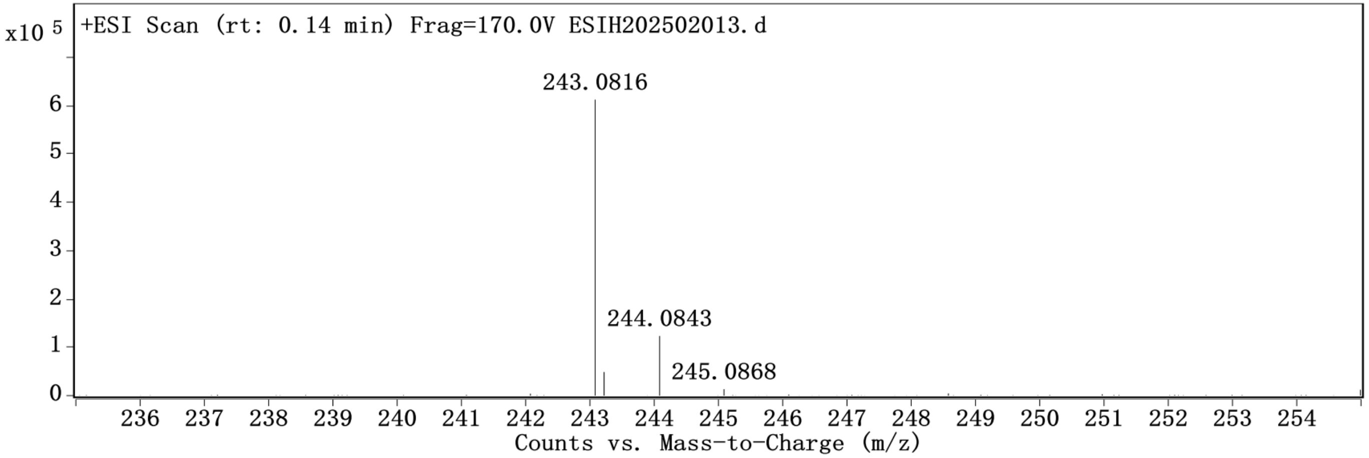

Formula Calculator Results

m/z	Calc m/z	Diff (mDa)	Diff (ppm)	Ion Formula	Ion
243.0816	243.0816	-0.06	-0.26	C15 H12 F O2	(M+H)+

--- End Of Report ---

Compound 5j ^{1}H NMR (600 MHz, $CDCl_3$)

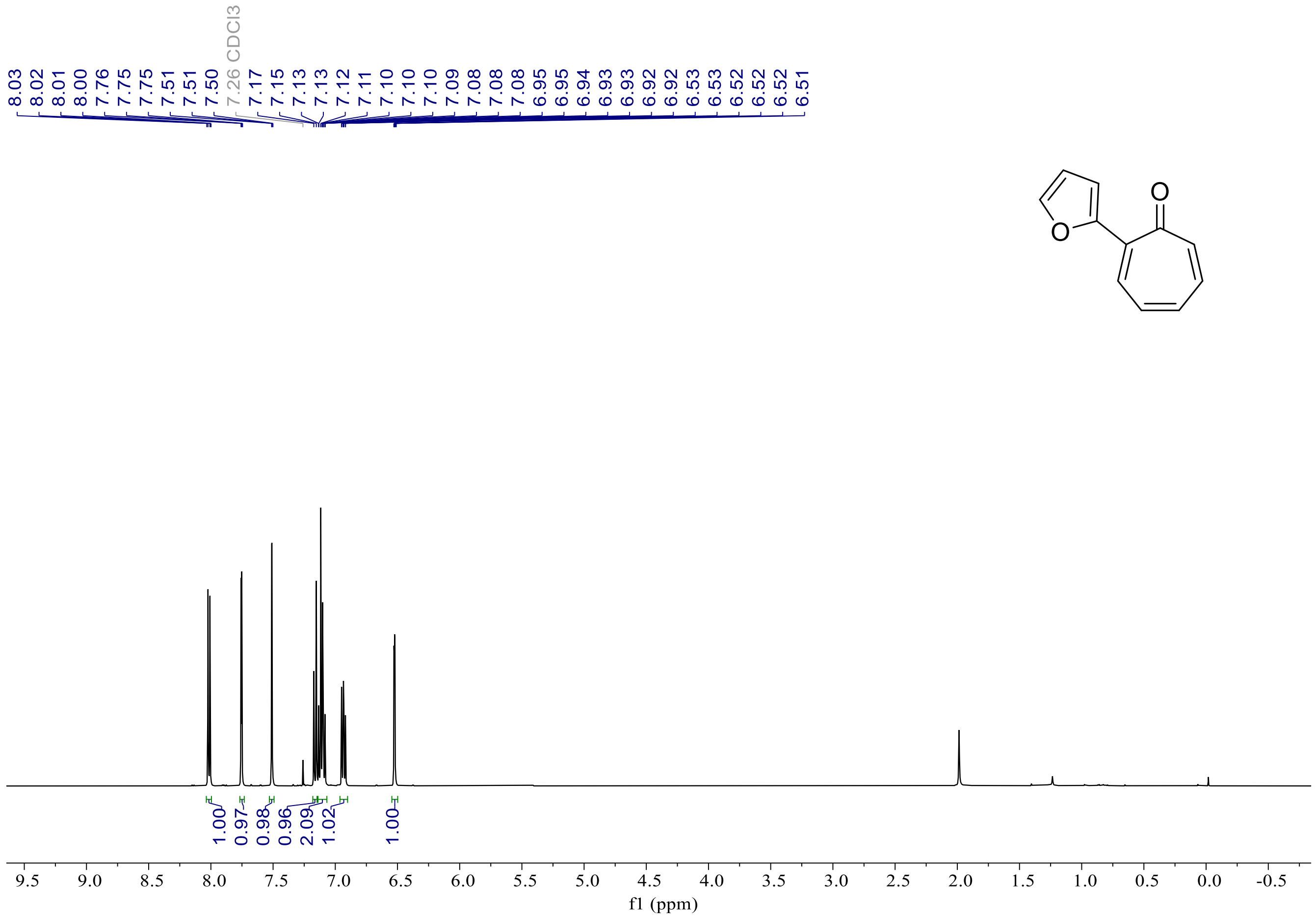

Compound 5j ^{13}C NMR (150 MHz, $CDCl_3$)

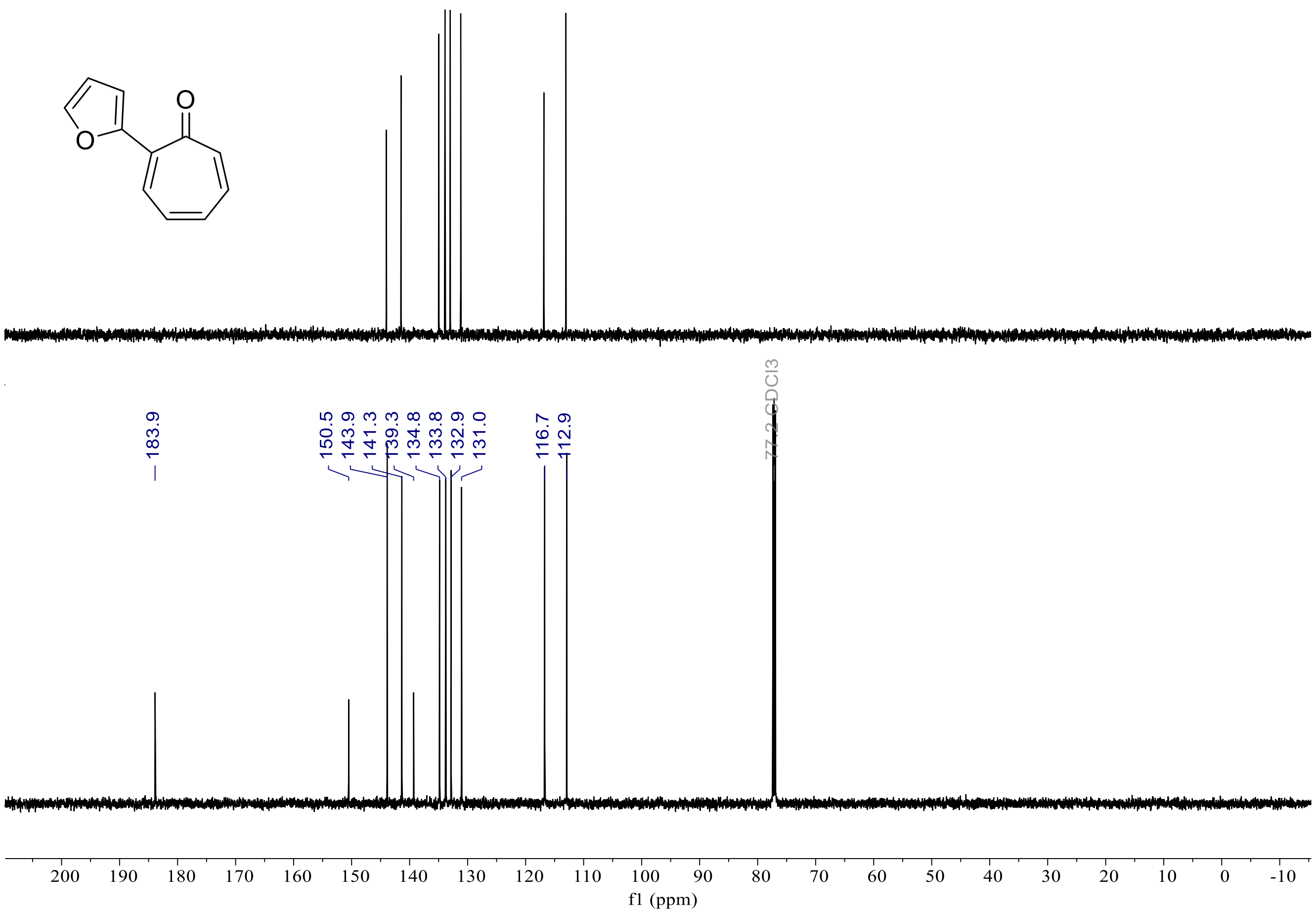

Compound 5j HRMS (ESI-TOF)

**Data Filename**	ESIH202404278.d	**Sample Name**	D4-ZQT18-18
**Sample ID**		**Position**	P1-A3
**Instrument Name**	Agilent 6520 Q-TOF	**Acq Method**	20160322_MS_ESIH_POS_1min.m
**Acquired Time**	9/4/2024 2:13:11 PM	**DA Method**	ESI-HR-20231114.m
**Comment**	ESIH by fangsu		

User Spectra

Fragmentor Voltage	Collision Energy	Ionization Mode
170	0	ESI

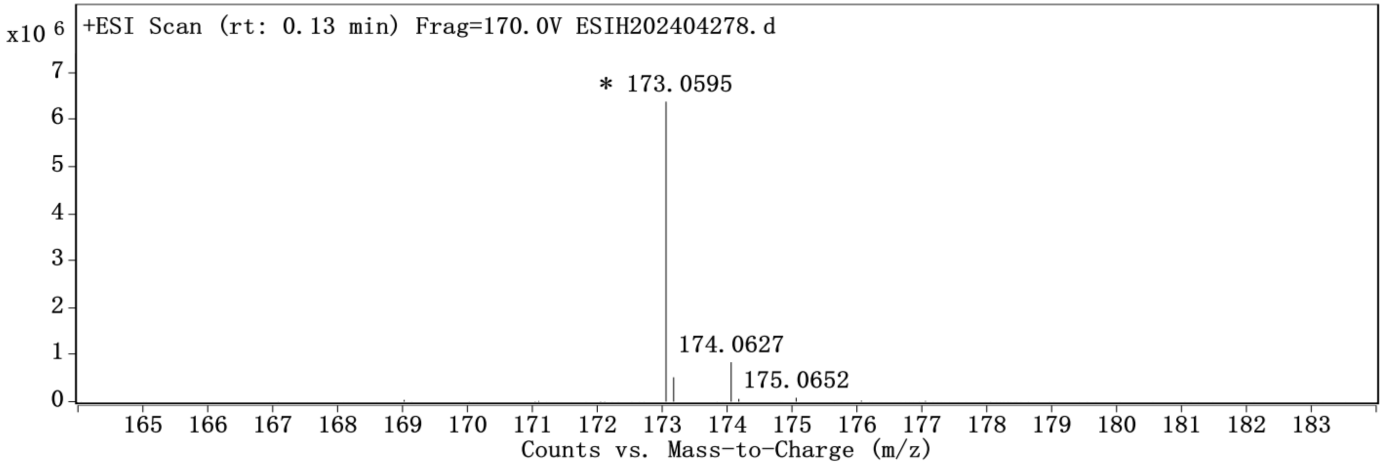

Formula Calculator Results

m/z	Calc m/z	Diff (mDa)	Diff (ppm)	Ion Formula	Ion
173.0595	173.0597	0.16	0.93	C11 H9 O2	(M+H)+

--- End Of Report ---

Compound 5l ¹H NMR (400 MHz, CDCl₃)

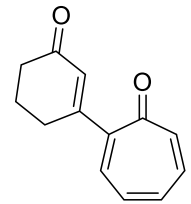

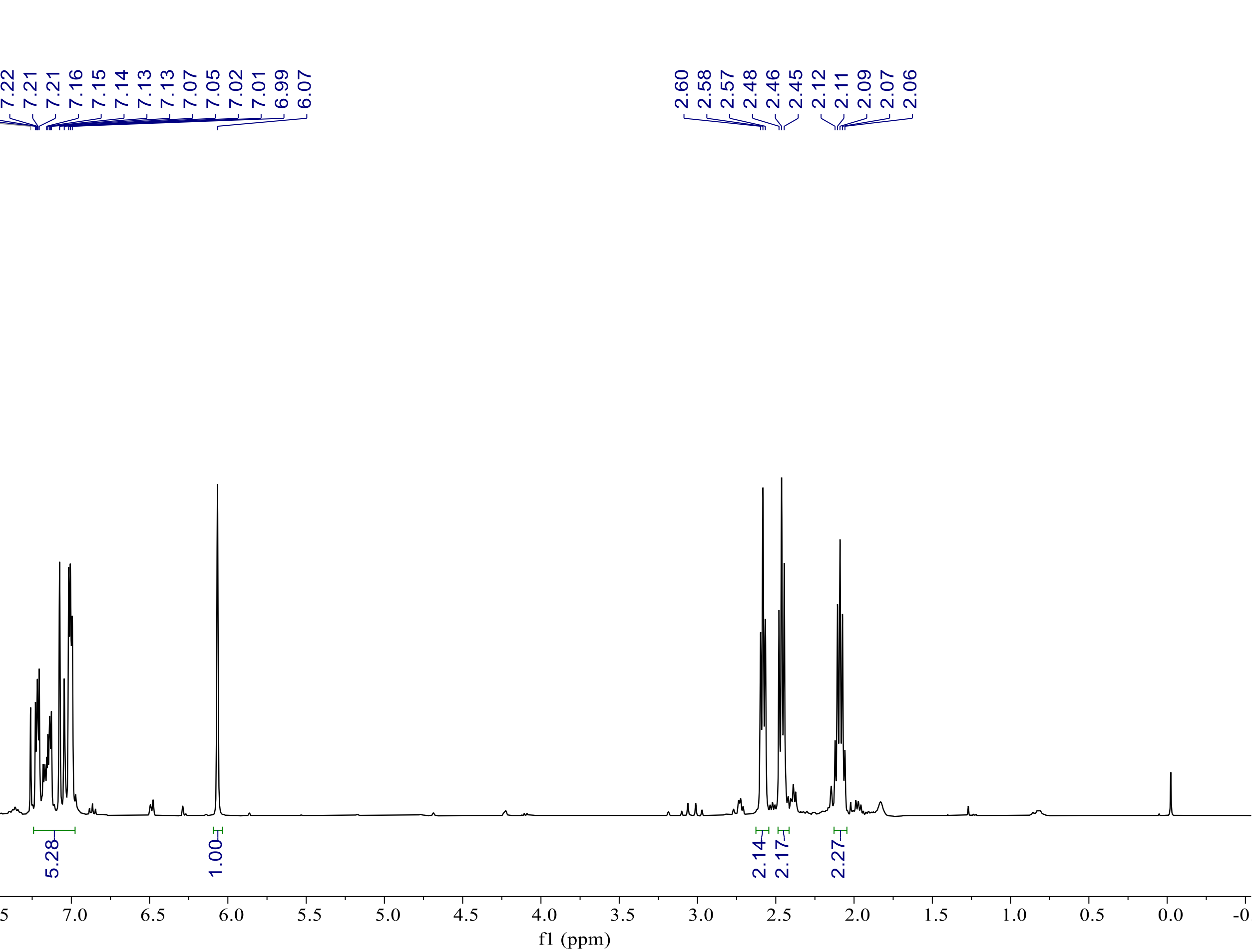

Compound 5l ^{13}C NMR (100 MHz, $CDCl_3$)

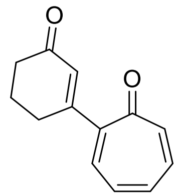

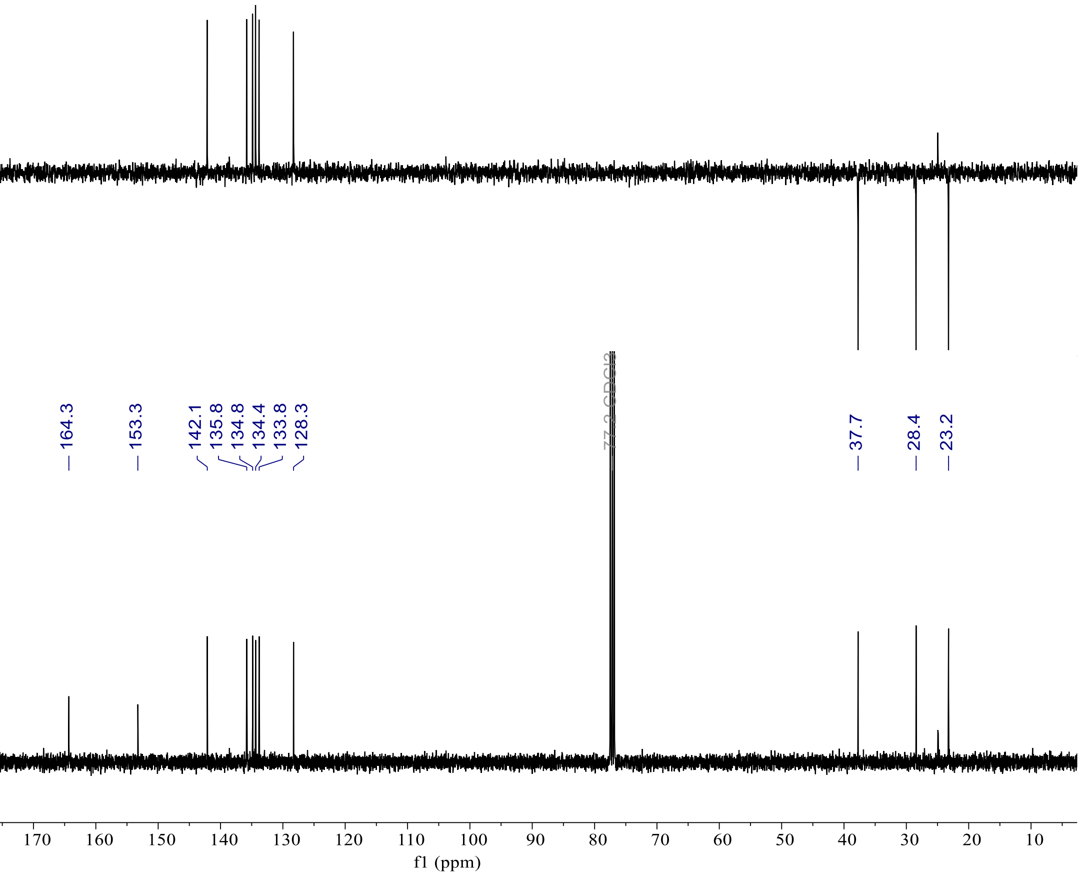

Compound 5l HRMS (ESI-TOF)

**Data Filename**	N_ESIH202502107.d	**Sample Name**	D4-ZQriv18-1
**Sample ID**		**Position**	P1-A1
**Instrument Name**	Agilent 6520 Q-TOF	**Acq Method**	20160324_MS_ESIH_NEG_1min.m
**Acquired Time**	4/1/2025 2:36:05 PM	**DA Method**	ESI-HR-20231114.m
**Comment**	ESIH by fangsu		

User Spectra

Fragmentor Voltage	Collision Energy	Ionization Mode
170	0	ESI

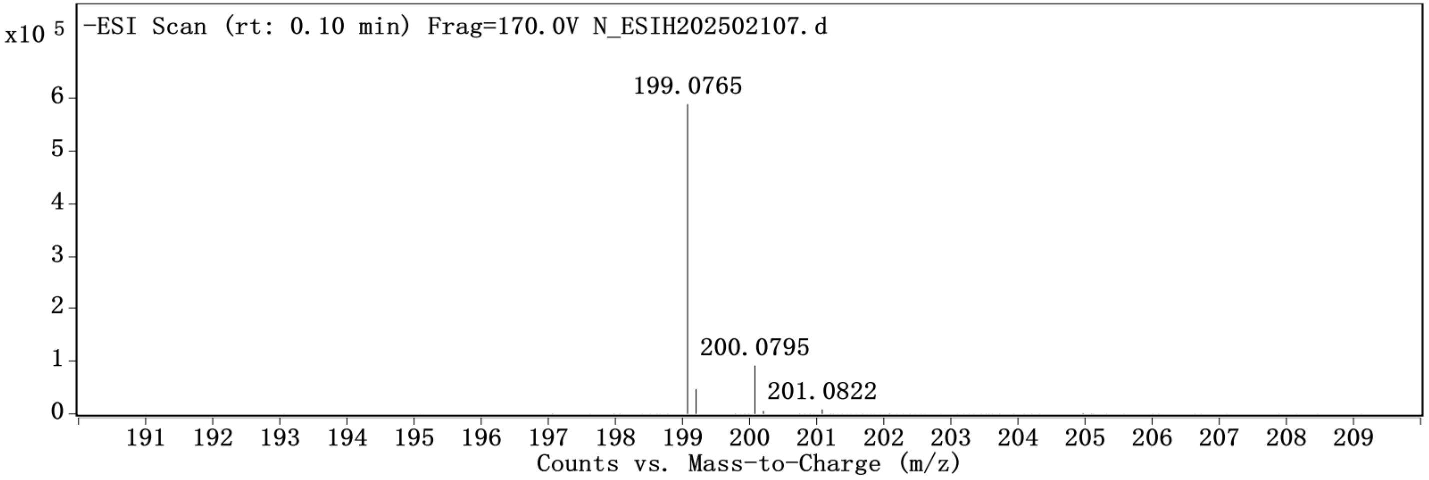

Formula Calculator Results

m/z	Calc m/z	Diff (mDa)	Diff (ppm)	Ion Formula	Ion
199.0765	199.0765	-0.04	-0.19	C13 H11 O2	(M-H)-

--- End Of Report ---

Compound 5n ^{1}H NMR (600 MHz, CDCl3)

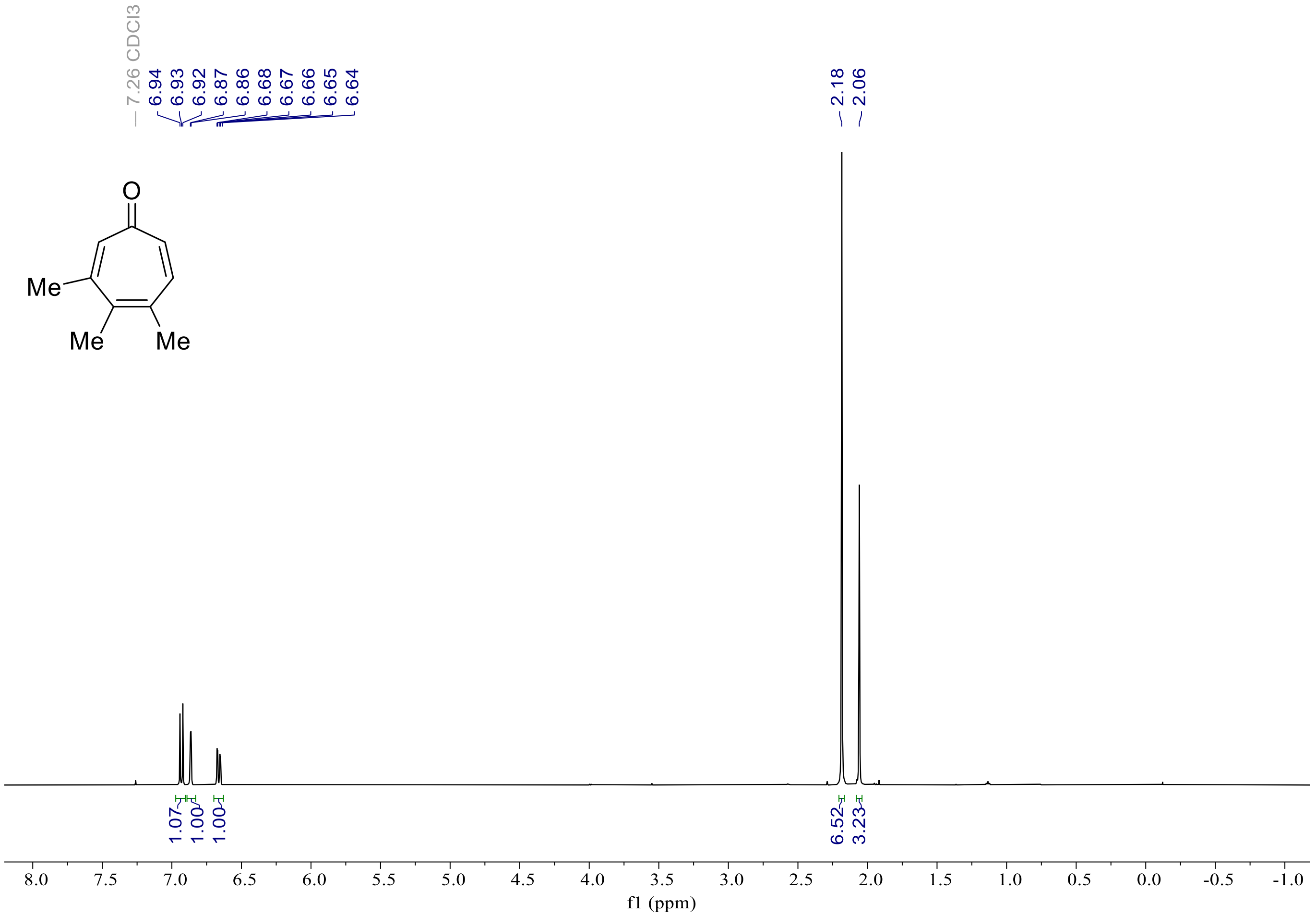

Compound 5n ^{13}C NMR (150 MHz, $CDCl_3$)

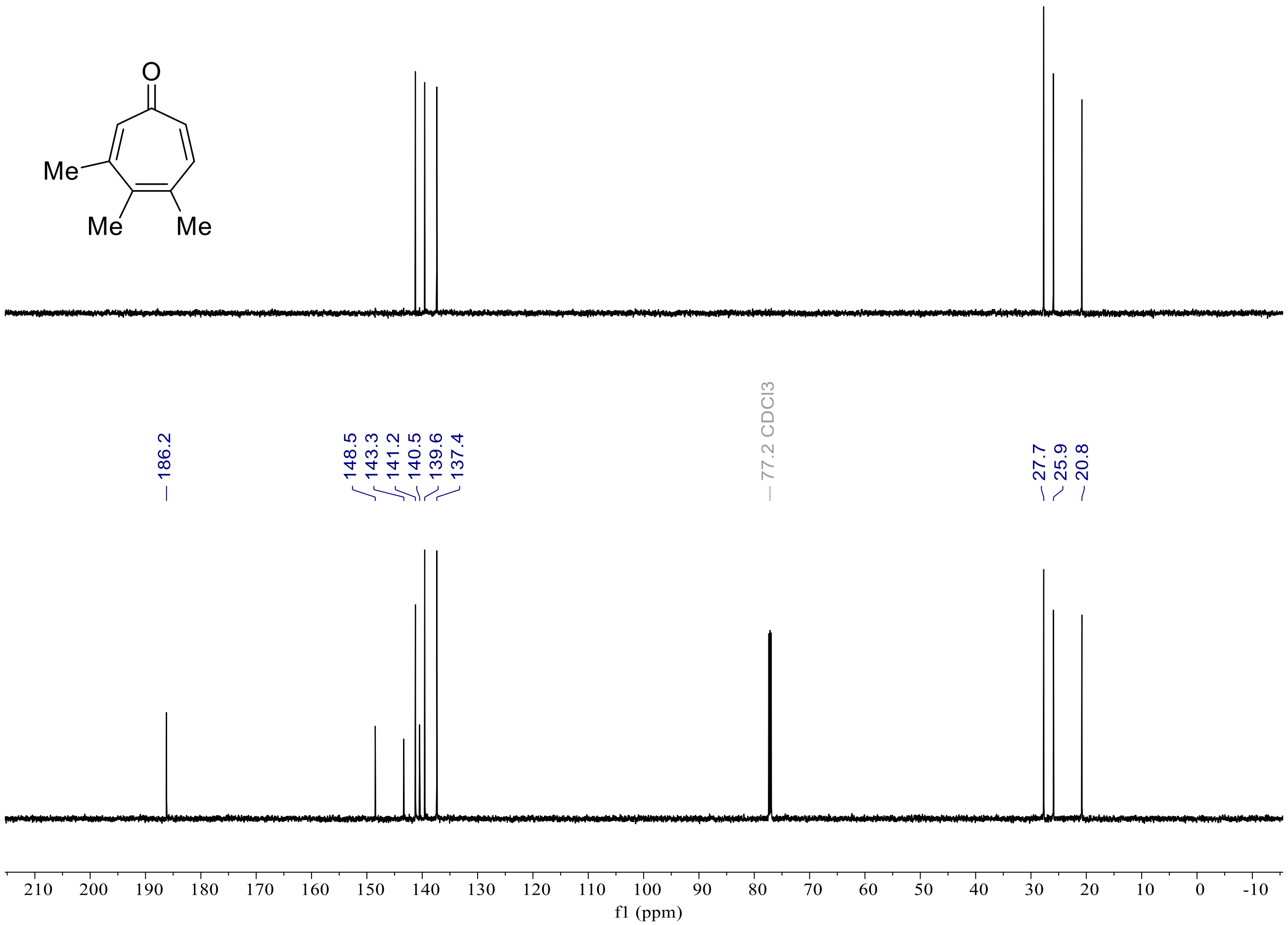

Compound 5n HRMS (ESI-TOF)

**Data Filename**	ESIH202501963.d	**Sample Name**	D4-ZQriv13
**Sample ID**		**Position**	P1-A4
**Instrument Name**	Agilent 6520 Q-TOF	**Acq Method**	20160322_MS_ESIH_POS_1min.m
**Acquired Time**	3/21/2025 4:06:00 PM	**DA Method**	ESI-HR-20231114.m
**Comment**	ESIH by fangsu		

User Spectra

Fragmentor Voltage	Collision Energy	Ionization Mode
170	0	ESI

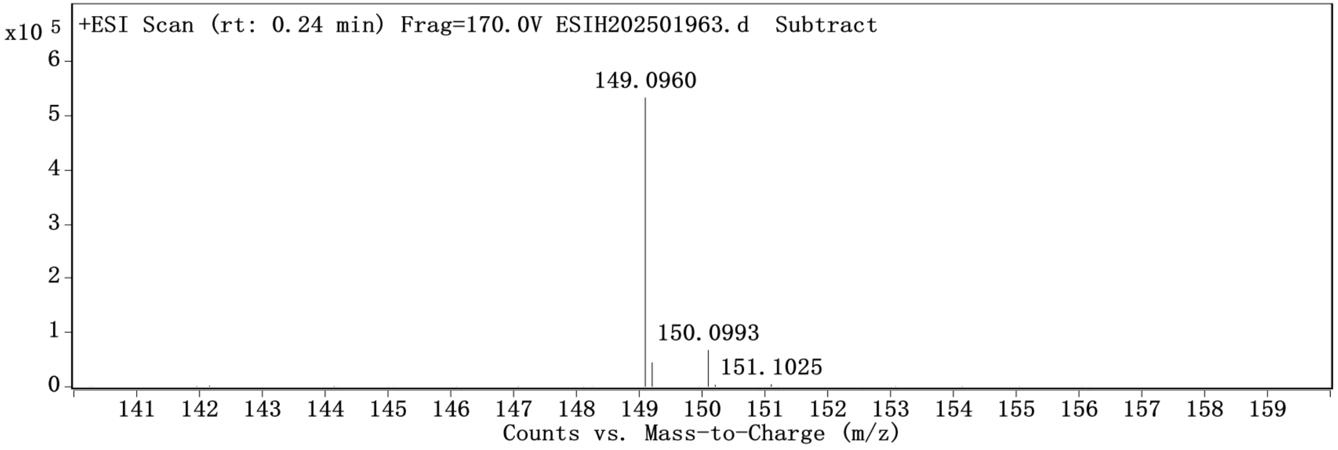

Formula Calculator Results

m/z	Calc m/z	Diff (mDa)	Diff (ppm)	Ion Formula	Ion
149.096	149.0961	0.07	0.46	C10 H13 O	(M+H)+

--- End Of Report ---

Compound 5o ¹H NMR (600 MHz, CDCl₃)

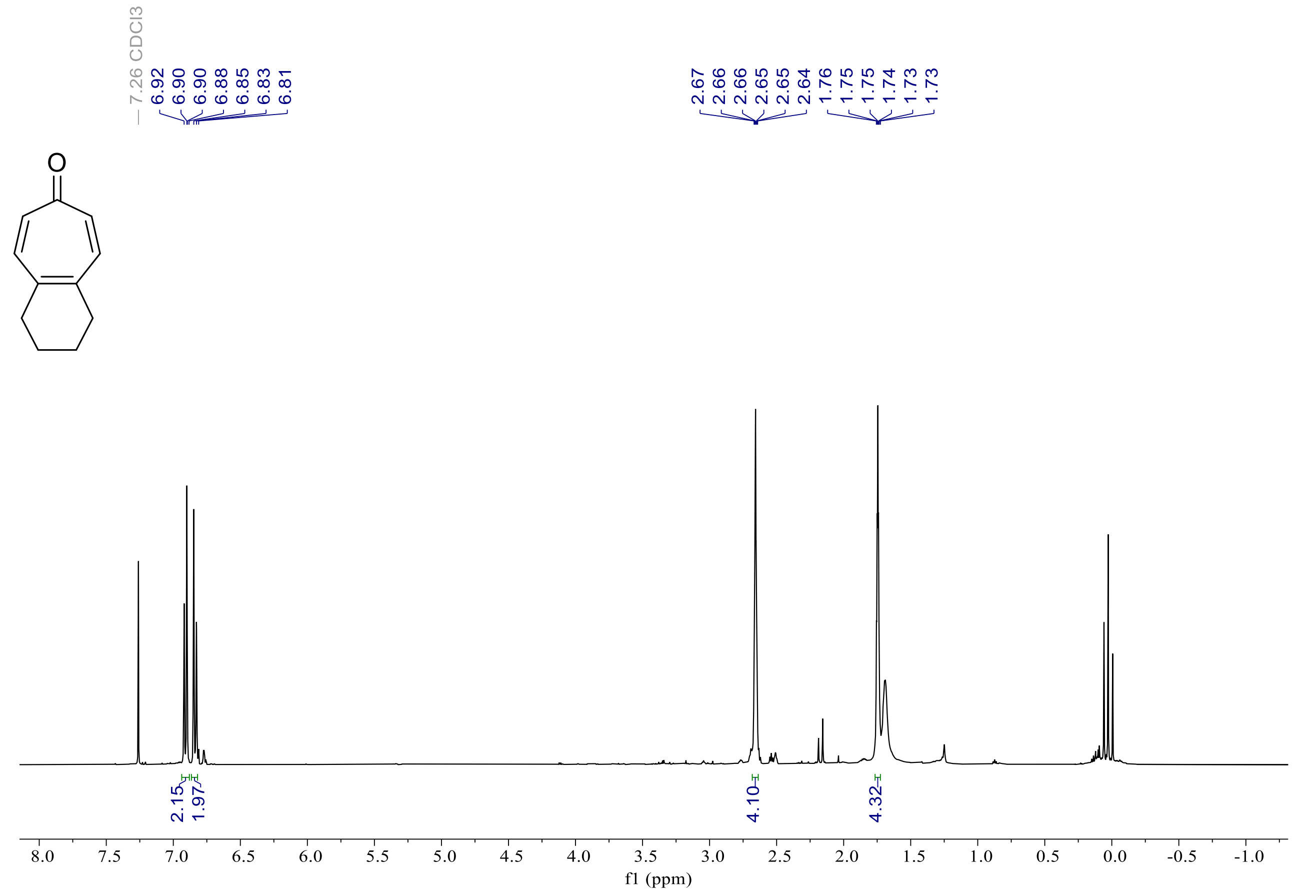

Compound 5o ^{13}C NMR (150 MHz, $CDCl_3$)

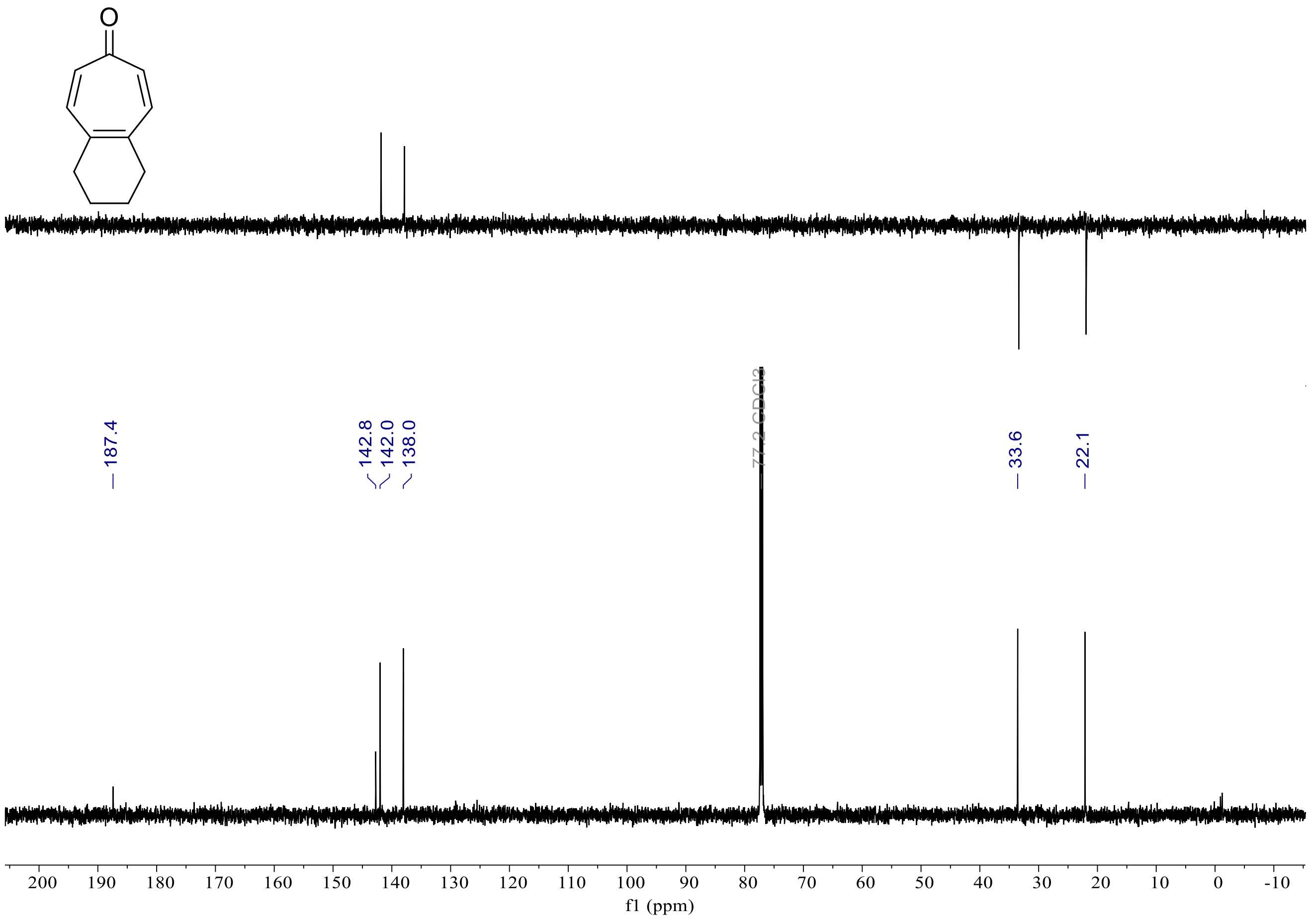

Compound 5o HRMS (ESI-TOF)

**Data Filename**	ESIH202403600.d	**Sample Name**	D4-ZQDe6A
**Sample ID**		**Position**	P1-B5
**Instrument Name**	Agilent 6520 Q-TOF	**Acq Method**	20160322_MS_ESIH_POS_1min.m
**Acquired Time**	7/9/2024 4:17:13 PM	**DA Method**	ESI-HR-20231114.m
**Comment**	ESIH by fangsu		

User Spectra

Fragmentor Voltage	Collision Energy	Ionization Mode
170	0	ESI

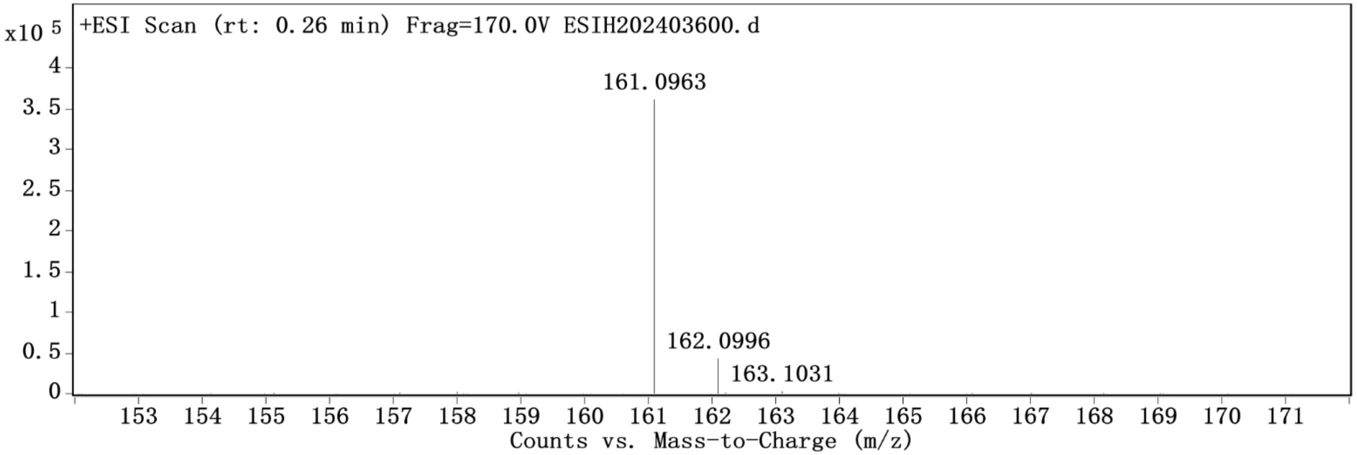

Formula Calculator Results

m/z	Calc m/z	Diff (mDa)	Diff (ppm)	Ion Formula	Ion
161.0963	161.0961	-0.2	-1.24	C11 H13 O	(M+H)+

--- End Of Report ---

Compound S8 ^{1}H NMR (600 MHz, $CDCl_3$)

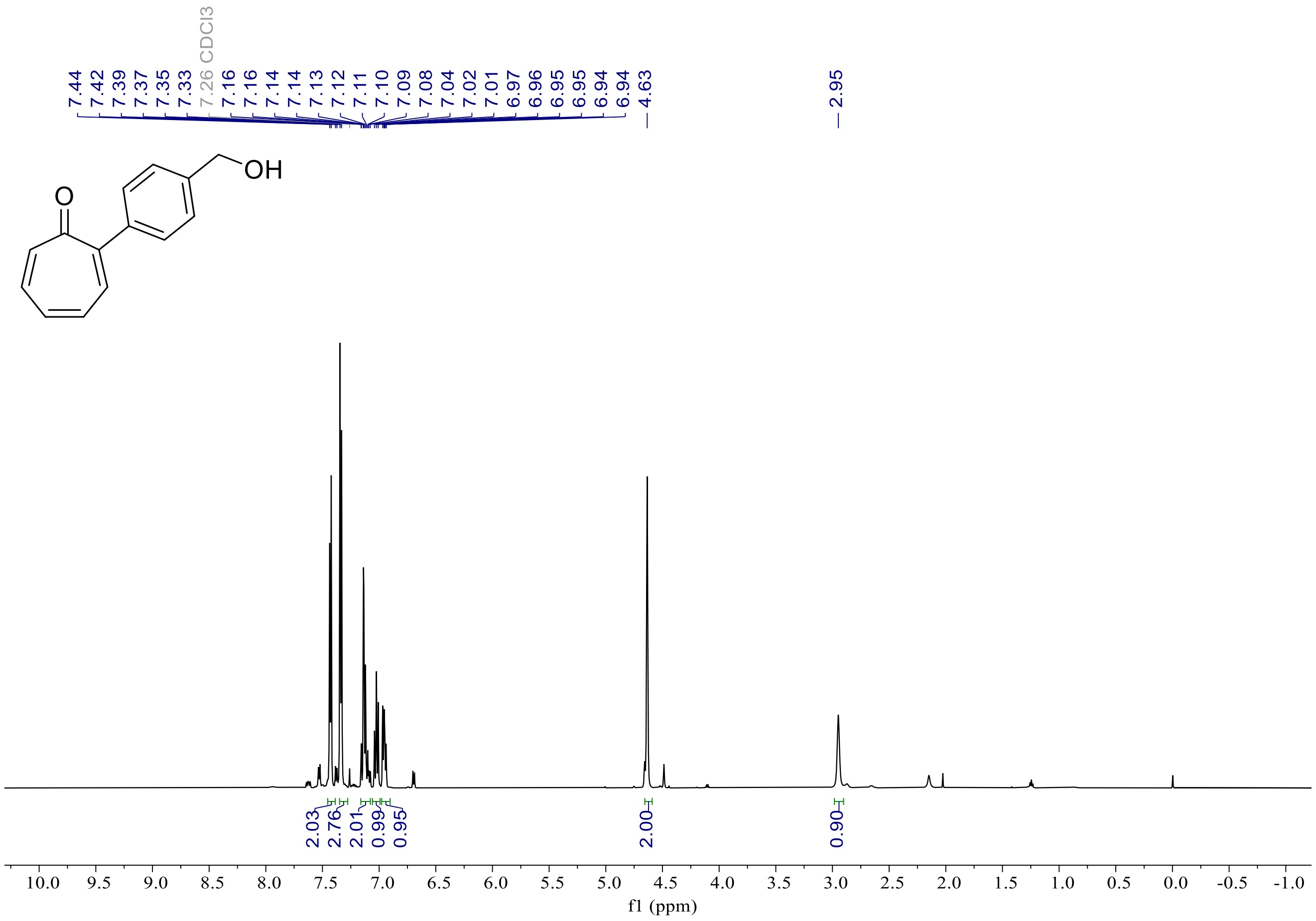

Compound S8 ^{13}C NMR (150 MHz, CDCl$_3$)

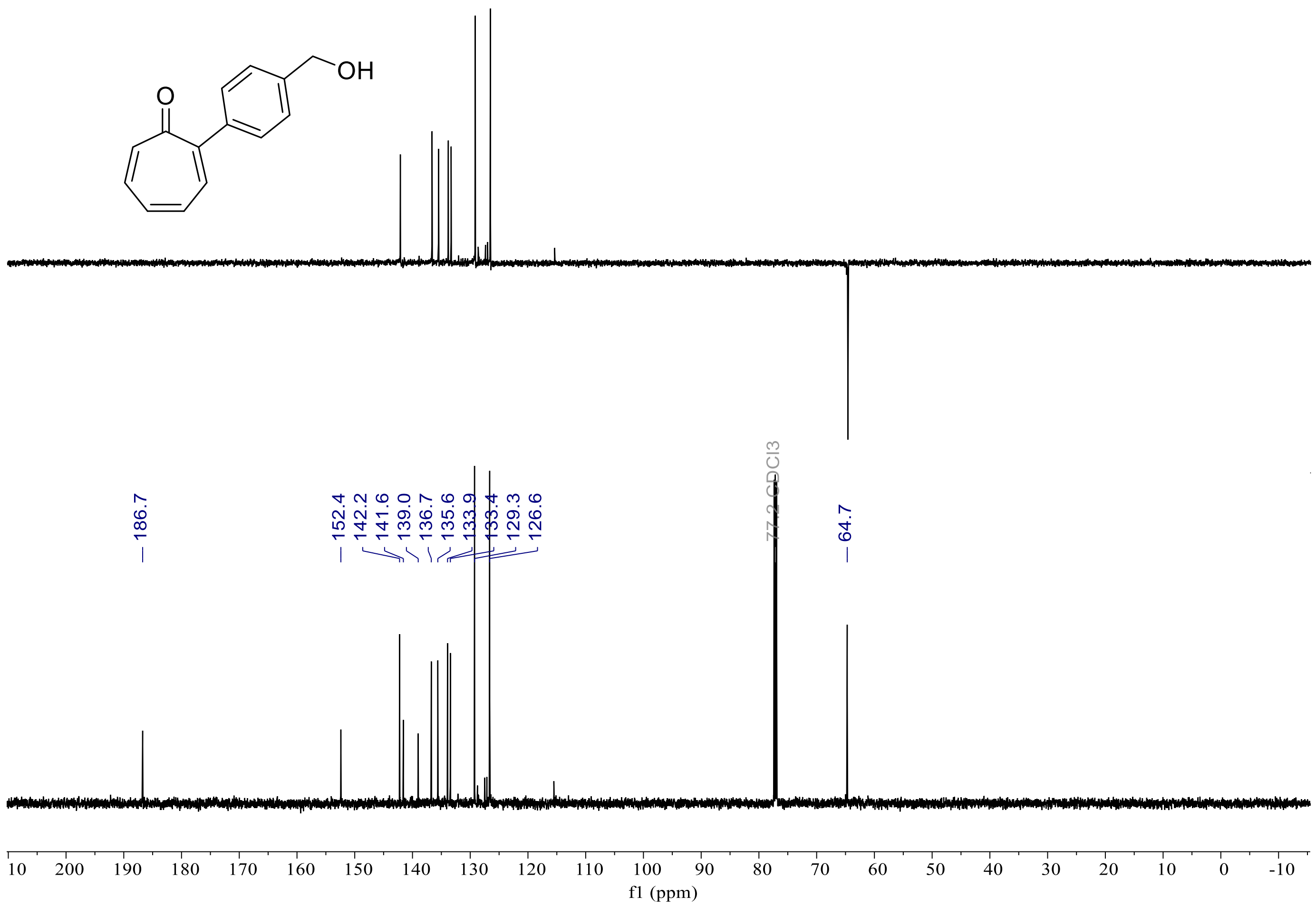

Compound S8 HRMS (ESI-TOF)

**Data Filename**	ESIH202405714.d	**Sample Name**	D4-ZDG2
**Sample ID**		**Position**	P1-D5
**Instrument Name**	Agilent 6520 Q-TOF	**Acq Method**	20160322_MS_ESIH_POS_1min.m
**Acquired Time**	12/4/2024 4:36:04 PM	**DA Method**	ESI-HR-20231114.m
**Comment**	ESIH by fangsu		

User Spectra

Fragmentor Voltage	Collision Energy	Ionization Mode
170	0	ESI

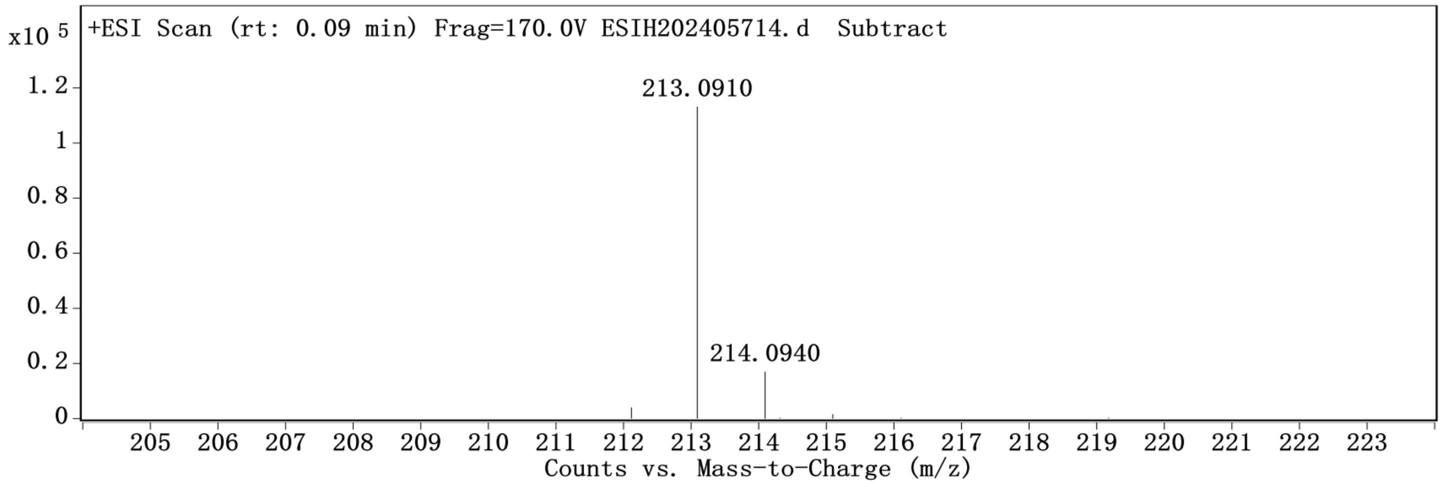

Formula Calculator Results

m/z	Calc m/z	Diff (mDa)	Diff (ppm)	Ion Formula	Ion
213.091	213.091	0.01	0.06	C14 H13 O2	(M+H)+

--- End Of Report ---

Compound 5q ¹H NMR (400 MHz, CDCl₃)

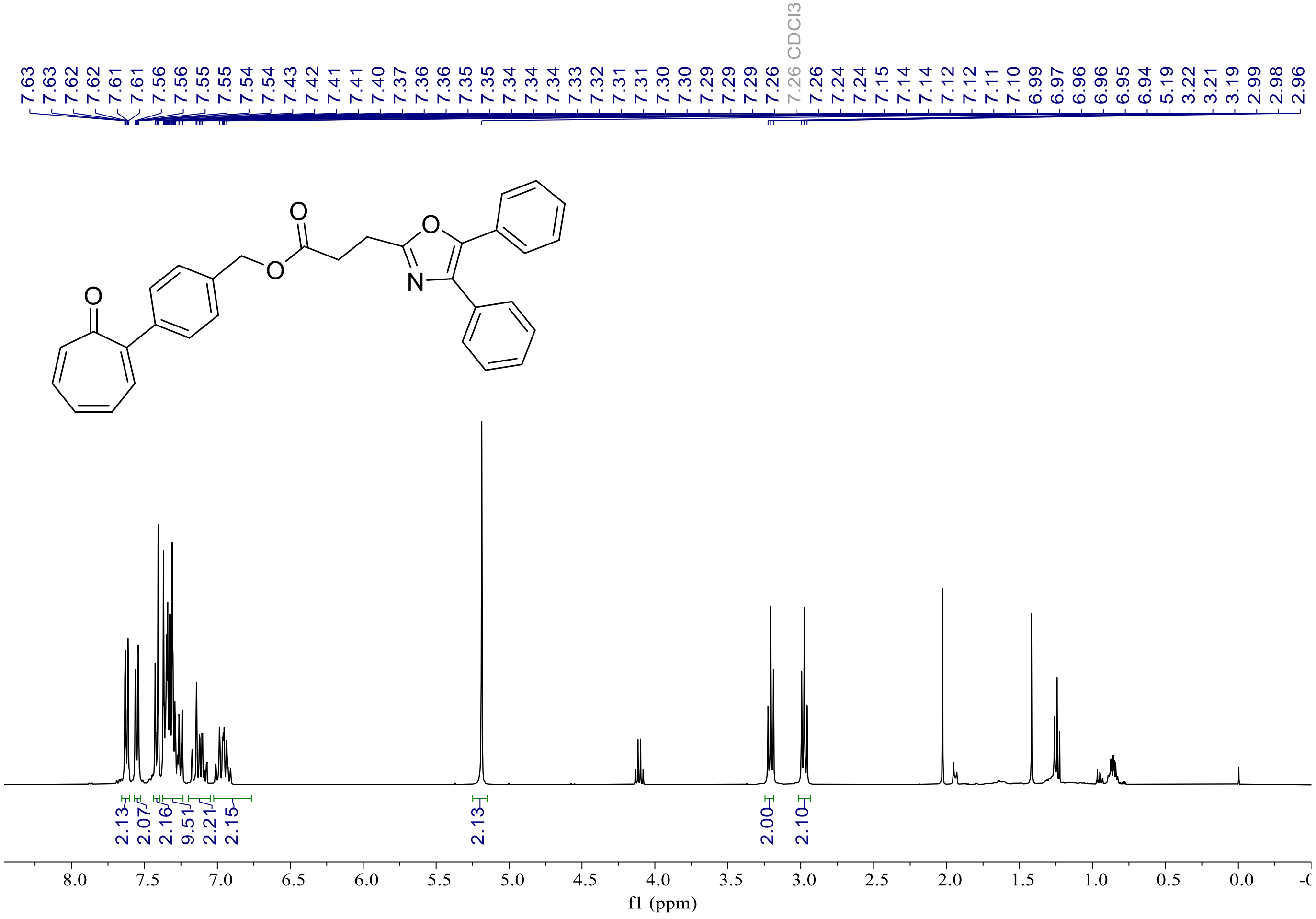

Compound 5q ^{13}C NMR (150 MHz, $CDCl_3$)

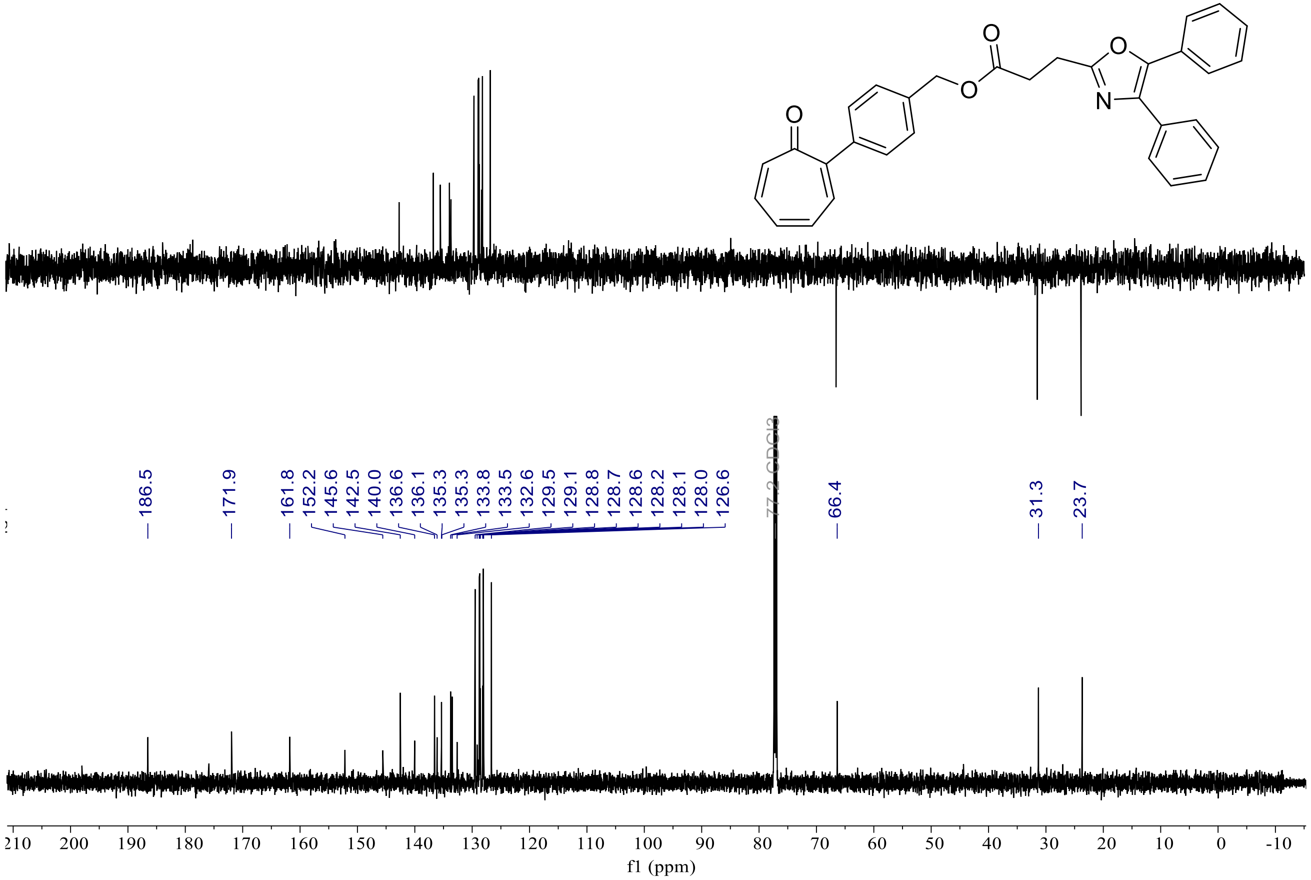

Compound 5q HRMS (ESI-TOF):

**Data Filename**	ESIH202405819.d	**Sample Name**	D4-ZDG3-1B
**Sample ID**		**Position**	P1-E6
**Instrument Name**	Agilent 6520 Q-TOF	**Acq Method**	20160322_MS_ESIH_POS_1min.m
**Acquired Time**	12/6/2024 11:05:58 AM	**DA Method**	ESI-HR-20231114.m
**Comment**	ESIH by fangsu		

User Spectra

Fragmentor Voltage	Collision Energy	Ionization Mode
170	0	ESI

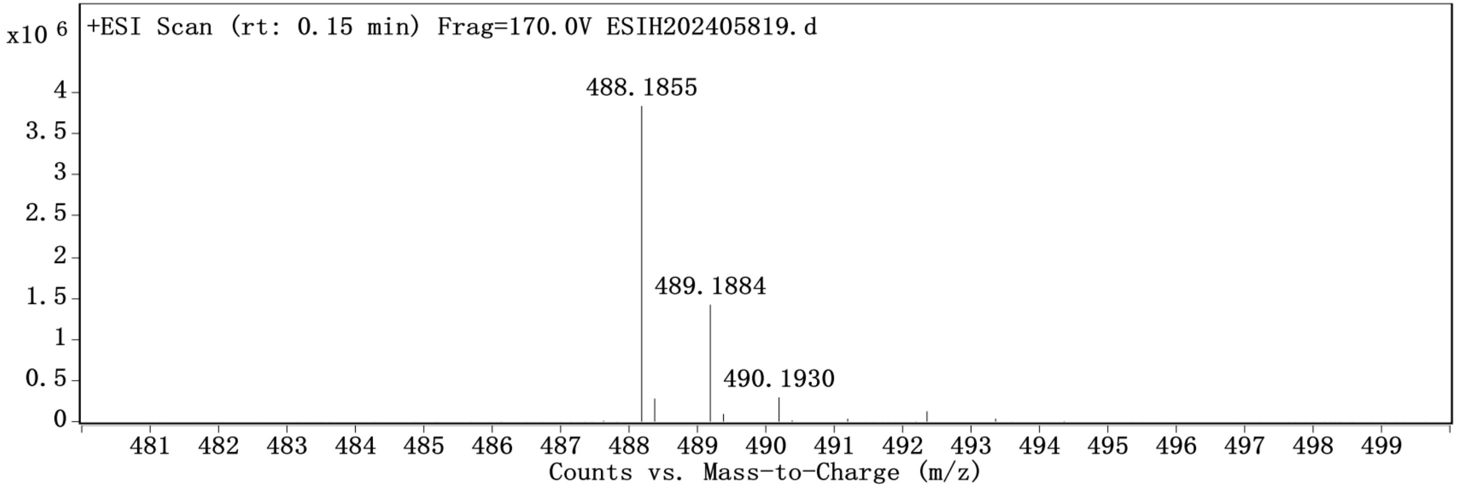

Formula Calculator Results

m/z	Calc m/z	Diff (mDa)	Diff (ppm)	Ion Formula	Ion
488.1855	488.1856	0.14	0.29	C32 H26 N O4	(M+H)+

--- End Of Report ---

Compound 5r ¹H NMR (600 MHz, $CDCl_3$)

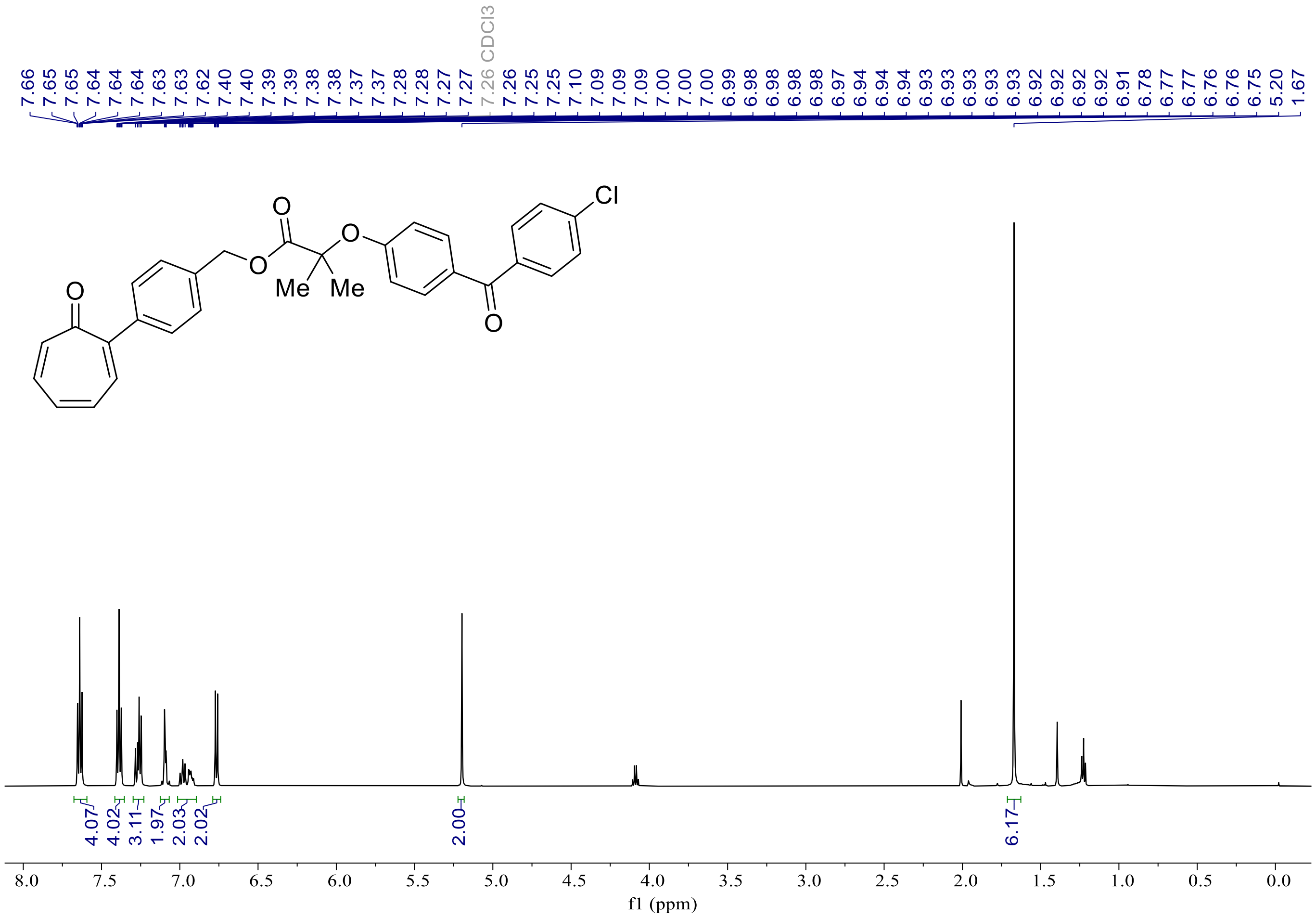

Compound 5r ^{13}C NMR (150 MHz, CDCl$_3$)

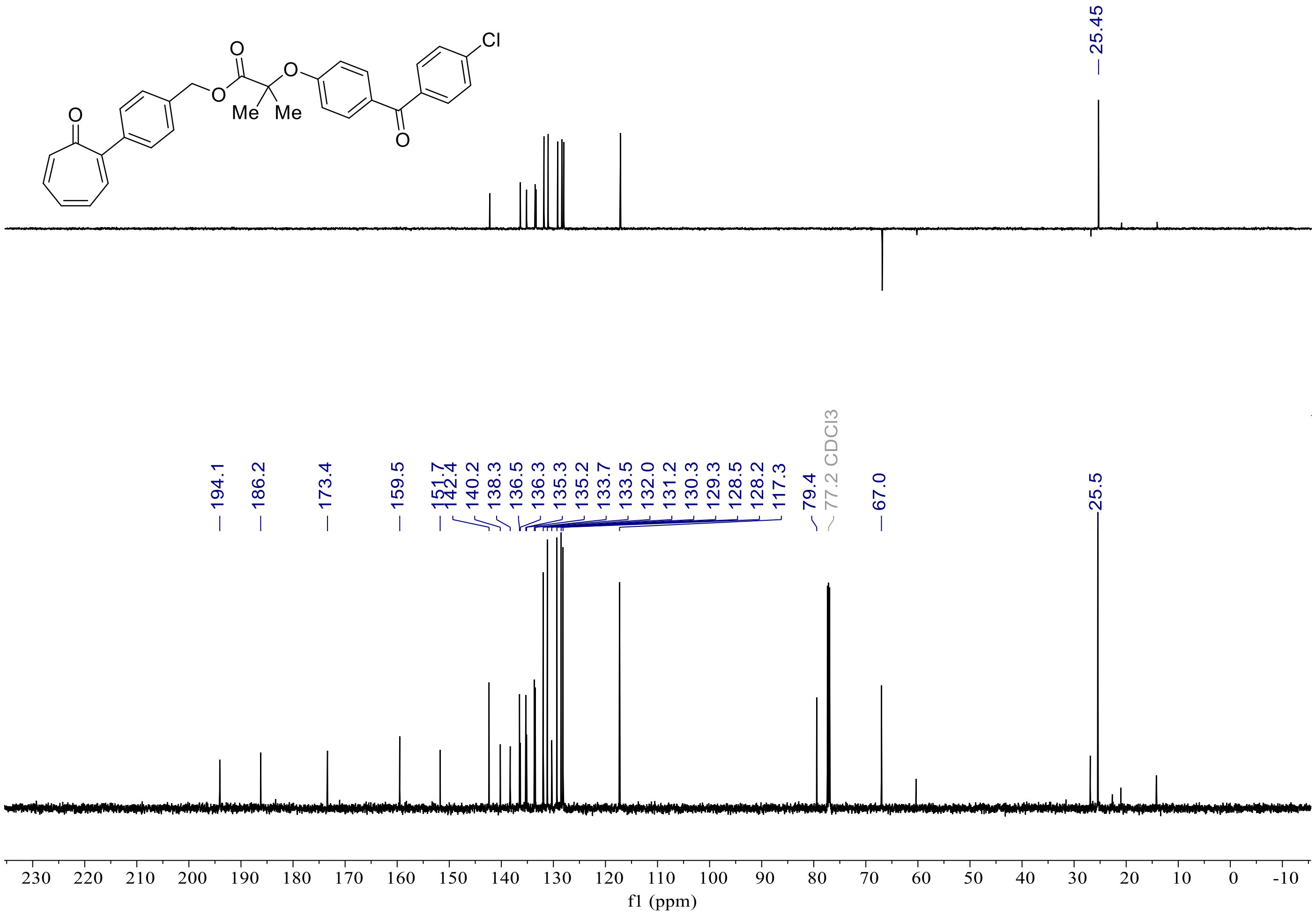

Compound 5r HRMS (ESI-TOF)

**Data Filename**	ESIH202405820.d	**Sample Name**	D4-ZDG3-2B
**Sample ID**		**Position**	P1-E7
**Instrument Name**	Agilent 6520 Q-TOF	**Acq Method**	20160322_MS_ESIH_POS_1min.m
**Acquired Time**	12/6/2024 11:07:14 AM	**DA Method**	ESI-HR-20231114.m
**Comment**	ESIH by fangsu		

User Spectra

Fragmentor Voltage	Collision Energy	Ionization Mode
170	0	ESI

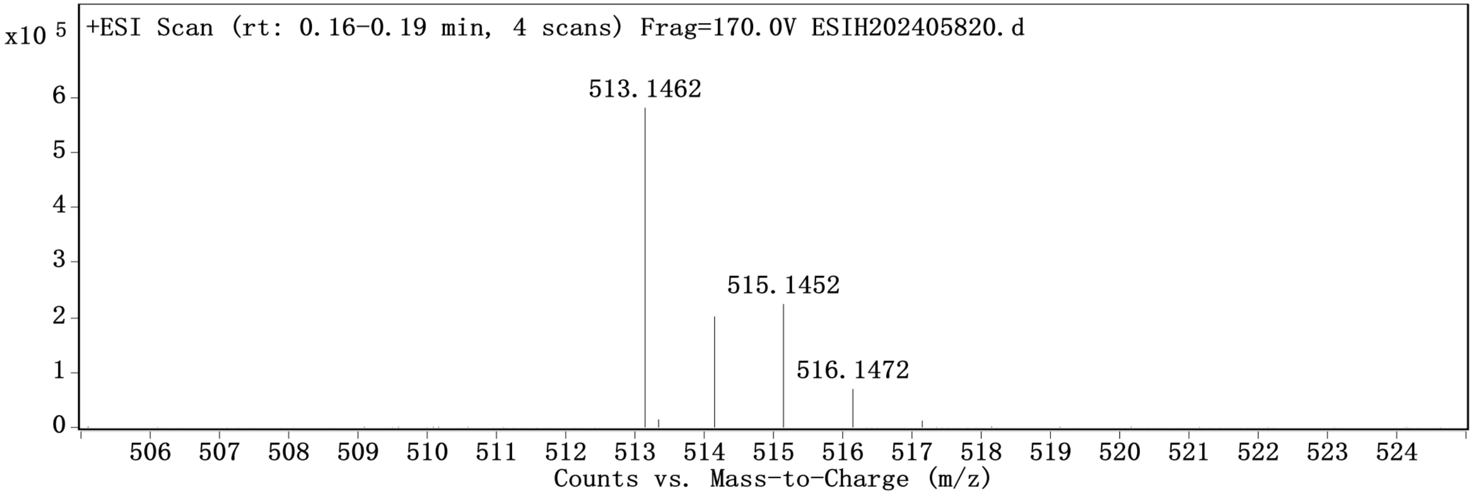

Formula Calculator Results

m/z	Calc m/z	Diff (mDa)	Diff (ppm)	Ion Formula	Ion
513.1462	513.1463	0.1	0.2	C31 H26 Cl O5	(M+H)+

--- End Of Report ---

Compound 5s ¹H NMR (600 MHz, CDCl₃)

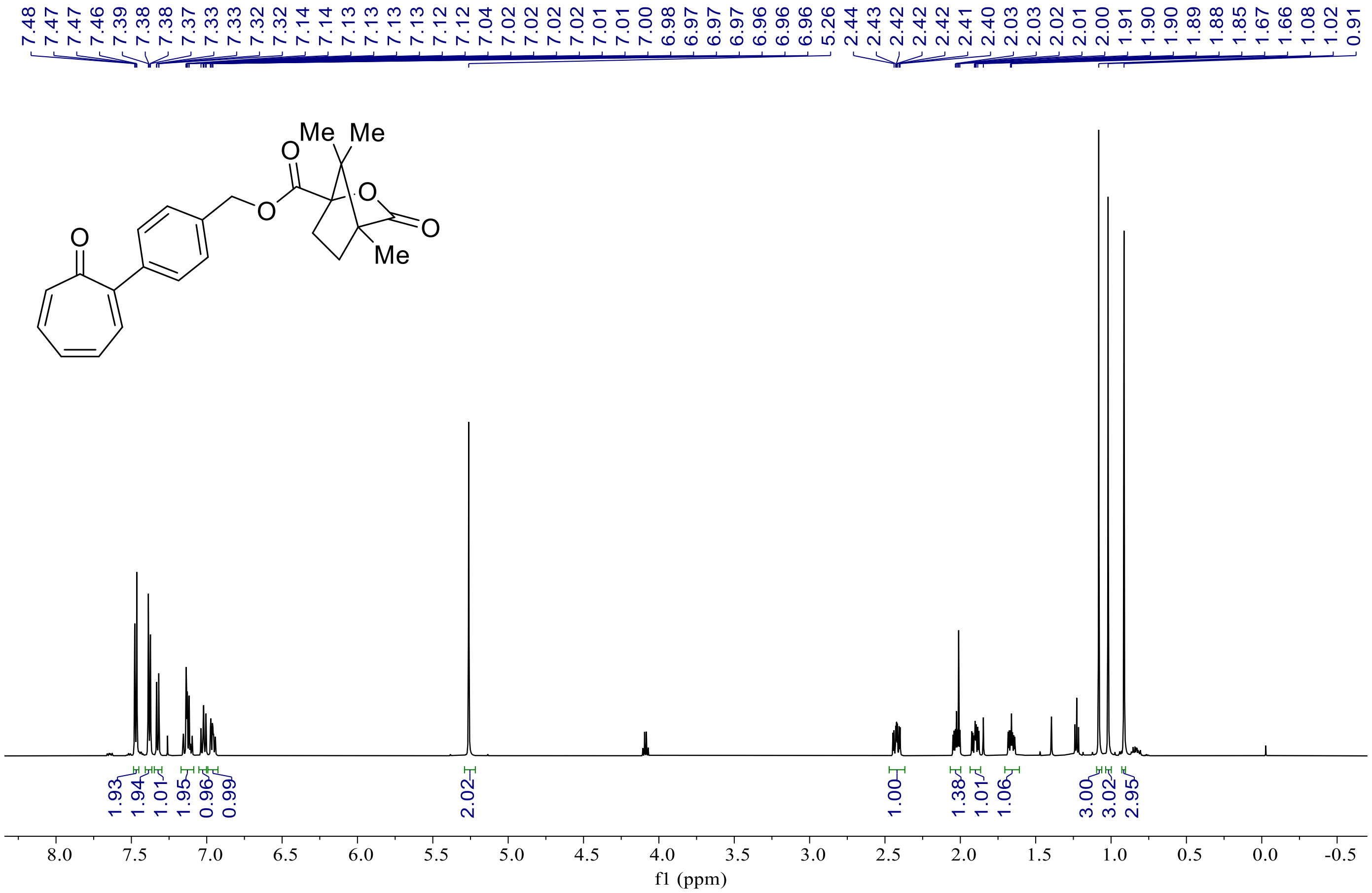

Compound 5s ^{13}C NMR (150 MHz, $CDCl_3$)

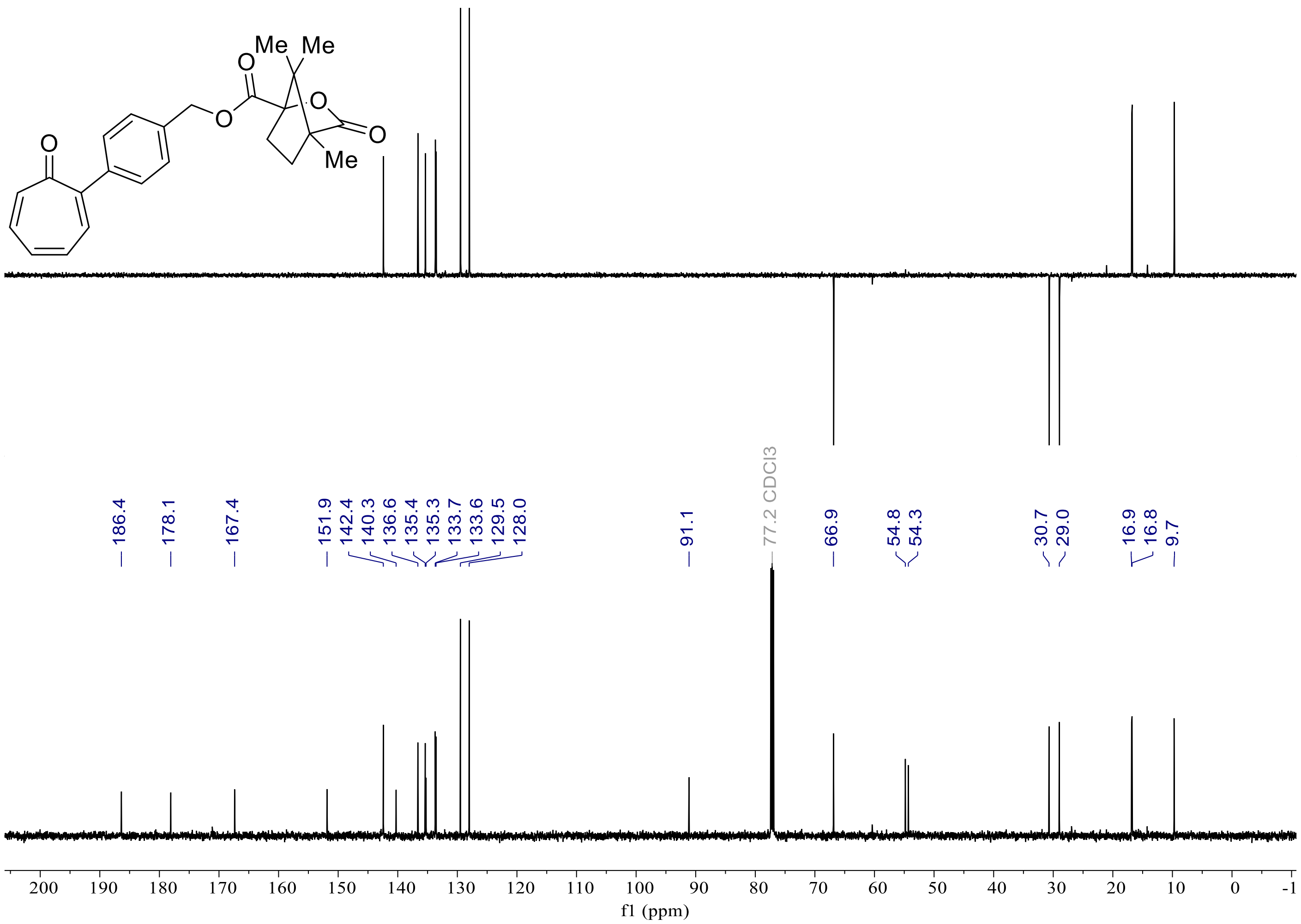

Compound 5s HRMS (ESI-TOF)

**Data Filename**	ESIH202405822.d	**Sample Name**	D4-ZDG3-3
**Sample ID**		**Position**	P1-E9
**Instrument Name**	Agilent 6520 Q-TOF	**Acq Method**	20160322_MS_ESIH_POS_1min.m
**Acquired Time**	12/6/2024 11:09:44 AM	**DA Method**	ESI-HR-20231114.m
**Comment**	ESIH by fangsu		

User Spectra

Fragmentor Voltage	Collision Energy	Ionization Mode
170	0	ESI

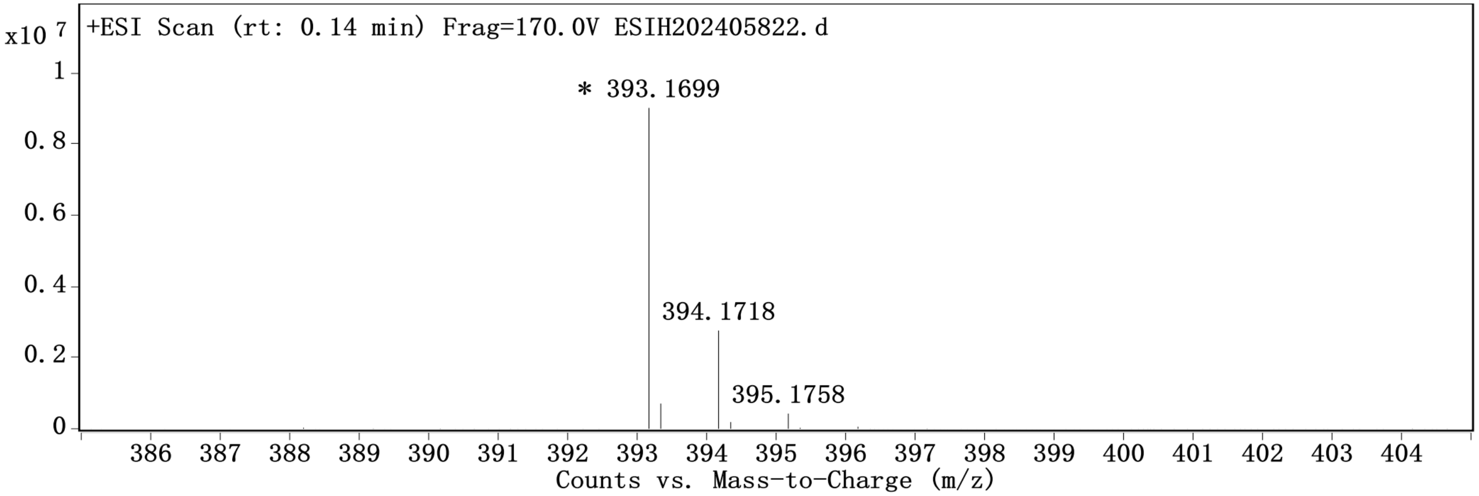

Formula Calculator Results

m/z	Calc m/z	Diff (mDa)	Diff (ppm)	Ion Formula	Ion
393.1699	393.1697	-0.28	-0.7	C24 H25 O5	(M+H)+

--- End Of Report ---

Compound 5t ^{1}H NMR (600 MHz, $CDCl_3$)

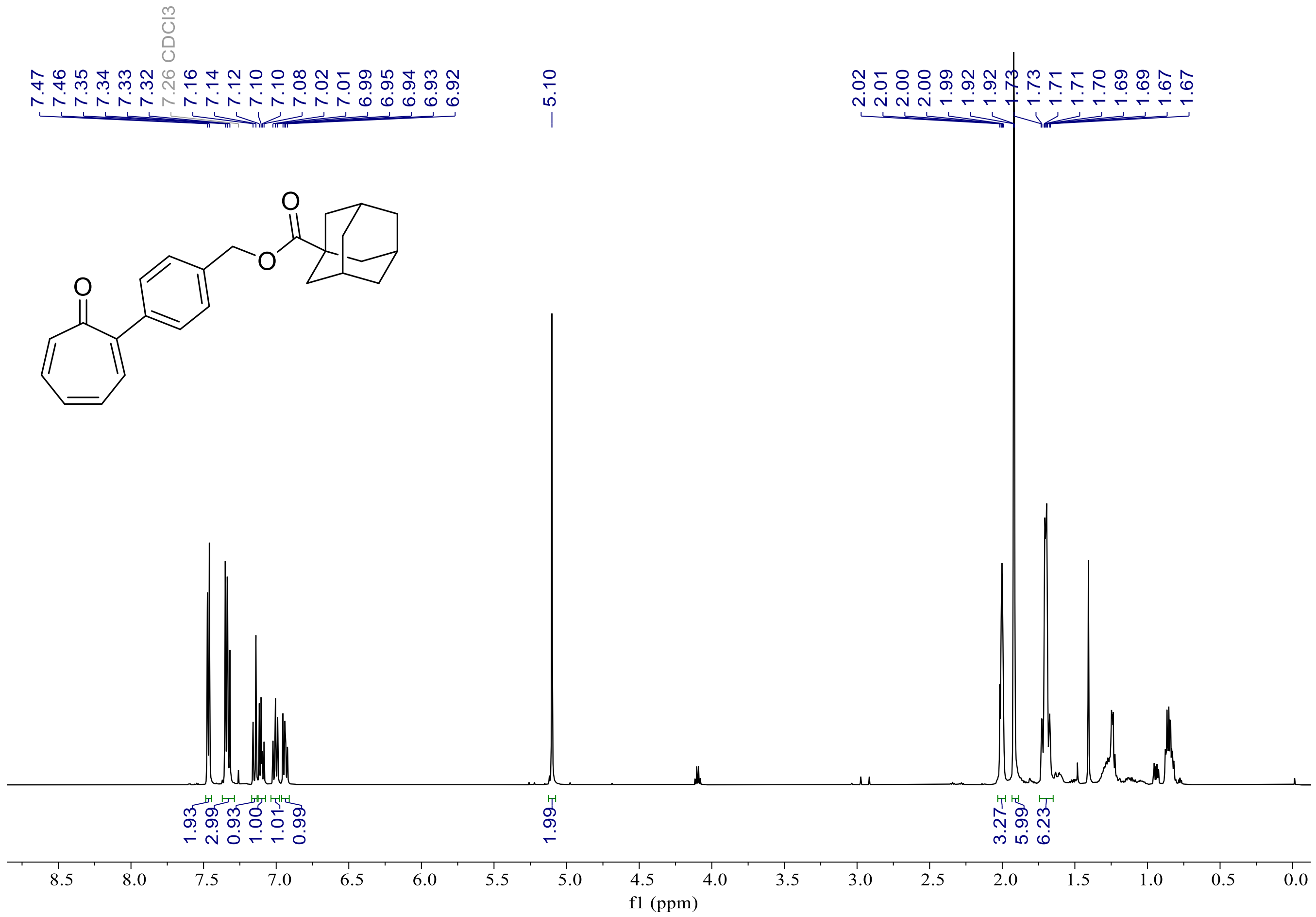

Compound 5t ^{13}C NMR (150 MHz, $CDCl_3$)

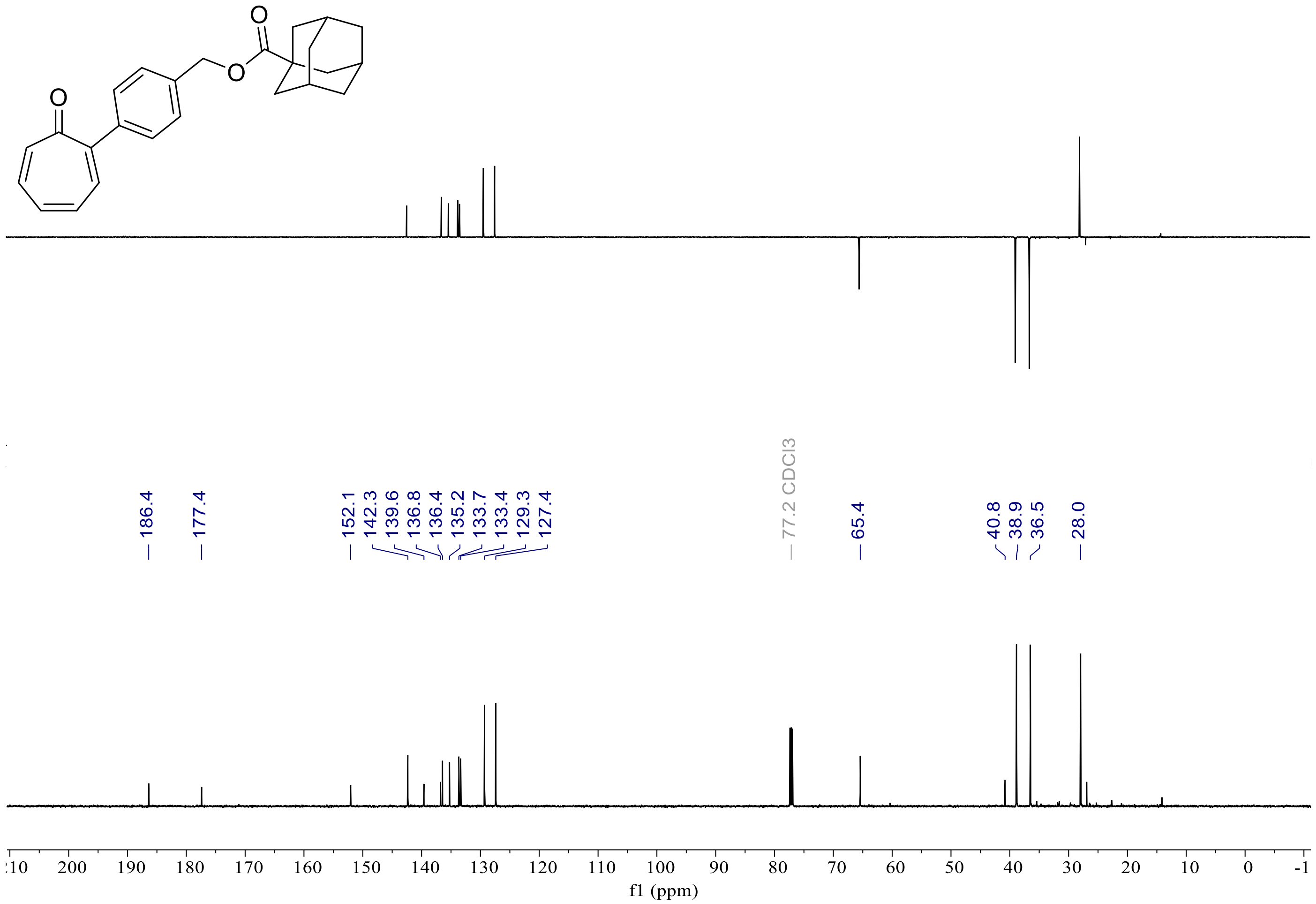

Compound 5t HRMS (ESI-TOF)

**Data Filename**	ESIH202405845.d	**Sample Name**	D4-ZDG3-4
**Sample ID**		**Position**	P1-A8
**Instrument Name**	Agilent 6520 Q-TOF	**Acq Method**	20160322_MS_ESIH_POS_1min.m
**Acquired Time**	12/9/2024 2:56:53 PM	**DA Method**	ESI-HR-20231114.m
**Comment**	ESIH by huangqiongping		

User Spectra

Fragmentor Voltage	Collision Energy	Ionization Mode
170	0	ESI

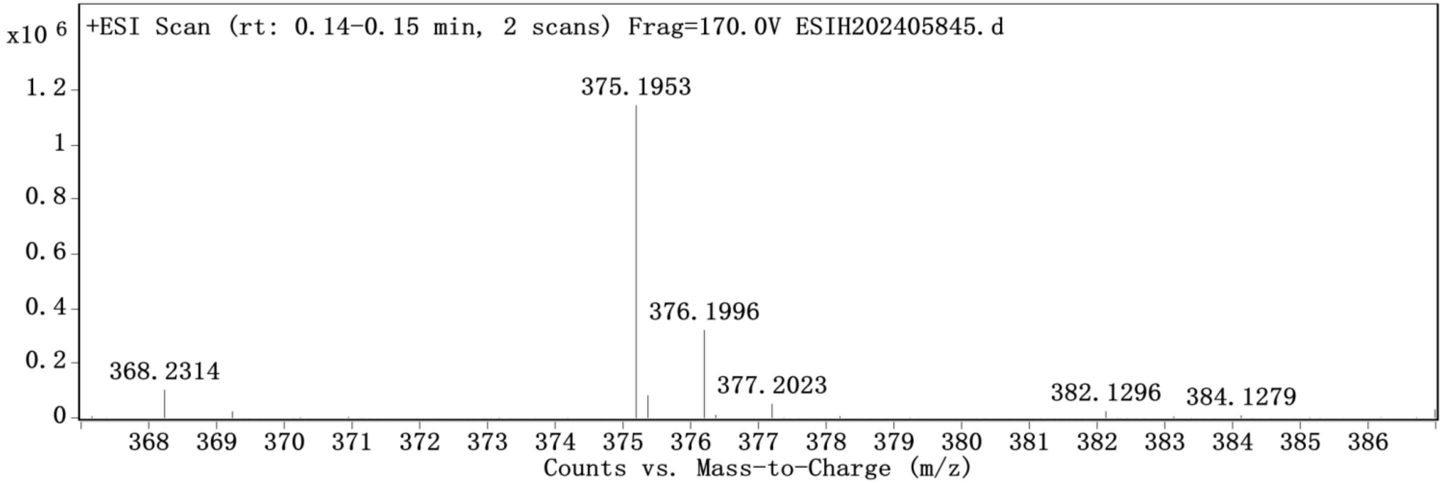

Formula Calculator Results

m/z	Calc m/z	Diff (mDa)	Diff (ppm)	Ion Formula	Ion
375.1953	375.1955	0.19	0.5	C25 H27 O3	(M+H)+

--- End Of Report ---

Compound 5u ^{1}H NMR (600 MHz, $CDCl_3$)

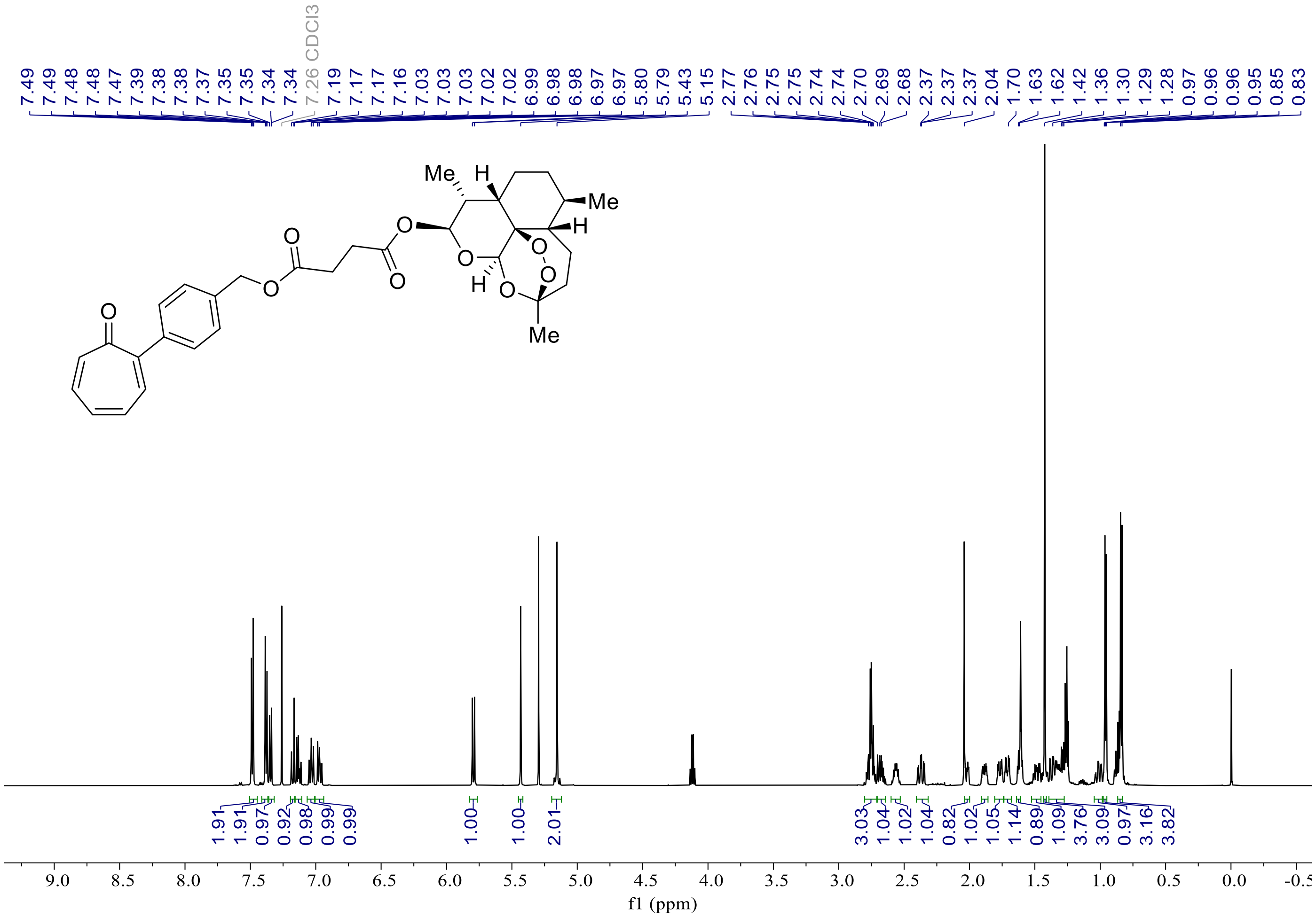

Compound 5u ^{13}C NMR (150 MHz, $CDCl_3$)

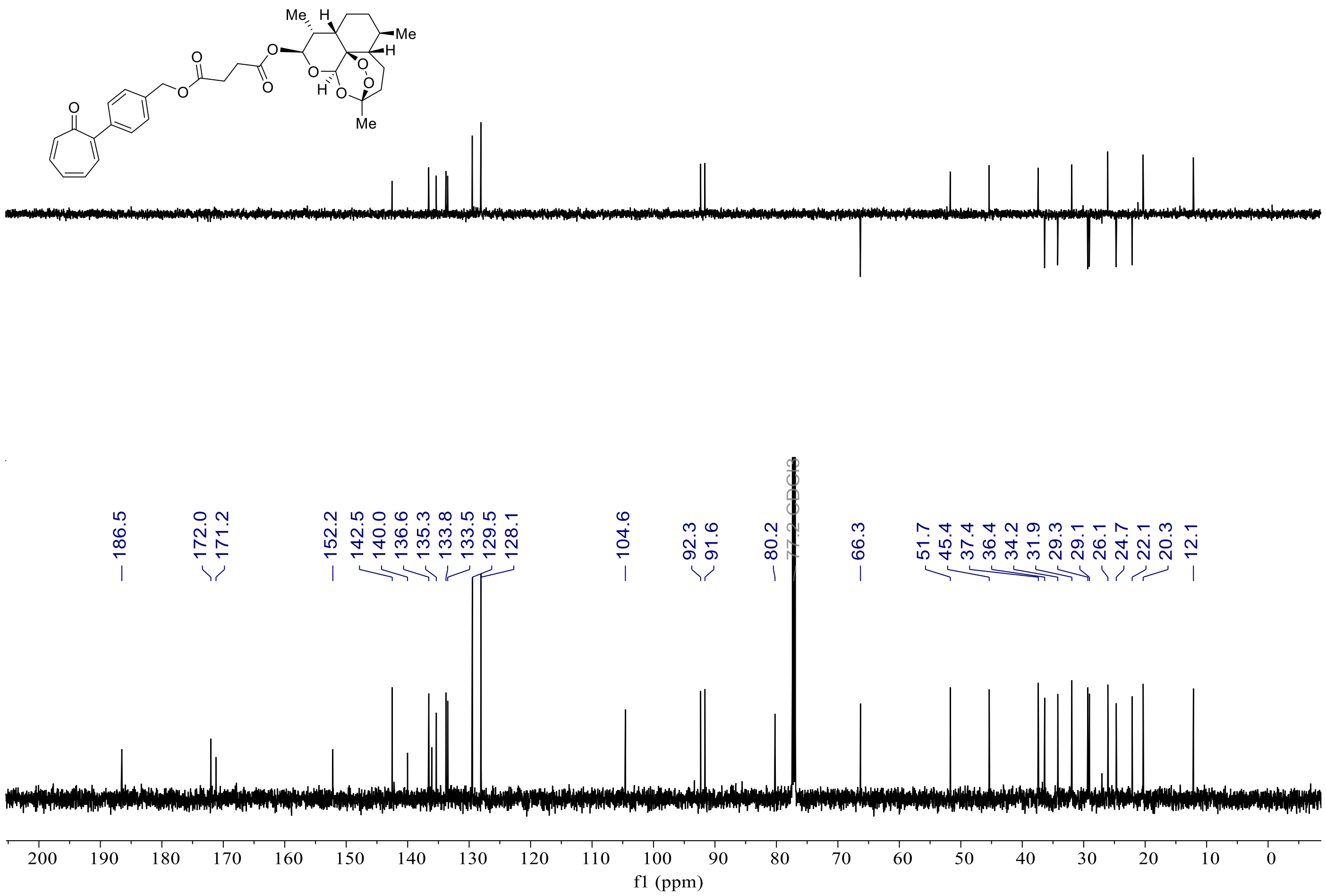

Compound 5u HRMS (ESI-TOF)

**Data Filename**	ESIH202405847.d	**Sample Name**	D4-ZDG3-5
**Sample ID**		**Position**	P1-B1
**Instrument Name**	Agilent 6520 Q-TOF	**Acq Method**	20160322_MS_ESIH_POS_1min.m
**Acquired Time**	12/9/2024 2:59:25 PM	**DA Method**	ESI-HR-20231114.m
**Comment**	ESIH by huangqiongping		

User Spectra

Fragmentor Voltage	Collision Energy	Ionization Mode
170	0	ESI

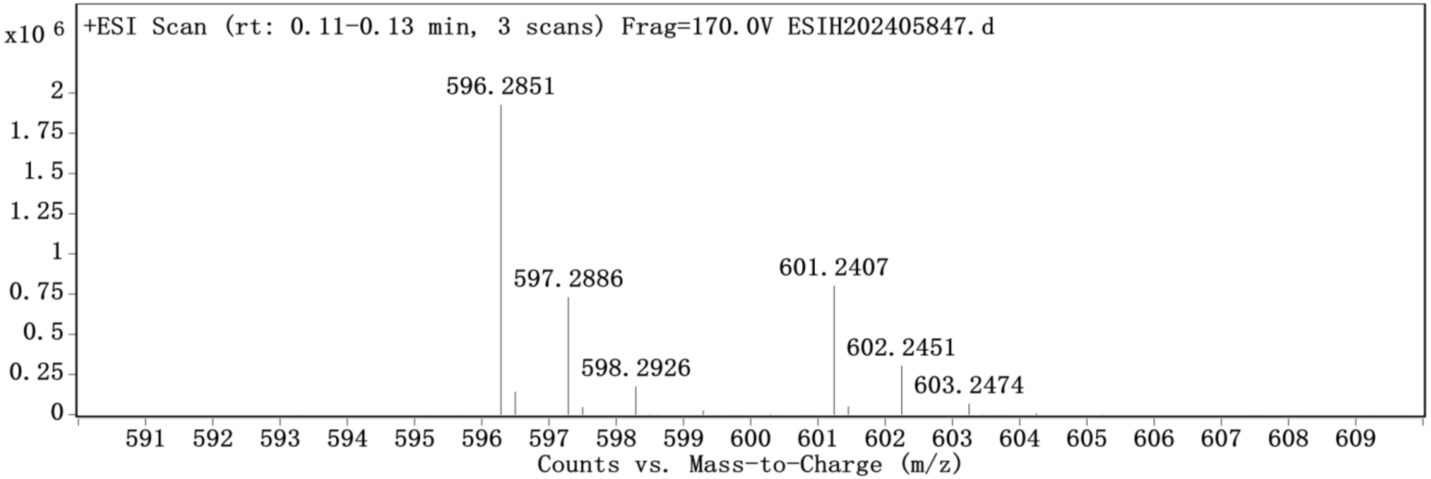

Formula Calculator Results

m/z	Calc m/z	Diff (mDa)	Diff (ppm)	Ion Formula	Ion
601.2407	601.2408	0.06	0.1	C33 H38 Na O9	(M+Na)+
596.2851	596.2854	0.33	0.56	C33 H42 N O9	(M+NH4)+

--- End Of Report ---

Compound 5v ^{1}H NMR (600 MHz, $CDCl_3$)

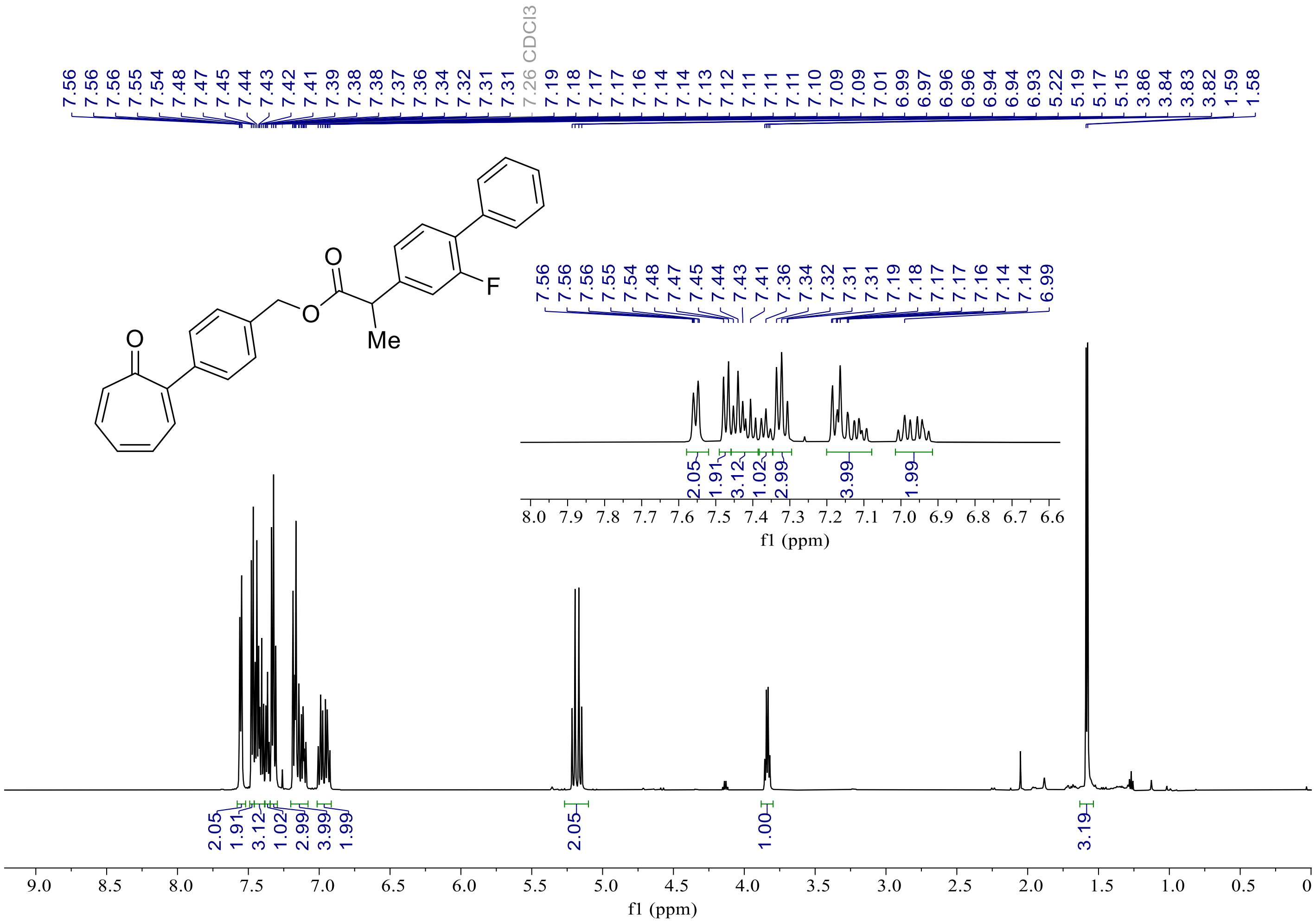

Compound 5v ^{13}C NMR (150 MHz, $CDCl_3$)

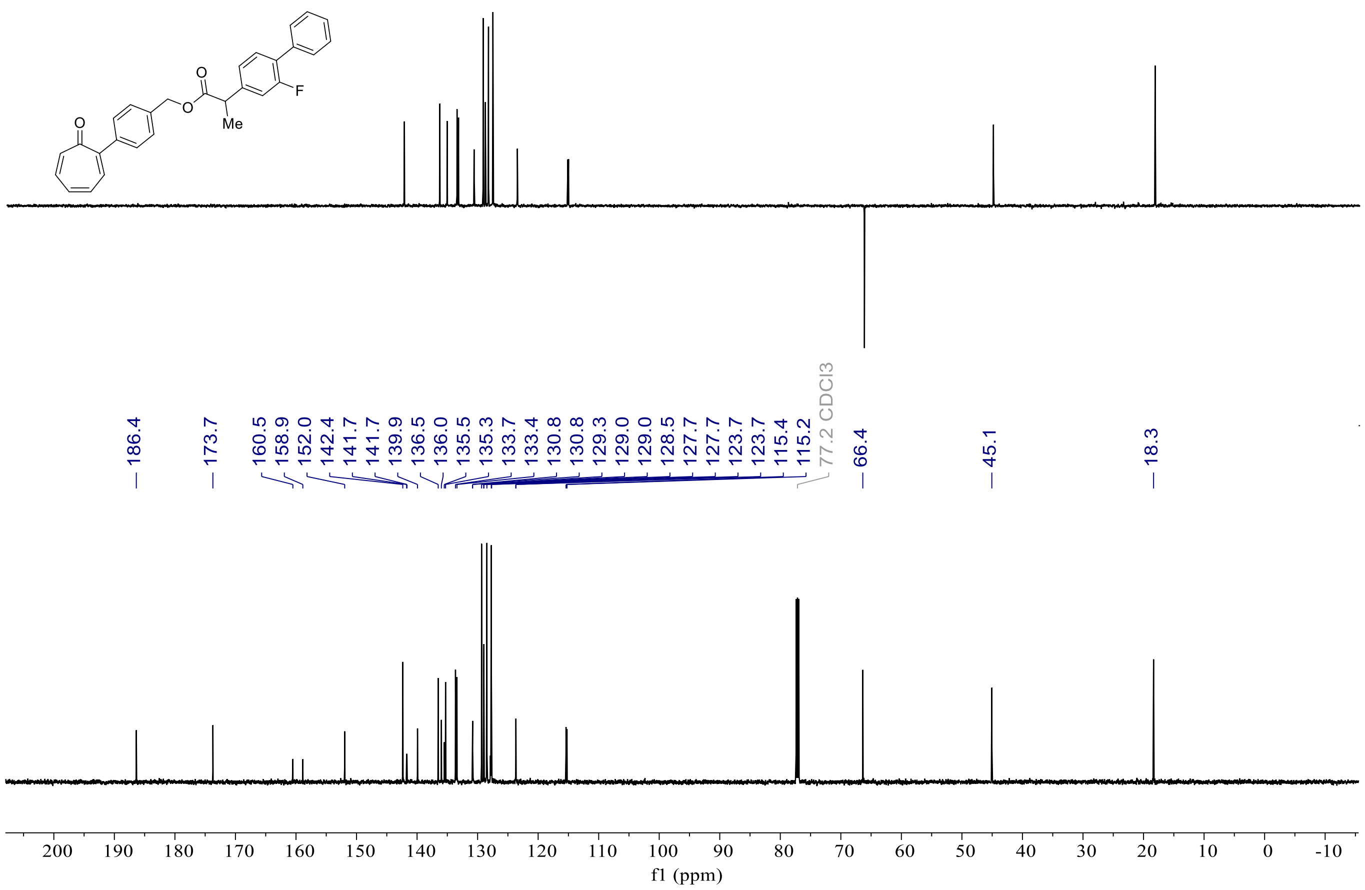

Compound 5v HRMS (ESI-TOF)

**Data Filename**	ESIH202406116.d	**Sample Name**	D4-D4-ZDG3-9
**Sample ID**		**Position**	P1-B6
**Instrument Name**	Agilent 6520 Q-TOF	**Acq Method**	20160322_MS_ESIH_POS_1min.m
**Acquired Time**	12/25/2024 5:24:17 PM	**DA Method**	ESI-HR-20231114.m
**Comment**	ESIH by fangsu		

User Spectra

Fragmentor Voltage	Collision Energy	Ionization Mode
170	0	ESI

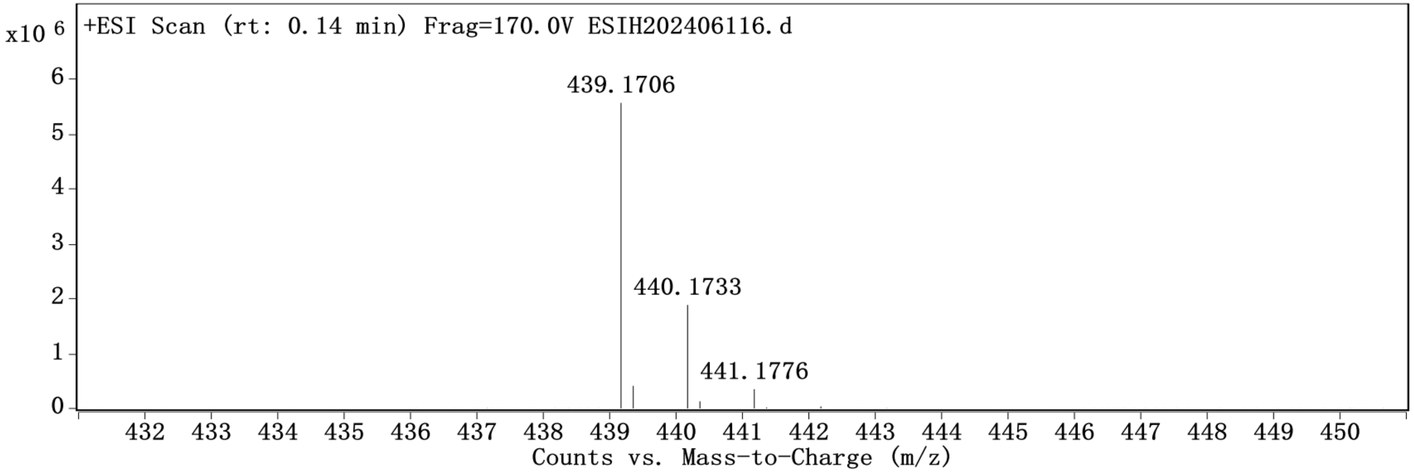

Formula Calculator Results

m/z	Calc m/z	Diff (mDa)	Diff (ppm)	Ion Formula	Ion
439.1706	439.1704	-0.18	-0.4	C29 H24 F O3	(M+H)+

--- End Of Report ---

Compound 9k ^{1}H NMR (600 MHz, CDCl$_3$)

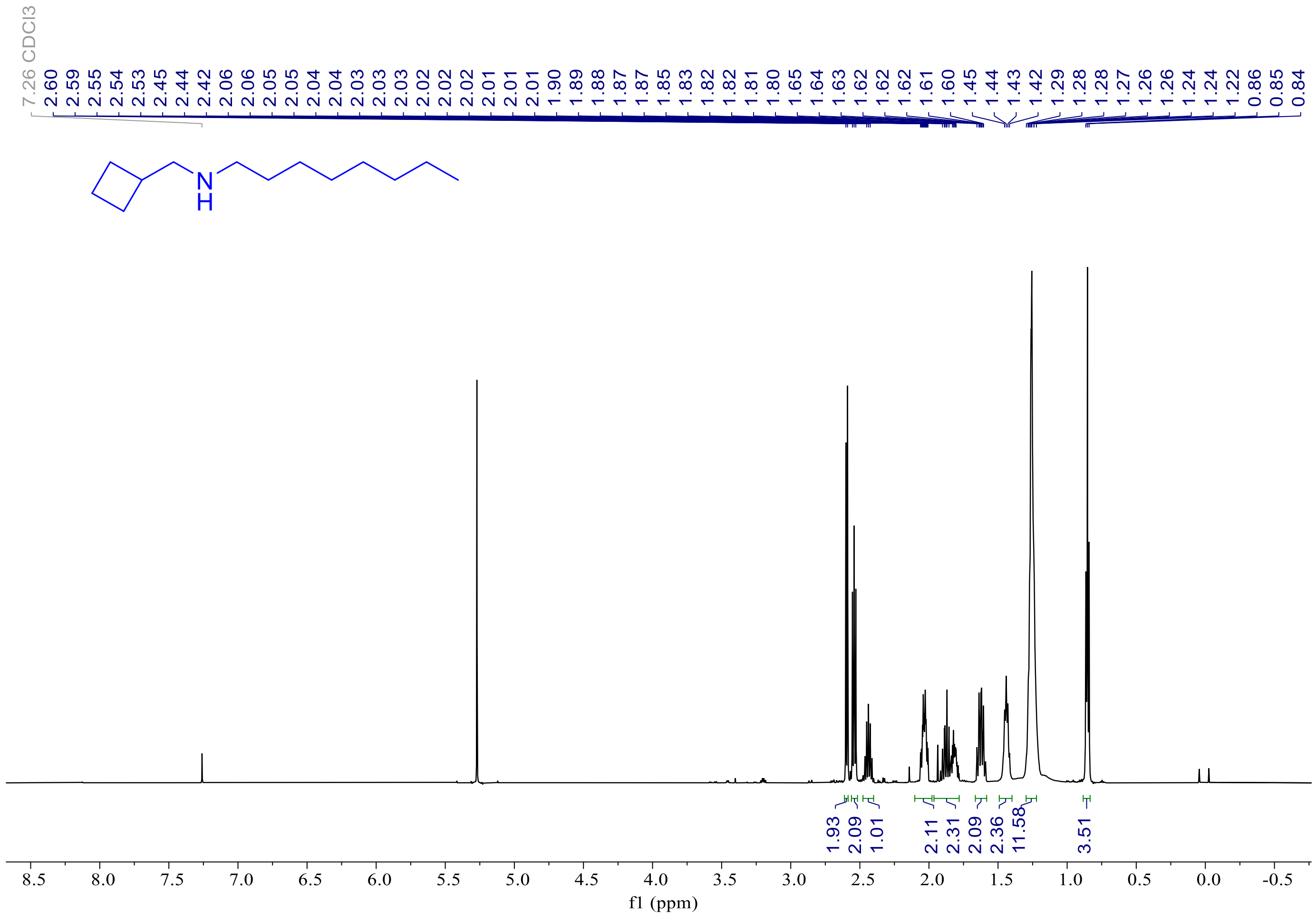

Compound 9k ^{13}C NMR (150 MHz, $CDCl_3$)

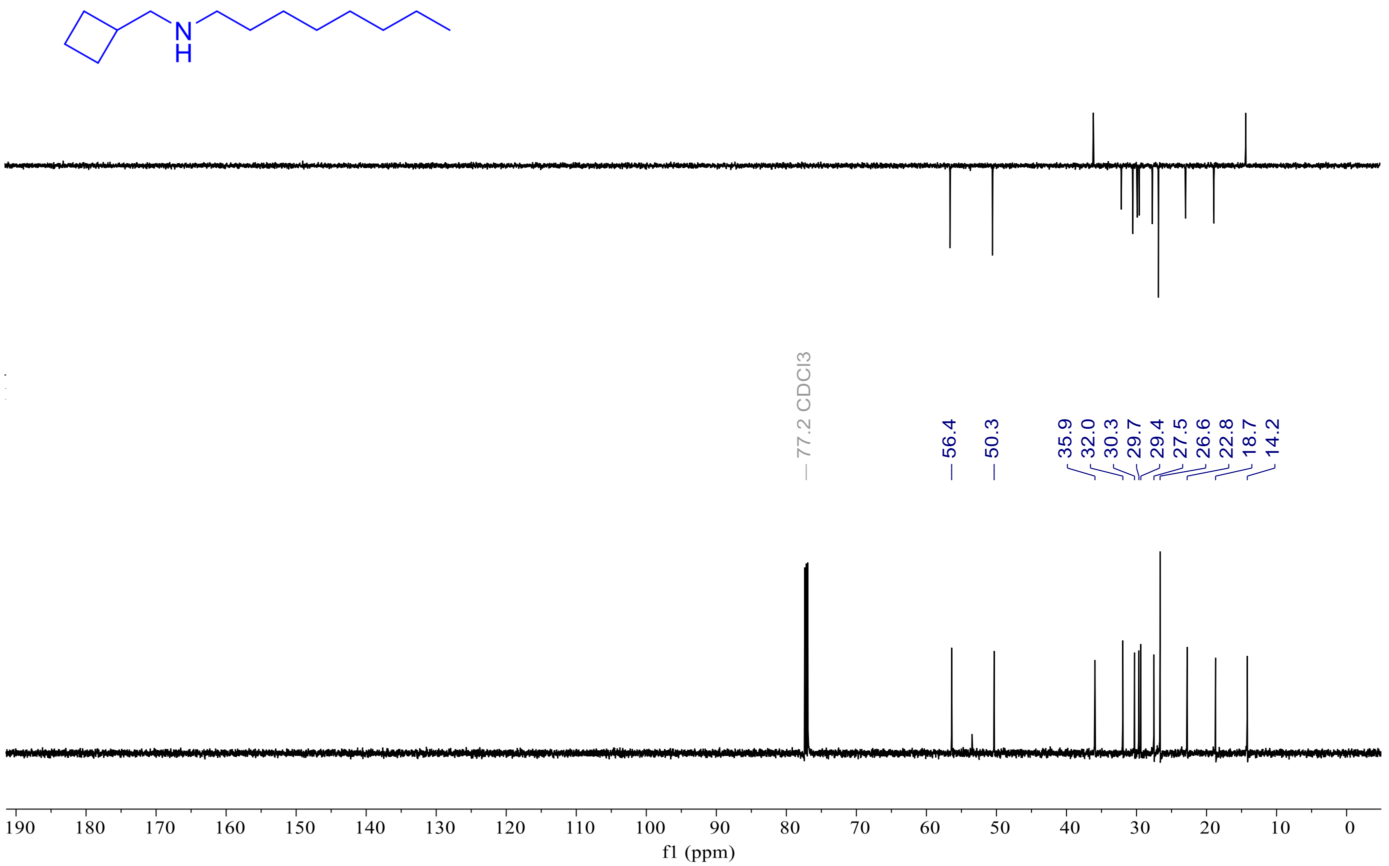

Compound 9k HRMS (ESI-TOF)

**Data Filename**	ESIH202405594.d	**Sample Name**	D4-D4-ZQSq3
**Sample ID**		**Position**	P1-A4
**Instrument Name**	Agilent 6520 Q-TOF	**Acq Method**	20160322_MS_ESIH_POS_1min.m
**Acquired Time**	11/28/2024 2:12:29 PM	**DA Method**	ESI-HR-20231114.m
**Comment**	ESIH by fangsu		

User Spectra

Fragmentor Voltage	Collision Energy	Ionization Mode
170	0	ESI

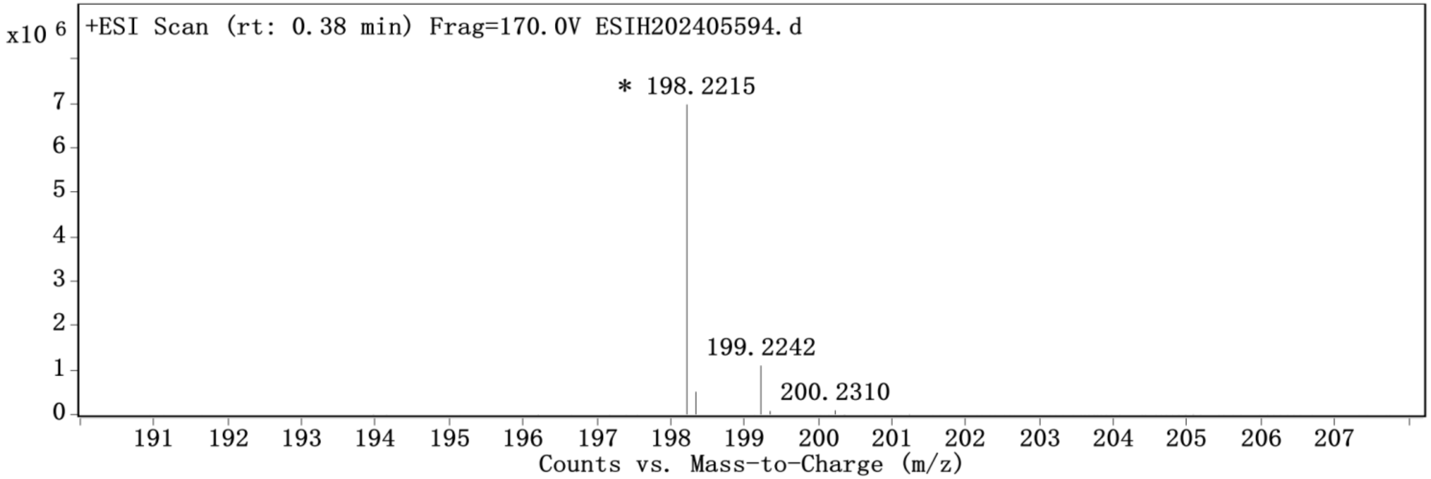

Formula Calculator Results

m/z	Calc m/z	Diff (mDa)	Diff (ppm)	Ion Formula	Ion
198.2215	198.2216	0.09	0.44	C13 H28 N	(M+H)+

--- End Of Report ---

Compound 9m ^{1}H NMR (600 MHz, $CDCl_3$)

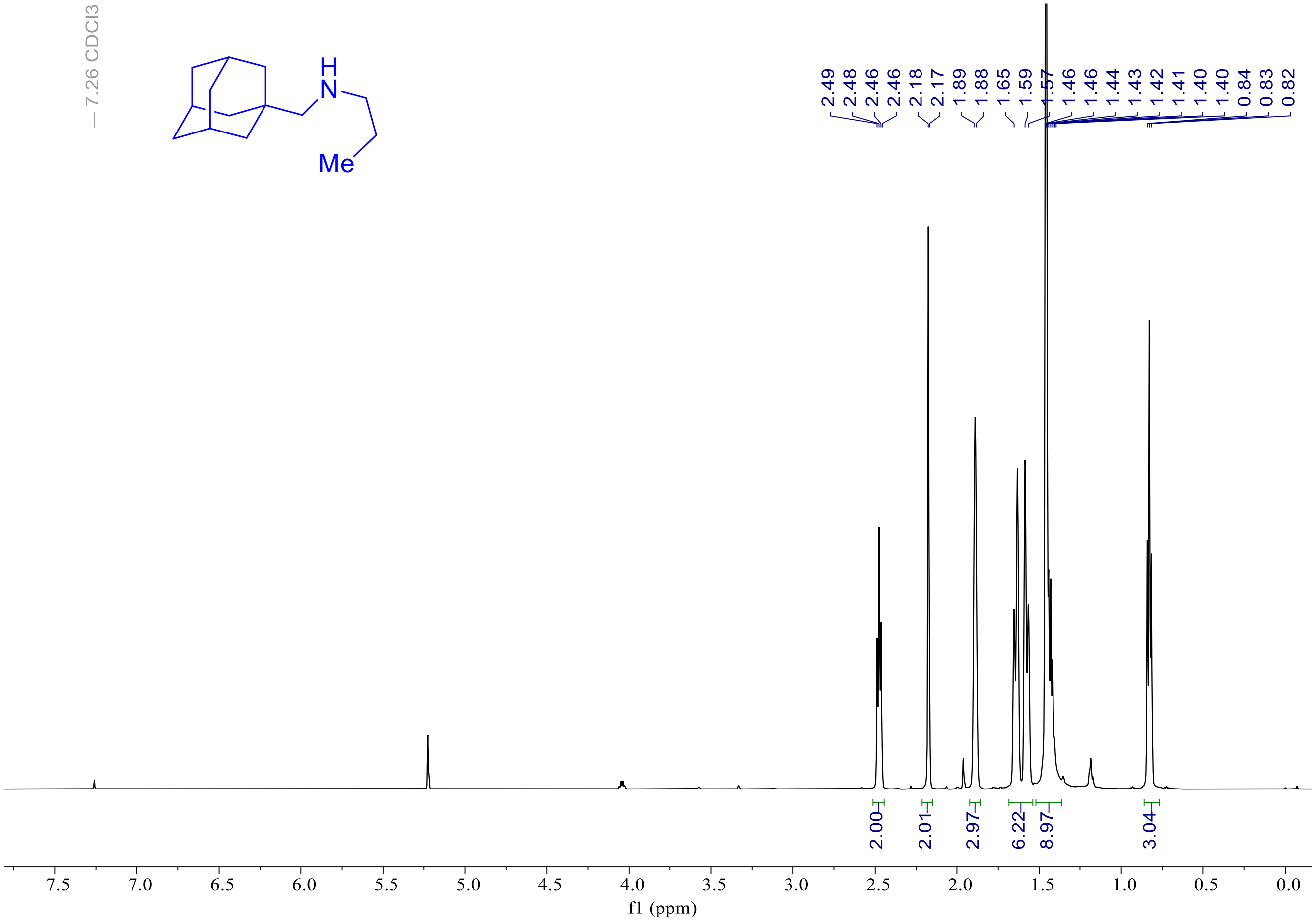

Compound 9m ^{13}C NMR (150 MHz, $CDCl_3$)

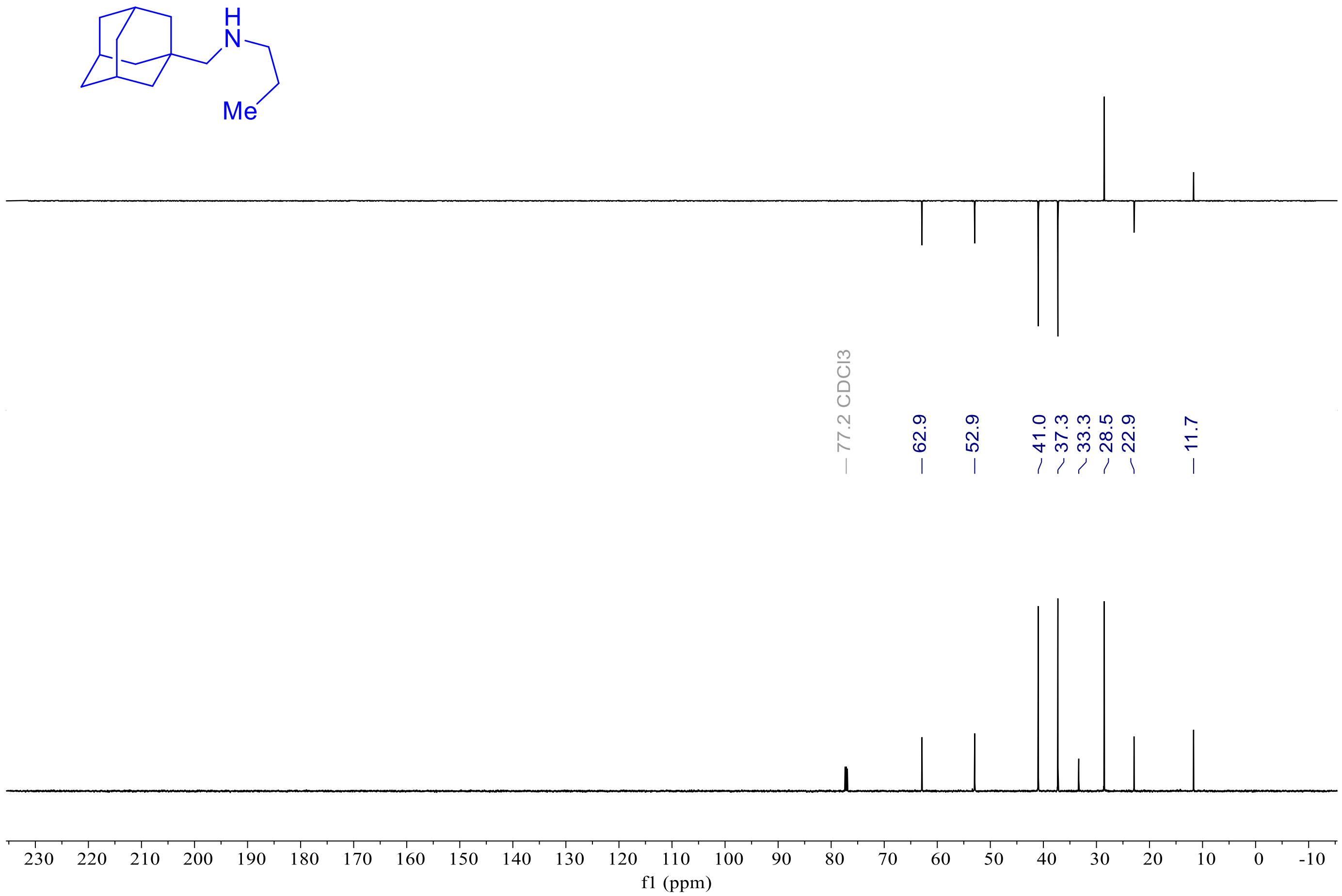

Compound 9m HRMS (ESI-TOF)

**Data Filename**	ESIH202405593.d	**Sample Name**	D4-D4-ZQAda5
**Sample ID**		**Position**	P1-A3
**Instrument Name**	Agilent 6520 Q-TOF	**Acq Method**	20160322_MS_ESIH_POS_1min.m
**Acquired Time**	11/28/2024 2:11:13 PM	**DA Method**	ESI-HR-20231114.m
**Comment**	ESIH by fangsu		

User Spectra

Fragmentor Voltage	Collision Energy	Ionization Mode
170	0	ESI

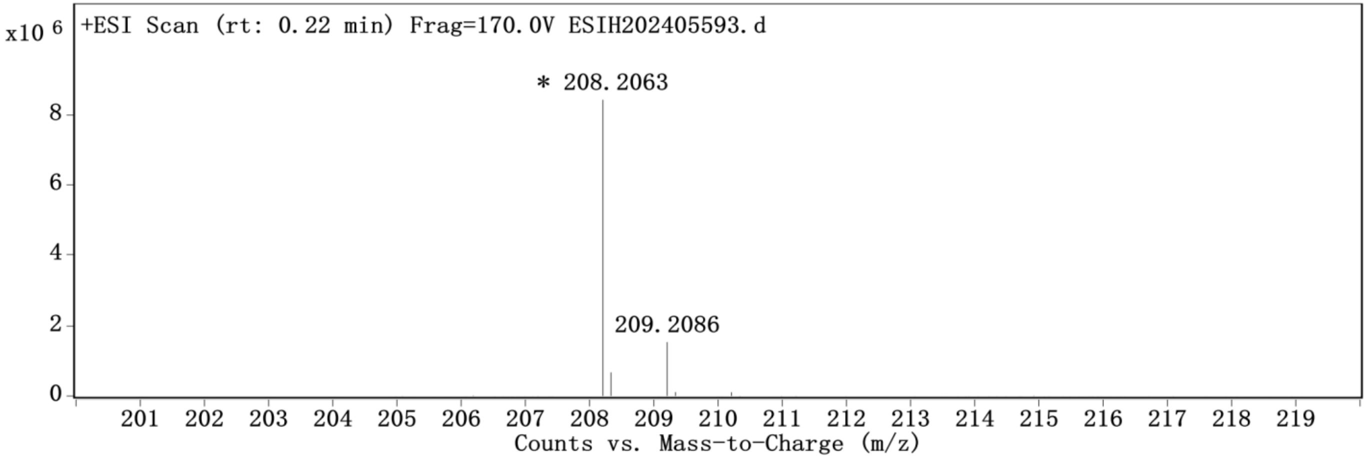

Formula Calculator Results

m/z	Calc m/z	Diff (mDa)	Diff (ppm)	Ion Formula	Ion
208.2063	208.206	-0.33	-1.6	C14 H26 N	(M+H)+

--- End Of Report ---

Compound 9n ^{1}H NMR (400 MHz, $CDCl_3$)

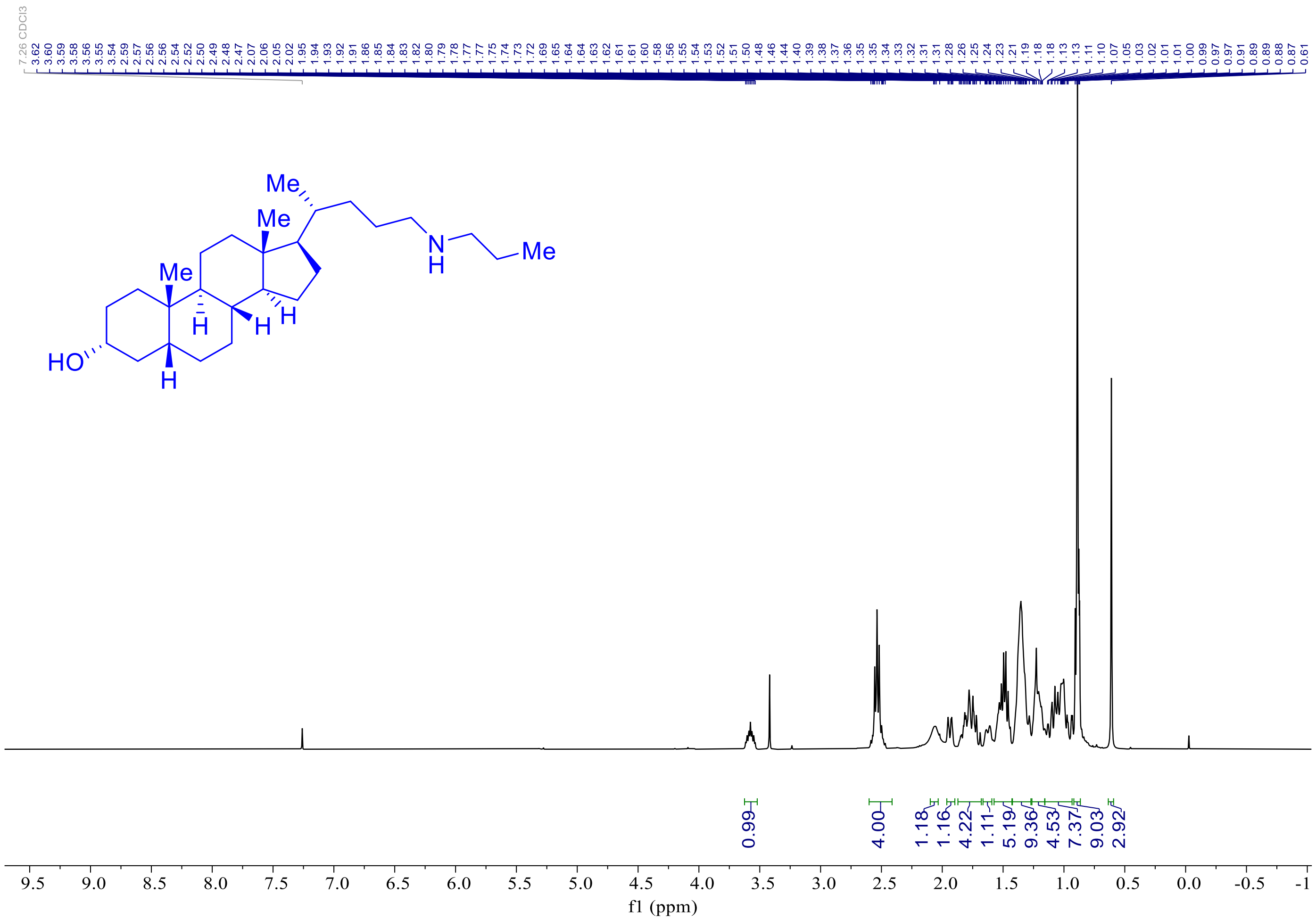

Compound 9n ^{13}C NMR (100 MHz, $CDCl_3$)

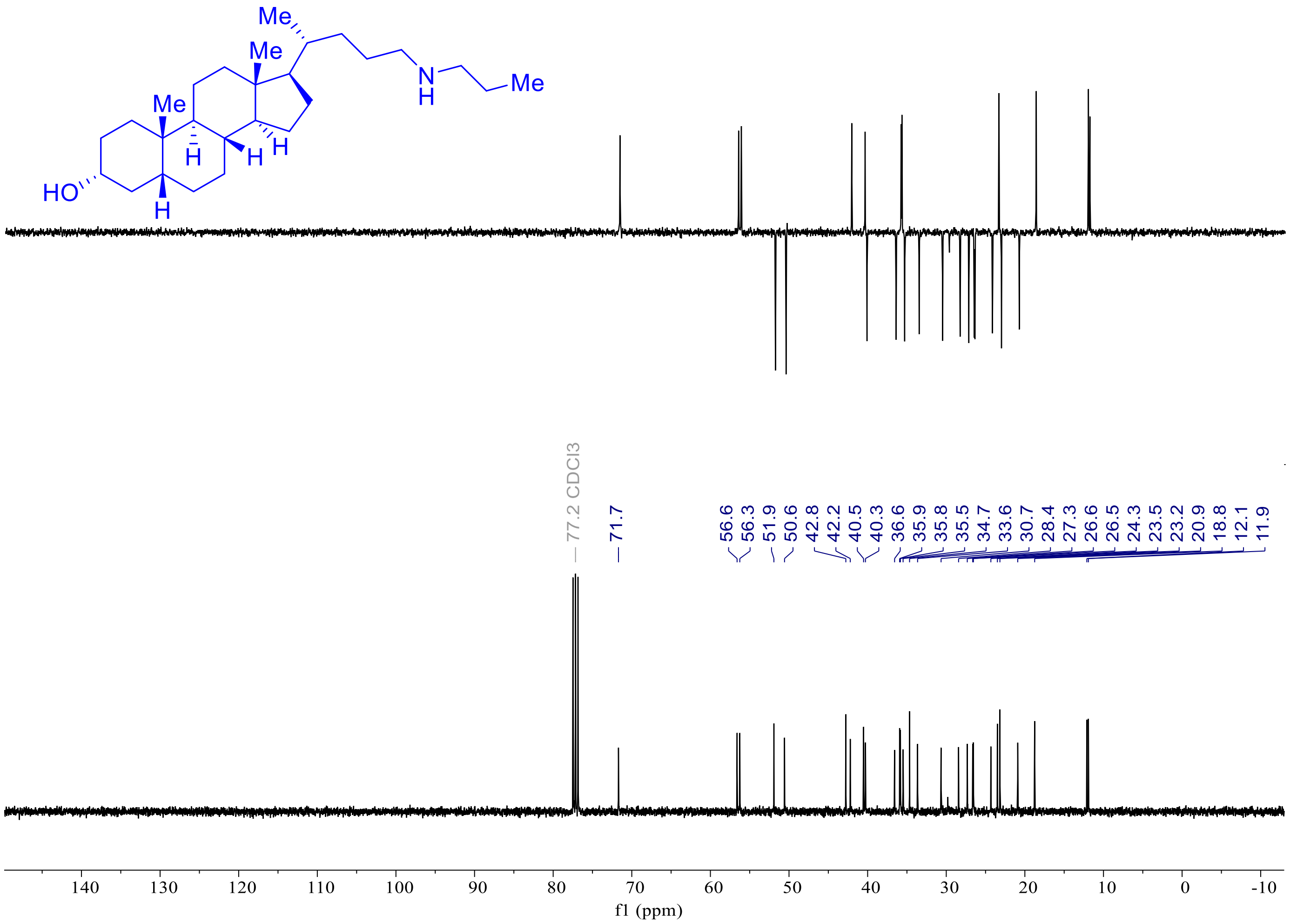

Compound 9n HRMS (ESI-TOF)

**Data Filename**	ESIH202405596.d	**Sample Name**	D4-D4-ZQSt4T2
**Sample ID**		**Position**	P1-A6
**Instrument Name**	Agilent 6520 Q-TOF	**Acq Method**	20160322_MS_ESIH_POS_1min.m
**Acquired Time**	11/28/2024 2:14:59 PM	**DA Method**	ESI-HR-20231114.m
**Comment**	ESIH by fangsu		

User Spectra

Fragmentor Voltage	Collision Energy	Ionization Mode
170	0	ESI

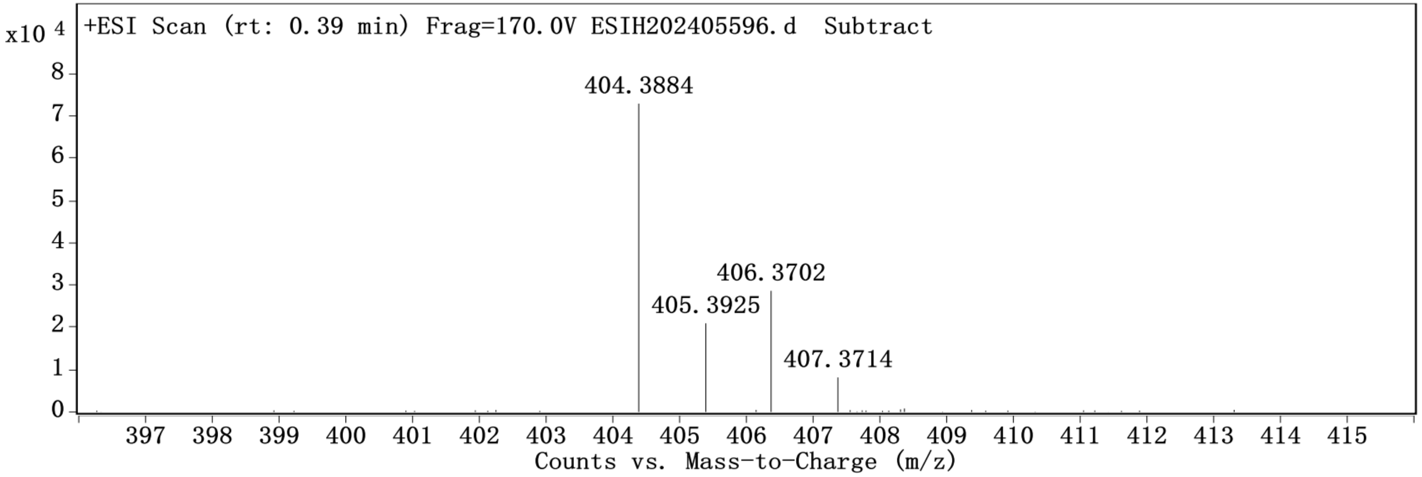

Formula Calculator Results

m/z	Calc m/z	Diff (mDa)	Diff (ppm)	Ion Formula	Ion
404.3884	404.3887	0.33	0.82	C27 H50 N O	(M+H)+

--- End Of Report ---

Compound 7a ^{1}H NMR (400 MHz, $CDCl_3$)

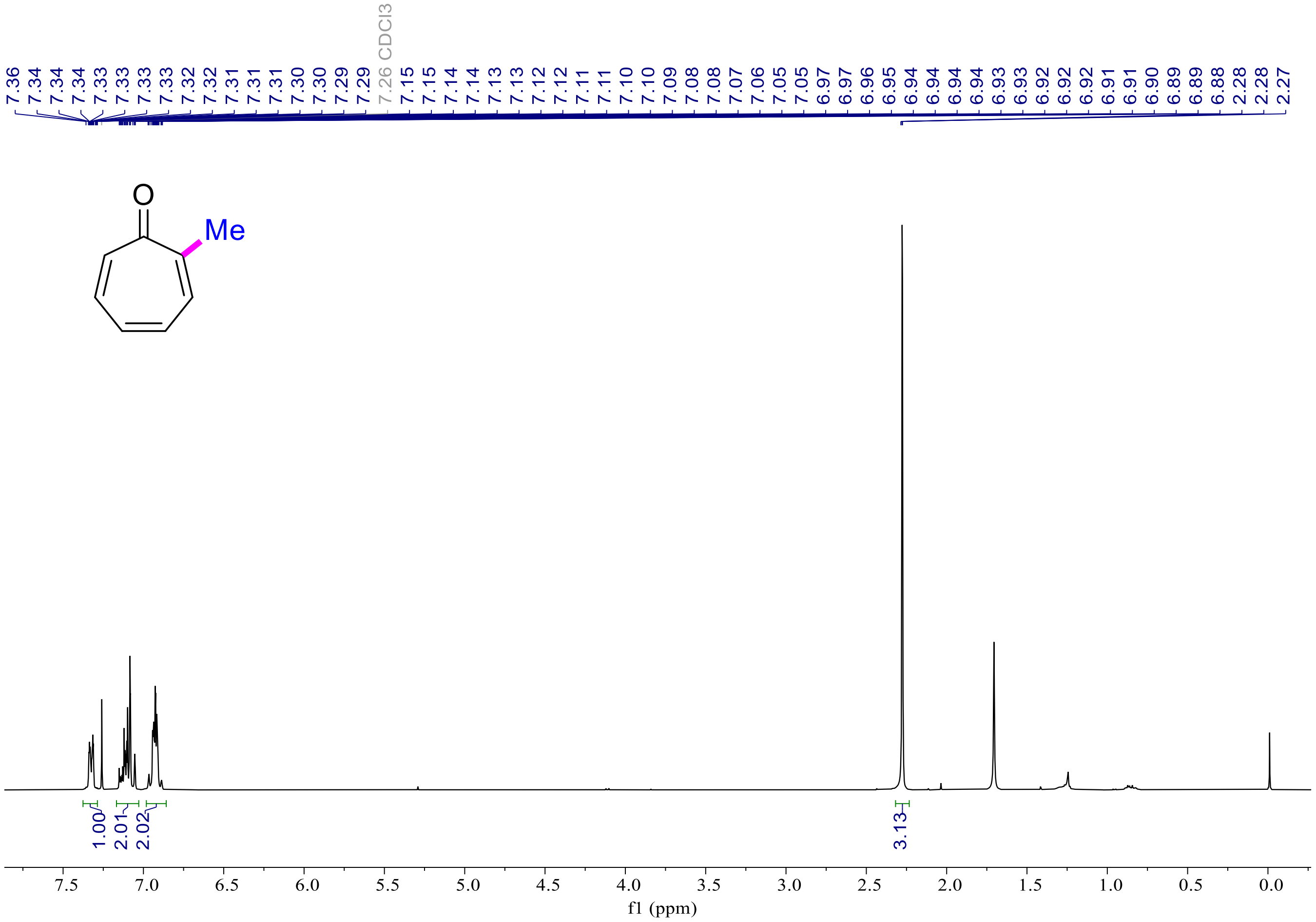

Compound 7a ^{13}C NMR (125 MHz, $CDCl_3$)

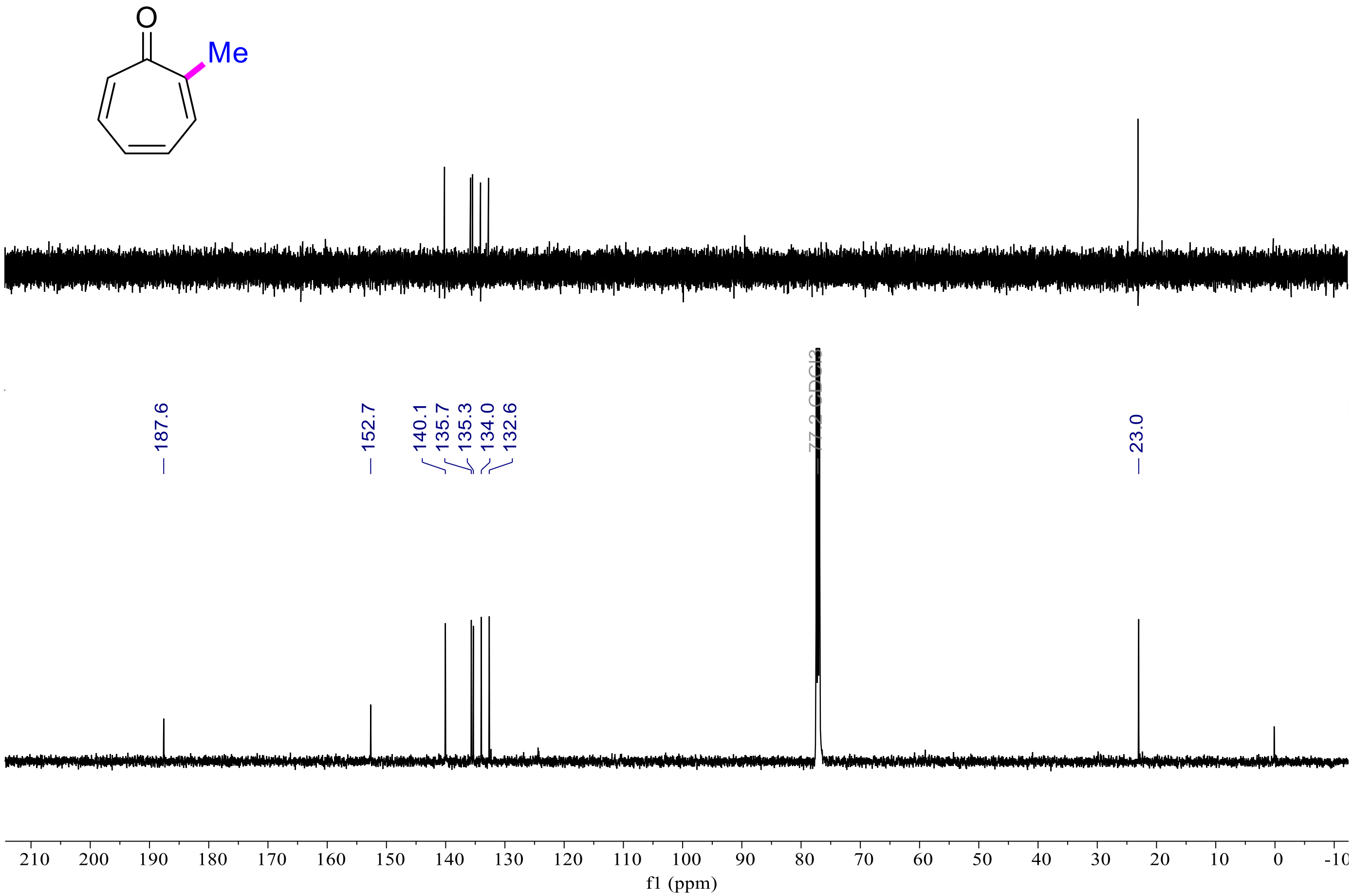

Compound 7a HRMS (ESI-TOF):

**Data Filename**	ESIH202403990-1.d	**Sample Name**	D4-ZQT14-6A
**Sample ID**		**Position**	P1-E1
**Instrument Name**	Agilent 6520 Q-TOF	**Acq Method**	20160322_MS_ESIH_POS_1min.m
**Acquired Time**	8/14/2024 4:05:09 PM	**DA Method**	ESI-HR-20231114.m
**Comment**	ESIH by fangsu		

User Spectra

Fragmentor Voltage	Collision Energy	Ionization Mode
170	0	ESI

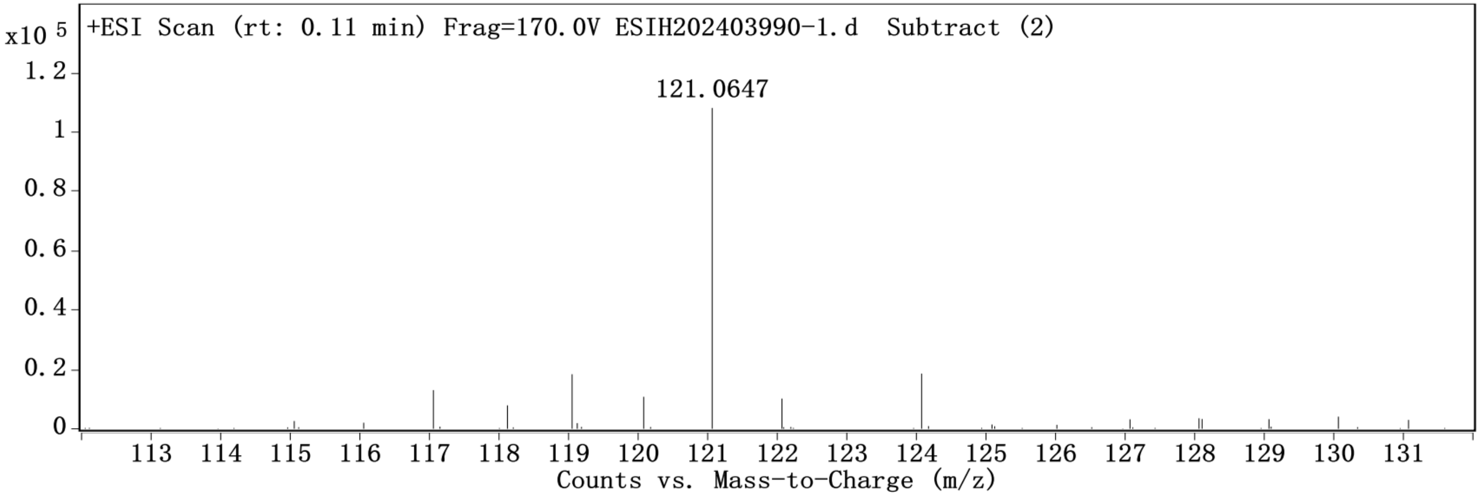

Formula Calculator Results

m/z	Calc m/z	Diff (mDa)	Diff (ppm)	Ion Formula	Ion
121.0647	121.0648	0.08	0.69	C8 H9 O	(M+H)+

--- End Of Report ---

Compound 7c ^{1}H NMR (600 MHz, $CDCl_3$)

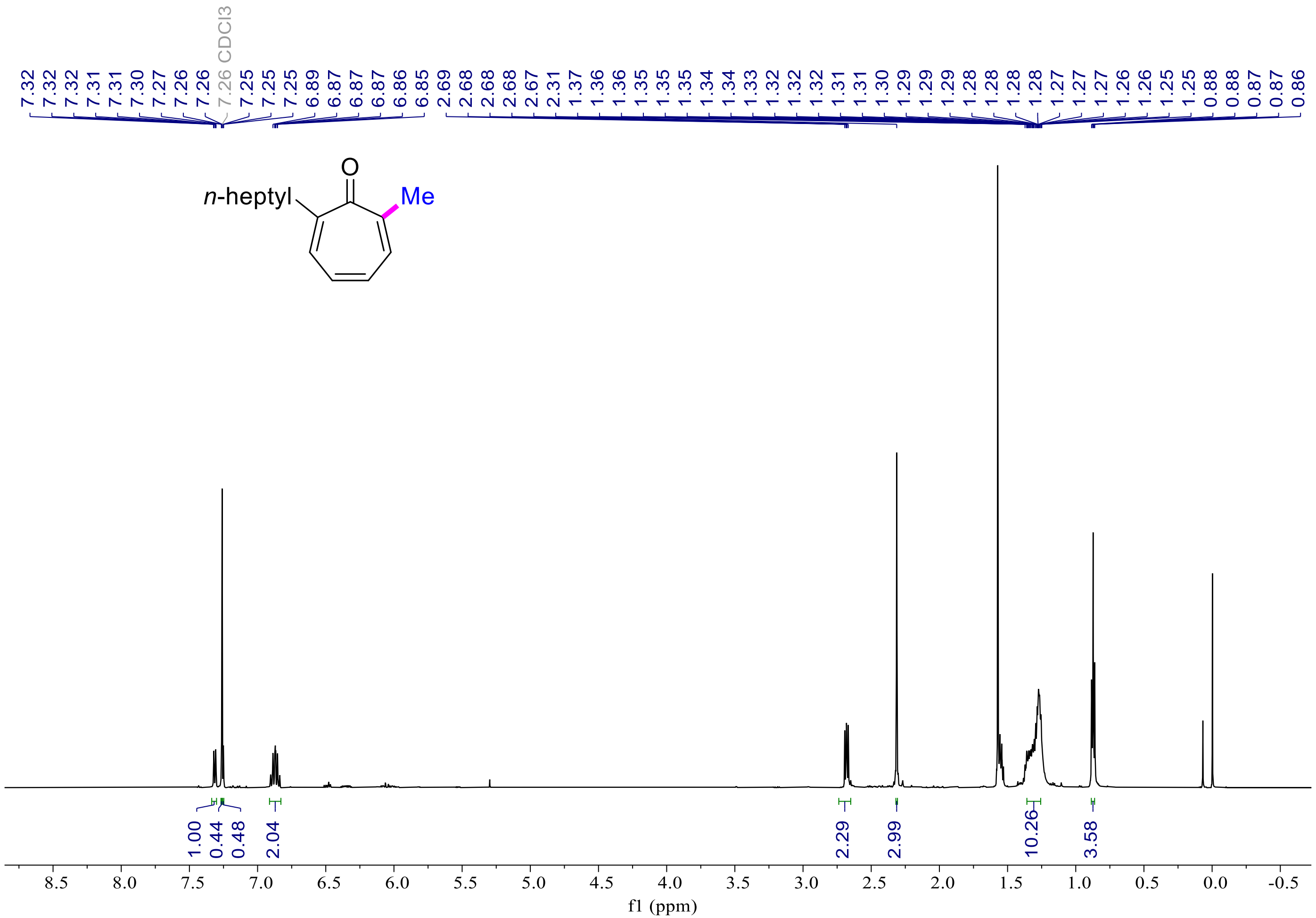

Compound 7c ^{13}C NMR (125 MHz, CDCl$_3$)

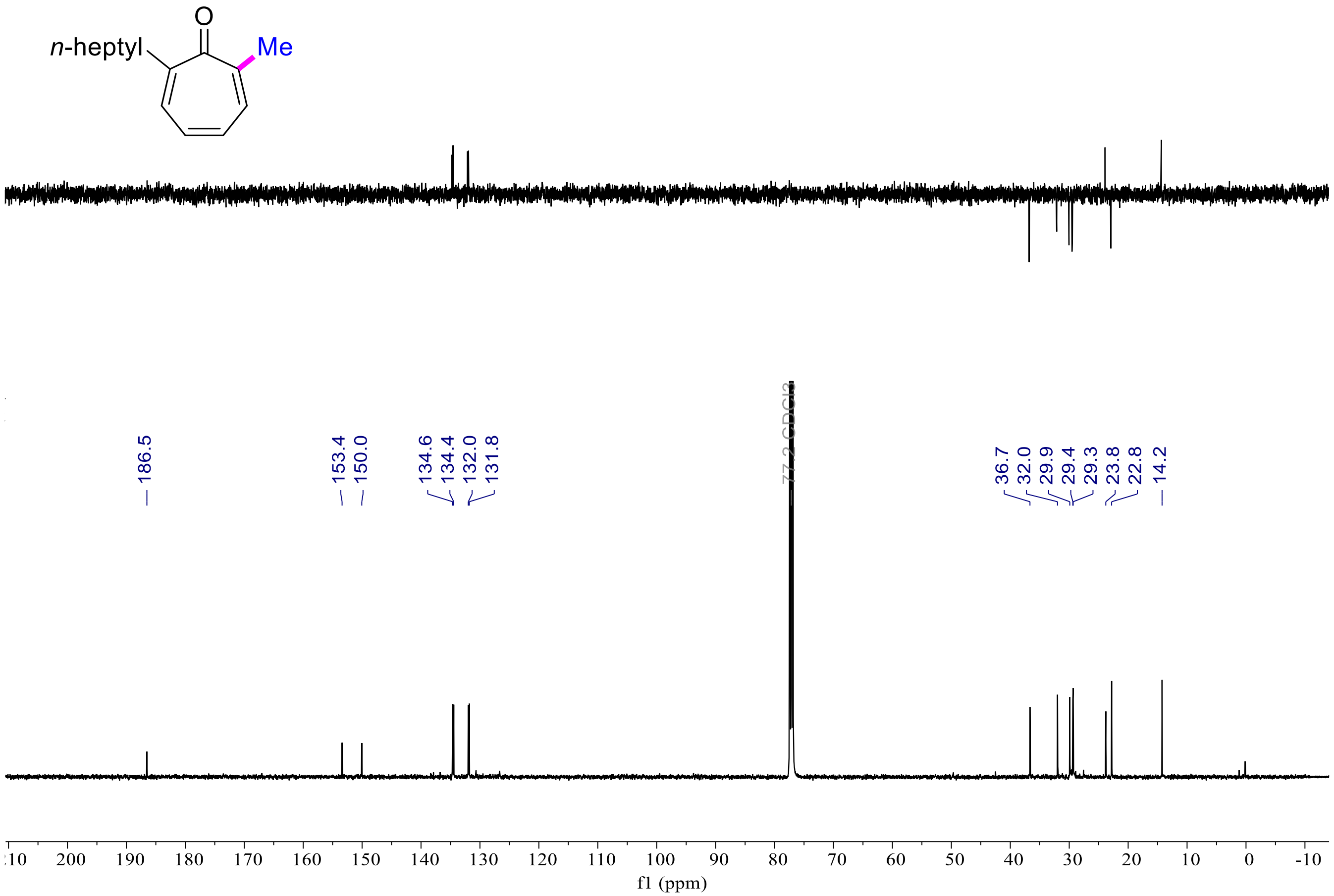

Compound 7c HRMS (ESI-TOF)

**Data Filename**	ESIH202404429.d	**Sample Name**	D4-ZQT18-27
**Sample ID**		**Position**	P1-A9
**Instrument Name**	Agilent 6520 Q-TOF	**Acq Method**	20160322_MS_ESIH_POS_1min.m
**Acquired Time**	9/11/2024 2:53:53 PM	**DA Method**	ESI-HR-20231114.m
**Comment**	ESIH by fangsu		

User Spectra

Fragmentor Voltage	Collision Energy	Ionization Mode
170	0	ESI

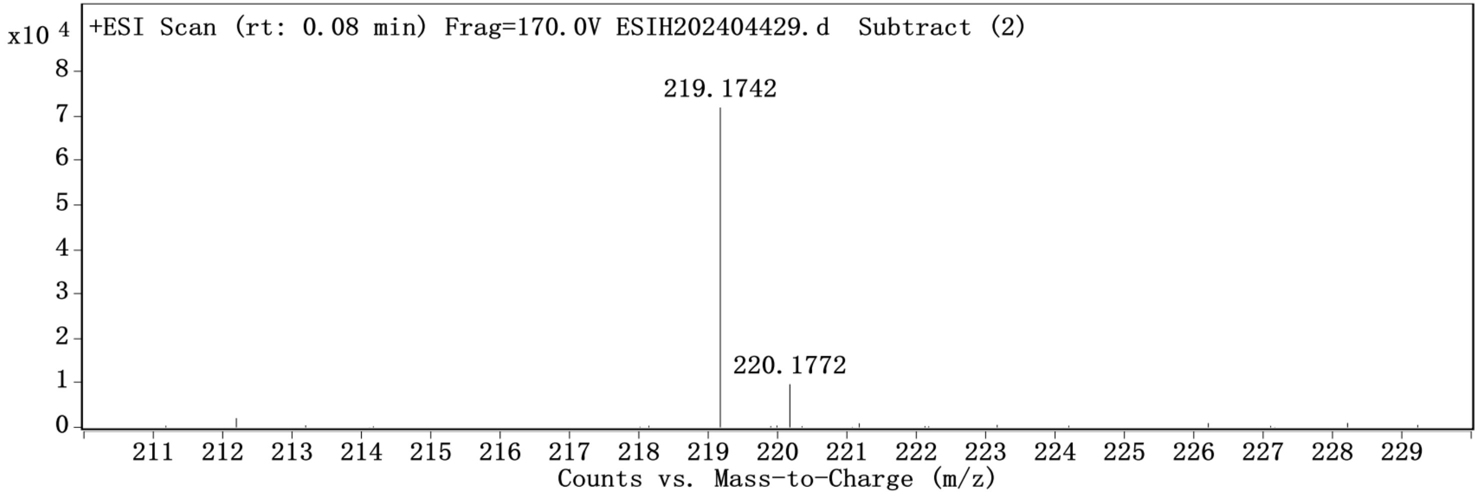

Formula Calculator Results

m/z	Calc m/z	Diff (mDa)	Diff (ppm)	Ion Formula	Ion
219.1742	219.1743	0.12	0.54	C15 H23 O	(M+H)+

--- End Of Report ---

Compound 7d ^{1}H NMR (600 MHz, $CDCl_3$)

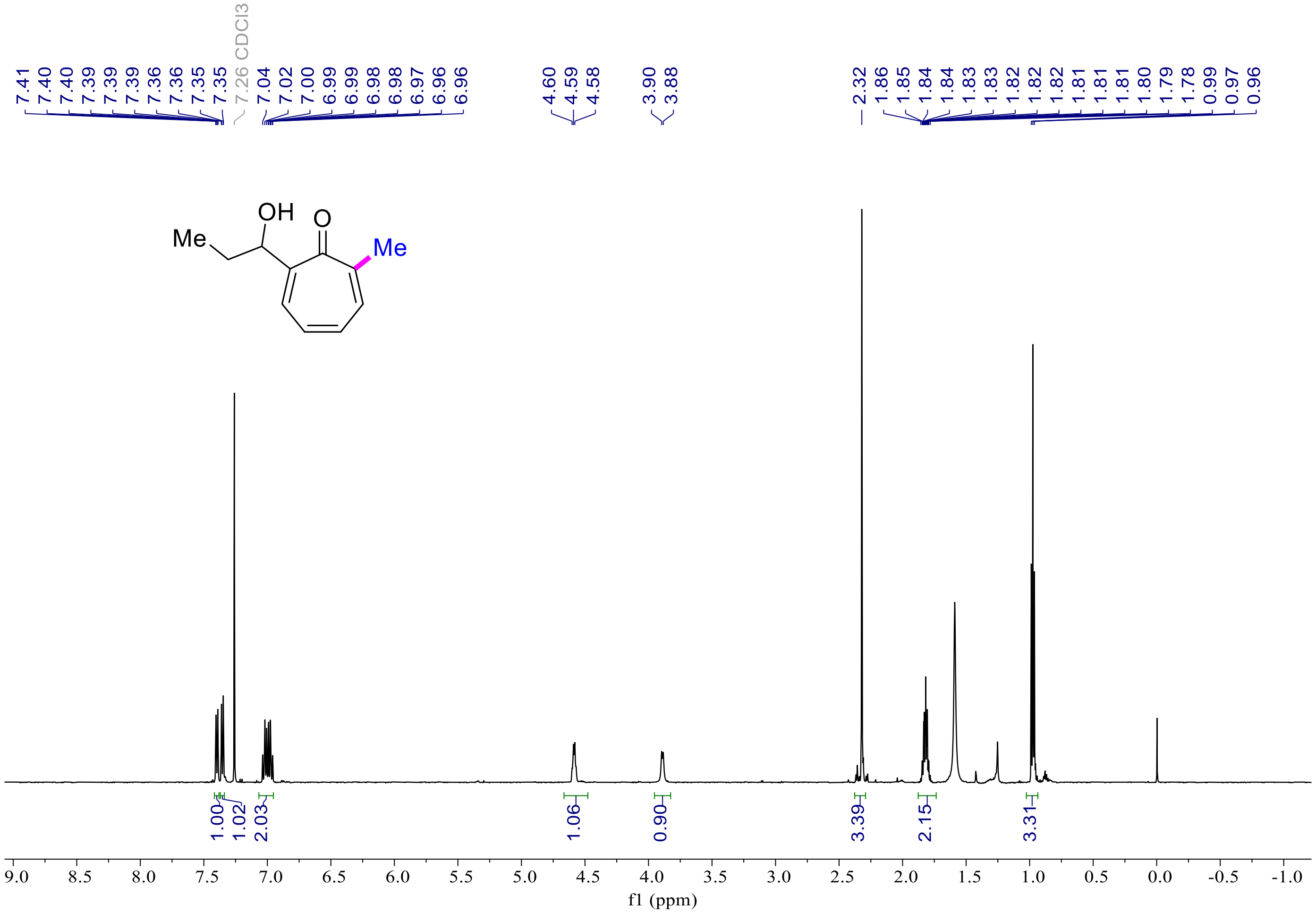

Compound 7d ^{13}C NMR (125 MHz, $CDCl_3$)

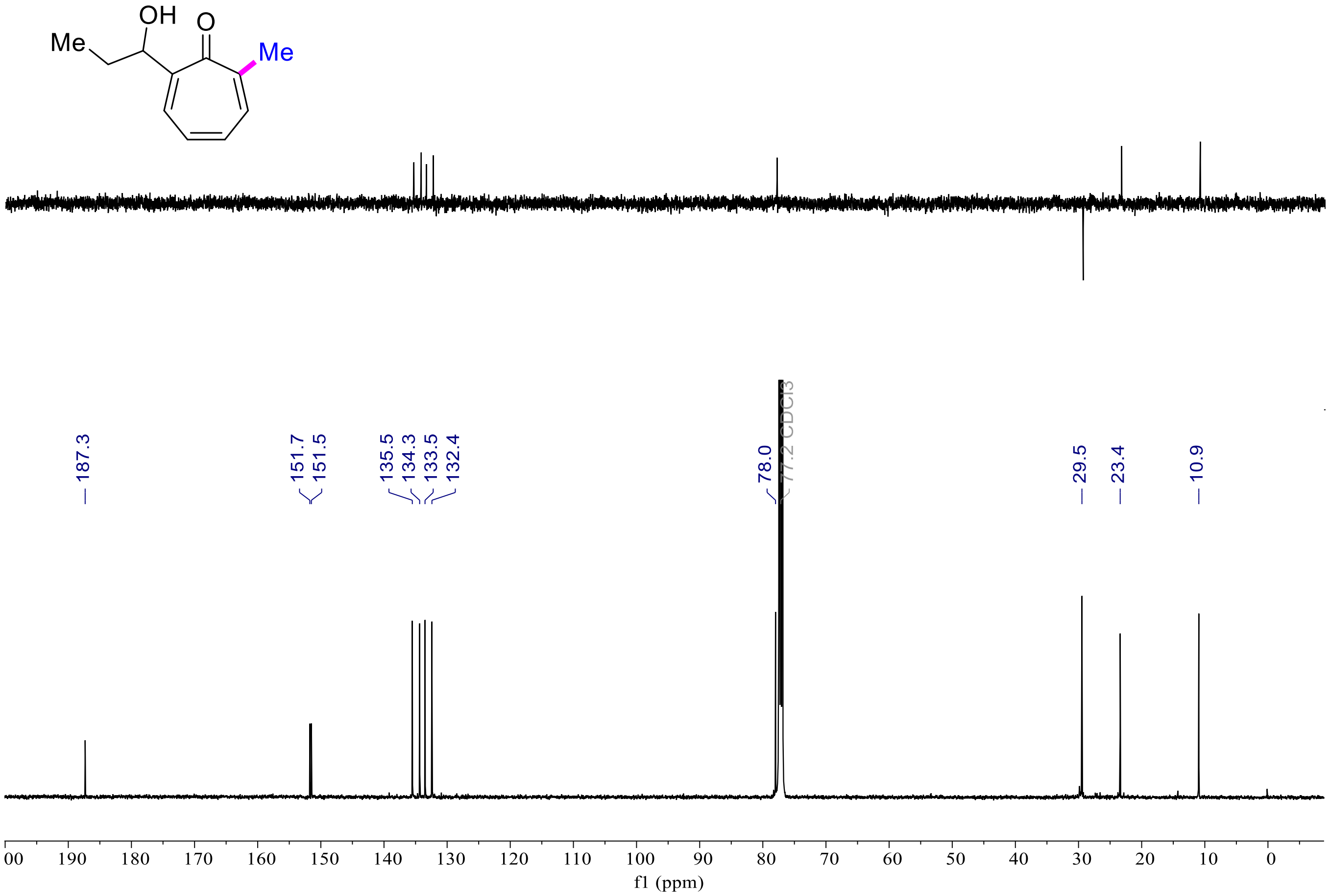

Compound 7d HRMS (ESI-TOF)

**Data Filename**	ESIH202404397.d	**Sample Name**	D4-ZQT20-2
**Sample ID**		**Position**	P1-A8
**Instrument Name**	Agilent 6520 Q-TOF	**Acq Method**	20160322_MS_ESIH_POS_1min.m
**Acquired Time**	9/10/2024 2:54:36 PM	**DA Method**	ESI-HR-20231114.m
**Comment**	ESIH by fangsu		

User Spectra

Fragmentor Voltage	Collision Energy	Ionization Mode
170	0	ESI

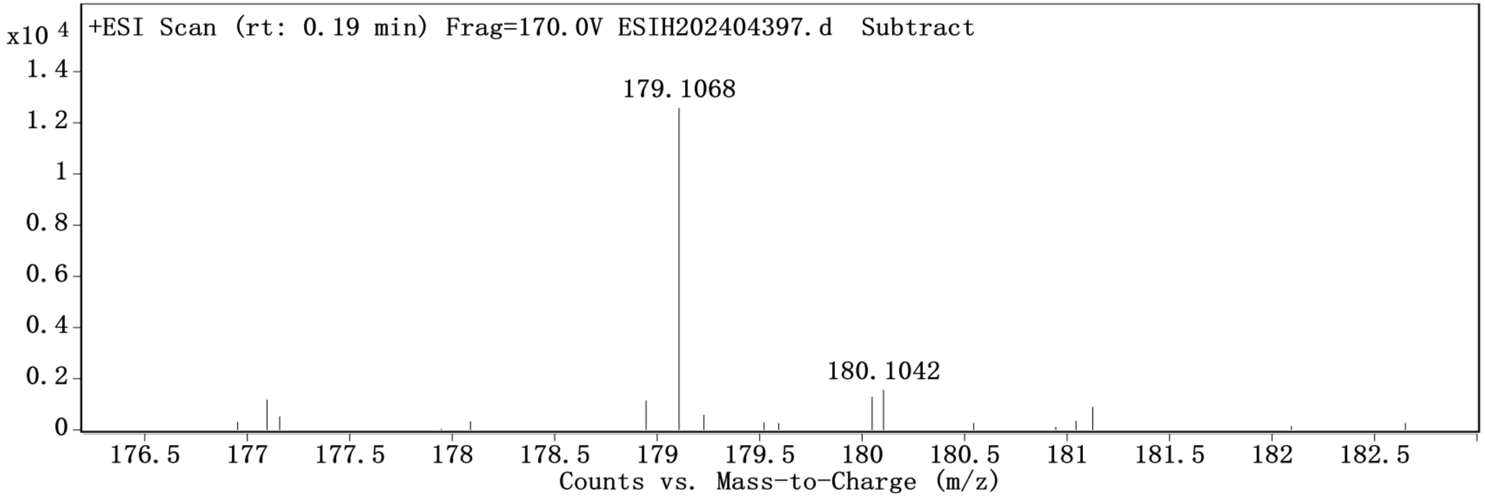

Formula Calculator Results

m/z	Calc m/z	Diff (mDa)	Diff (ppm)	Ion Formula	Ion
179.1068	179.1067	-0.11	-0.6	C11 H15 O2	(M+H)+

--- End Of Report ---

Compound 7e ^{1}H NMR (600 MHz, $CDCl_3$)

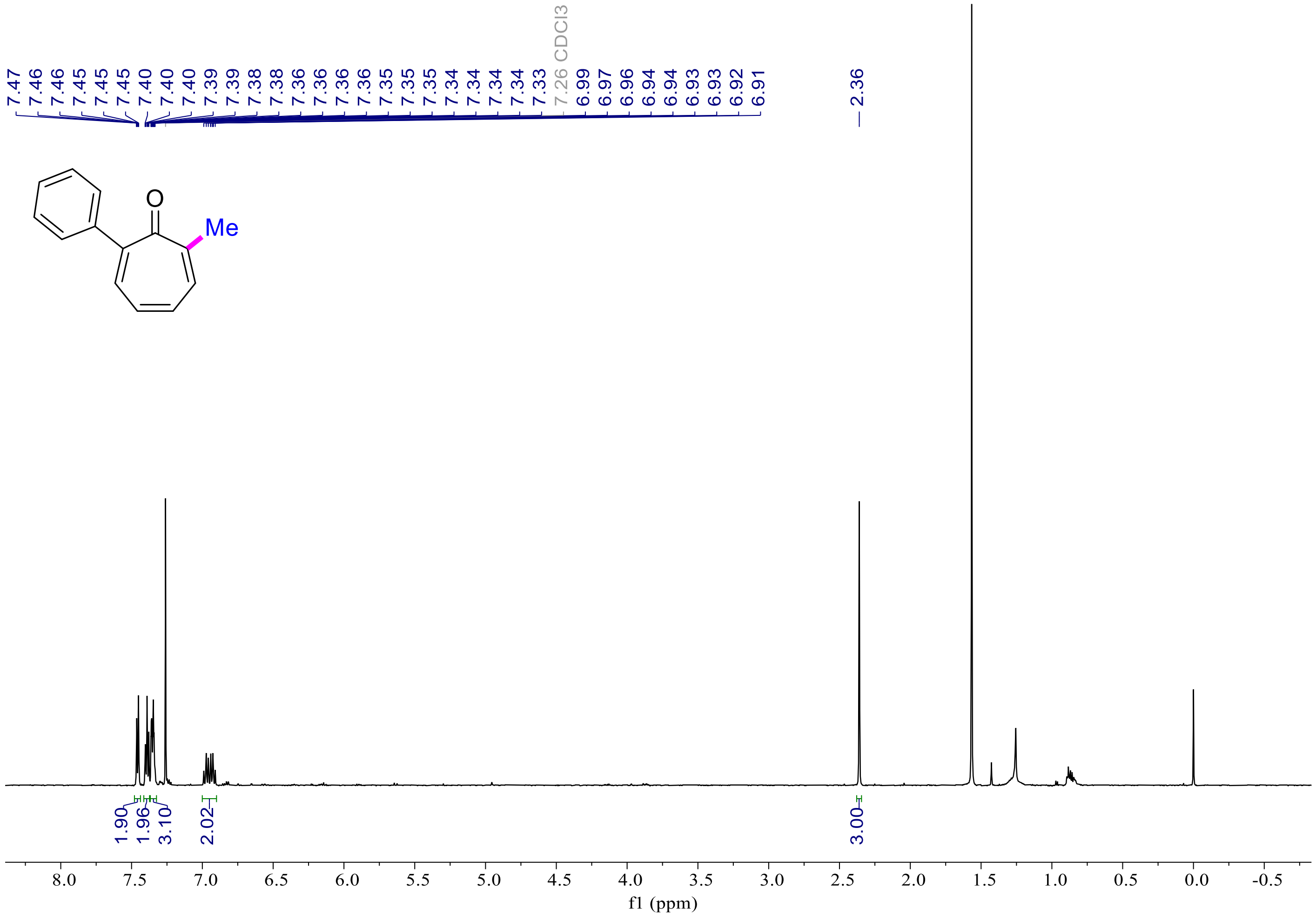

Compound 7e ^{13}C NMR (150 MHz, CDCl$_3$)

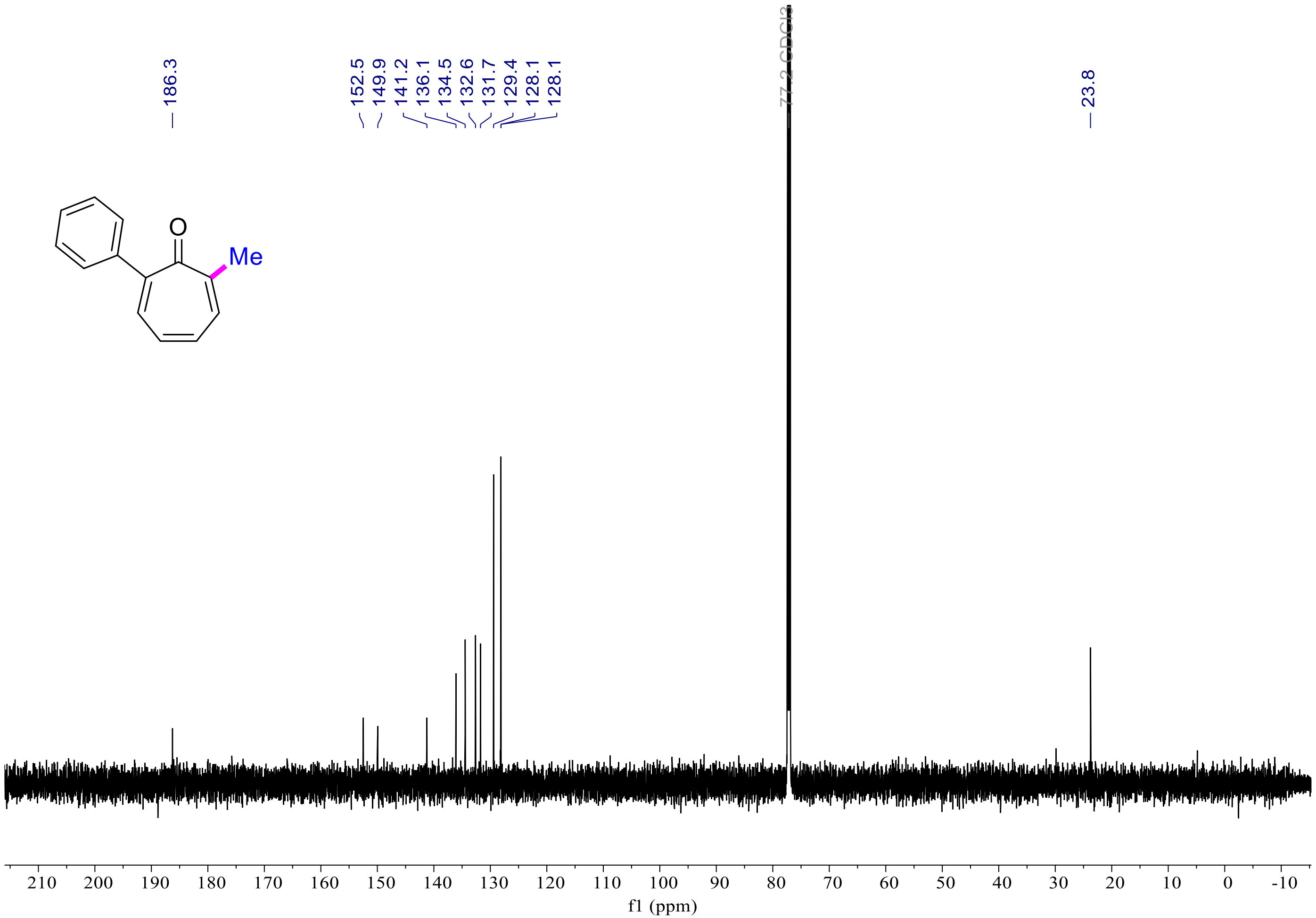

Compound 7e HRMS (ESI-TOF)

**Data Filename**	ESIH202404243.d	**Sample Name**	D4-ZQT18-11
**Sample ID**		**Position**	P1-C4
**Instrument Name**	Agilent 6520 Q-TOF	**Acq Method**	20160322_MS_ESIH_POS_1min.m
**Acquired Time**	9/2/2024 3:49:34 PM	**DA Method**	ESI-HR-20231114.m
**Comment**	ESIH by fangsu		

User Spectra

**Fragmentor Voltage**	**Collision Energy**	**Ionization Mode**
170	0	ESI

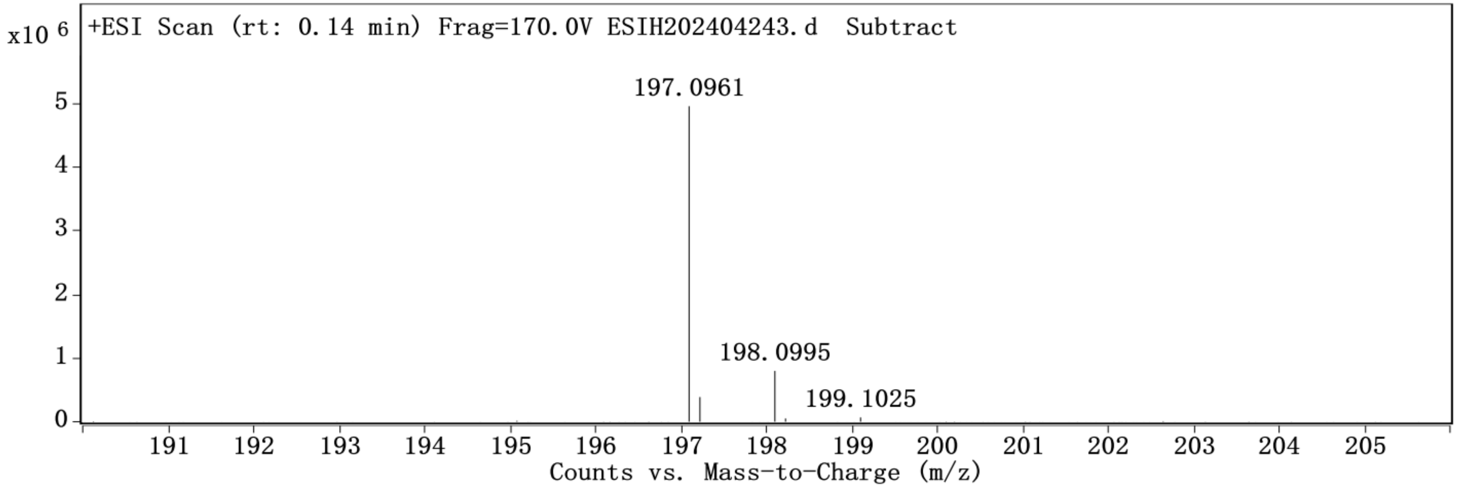

Formula Calculator Results

m/z	Calc m/z	Diff (mDa)	Diff (ppm)	Ion Formula	Ion
197.0961	197.0961	-0.03	-0.15	C14 H13 O	(M+H)+

--- End Of Report ---

Compound 7f ^{1}H NMR (600 MHz, $CDCl_3$)

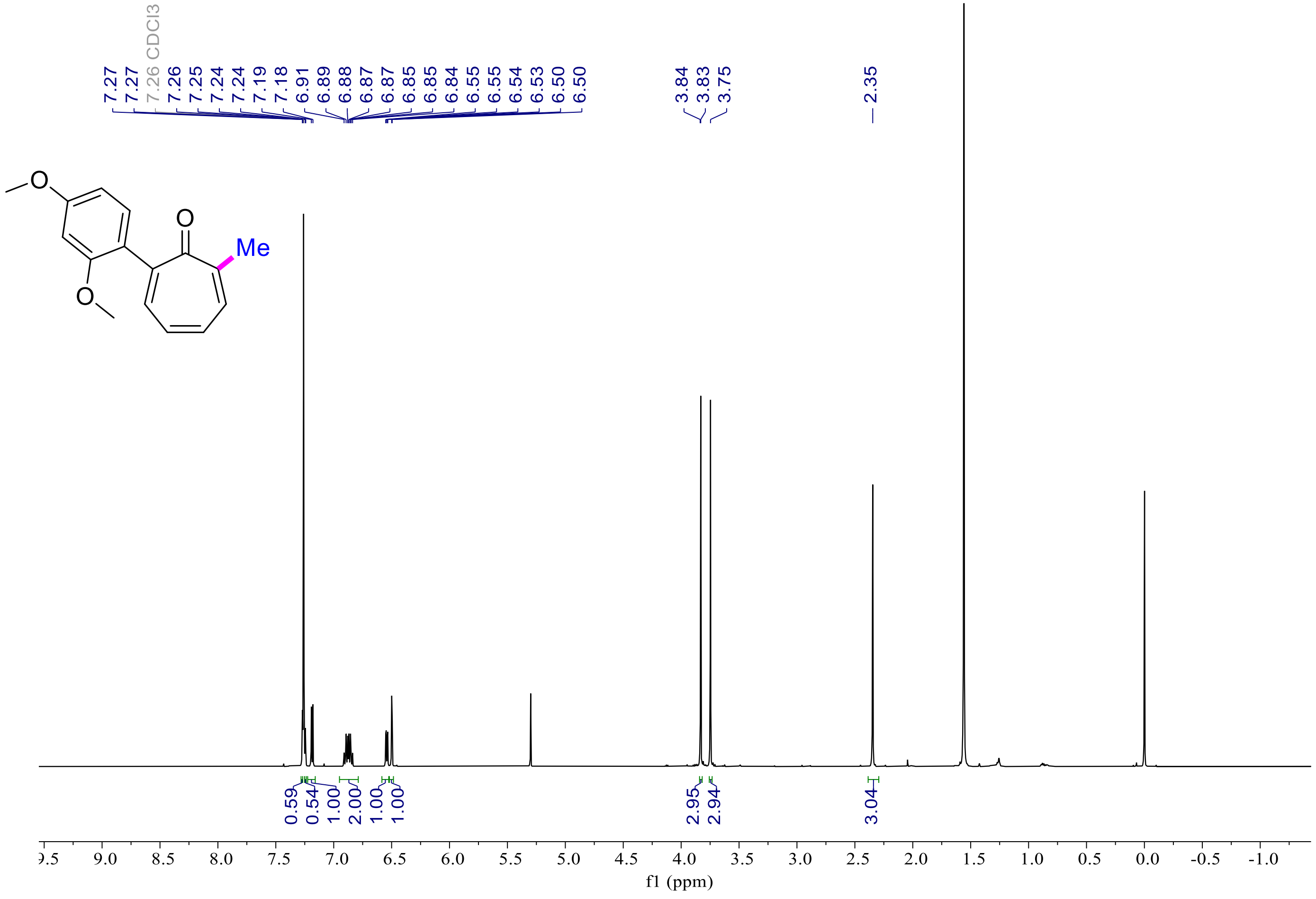

Compound 7f ^{13}C NMR (150 MHz, $CDCl_3$)

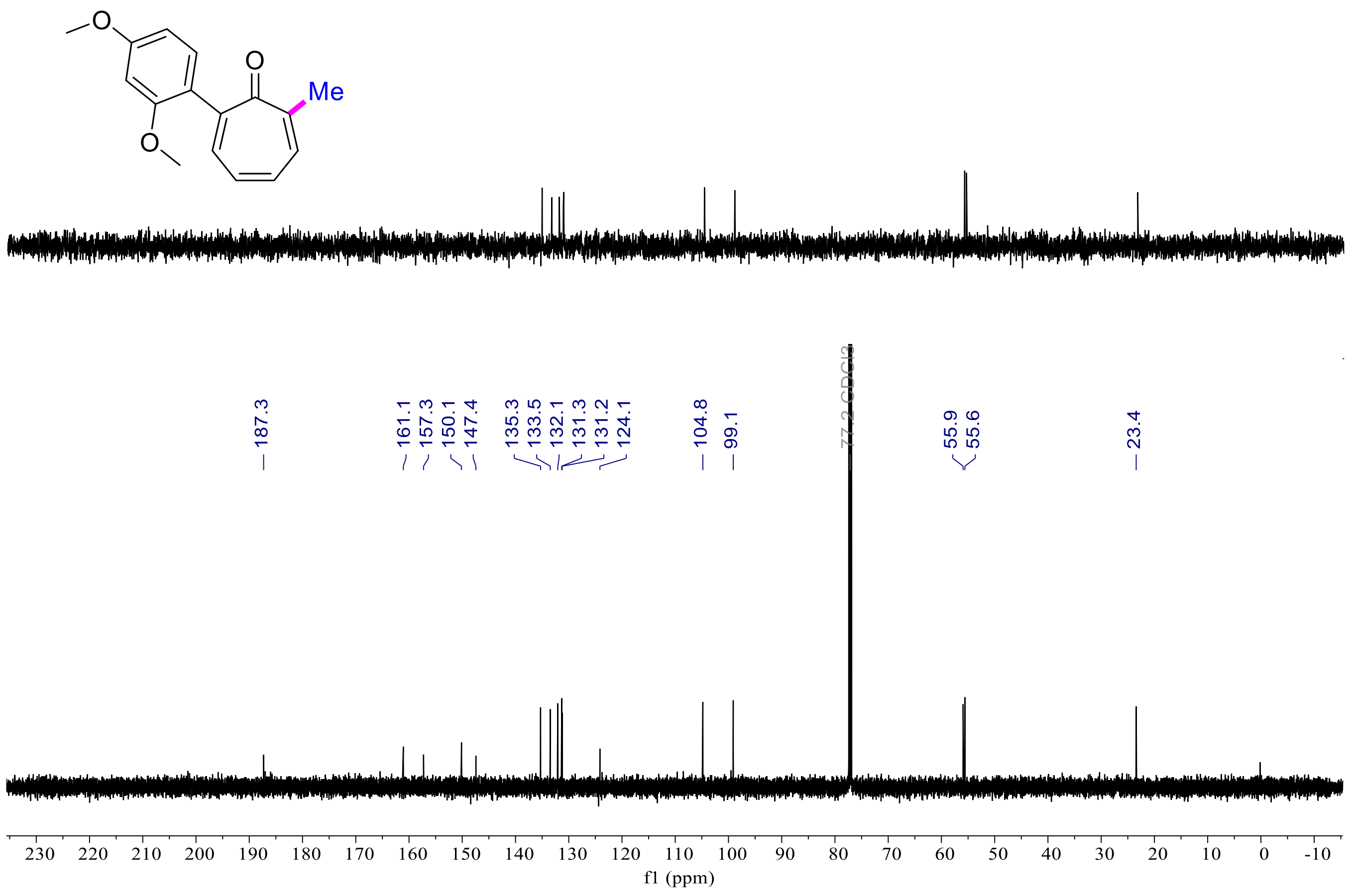

Compound 7f HRMS (ESI-TOF)

**Data Filename**	ESIH202404479.d	**Sample Name**	D4-ZQT18-31
**Sample ID**		**Position**	P1-F2
**Instrument Name**	Agilent 6520 Q-TOF	**Acq Method**	20160322_MS_ESIH_POS_1min.m
**Acquired Time**	9/13/2024 3:58:58 PM	**DA Method**	ESI-HR-20231114.m
**Comment**	ESIH by fangsu		

User Spectra

Fragmentor Voltage	Collision Energy	Ionization Mode
170	0	ESI

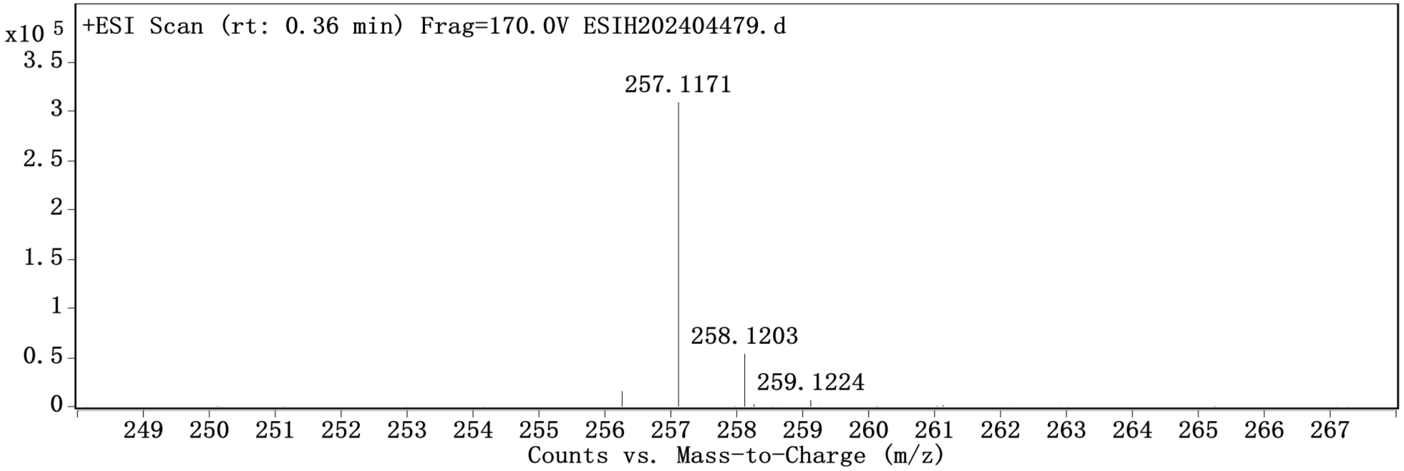

Formula Calculator Results

m/z	Calc m/z	Diff (mDa)	Diff (ppm)	Ion Formula	Ion
257.1171	257.1172	0.15	0.58	C16 H17 O3	(M+H)+

--- End Of Report ---

Compound 7g ¹H NMR (400 MHz, CDCl₃)

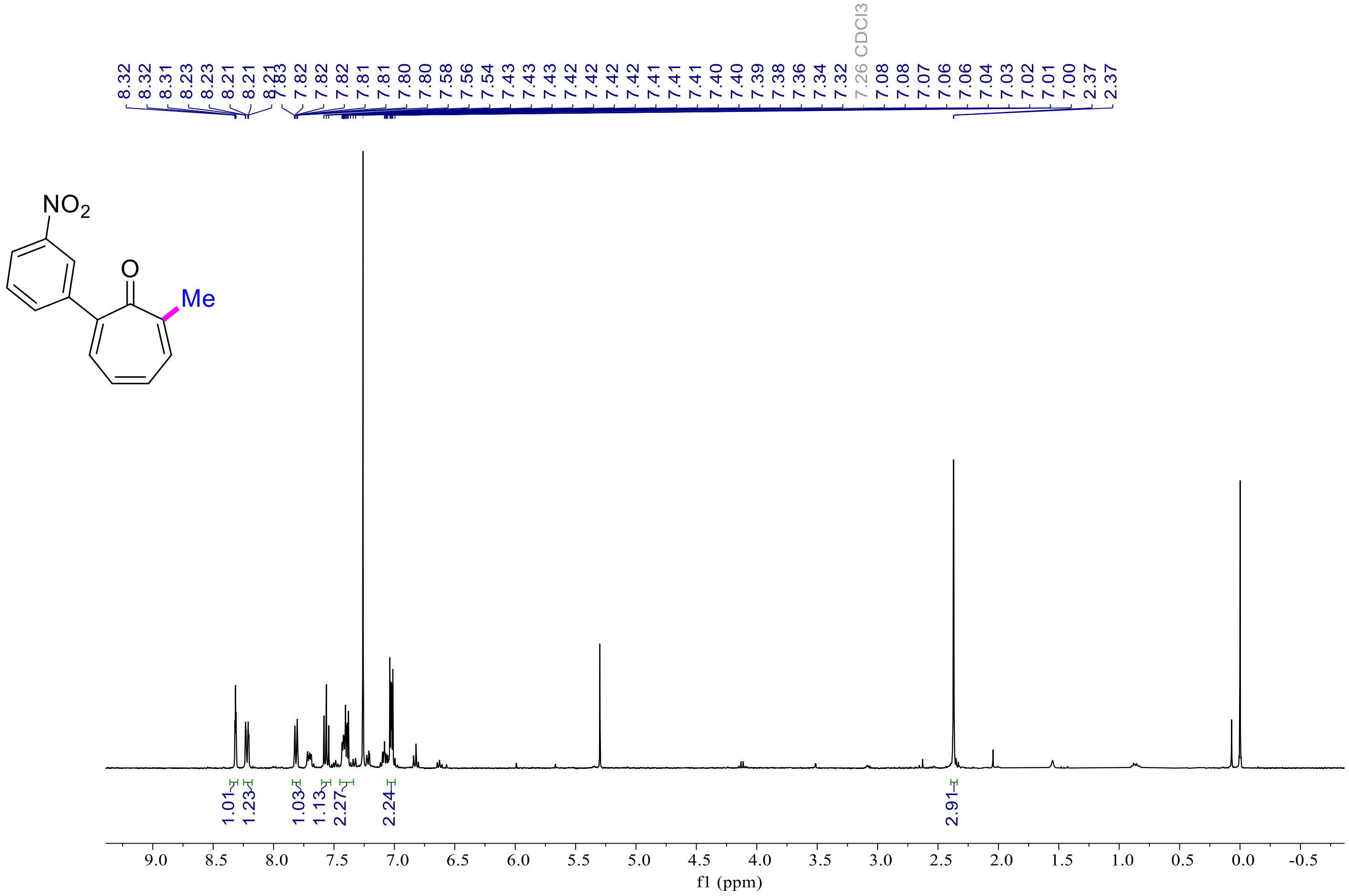

Compound 7g ^{13}C NMR (125 MHz, $CDCl_3$)

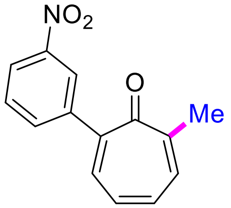

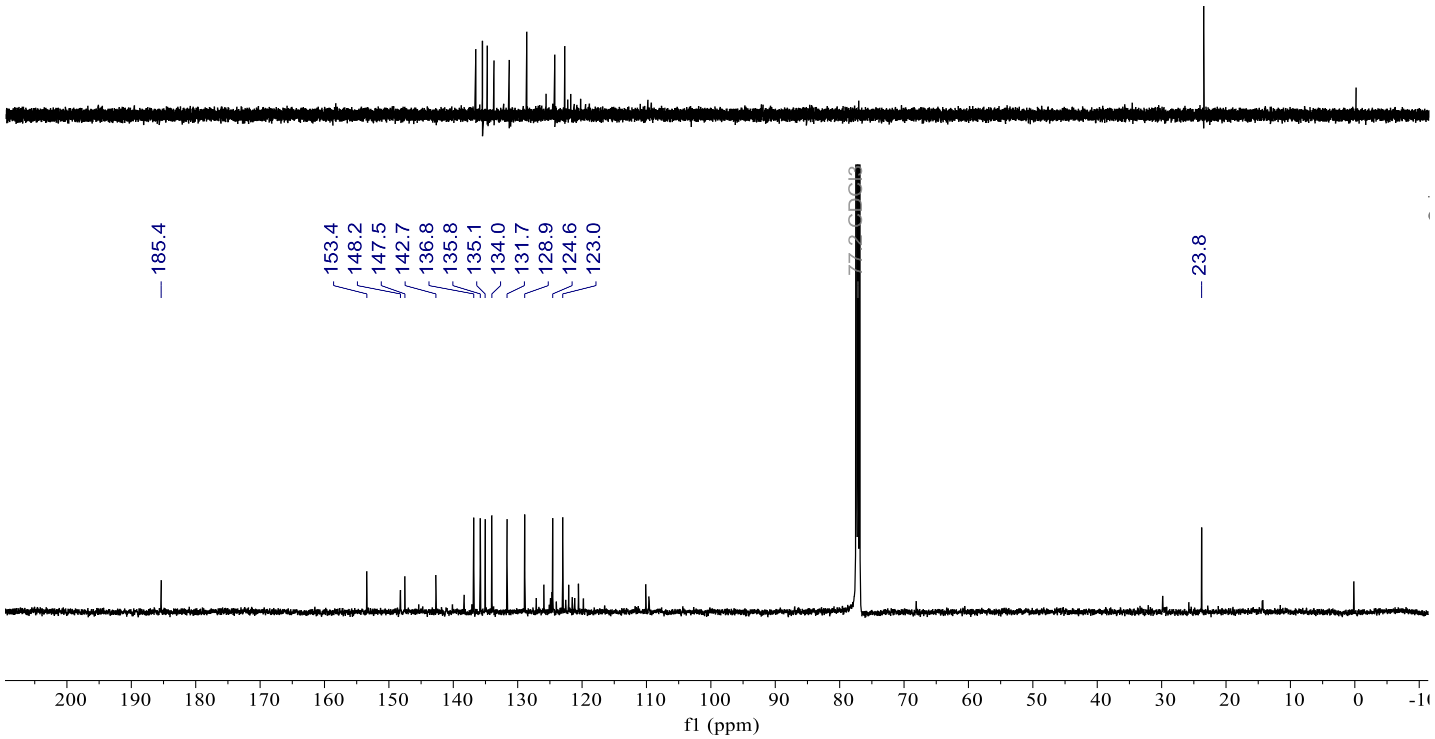

Compound 7g HRMS (ESI-TOF)

**Data Filename**	ESIH202501852.d	**Sample Name**	D4-ZQriv7
**Sample ID**		**Position**	P1-B6
**Instrument Name**	Agilent 6520 Q-TOF	**Acq Method**	20160322_MS_ESIH_POS_1min.m
**Acquired Time**	3/18/2025 2:43:40 PM	**DA Method**	ESI-HR-20231114.m
**Comment**	ESIH by fangsu		

User Spectra

Fragmentor Voltage	Collision Energy	Ionization Mode
170	0	ESI

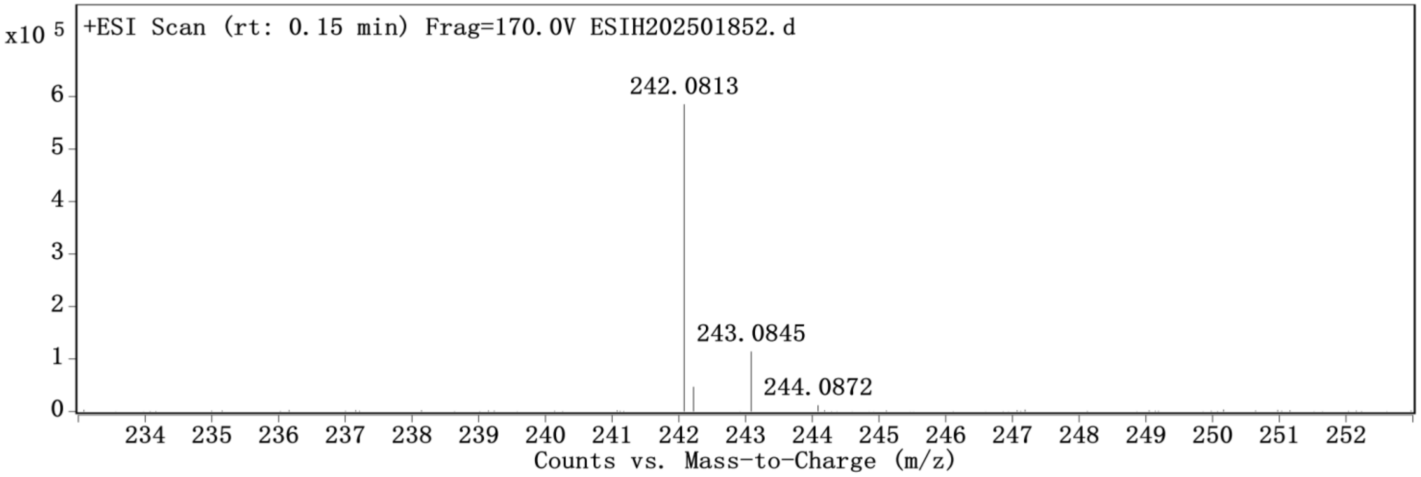

Formula Calculator Results

m/z	Calc m/z	Diff (mDa)	Diff (ppm)	Ion Formula	Ion
242.0813	242.0812	-0.16	-0.67	C14 H12 N O3	(M+H)+

--- End Of Report ---

Compound 7h ^{1}H NMR (500 MHz, $CDCl_3$)

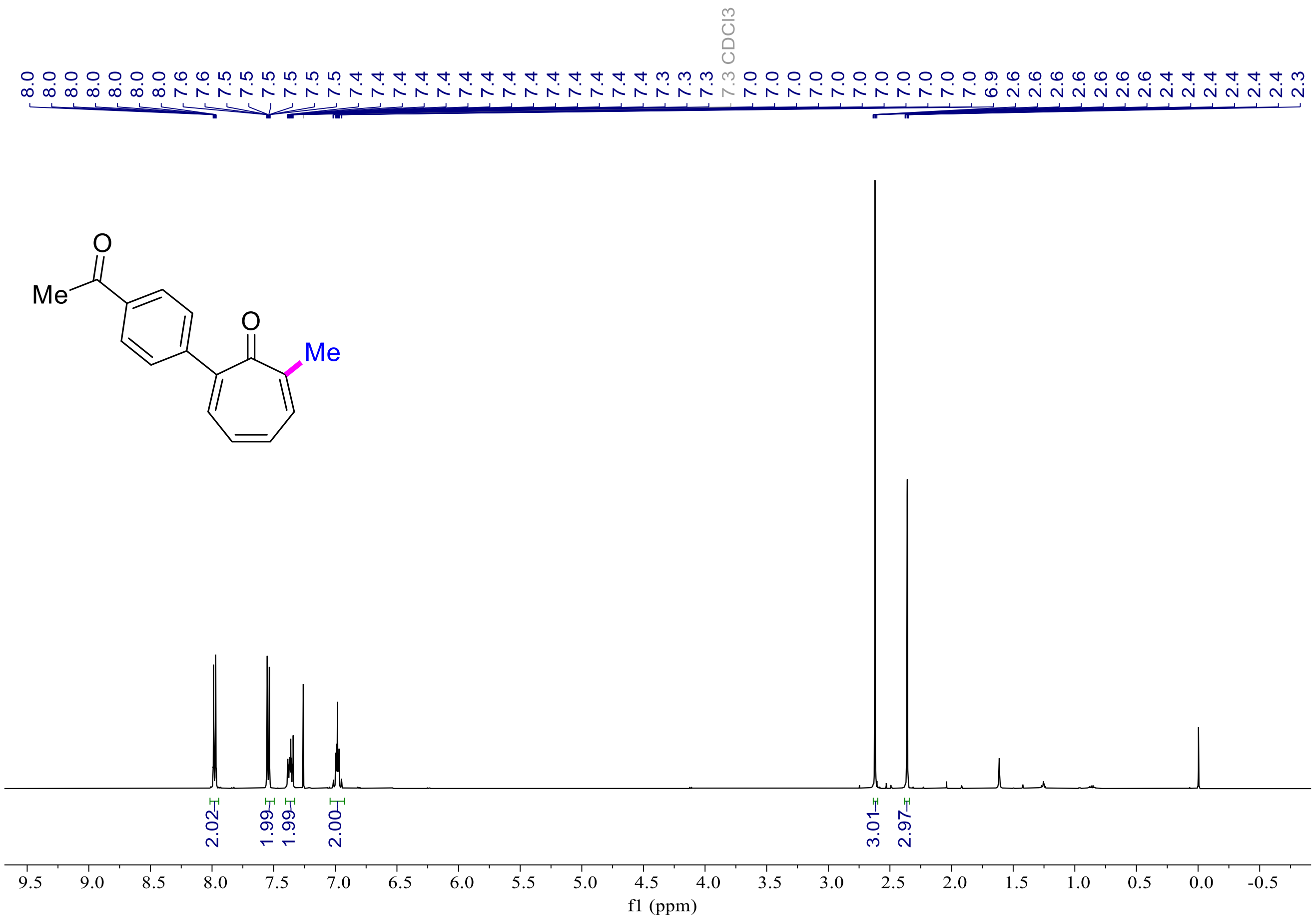

Compound 7h ^{13}C NMR (125 MHz, $CDCl_3$)

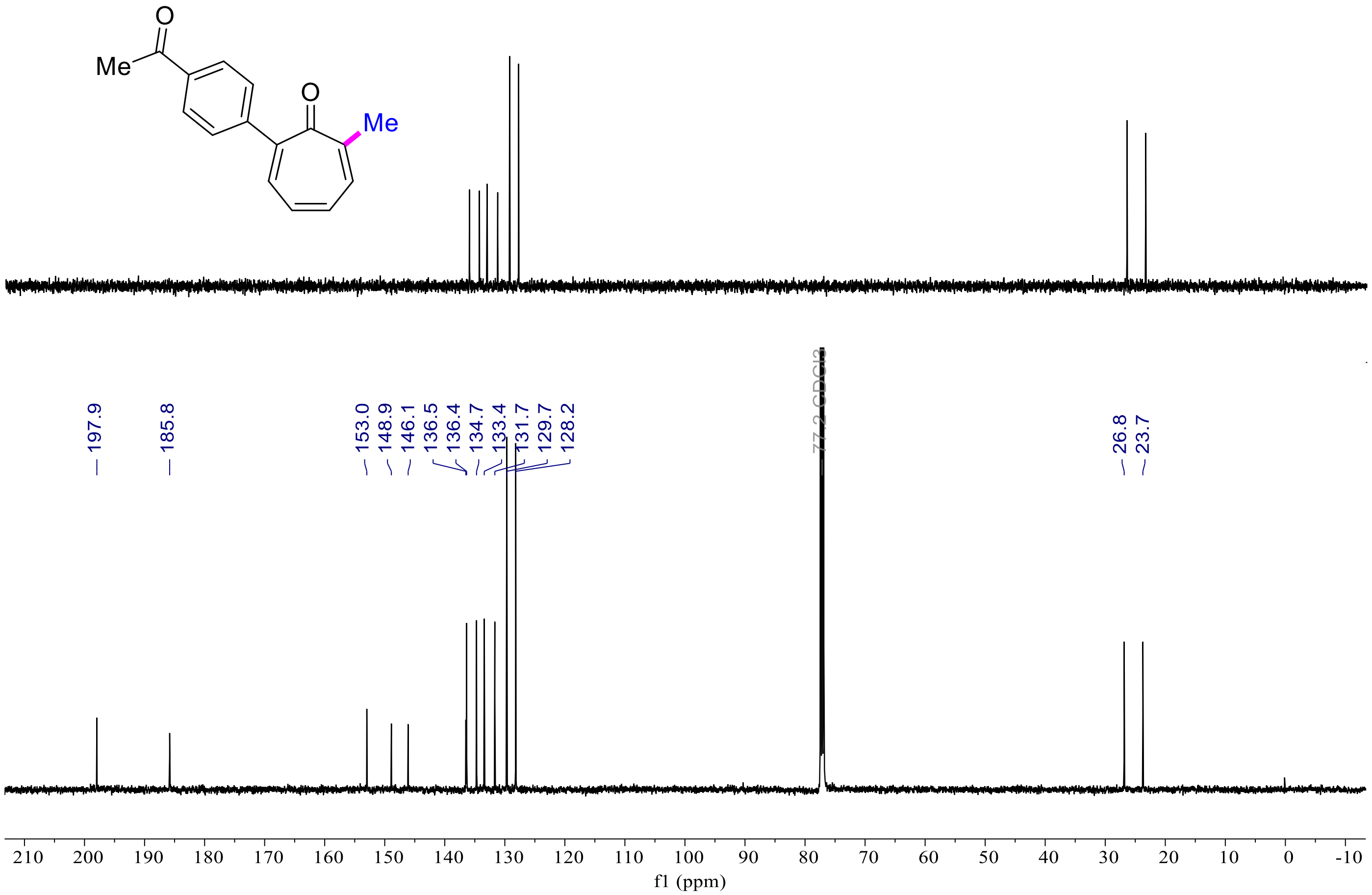

Compound 7h HRMS (ESI-TOF)

**Data Filename**	ESIH202501854.d	**Sample Name**	D4-ZQriv9
**Sample ID**		**Position**	P1-B8
**Instrument Name**	Agilent 6520 Q-TOF	**Acq Method**	20160322_MS_ESIH_POS_1min.m
**Acquired Time**	3/18/2025 2:46:15 PM	**DA Method**	ESI-HR-20231114.m
**Comment**	ESIH by fangsu		

User Spectra

Fragmentor Voltage	Collision Energy	Ionization Mode
170	0	ESI

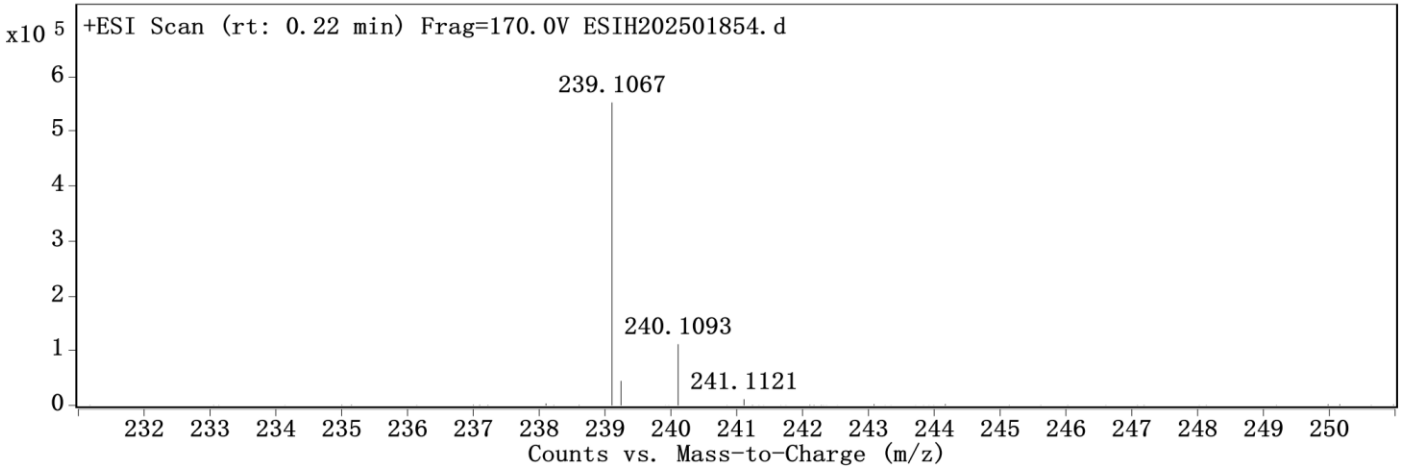

Formula Calculator Results

m/z	Calc m/z	Diff (mDa)	Diff (ppm)	Ion Formula	Ion
239.1067	239.1067	-0.01	-0.03	C16 H15 O2	(M+H)+

--- End Of Report ---

Compound 7i ^{1}H NMR (600 MHz, $CDCl_3$)

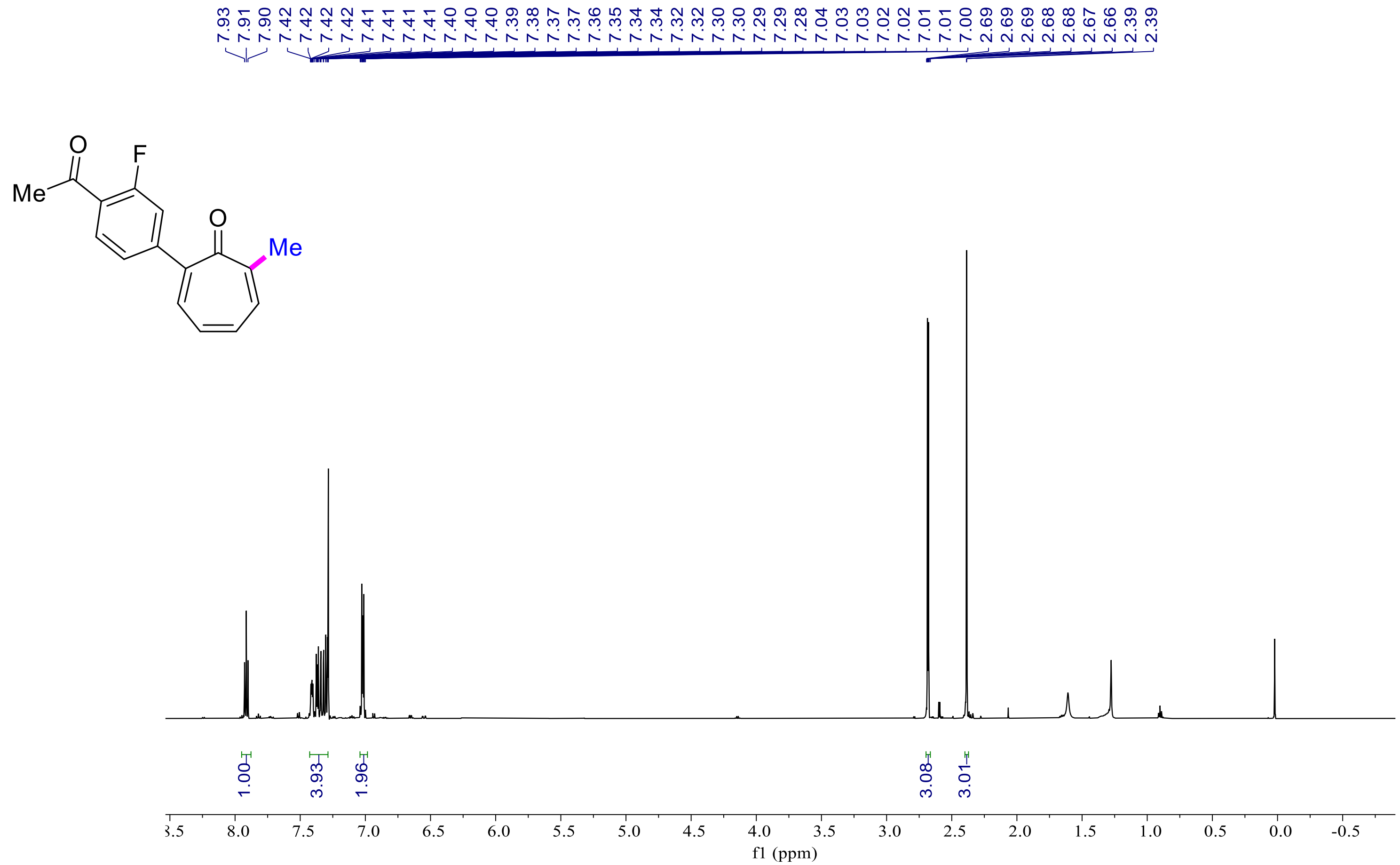

Compound 7i ^{13}C NMR (125 MHz, $CDCl_3$)

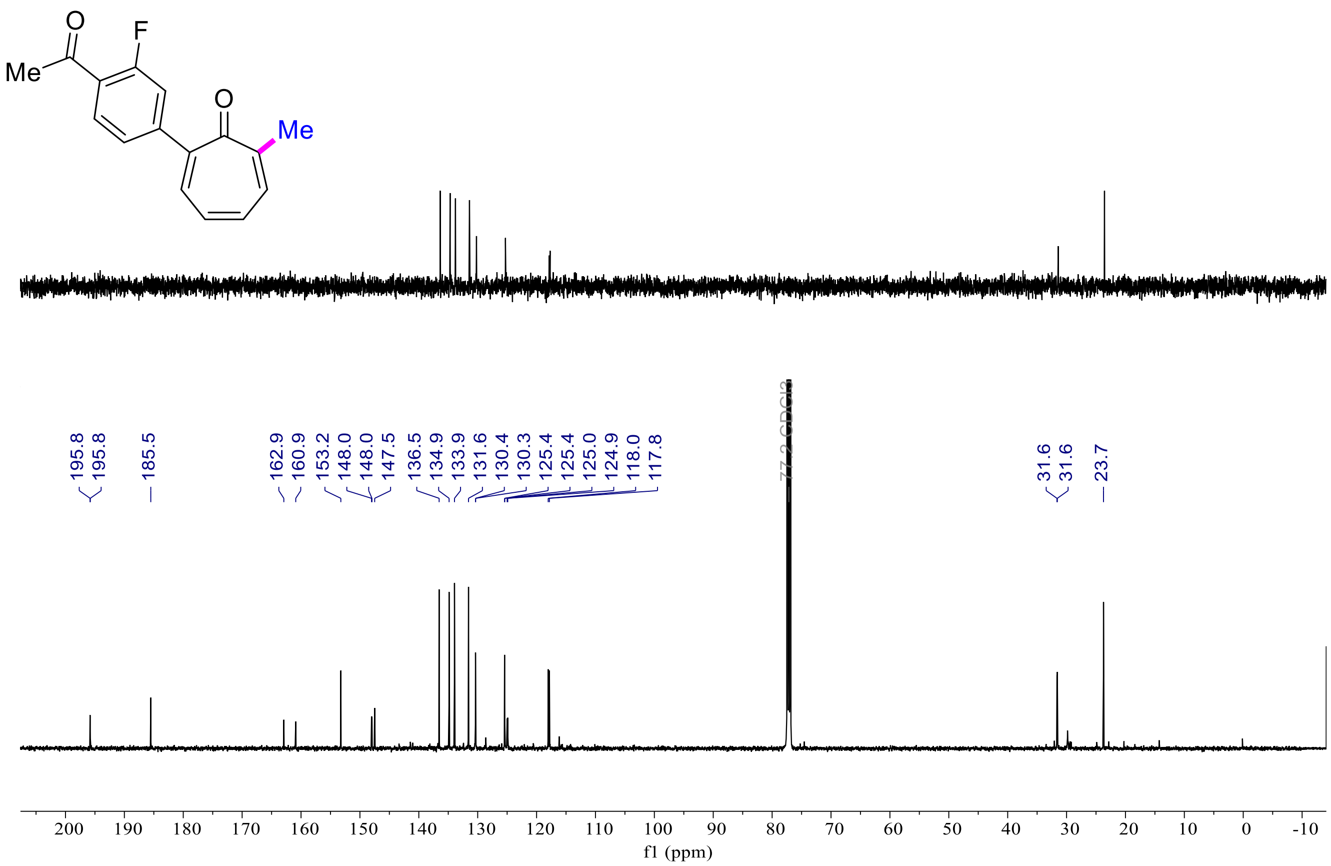

Compound 7i ^{19}F NMR (471 MHz, $CDCl_3$)

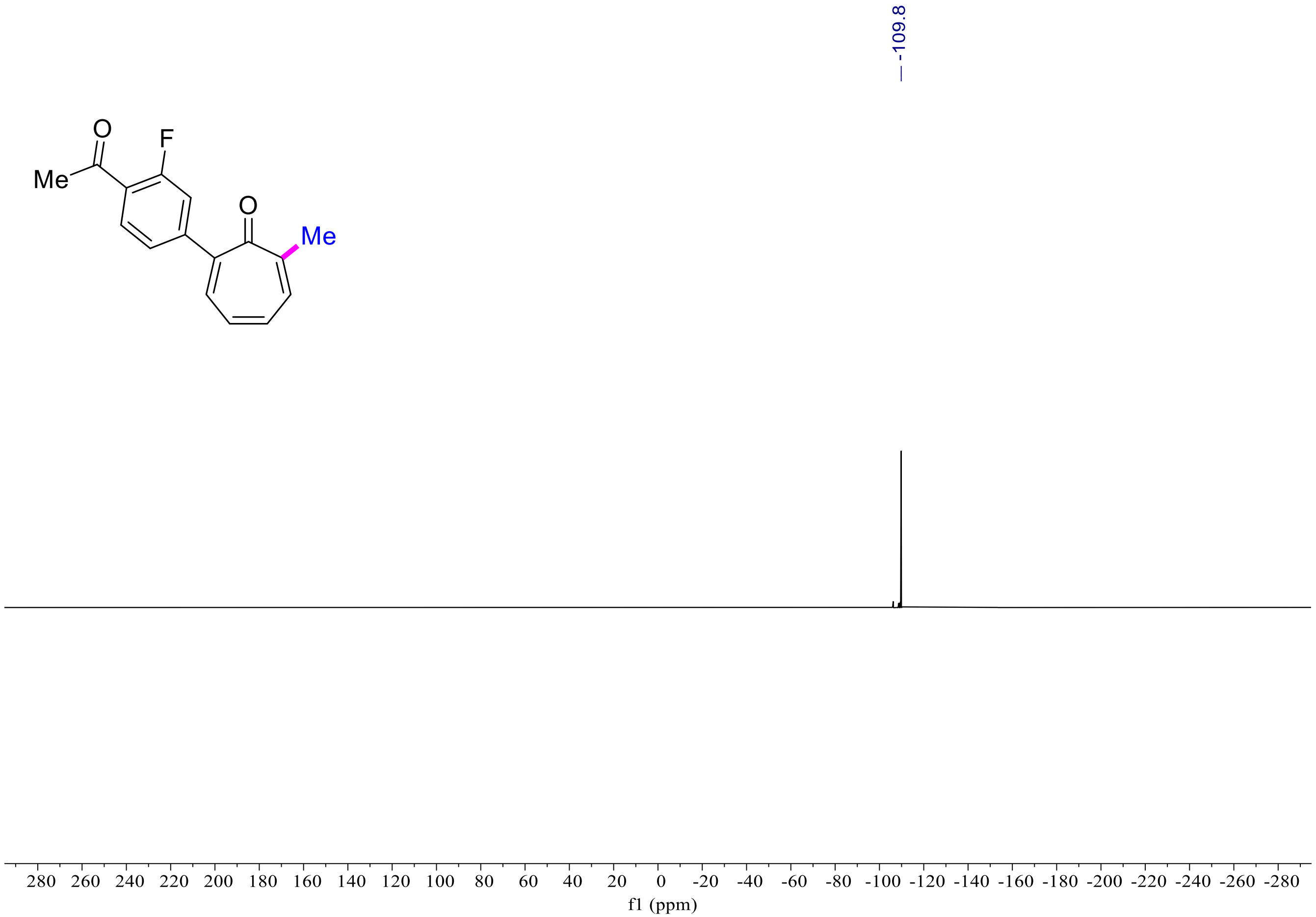

Compound 7i HRMS (ESI-TOF)

**Data Filename**	ESIH202502015.d	**Sample Name**	D4-ZQriv18-4
**Sample ID**		**Position**	P1-A6
**Instrument Name**	Agilent 6520 Q-TOF	**Acq Method**	20160322_MS_ESIH_POS_1min.m
**Acquired Time**	3/25/2025 2:59:12 PM	**DA Method**	ESI-HR-20231114.m
**Comment**	ESIH by fangsu		

User Spectra

Fragmentor Voltage	Collision Energy	Ionization Mode
170	0	ESI

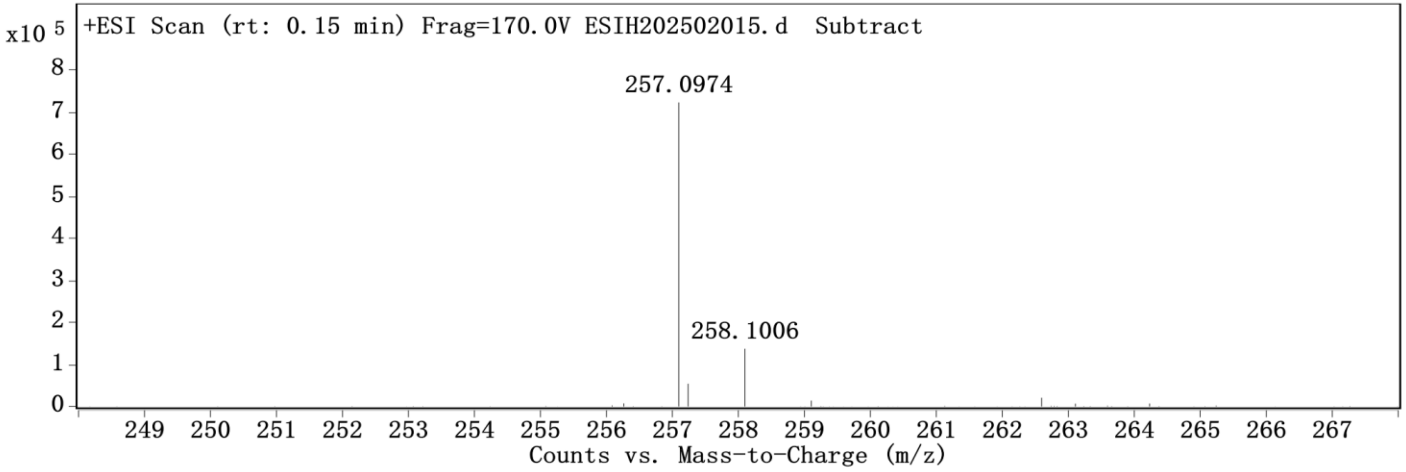

Formula Calculator Results

m/z	Calc m/z	Diff (mDa)	Diff (ppm)	Ion Formula	Ion
257.0974	257.0972	-0.18	-0.72	C16 H14 F O2	(M+H)+

--- End Of Report ---

Compound 7j ^{1}H NMR (600 MHz, $CDCl_3$)

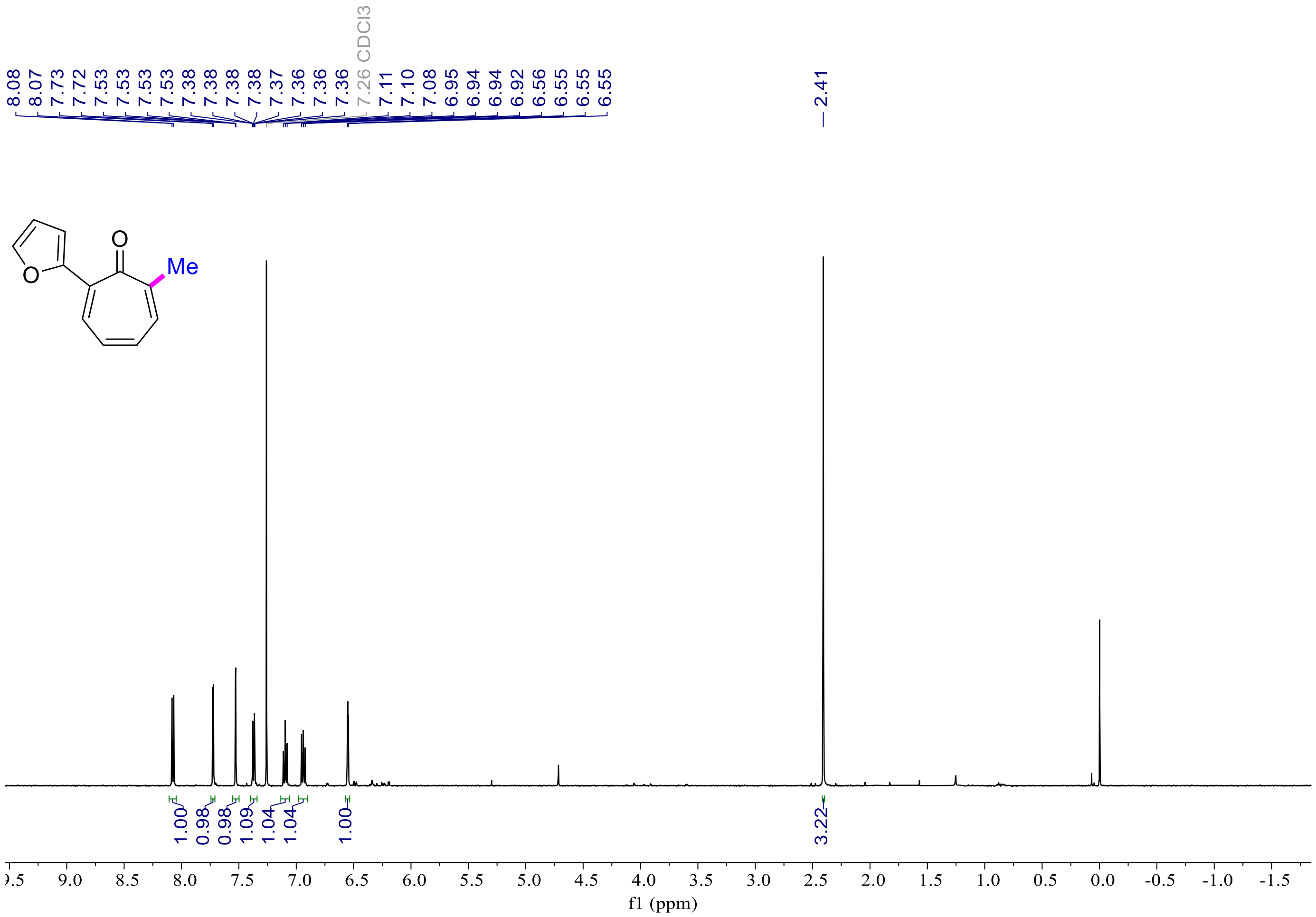

Compound 7j ^{13}C NMR (125 MHz, $CDCl_3$)

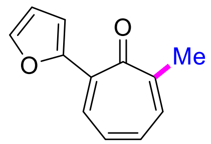

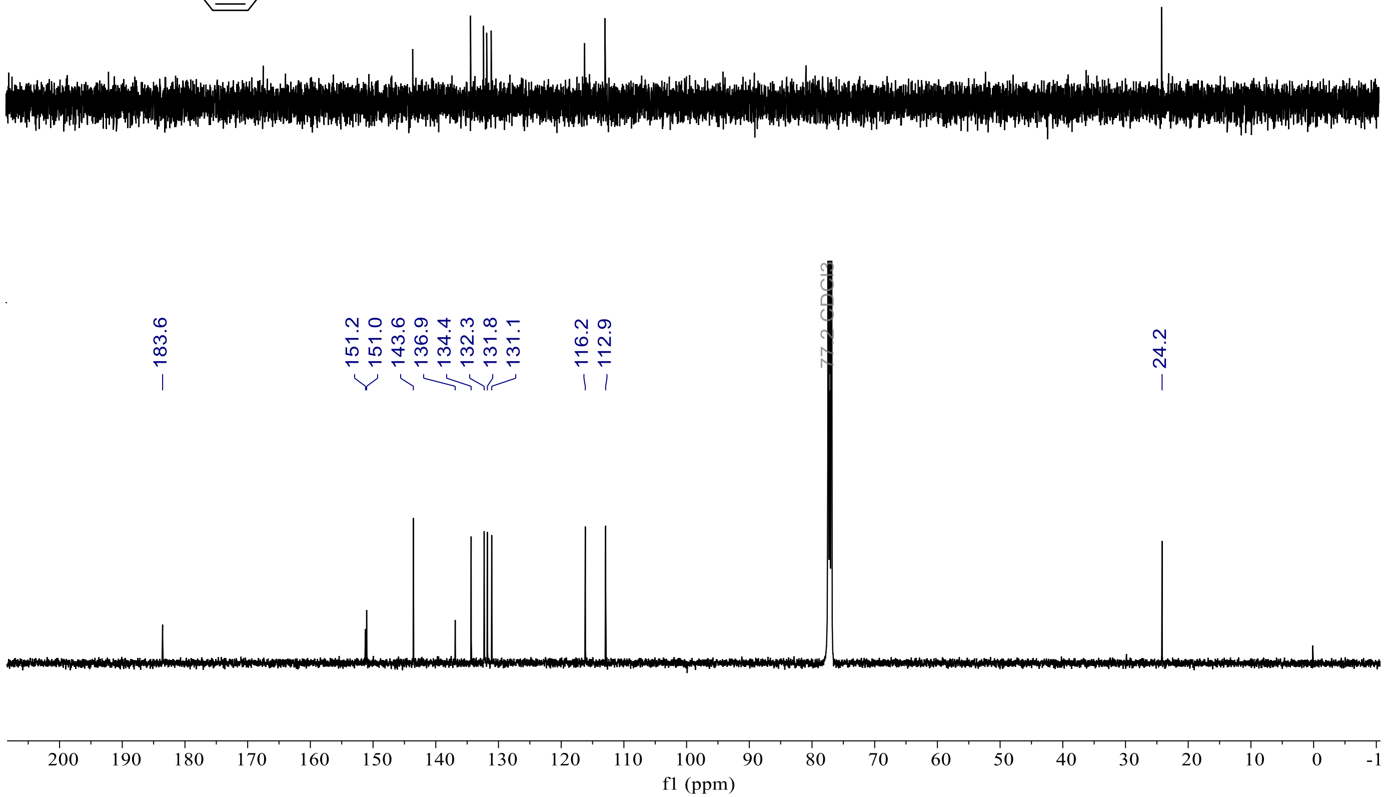

Compound 7j HRMS (ESI-TOF)

**Data Filename**	ESIH202404291.d	**Sample Name**	D4-ZQT18-19
**Sample ID**		**Position**	P1-A6
**Instrument Name**	Agilent 6520 Q-TOF	**Acq Method**	20160322_MS_ESIH_POS_1min.m
**Acquired Time**	9/5/2024 2:17:50 PM	**DA Method**	ESI-HR-20231114.m
**Comment**	ESIH by fangsu		

User Spectra

Fragmentor Voltage	Collision Energy	Ionization Mode
170	0	ESI

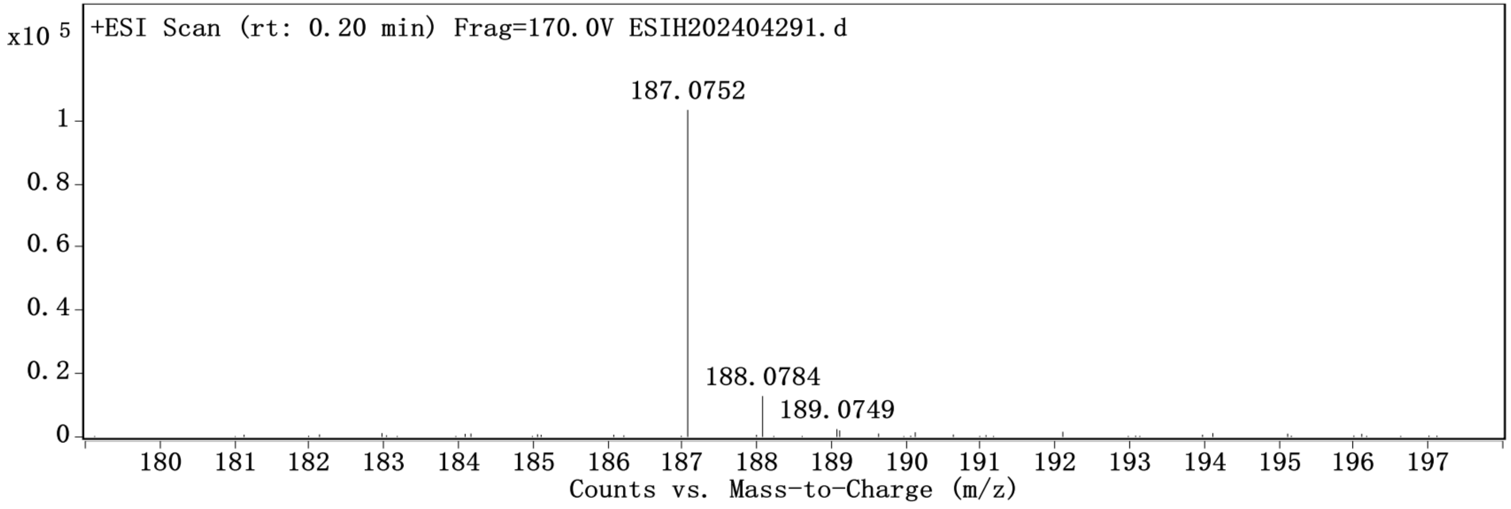

Formula Calculator Results

m/z	Calc m/z	Diff (mDa)	Diff (ppm)	Ion Formula	Ion
187.0752	187.0754	0.13	0.68	C12 H11 O2	(M+H)+

--- End Of Report ---

Compound 7k ^{1}H NMR (600 MHz, $CDCl_3$)

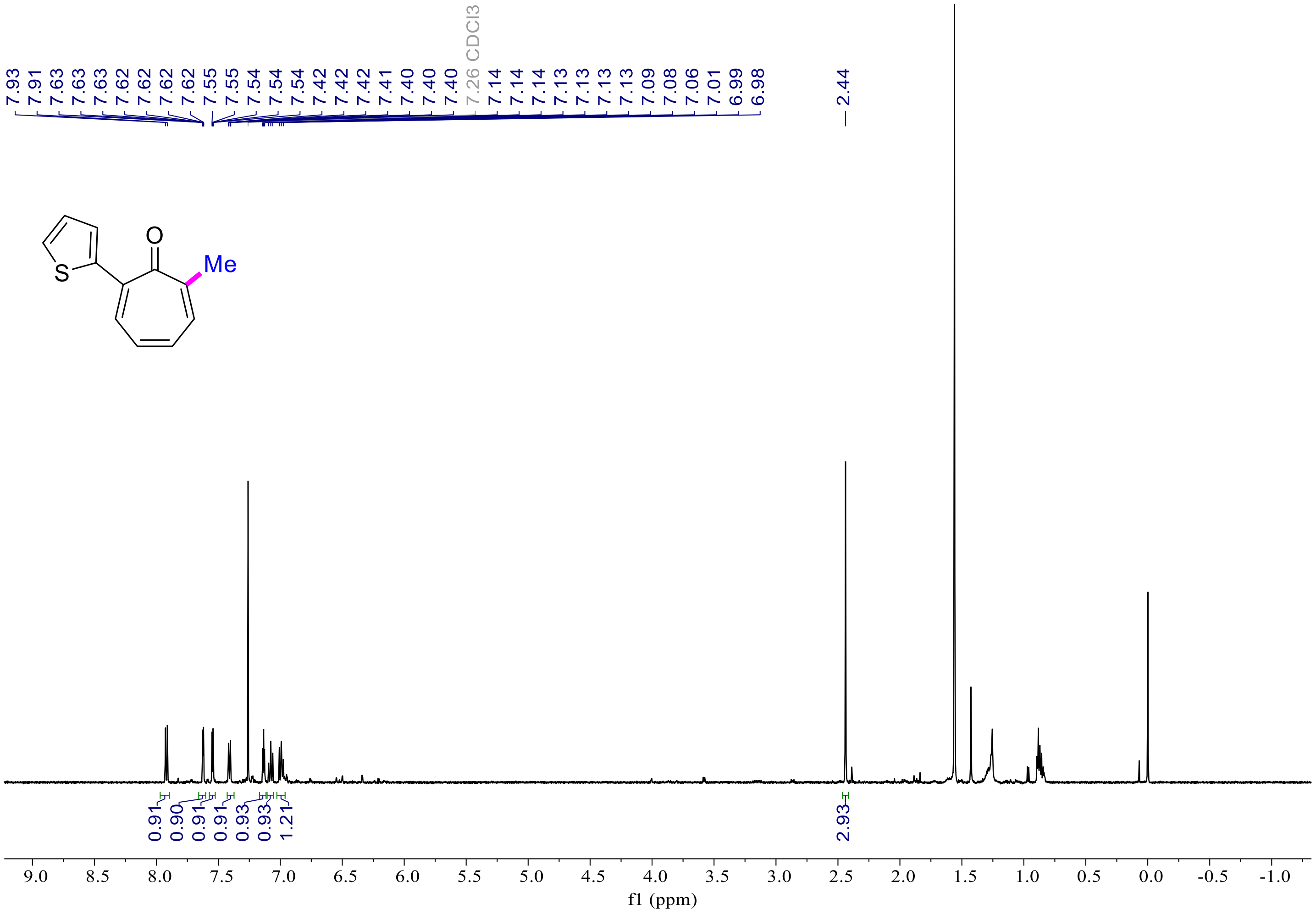

Compound 7k ^{13}C NMR (150 MHz, CDCl$_3$)

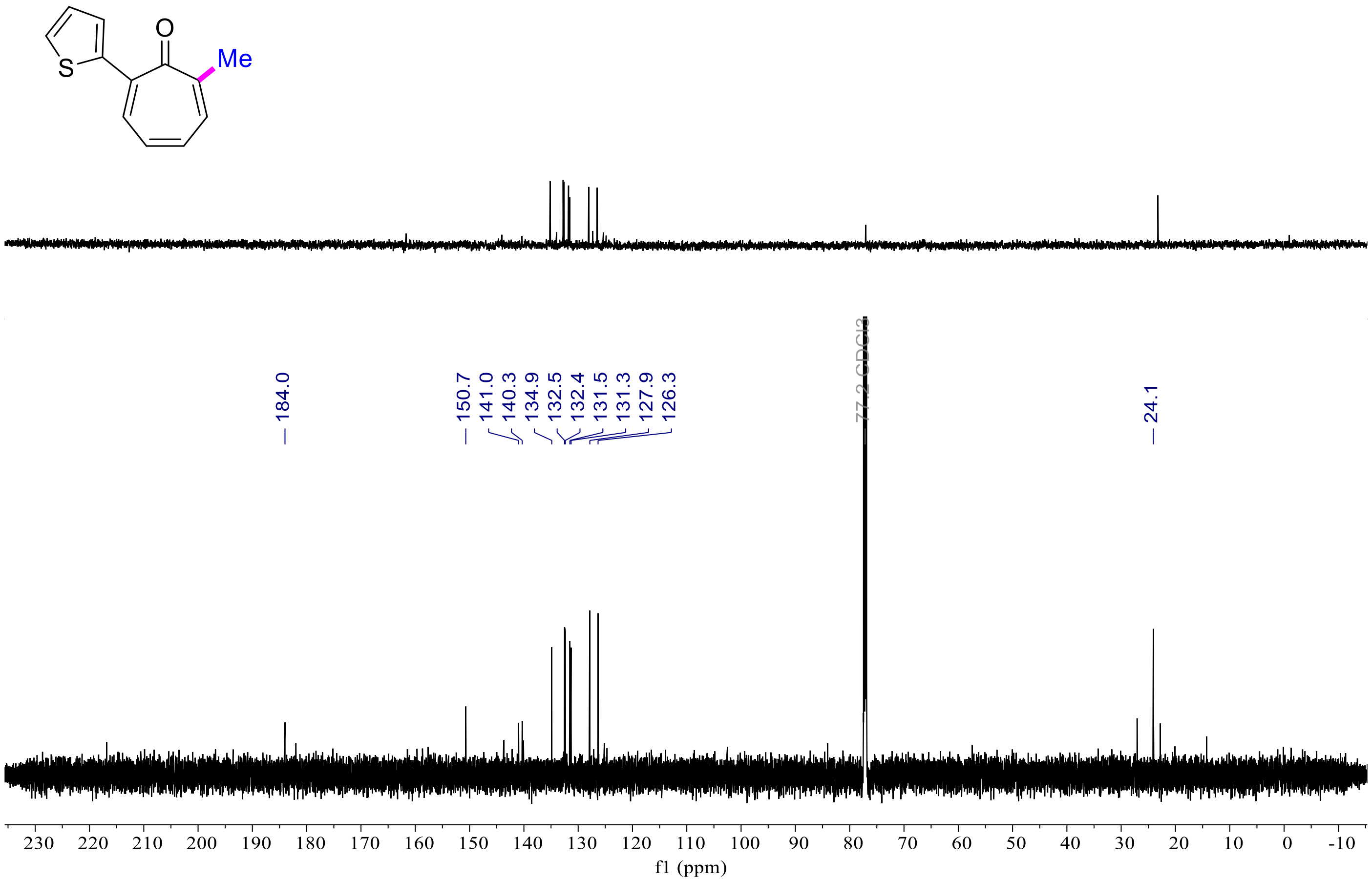

Compound 7k HRMS (ESI-TOF)

**Data Filename**	ESIH202404455.d	**Sample Name**	D4-ZQT18-29
**Sample ID**		**Position**	P1-B9
**Instrument Name**	Agilent 6520 Q-TOF	**Acq Method**	20160322_MS_ESIH_POS_1min.m
**Acquired Time**	9/13/2024 2:03:17 PM	**DA Method**	ESI-HR-20231114.m
**Comment**	ESIH by fangsu		

User Spectra

**Fragmentor Voltage**	**Collision Energy**	**Ionization Mode**
170	0	ESI

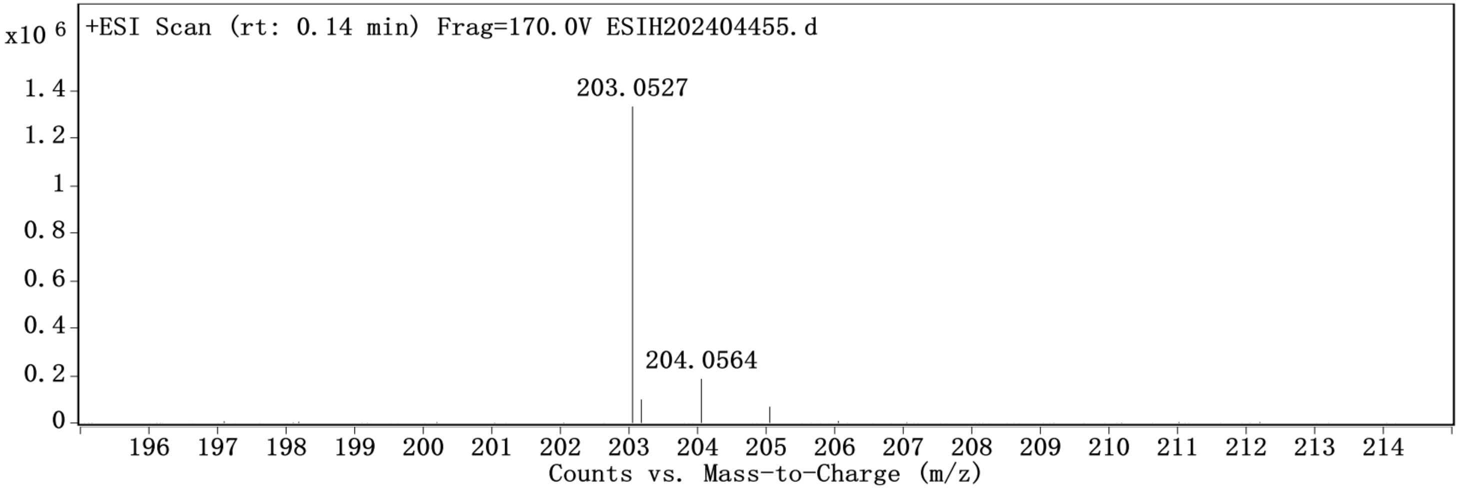

Formula Calculator Results

m/z	Calc m/z	Diff (mDa)	Diff (ppm)	Ion Formula	Ion
203.0527	203.0525	-0.2	-0.97	C12 H11 O S	(M+H)+

--- End Of Report ---

Compound 7l ¹H NMR (600 MHz, $CDCl_3$)

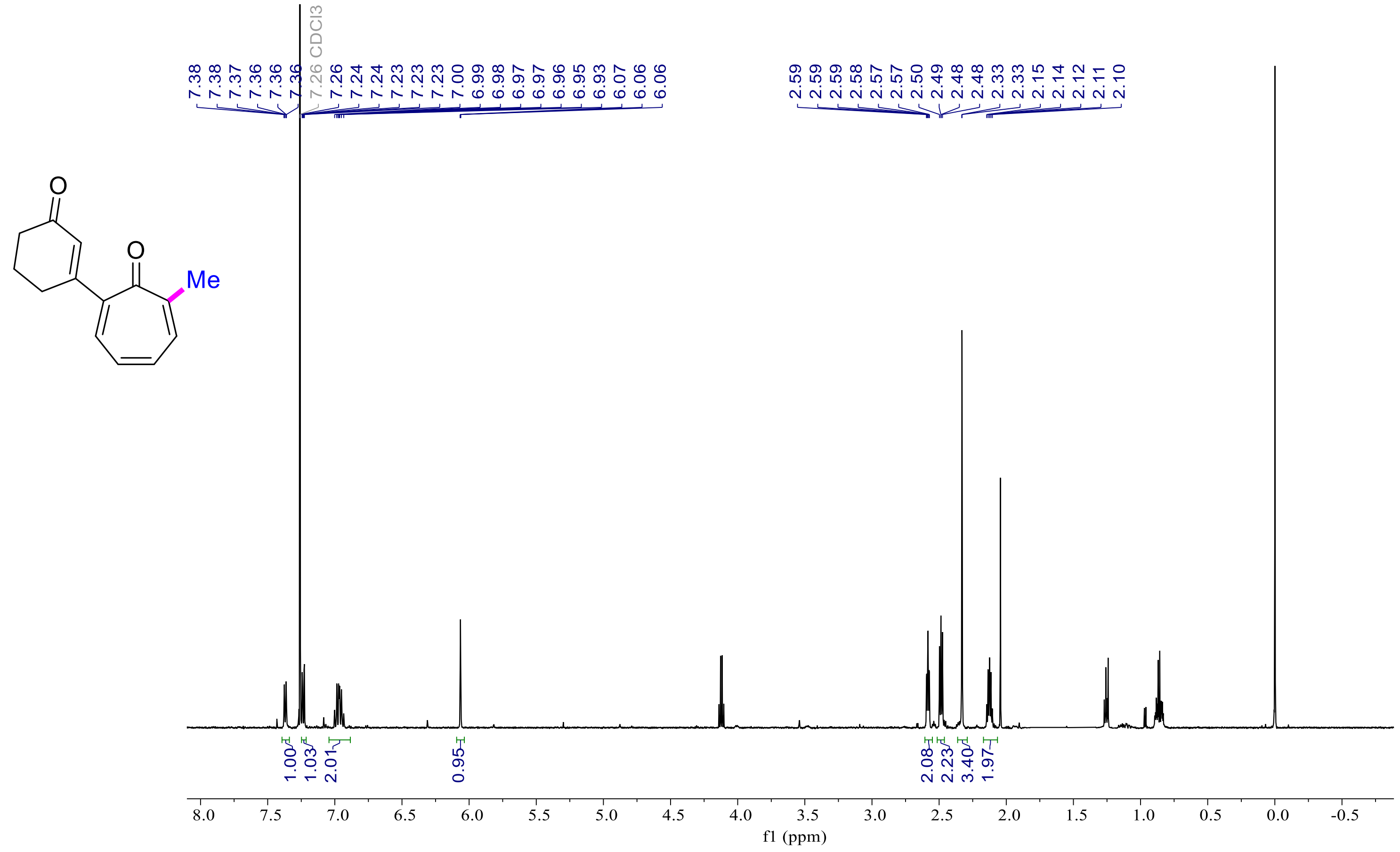

Compound 7l ^{13}C NMR (125 MHz, $CDCl_3$)

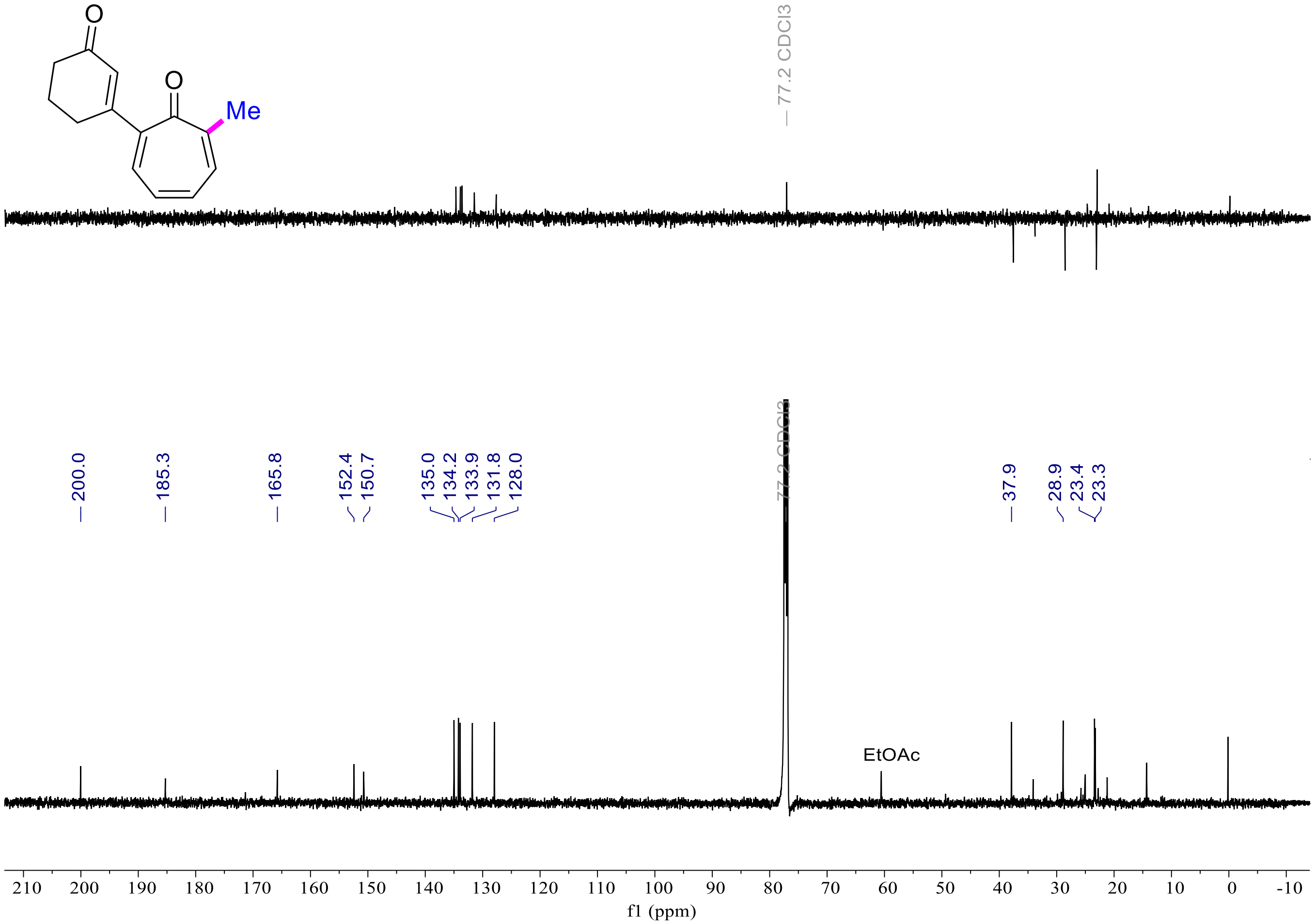

Compound 7l HRMS (ESI-TOF)

**Data Filename**	ESIH202502014.d	**Sample Name**	D4-ZQriv18-3A
**Sample ID**		**Position**	P1-A5
**Instrument Name**	Agilent 6520 Q-TOF	**Acq Method**	20160322_MS_ESIH_POS_1min.m
**Acquired Time**	3/25/2025 2:57:57 PM	**DA Method**	ESI-HR-20231114.m
**Comment**	ESIH by fangsu		

User Spectra

Fragmentor Voltage	Collision Energy	Ionization Mode
170	0	ESI

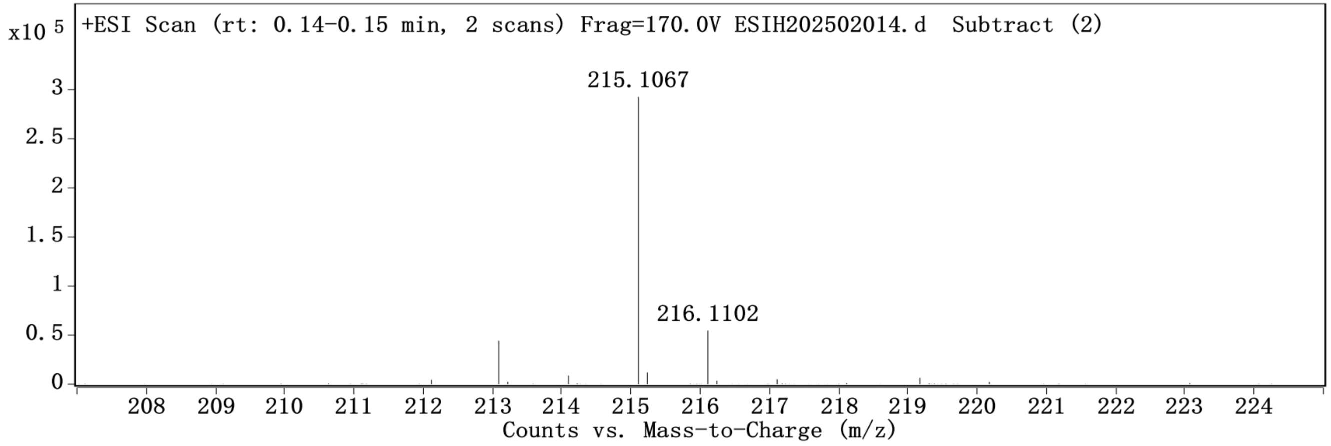

Formula Calculator Results

m/z	Calc m/z	Diff (mDa)	Diff (ppm)	Ion Formula	Ion
215.1067	215.1067	-0.02	-0.1	C14 H15 O2	(M+H)+

--- End Of Report ---

Compounds 7ma and 7mb ¹H NMR (600 MHz, CDCl₃)

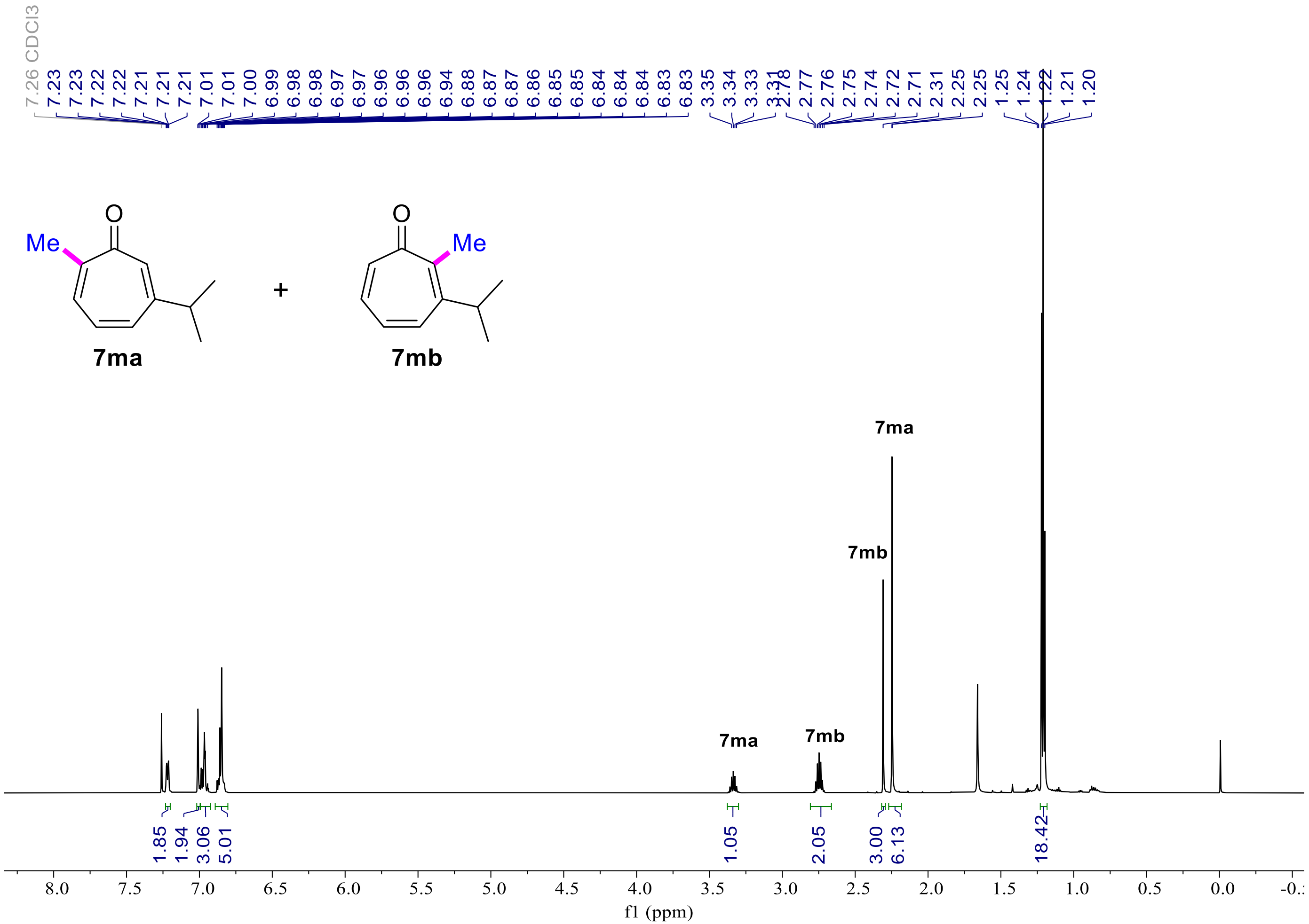

Compound 7ma and 7mb ^{13}C NMR (150 MHz, $CDCl_3$)

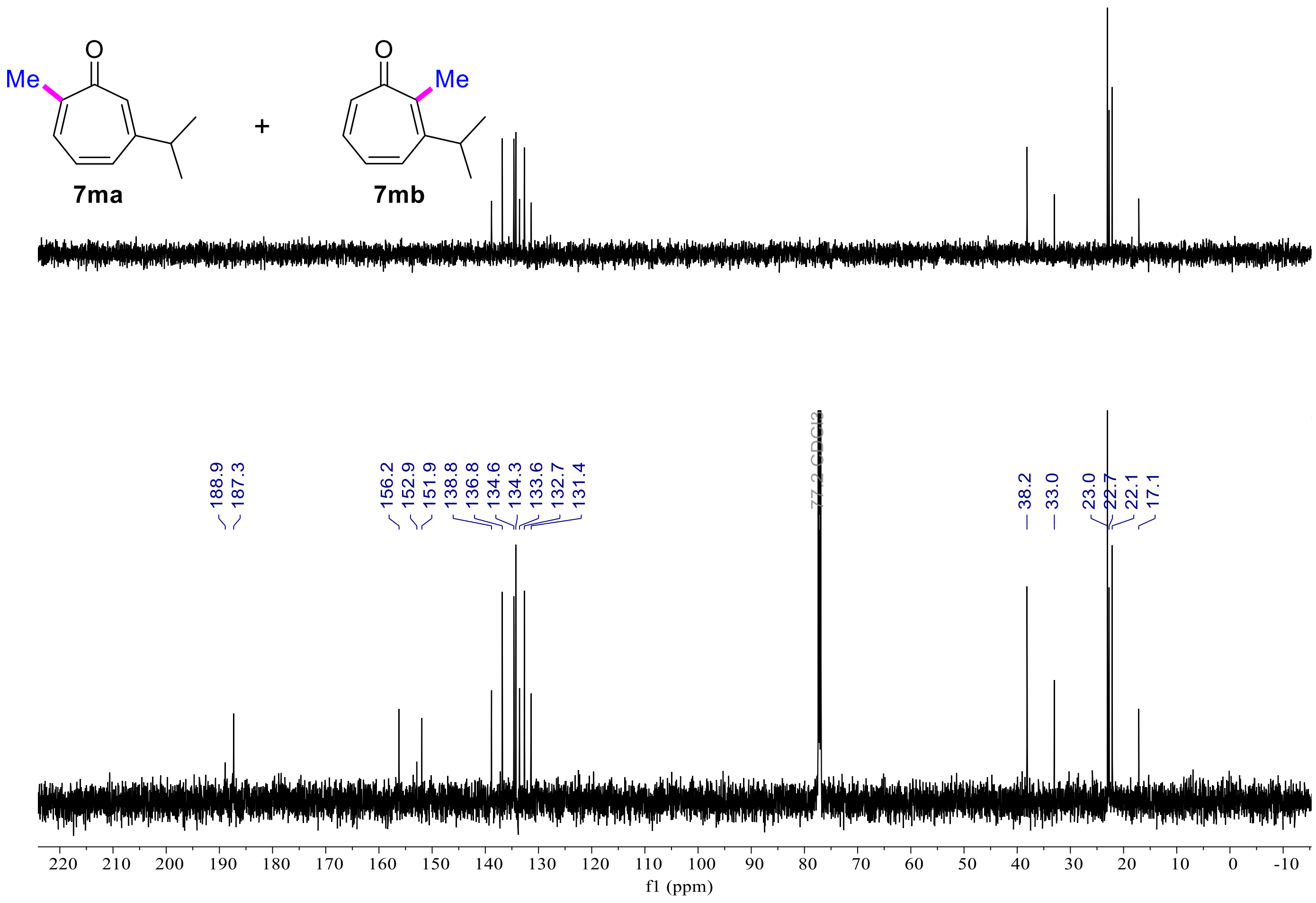

Compound 7ma and 7mb HRMS (ESI-TOF)

**Data Filename**	ESIH202404637.d	**Sample Name**	D4-ZQT18-41
**Sample ID**		**Position**	P1-E3
**Instrument Name**	Agilent 6520 Q-TOF	**Acq Method**	20160322_MS_ESIH_POS_1min.m
**Acquired Time**	9/23/2024 3:46:38 PM	**DA Method**	ESI-HR-20231114.m
**Comment**	ESIH by fangsu		

User Spectra

Fragmentor Voltage	Collision Energy	Ionization Mode
170	0	ESI

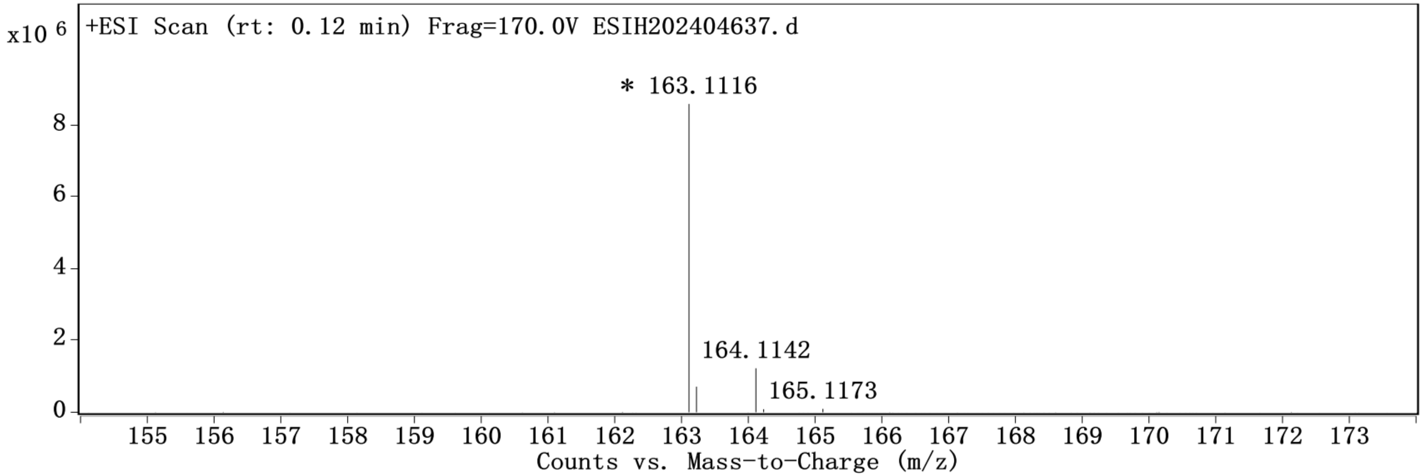

Formula Calculator Results

m/z	Calc m/z	Diff (mDa)	Diff (ppm)	Ion Formula	Ion
163.1116	163.1117	0.12	0.73	C11 H15 O	(M+H)+

--- End Of Report ---

Compound 7n ^{1}H NMR (600 MHz, $CDCl_3$)

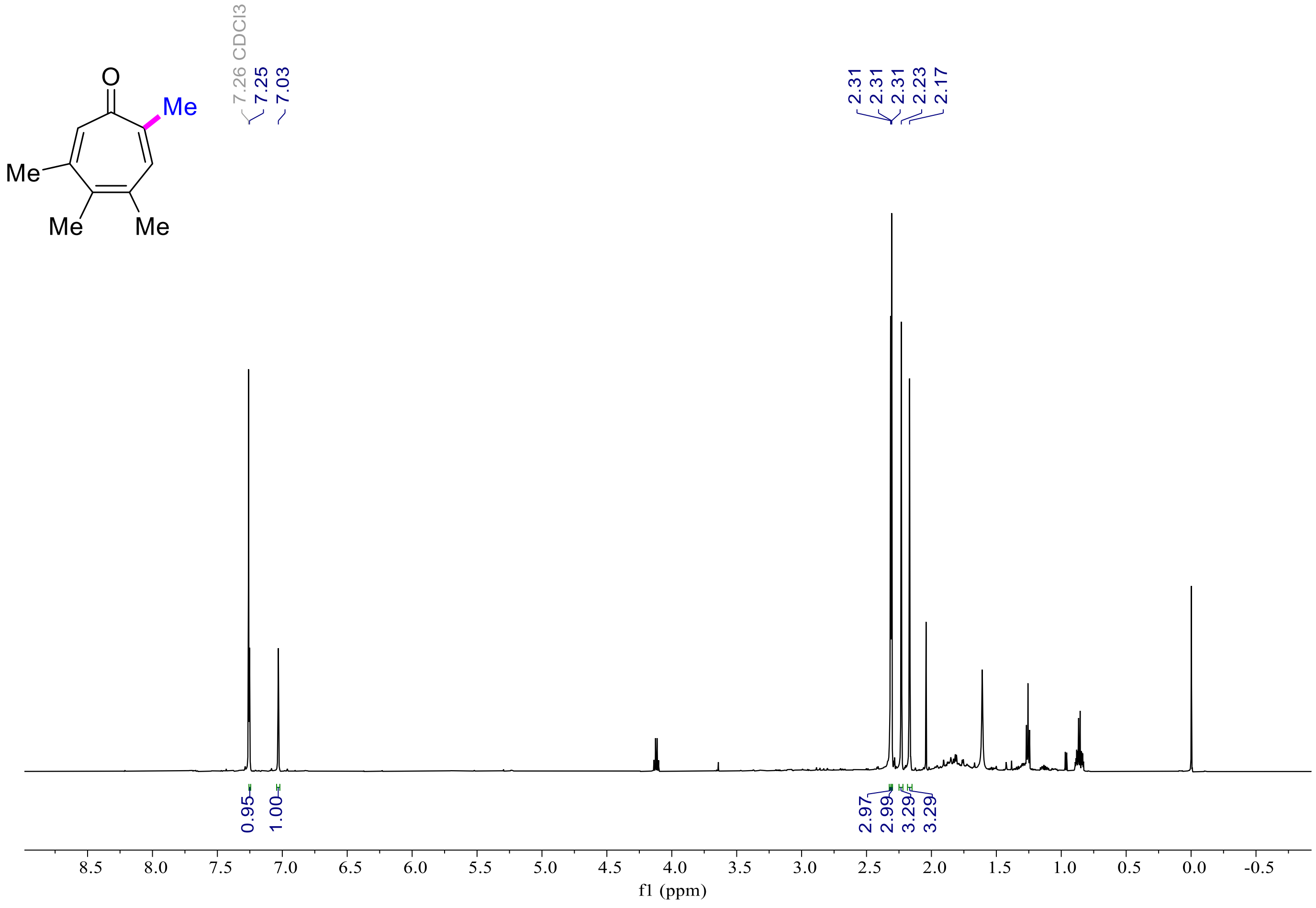

Compound 7n ^{13}C NMR (125 MHz, $CDCl_3$)

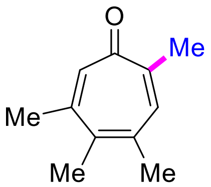

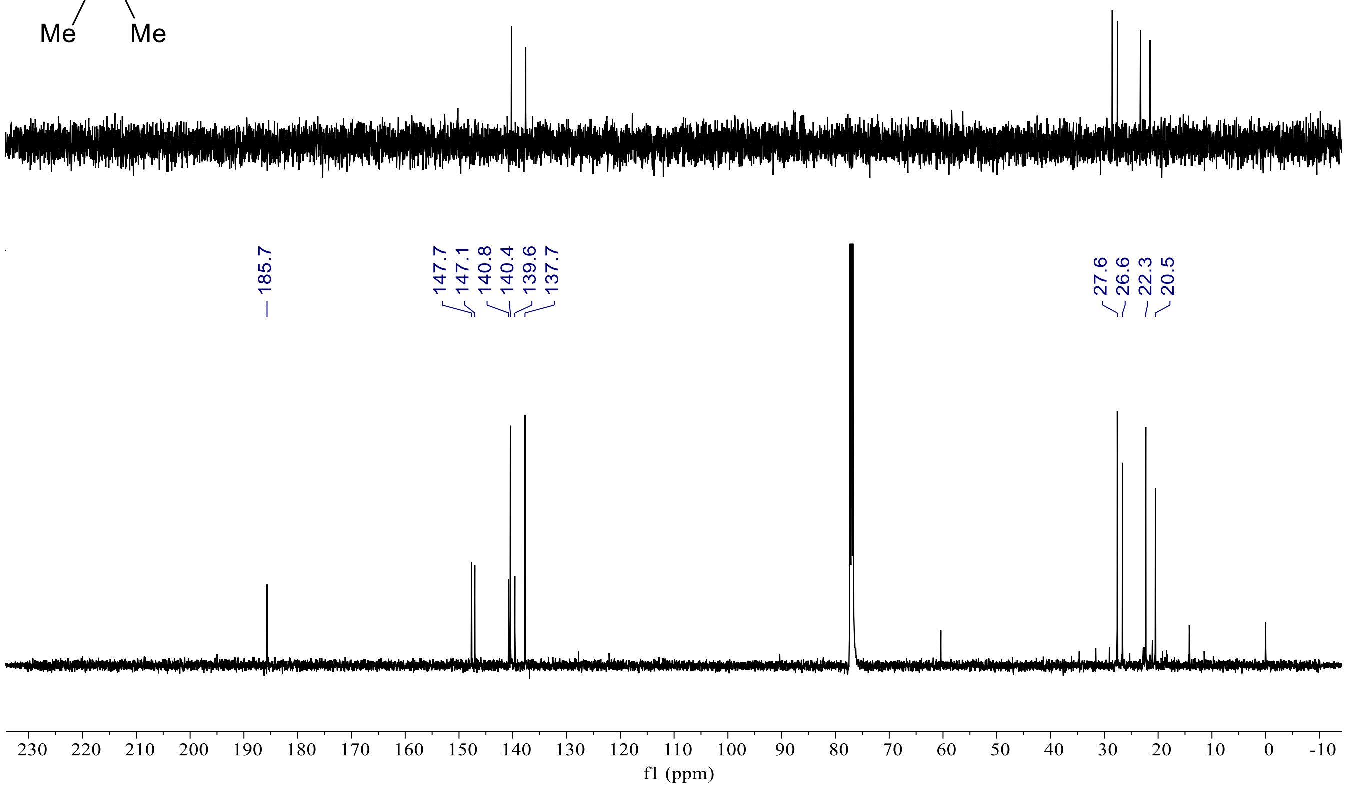

Compound 7n HRMS (ESI-TOF)

**Data Filename**	ESIH202501964.d	**Sample Name**	D4-ZQriv14
**Sample ID**		**Position**	P1-A5
**Instrument Name**	Agilent 6520 Q-TOF	**Acq Method**	20160322_MS_ESIH_POS_1min.m
**Acquired Time**	3/21/2025 4:07:17 PM	**DA Method**	ESI-HR-20231114.m
**Comment**	ESIH by fangsu		

User Spectra

Fragmentor Voltage	Collision Energy	Ionization Mode
170	0	ESI

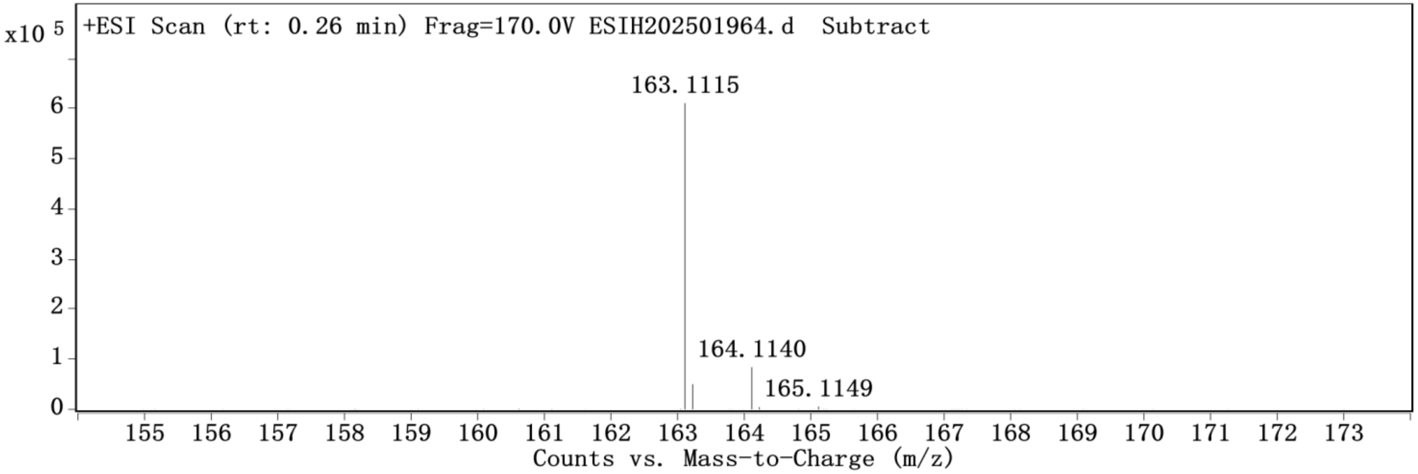

Formula Calculator Results

m/z	Calc m/z	Diff (mDa)	Diff (ppm)	Ion Formula	Ion
163.1115	163.1117	0.25	1.55	C11 H15 O	(M+H)+

--- End Of Report ---

Compound 7o ^{1}H NMR (600 MHz, $CDCl_3$)

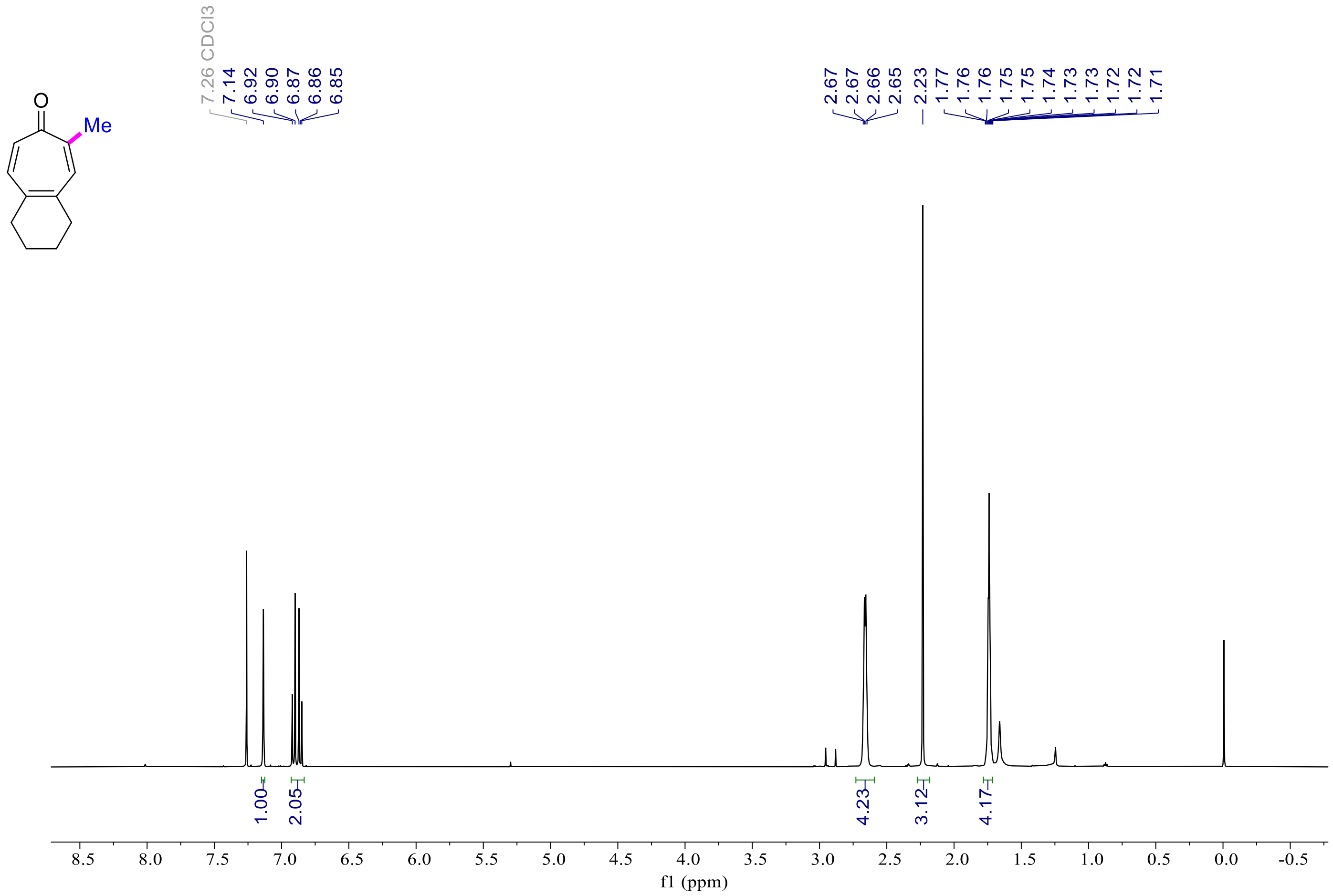

Compound 7o ^{13}C NMR (125 MHz, $CDCl_3$)

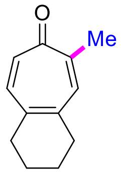

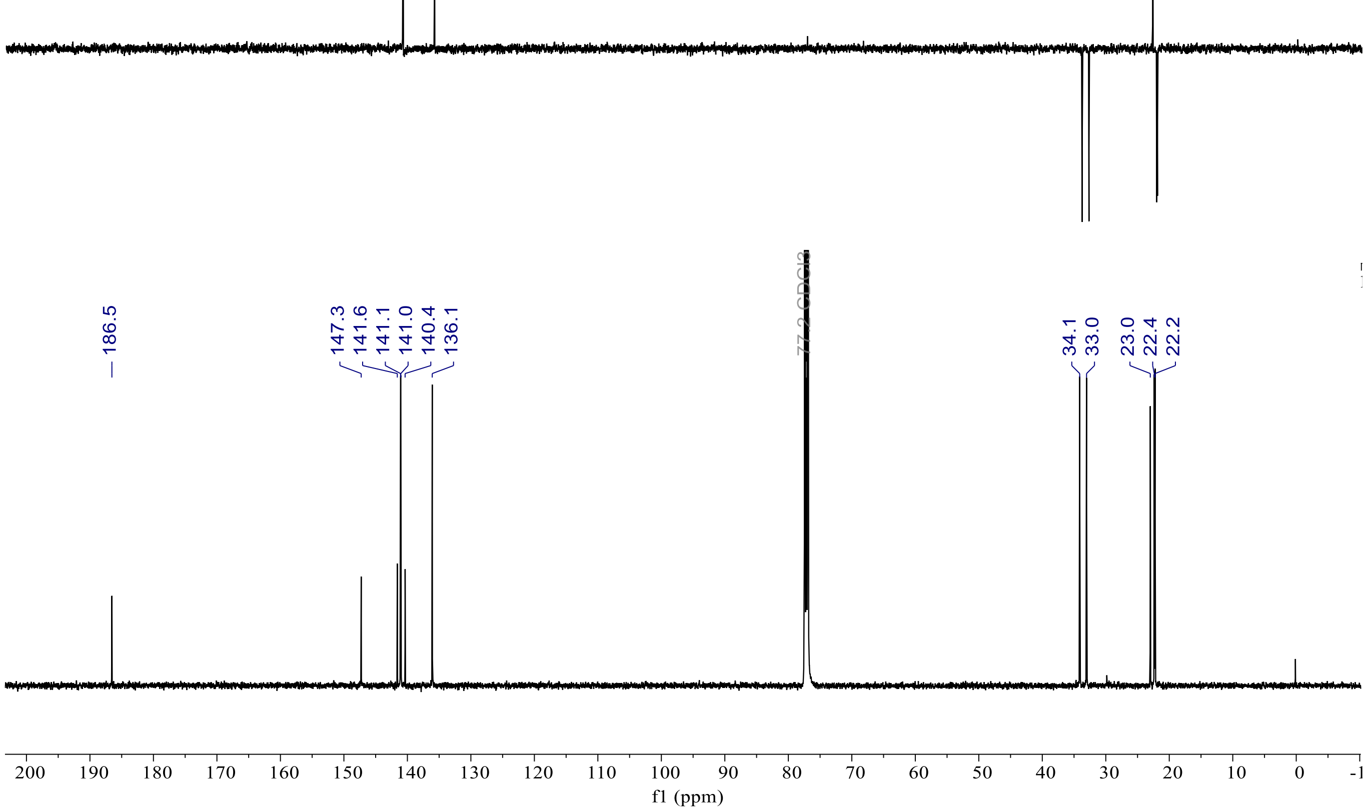

Compound 7o HRMS (ESI-TOF)

**Data Filename**	ESIH202501759.d	**Sample Name**	D4-ZQriv1
**Sample ID**		**Position**	P1-A3
**Instrument Name**	Agilent 6520 Q-TOF	**Acq Method**	20160322_MS_ESIH_POS_1min.m
**Acquired Time**	3/13/2025 4:26:33 PM	**DA Method**	ESI-HR-20231114.m
**Comment**	ESIH by fangsu		

User Spectra

Fragmentor Voltage	Collision Energy	Ionization Mode
170	0	ESI

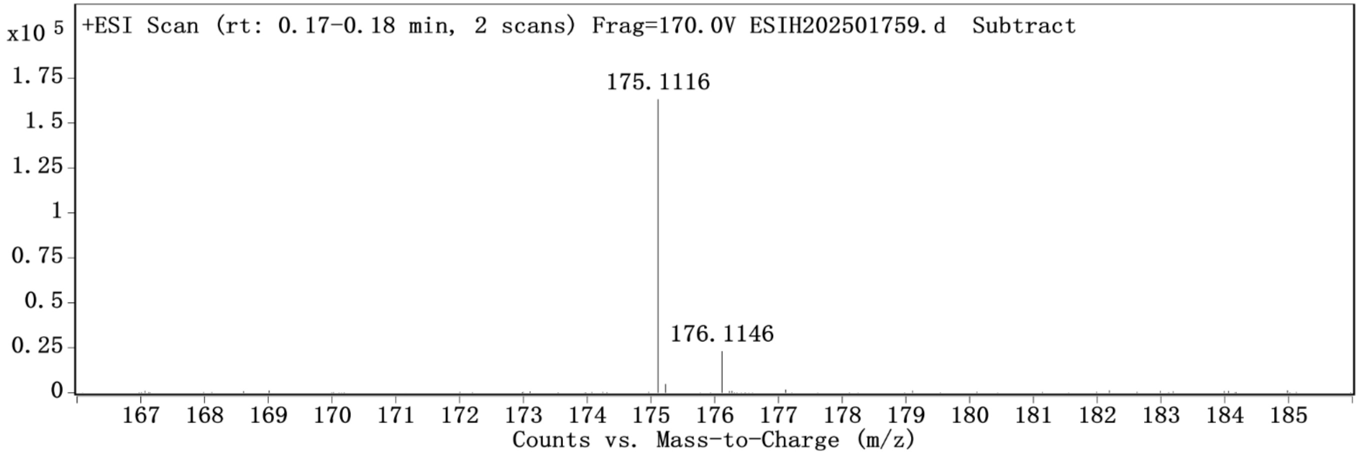

Formula Calculator Results

m/z	Calc m/z	Diff (mDa)	Diff (ppm)	Ion Formula	Ion
175.1116	175.1117	0.16	0.92	C12 H15 O	(M+H)+

--- End Of Report ---

Compound 7pa ^{1}H NMR (600 MHz, $CDCl_3$)

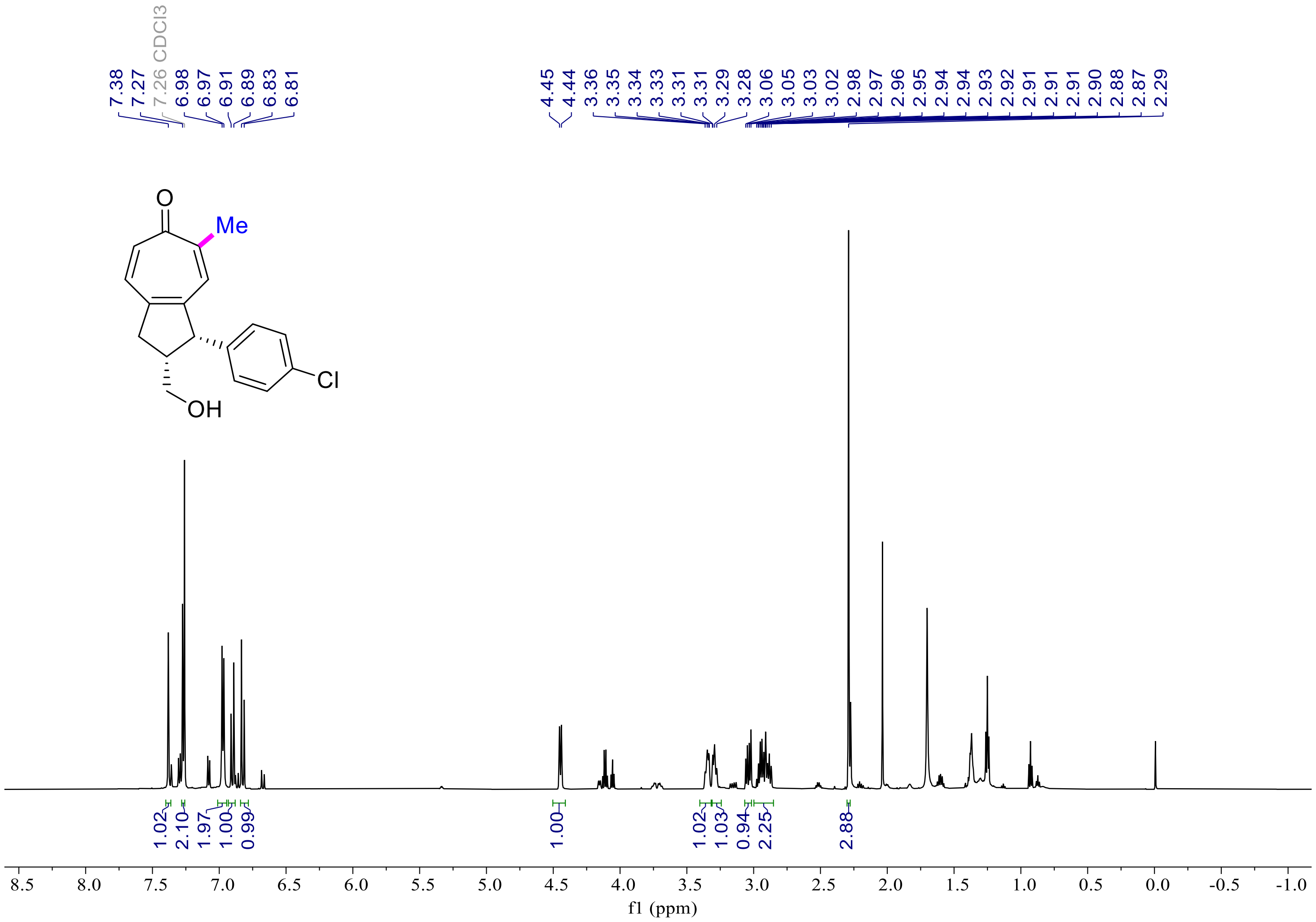

Compound 7pa ^{13}C NMR (125 MHz, $CDCl_3$)

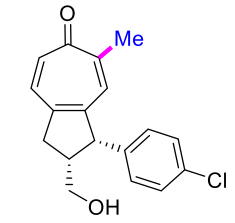

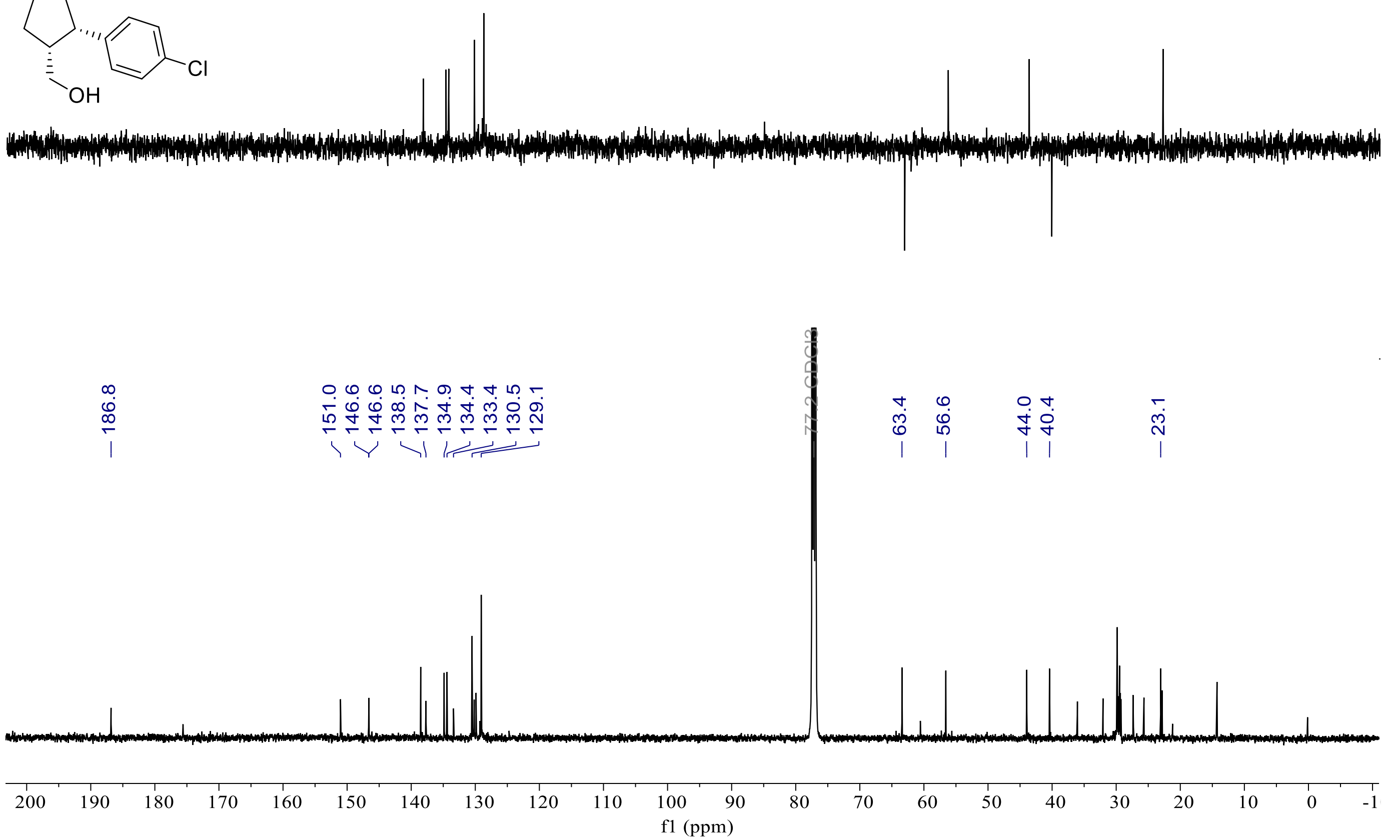

Compound 7pa HRMS (ESI-TOF)

**Data Filename**	ESIH202404186.d	**Sample Name**	D4-ZQT18-3A
**Sample ID**		**Position**	P1-B1
**Instrument Name**	Agilent 6520 Q-TOF	**Acq Method**	20160322_MS_ESIH_POS_1min.m
**Acquired Time**	8/28/2024 3:50:11 PM	**DA Method**	ESI-HR-20231114.m
**Comment**	ESIH by fangsu		

User Spectra

Fragmentor Voltage	Collision Energy	Ionization Mode
170	0	ESI

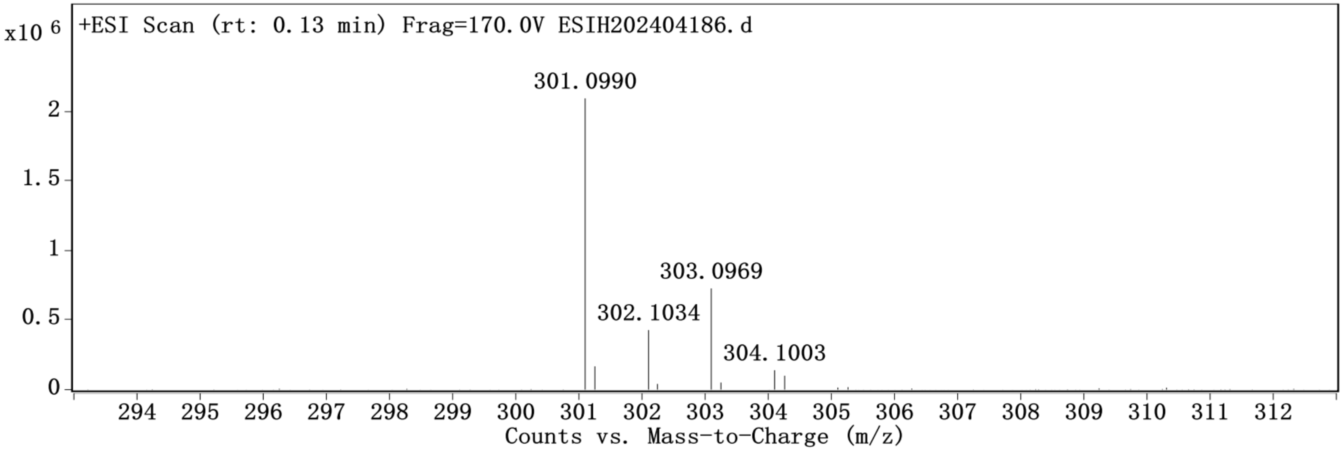

Formula Calculator Results

m/z	Calc m/z	Diff (mDa)	Diff (ppm)	Ion Formula	Ion
301.099	301.099	-0.06	-0.2	C18 H18 Cl O2	(M+H)+

--- End Of Report ---

Compound 7pb ^{1}H NMR (600 MHz, $CDCl_3$)

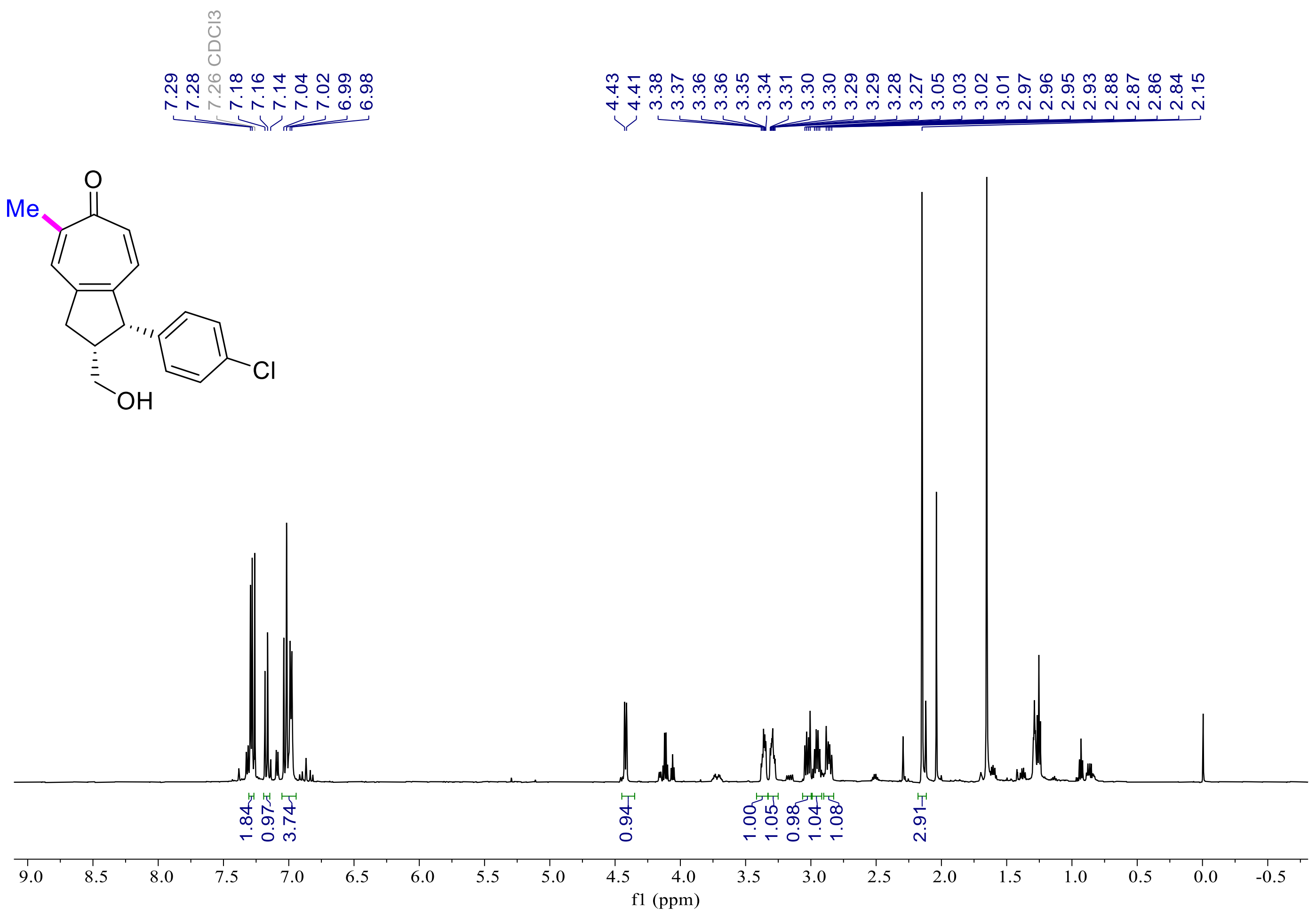

Compound 7pb ^{13}C NMR (125 MHz, $CDCl_3$)

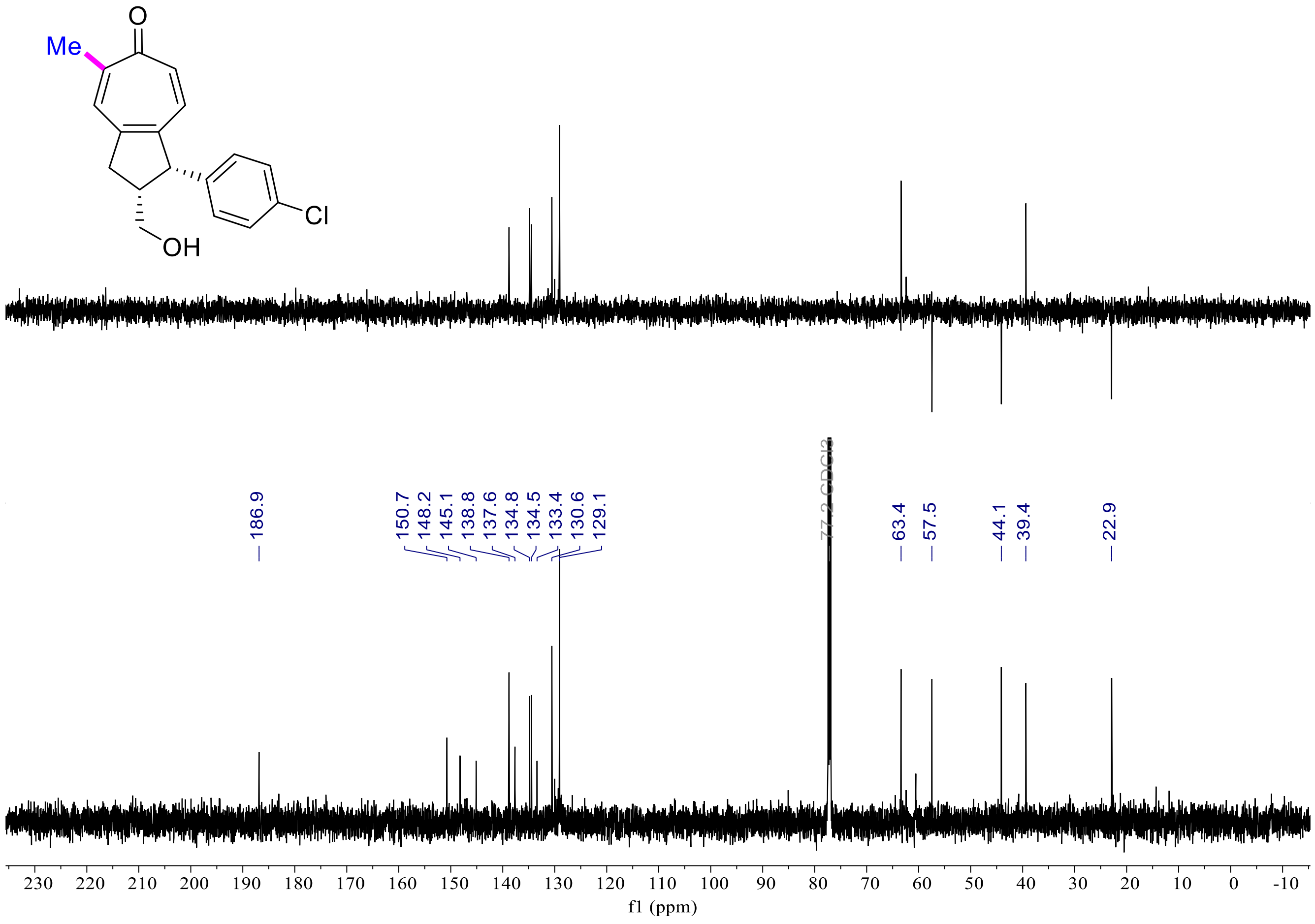

Compound 7pb HRMS (ESI-TOF)

**Data Filename**	ESIH202404187.d	**Sample Name**	D4-ZQT18-3B
**Sample ID**		**Position**	P1-B2
**Instrument Name**	Agilent 6520 Q-TOF	**Acq Method**	20160322_MS_ESIH_POS_1min.m
**Acquired Time**	8/28/2024 3:51:28 PM	**DA Method**	ESI-HR-20231114.m
**Comment**	ESIH by fangsu		

User Spectra

Fragmentor Voltage	Collision Energy	Ionization Mode
170	0	ESI

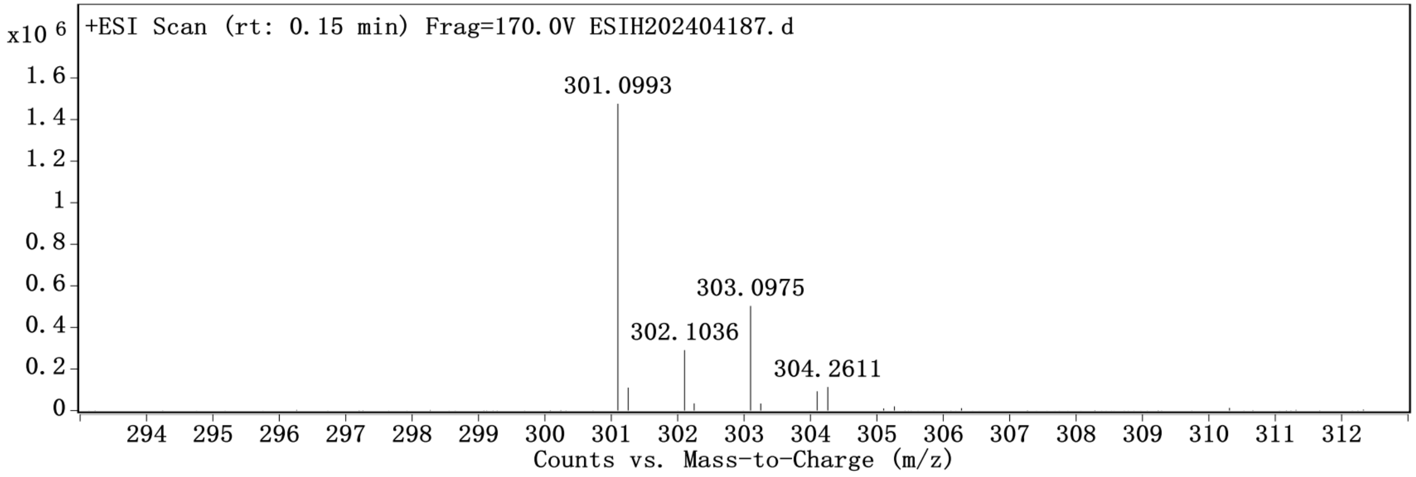

Formula Calculator Results

m/z	Calc m/z	Diff (mDa)	Diff (ppm)	Ion Formula	Ion
301.0993	301.099	-0.34	-1.12	C18 H18 Cl O2	(M+H)+

--- End Of Report ---

Compound 7q ^{1}H NMR (600 MHz, $CDCl_3$)

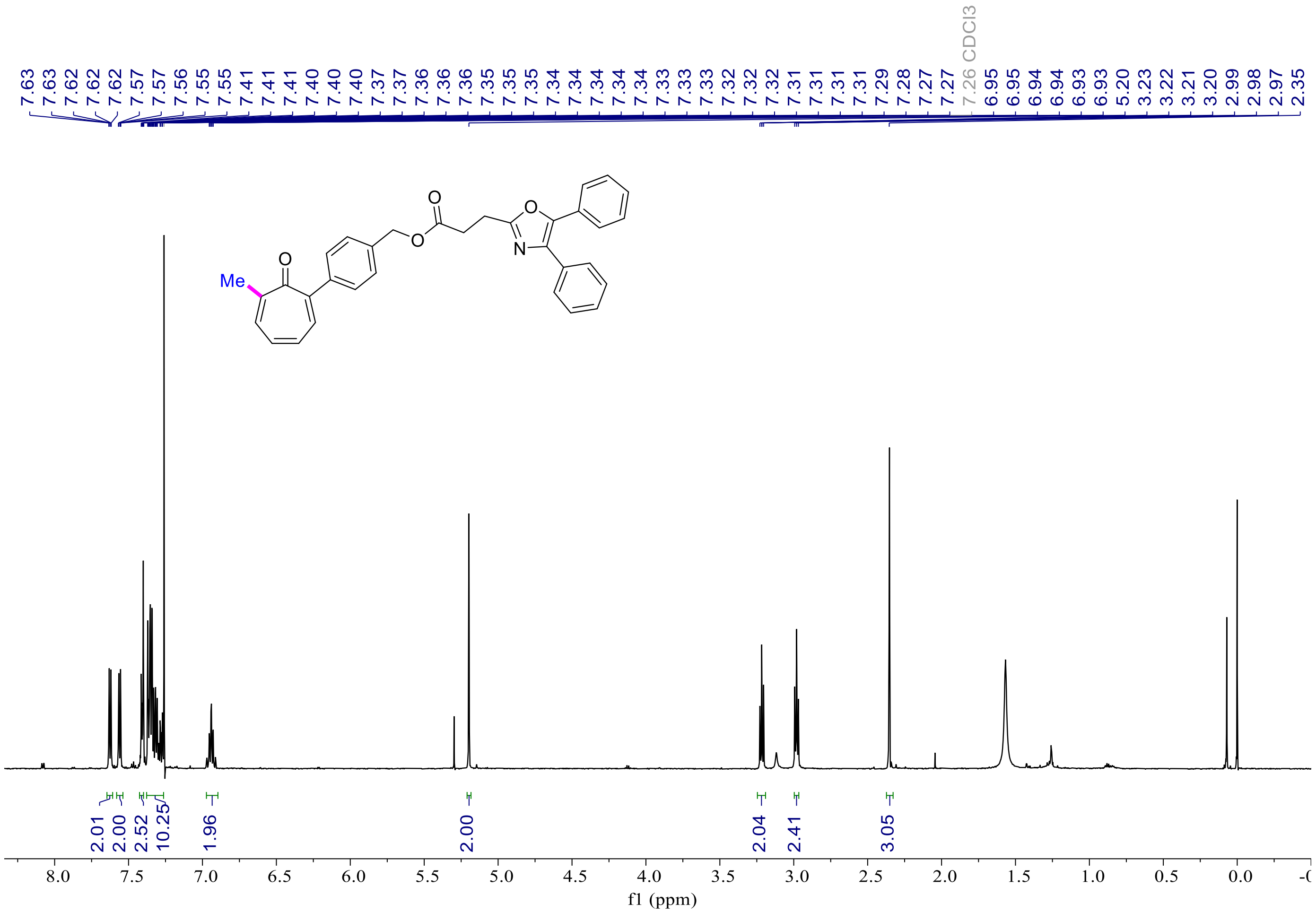

Compound 7q ^{13}C NMR (150 MHz, CDCl$_3$)

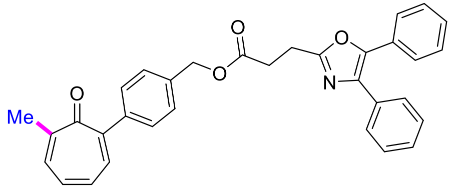

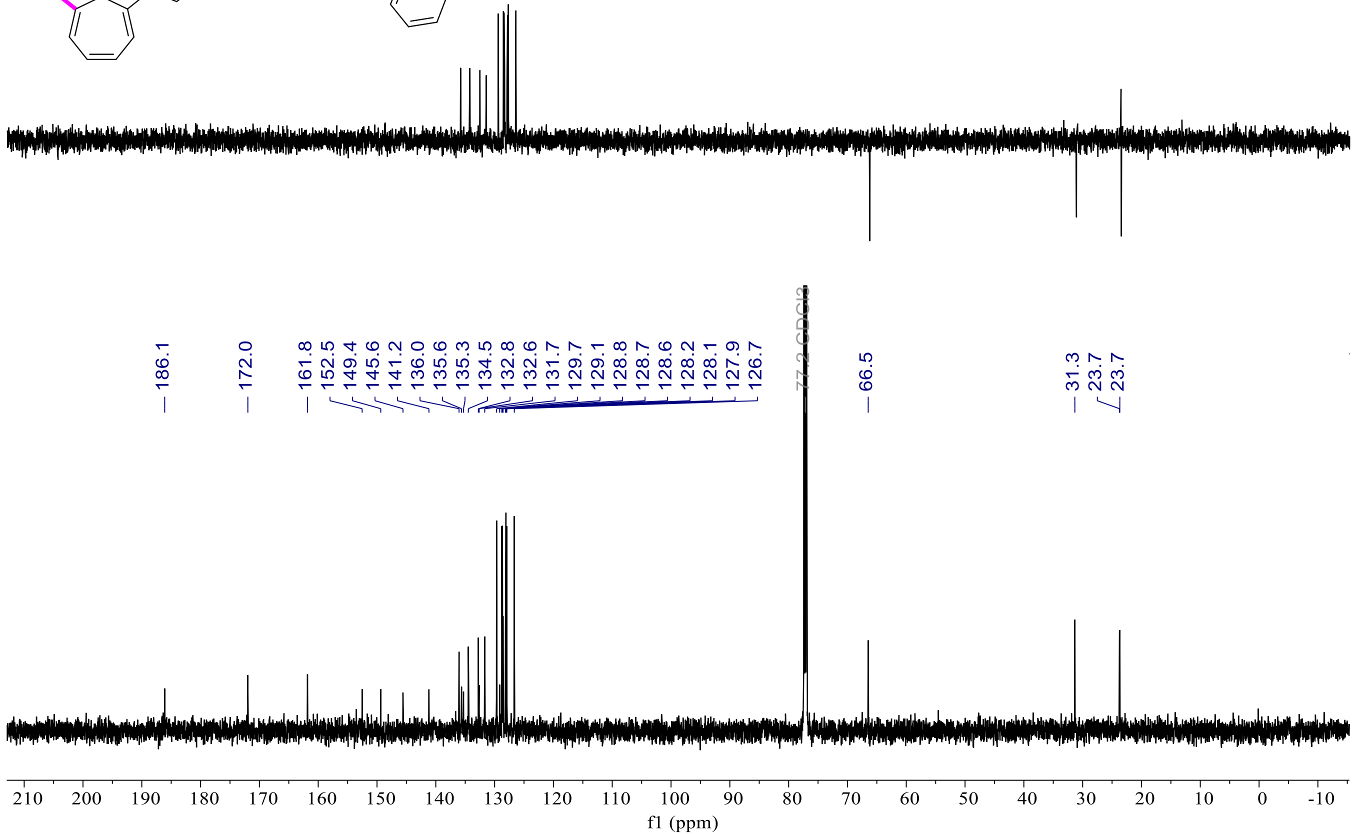

Compound 7q HRMS (ESI-TOF)

**Data Filename**	ESIH202405817.d	**Sample Name**	D4-ZDG4-1
**Sample ID**		**Position**	P1-E4
**Instrument Name**	Agilent 6520 Q-TOF	**Acq Method**	20160322_MS_ESIH_POS_1min.m
**Acquired Time**	12/6/2024 11:03:25 AM	**DA Method**	ESI-HR-20231114.m
**Comment**	ESIH by fangsu		

User Spectra

Fragmentor Voltage	Collision Energy	Ionization Mode
170	0	ESI

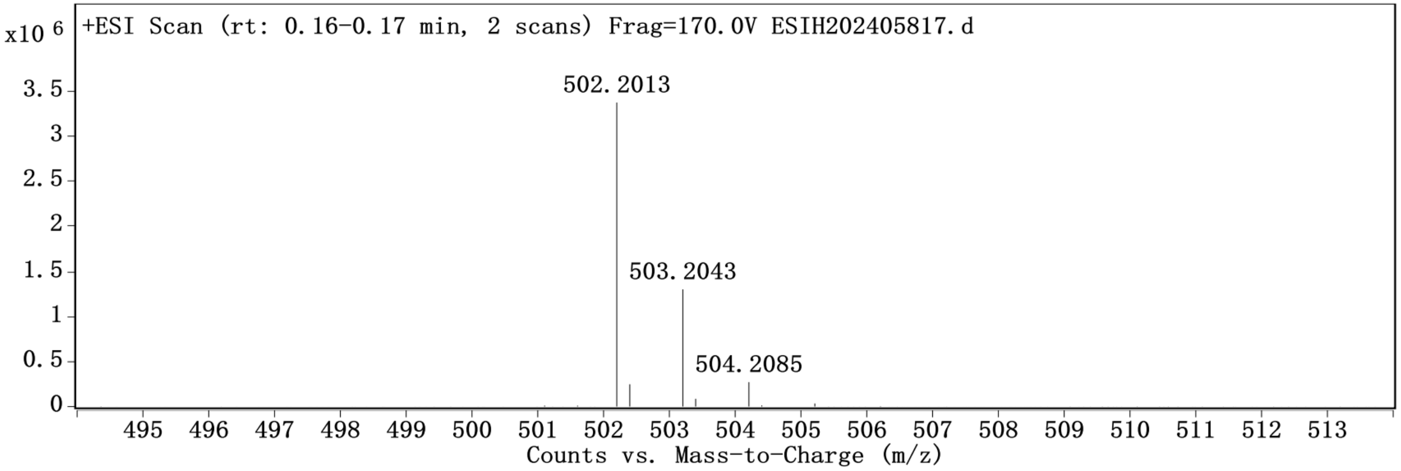

Formula Calculator Results

m/z	Calc m/z	Diff (mDa)	Diff (ppm)	Ion Formula	Ion
502.2013	502.2013	-0.06	-0.11	C33 H28 N O4	(M+H)+

--- End Of Report ---

Compound 7r ^{1}H NMR (600 MHz, $CDCl_3$)

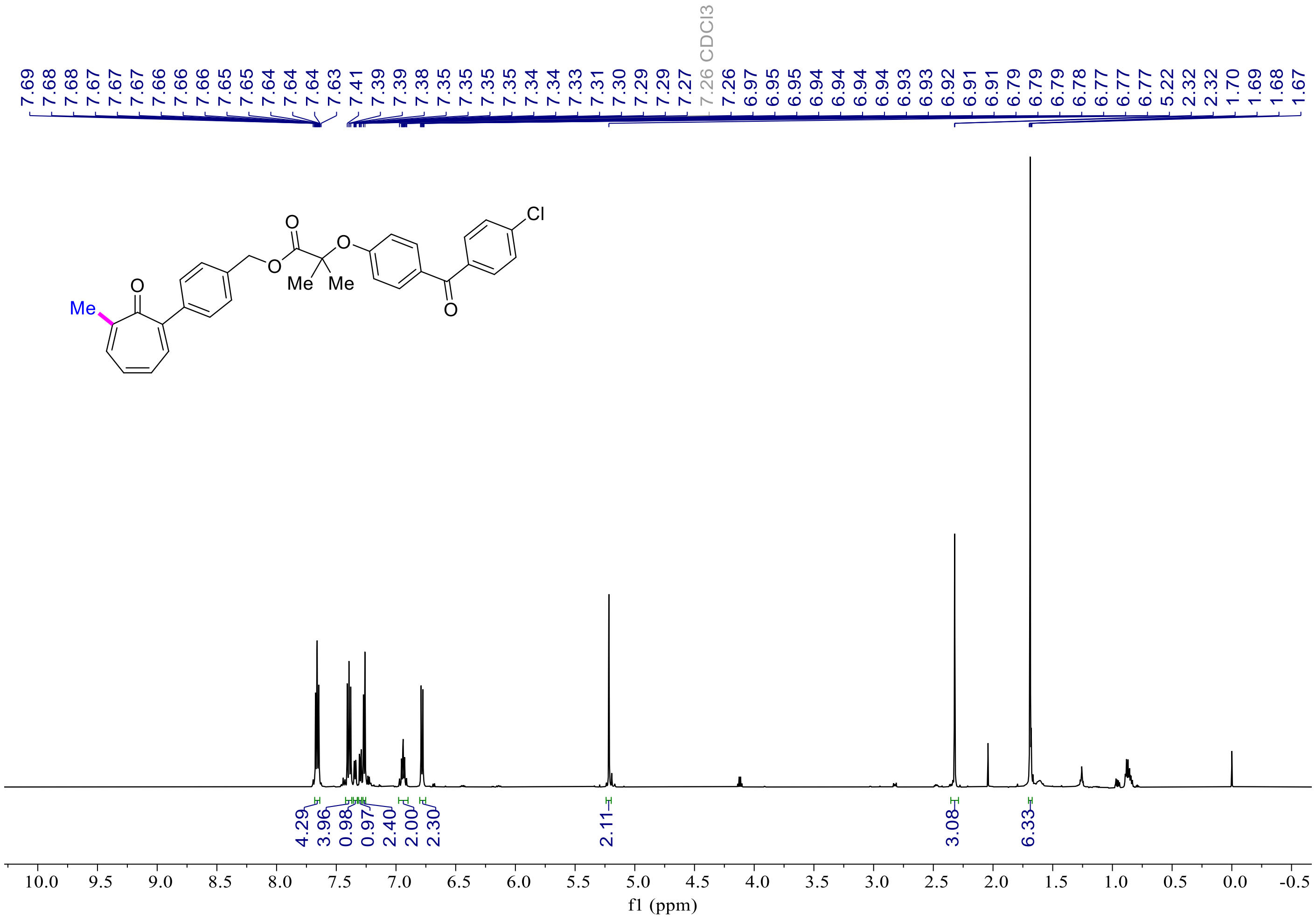

Compound 7r ^{13}C NMR (150 MHz, $CDCl_3$)

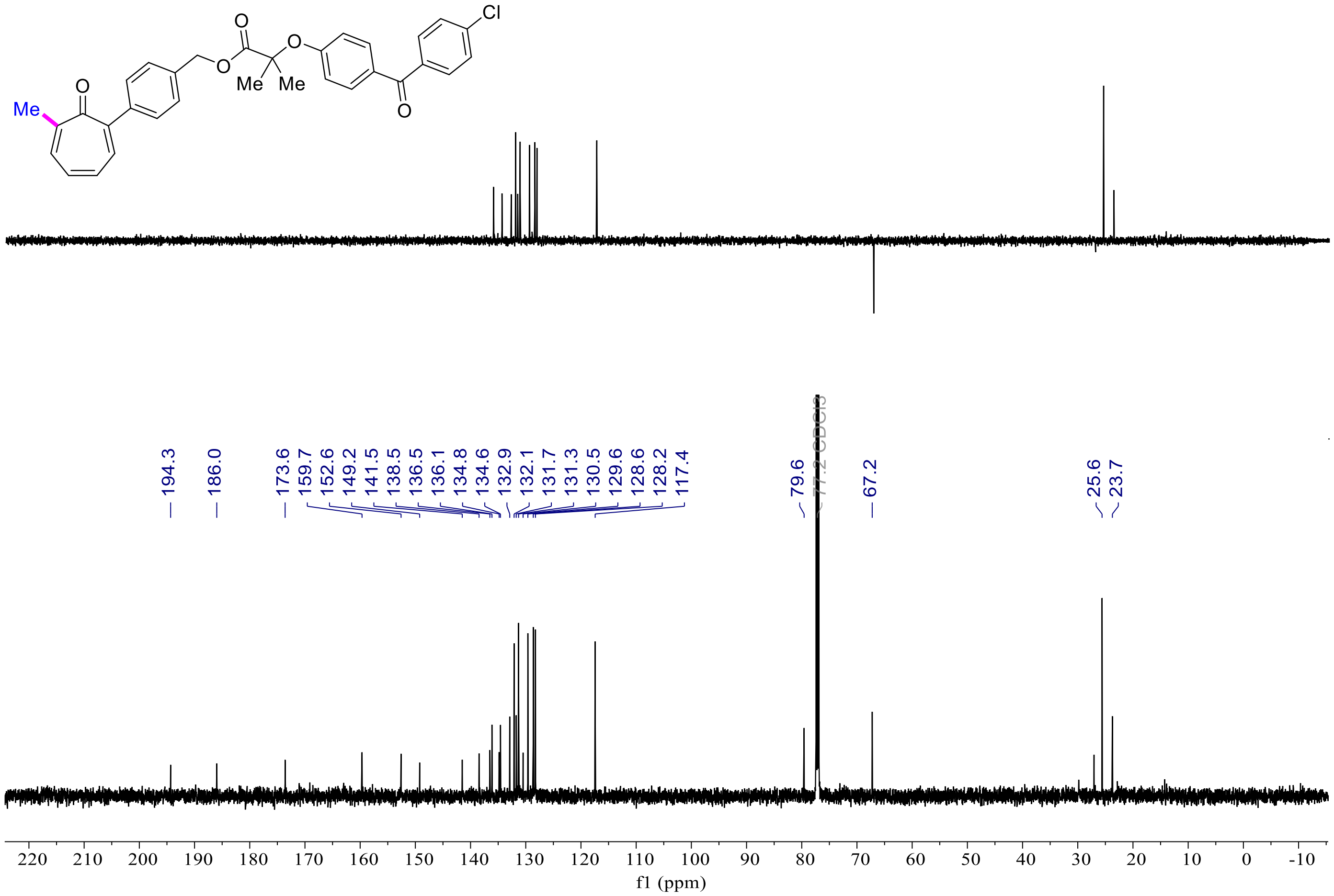

Compound 7r HRMS (ESI-TOF)

**Data Filename**	ESIH202405818.d	**Sample Name**	D4-ZDG4-2
**Sample ID**		**Position**	P1-E5
**Instrument Name**	Agilent 6520 Q-TOF	**Acq Method**	20160322_MS_ESIH_POS_1min.m
**Acquired Time**	12/6/2024 11:04:41 AM	**DA Method**	ESI-HR-20231114.m
**Comment**	ESIH by fangsu		

User Spectra

Fragmentor Voltage	Collision Energy	Ionization Mode
170	0	ESI

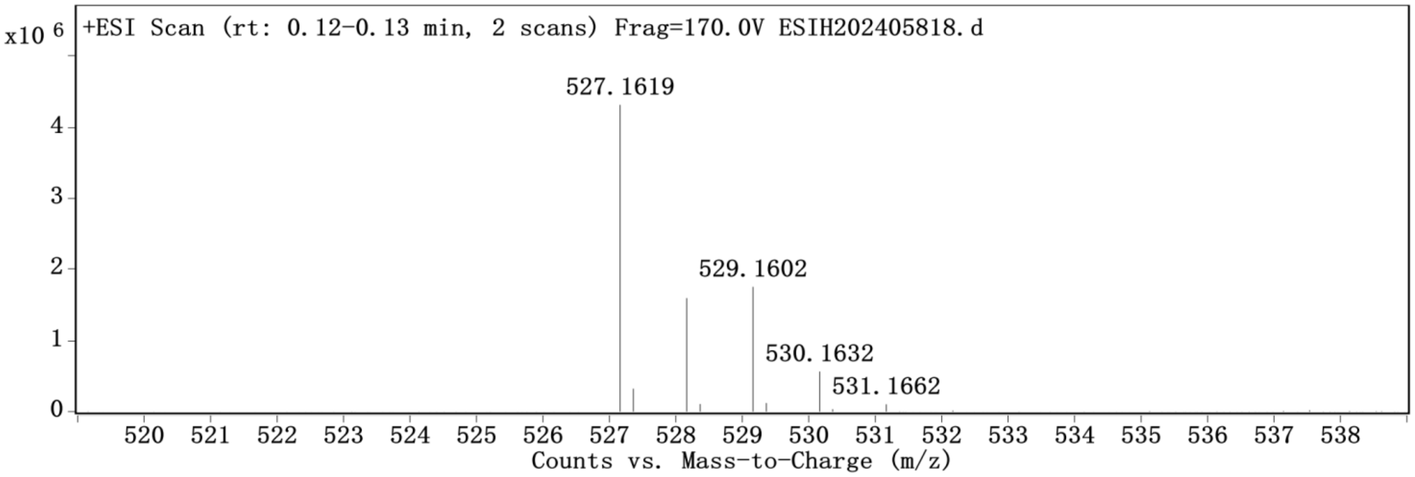

Formula Calculator Results

m/z	Calc m/z	Diff (mDa)	Diff (ppm)	Ion Formula	Ion
527.1619	527.162	0.1	0.18	C32 H28 Cl O5	(M+H)+

--- End Of Report ---

Compound 7s ^{1}H NMR (600 MHz, $CDCl_3$)

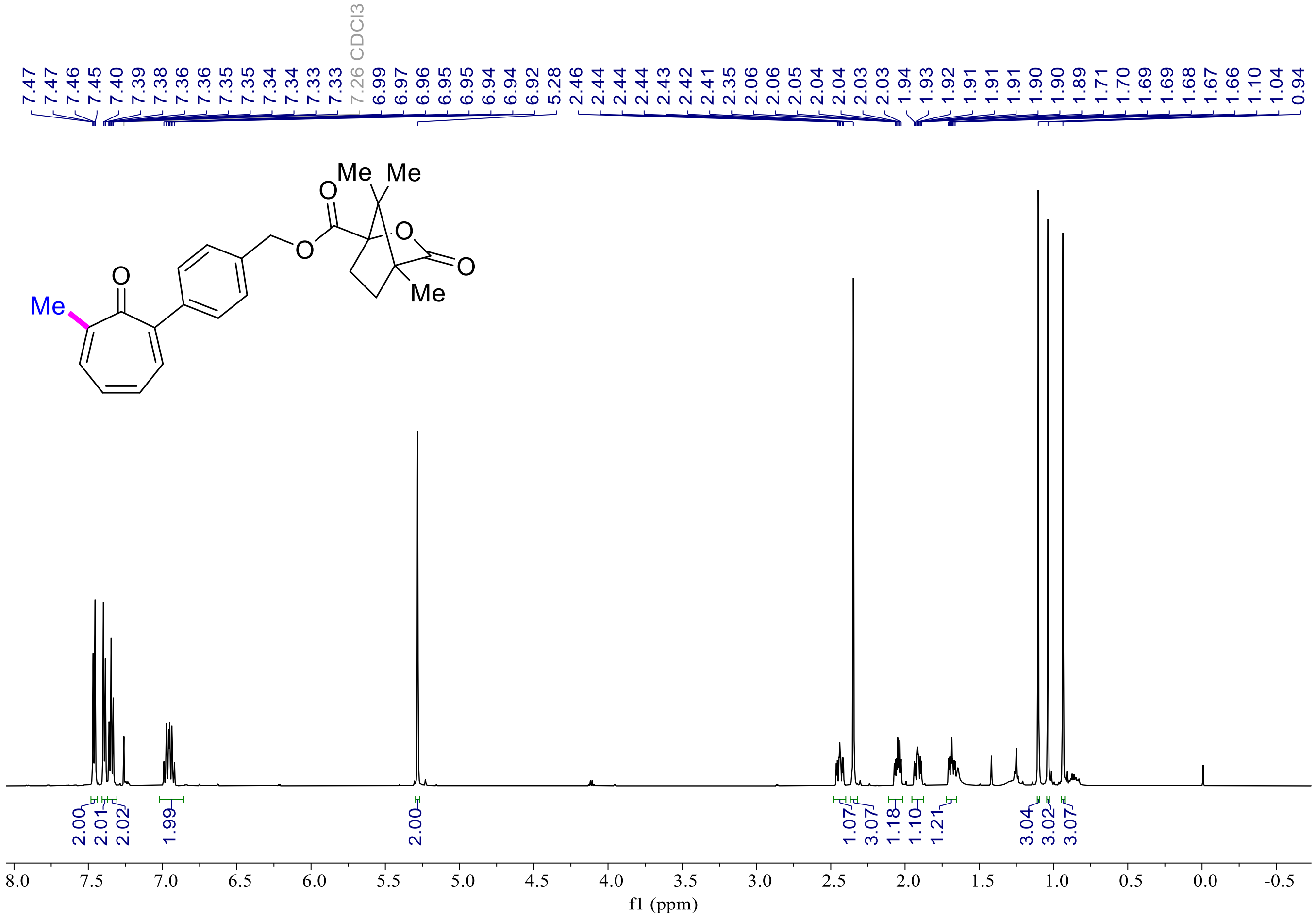

Compound 7s ^{13}C NMR (150 MHz, CDCl$_3$)

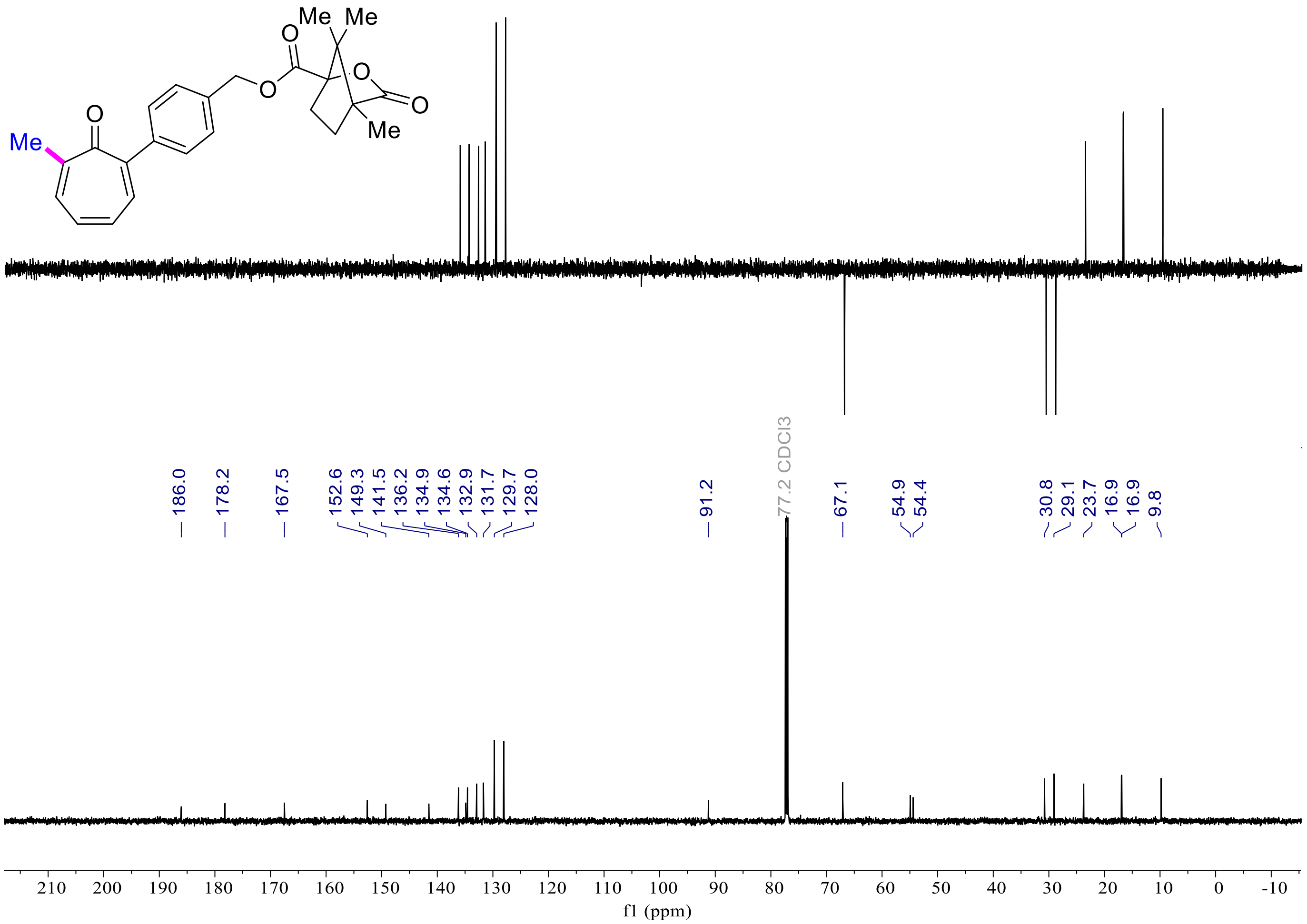

Compound 7s HRMS (ESI-TOF)

**Data Filename**	ESIH202405823.d	**Sample Name**	D4-ZDG4-3
**Sample ID**		**Position**	P1-F1
**Instrument Name**	Agilent 6520 Q-TOF	**Acq Method**	20160322_MS_ESIH_POS_1min.m
**Acquired Time**	12/6/2024 11:11:04 AM	**DA Method**	ESI-HR-20231114.m
**Comment**	ESIH by fangsu		

User Spectra

Fragmentor Voltage	Collision Energy	Ionization Mode
170	0	ESI

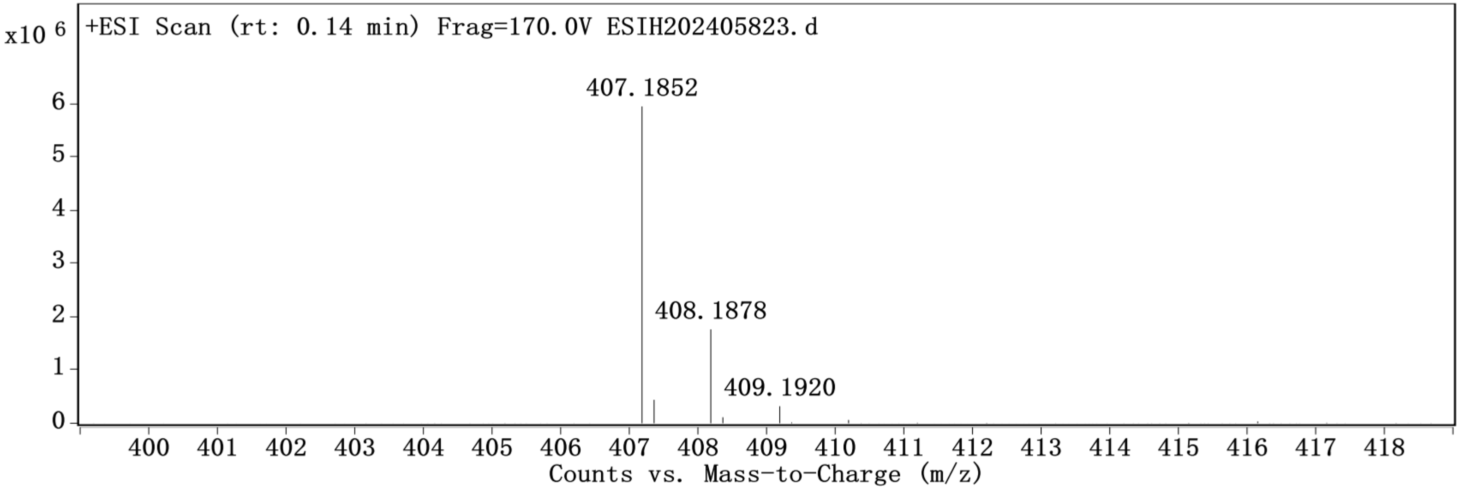

Formula Calculator Results

m/z	Calc m/z	Diff (mDa)	Diff (ppm)	Ion Formula	Ion
407.1852	407.1853	0.12	0.3	C25 H27 O5	(M+H)+

--- End Of Report ---

Compound 7t ^{1}H NMR (600 MHz, CDCl$_3$)

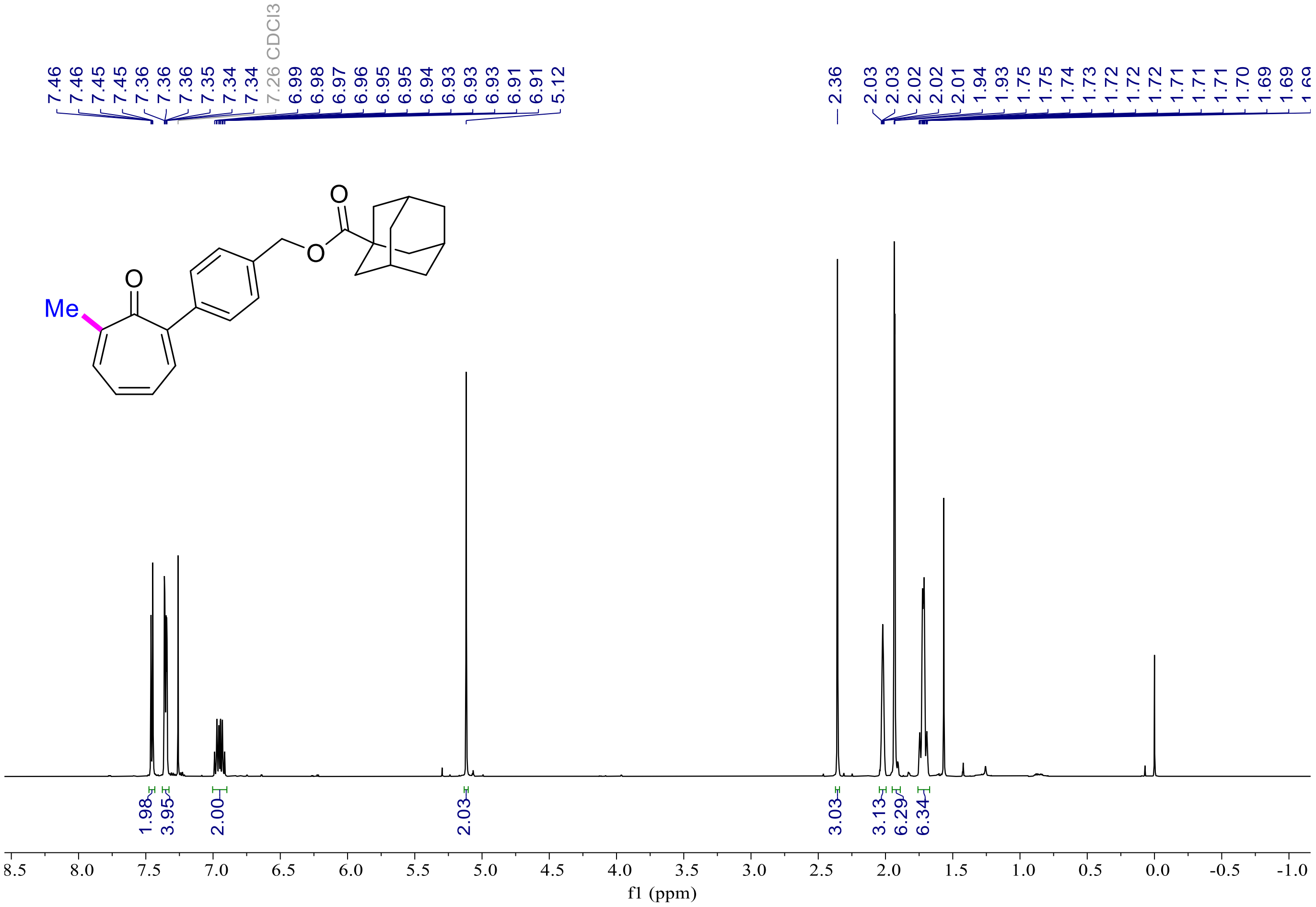

Compound 7t ^{13}C NMR (150 MHz, $CDCl_3$)

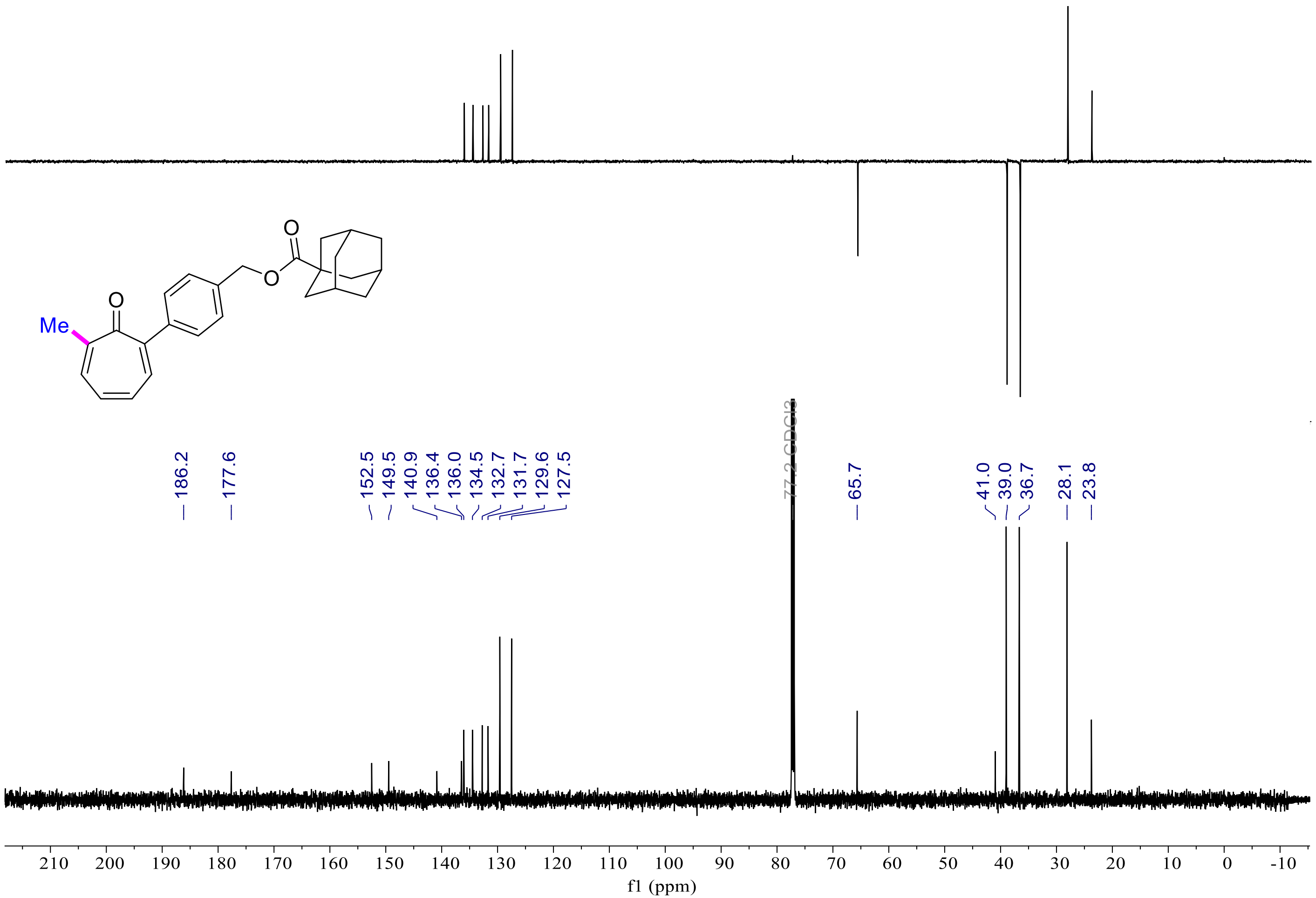

Compound 7t HRMS (ESI-TOF)

**Data Filename**	ESIH202405846.d	**Sample Name**	D4-ZDG4-4
**Sample ID**		**Position**	P1-A9
**Instrument Name**	Agilent 6520 Q-TOF	**Acq Method**	20160322_MS_ESIH_POS_1min.m
**Acquired Time**	12/9/2024 2:58:08 PM	**DA Method**	ESI-HR-20231114.m
**Comment**	ESIH by huangqiongping		

User Spectra

Fragmentor Voltage	Collision Energy	Ionization Mode
170	0	ESI

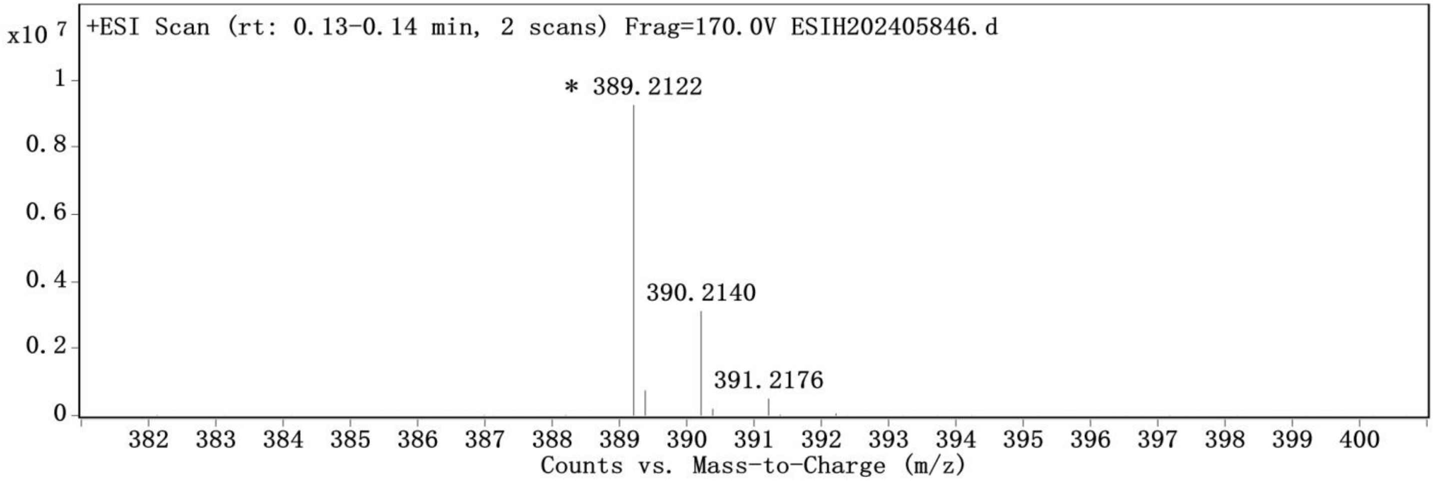

Formula Calculator Results

m/z	Calc m/z	Diff (mDa)	Diff (ppm)	Ion Formula	Ion
389.2122	389.2111	-1.09	-2.8	C26 H29 O3	(M+H)+

--- End Of Report ---

Compound 7u ^{1}H NMR (600 MHz, $CDCl_3$)

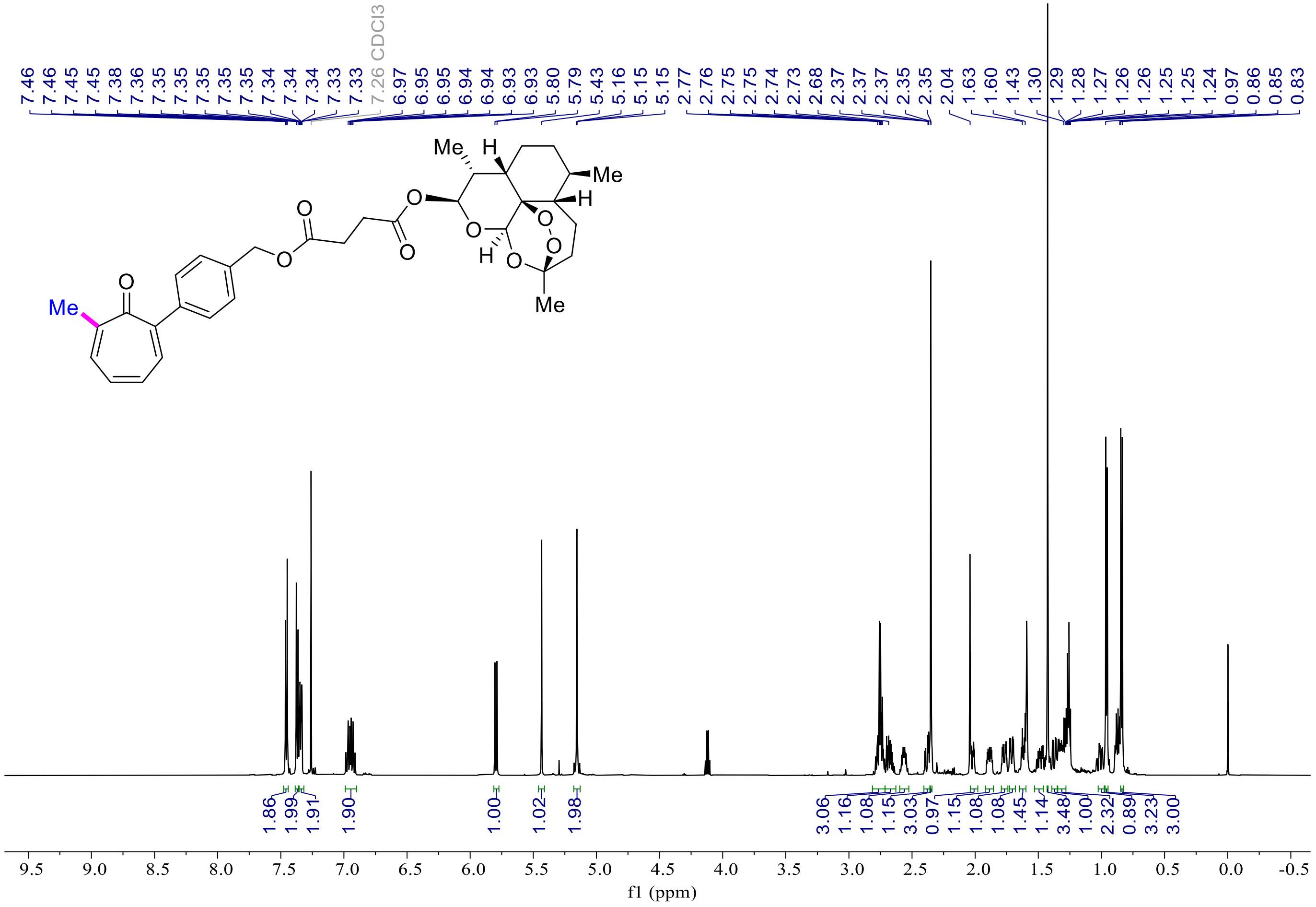

Compound 7u ^{13}C NMR (150 MHz, $CDCl_3$)

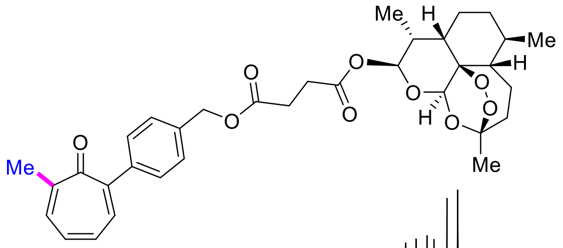

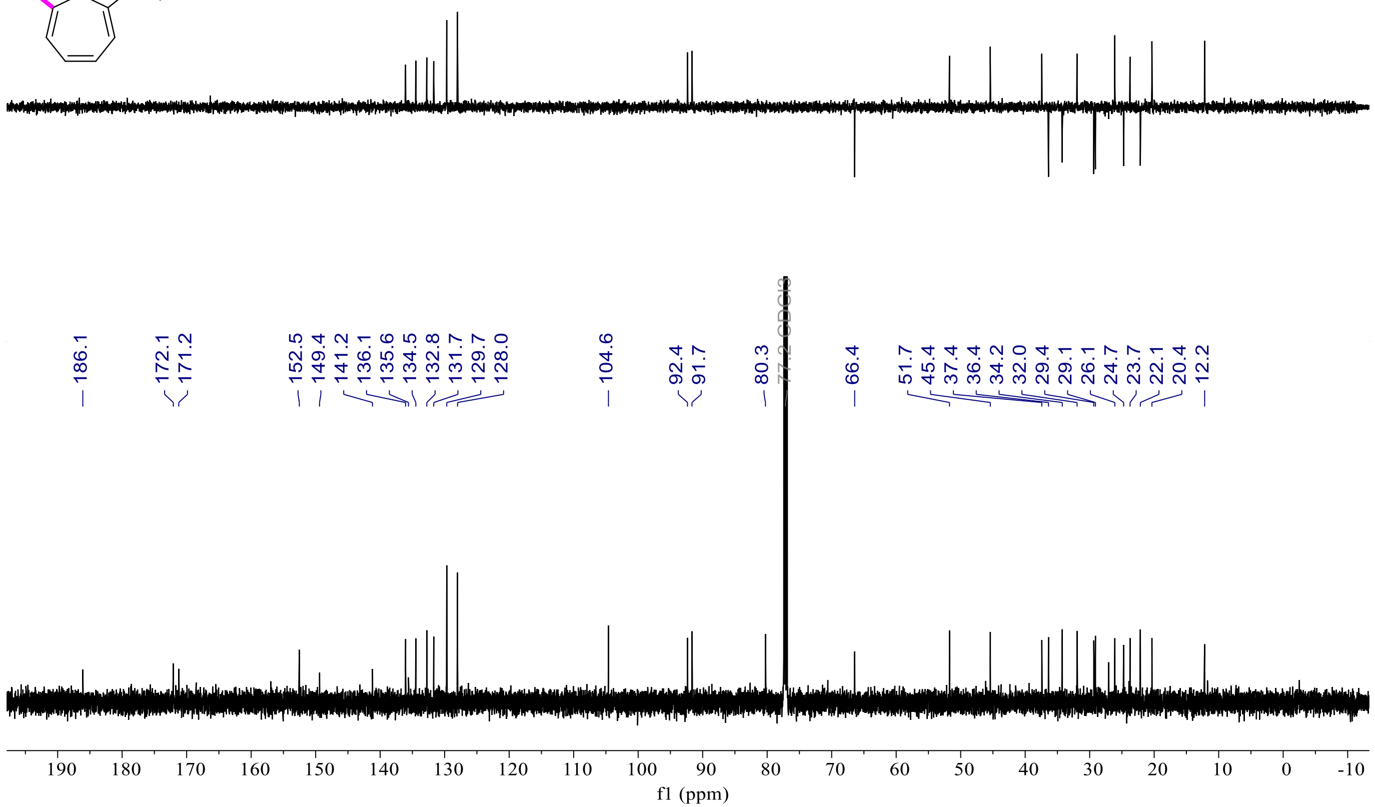

Compound 7u HRMS (ESI-TOF)

**Data Filename**	ESIH202405848.d	**Sample Name**	D4-ZDG4-5
**Sample ID**		**Position**	P1-B2
**Instrument Name**	Agilent 6520 Q-TOF	**Acq Method**	20160322_MS_ESIH_POS_1min.m
**Acquired Time**	12/9/2024 3:00:41 PM	**DA Method**	ESI-HR-20231114.m
**Comment**	ESIH by huangqiongping		

User Spectra

Fragmentor Voltage	Collision Energy	Ionization Mode
170	0	ESI

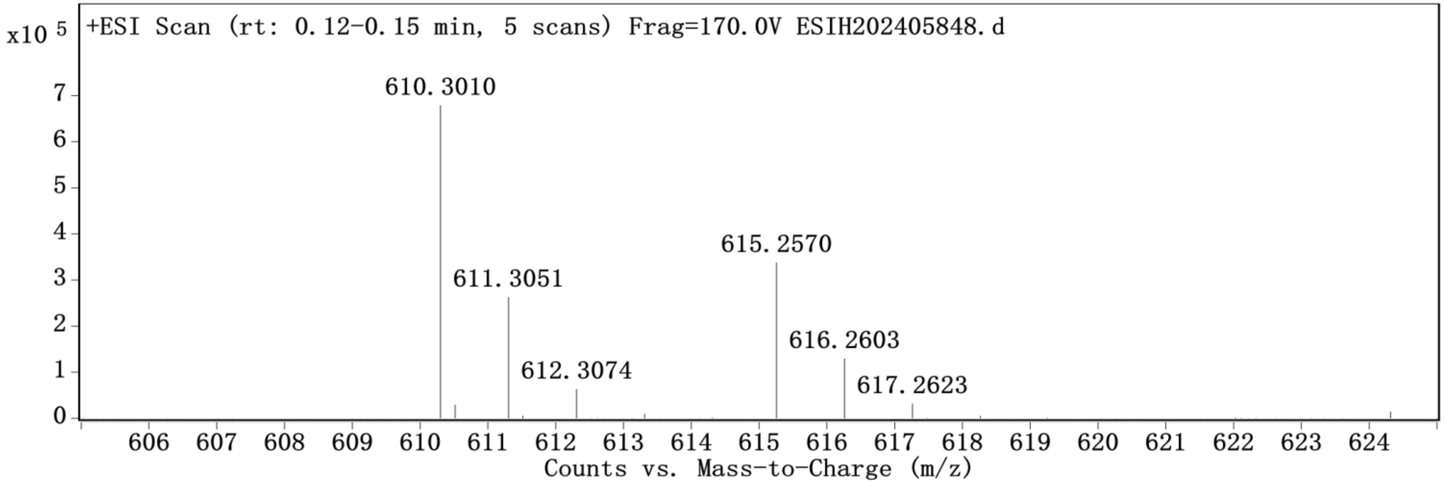

Formula Calculator Results

m/z	Calc m/z	Diff (mDa)	Diff (ppm)	Ion Formula	Ion
615.257	615.2565	-0.54	-0.88	C34 H40 Na O9	(M+Na)+
610.301	610.3011	0.02	0.03	C34 H44 N O9	(M+NH4)+

--- End Of Report ---

Compound 7v ^{1}H NMR (600 MHz, $CDCl_3$)

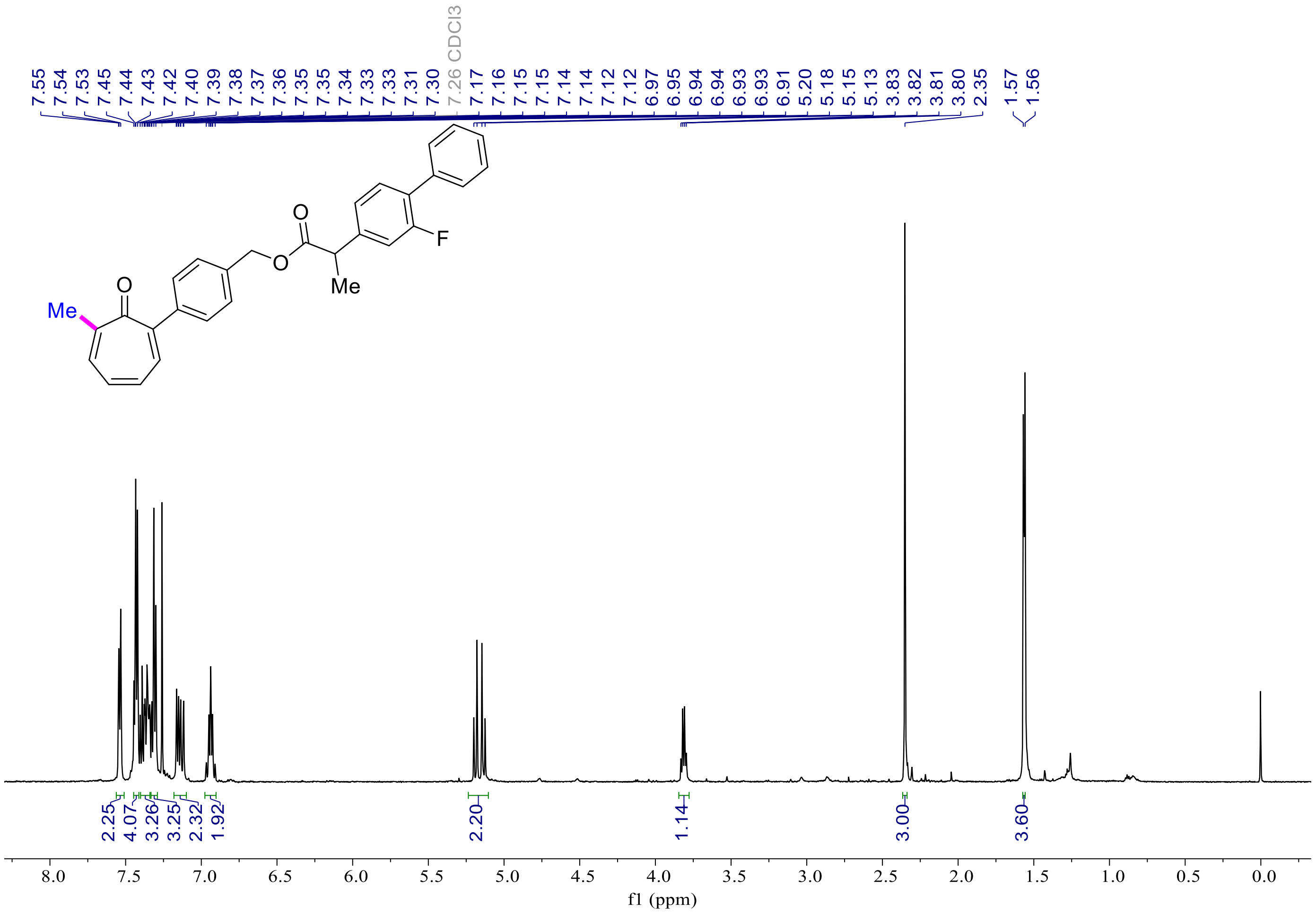

Compound 7v ^{13}C NMR (125 MHz, $CDCl_3$)

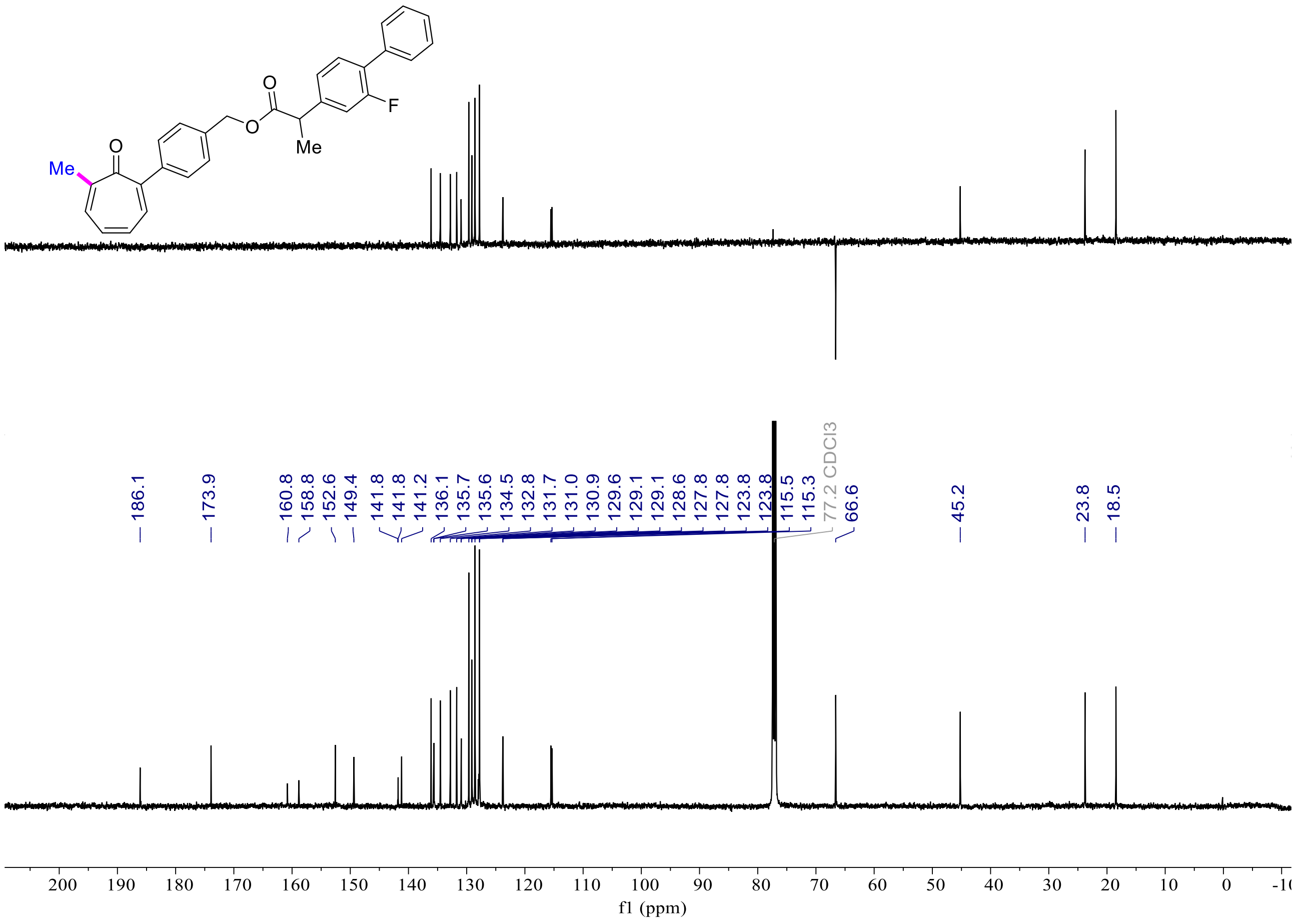

Compound 7v ^{19}F NMR (471 MHz, CDCl$_3$)

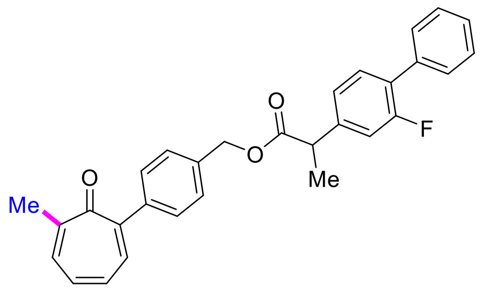

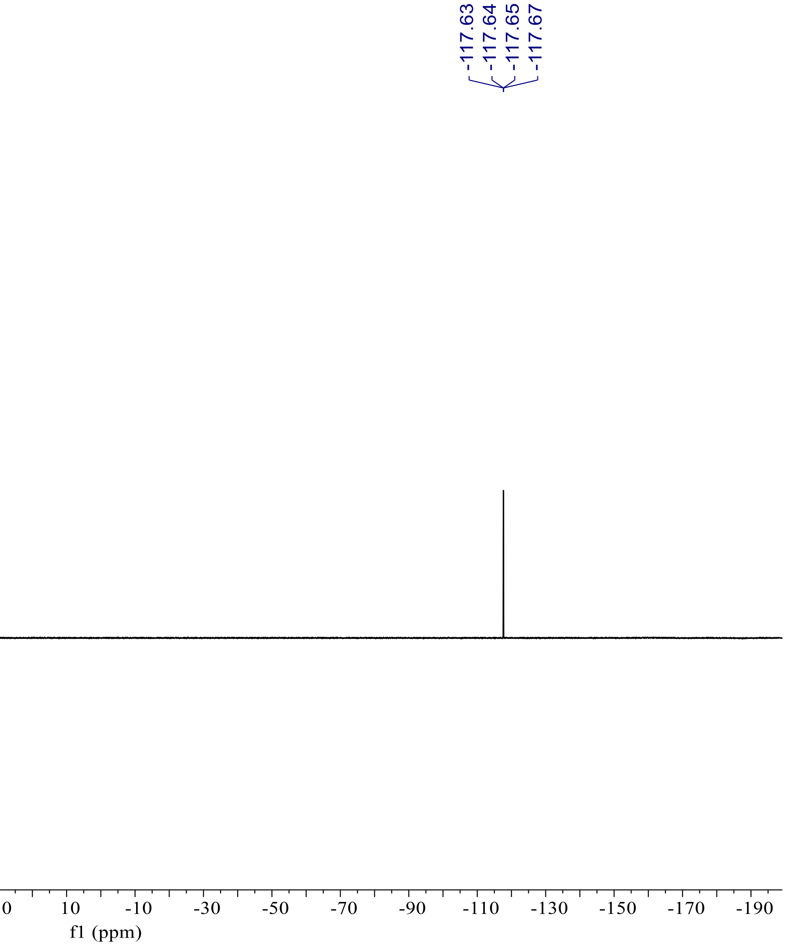

Compound 7v HRMS (ESI-TOF)

**Data Filename**	ESIH202406117.d	**Sample Name**	D4-D4-ZDG3-10C
**Sample ID**		**Position**	P1-B7
**Instrument Name**	Agilent 6520 Q-TOF	**Acq Method**	20160322_MS_ESIH_POS_1min.m
**Acquired Time**	12/25/2024 5:25:35 PM	**DA Method**	ESI-HR-20231114.m
**Comment**	ESIH by fangsu		

User Spectra

Fragmentor Voltage	Collision Energy	Ionization Mode
170	0	ESI

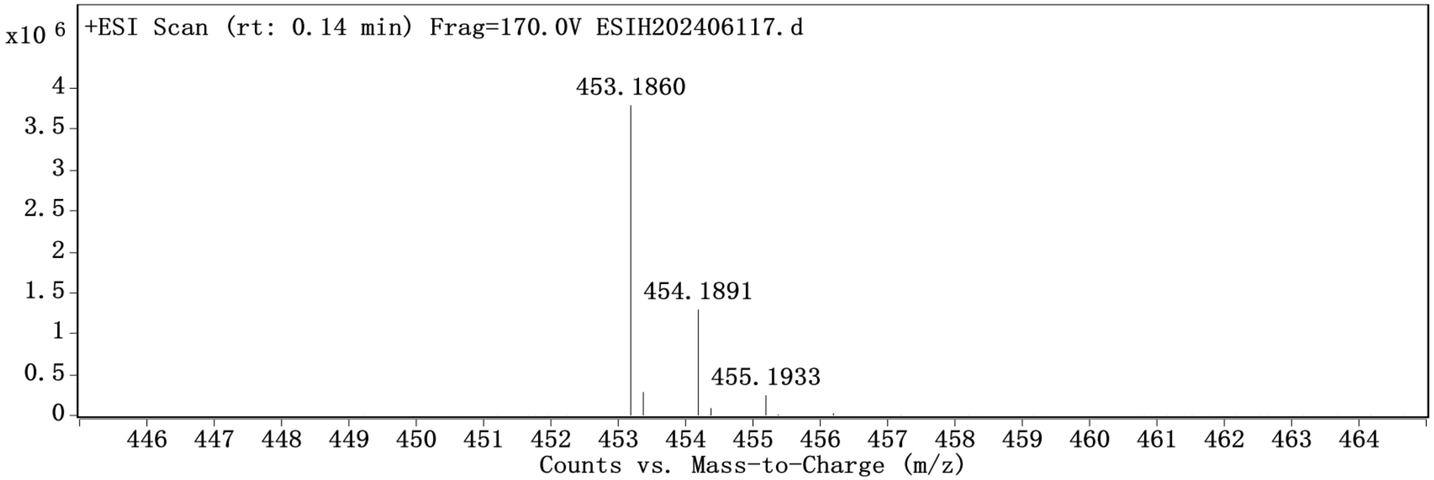

Formula Calculator Results

m/z	Calc m/z	Diff (mDa)	Diff (ppm)	Ion Formula	Ion
453.186	453.186	0.04	0.08	C30 H26 F O3	(M+H)+

--- End Of Report ---

Compound 8b ^{1}H NMR (500 MHz, CD_3OD)

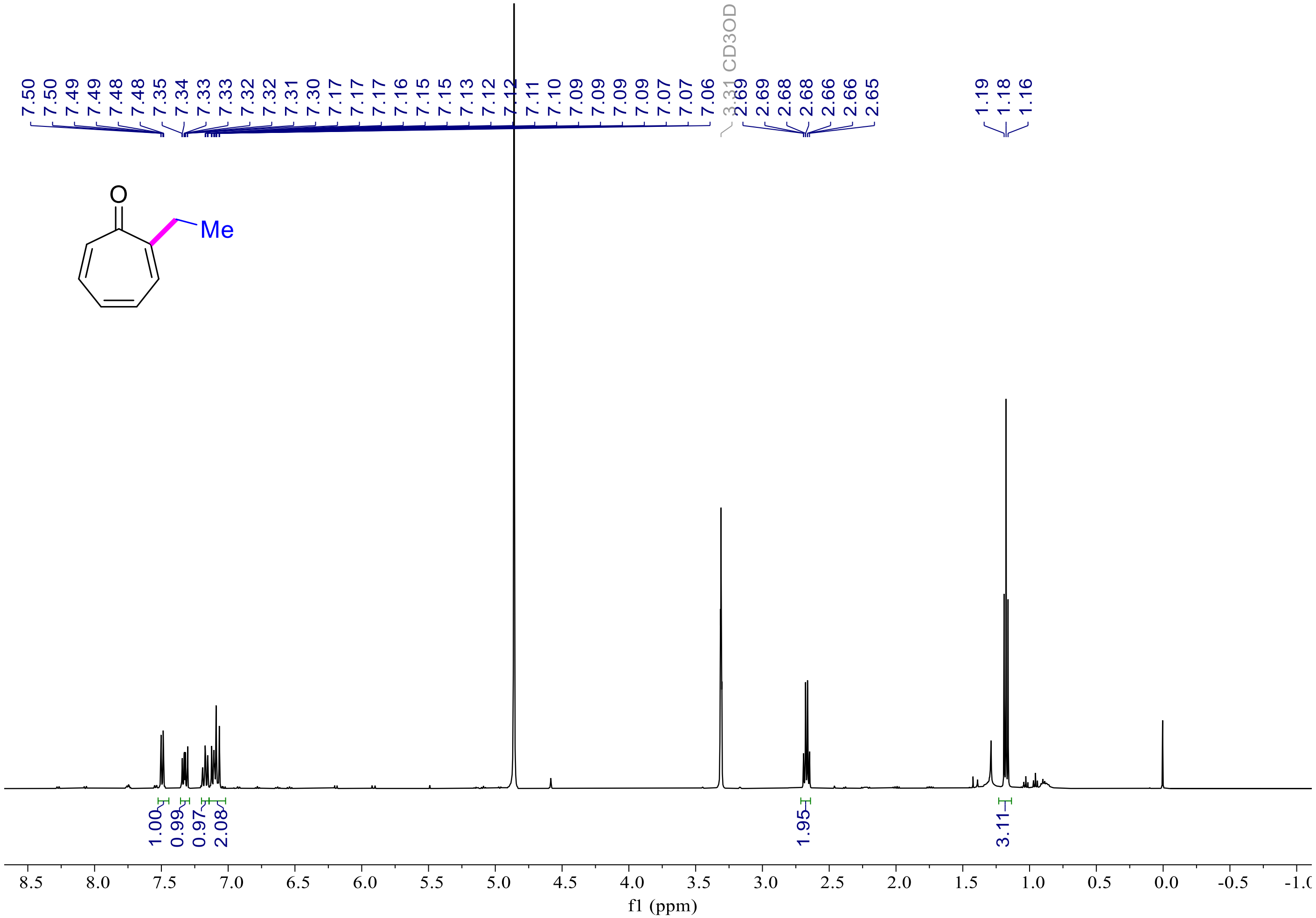

Compound 8b ^{13}C NMR (125 MHz, CD_3OD)

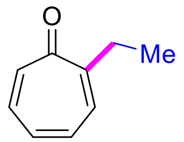

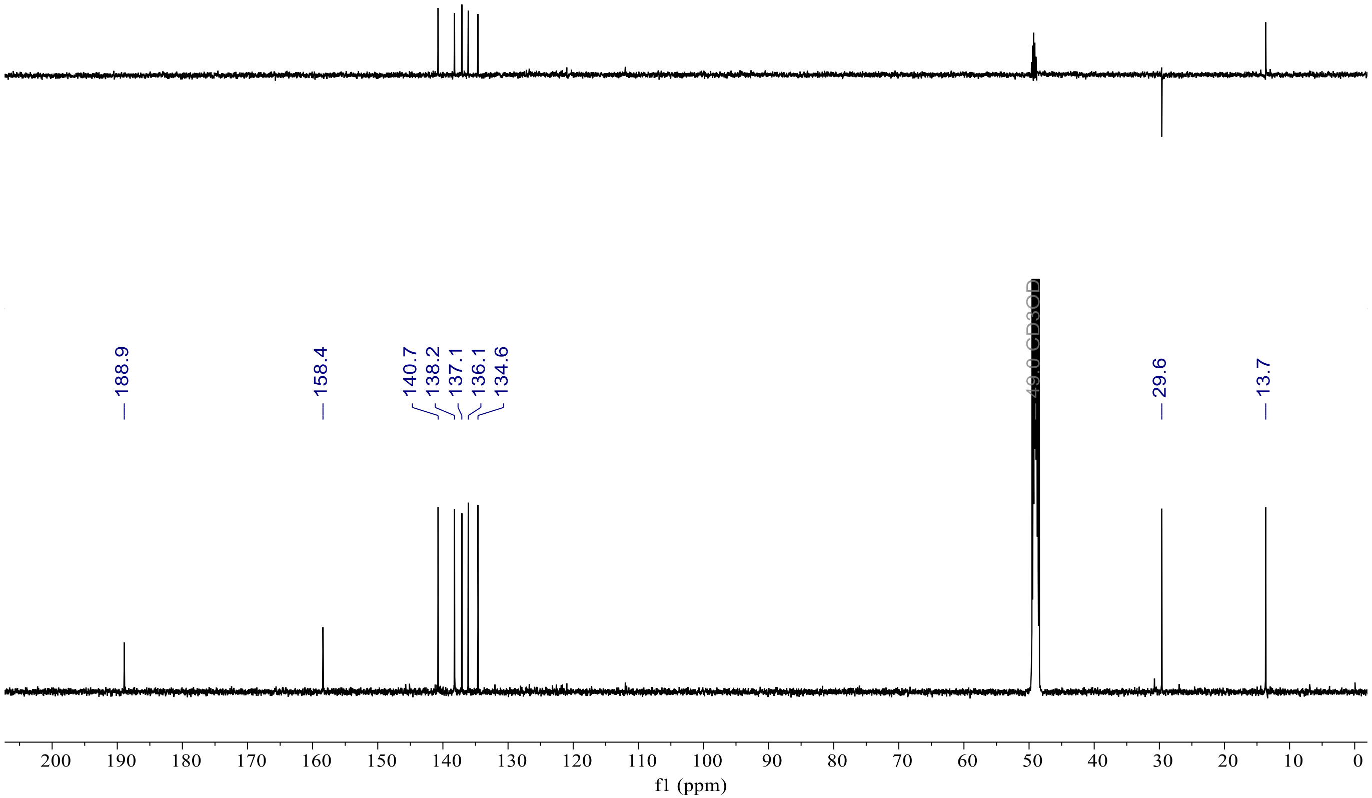

Compound 8b HRMS (ESI-TOF):

**Data Filename**	ESIH202403909.d	**Sample Name**	D4-ZQT11-C
**Sample ID**		**Position**	P1-B1
**Instrument Name**	Agilent 6520 Q-TOF	**Acq Method**	20160322_MS_ESIH_POS_1min.m
**Acquired Time**	7/26/2024 1:57:47 PM	**DA Method**	ESI-HR-20231114.m
**Comment**	ESIH by fangsu		

User Spectra

Fragmentor Voltage	Collision Energy	Ionization Mode
170	0	ESI

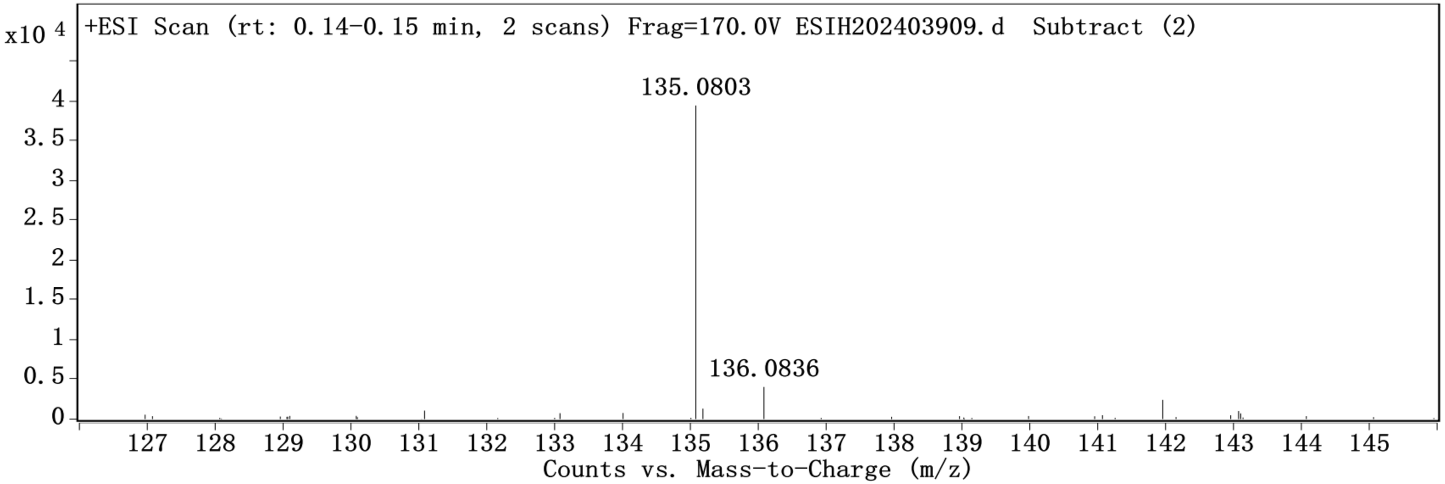

Formula Calculator Results

m/z	Calc m/z	Diff (mDa)	Diff (ppm)	Ion Formula	Ion
135.0803	135.0804	0.16	1.2	C9 H11 O	(M+H)+

--- End Of Report ---

Compound 8c ^{1}H NMR (400 MHz, $CDCl_3$)

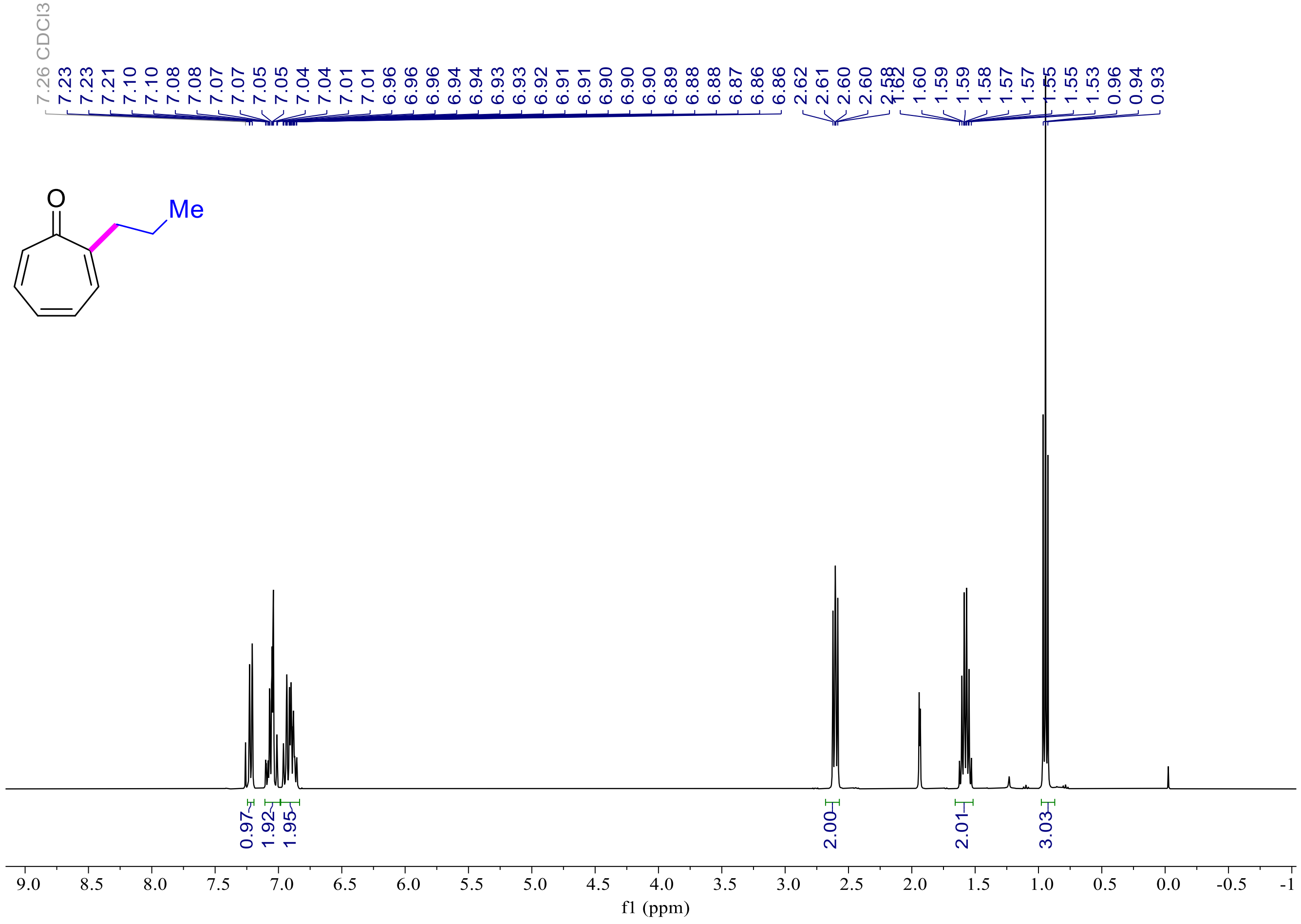

Compound 8c ^{13}C NMR (100 MHz, $CDCl_3$)

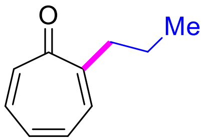

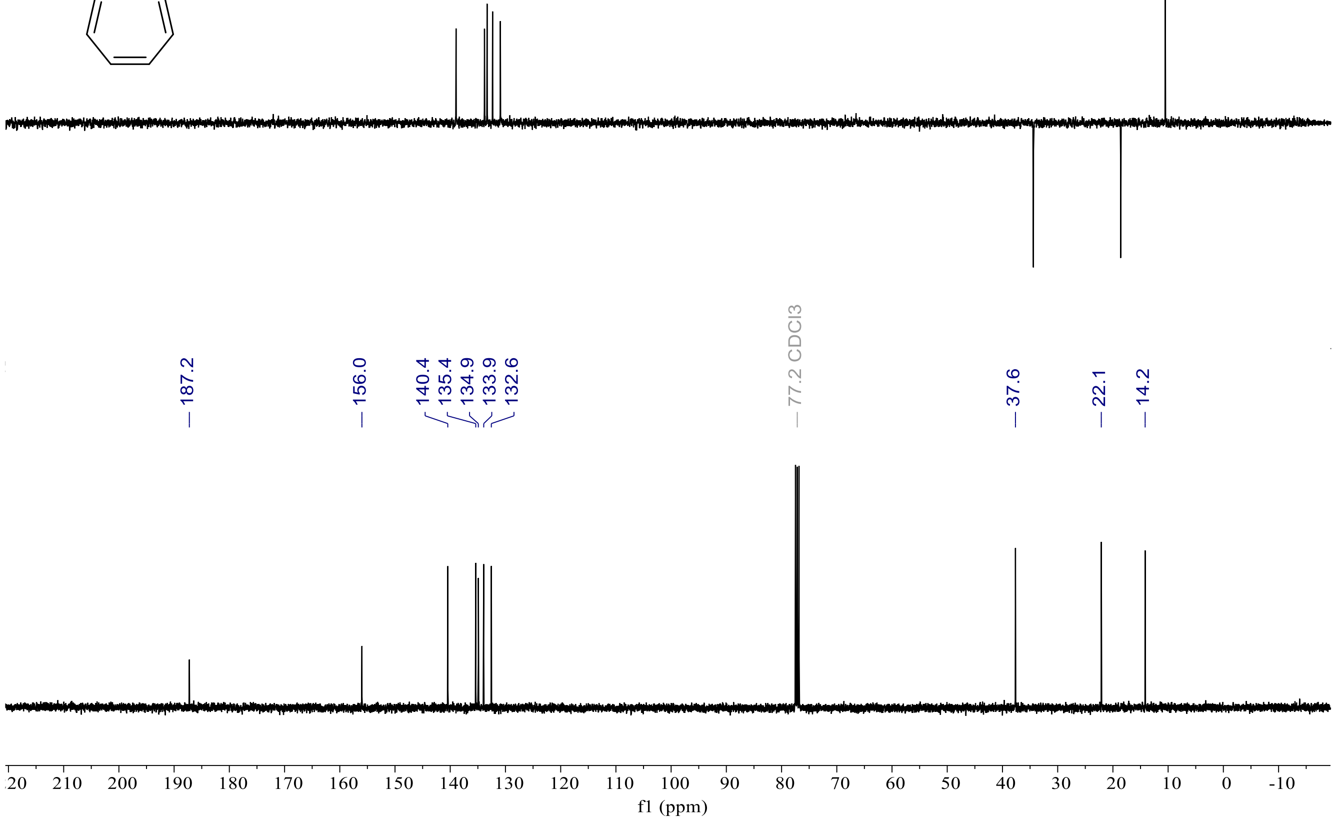

Compound 8c HRMS (ESI-TOF):

**Data Filename**	ESIH202404154.d	**Sample Name**	D4-ZQT18-2
**Sample ID**		**Position**	P1-B1
**Instrument Name**	Agilent 6520 Q-TOF	**Acq Method**	20160322_MS_ESIH_POS_1min.m
**Acquired Time**	8/27/2024 3:40:46 PM	**DA Method**	ESI-HR-20231114.m
**Comment**	ESIH by fangsu		

User Spectra

Fragmentor Voltage	Collision Energy	Ionization Mode
170	0	ESI

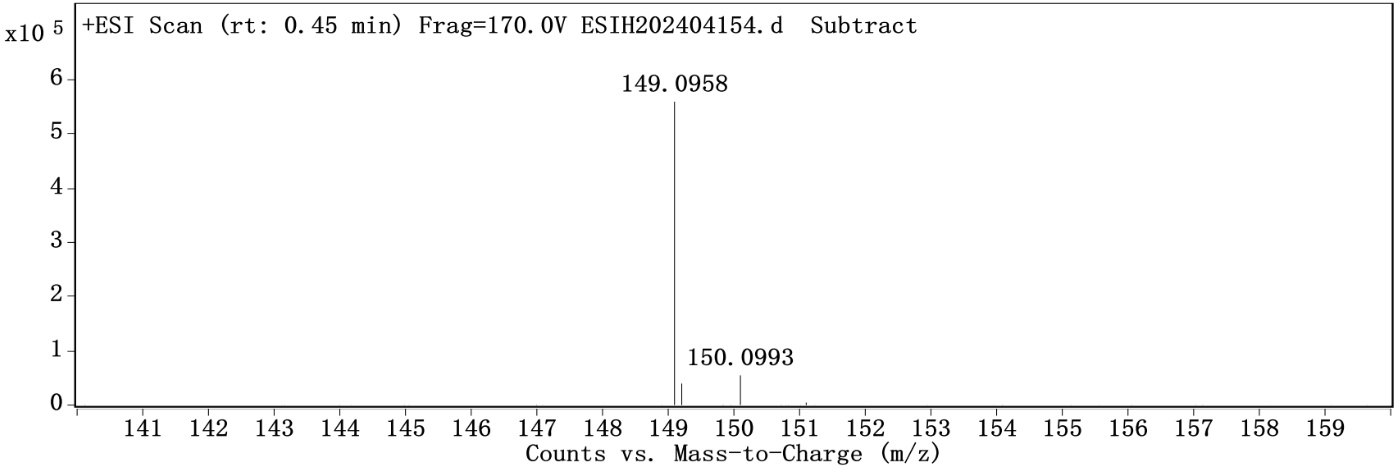

Formula Calculator Results

m/z	Calc m/z	Diff (mDa)	Diff (ppm)	Ion Formula	Ion
149.0958	149.0961	0.34	2.25	C10 H13 O	(M+H)+

--- End Of Report ---

Compound 8d ^{1}H NMR (600 MHz, $CDCl_3$)

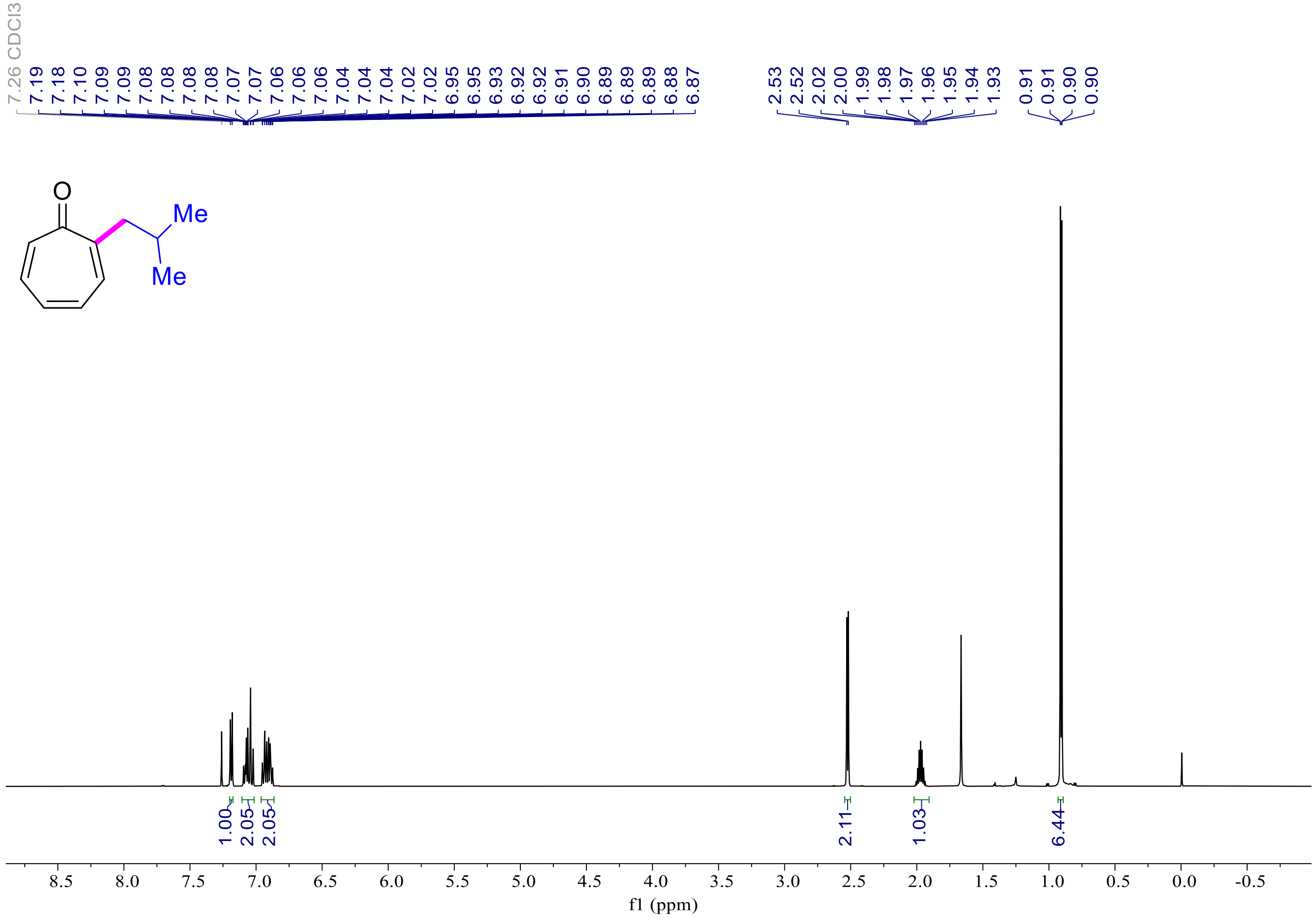

Compound 8d ^{13}C NMR (150 MHz, $CDCl_3$)

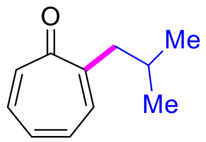

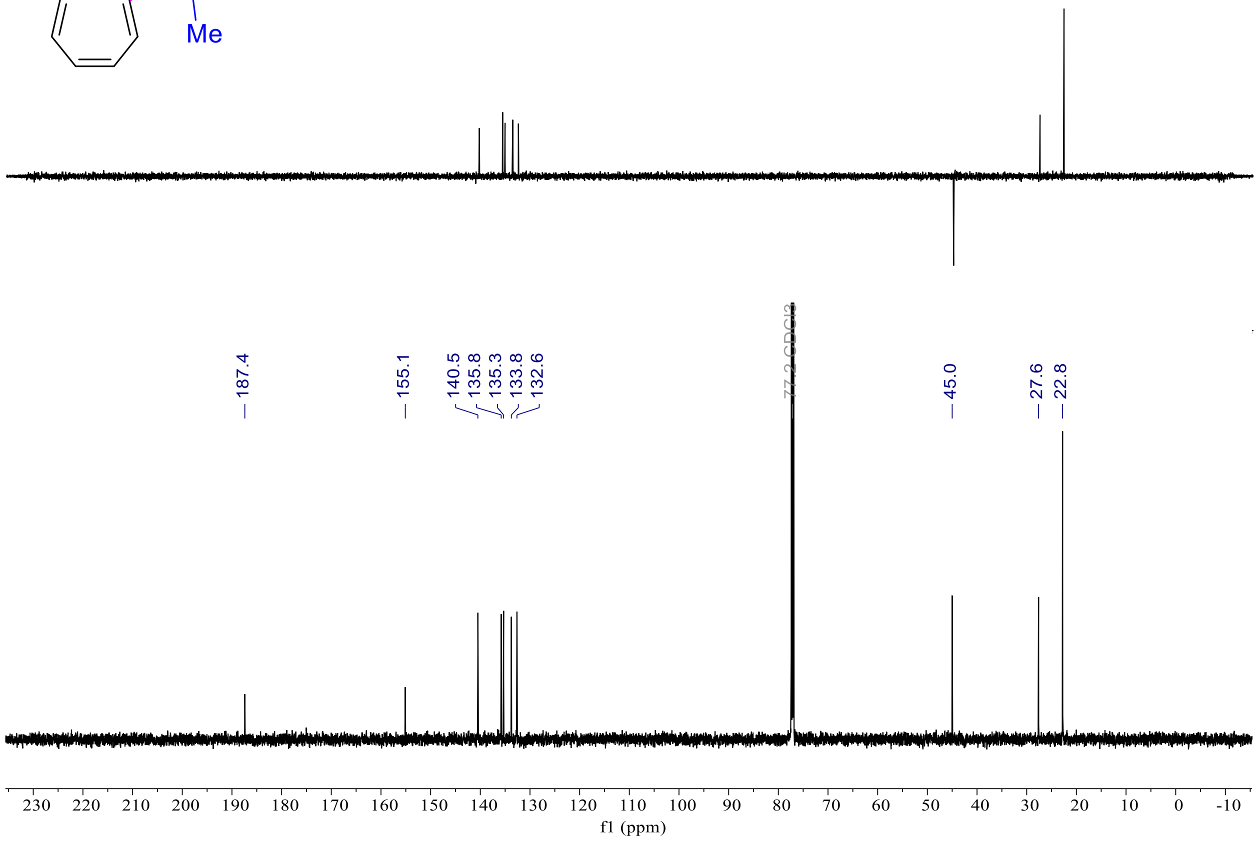

Compound 8d HRMS (ESI-TOF):

**Data Filename**	ESIH202404486.d	**Sample Name**	D4-ZQT18-4
**Sample ID**		**Position**	P1-A1
**Instrument Name**	Agilent 6520 Q-TOF	**Acq Method**	20160322_MS_ESIH_POS_1min.m
**Acquired Time**	9/14/2024 4:12:03 PM	**DA Method**	ESI-HR-20231114.m
**Comment**	ESIH by fangsu		

User Spectra

Fragmentor Voltage	Collision Energy	Ionization Mode
170	0	ESI

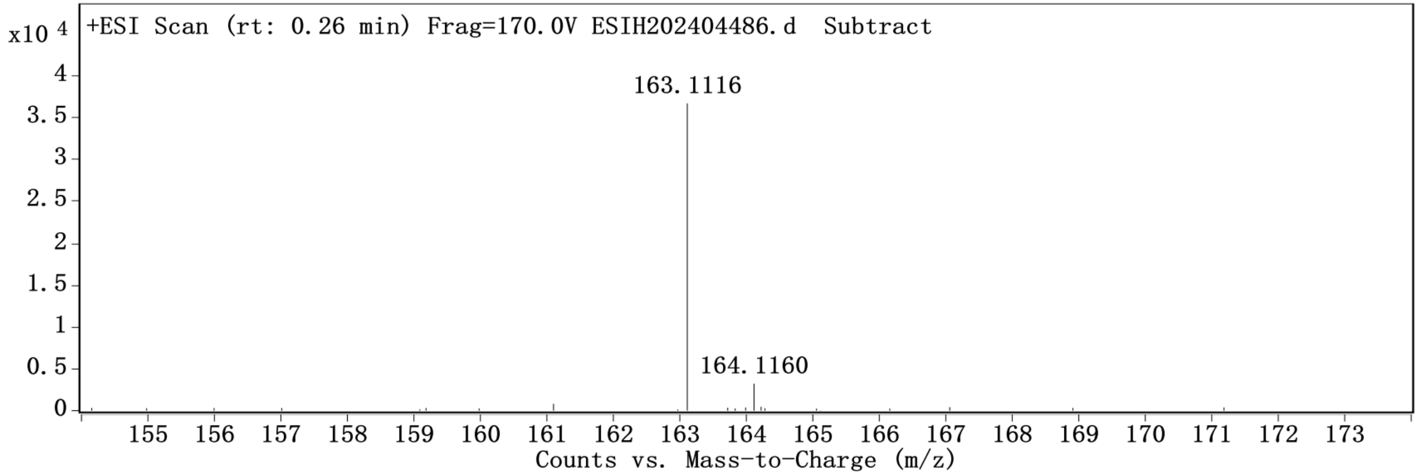

Formula Calculator Results

m/z	Calc m/z	Diff (mDa)	Diff (ppm)	Ion Formula	Ion
163.1116	163.1117	0.13	0.79	C11 H15 O	(M+H)+

--- End Of Report ---

Compound 8e ¹H NMR (600 MHz, $CDCl_3$)

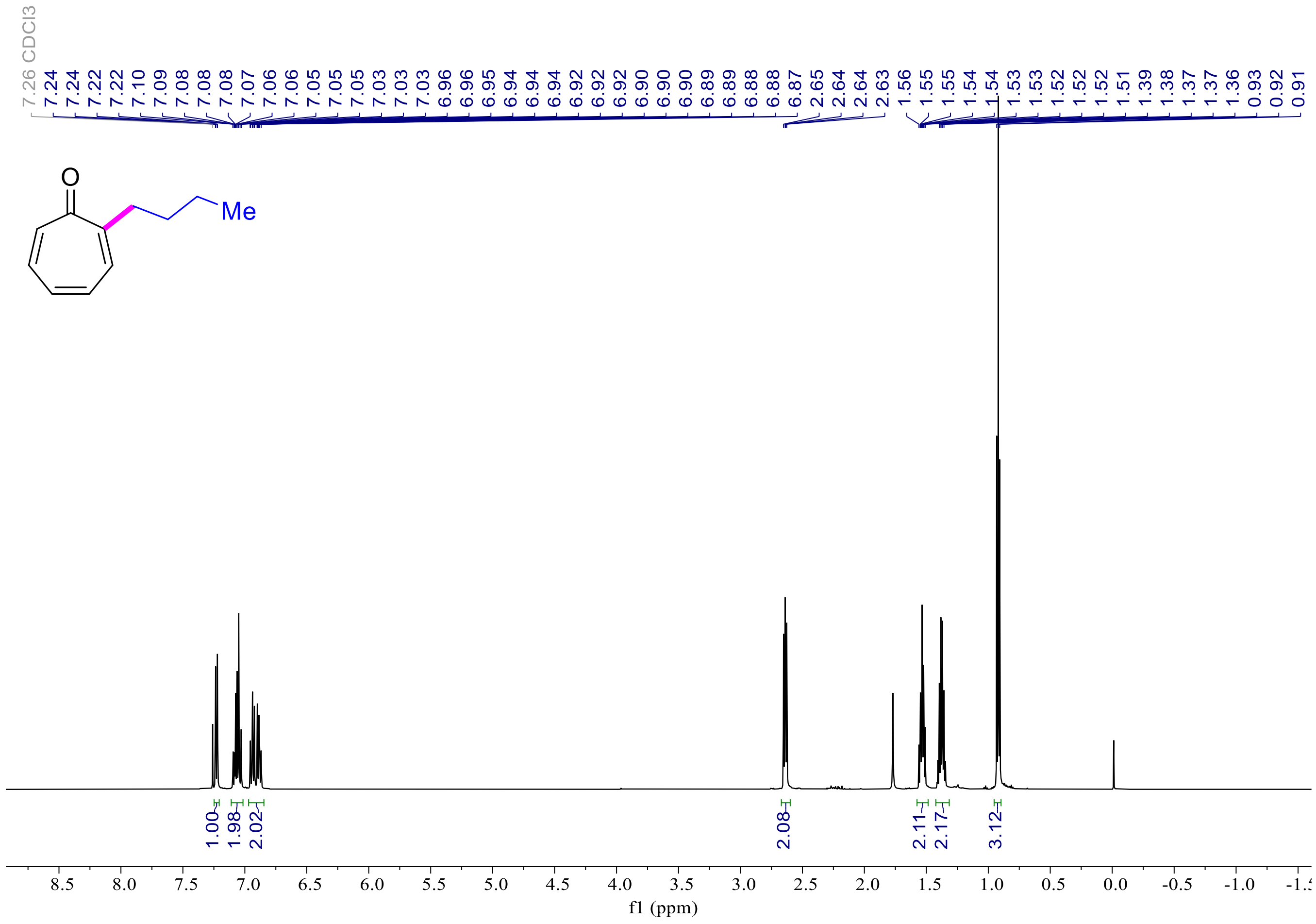

Compound 8e ^{13}C NMR (150 MHz, $CDCl_3$)

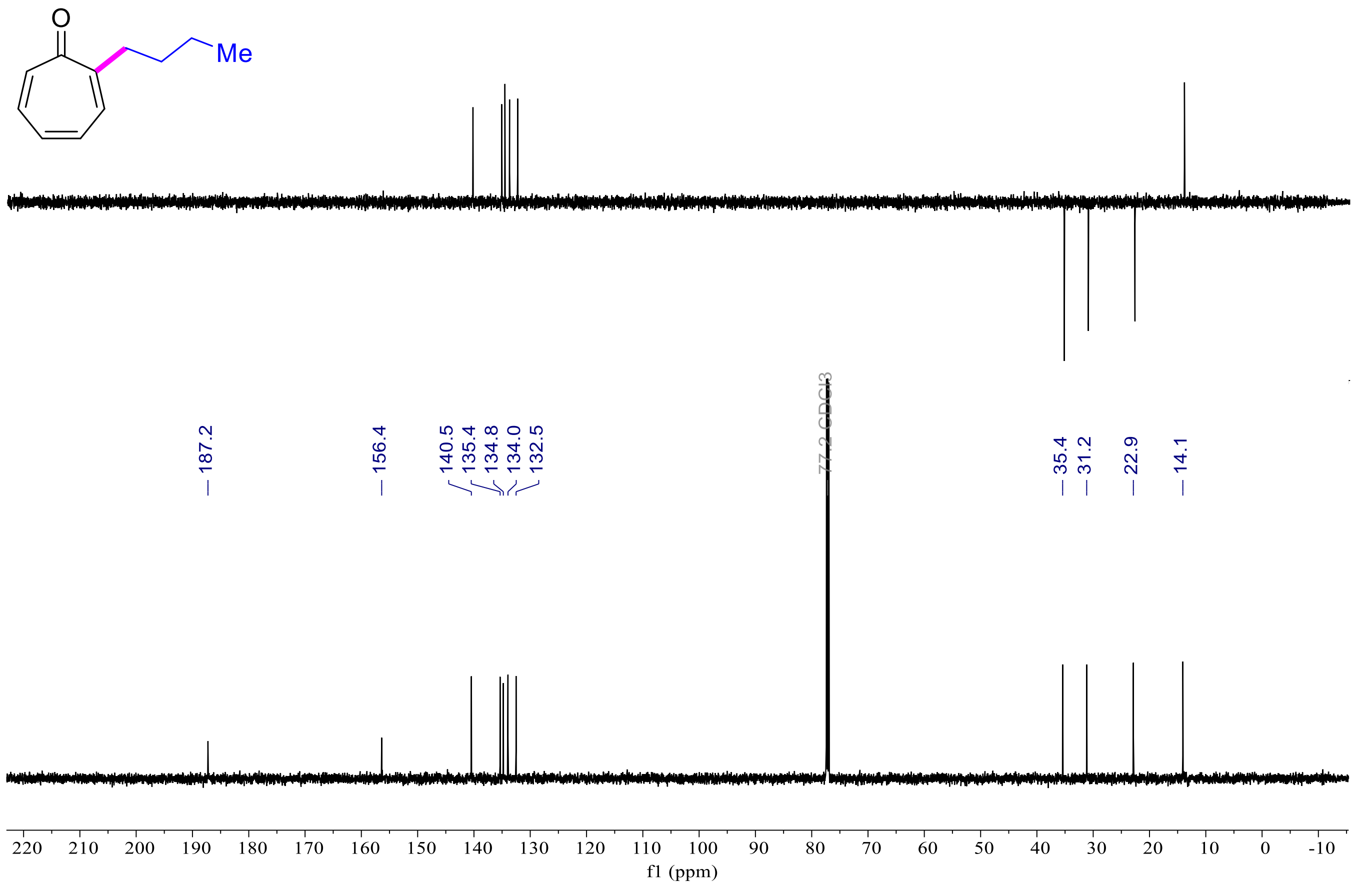

Compound 8e HRMS (ESI-TOF):

**Data Filename**	ESIH202404772.d	**Sample Name**	D4-ZQT18-45B
**Sample ID**		**Position**	P1-C3
**Instrument Name**	Agilent 6520 Q-TOF	**Acq Method**	20160322_MS_ESIH_POS_1min.m
**Acquired Time**	9/27/2024 4:34:51 PM	**DA Method**	ESI-HR-20231114.m
**Comment**	ESIH by fangsu		

User Spectra

Fragmentor Voltage	Collision Energy	Ionization Mode
170	0	ESI

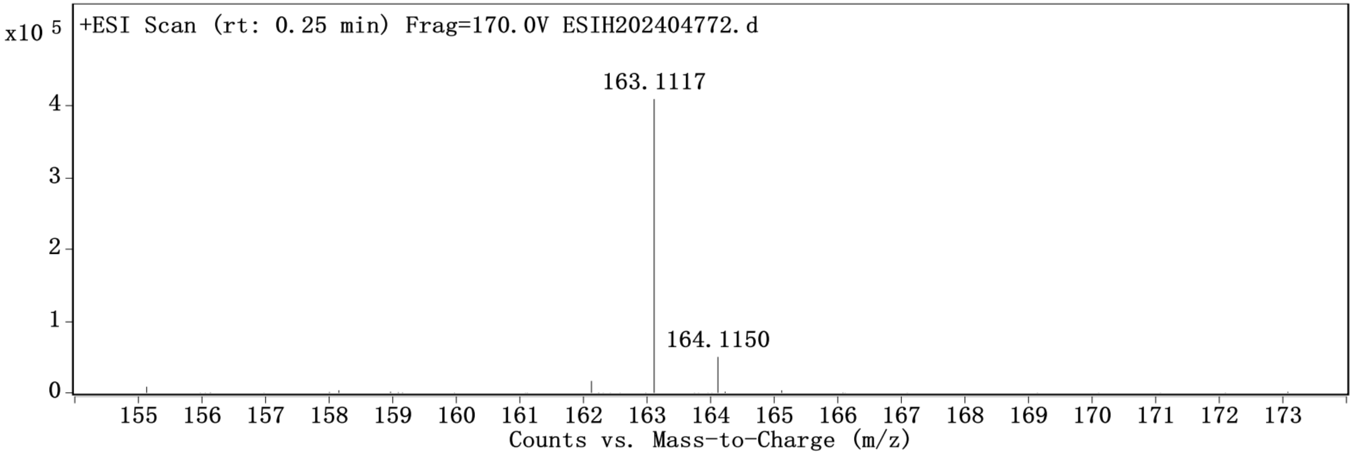

Formula Calculator Results

m/z	Calc m/z	Diff (mDa)	Diff (ppm)	Ion Formula	Ion
163.1117	163.1117	0.04	0.26	C11 H15 O	(M+H)+

--- End Of Report ---

Compound 8f ^{1}H NMR (400 MHz, $CDCl_3$)

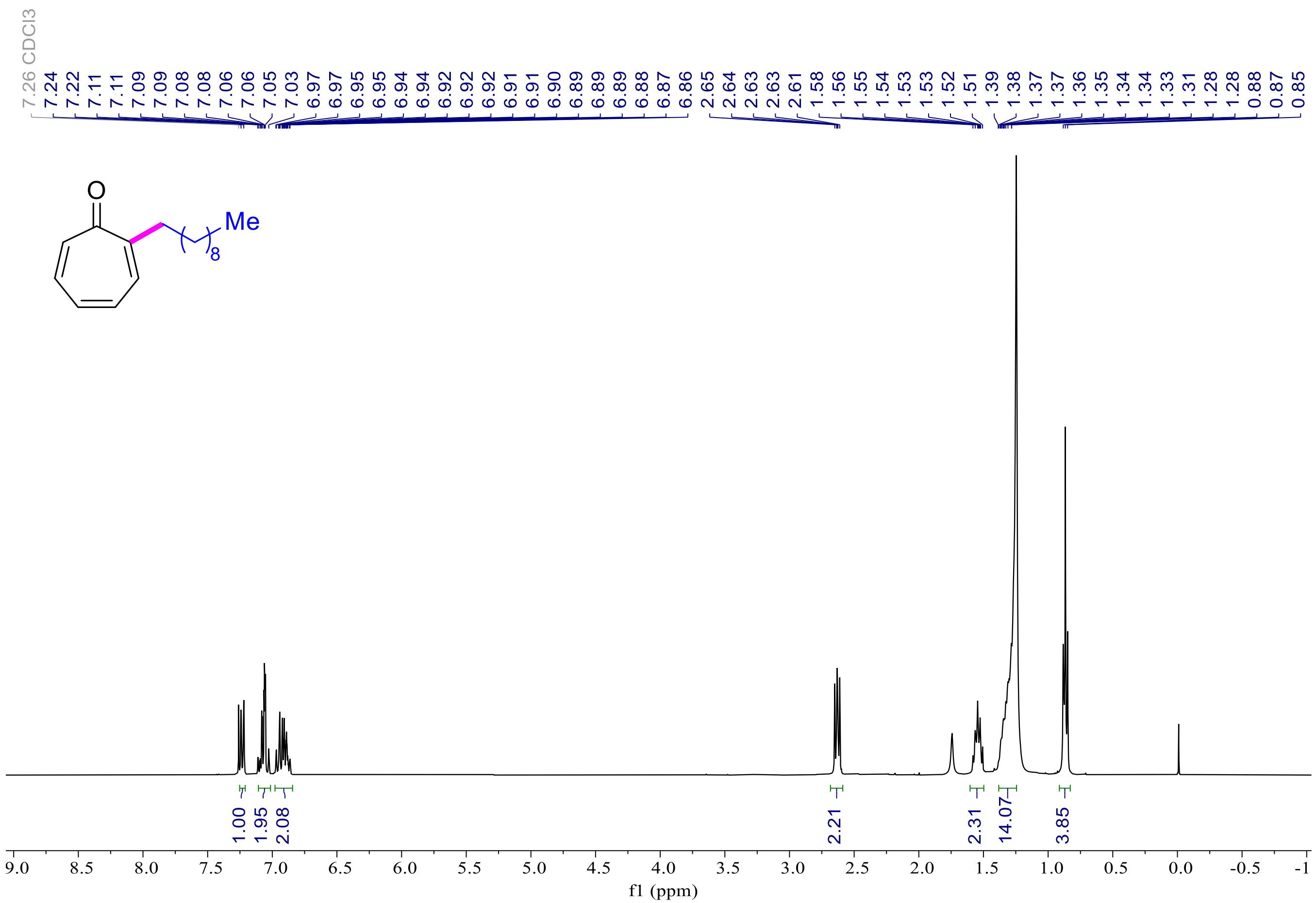

Compound 8f ^{13}C NMR (100 MHz, $CDCl_3$)

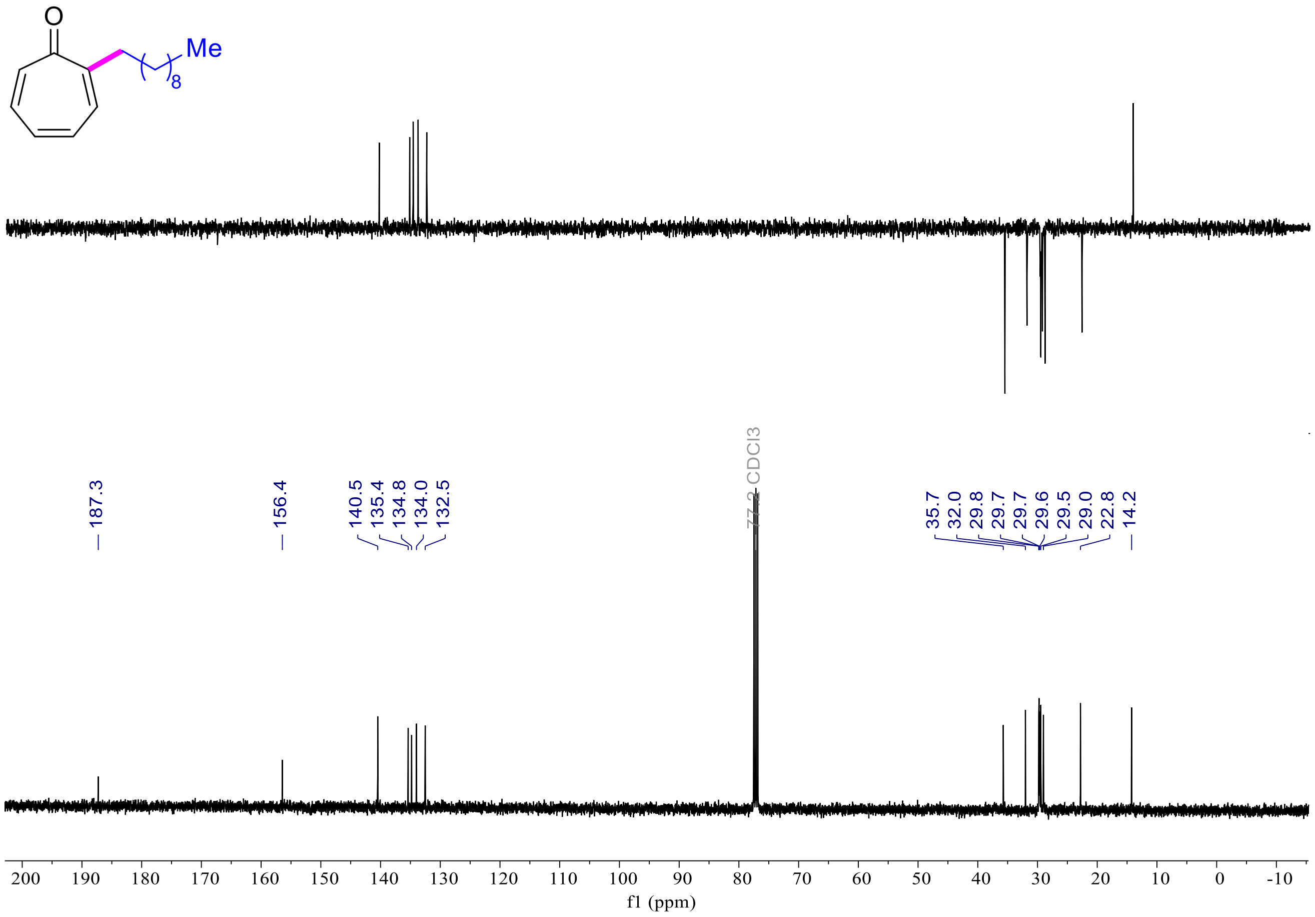

Compound 8f HRMS (ESI-TOF):

**Data Filename**	ESIH202404773.d	**Sample Name**	D4-ZQT18-43
**Sample ID**		**Position**	P1-D1
**Instrument Name**	Agilent 6520 Q-TOF	**Acq Method**	20160322_MS_ESIH_POS_1min.m
**Acquired Time**	9/27/2024 4:47:09 PM	**DA Method**	ESI-HR-20231114.m
**Comment**	ESIH by fangsu		

User Spectra

Fragmentor Voltage	Collision Energy	Ionization Mode
170	0	ESI

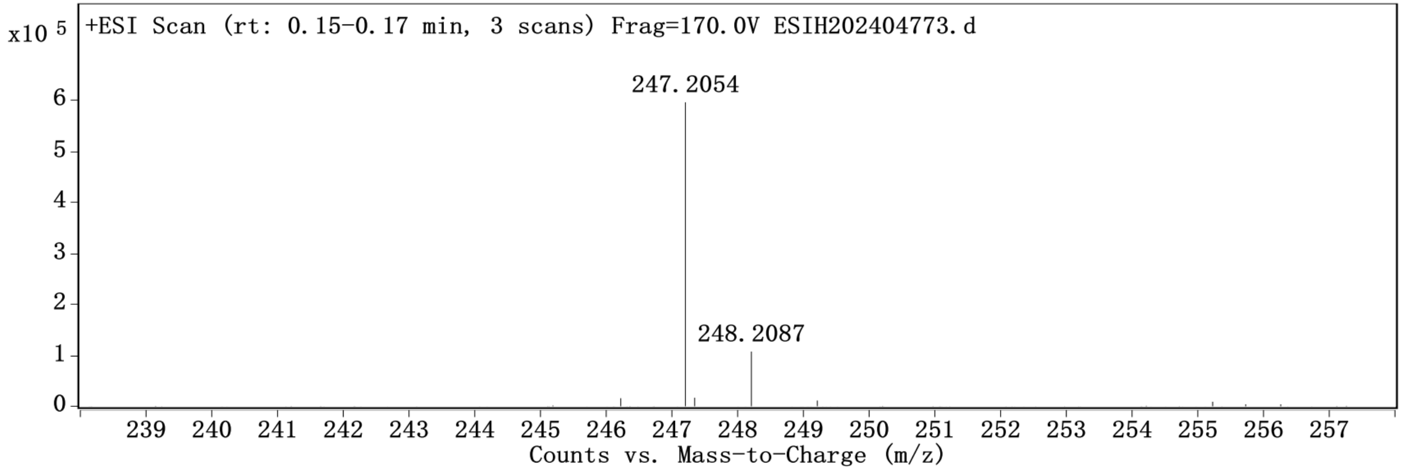

Formula Calculator Results

m/z	Calc m/z	Diff (mDa)	Diff (ppm)	Ion Formula	Ion
247.2054	247.2056	0.26	1.04	C17 H27 O	(M+H)+

--- End Of Report ---

Compound 8g ^{1}H NMR (600 MHz, CDCl$_3$)

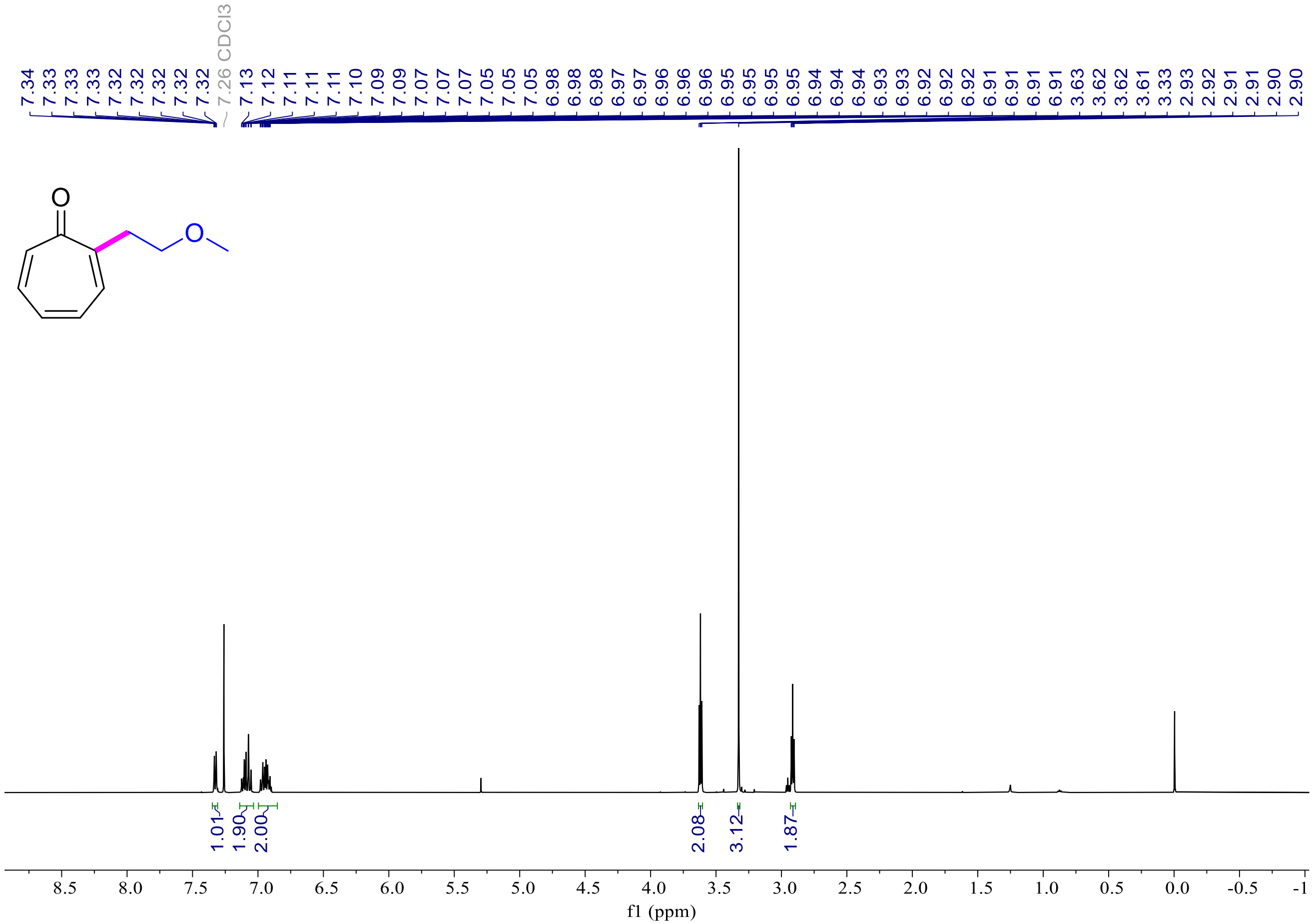

Compound 8g ^{13}C NMR (150 MHz, $CDCl_3$)

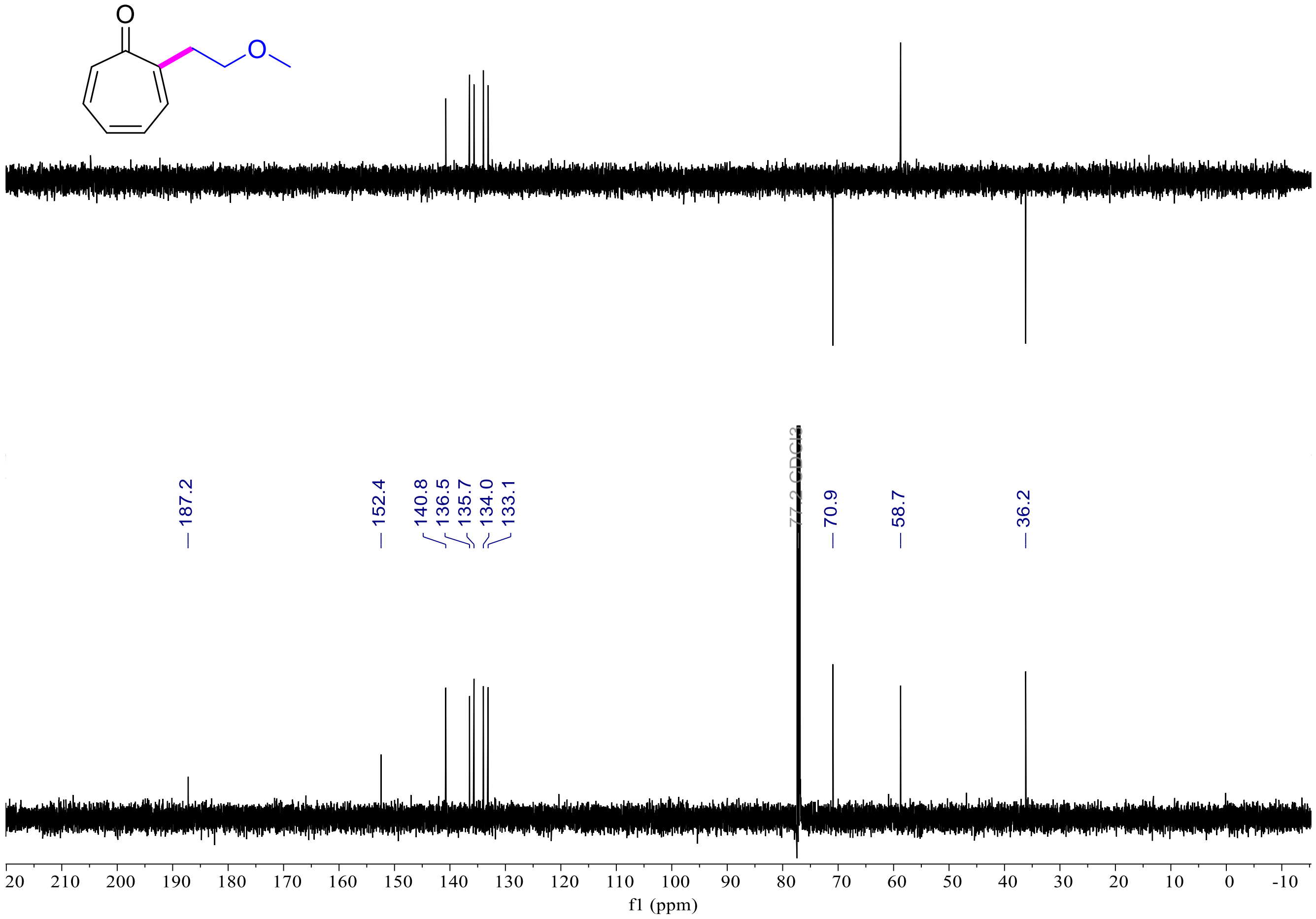

Compound 8g HRMS (ESI-TOF):

**Data Filename**	ESIH202404197.d	**Sample Name**	D4-ZQT18-5A
**Sample ID**		**Position**	P1-B1
**Instrument Name**	Agilent 6520 Q-TOF	**Acq Method**	20160322_MS_ESIH_POS_1min.m
**Acquired Time**	8/29/2024 4:12:35 PM	**DA Method**	ESI-HR-20231114.m
**Comment**	ESIH by fangsu		

User Spectra

**Fragmentor Voltage**	**Collision Energy**	**Ionization Mode**
170	0	ESI

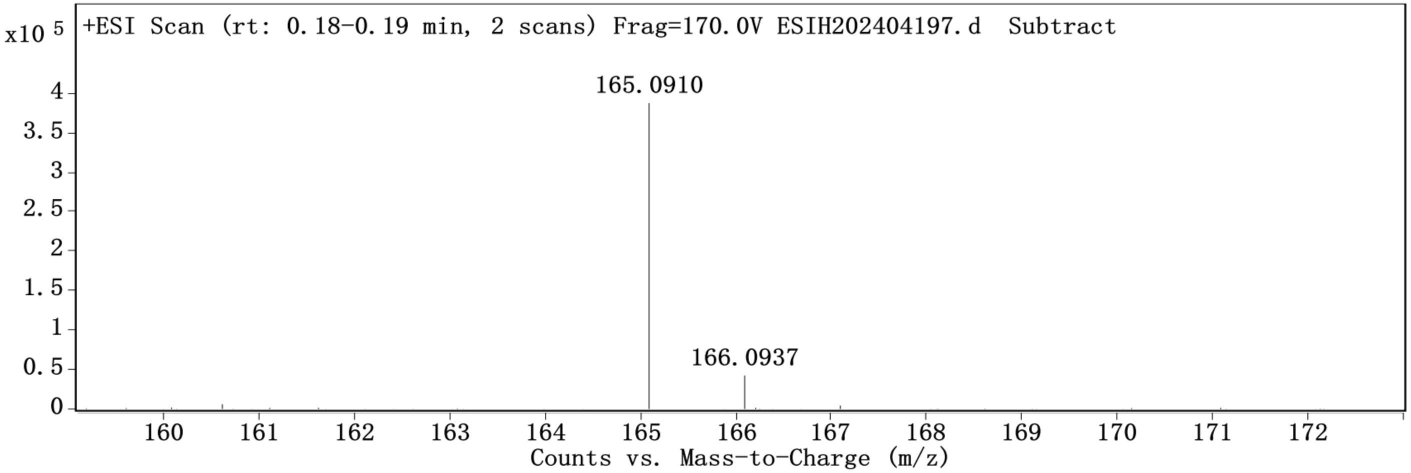

Formula Calculator Results

m/z	Calc m/z	Diff (mDa)	Diff (ppm)	Ion Formula	Ion
165.091	165.091	-0.02	-0.13	C10 H13 O2	(M+H)+

--- End Of Report ---

Compound 8h ^{1}H NMR (600 MHz, $CDCl_3$)

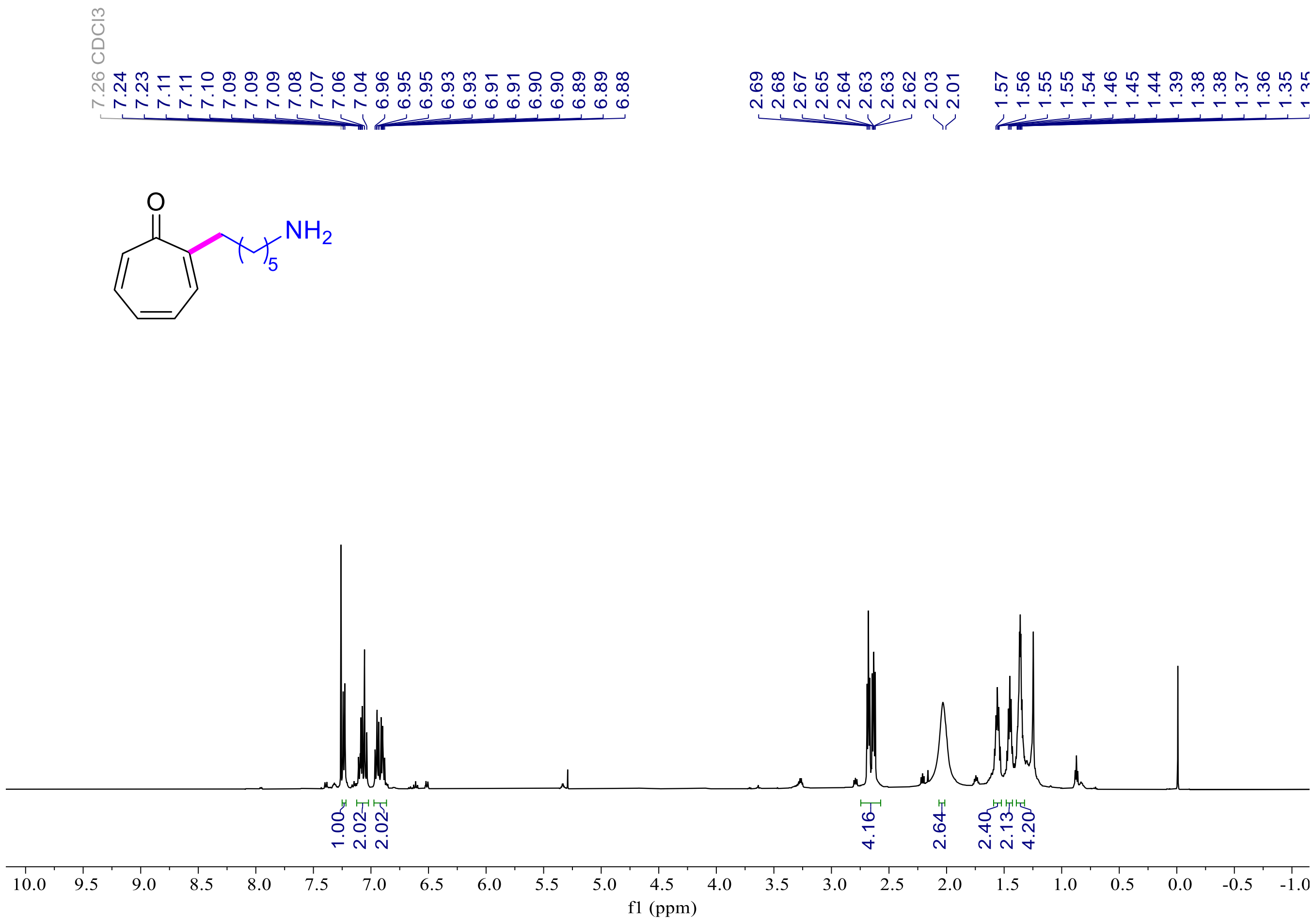

Compound 8h ^{13}C NMR (150 MHz, $CDCl_3$)

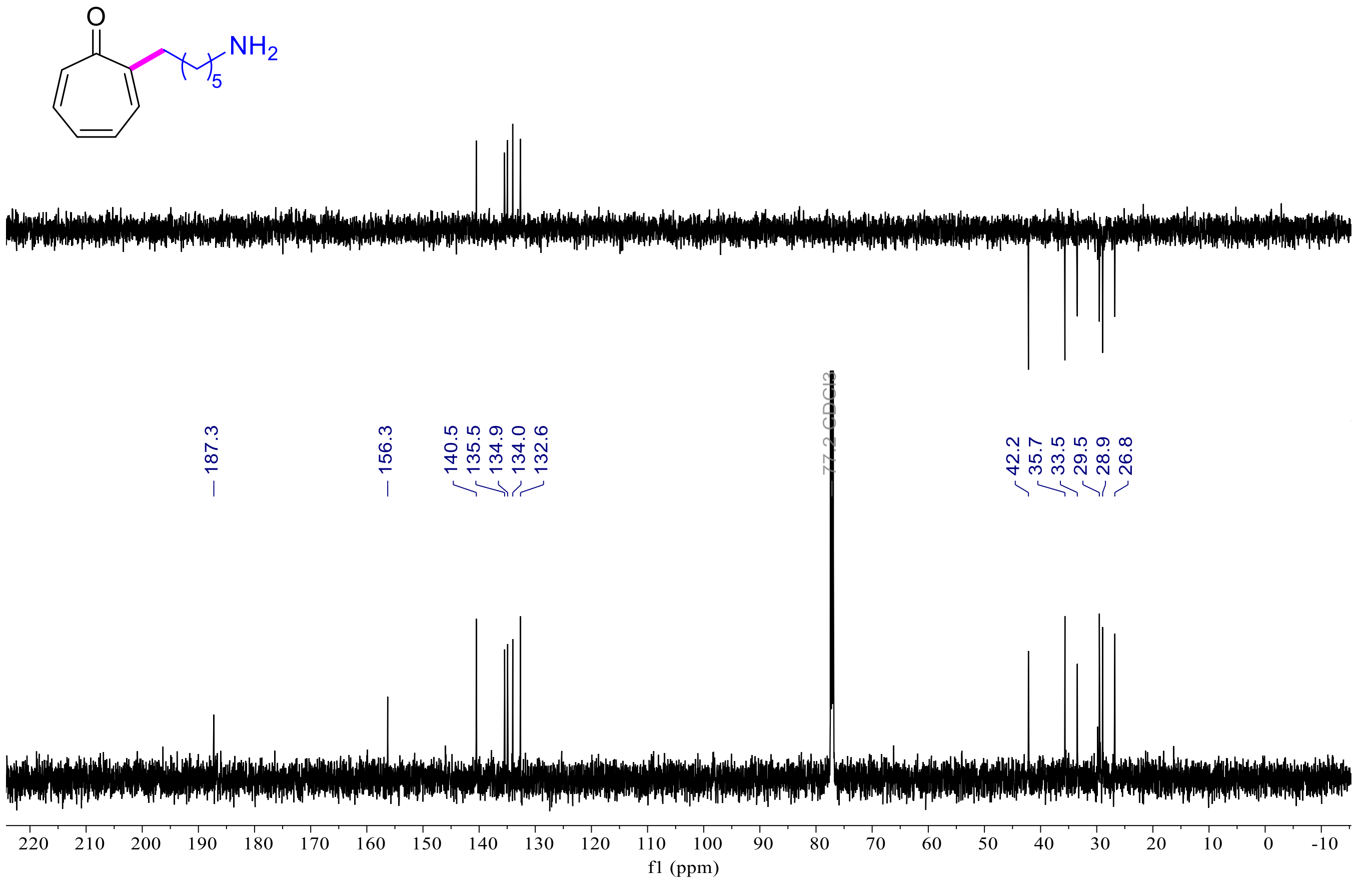

Compound 8h HRMS (ESI-TOF):

**Data Filename**	ESIH202404984.d	**Sample Name**	D4-ZQT18-56C
**Sample ID**		**Position**	P1-B5
**Instrument Name**	Agilent 6520 Q-TOF	**Acq Method**	20160322_MS_ESIH_POS_1min.m
**Acquired Time**	10/22/2024 3:52:15 PM	**DA Method**	ESI-HR-20231114.m
**Comment**	ESIH by fangsu		

User Spectra

Fragmentor Voltage	Collision Energy	Ionization Mode
170	0	ESI

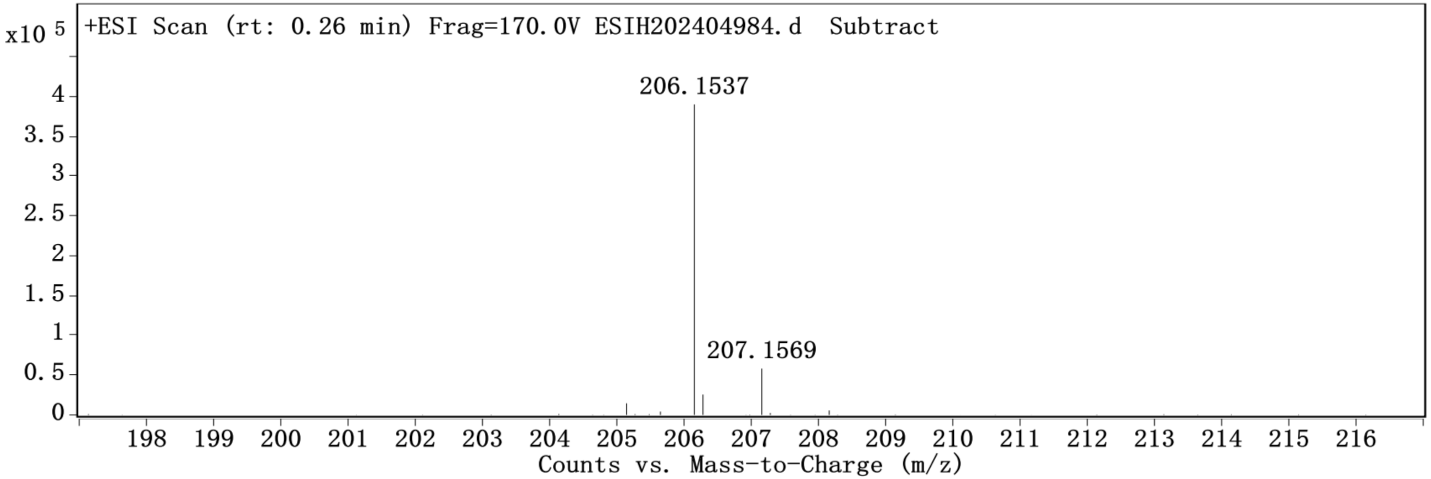

Formula Calculator Results

m/z	Calc m/z	Diff (mDa)	Diff (ppm)	Ion Formula	Ion
206.1537	206.1539	0.26	1.27	C13 H20 N O	(M+H)+

--- End Of Report ---

Compound 8i ^{1}H NMR (400 MHz, $CDCl_3$)

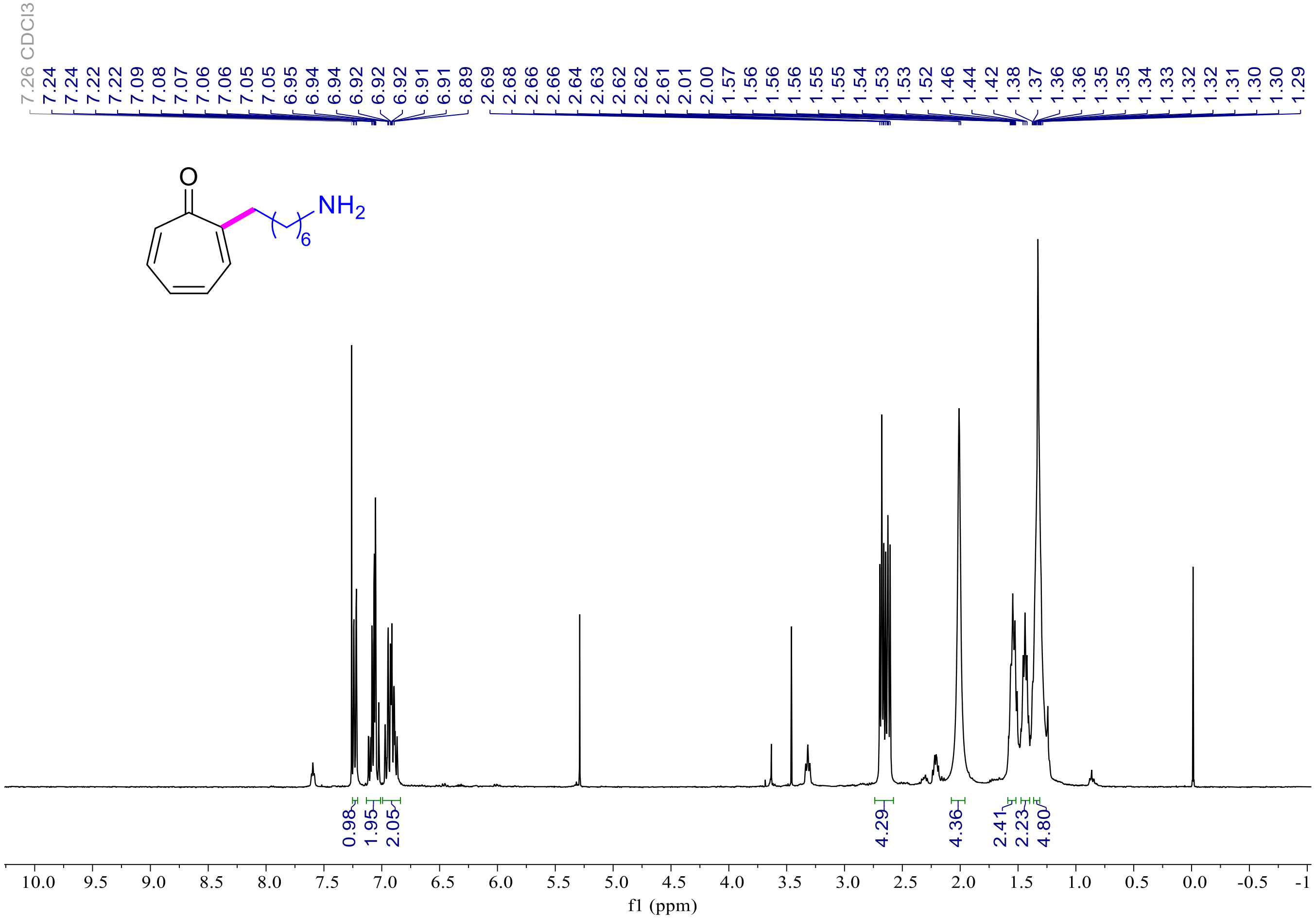

Compound 8i ^{13}C NMR (100 MHz, $CDCl_3$)

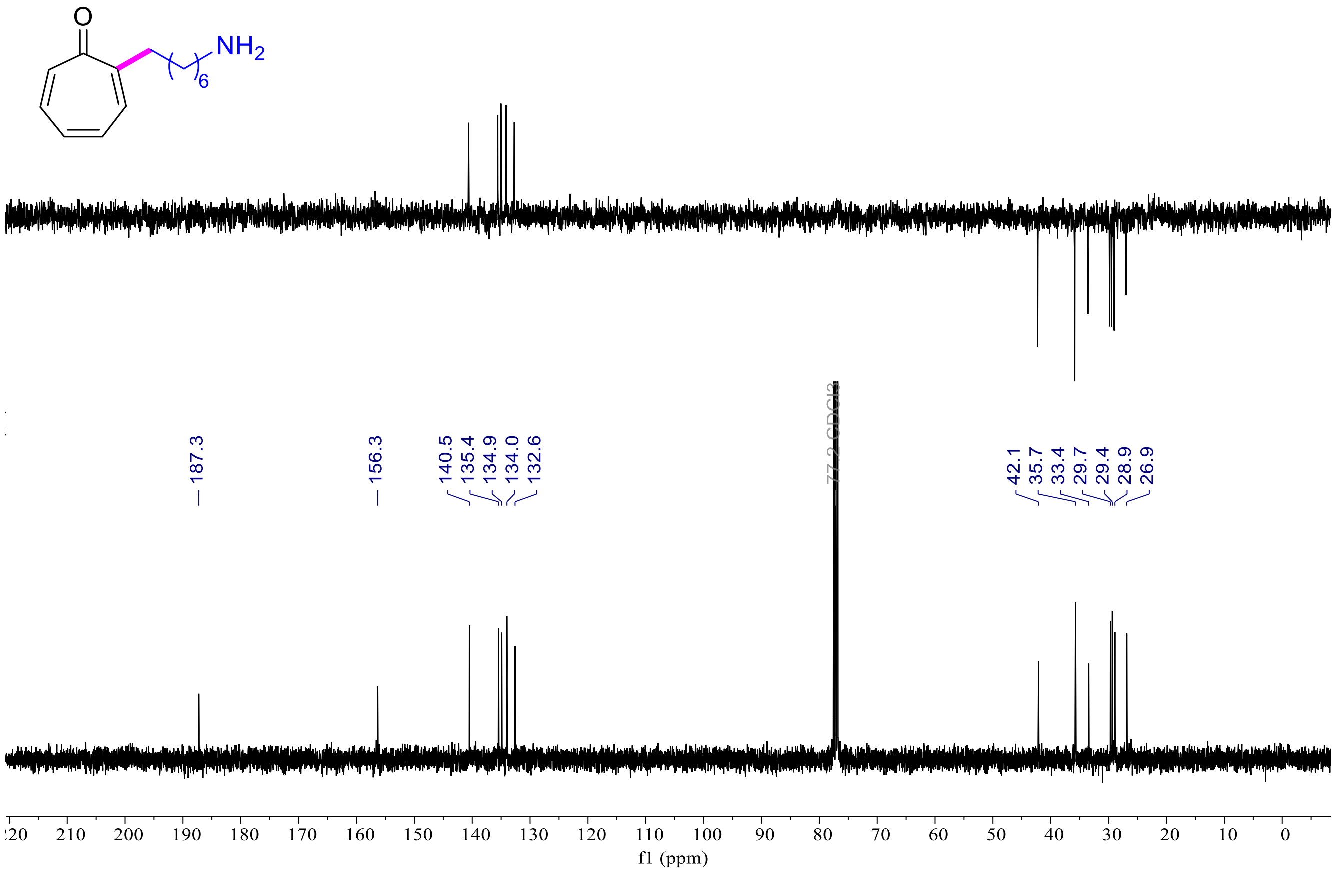

Compound 8i HRMS (ESI-TOF):

**Data Filename**	ESIH202404943.d	**Sample Name**	D4-ZQT18-51D
**Sample ID**		**Position**	P1-D4
**Instrument Name**	Agilent 6520 Q-TOF	**Acq Method**	20160322_MS_ESIH_POS_1min.m
**Acquired Time**	10/18/2024 2:51:27 PM	**DA Method**	ESI-HR-20231114.m
**Comment**	ESIH by fangsu		

User Spectra

Fragmentor Voltage	Collision Energy	Ionization Mode
170	0	ESI

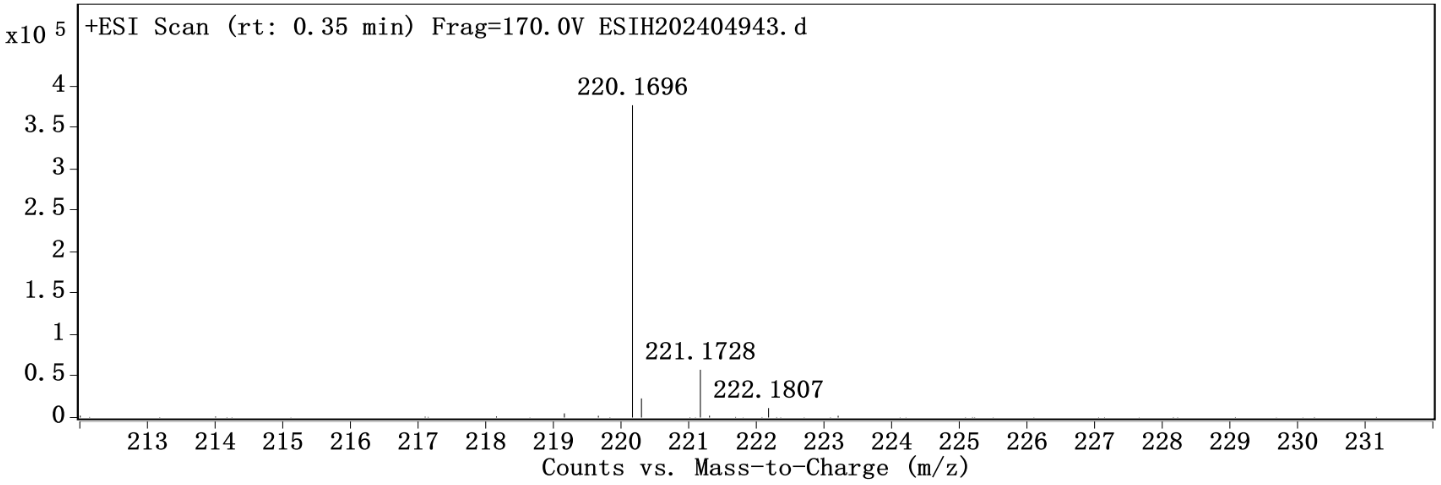

Formula Calculator Results

m/z	Calc m/z	Diff (mDa)	Diff (ppm)	Ion Formula	Ion
220.1696	220.1696	0	0	C14 H22 N O	(M+H)+

--- End Of Report ---

Compound 8j ^{1}H NMR (600 MHz, $CDCl_3$)

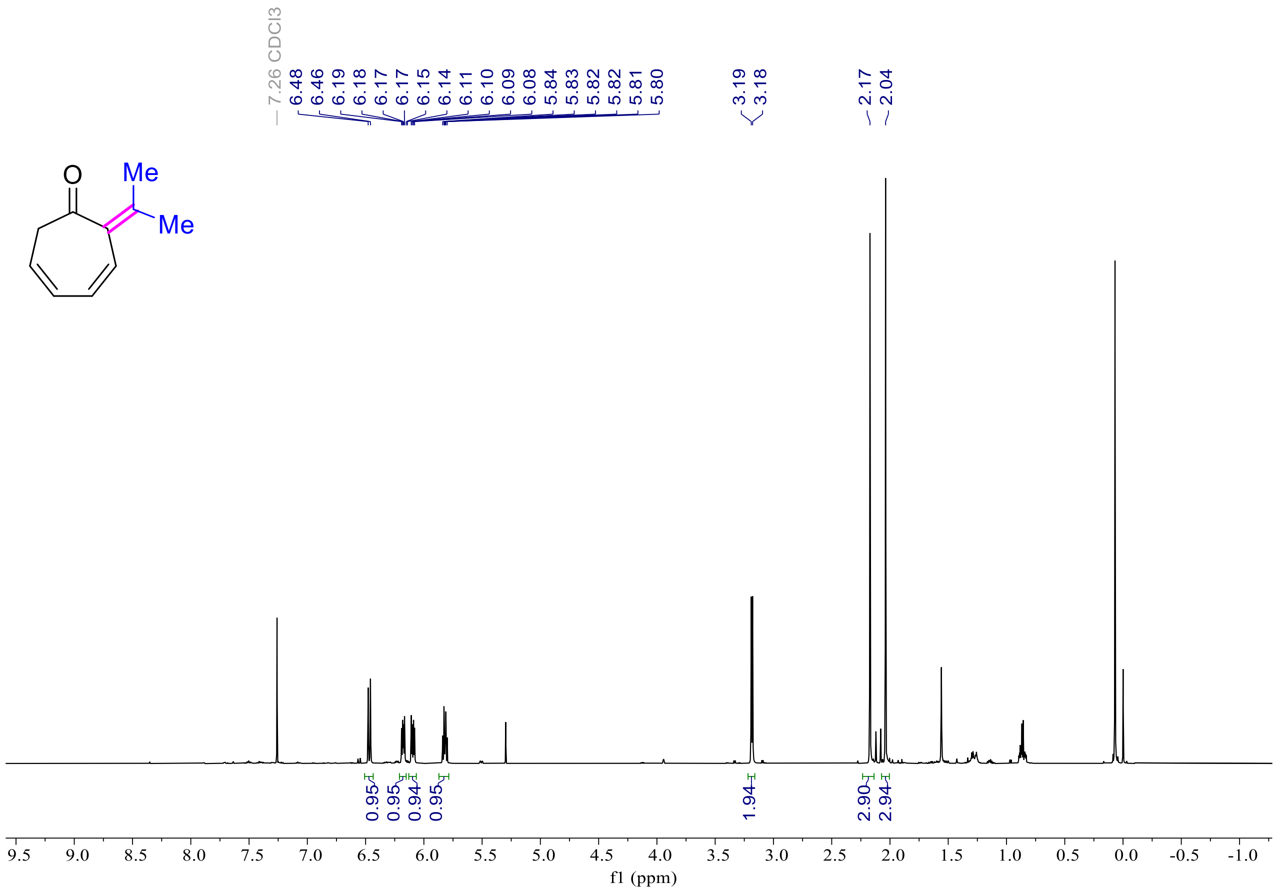

Compound 8j ^{13}C NMR (150 MHz, $CDCl_3$)

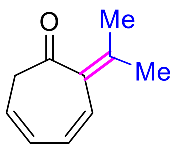

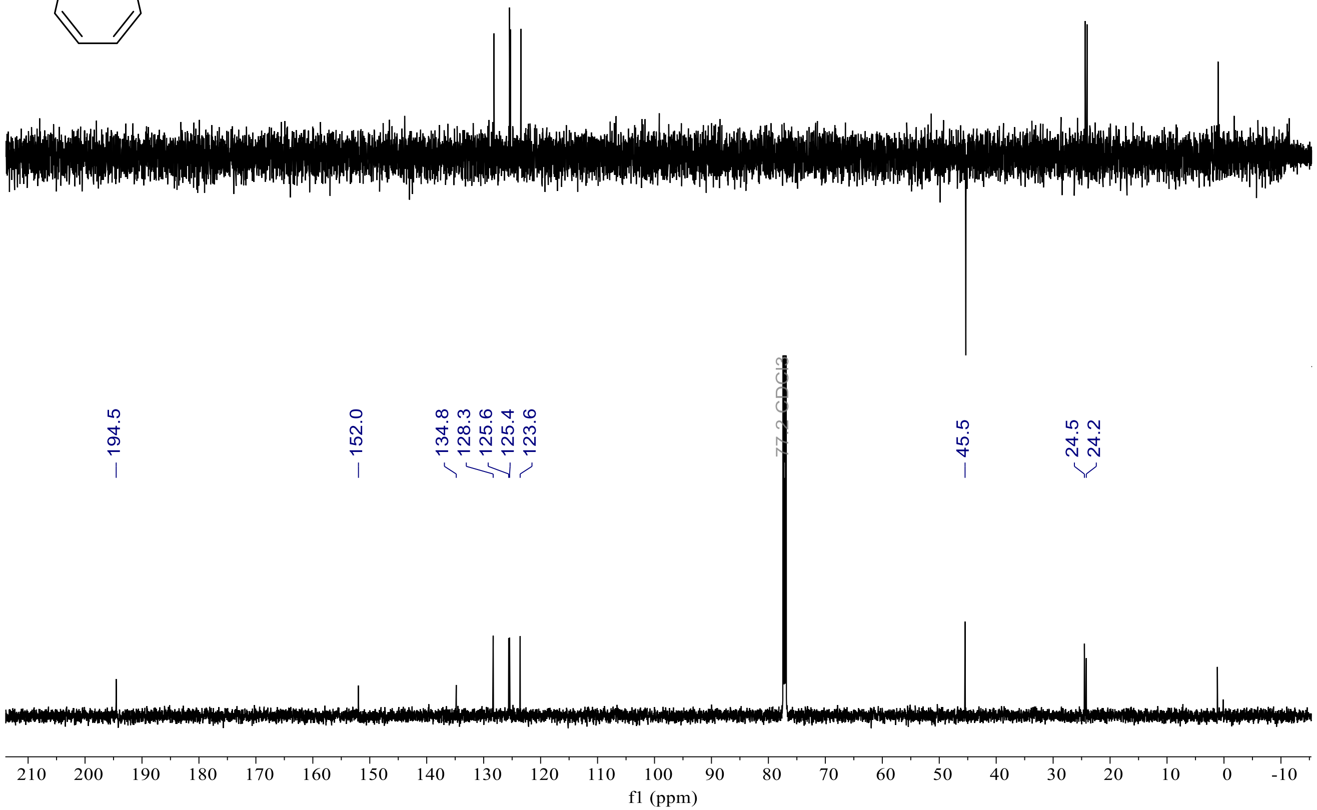

Compound 8j HSQC spectrum (800/200 MHz, $CDCl_3$)

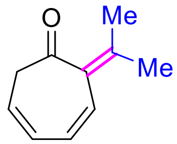

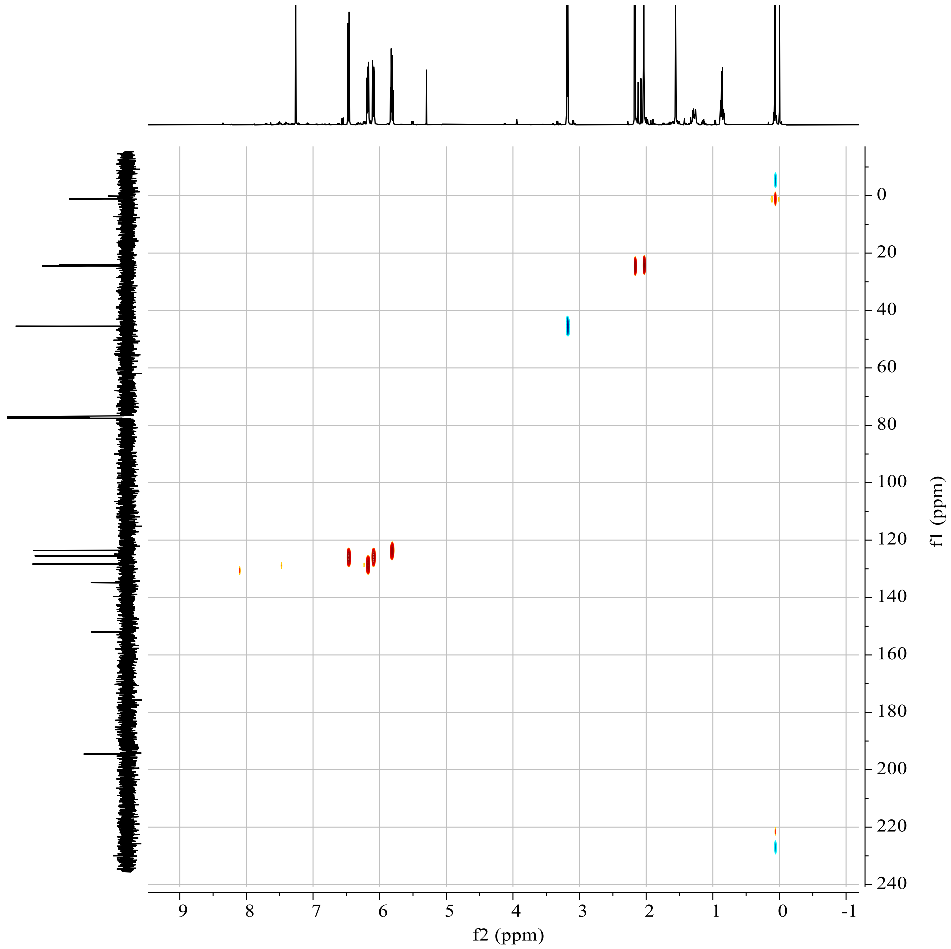

Compound 8j HMBC spectrum (800/200 MHz, $CDCl_3$)

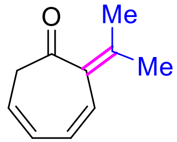

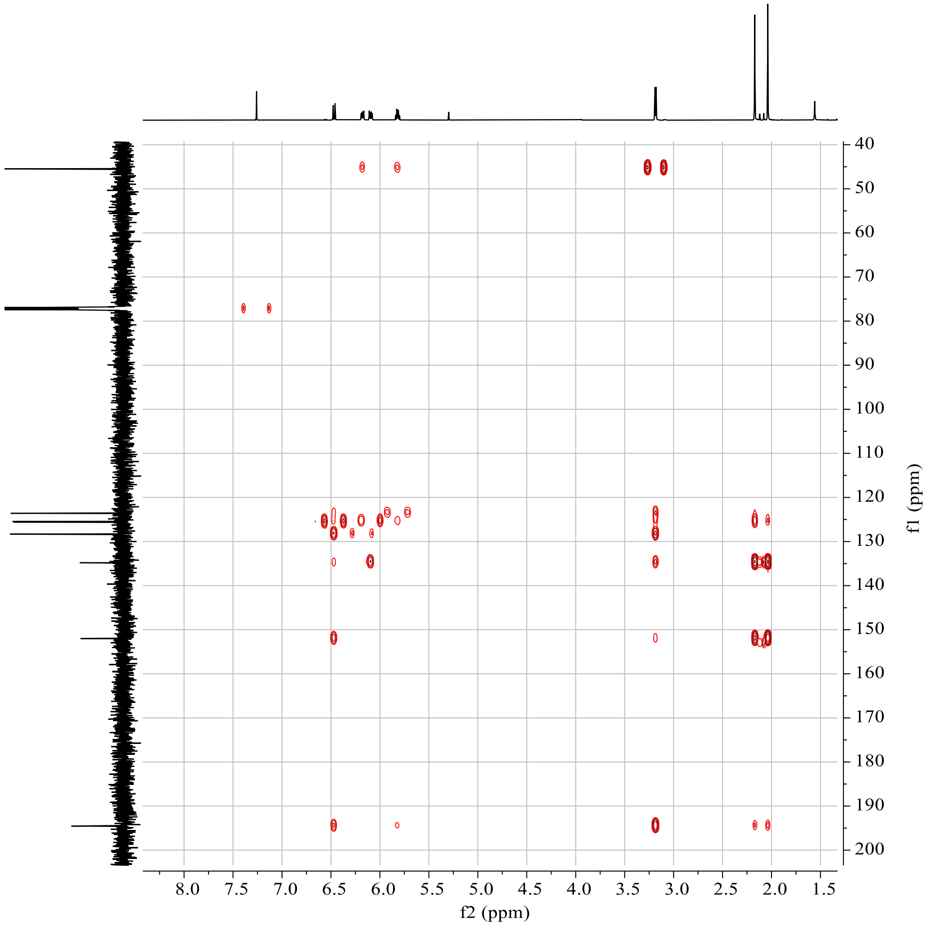

Compound 8j ROESY spectrum (800 MHz, CDCl₃)

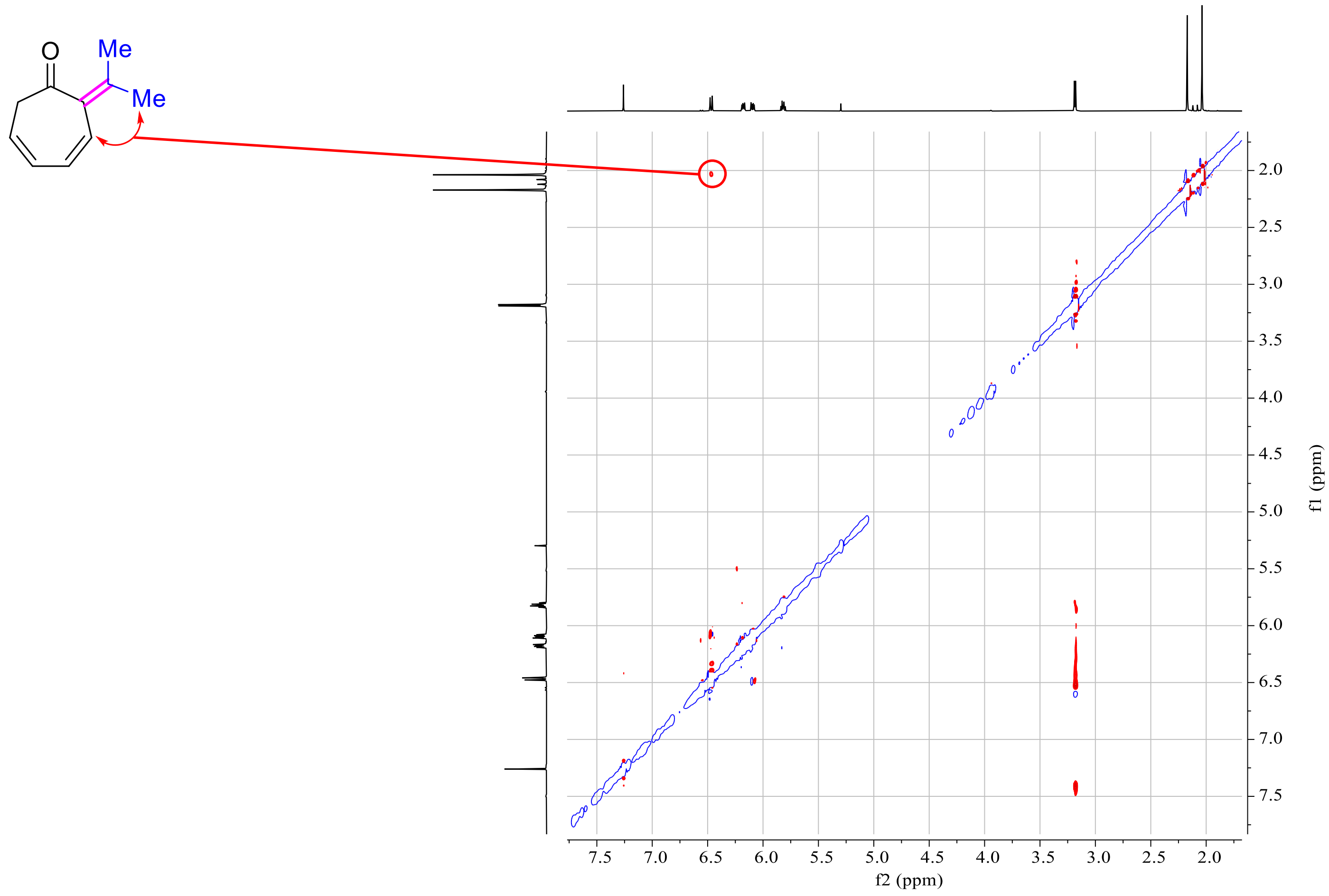

Compound 8j HRMS (ESI-TOF):

**Data Filename**	ESIH202500151-1.d	**Sample Name**	D4-ZL1new
**Sample ID**		**Position**	P1-C6
**Instrument Name**	Agilent 6520 Q-TOF	**Acq Method**	20160322_MS_ESIH_POS_1min.m
**Acquired Time**	1/10/2025 4:16:51 PM	**DA Method**	ESI-HR-20231114.m
**Comment**	ESIH by fangsu		

User Spectra

Fragmentor Voltage	Collision Energy	Ionization Mode
170	0	ESI

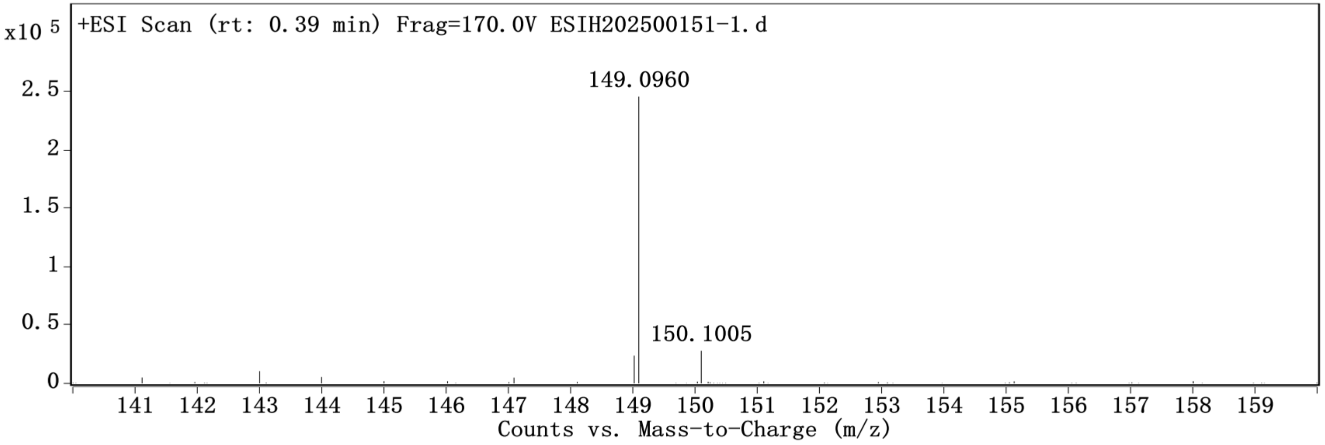

Formula Calculator Results

m/z	Calc m/z	Diff (mDa)	Diff (ppm)	Ion Formula	Ion
149.096	149.0961	0.13	0.9	C10 H13 O	(M+H)+

--- End Of Report ---

Compound 8hs ¹H NMR (600 MHz, CDCl₃)

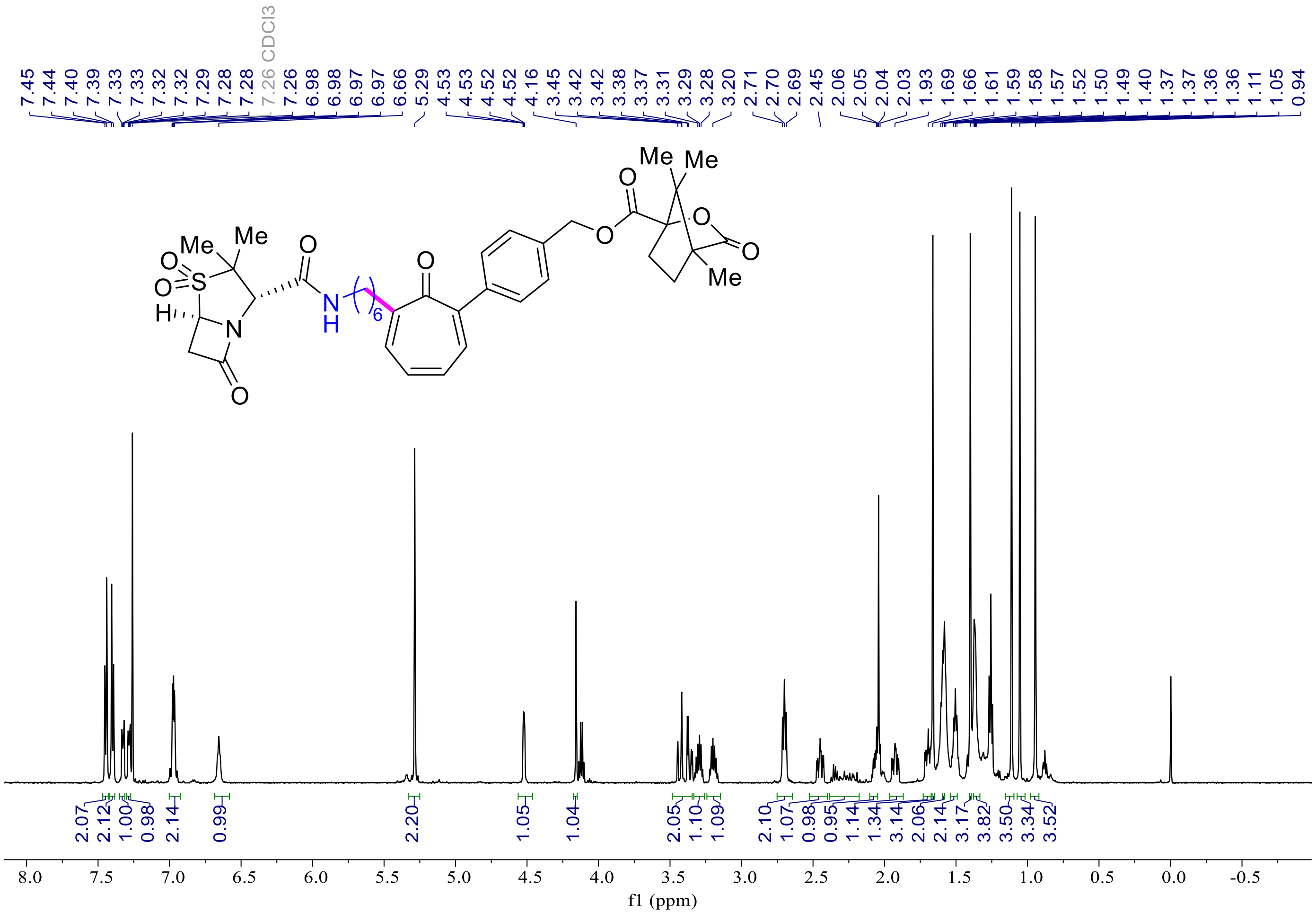

Compound 8hs ^{13}C NMR (125 MHz, $CDCl_3$)

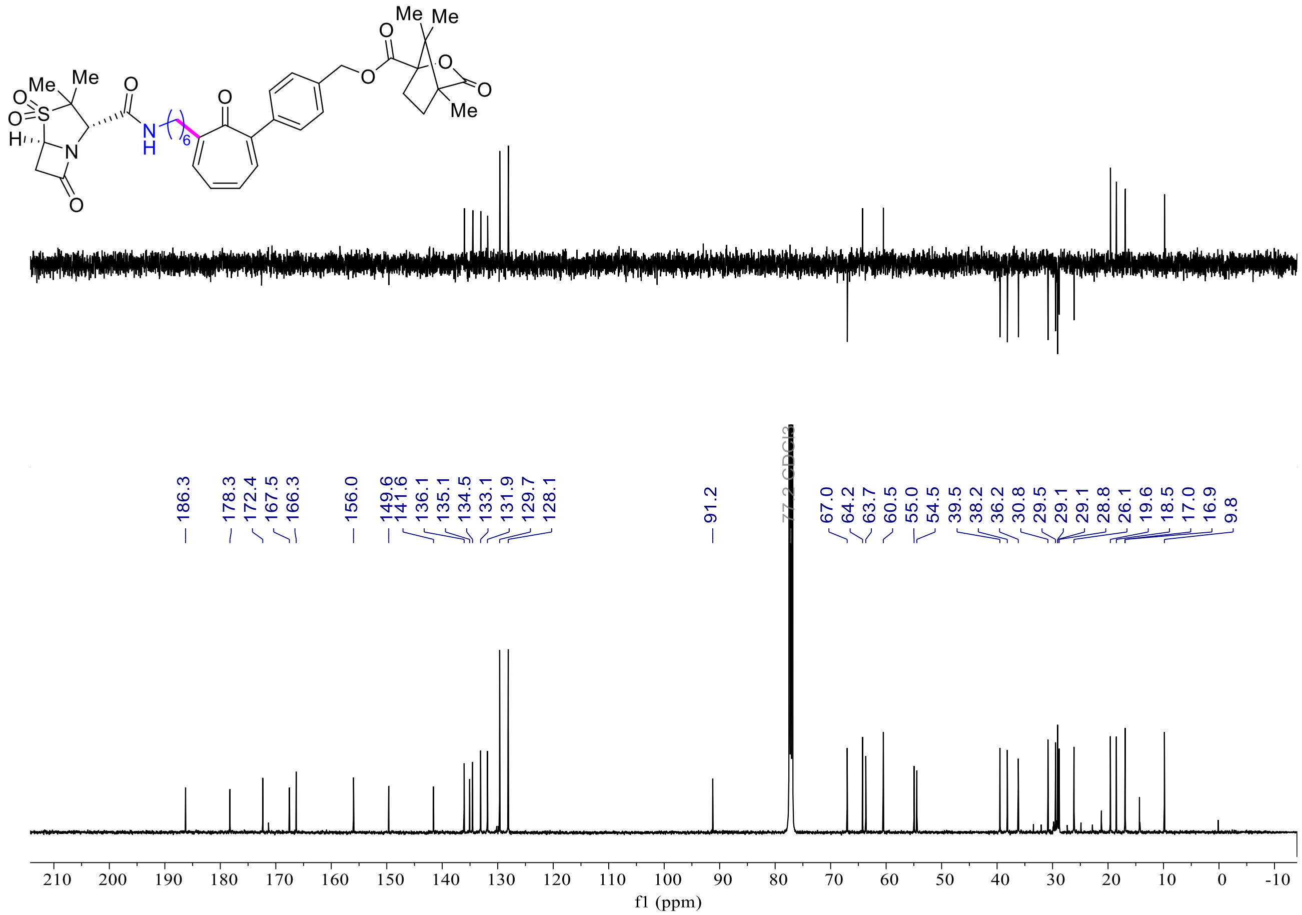

Compound 8hs HRMS (ESI-TOF):

**Data Filename**	ESIH202405931.d	**Sample Name**	D4-ZDG7-3
**Sample ID**		**Position**	P1-C3
**Instrument Name**	Agilent 6520 Q-TOF	**Acq Method**	20160322_MS_ESIH_POS_1min.m
**Acquired Time**	12/13/2024 2:11:25 PM	**DA Method**	ESI-HR-20231114.m
**Comment**	ESIH by fangsu		

User Spectra

Fragmentor Voltage	Collision Energy	Ionization Mode
170	0	ESI

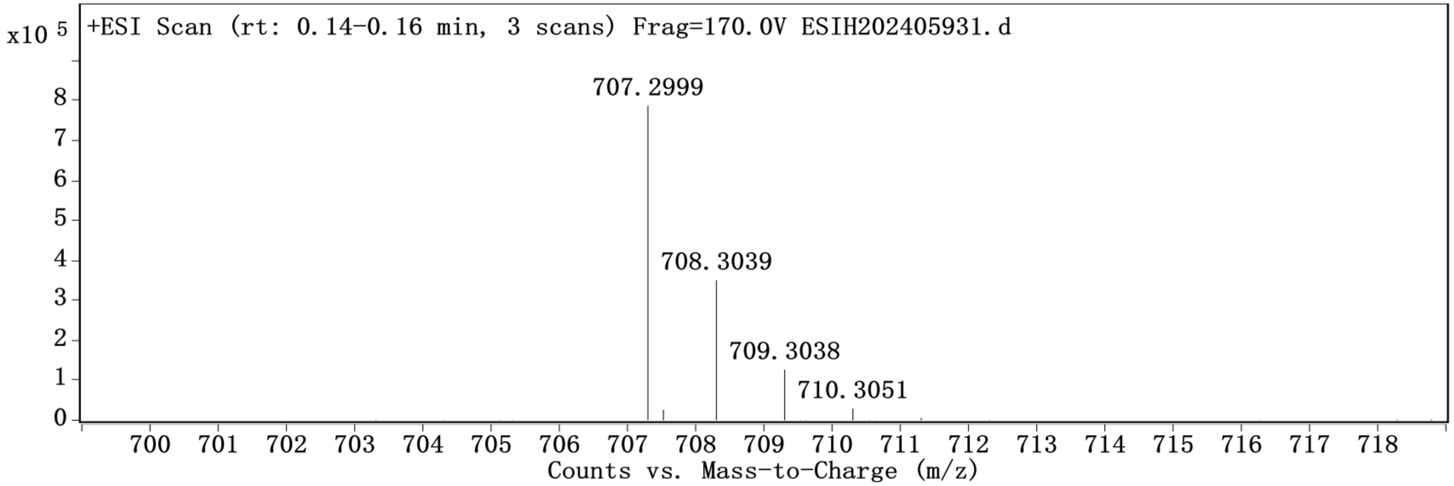

Formula Calculator Results

m/z	Calc m/z	Diff (mDa)	Diff (ppm)	Ion Formula	Ion
707.2999	707.2997	-0.19	-0.28	C38 H47 N2 O9 S	(M+H)+

--- End Of Report ---

Compound 10b ^{1}H NMR (600 MHz, $CDCl_3$)

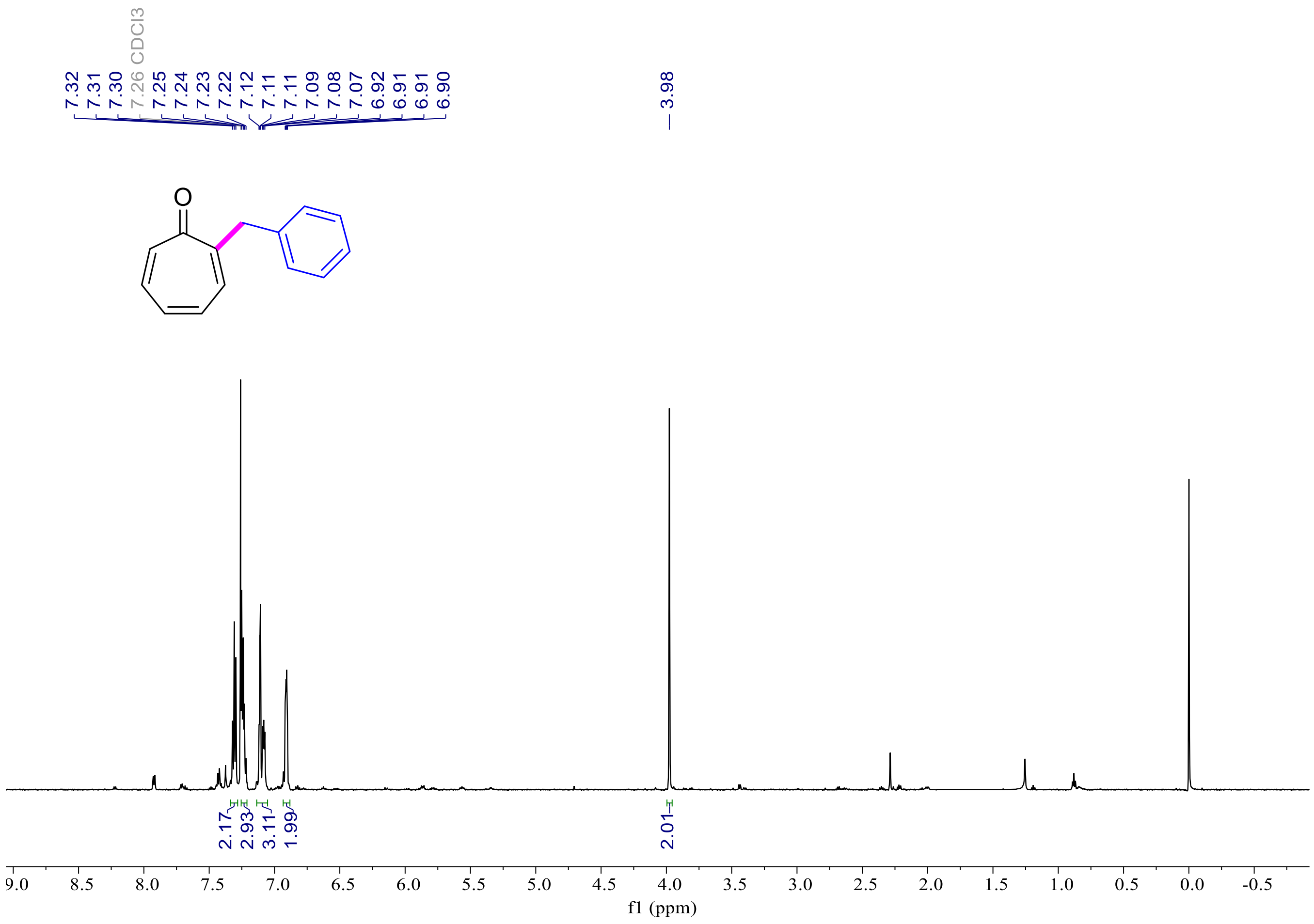

Compound 10b ^{13}C NMR (125 MHz, $CDCl_3$)

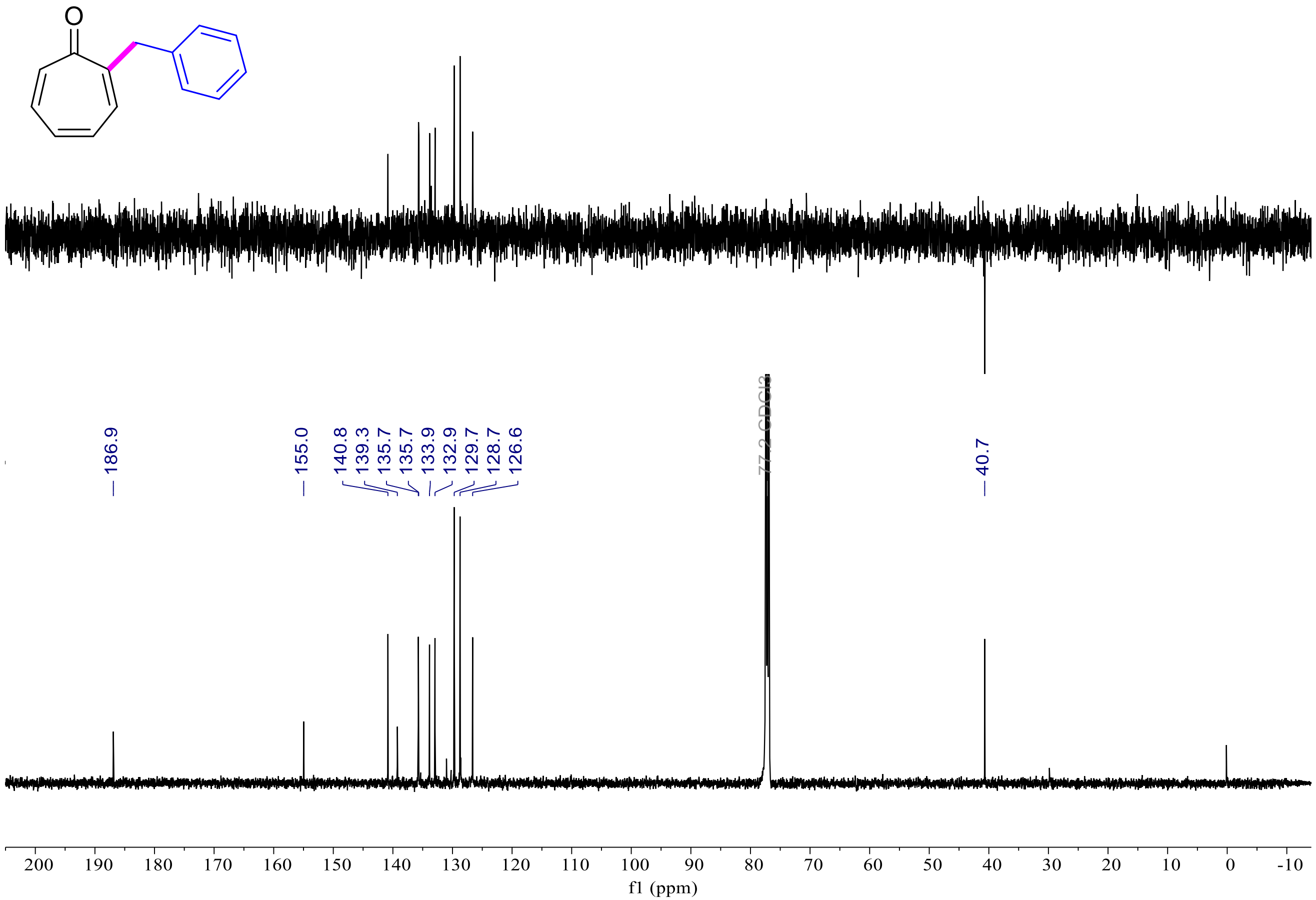

Compound 10b HRMS (ESI-TOF):

**Data Filename**	ESIH202404454.d	**Sample Name**	D4-ZQT21-1A
**Sample ID**		**Position**	P1-B8
**Instrument Name**	Agilent 6520 Q-TOF	**Acq Method**	20160322_MS_ESIH_POS_1min.m
**Acquired Time**	9/13/2024 2:02:01 PM	**DA Method**	ESI-HR-20231114.m
**Comment**	ESIH by fangsu		

User Spectra

Fragmentor Voltage	Collision Energy	Ionization Mode
170	0	ESI

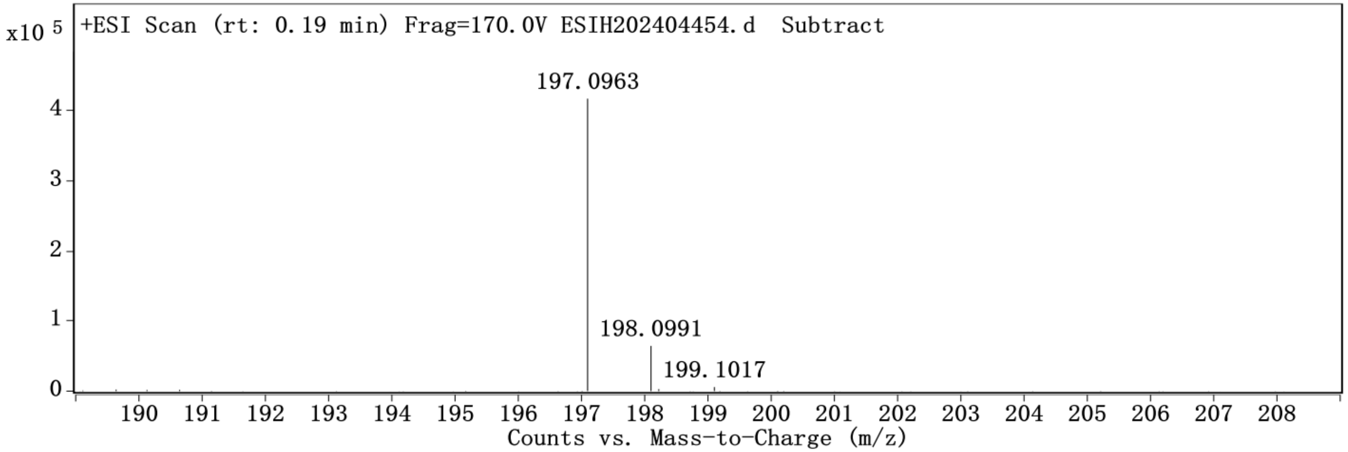

Formula Calculator Results

m/z	Calc m/z	Diff (mDa)	Diff (ppm)	Ion Formula	Ion
197.0963	197.0961	-0.19	-0.97	C14 H13 O	(M+H)+

--- End Of Report ---

Compound 10c ¹H NMR (600 MHz, CDCl₃)

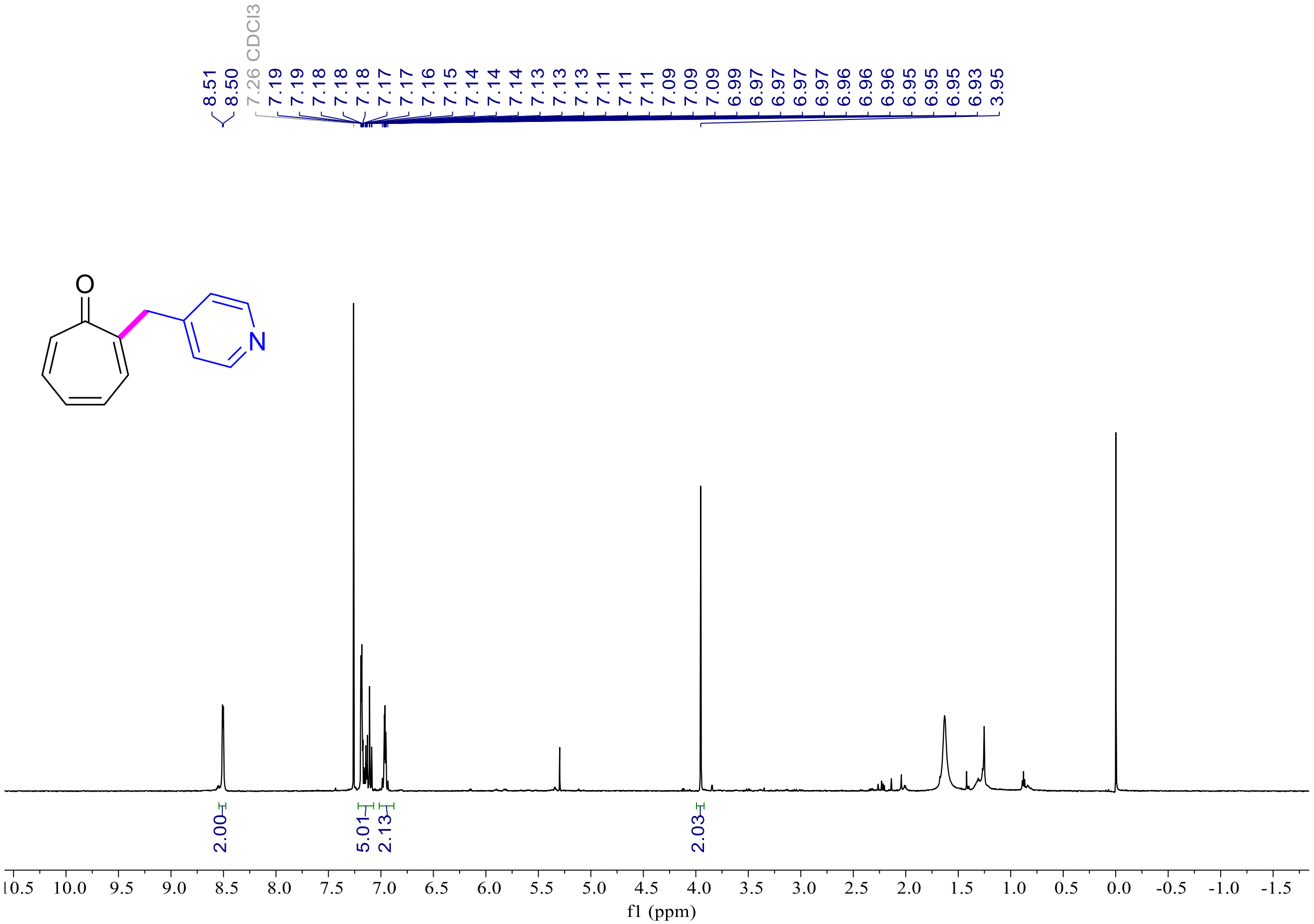

Compound 10c ^{13}C NMR (150 MHz, $CDCl_3$)

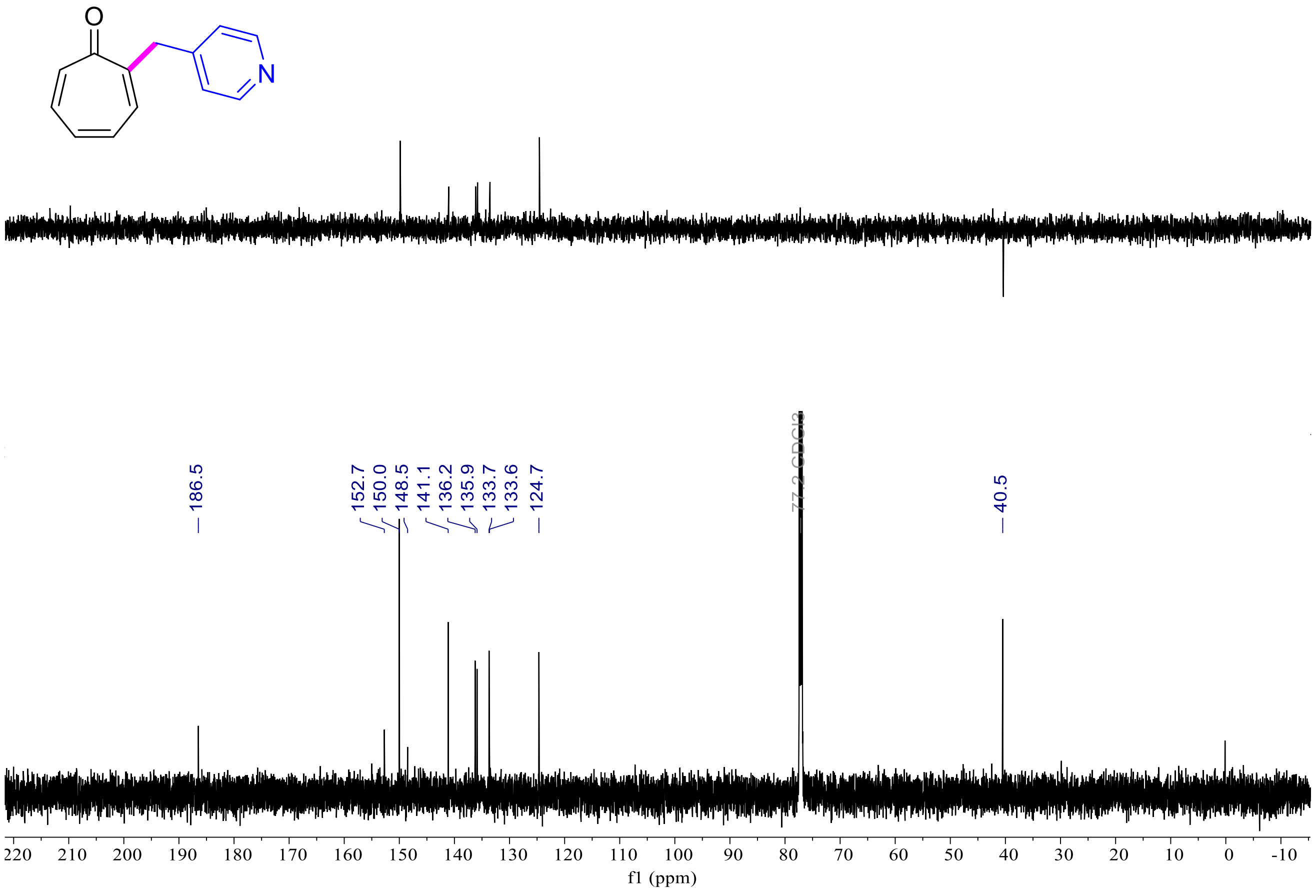

Compound 10c HRMS (ESI-TOF):

**Data Filename**	ESIH202404800.d	**Sample Name**	D4-ZQT18-47B
**Sample ID**		**Position**	P1-B1
**Instrument Name**	Agilent 6520 Q-TOF	**Acq Method**	20160322_MS_ESIH_POS_1min.m
**Acquired Time**	10/15/2024 4:28:33 PM	**DA Method**	ESI-HR-20231114.m
**Comment**	ESIH by fangsu		

User Spectra

Fragmentor Voltage	Collision Energy	Ionization Mode
170	0	ESI

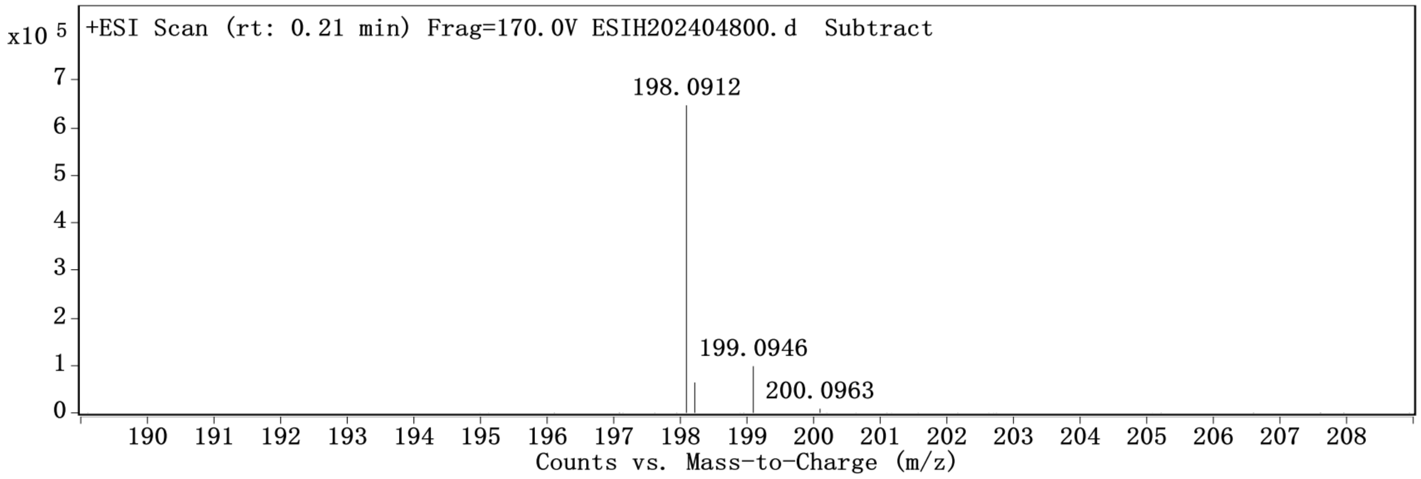

Formula Calculator Results

m/z	Calc m/z	Diff (mDa)	Diff (ppm)	Ion Formula	Ion
198.0912	198.0913	0.19	0.94	C13 H12 N O	(M+H)+

--- End Of Report ---

Compound 10d ^1H NMR (600 MHz, $CDCl_3$)

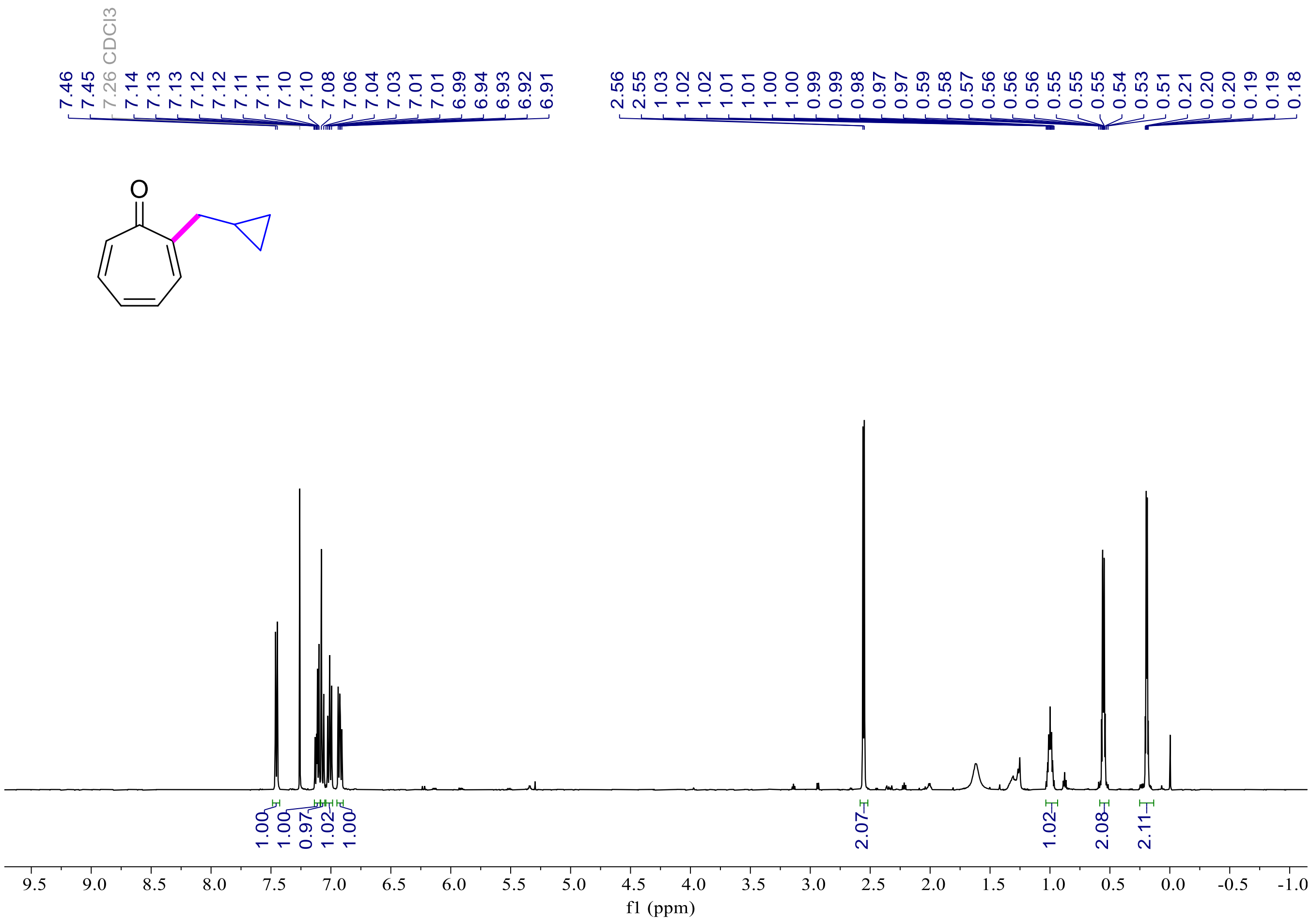

Compound 10d ^{13}C NMR (150 MHz, $CDCl_3$)

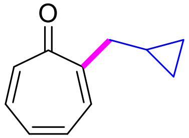

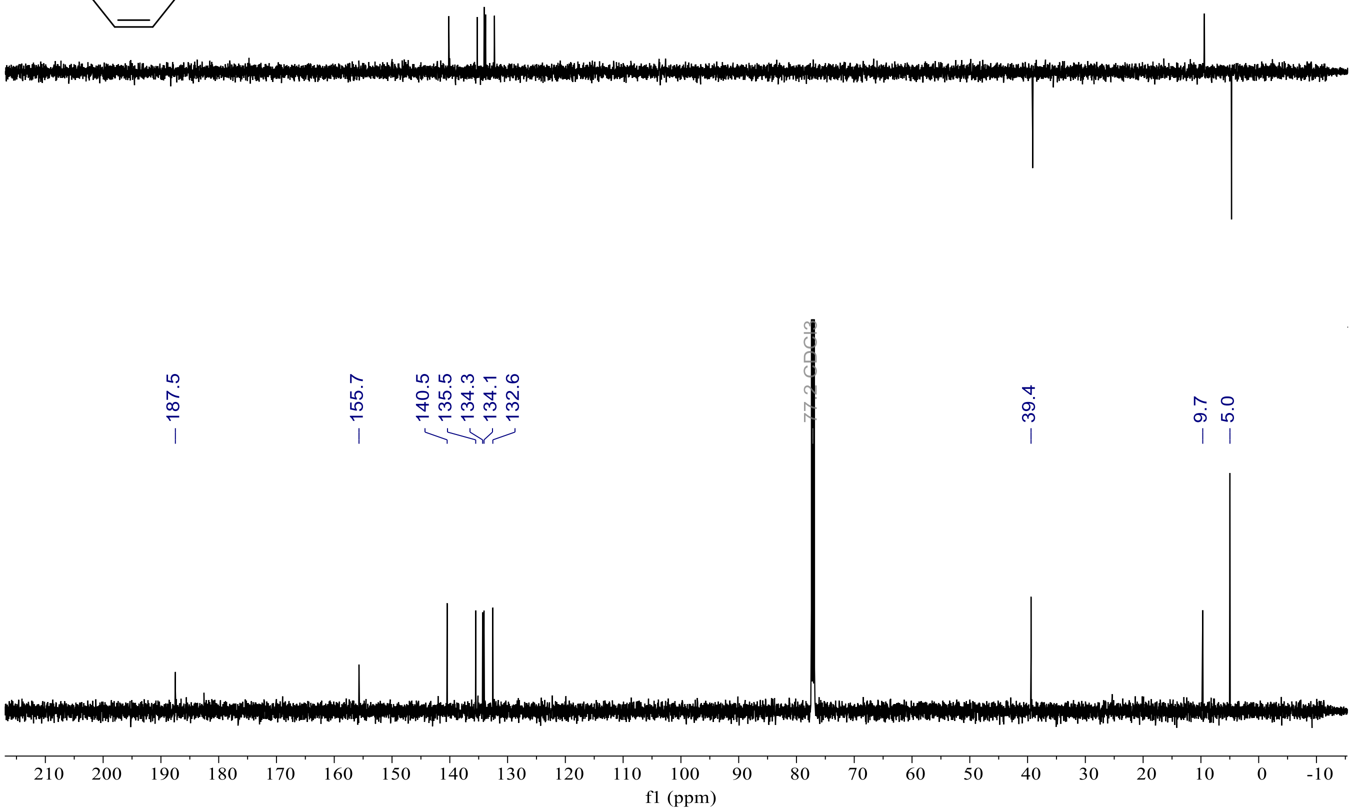

Compound 10d HRMS (ESI-TOF):

**Data Filename**	ESIH202500043.d	**Sample Name**	D4-ZDG3-13A
**Sample ID**		**Position**	P1-A3
**Instrument Name**	Agilent 6520 Q-TOF	**Acq Method**	20160322_MS_ESIH_POS_1min.m
**Acquired Time**	1/3/2025 3:54:04 PM	**DA Method**	ESI-HR-20231114.m
**Comment**	ESIH by fangsu		

User Spectra

Fragmentor Voltage	Collision Energy	Ionization Mode
170	0	ESI

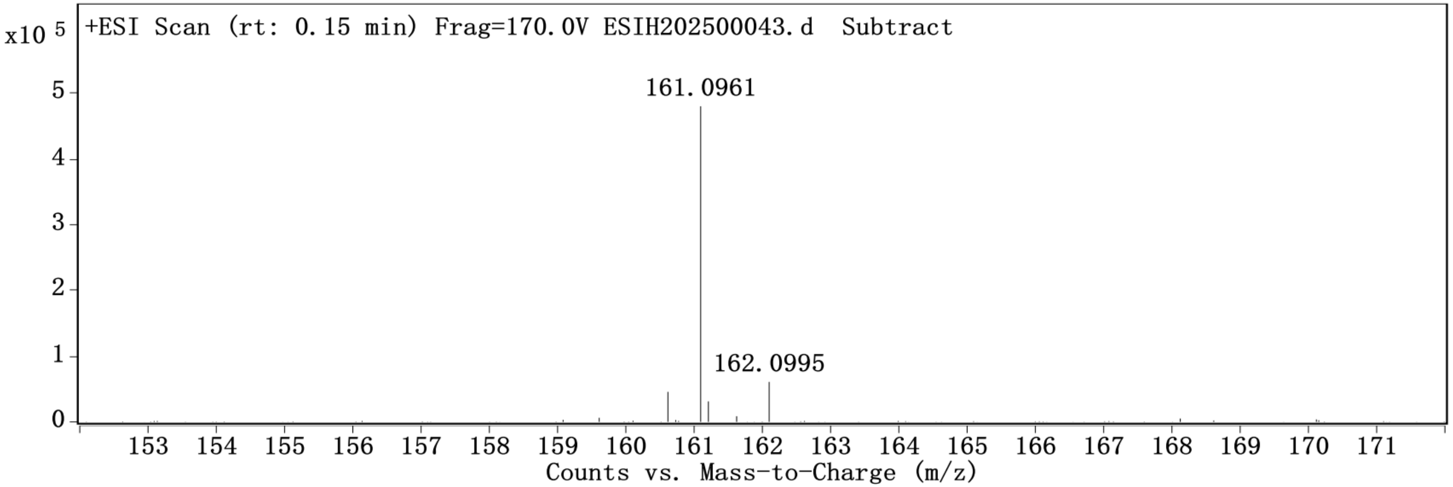

Formula Calculator Results

m/z	Calc m/z	Diff (mDa)	Diff (ppm)	Ion Formula	Ion
161.0961	161.0961	0.02	0.13	C11 H13 O	(M+H)+

--- End Of Report ---

Compound 10ka ¹H NMR (600 MHz, $CDCl_3$)

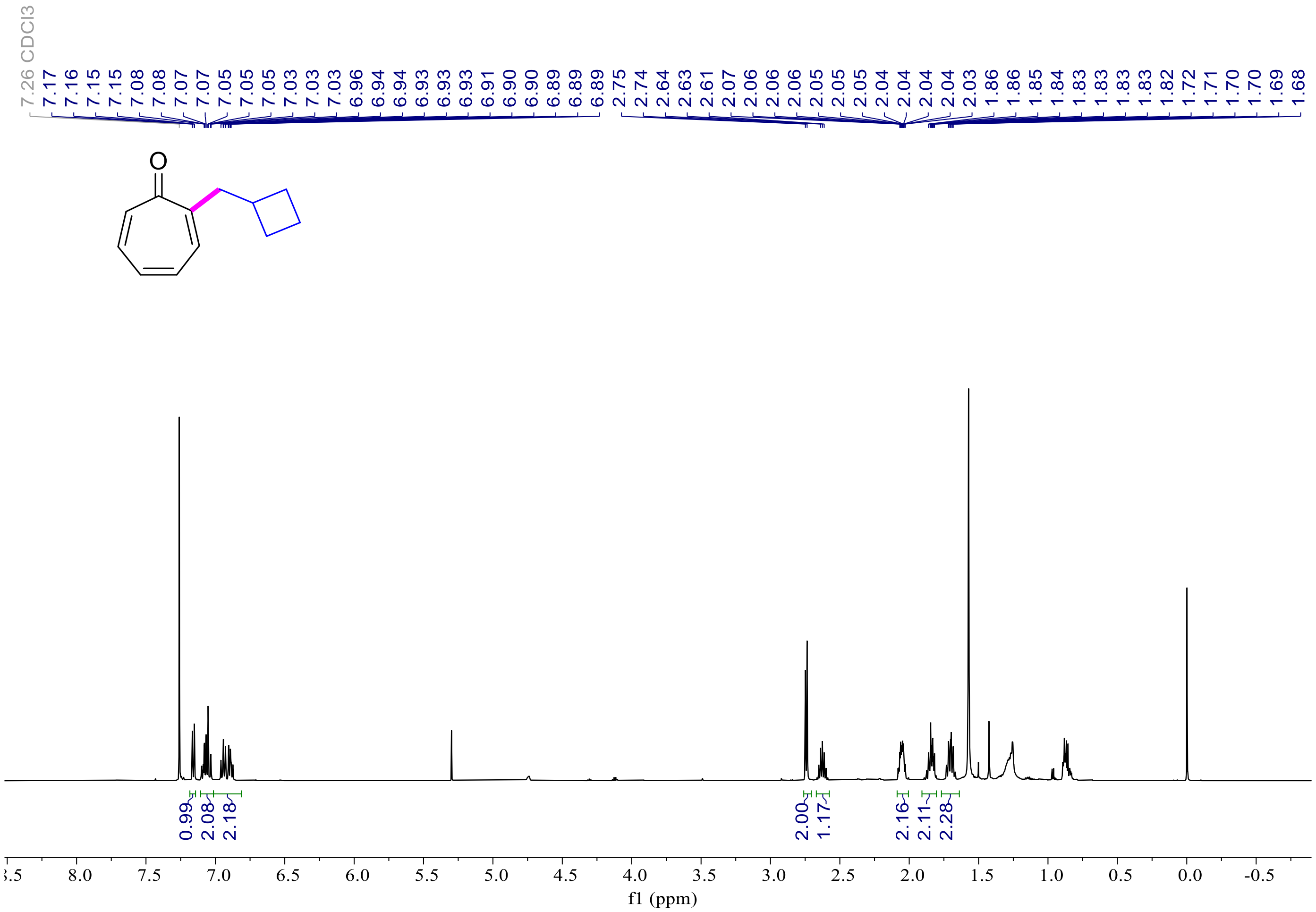

Compound 10ka ^{13}C NMR (150 MHz, $CDCl_3$)

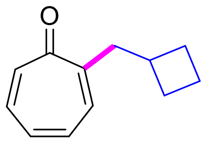

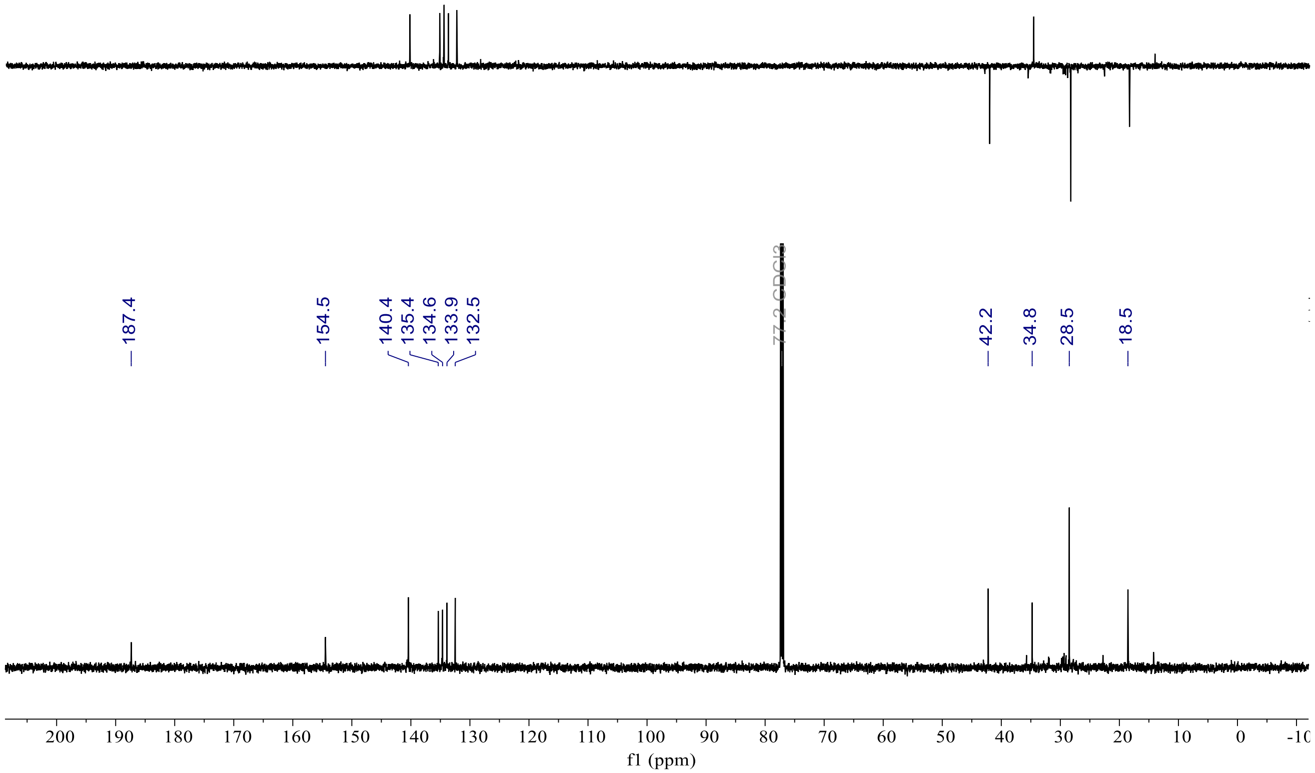

Compound 10ka HRMS (ESI-TOF):

**Data Filename**	ESIH202405035.d	**Sample Name**	D4-ZQSq4B
**Sample ID**		**Position**	P1-D2
**Instrument Name**	Agilent 6520 Q-TOF	**Acq Method**	20160322_MS_ESIH_POS_1min.m
**Acquired Time**	10/24/2024 3:10:24 PM	**DA Method**	ESI-HR-20231114.m
**Comment**	ESIH by fangsu		

User Spectra

Fragmentor Voltage	Collision Energy	Ionization Mode
170	0	ESI

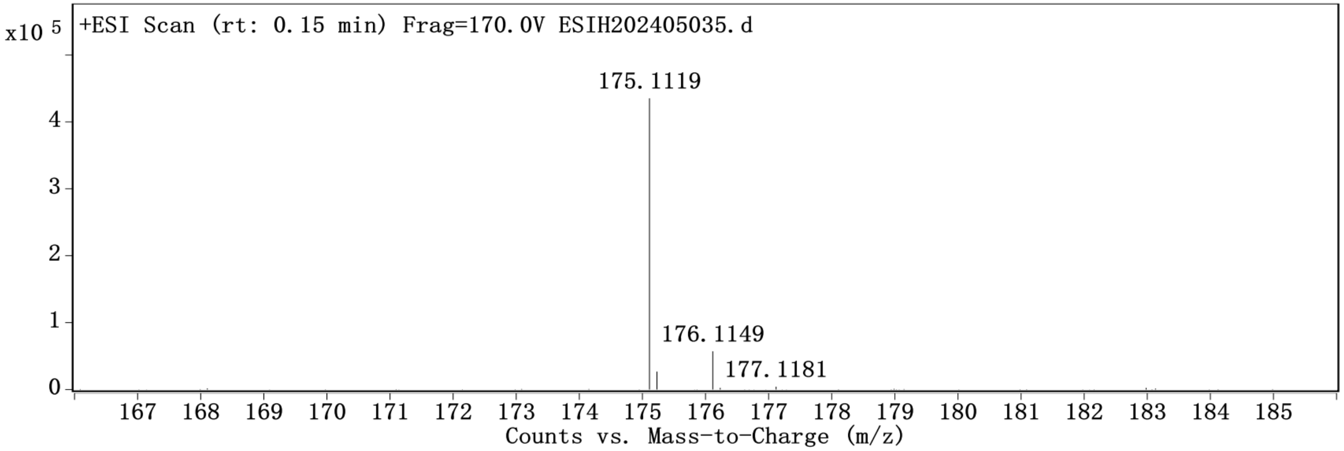

Formula Calculator Results

m/z	Calc m/z	Diff (mDa)	Diff (ppm)	Ion Formula	Ion
175.1119	175.1117	-0.18	-1.04	C12 H15 O	(M+H)+

--- End Of Report ---

Compound 10kb ¹H NMR (600 MHz, CDCl₃)

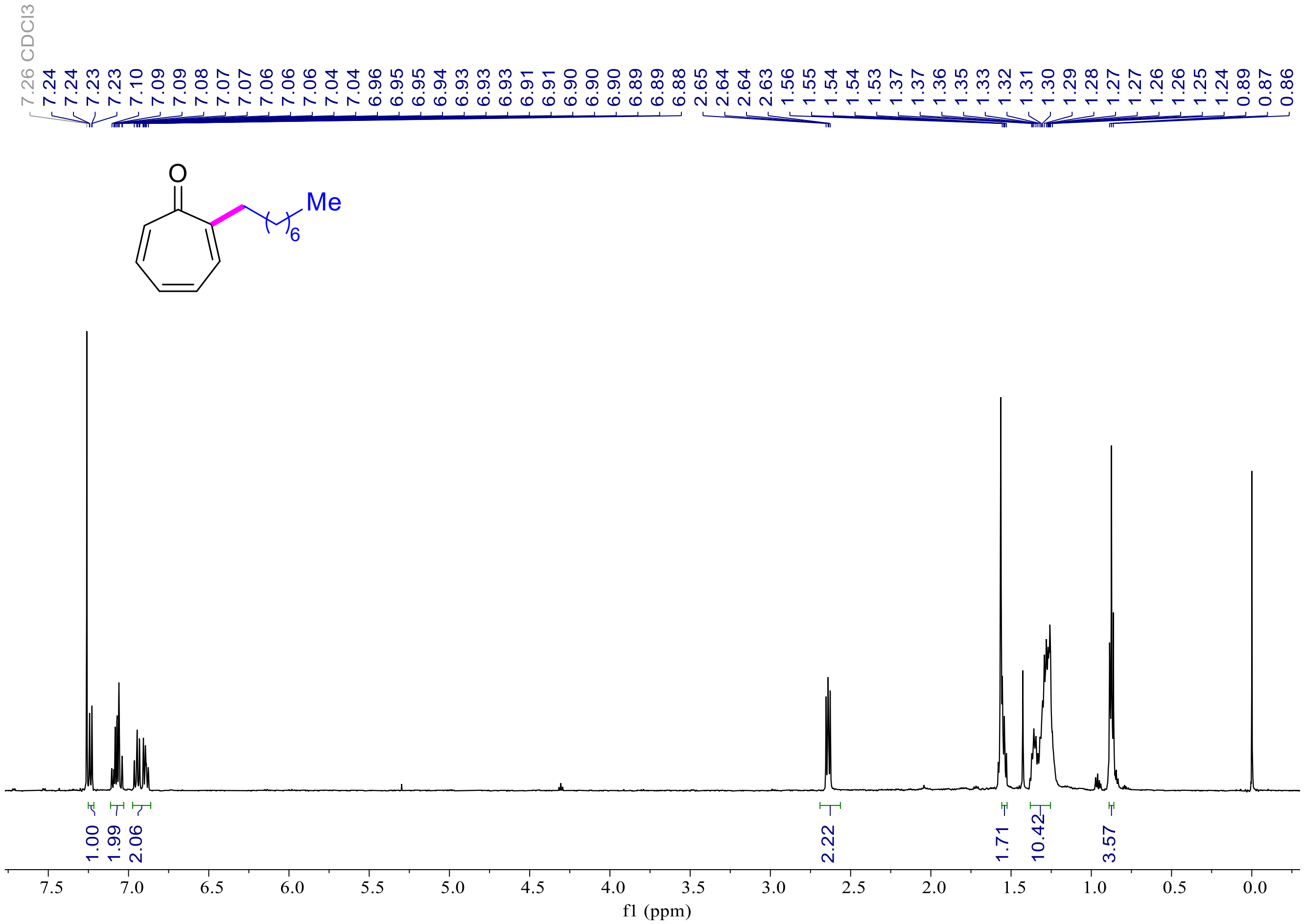

Compound 10kb ^{13}C NMR (150 MHz, $CDCl_3$)

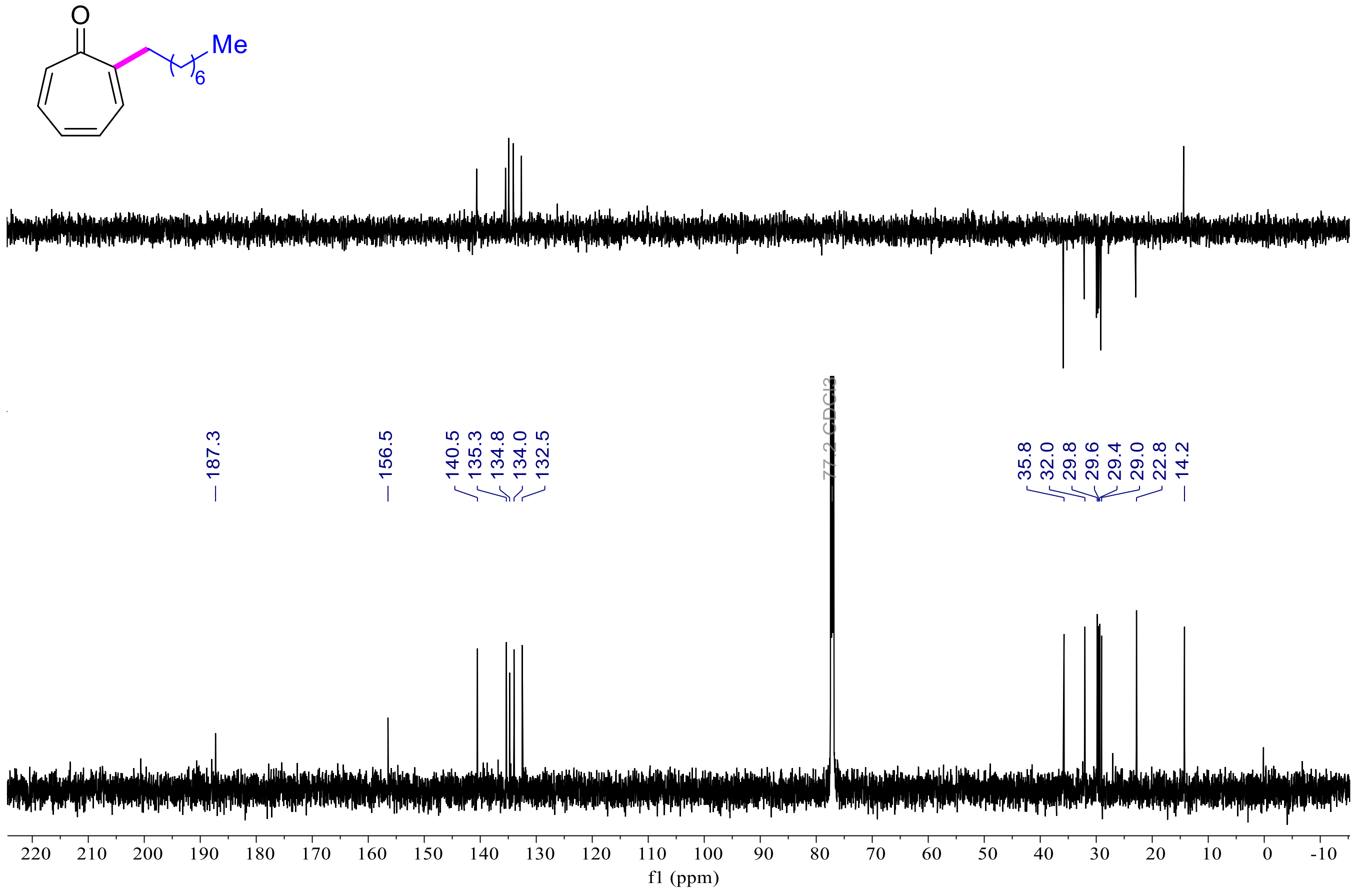

Compound 10kb HRMS (ESI-TOF):

**Data Filename**	ESIH202405034.d	**Sample Name**	D4-ZQSq4A
**Sample ID**		**Position**	P1-D1
**Instrument Name**	Agilent 6520 Q-TOF	**Acq Method**	20160322_MS_ESIH_POS_1min.m
**Acquired Time**	10/24/2024 3:09:08 PM	**DA Method**	ESI-HR-20231114.m
**Comment**	ESIH by fangsu		

User Spectra

Fragmentor Voltage	Collision Energy	Ionization Mode
170	0	ESI

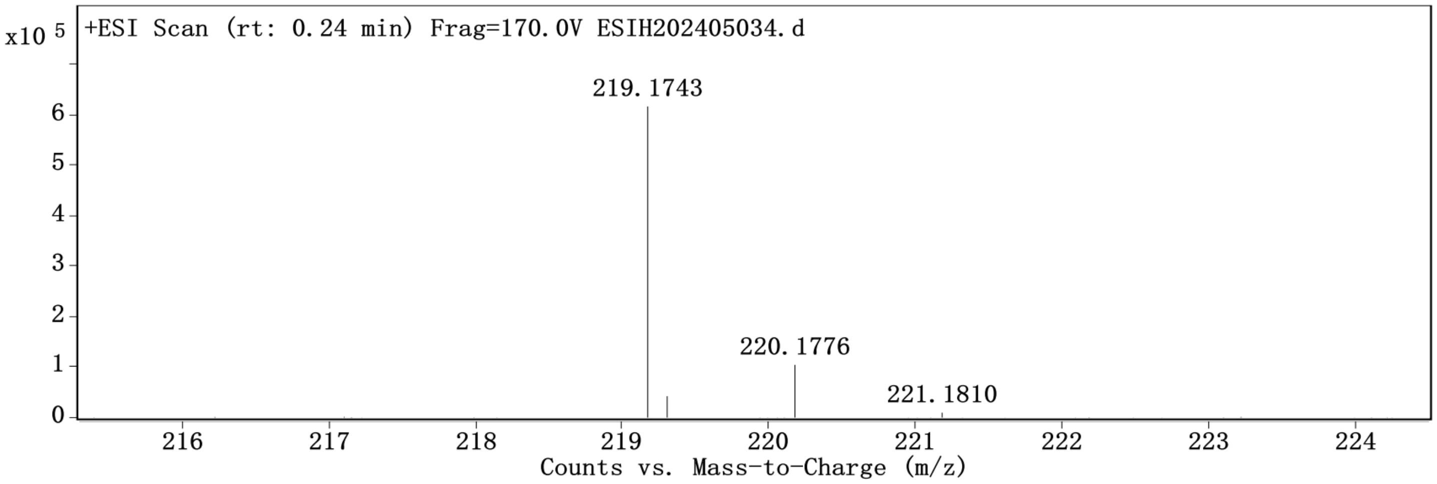

Formula Calculator Results

m/z	Calc m/z	Diff (mDa)	Diff (ppm)	Ion Formula	Ion
219.1743	219.1743	0.04	0.18	C15 H23 O	(M+H)+

--- End Of Report ---

Compound 10n ^{1}H NMR (400 MHz, CDCl3)

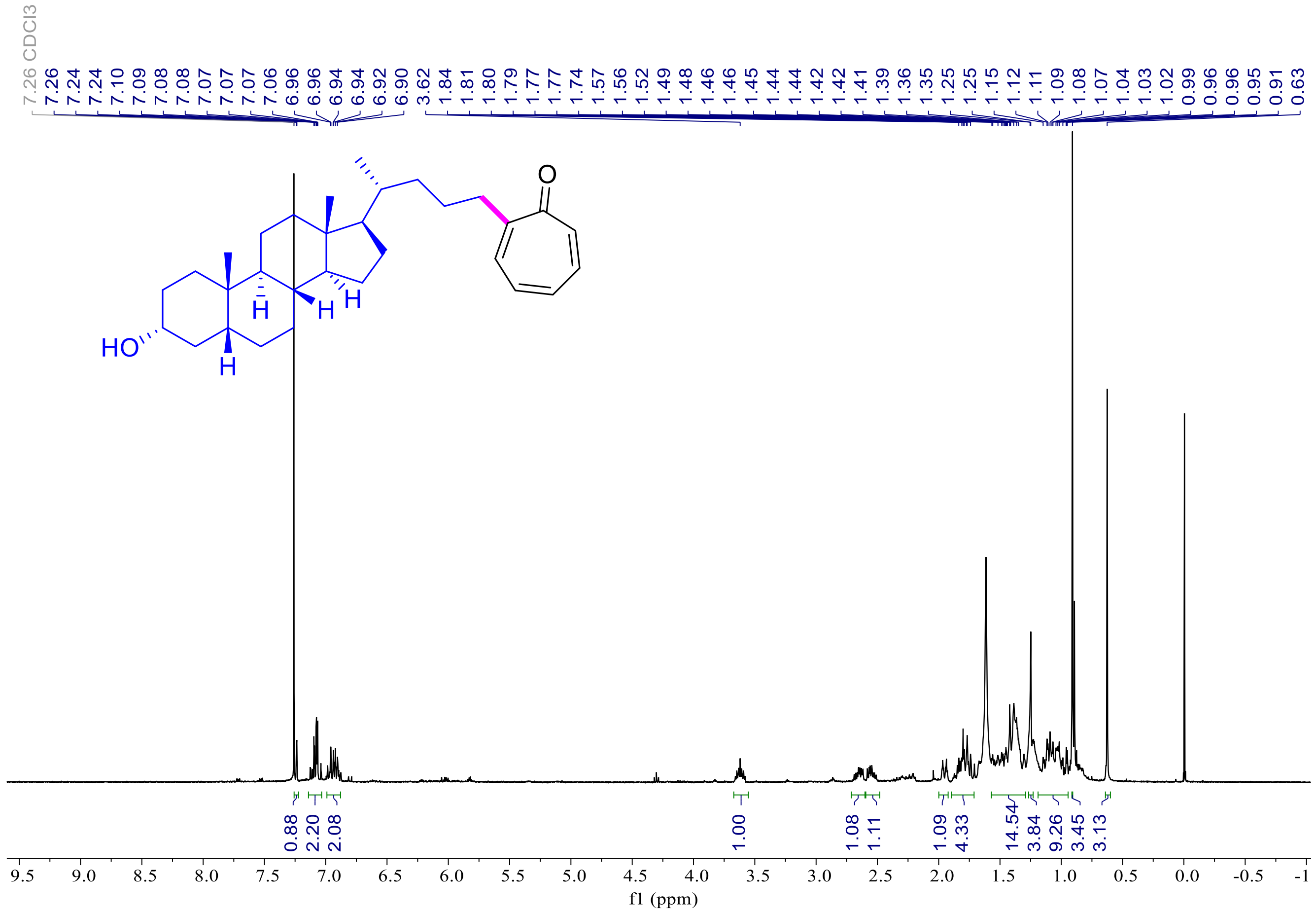

Compound 10n ^{13}C NMR (150 MHz, CDCl$_3$)

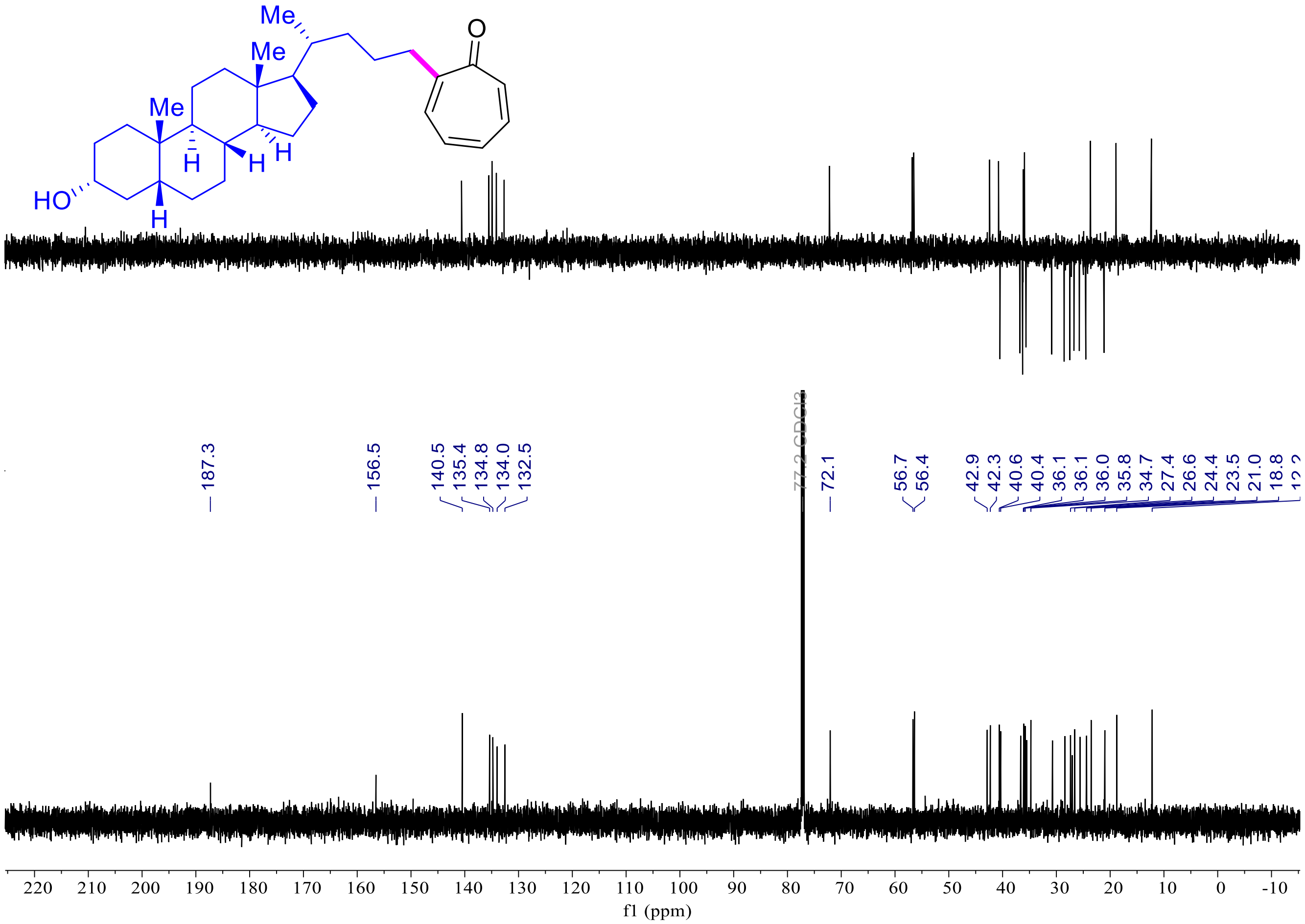

Compound 10n HRMS (ESI-TOF):

**Data Filename**	ESIH202404801.d	**Sample Name**	D4-ZQSt5T3
**Sample ID**		**Position**	P1-B2
**Instrument Name**	Agilent 6520 Q-TOF	**Acq Method**	20160322_MS_ESIH_POS_1min.m
**Acquired Time**	10/15/2024 4:29:51 PM	**DA Method**	ESI-HR-20231114.m
**Comment**	ESIH by fangsu		

User Spectra

Fragmentor Voltage	Collision Energy	Ionization Mode
170	0	ESI

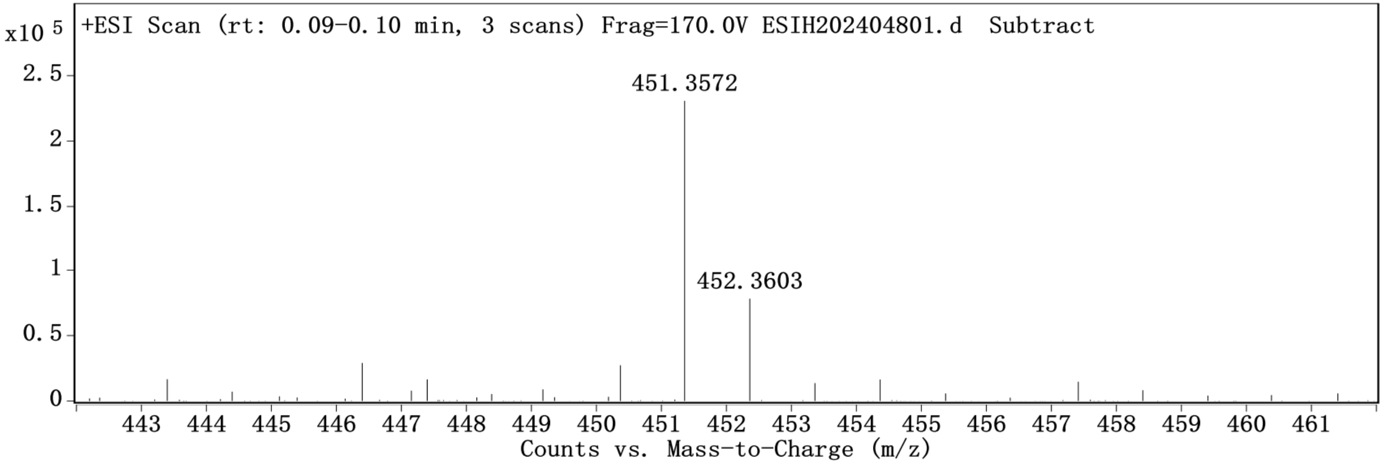

Formula Calculator Results

m/z	Calc m/z	Diff (mDa)	Diff (ppm)	Ion Formula	Ion
451.3572	451.3571	-0.17	-0.37	C31 H47 O2	(M+H)+

--- End Of Report ---

Compound 13 ^{1}H NMR (600 MHz, $CDCl_3$)

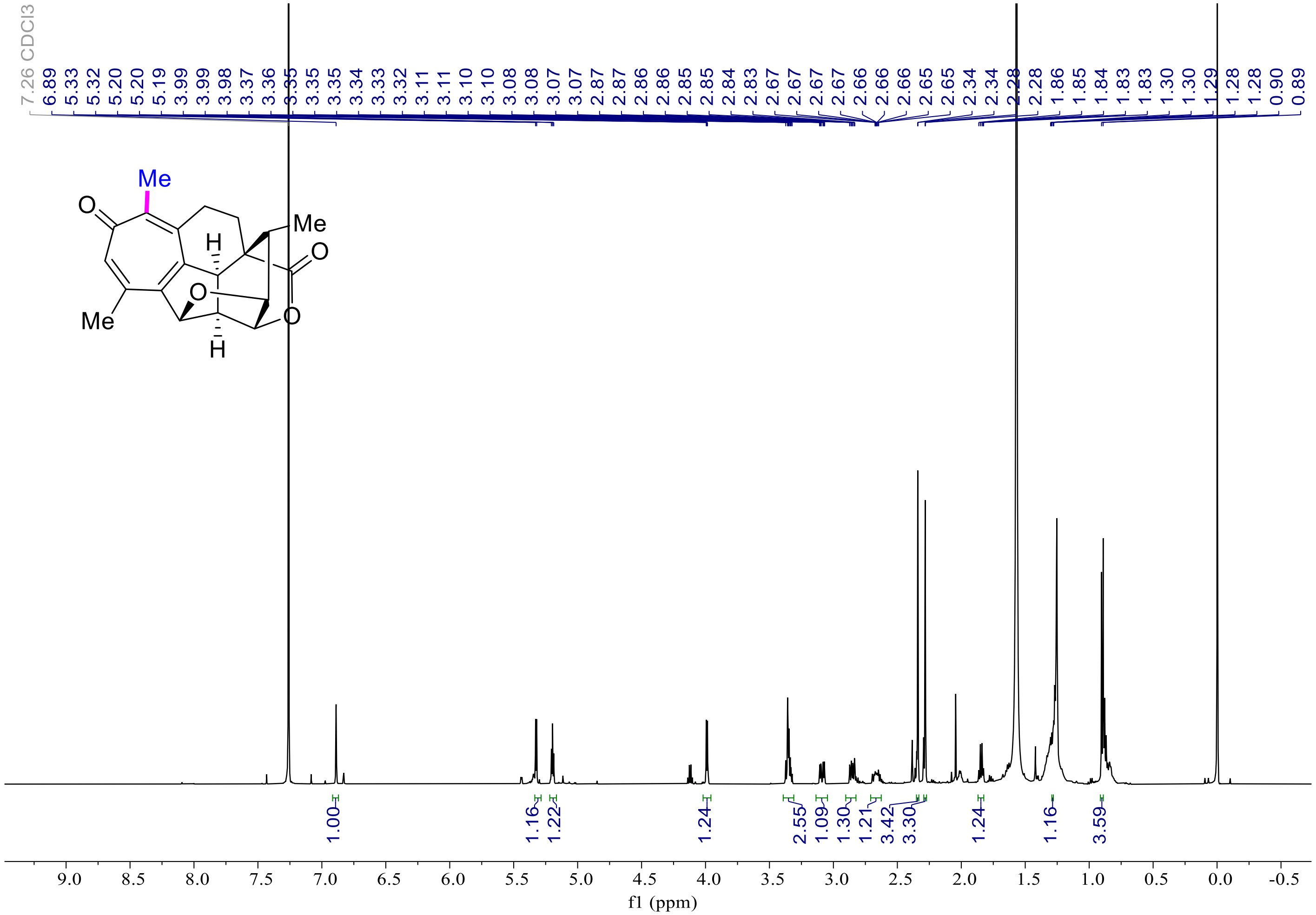

Compound 13 ^{13}C NMR (125 MHz, $CDCl_3$)

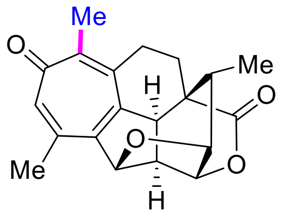

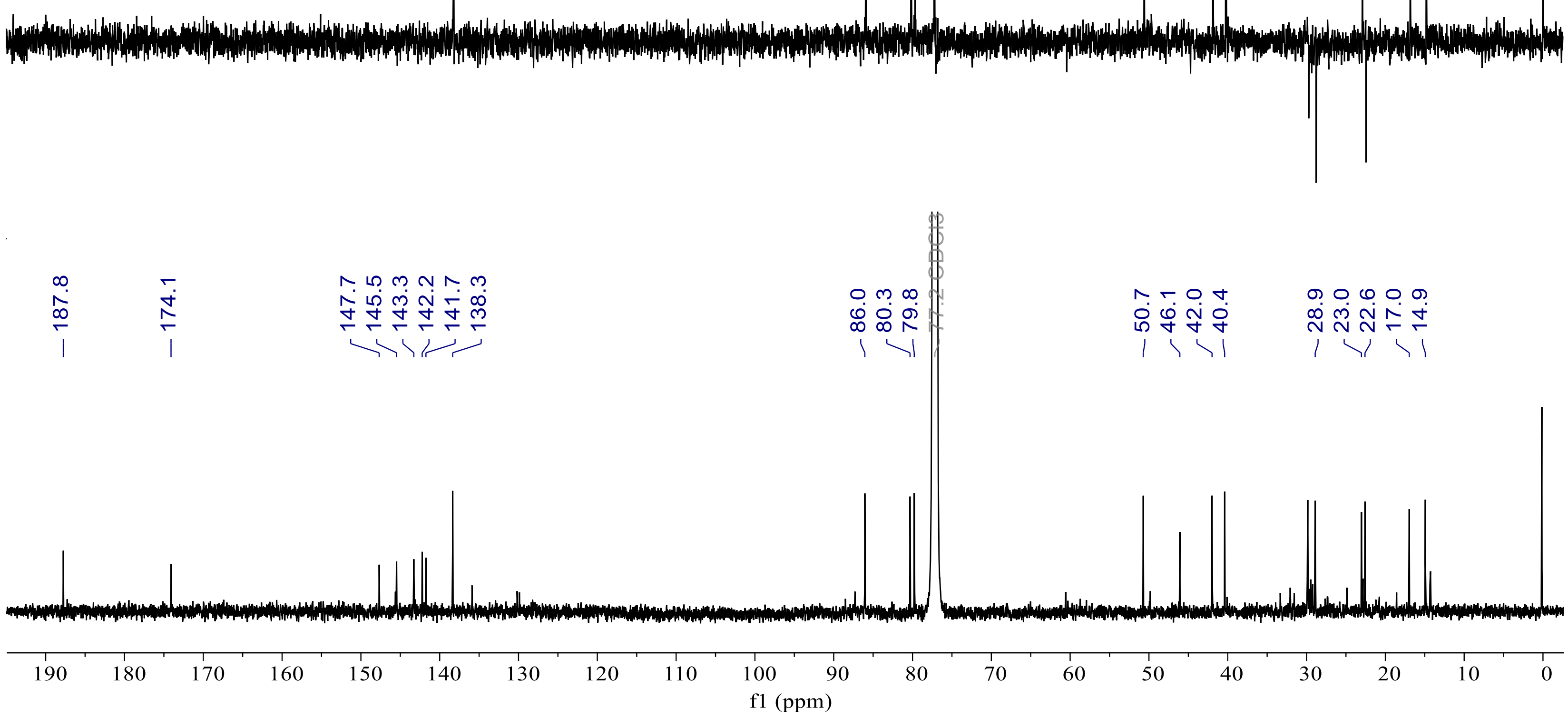

Compound 13 HRMS (ESI-TOF)

**Data Filename**	ESIH202404026.d	**Sample Name**	D4-ZQT15-1B
**Sample ID**		**Position**	P1-A3
**Instrument Name**	Agilent 6520 Q-TOF	**Acq Method**	20160322_MS_ESIH_POS_1min.m
**Acquired Time**	8/16/2024 2:52:41 PM	**DA Method**	ESI-HR-20231114.m
**Comment**	ESIH by fangsu		

User Spectra

Fragmentor Voltage	Collision Energy	Ionization Mode
170	0	ESI

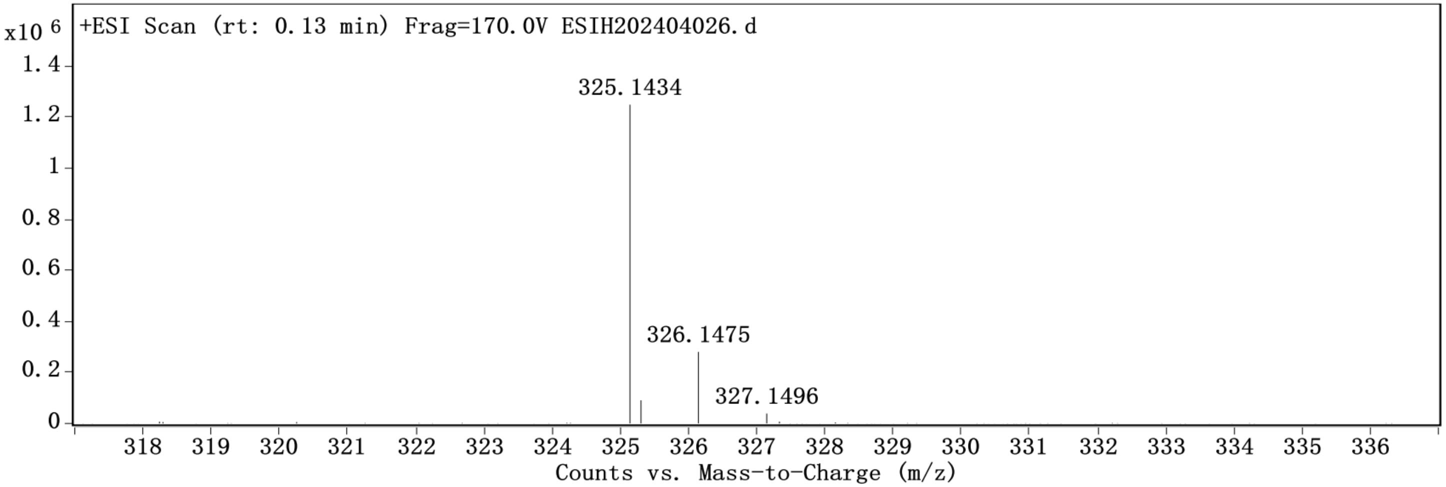

Formula Calculator Results

m/z	Calc m/z	Diff (mDa)	Diff (ppm)	Ion Formula	Ion
325.1434	325.1434	0.04	0.12	C20 H21 O4	(M+H)+

--- End Of Report ---

Compound 14 ^{1}H NMR (600 MHz, $CDCl_3$)

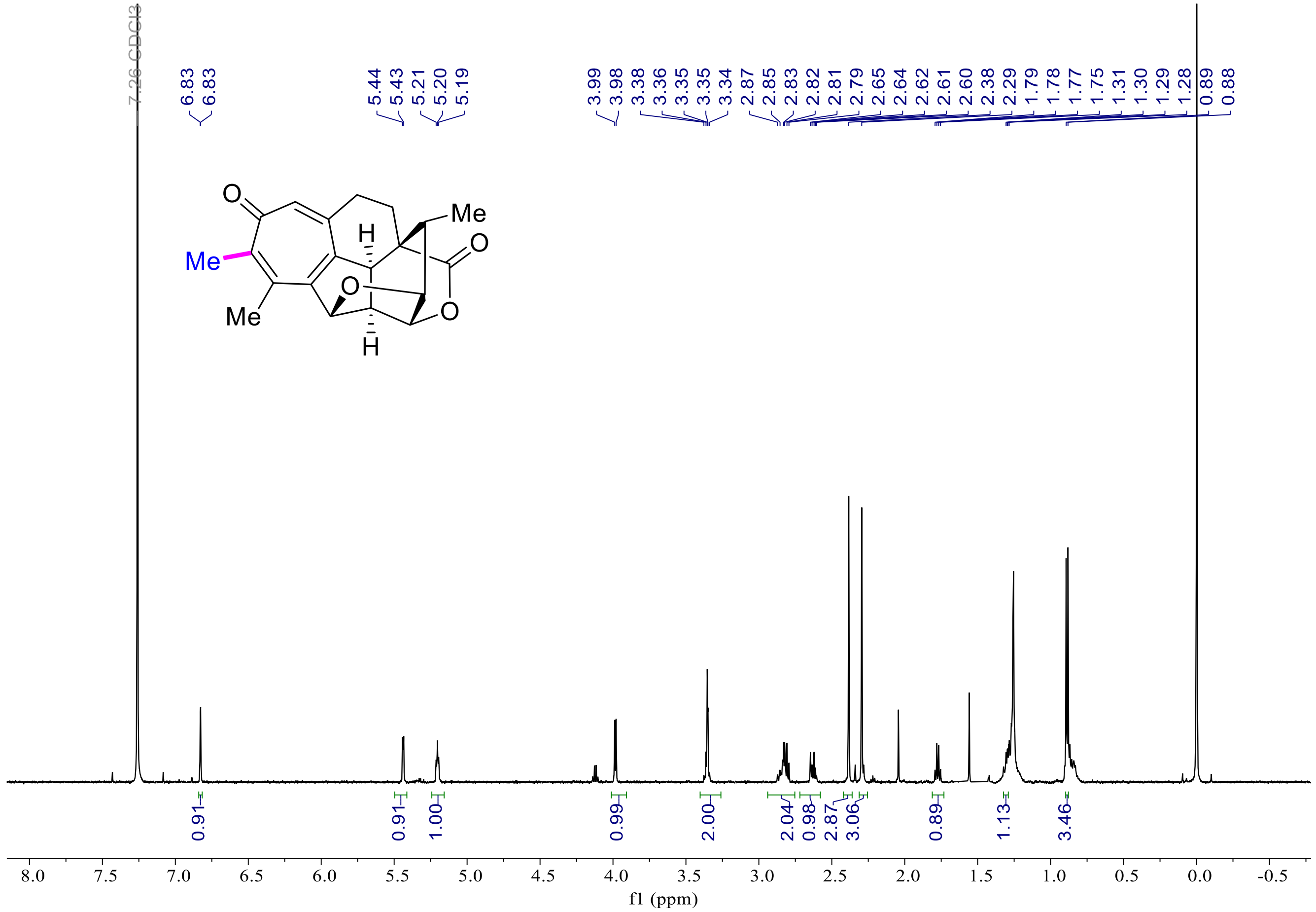

Compound 14 ^{13}C NMR (125 MHz, $CDCl_3$)

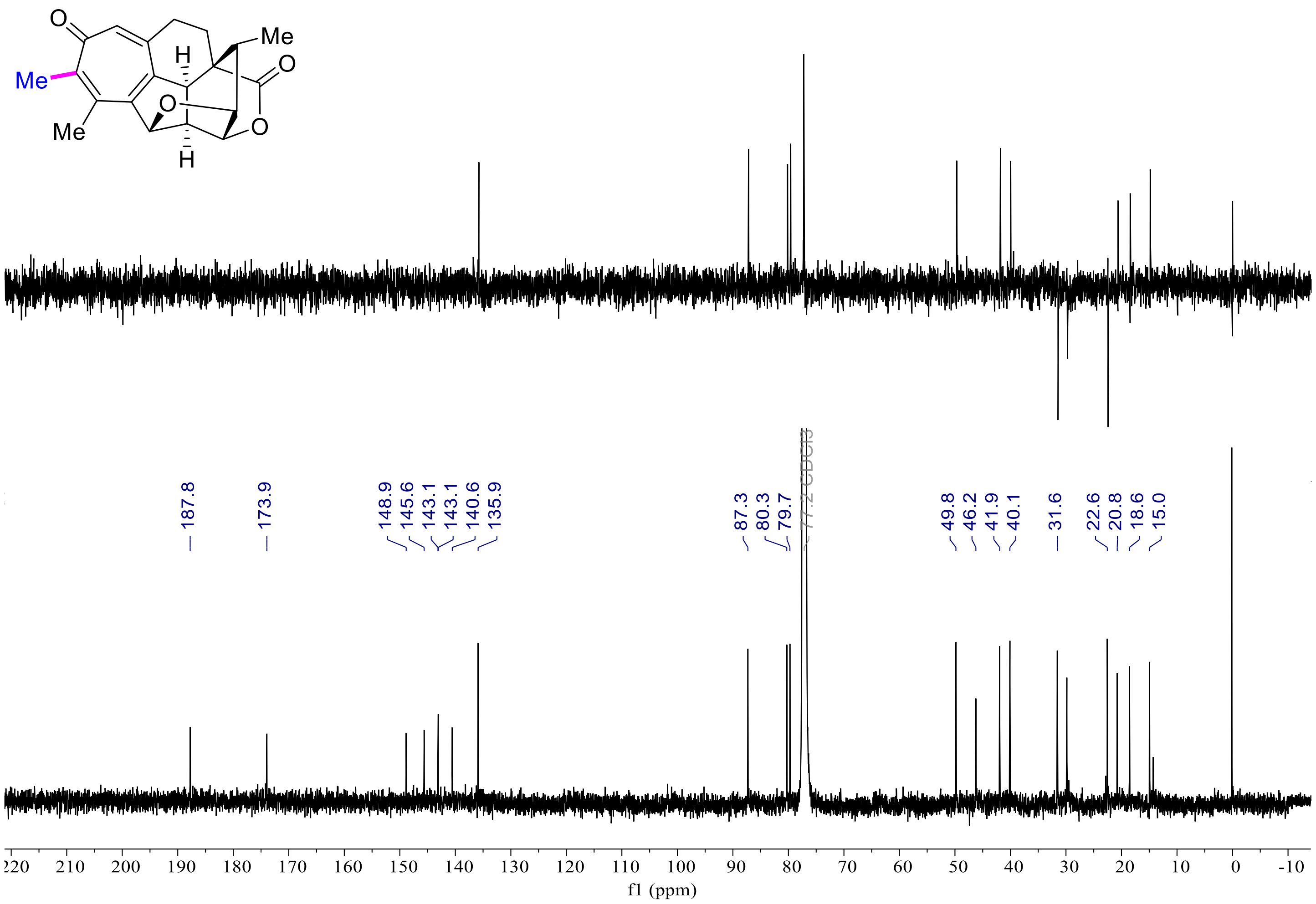

Compound 14 HRMS (ESI-TOF):

**Data Filename**	ESIH202404025.d	**Sample Name**	D4-ZQT15-1A
**Sample ID**		**Position**	P1-A2
**Instrument Name**	Agilent 6520 Q-TOF	**Acq Method**	20160322_MS_ESIH_POS_1min.m
**Acquired Time**	8/16/2024 2:51:26 PM	**DA Method**	ESI-HR-20231114.m
**Comment**	ESIH by fangsu		

User Spectra

Fragmentor Voltage	Collision Energy	Ionization Mode
170	0	ESI

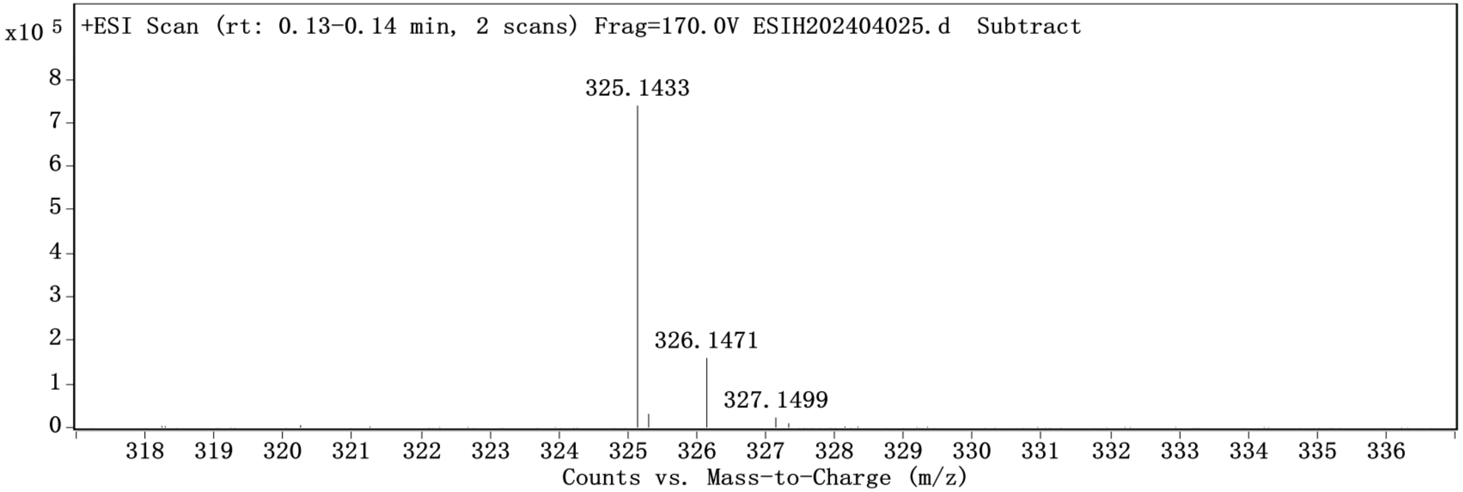

Formula Calculator Results

m/z	Calc m/z	Diff (mDa)	Diff (ppm)	Ion Formula	Ion
325.1433	325.1434	0.09	0.29	C20 H21 O4	(M+H)+

--- End Of Report ---

Compound 3 ^{1}H NMR (800 MHz, CDCl$_3$)

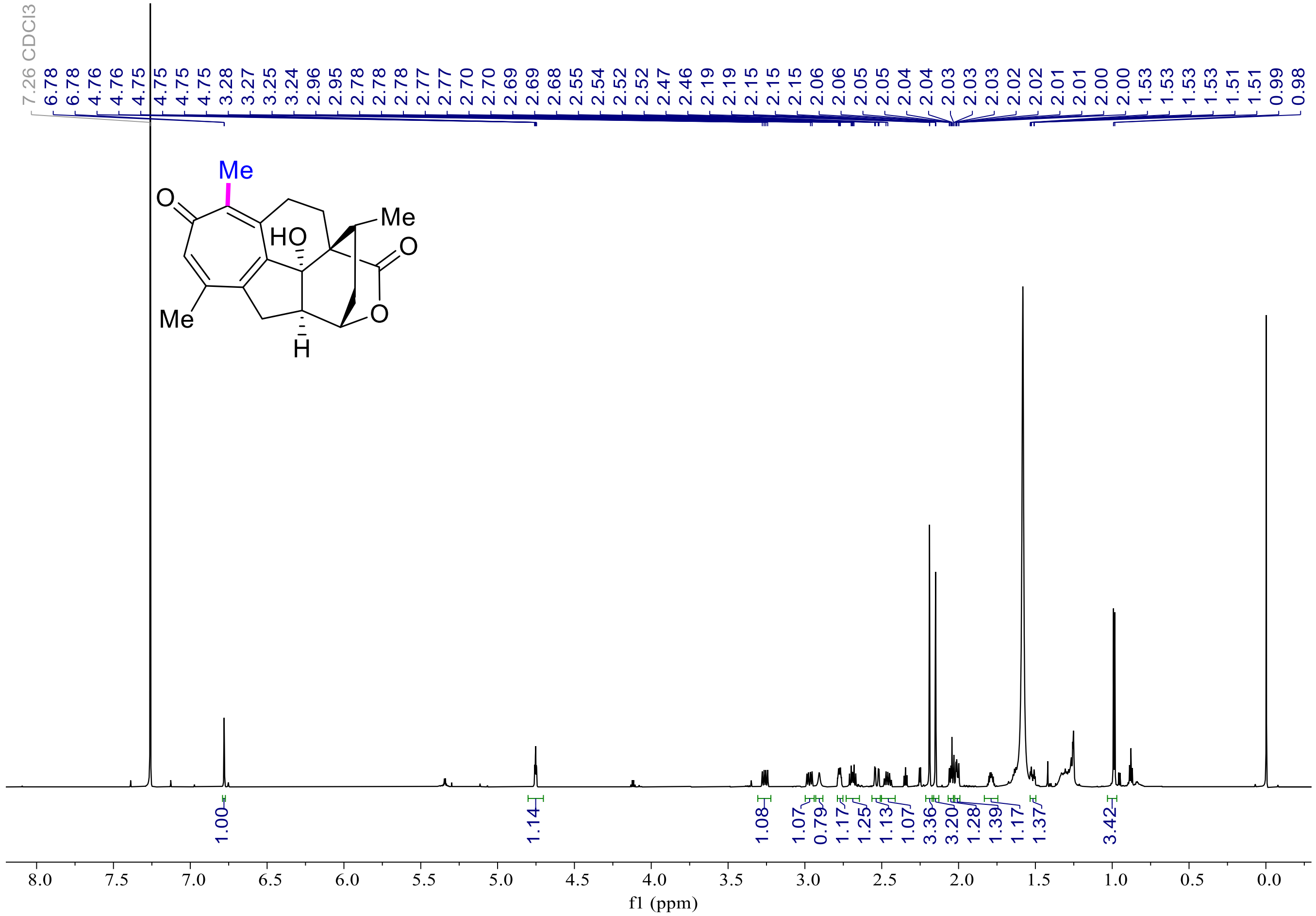

Compound 3 ^{13}C NMR (125 MHz, $CDCl_3$)

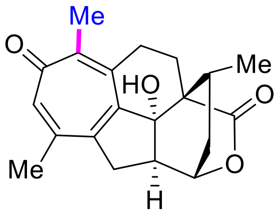

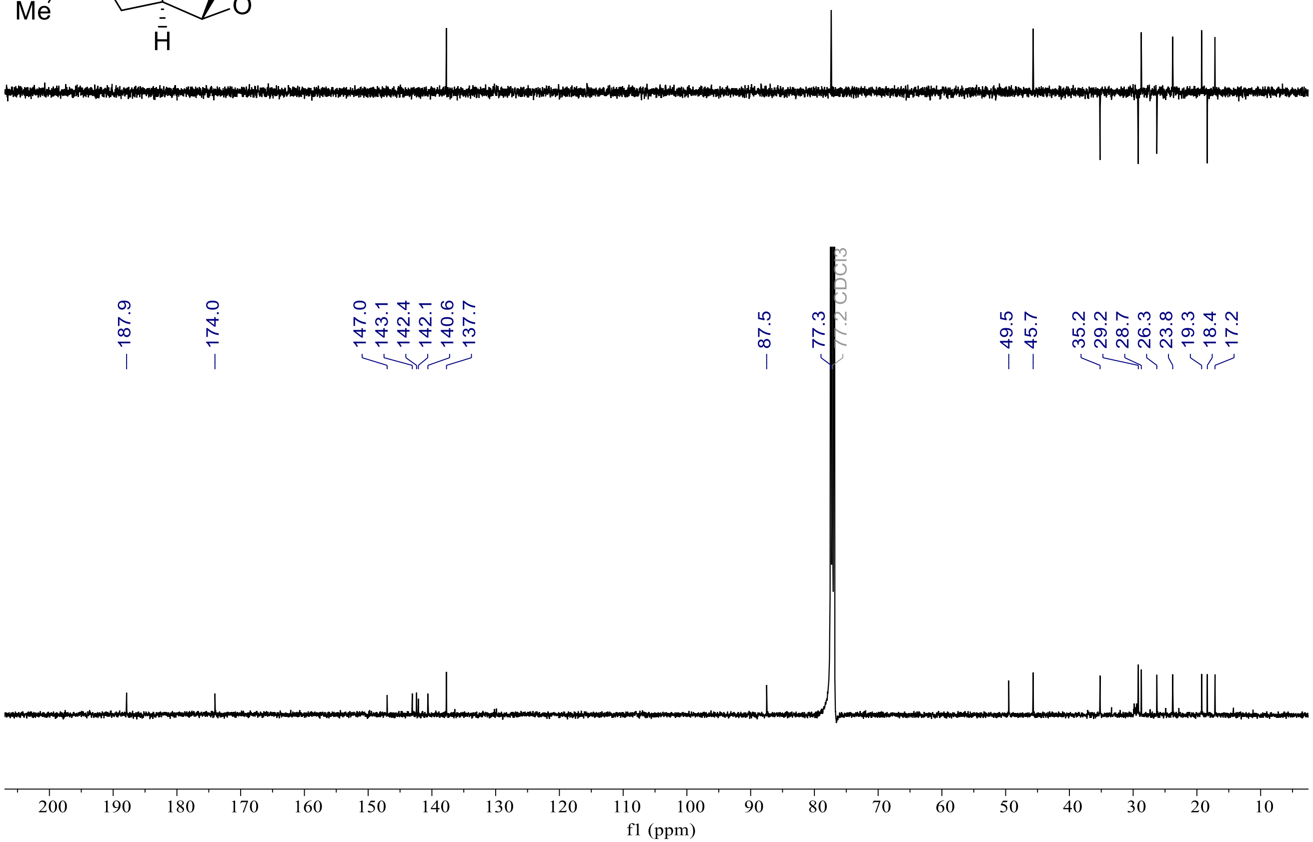

Compound 3 HRMS (ESI-TOF):

**Data Filename**	ESIH202404056.d	**Sample Name**	D4-ZQT17B
**Sample ID**		**Position**	P1-A2
**Instrument Name**	Agilent 6520 Q-TOF	**Acq Method**	20160322_MS_ESIH_POS_1min.m
**Acquired Time**	8/20/2024 3:03:24 PM	**DA Method**	ESI-HR-20231114.m
**Comment**	ESIH by fangsu		

User Spectra

**Fragmentor Voltage**	**Collision Energy**	**Ionization Mode**
170	0	ESI

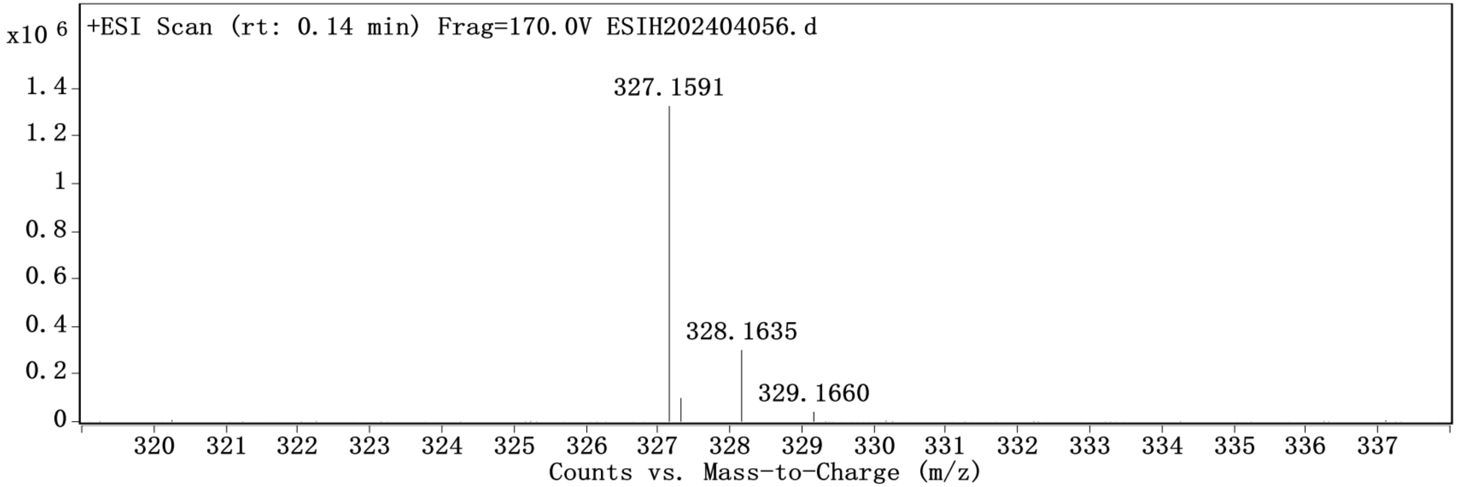

Formula Calculator Results

m/z	Calc m/z	Diff (mDa)	Diff (ppm)	Ion Formula	Ion
327.1591	327.1591	0.02	0.06	C20 H23 O4	(M+H)+

--- End Of Report ---

Compound 4 ^{1}H NMR (500 MHz, $CDCl_3$)

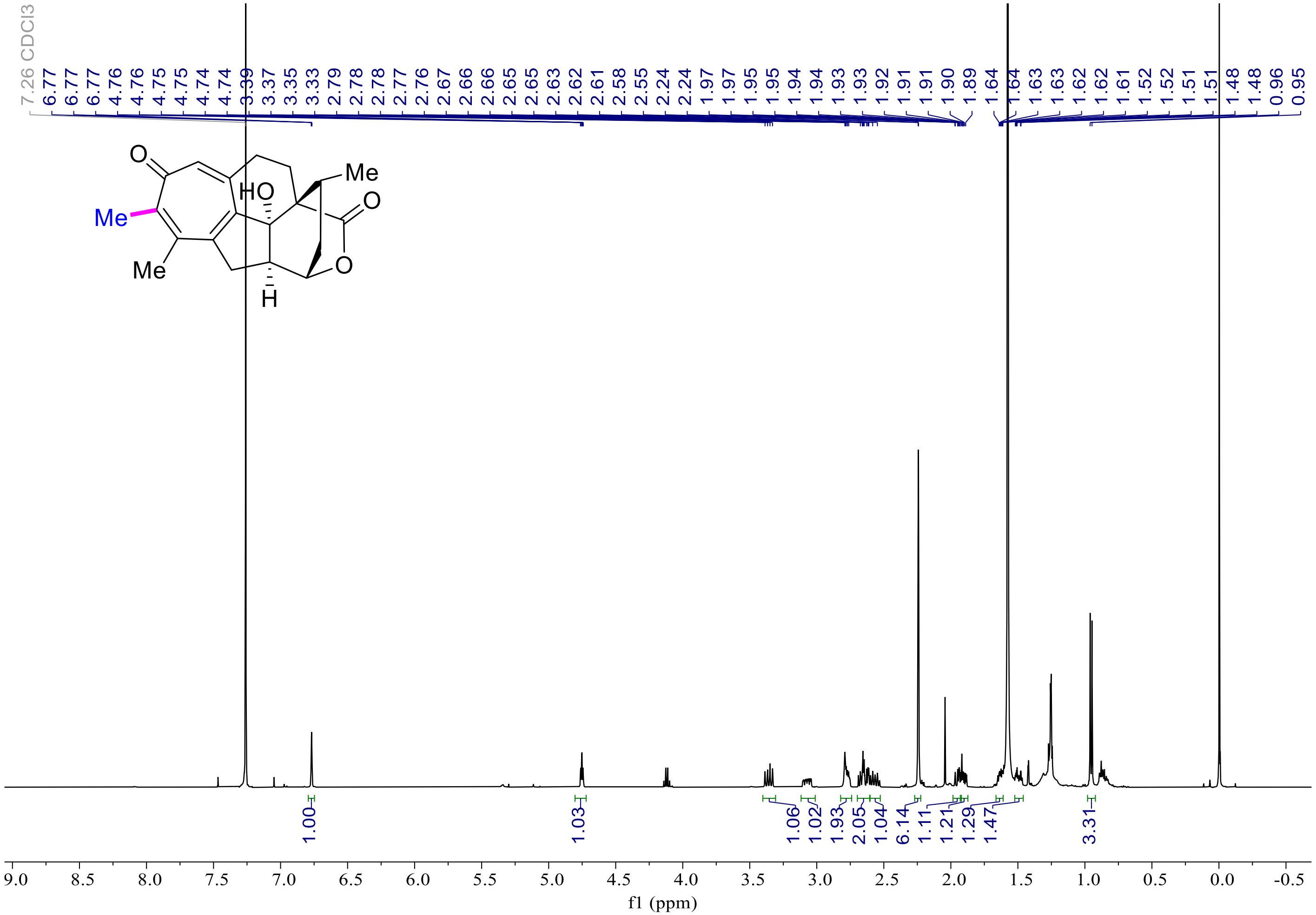

Compound 4 ^{13}C NMR (125 MHz, $CDCl_3$)

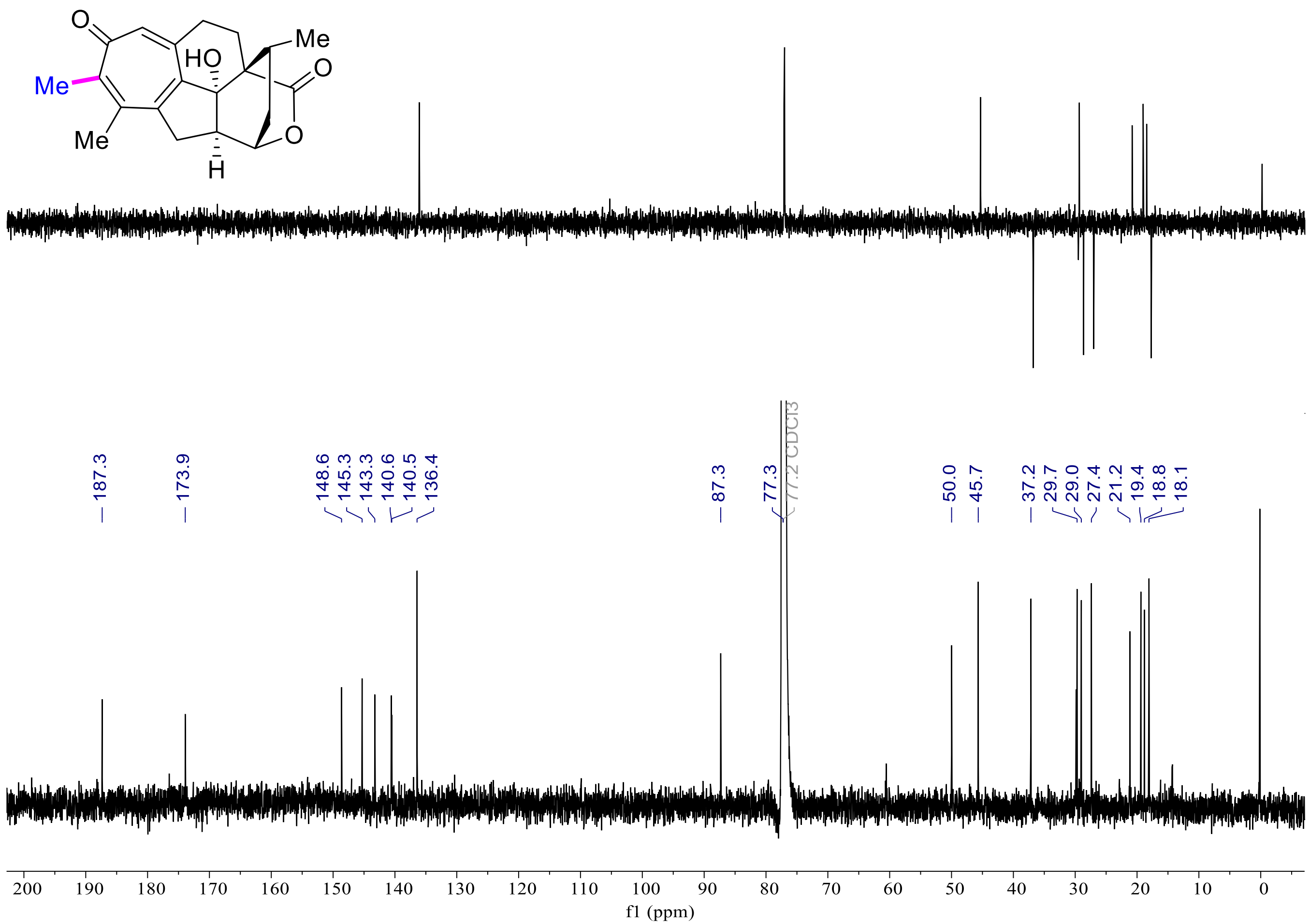

Compound 4 HRMS (ESI-TOF)

**Data Filename**	ESIH202404055.d	**Sample Name**	D4-ZQT17A
**Sample ID**		**Position**	P1-A1
**Instrument Name**	Agilent 6520 Q-TOF	**Acq Method**	20160322_MS_ESIH_POS_1min.m
**Acquired Time**	8/20/2024 3:02:08 PM	**DA Method**	ESI-HR-20231114.m
**Comment**	ESIH by fangsu		

User Spectra

Fragmentor Voltage	Collision Energy	Ionization Mode
170	0	ESI

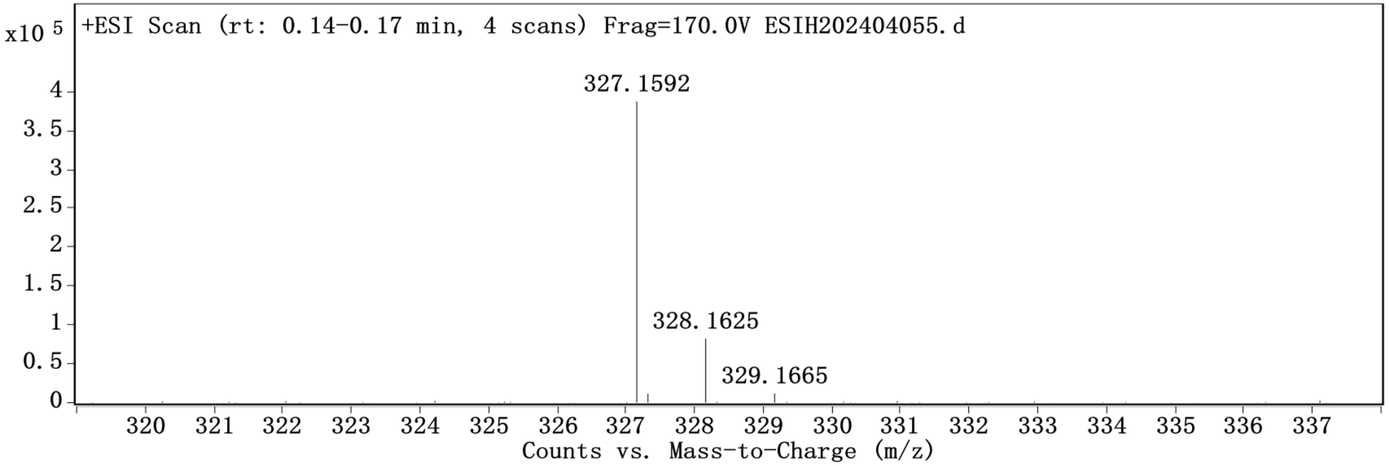

Formula Calculator Results

m/z	Calc m/z	Diff (mDa)	Diff (ppm)	Ion Formula	Ion
327.1592	327.1591	-0.11	-0.35	C20 H23 O4	(M+H)+

--- End Of Report ---

X-Ray Crystallography Data

Table S5 X-ray crystallography data for compound 4 (cephafortunoid B)

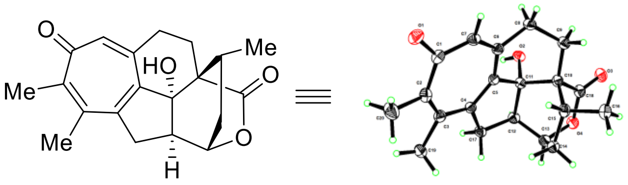

cephafortunoid B, **4**

Identification code	Cephafortunoid B, **4**
Empirical formula	$C_{20}H_{22}O_4$
Formula weight	326.37
Temperature/K	150
Crystal system	orthorhombic
Space group	$P2_12_12_1$
a/Å	8.3302(4)
b/Å	9.9529(4)
c/Å	19.2217(9)
α/°	90
β/°	90
γ/°	90
Volume/Å^3	1593.66(12)
Z	4
ρ_{calc}g/cm^3	1.360
μ/mm^{-1}	0.760
F(000)	696.0
Crystal size/mm^3	0.12 × 0.05 × 0.03
Radiation	CuKα (λ = 1.54178)
2Θ range for data collection/°	9.202 to 136.452
Index ranges	$-10 \leq h \leq 9$, $-11 \leq k \leq 11$, $-23 \leq l \leq 22$
Reflections collected	16939
Independent reflections	2893 [R_{int} = 0.0764, R_{sigma} = 0.0565]
Data/restraints/parameters	2893/0/229
Goodness-of-fit on F^2	1.086
Final R indexes [I>=2σ (I)]	R_1 = 0.0512, wR_2 = 0.1377
Final R indexes [all data]	R_1 = 0.0550, wR_2 = 0.1423
Largest diff. peak/hole / e Å^{-3}	0.29/-0.20
Flack parameter	0.06(13)

NMR Data Comparison of Synthetic and Natural Products

Table S6 ^{1}H-NMR data comparison for compound 3 (cephafortunoid A)

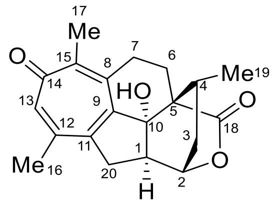

cephafortunoid A, **3**

Position	Cephafortunoid A isolated (600MHz)[16] δH (J in Hz)	Cephafortunoid A synthesized (800 MHz) δH (J in Hz)
1	2.82 m	2.78 dddd (9.8, 5.1, 3.4 1.8)
2	4.75 td (4.3, 1.1)	4.75 td (4.5, 1.3)
3	a 2.00 m; b 1.50 dtd (14.9, 4.3, 1.7)	a 2.01 m; b 1.52 dtd (14.8, 4.7, 1.8)
4	1.75 m	1.79 dqd (14.1, 7.0, 5.1)
6	α 2.72 dt (14.5, 8.9), β 2.03 m	α 2.69 m, β 2.05 m
7	α 2.95 dd (18.8, 8.5); β 2.43 dt (18.8, 8.9)	α 2.97 dd (18.7, 8.5); β 2.46 dt (18.3, 8.9)
13	6.72 d (0.9)	6.78 d (1.31)
16	2.17 d (0.9)	2.19 d (1.1)
17	2.09 s	2.15 s
19	0.98 d (7.0)	0.99 d (7.0)
20	α 3.27 dd (18.5, 9.7); β 2.51 dd (18.5, 3.0)	α 3.26 dd (18.5, 9.6); β 2.53 dd (18.6, 3.6)

Table S7 ^{13}C-NMR data comparison for compound 3 (cephafortunoid A)

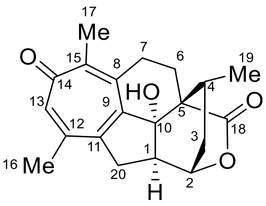

cephafortunoid A, **3**

Position	Cephafortunoid A isolated (150 MHz) δC	Cephafortunoid A synthesized (125 MHz) δC	△δ C
1	45.6	45.7	0.1
2	77.4	77.3	−0.1
3	29.2	29.2	0.0
4	28.7	28.7	0.0
5	49.5	49.5	0.0
6	18.4	18.4	0.0
7	26.5	26.3	−0.2
8	141.5	140.6	−0.9
9	142.7	142.1	−0.6
10	87.3	87.5	0.2
11	143.2	143.1	−0.1
12	142.8	142.4	−0.4
13	137.5	137.7	0.2
14	187.7	187.9	0.2
15	146.8	147.0	0.2
16	23.9	23.8	−0.1
17	17.1	17.2	0.1
18	174.1	174.0	−0.1
19	19.3	19.3	0.0
20	35.3	35.2	−0.1

Table S8 ^{1}H-NMR data comparison for compound 4 (cephafortunoid B)

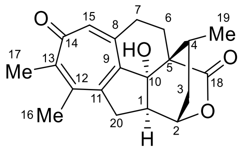

cephafortunoid B, **4**

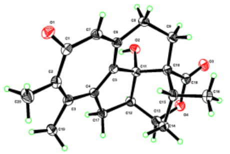

CCDC 2416905

Position	Cephafortunoid B isolated (600 MHz)[16] δH (J in Hz)	Cephafortunoid B synthesized (500 MHz) δH (J in Hz)
1	2.86 m	2.77 m, overlap
2	4.75 t (4.3)	4.75 td (4.6, 1.2)
3	a 1.92 m; b 1.48 dt (14.5, 4.3)	a 1.95 m; b 1.49 m
4	1.55 m	1.63 m
6	α 2.69 m, β 1.88 m	α 2.69 m, overlap ; β 1.90 m
7	α 3.09 ddd (17.7, 9.2, 2,6); β 2.55 dt (17.7, 7.5)	α 3.07 dddd (17.5, 9.3, 3.8, 1.6); β 2.56 m
15	6.83 s	6.77 t (1.33)
16	2.26 s	2.24 s, overlap
17	2.21 s	2.24 s, overlap
19	0.94 d (6.8)	0.95 d (7.0)
20	α 3.42 dd (18.3, 9.4); β 2.62 dd (18.3, 2.5)	α 3.36 dd (18.5, 9.5); β 2.65 m, overlap

Table S9 ^{13}C-NMR data comparison for compound 4 (cephafortunoid B)

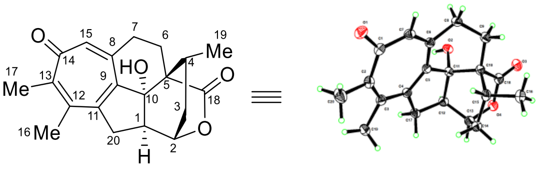

Position	Cephafortunoid B isolated (150 MHz) δC	Cephafortunoid B synthesized (125 MHz) δC	Δδ C
1	45.5	45.7	0.2
2	77.5	77.3	−0.2
3	29.1	29.0	−0.1
4	29.7	29.7	0.0
5	49.8	50.0	0.2
6	18.0	18.0	0.0
7	27.6	27.4	−0.2
8	145.0	143.3	−1.7
9	141.9	140.5	−1.4
10	86.7	87.3	0.6
11	146.2	145.3	−0.9
12	148.4	148.6	0.2
13	142.2	140.6	−1.6
14	186.4	187.3	0.9
15	136.2	136.4	0.2
16	21.5	21.2	−0.3
17	18.7	18.8	0.1
18	173.9	173.9	0.0
19	19.4	19.4	0.0
20	37.4	37.2	−0.2

Note: Some tiny differences resulted from the NMR equipment and the low concentration,[19,20] and the X-ray Crystallography Data in Table S5 unambiguously confirmed the synthetic structure.

NMR Calculations for Compound 8j and Its Isomer 8j′

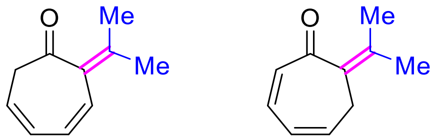

8j **8j'**

Due to the similarity in their chemical environments, it is challenging to differentiate between compounds **8j** and **8j′** based solely on NMR data. To further elucidate their structures, NMR calculations were conducted. Two isomers **8j** and **8j′** were performed by the conformational searches with the torsional sampling (Monte Carlo Multiple Minimum, MCMM) method under OPLS2013 force field by Macromodel 10.2 program (Schrödinger Release 2015-2: MacroModel, Schrödinger, LLC, New York, NY). The value of the "Energy window for saving structures" was set as 5.02 kcal/mol. All the candidate conformers were subjected to the GIAO NMR calculations at the RHF/STO-3G level in Gaussian 16 program package. The Gaussian output files, together with corresponding Excel file containing the experimental chemical shifts and key coupling constants and the labels of each nucleus associated with each experimental value, were subjected to the machine learning based Python program, ML_*J*DP4 (https://github.com/Sarotti-Lab/ML_*J*_DP4), according to the ML-*J*-DP4 guideline.[21-23] Cartesian coordinates of all conformers of isomer **8j** and **8j′** will be made available on request.

Table S10 ML-*J*-DP4 probabilities for candidate **8j** and its isomer **8j′**

	**Isomer 8j**	**Isomer 8j′**
DP4-H	99.95	0.05
DP4-C	100	0
DP4-HC	100	0

Table S11 Comparison of the experimental and calculated NMR for **8j** and **8j′**

**Exp.**	**Chemical shifts (unscaled)**				**Chemical shifts (scaled)**			
	**8j**	**errors**	**8j′**	**errors**	**8j**	**errors**	**8j′**	**errors**
125.6	123.0304	2.5696	123.7334	1.8666	125.0116	0.5884	126.8277	1.2277
125.4	116.223	9.177	123.2532	2.1468	118.0455	7.3545	126.3501	0.9501
128.3	123.9889	4.3111	122.1021	6.1979	125.9925	2.3075	125.205	3.095
123.6	125.6845	2.0845	136.5025	12.9025	127.7277	4.1277	139.5296	15.9296
45.5	44.1836	1.3164	28.1946	17.3054	44.3261	1.1739	31.7919	13.7081
134.8	132.8776	1.9224	128.5802	6.2198	135.0885	0.2885	131.6491	3.1509
194.5	192.349	2.151	190.7951	3.7049	195.9468	1.4468	193.5364	0.9636
152	152.3081	0.3081	143.699	8.301	154.9721	2.9721	146.6883	5.3117
24.5	27.2048	2.7048	26.1069	1.6069	26.9513	2.4513	29.7152	5.2152
24.2	24.651	0.451	23.4847	0.7153	24.338	0.138	27.1068	2.9068
6.47	6.7224	0.2524	6.2297	0.2403	6.6409	0.1709	6.0111	0.4589
6.09	5.7908	0.2992	6.503	0.413	5.8161	0.2739	6.2367	0.1467
6.18	5.9677	0.2123	6.0149	0.1651	5.9727	0.2073	5.8339	0.3461
5.82	6.1532	0.3332	6.9057	1.0857	6.1369	0.3169	6.569	0.749
3.18	2.8312	0.3488	2.8373	0.3427	3.1962	0.0162	3.2116	0.0316
3.18	2.8319	0.3481	2.8376	0.3424	3.1956	0.0156	3.2114	0.0314
2.17	1.467	0.703	1.283	0.887	1.9877	0.1823	1.9286	0.2414
2.04	1.6885	0.3515	1.5243	0.5157	2.1839	0.1439	2.1277	0.0877

Reaction Setup

Photochemical reaction was carried out under visible light irradiation by LEDs at 25ºC. Photosyn-10 manufactured by Shanghai Quanhuan Technology Co., Ltd was used in this system. Figure S7 shows the reaction setup with various wavelength.

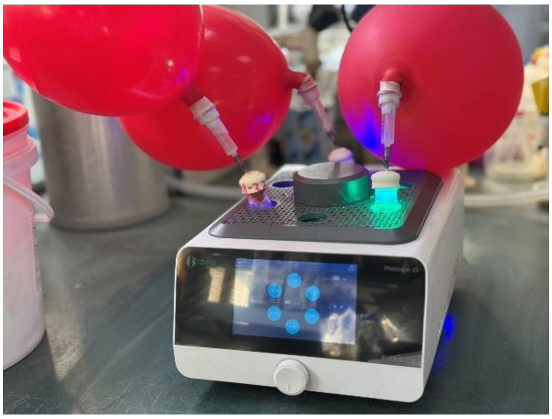

Figure S7 Picture of the reaction irradiation setup with various wavelength.

Quantum yield detection was carried out on HQI-ZX designed by the same company. Figure S8 shows the reaction setup for quantum yield detection (λ=455 nm).

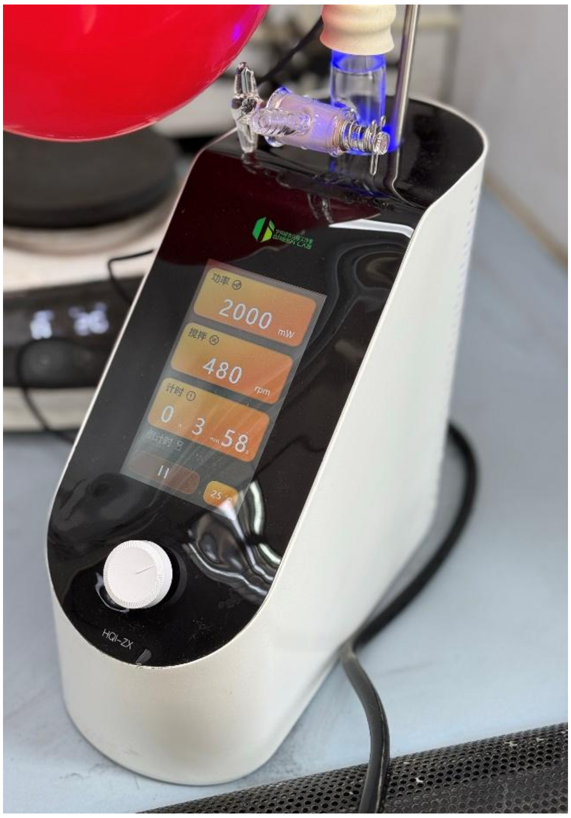

Figure S8 Picture of the reactor for quantum yield detection.

References

1. M. C. Barret, P. H. Bhatia, G. Kociok-Köhn and K. C. Molloy, *Transition Met. Chem.*, 2014, **39**, 543–551.
2. T. Nozoe, S. Ishikawa and K. Shindo, *Heterocycles*, 1989, **28**, 733–744.
3. L. Shen, J. Hu, H. Wang, A. Wang, Y. Lai and Y. Kang, *Chemical Research in Chinese Universities*, 2015, **31**, 367–371.
4. C. L. Barløse, J. Faghtmann, M. Kaasik, R. Mastroddi and K. A. Jørgensen, *Org. Lett.*, 2024, **26**, 1539–1543.
5. D. Haas, D. Sustac-Roman, S. Schwarz and P. Knochel, *Org. Lett.*, 2016, **18**, 6380–6383.
6. Z.-P. Ge, H.-C. Liu, G.-C. Wang, Q.-F. Liu, C.-H. Xu, J. Ding, Y.-Y. Fan and J.-M. Yue, *J. Nat. Prod.*, 2019, **82**, 1565–1575.
7. N. R. Kumar, A. R. Agrawal and S. S. Zade, *Chem. - Eur. J.*, 2019, **25**, 14064–14071.
8. S. N. Ononye, M. D. VanHeyst, E. Z. Oblak, W. Zhou, M. Ammar, A. C. Anderson and D. L. Wright, *ACS Med. Chem. Lett.*, 2013, **4**, 757–761.
9. Y. Zheng, E. H. Ghazvini Zadeh and Y. Yuan, *Eur. J. Org. Chem.*, 2016, **2016**, 2115–2119.
10. D. Filippini and M. Silvi, *Nat. Chem.*, 2022, **14**, 66–70.
11. D. I. Schuster, L. Wang and J. M. Van der Veen, *J. Am. Chem. Soc.*, 1985, **107**, 7045–7053.
12. N. C. M. Lubrin, A. Vlasceanu, B. N. Frandsen, A. B. Skov, M. D. Kilde, K. V. Mikkelsen and M. B. Nielsen, *Eur. J. Org. Chem.*, 2017, **2017**, 2932–2939.
13. R. Kato, T. Suzuki and K. Tanino, *Chem. Commun.*, 2024, **60**, 6619–6622.
14. A. Brunetti, M. Garbini, N. Gino Kub, M. Monari, R. Pedrazzani, C. Zanardi, G. Bertuzzi and M. Bandini, *Adv. Synth. Catal.*, 2024, **366**, 1965–1971.
15. Y. Shimizu, H. Morimoto, M. Zhang and T. Ohshima, *Angew. Chem. Int. Ed.*, 2012, **51**, 8564–8567.
16. Y. Li, Y. Wang, Z. Shao, C. Zhao, Q. Jing, D. Li, B. Lin, Y. Jing, Z. Li and H. Hua, *Bioorg. Chem.*, 2020, **103**, 104226.
17. M. Montalti, A. Credi, L. Prodi and M. T. Gandolfi, *Handbook of photochemistry*, CRC press, 2006.
18. X. Zhao, L. Bai, J. Li and X. Jiang, *J. Am. Chem. Soc.*, 2024, **146**, 11173–11180.
19. A. Mitra, P. J. Seaton, R. Ali Assarpour and T. Williamson, *Tetrahedron*, 1998, **54**, 15489–15498.
20. I. Katsuyama, A. A. Khalil, C. Dunbar and J. K. Zjawiony, *Spectrosc. Lett.*, 2003, **36**, 477–485.
21. Y.-H. Tsai, M. Amichetti, M. M. Zanardi, R. Grimson, A. H. Daranas and A. M. Sarotti, *Org. Lett.*, 2022, **24**, 7487–7491.
22. C.-P. Liu, Y. Gao, W.-J. Li, C.-H. Xu, Q.-F. Liu, J. Ding, J.-X. Zhao and J.-M. Yue, *Tetrahedron*, 2024, **156**, 133942.
23. B. Zhou, Q. Gong, Y. Fu, J.-S. Zhou, H.-Y. Zhang and J.-M. Yue, *Org. Lett.*, 2023, **25**, 1464–1469.
